# Supplementary figures and images for: Imputed gene associations identify replicable trans‐acting genes enriched in transcription pathways and complex traits
Source: Genet Epidemiol. 2019 Apr 4;43(6):596–608. doi: 10.1002/gepi.22205 (PMC6687523; doi:10.1002/gepi.22205)

**Whole Blood Replication Distribution**

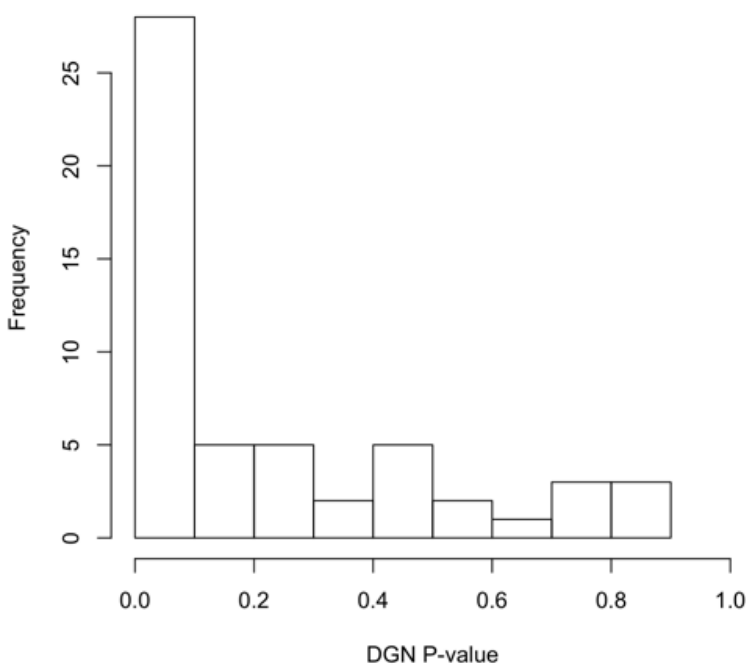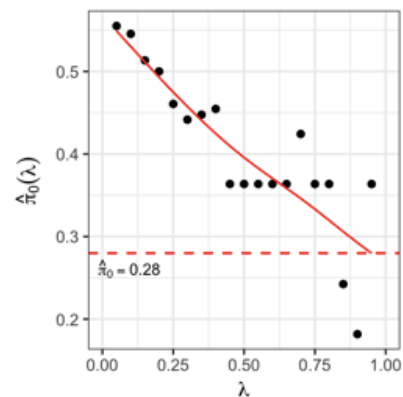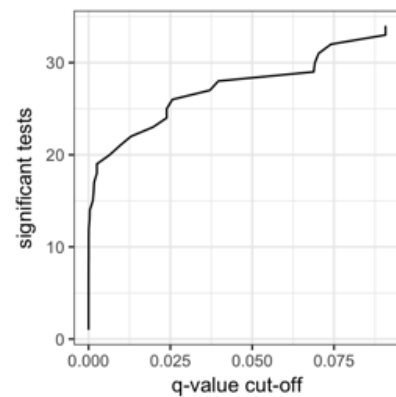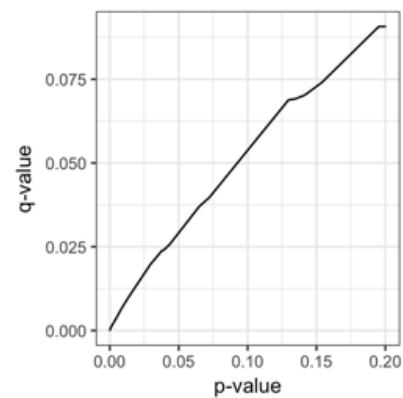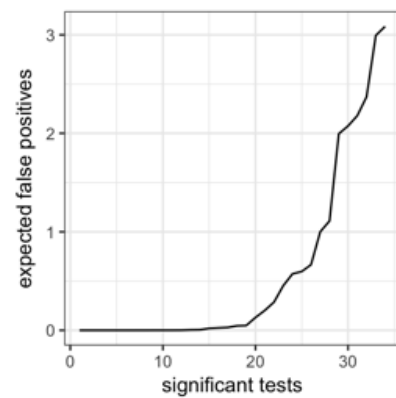

**Multi-tissue Replication Distribution**

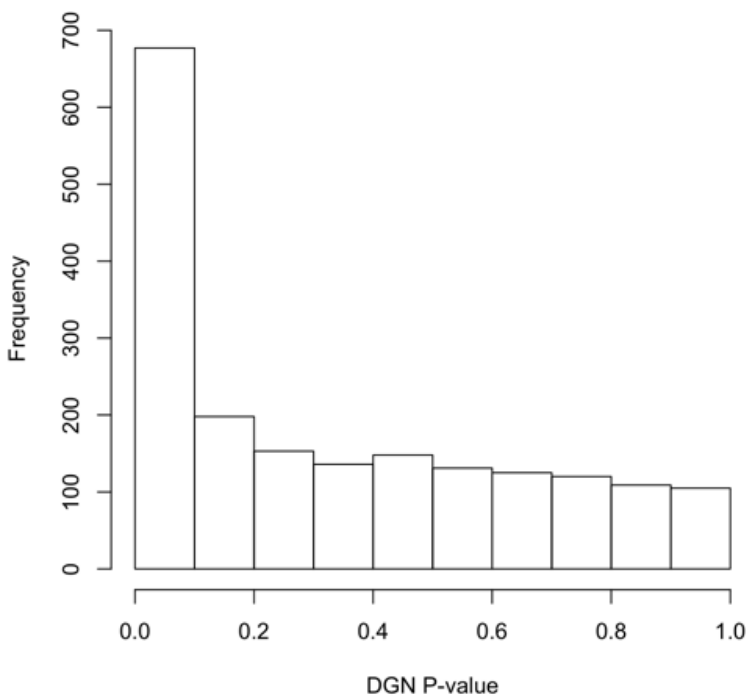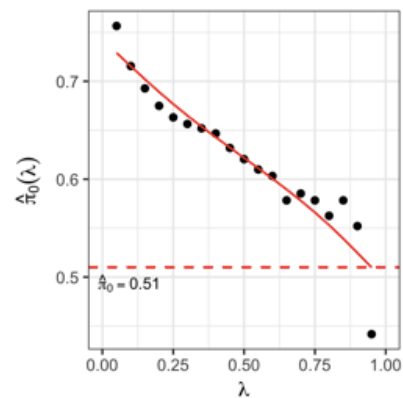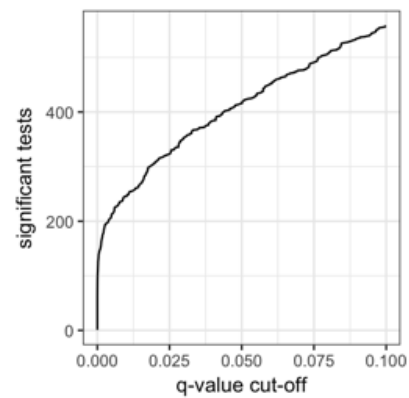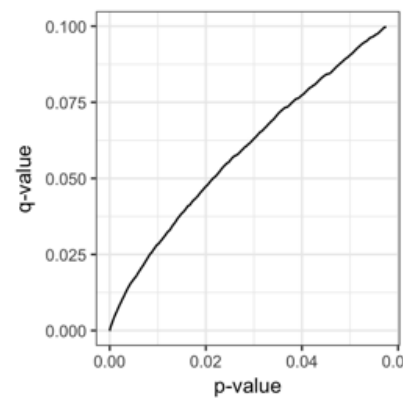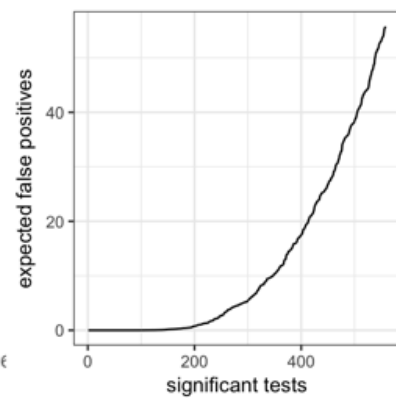

Supplement: Supplementary file 1 — Supplementary Information [file GEPI-43-596-s001.pdf]

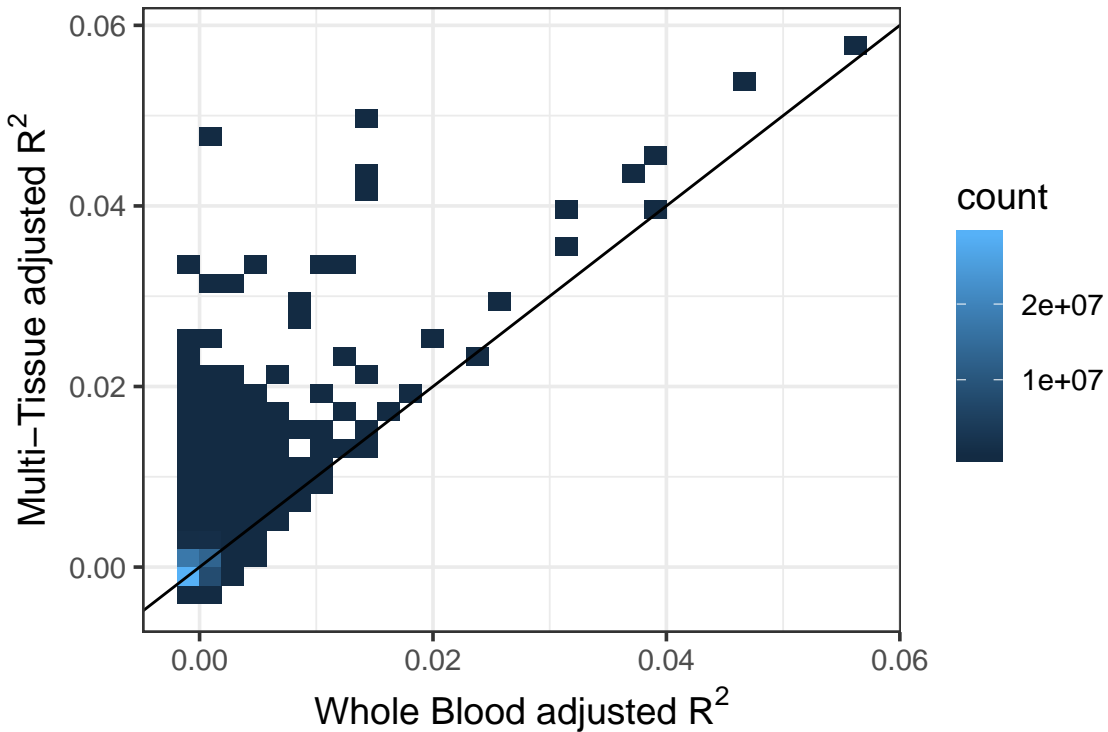

Supplement: Supplementary file 2 — Supplementary Information [file GEPI-43-596-s002.pdf]
