# Supplementary material for: Imputed gene associations identify replicable trans‐acting genes enriched in transcription pathways and complex traits
Source: Genet Epidemiol. 2019 Apr 4;43(6):596–608. doi: 10.1002/gepi.22205 (PMC6687523; doi:10.1002/gepi.22205)
Supplement: Supplementary file 3 — Supplementary Information [file GEPI-43-596-s003.pdf]

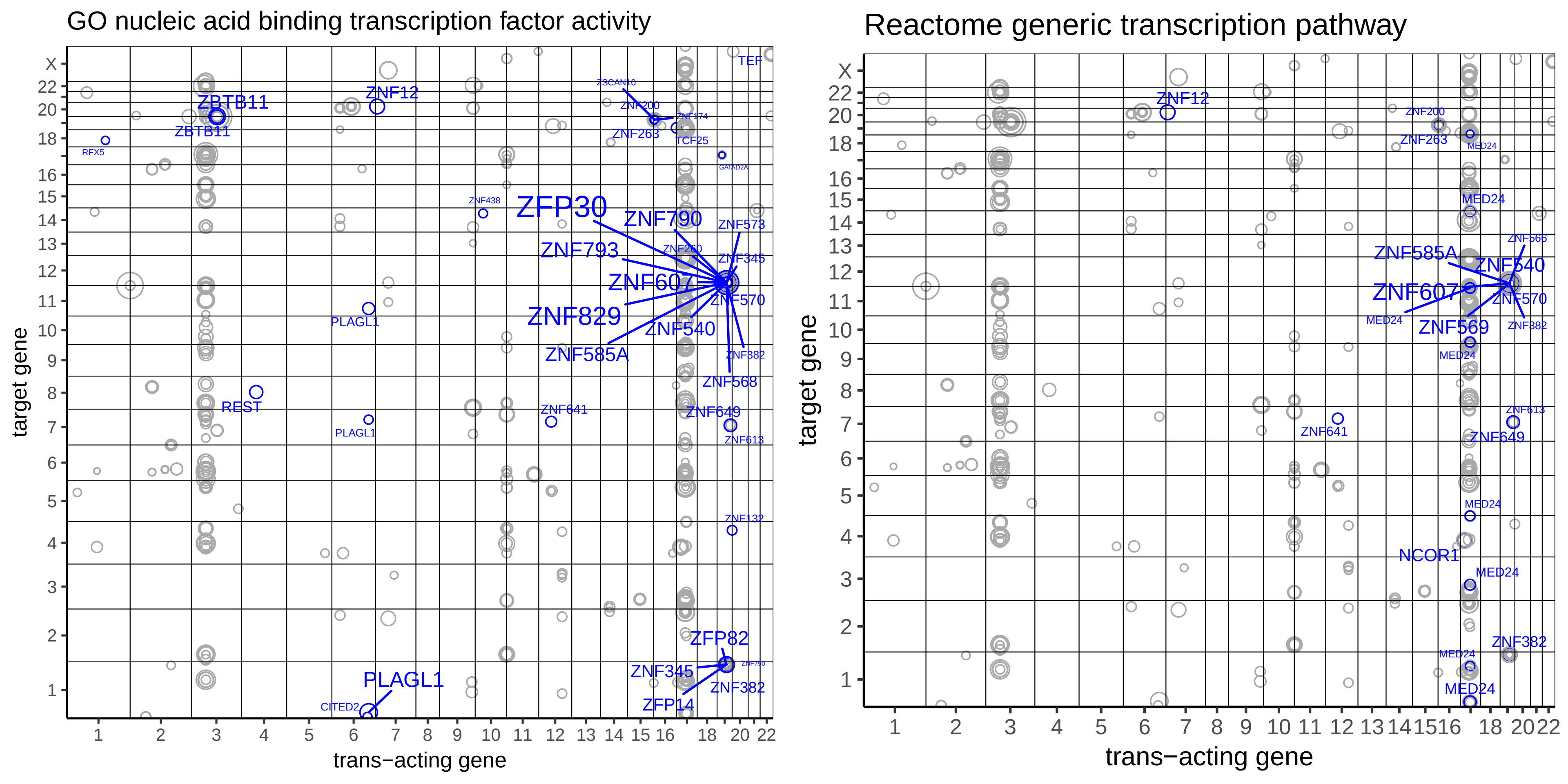
GO nucleic acid binding transcription factor activity
target gene
trans−acting gene
ZBTB11
ZBTB11
RFX5
ZNF12
ZSCAN10
ZNF200
ZNF174
ZNF263
TCF25
TEF
ZNF438
ZFP30
ZNF790
ZNF573
ZNF793
ZNF260
ZNF345
ZNF607
ZNF570
ZNF829
ZNF540
ZNF585A
ZNF382
ZNF568
PLAGL1
REST
PLAGL1
ZNF641
ZNF649
ZNF613
ZNF132
ZFP82
ZNF345
ZNF382
ZFP14
PLAGL1
CITED2
Reactome generic transcription pathway
target gene
trans−acting gene
ZNF12
ZNF200
ZNF263
MED24
MED24
ZNF585A
ZNF566
ZNF540
ZNF607
ZNF570
MED24
ZNF569
ZNF382
MED24
ZNF641
ZNF613
ZNF649
MED24
NCOR1
MED24
ZNF382
MED24
MED24
