# Supplementary material for: Imputed gene associations identify replicable trans‐acting genes enriched in transcription pathways and complex traits
Source: Genet Epidemiol. 2019 Apr 4;43(6):596–608. doi: 10.1002/gepi.22205 (PMC6687523; doi:10.1002/gepi.22205)
Supplement: Supplementary file 5 — Supplementary Information [file GEPI-43-596-s005.docx]

| predgene | predname | predChr | predS1 | predS2 | obsgene | obsname | obsChr | obsS1 | obsS2 | FHS_stat | FHS_beta | FHS_pval | FHS_FDR | DGN_stat | DGN_beta | DGN_pval | DGN_FDR | trans_eQTL_in_trans_eQTLGen | trans_eQTL_is_cis_eQTL |
| --- | --- | --- | --- | --- | --- | --- | --- | --- | --- | --- | --- | --- | --- | --- | --- | --- | --- | --- | --- |
| ENSG00000138468 | SENP7 | 3 | 101043049 | 101232085 | ENSG00000196724 | ZNF418 | 19 | 58433252 | 58446761 | 16.7079133 | 0.1996122 | 5.72E-61 | 1.38E-53 | 15.9758666 | 1.28047628 | 6.86E-51 | 3.70E-49 | No | NA |
| ENSG00000005020 | SKAP2 | 7 | 26706681 | 27034858 | ENSG00000176358 | TAC4 | 17 | 47915671 | 47925379 | 13.9539315 | 0.09278781 | 2.05E-43 | 2.48E-36 | -2.1811768 | -0.129578 | 0.02942293 | 0.94153366 | No | NA |
| ENSG00000105997 | HOXA3 | 7 | 27145803 | 27179844 | ENSG00000176358 | TAC4 | 17 | 47915671 | 47925379 | 13.7009384 | 0.7635341 | 6.03E-42 | 4.86E-35 | -2.0692859 | -1.0684776 | 0.038798 | 1 | No | NA |
| ENSG00000105996 | HOXA2 | 7 | 27139721 | 27142430 | ENSG00000176358 | TAC4 | 17 | 47915671 | 47925379 | 12.547298 | 0.18749007 | 1.46E-35 | 8.86E-29 | -2.3585315 | -0.3158826 | 0.01855571 | 0.61233833 | No | NA |
| ENSG00000105991 | HOXA1 | 7 | 27132612 | 27135615 | ENSG00000176358 | TAC4 | 17 | 47915671 | 47925379 | 11.4400719 | 0.18744678 | 6.38E-30 | 3.08E-23 | -2.4863173 | -0.3627281 | 0.01308374 | 0.44484713 | No | NA |
| ENSG00000112701 | SENP6 | 6 | 76311225 | 76427997 | ENSG00000124102 | PI3 | 20 | 43803517 | 43805185 | -9.0856468 | -0.1888231 | 1.47E-19 | 5.94E-13 | -6.3926792 | -0.3992014 | 2.59E-10 | 1.30E-08 | No | NA |
| ENSG00000166323 | C11orf65 | 11 | 108179246 | 108338258 | ENSG00000197409 | HIST1H3D | 6 | 26197068 | 26197497 | -8.2982681 | -3.8130237 | 1.36E-16 | 4.69E-10 | NA | NA | NA | NA | No | NA |
| ENSG00000136819 | C9orf78 | 9 | 132589569 | 132598142 | ENSG00000128294 | TPST2 | 22 | 26921458 | 26992681 | 8.14359523 | 0.04239587 | 4.84E-16 | 1.46E-09 | 1.51678484 | 0.08709421 | 0.12966453 | 1 | No | NA |
| ENSG00000149311 | ATM | 11 | 108094805 | 108239829 | ENSG00000197409 | HIST1H3D | 6 | 26197068 | 26197497 | 8.03972274 | 0.25561761 | 1.12E-15 | 3.02E-09 | 4.55506792 | 0.73740308 | 5.94E-06 | 0.00027918 | No | NA |
| ENSG00000136827 | TOR1A | 9 | 132575223 | 132586413 | ENSG00000128294 | TPST2 | 22 | 26921458 | 26992681 | 8.02451124 | 0.05701133 | 1.27E-15 | 3.07E-09 | 1.84707565 | 0.14599736 | 0.06505712 | 1 | No | NA |
| ENSG00000136816 | TOR1B | 9 | 132565432 | 132573560 | ENSG00000128294 | TPST2 | 22 | 26921458 | 26992681 | 8.01254624 | 0.05298352 | 1.40E-15 | 3.07E-09 | 1.42642672 | 0.1040303 | 0.15408446 | 1 | No | NA |
| ENSG00000181007 | ZFP82 | 19 | 36874022 | 36909558 | ENSG00000116962 | NID1 | 1 | 236139130 | 236228462 | -7.2407453 | -0.1052769 | 5.17E-13 | 1.04E-06 | -6.44326 | -0.8819081 | 1.88E-10 | 9.59E-09 | No | NA |
| ENSG00000139531 | SUOX | 12 | 56390964 | 56400425 | ENSG00000105085 | MED26 | 19 | 16698215 | 16739873 | -7.1511569 | -0.0832829 | 9.89E-13 | 1.84E-06 | -1.1621219 | -0.1398957 | 0.24548735 | 1 | Yes | Yes |
| ENSG00000128973 | CLN6 | 15 | 68499330 | 68549444 | ENSG00000180423 | HARBI1 | 11 | 46624411 | 46638777 | -7.0992082 | -0.0631732 | 1.44E-12 | 2.48E-06 | 2.64018224 | 0.22880619 | 0.00842647 | 0.29492659 | No | NA |
| ENSG00000138468 | SENP7 | 3 | 101043049 | 101232085 | ENSG00000196247 | ZNF107 | 7 | 64126511 | 64171404 | 7.07356977 | 0.12849575 | 1.73E-12 | 2.78E-06 | 4.01067857 | 0.36018724 | 6.55E-05 | 0.0028165 | No | NA |
| ENSG00000115232 | ITGA4 | 2 | 182321934 | 182400914 | ENSG00000096006 | CRISP3 | 6 | 49695097 | 49712150 | -6.9168185 | -0.1600721 | 5.22E-12 | 7.43E-06 | -2.9622122 | -0.3166897 | 0.00313295 | 0.11362056 | Yes | Yes |
| ENSG00000138468 | SENP7 | 3 | 101043049 | 101232085 | ENSG00000176293 | ZNF135 | 19 | 58570607 | 58593375 | 6.91880167 | 0.05894235 | 5.15E-12 | 7.43E-06 | 9.11541669 | 0.79081452 | 4.80E-19 | 2.54E-17 | No | NA |
| ENSG00000146576 | C7orf26 | 7 | 6629648 | 6648357 | ENSG00000124102 | PI3 | 20 | 43803517 | 43805185 | -6.7575354 | -0.3678996 | 1.57E-11 | 2.11E-05 | -4.1882248 | -0.7024332 | 3.08E-05 | 0.0014076 | No | NA |
| ENSG00000144488 | ESPNL | 2 | 239008798 | 239041928 | ENSG00000088854 | C20orf194 | 20 | 3229951 | 3388272 | 6.6955039 | 0.32290625 | 2.39E-11 | 3.05E-05 | -0.9630256 | -0.1601551 | 0.33578774 | 1 | No | NA |
| ENSG00000123636 | BAZ2B | 2 | 160175490 | 160473203 | ENSG00000146426 | TIAM2 | 6 | 155181872 | 155575707 | -6.6015694 | -0.0981678 | 4.51E-11 | 5.45E-05 | -3.5898523 | -0.4544739 | 0.00034835 | 0.01428243 | No | NA |
| ENSG00000118495 | PLAGL1 | 6 | 144261437 | 144385735 | ENSG00000135378 | PRRG4 | 11 | 32851489 | 32879669 | -6.5939245 | -0.169646 | 4.74E-11 | 5.46E-05 | -3.1644544 | -0.3591377 | 0.00160478 | 0.0625863 | No | NA |
| ENSG00000188511 | C22orf34 | 22 | 49808176 | 50051190 | ENSG00000149575 | SCN2B | 11 | 118032666 | 118047388 | -6.4447779 | -0.0584856 | 1.27E-10 | 0.00013964 | 0.7884381 | 0.06420086 | 0.4306436 | 1 | No | NA |
| ENSG00000188452 | CERKL | 2 | 182401403 | 182521843 | ENSG00000096006 | CRISP3 | 6 | 49695097 | 49712150 | -6.4174529 | -0.124211 | 1.52E-10 | 0.00015963 | -3.1183553 | -0.2898046 | 0.0018752 | 0.07125756 | Yes | Yes |
| ENSG00000188511 | C22orf34 | 22 | 49808176 | 50051190 | ENSG00000154319 | FAM167A | 8 | 11278972 | 11332224 | -6.3123531 | -0.058562 | 2.99E-10 | 0.00030167 | -0.1938187 | -0.0157873 | 0.84636064 | 1 | No | NA |
| ENSG00000176024 | ZNF613 | 19 | 52430400 | 52452012 | ENSG00000164659 | KIAA1324L | 7 | 86506222 | 86689015 | -6.2070919 | -0.3024265 | 5.85E-10 | 0.00056568 | -4.650438 | -1.363852 | 3.80E-06 | 0.0001824 | No | NA |
| ENSG00000123636 | BAZ2B | 2 | 160175490 | 160473203 | ENSG00000116991 | SIPA1L2 | 1 | 232533711 | 232697304 | -6.0040181 | -0.1102538 | 2.07E-09 | 0.00192105 | -2.9684328 | -0.376626 | 0.00307083 | 0.11362056 | No | NA |
| ENSG00000145217 | SLC26A1 | 4 | 972861 | 987228 | ENSG00000132740 | IGHMBP2 | 11 | 68671310 | 68708067 | -5.9623787 | -0.5432229 | 2.66E-09 | 0.00238453 | 1.28109852 | 0.1138528 | 0.20048189 | 1 | No | NA |
| ENSG00000174885 | NLRP6 | 11 | 278365 | 285359 | ENSG00000138722 | MMRN1 | 4 | 90800683 | 90875780 | -5.9495735 | -0.1411022 | 2.88E-09 | 0.00248526 | 0.26930119 | 0.04137353 | 0.78775831 | 1 | No | NA |
| ENSG00000112701 | SENP6 | 6 | 76311225 | 76427997 | ENSG00000124107 | SLPI | 20 | 43880880 | 43883205 | -5.9026447 | -0.1383465 | 3.82E-09 | 0.00318626 | -5.9913498 | -0.3751112 | 2.98E-09 | 1.46E-07 | No | NA |
| ENSG00000132436 | FIGNL1 | 7 | 50511831 | 50518088 | ENSG00000013583 | HEBP1 | 12 | 13127798 | 13153207 | 5.84546925 | 0.16683113 | 5.38E-09 | 0.00424124 | 1.47104305 | 0.19128282 | 0.14162142 | 1 | No | NA |
| ENSG00000004534 | RBM6 | 3 | 49977440 | 50114683 | ENSG00000163357 | DCST1 | 1 | 155006300 | 155021740 | 5.84378292 | 0.02375915 | 5.44E-09 | 0.00424124 | -0.4556584 | -0.0243566 | 0.64874313 | 1 | No | NA |
| ENSG00000126391 | FRMD8 | 11 | 65154070 | 65180996 | ENSG00000188886 | ASTL | 2 | 96789589 | 96804175 | 5.8053275 | 0.21321408 | 6.83E-09 | 0.00516419 | 0.92280902 | 0.29942911 | 0.35634868 | 1 | No | NA |
| ENSG00000123636 | BAZ2B | 2 | 160175490 | 160473203 | ENSG00000111261 | MANSC1 | 12 | 12482198 | 12503475 | -5.7725755 | -0.1560294 | 8.29E-09 | 0.0060775 | -8.6064791 | -1.055513 | 3.22E-17 | 1.67E-15 | No | NA |
| ENSG00000164078 | MST1R | 3 | 49924435 | 49936696 | ENSG00000163357 | DCST1 | 1 | 155006300 | 155021740 | 5.76542747 | 0.16744963 | 8.65E-09 | 0.00615252 | -0.3674086 | -0.1505192 | 0.71339877 | 1 | No | NA |
| ENSG00000073605 | GSDMB | 17 | 38060848 | 38074903 | ENSG00000131873 | CHSY1 | 15 | 101715928 | 101792137 | 5.73620621 | 0.04150463 | 1.03E-08 | 0.00709618 | 1.05789963 | 0.07557682 | 0.29037889 | 1 | Yes | Yes |
| ENSG00000172057 | ORMDL3 | 17 | 38077294 | 38083854 | ENSG00000131873 | CHSY1 | 15 | 101715928 | 101792137 | 5.69542073 | 0.03988704 | 1.30E-08 | 0.00875521 | 1.13239146 | 0.07747998 | 0.25776494 | 1 | Yes | Yes |
| ENSG00000204516 | MICB | 6 | 31462658 | 31478901 | ENSG00000134765 | DSC1 | 18 | 28709199 | 28742819 | -5.6370882 | -0.0429099 | 1.83E-08 | 0.01194443 | -1.8003855 | -0.1977278 | 0.07212725 | 1 | No | NA |
| ENSG00000151500 | THYN1 | 11 | 134118173 | 134123264 | ENSG00000002933 | TMEM176A | 7 | 150498622 | 150502208 | -5.5775347 | -0.3183178 | 2.57E-08 | 0.01595418 | -1.1828389 | -0.2372291 | 0.23717859 | 1 | No | NA |
| ENSG00000104728 | ARHGEF10 | 8 | 1772142 | 1906807 | ENSG00000187017 | ESPN | 1 | 6484848 | 6521040 | -5.5774722 | -0.1716408 | 2.57E-08 | 0.01595418 | 0.83271051 | 0.23738361 | 0.40522413 | 1 | No | NA |
| ENSG00000128274 | A4GALT | 22 | 43088127 | 43117304 | ENSG00000213988 | ZNF90 | 19 | 20188803 | 20237885 | -5.5290568 | -0.064156 | 3.39E-08 | 0.02048587 | -0.7646406 | -0.0527709 | 0.44468167 | 1 | No | NA |
| ENSG00000033800 | PIAS1 | 15 | 68346517 | 68481608 | ENSG00000180423 | HARBI1 | 11 | 46624411 | 46638777 | -5.5231593 | -0.1123238 | 3.50E-08 | 0.02066472 | 1.29618881 | 0.25244553 | 0.1952355 | 1 | No | NA |
| ENSG00000172057 | ORMDL3 | 17 | 38077294 | 38083854 | ENSG00000112053 | SLC26A8 | 6 | 35911291 | 35992645 | 5.50767622 | 0.06400128 | 3.82E-08 | 0.02201717 | 0.82986427 | 0.0567989 | 0.4068308 | 1 | No | NA |
| ENSG00000076003 | MCM6 | 2 | 136597196 | 136633996 | ENSG00000132386 | SERPINF1 | 17 | 1665253 | 1680868 | -5.4873837 | -0.0946703 | 4.29E-08 | 0.02356176 | -3.232656 | -0.5817762 | 0.00127007 | 0.05080296 | No | NA |
| ENSG00000174885 | NLRP6 | 11 | 278365 | 285359 | ENSG00000049323 | LTBP1 | 2 | 33172039 | 33624576 | -5.4908213 | -0.174046 | 4.21E-08 | 0.02356176 | -0.1669205 | -0.0256451 | 0.86746928 | 1 | No | NA |
| ENSG00000196526 | AFAP1 | 4 | 7760441 | 7874040 | ENSG00000158747 | NBL1 | 1 | 19969726 | 19974164 | 5.47480484 | 0.0238643 | 4.60E-08 | 0.02472516 | 1.49659776 | 0.0823433 | 0.13484092 | 1 | No | NA |
| ENSG00000139572 | GPR84 | 12 | 54756229 | 54758271 | ENSG00000049323 | LTBP1 | 2 | 33172039 | 33624576 | 5.46503383 | 0.28843136 | 4.86E-08 | 0.02554988 | 3.66406643 | 0.9053171 | 0.00026243 | 0.0110221 | No | NA |
| ENSG00000073605 | GSDMB | 17 | 38060848 | 38074903 | ENSG00000112053 | SLC26A8 | 6 | 35911291 | 35992645 | 5.45635164 | 0.06551403 | 5.10E-08 | 0.0262518 | 0.53868425 | 0.03850117 | 0.59023506 | 1 | No | NA |
| ENSG00000109762 | SNX25 | 4 | 186125391 | 186291339 | ENSG00000149575 | SCN2B | 11 | 118032666 | 118047388 | 5.43525429 | 0.15702094 | 5.74E-08 | 0.02891897 | -0.6465143 | -0.173998 | 0.51810759 | 1 | No | NA |
| ENSG00000119408 | NEK6 | 9 | 127019885 | 127115586 | ENSG00000213085 | CCDC19 | 1 | 159842154 | 159869953 | 5.42887227 | 0.07843762 | 5.95E-08 | 0.02934238 | 4.17550406 | 0.47020878 | 3.26E-05 | 0.0014344 | No | NA |
| ENSG00000075856 | SART3 | 12 | 108916357 | 108955176 | ENSG00000169413 | RNASE6 | 14 | 21249210 | 21250626 | -5.4253152 | -0.2181682 | 6.07E-08 | 0.02934238 | 0.70989218 | 0.11037645 | 0.47795089 | 1 | No | NA |
| ENSG00000111639 | MRPL51 | 12 | 6601150 | 6602212 | ENSG00000100079 | LGALS2 | 22 | 37966255 | 37978623 | 5.40455765 | 0.68727835 | 6.81E-08 | 0.0316621 | -0.3788524 | -0.1019757 | 0.70488488 | 1 | No | NA |
| ENSG00000177700 | POLR2L | 11 | 842414 | 842545 | ENSG00000169413 | RNASE6 | 14 | 21249210 | 21250626 | 5.40773158 | 3.7228877 | 6.69E-08 | 0.0316621 | -0.2518053 | -0.0289668 | 0.80124782 | 1 | No | NA |
| ENSG00000139899 | CBLN3 | 14 | 24895738 | 24897611 | ENSG00000125534 | PPDPF | 20 | 62152077 | 62153559 | -5.3711624 | -0.0562151 | 8.19E-08 | 0.03736451 | -2.0238627 | -0.1284839 | 0.04327289 | 1 | No | NA |
| ENSG00000115866 | DARS | 2 | 136664247 | 136743670 | ENSG00000132386 | SERPINF1 | 17 | 1665253 | 1680868 | -5.3517099 | -0.0759585 | 9.12E-08 | 0.04081688 | -4.1895115 | -0.6510872 | 3.06E-05 | 0.0014076 | No | NA |
| ENSG00000182257 | C22orf26 | 22 | 46445358 | 46447779 | ENSG00000172322 | CLEC12A | 12 | 10124014 | 10138194 | -5.3198277 | -0.3598004 | 1.09E-07 | 0.04772459 | -2.0785738 | -0.3103906 | 0.03793321 | 1 | No | NA |
