# Supplementary material for: Imputed gene associations identify replicable trans‐acting genes enriched in transcription pathways and complex traits
Source: Genet Epidemiol. 2019 Apr 4;43(6):596–608. doi: 10.1002/gepi.22205 (PMC6687523; doi:10.1002/gepi.22205)
Supplement: Supplementary file 6 — Supplementary Information [file GEPI-43-596-s006.docx]

| predgene | predname | predChr | predS1 | predS2 | obsgene | obsname | obsChr | obsS1 | obsS2 | FHS_stat | FHS_beta | FHS_pval | FHS_FDR | DGN_stat | DGN_beta | DGN_pval | rsid | eQTLGen_trans_P | eQTLGen_trans_FDR | eQTLGen_cis_P | eQTLGen_cis_FDR |
| --- | --- | --- | --- | --- | --- | --- | --- | --- | --- | --- | --- | --- | --- | --- | --- | --- | --- | --- | --- | --- | --- |
| ENSG00000139531 | SUOX | 12 | 56390964 | 56400425 | ENSG00000105085 | MED26 | 19 | 16698215 | 16739873 | -7.1511569 | -0.0832829 | 9.89E-13 | 1.84E-06 | -1.1621219 | -0.1398957 | 0.24548735 | rs10876864 | 6.01E-06 | 0.03846264 | 3.2717e-310 | 0 |
| ENSG00000115232 | ITGA4 | 2 | 182321934 | 182400914 | ENSG00000096006 | CRISP3 | 6 | 49695097 | 49712150 | -6.9168185 | -0.1600721 | 5.22E-12 | 7.43E-06 | -2.9622122 | -0.3166897 | 0.00313295 | rs1375493 | 1.23E-27 | 0 | 3.2717e-310 | 0 |
| ENSG00000115232 | ITGA4 | 2 | 182321934 | 182400914 | ENSG00000096006 | CRISP3 | 6 | 49695097 | 49712150 | -6.9168185 | -0.1600721 | 5.22E-12 | 7.43E-06 | -2.9622122 | -0.3166897 | 0.00313295 | rs2124440 | 8.36E-27 | 0 | 3.2717e-310 | 0 |
| ENSG00000115232 | ITGA4 | 2 | 182321934 | 182400914 | ENSG00000096006 | CRISP3 | 6 | 49695097 | 49712150 | -6.9168185 | -0.1600721 | 5.22E-12 | 7.43E-06 | -2.9622122 | -0.3166897 | 0.00313295 | rs1449263 | 6.73E-26 | 0 | 3.2717e-310 | 0 |
| ENSG00000188452 | CERKL | 2 | 182401403 | 182521843 | ENSG00000096006 | CRISP3 | 6 | 49695097 | 49712150 | -6.4174529 | -0.124211 | 1.52E-10 | 0.00015963 | -3.1183553 | -0.2898046 | 0.0018752 | rs1375493 | 1.23E-27 | 0 | 3.47E-111 | 0 |
| ENSG00000188452 | CERKL | 2 | 182401403 | 182521843 | ENSG00000096006 | CRISP3 | 6 | 49695097 | 49712150 | -6.4174529 | -0.124211 | 1.52E-10 | 0.00015963 | -3.1183553 | -0.2898046 | 0.0018752 | rs2124440 | 8.36E-27 | 0 | 6.92E-104 | 0 |
| ENSG00000188452 | CERKL | 2 | 182401403 | 182521843 | ENSG00000096006 | CRISP3 | 6 | 49695097 | 49712150 | -6.4174529 | -0.124211 | 1.52E-10 | 0.00015963 | -3.1183553 | -0.2898046 | 0.0018752 | rs1449263 | 6.73E-26 | 0 | 1.10E-101 | 0 |
| ENSG00000073605 | GSDMB | 17 | 38060848 | 38074903 | ENSG00000131873 | CHSY1 | 15 | 101715928 | 101792137 | 5.73620621 | 0.04150463 | 1.03E-08 | 0.00709618 | 1.05789963 | 0.07557682 | 0.29037889 | rs12946510 | 2.91E-06 | 0.02114821 | 3.2717e-310 | 0 |
| ENSG00000073605 | GSDMB | 17 | 38060848 | 38074903 | ENSG00000131873 | CHSY1 | 15 | 101715928 | 101792137 | 5.73620621 | 0.04150463 | 1.03E-08 | 0.00709618 | 1.05789963 | 0.07557682 | 0.29037889 | rs4794820 | 1.44E-06 | 0.01124255 | 3.2717e-310 | 0 |
| ENSG00000073605 | GSDMB | 17 | 38060848 | 38074903 | ENSG00000131873 | CHSY1 | 15 | 101715928 | 101792137 | 5.73620621 | 0.04150463 | 1.03E-08 | 0.00709618 | 1.05789963 | 0.07557682 | 0.29037889 | rs4795397 | 7.15E-07 | 0.00616682 | 3.2717e-310 | 0 |
| ENSG00000172057 | ORMDL3 | 17 | 38077294 | 38083854 | ENSG00000131873 | CHSY1 | 15 | 101715928 | 101792137 | 5.69542073 | 0.03988704 | 1.30E-08 | 0.00875521 | 1.13239146 | 0.07747998 | 0.25776494 | rs2872507 | 5.39E-06 | 0.03496901 | 3.2717e-310 | 0 |
| ENSG00000172057 | ORMDL3 | 17 | 38077294 | 38083854 | ENSG00000131873 | CHSY1 | 15 | 101715928 | 101792137 | 5.69542073 | 0.03988704 | 1.30E-08 | 0.00875521 | 1.13239146 | 0.07747998 | 0.25776494 | rs8069176 | 2.38E-06 | 0.01771265 | 3.2717e-310 | 0 |
