# Supplementary material for: Imputed gene associations identify replicable trans‐acting genes enriched in transcription pathways and complex traits
Source: Genet Epidemiol. 2019 Apr 4;43(6):596–608. doi: 10.1002/gepi.22205 (PMC6687523; doi:10.1002/gepi.22205)
Supplement: Supplementary file 7 — Supplementary Information [file GEPI-43-596-s007.pdf]

predgene	predname	predChr	predS1	predS2	obsgene	obsname	obsChr	obsS1	obsS2	FHS_stat	FHS_beta	FHS_pval	FHS_FDR	DGN_stat	DGN_beta	DGN_pval	DGN_FDR	trans_eQTL_in_trans_eQTLGen	trans_eQTL_is_cis_eQTL
ENSG00000004534	RBM6	3	49977440	50114683	ENSG00000103023	PRSS54	16	58318309	58328951	0.057762	148.2	3.41E-63	6.99E-55	NA	NA	NA	NA	No	NA
ENSG00000004534	RBM6	3	49977440	50114683	ENSG00000134595	SOX3	X	139585152	139587225	0.053094	135.55	5.27E-58	5.40E-50	NA	NA	NA	NA	No	NA
ENSG00000228008	CTD-2330K9.3	3	49943495	49954370	ENSG00000103023	PRSS54	16	58318309	58328951	0.053295	90.711	4.16E-57	2.84E-49	NA	NA	NA	NA	No	NA
ENSG00000188511	C22orf34	22	49808176	50051190	ENSG00000258986	TMEM179	14	104941015	105071984	0.054092	55.264	5.08E-56	2.61E-48	NA	NA	NA	NA	No	NA
ENSG00000228008	CTD-2330K9.3	3	49943495	49954370	ENSG00000134595	SOX3	X	139585152	139587225	0.051795	88.019	1.88E-55	7.72E-48	NA	NA	NA	NA	No	NA
ENSG00000164078	MST1R	3	49924435	49936696	ENSG00000103023	PRSS54	16	58318309	58328951	0.049849	63.39	2.63E-52	8.98E-45	NA	NA	NA	NA	No	NA
ENSG00000138468	SENP7	3	101043049	101232085	ENSG00000196724	ZNF418	19	58433252	58446761	0.048676	82.446	5.13E-52	1.50E-44	0.2036	78.228	4.57E-45	8.69E-42	No	NA
ENSG00000090857	PDPR	16	70147529	70195203	ENSG00000255833	TIFAB	5	134787899	134788089	0.050325	42.667	5.11E-51	1.31E-43	0.0066502	0.87414	0.52636	1	No	NA
ENSG00000001617	SEMA3F	3	50192478	50226508	ENSG00000134595	SOX3	X	139585152	139587225	0.049294	50.108	9.02E-51	2.05E-43	NA	NA	NA	NA	No	NA
ENSG00000164076	CAMKV	3	49895422	49907655	ENSG00000103023	PRSS54	16	58318309	58328951	0.04655	78.669	1.11E-49	2.27E-42	NA	NA	NA	NA	No	NA
ENSG00000164078	MST1R	3	49924435	49936696	ENSG00000134595	SOX3	X	139585152	139587225	0.046333	58.702	1.84E-48	3.43E-41	NA	NA	NA	NA	No	NA
ENSG00000001617	SEMA3F	3	50192478	50226508	ENSG00000103023	PRSS54	16	58318309	58328951	0.046945	47.602	3.26E-48	5.57E-41	NA	NA	NA	NA	No	NA
ENSG00000181019	NQO1	16	69740899	69760854	ENSG00000255833	TIFAB	5	134787899	134788089	0.049767	25.281	2.79E-47	4.40E-40	0.0064673	0.59301	0.82055	1	No	NA
ENSG00000005020	SKAP2	7	26706681	27034858	ENSG00000176358	TAC4	17	47915671	47925379	0.048243	27.192	2.20E-46	3.21E-39	0.010753	1.1015	0.35883	1	No	NA
ENSG00000183763	TRAIP	3	49866034	49894007	ENSG00000103023	PRSS54	16	58318309	58328951	0.044014	55.629	6.19E-46	8.46E-39	NA	NA	NA	NA	No	NA
ENSG00000105997	HOXA3	7	27145803	27179844	ENSG00000176358	TAC4	17	47915671	47925379	0.043945	55.537	7.36E-46	9.43E-39	0.005358	1.2349	0.29436	1	No	NA
ENSG00000151883	PARP8	5	49961733	50142356	ENSG00000240386	LCE1F	1	152748848	152749445	0.04422	37.252	2.08E-44	2.51E-37	NA	NA	NA	NA	No	NA
ENSG00000173402	DAG1	3	49506146	49573048	ENSG00000134595	SOX3	X	139585152	139587225	0.042401	53.5	3.51E-44	3.99E-37	NA	NA	NA	NA	No	NA
ENSG00000243477	NAT6	3	50333833	50335514	ENSG00000103023	PRSS54	16	58318309	58328951	0.043861	36.936	5.08E-44	5.48E-37	NA	NA	NA	NA	No	NA
ENSG00000186792	HYAL3	3	50331063	50333204	ENSG00000103023	PRSS54	16	58318309	58328951	0.045901	23.222	3.66E-43	3.75E-36	NA	NA	NA	NA	No	NA
ENSG00000114378	HYAL1	3	50337320	50349812	ENSG00000134595	SOX3	X	139585152	139587225	0.040878	51.496	1.58E-42	1.54E-35	NA	NA	NA	NA	No	NA
ENSG00000182179	UBA7	3	49842640	49851379	ENSG00000103023	PRSS54	16	58318309	58328951	0.040488	50.984	4.16E-42	3.88E-35	NA	NA	NA	NA	No	NA
ENSG00000228008	CTD-2330K9.3	3	49943495	49954370	ENSG00000100228	RAB36	22	23487513	23506537	0.039556	66.362	4.79E-42	4.09E-35	0.0025883	0.59491	0.6664	1	No	NA
ENSG00000075407	ZNF37A	10	38383264	38412276	ENSG00000176200	OR4D11	11	59271049	59271984	0.042039	35.334	4.64E-42	4.09E-35	NA	NA	NA	NA	No	NA
ENSG00000186792	HYAL3	3	50331063	50333204	ENSG00000134595	SOX3	X	139585152	139587225	0.044632	22.55	8.10E-42	6.64E-35	NA	NA	NA	NA	No	NA
ENSG00000164068	RNF123	3	49728563	49753910	ENSG00000103023	PRSS54	16	58318309	58328951	0.03924	65.812	1.05E-41	8.31E-35	NA	NA	NA	NA	No	NA
ENSG00000145022	TCTA	3	49452253	49453908	ENSG00000103023	PRSS54	16	58318309	58328951	0.039268	49.384	8.72E-41	6.62E-34	NA	NA	NA	NA	No	NA
ENSG00000183763	TRAIP	3	49866034	49894007	ENSG00000134595	SOX3	X	139585152	139587225	0.039211	49.31	1.00E-40	7.35E-34	NA	NA	NA	NA	No	NA
ENSG00000164077	MON1A	3	49946302	49967606	ENSG00000103023	PRSS54	16	58318309	58328951	0.038322	64.21	1.05E-40	7.41E-34	NA	NA	NA	NA	No	NA
ENSG00000164076	CAMKV	3	49895422	49907655	ENSG00000134595	SOX3	X	139585152	139587225	0.037935	63.535	2.76E-40	1.89E-33	NA	NA	NA	NA	No	NA
ENSG00000238243	OR2W3	1	248058859	248060449	ENSG00000166086	JAM3	11	133938820	134018713	0.044029	18.519	7.89E-40	5.22E-33	0.062774	5.0736	3.09E-08	5.73E-05	Yes	Yes
ENSG00000114378	HYAL1	3	50337320	50349812	ENSG00000103023	PRSS54	16	58318309	58328951	0.03798	47.701	2.15E-39	1.38E-32	NA	NA	NA	NA	No	NA
ENSG00000001617	SEMA3F	3	50192478	50226508	ENSG00000100228	RAB36	22	23487513	23506537	0.038552	38.751	3.85E-39	2.39E-32	0.0019818	0.36379	0.87344	1	No	NA
ENSG00000173402	DAG1	3	49506146	49573048	ENSG00000103023	PRSS54	16	58318309	58328951	0.037707	47.345	4.23E-39	2.55E-32	NA	NA	NA	NA	No	NA
ENSG00000243477	NAT6	3	50333833	50335514	ENSG00000134595	SOX3	X	139585152	139587225	0.039127	32.787	6.15E-39	3.60E-32	NA	NA	NA	NA	No	NA
ENSG00000185614	FAM212A	3	49840687	49842463	ENSG00000103023	PRSS54	16	58318309	58328951	0.036117	60.377	2.59E-38	1.47E-31	NA	NA	NA	NA	No	NA
ENSG00000114388	NPRL2	3	50384919	50387453	ENSG00000103023	PRSS54	16	58318309	58328951	0.034688	86.873	8.58E-38	4.75E-31	NA	NA	NA	NA	No	NA
ENSG00000145022	TCTA	3	49452253	49453908	ENSG00000134595	SOX3	X	139585152	139587225	0.035729	44.769	5.74E-37	3.10E-30	NA	NA	NA	NA	No	NA
ENSG00000114388	NPRL2	3	50384919	50387453	ENSG00000134595	SOX3	X	139585152	139587225	0.033086	82.722	4.73E-36	2.49E-29	NA	NA	NA	NA	No	NA
ENSG00000088543	C3orf18	3	50595462	50605182	ENSG00000134595	SOX3	X	139585152	139587225	0.035509	35.579	7.06E-36	3.62E-29	NA	NA	NA	NA	No	NA
ENSG00000088543	C3orf18	3	50595462	50605182	ENSG00000103023	PRSS54	16	58318309	58328951	0.035133	35.189	1.78E-35	8.89E-29	NA	NA	NA	NA	No	NA
ENSG00000164078	MST1R	3	49924435	49936696	ENSG00000100228	RAB36	22	23487513	23506537	0.034302	42.918	1.96E-35	9.58E-29	0.0093046	2.1531	0.072451	1	No	NA
ENSG00000108733	PEX12	17	33901814	33905882	ENSG00000158856	DMTN	8	21906506	21940038	0.034814	34.857	3.91E-35	1.86E-28	NA	NA	NA	NA	Yes	No
ENSG00000105996	HOXA2	7	27139721	27142430	ENSG00000176358	TAC4	17	47915671	47925379	0.037572	20.942	4.51E-35	2.10E-28	0.011063	1.7059	0.11644	1	No	NA
ENSG00000214706	IFRD2	3	50325840	50329487	ENSG00000103023	PRSS54	16	58318309	58328951	0.033595	42.002	1.13E-34	5.14E-28	NA	NA	NA	NA	No	NA
ENSG00000006125	AP2B1	17	33913918	34053436	ENSG00000158856	DMTN	8	21906506	21940038	0.035769	25.596	1.35E-34	6.00E-28	NA	NA	NA	NA	Yes	Yes
ENSG00000214706	IFRD2	3	50325840	50329487	ENSG00000134595	SOX3	X	139585152	139587225	0.033348	41.683	2.07E-34	9.04E-28	NA	NA	NA	NA	No	NA
ENSG00000090861	AARS	16	70286198	70323446	ENSG00000255833	TIFAB	5	134787899	134788089	0.034696	28.94	3.25E-34	1.39E-27	0.0016812	0.30852	0.90796	1	No	NA
ENSG00000205045	SLFN12L	17	33800708	33864880	ENSG00000158856	DMTN	8	21906506	21940038	0.03533	25.27	3.93E-34	1.64E-27	NA	NA	NA	NA	Yes	Yes
ENSG00000164076	CAMKV	3	49895422	49907655	ENSG00000100228	RAB36	22	23487513	23506537	0.031865	53.035	1.01E-33	4.16E-27	0.0029168	0.89516	0.44312	1	No	NA
ENSG00000225399	RP11-3B7.1	3	49297518	49298744	ENSG00000103023	PRSS54	16	58318309	58328951	0.031836	52.985	1.09E-33	4.38E-27	NA	NA	NA	NA	No	NA
ENSG00000182179	UBA7	3	49842640	49851379	ENSG00000134595	SOX3	X	139585152	139587225	0.032611	40.73	1.28E-33	5.05E-27	NA	NA	NA	NA	No	NA
ENSG00000176095	IP6K1	3	49761727	49823975	ENSG00000103023	PRSS54	16	58318309	58328951	0.032404	40.463	2.13E-33	8.25E-27	NA	NA	NA	NA	No	NA
ENSG00000225399	RP11-3B7.1	3	49297518	49298744	ENSG00000134595	SOX3	X	139585152	139587225	0.031283	52.036	4.29E-33	1.63E-26	NA	NA	NA	NA	No	NA
ENSG00000183763	TRAIP	3	49866034	49894007	ENSG00000100228	RAB36	22	23487513	23506537	0.032075	40.039	4.80E-33	1.79E-26	0.0083761	1.9364	0.10232	1	No	NA
ENSG00000108733	PEX12	17	33901814	33905882	ENSG00000070182	SPTB	14	65216372	65346601	0.032337	32.295	1.71E-32	6.27E-26	0.0055639	1.025	0.40152	1	No	NA
ENSG00000186792	HYAL3	3	50331063	50333204	ENSG00000100228	RAB36	22	23487513	23506537	0.035723	17.882	1.80E-32	6.47E-26	0.0019354	1.784	0.18199	1	No	NA
ENSG00000182504	CEP97	3	101442769	101489406	ENSG00000196724	ZNF418	19	58433252	58446761	0.032911	27.401	2.54E-32	8.98E-26	0.097719	16.516	3.88E-18	7.37E-15	No	NA
ENSG00000120784	ZFP30	19	38121910	38157163	ENSG00000111291	GPRC5D	12	13093709	13105081	0.034031	21.266	4.75E-32	1.65E-25	0.041956	4.9979	4.40E-06	0.0079816	No	NA
ENSG00000164077	MON1A	3	49946302	49967606	ENSG00000134595	SOX3	X	139585152	139587225	0.030054	49.928	9.00E-32	3.07E-25	NA	NA	NA	NA	No	NA
ENSG00000176095	IP6K1	3	49761727	49823975	ENSG00000134595	SOX3	X	139585152	139587225	0.030878	38.497	9.17E-32	3.08E-25	NA	NA	NA	NA	No	NA
ENSG00000185614	FAM212A	3	49840687	49842463	ENSG00000134595	SOX3	X	139585152	139587225	0.029848	49.574	1.50E-31	4.97E-25	NA	NA	NA	NA	No	NA
ENSG00000214706	IFRD2	3	50325840	50329487	ENSG00000100228	RAB36	22	23487513	23506537	0.03043	37.921	2.76E-31	8.97E-25	0.004203	0.77325	0.5691	1	No	NA

ENSG00000163947	ARHGEF3	3	56761446	57113357	ENSG00000259207	ITGB3	17	45387505	45389182	0.033203	20.731	3.49E-31	1.12E-24	0.071601	8.8017	1.20E-11	2.27E-08	No	NA
ENSG00000105991	HOXA1	7	27132612	27135615	ENSG00000176358	TAC4	17	47915671	47925379	0.02917	48.414	8.04E-31	2.53E-24	0.0076574	2.3612	0.069999	1	No	NA
ENSG00000081148	IMPG2	3	100941390	101039404	ENSG00000196724	ZNF418	19	58433252	58446761	0.032689	20.398	1.20E-30	3.74E-24	0.15421	20.808	3.82E-29	7.26E-26	No	NA
ENSG00000205045	SLFN12L	17	33800708	33864880	ENSG00000070182	SPTB	14	65216372	65346601	0.031795	22.659	2.08E-30	6.36E-24	0.021045	2.8069	0.0067753	1	No	NA
ENSG00000006125	AP2B1	17	33913918	34053436	ENSG00000070182	SPTB	14	65216372	65346601	0.03116	22.192	9.63E-30	2.90E-23	0.016031	2.4846	0.021708	1	No	NA
ENSG00000213380	COG8	16	69357429	69372480	ENSG00000255833	TIFAB	5	134787899	134788089	0.029029	28.892	5.56E-29	1.65E-22	0.001139	0.20891	0.9588	1	No	NA
ENSG00000164068	RNF123	3	49728563	49753910	ENSG00000134595	SOX3	X	139585152	139587225	0.027252	45.142	9.17E-29	2.68E-22	NA	NA	NA	NA	No	NA
ENSG00000173421	CCDC36	3	49235861	49295537	ENSG00000103023	PRSS54	16	58318309	58328951	0.028029	34.843	1.00E-28	2.90E-22	NA	NA	NA	NA	No	NA
ENSG00000243477	NAT6	3	50333833	50335514	ENSG00000100228	RAB36	22	23487513	23506537	0.029093	24.126	2.71E-28	7.71E-22	0.0070447	1.0819	0.37133	1	No	NA
ENSG00000108733	PEX12	17	33901814	33905882	ENSG00000022840	RNF10	12	120971283	121015397	0.028189	28.032	4.30E-28	1.21E-21	0.010127	1.8742	0.096301	1	Yes	No
ENSG00000114388	NPRL2	3	50384919	50387453	ENSG00000100228	RAB36	22	23487513	23506537	0.025655	63.653	5.17E-28	1.43E-21	0.00072042	0.22061	0.88212	1	No	NA
ENSG00000172660	TAF15	17	34136459	34174246	ENSG00000158856	DMTN	8	21906506	21940038	0.028606	23.711	8.80E-28	2.40E-21	NA	NA	NA	NA	Yes	No
ENSG00000187492	CDHR4	3	49828165	49837268	ENSG00000103023	PRSS54	16	58318309	58328951	0.025351	62.879	1.10E-27	2.96E-21	NA	NA	NA	NA	No	NA
ENSG00000141002	TCF25	16	89940014	89977792	ENSG00000175718	RBMXL3	X	114423963	114427431	0.027598	27.427	1.81E-27	4.82E-21	NA	NA	NA	NA	No	NA
ENSG00000172660	TAF15	17	34136459	34174246	ENSG00000070182	SPTB	14	65216372	65346601	0.028281	23.434	1.93E-27	5.07E-21	0.005744	0.88102	0.50813	1	No	NA
ENSG00000187492	CDHR4	3	49828165	49837268	ENSG00000134595	SOX3	X	139585152	139587225	0.024821	61.533	4.08E-27	1.06E-20	NA	NA	NA	NA	No	NA
ENSG00000182179	UBA7	3	49842640	49851379	ENSG00000100228	RAB36	22	23487513	23506537	0.026462	32.842	4.64E-27	1.19E-20	0.006112	1.4098	0.22871	1	No	NA
ENSG00000185909	KLHDC8B	3	49209044	49213917	ENSG00000134595	SOX3	X	139585152	139587225	0.025418	42.026	8.39E-27	2.12E-20	NA	NA	NA	NA	No	NA
ENSG00000164077	MON1A	3	49946302	49967606	ENSG00000100228	RAB36	22	23487513	23506537	0.02527	41.775	1.21E-26	3.02E-20	0.00046858	0.14345	0.93387	1	No	NA
ENSG00000003756	RBM5	3	50126341	50156454	ENSG00000103023	PRSS54	16	58318309	58328951	0.025083	41.457	1.92E-26	4.73E-20	NA	NA	NA	NA	No	NA
ENSG00000006125	AP2B1	17	33913918	34053436	ENSG00000022840	RNF10	12	120971283	121015397	0.027739	19.686	3.59E-26	8.76E-20	0.020938	3.2613	0.0035335	1	Yes	Yes
ENSG00000088543	C3orf18	3	50595462	50605182	ENSG00000100228	RAB36	22	23487513	23506537	0.026296	26.099	4.27E-26	1.03E-19	0.0048872	0.89973	0.48055	1	No	NA
ENSG00000141076	CIRH1A	16	69165194	69265033	ENSG00000255833	TIFAB	5	134787899	134788089	0.026103	25.902	6.82E-26	1.63E-19	0.0022297	0.4094	0.84246	1	Yes	No
ENSG00000101577	LPIN2	18	2916992	3013313	ENSG00000110172	CHORDC1	11	89934328	89956532	0.030825	11.802	1.18E-25	2.77E-19	0.0082657	0.63135	0.81658	1	No	NA
ENSG00000189091	SF3B3	16	70557691	70608820	ENSG00000255833	TIFAB	5	134787899	134788089	0.024303	40.135	1.30E-25	3.04E-19	0.0029904	0.6876	0.60062	1	No	NA
ENSG00000189180	ZNF33A	10	38299578	38354016	ENSG00000176200	OR4D11	11	59271049	59271984	0.026523	21.937	1.34E-25	3.08E-19	NA	NA	NA	NA	No	NA
ENSG00000154760	SLFN13	17	33762115	33775856	ENSG00000158856	DMTN	8	21906506	21940038	0.029526	13.348	1.74E-25	3.96E-19	NA	NA	NA	NA	Yes	No
ENSG00000126062	TMEM115	3	50392180	50392978	ENSG00000134595	SOX3	X	139585152	139587225	0.026978	19.131	2.22E-25	5.01E-19	NA	NA	NA	NA	No	NA
ENSG00000178057	NDUFAF3	3	49057892	49060928	ENSG00000103023	PRSS54	16	58318309	58328951	0.024822	30.755	2.55E-25	5.68E-19	NA	NA	NA	NA	No	NA
ENSG00000185869	ZNF829	19	37379026	37407193	ENSG00000111291	GPRC5D	12	13093709	13105081	0.026825	19.019	3.20E-25	7.06E-19	0.044878	7.1654	1.75E-07	0.0003227	No	NA
ENSG00000157349	DDX19B	16	70323566	70369186	ENSG00000255833	TIFAB	5	134787899	134788089	0.026131	21.604	3.44E-25	7.50E-19	0.0017396	0.31924	0.90156	1	No	NA
ENSG00000003756	RBM5	3	50126341	50156454	ENSG00000134595	SOX3	X	139585152	139587225	0.023784	39.258	4.66E-25	1.00E-18	NA	NA	NA	NA	No	NA
ENSG00000151948	GLT1D1	12	129337972	129469509	ENSG00000035720	STAP1	4	68424446	68473055	0.027823	15.353	5.89E-25	1.26E-18	0.0078315	0.79986	0.61652	1	No	NA
ENSG00000185614	FAM212A	3	49840687	49842463	ENSG00000100228	RAB36	22	23487513	23506537	0.023547	38.857	8.34E-25	1.76E-18	0.009571	2.957	0.031591	1	No	NA
ENSG00000173421	CCDC36	3	49235861	49295537	ENSG00000134595	SOX3	X	139585152	139587225	0.024261	30.042	1.00E-24	2.09E-18	NA	NA	NA	NA	No	NA
ENSG00000196782	MAML3	4	140637907	141075338	ENSG00000154473	BUB3	10	124913793	124924886	0.024145	29.895	1.33E-24	2.75E-18	0.0025761	0.59209	0.66844	1	No	NA
ENSG00000164068	RNF123	3	49728563	49753910	ENSG00000100228	RAB36	22	23487513	23506537	0.023166	38.213	2.13E-24	4.36E-18	0.0026132	0.80174	0.49301	1	No	NA
ENSG00000154760	SLFN13	17	33762115	33775856	ENSG00000070182	SPTB	14	65216372	65346601	0.0284	12.824	2.40E-24	4.87E-18	0.01803	1.3908	0.16408	1	Yes	No
ENSG00000178057	NDUFAF3	3	49057892	49060928	ENSG00000134595	SOX3	X	139585152	139587225	0.023782	29.435	3.21E-24	6.45E-18	NA	NA	NA	NA	No	NA
ENSG00000114738	MAPKAPK3	3	50649339	50686720	ENSG00000103023	PRSS54	16	58318309	58328951	0.025108	20.737	4.01E-24	7.99E-18	NA	NA	NA	NA	No	NA
ENSG00000100258	LMF2	22	50941376	50946135	ENSG00000258986	TMEM179	14	104941015	105071984	0.028158	12.711	4.22E-24	8.32E-18	NA	NA	NA	NA	No	NA
ENSG00000171804	WDR87	19	38375463	38397317	ENSG00000111291	GPRC5D	12	13093709	13105081	0.023341	28.876	9.38E-24	1.83E-17	0.035448	8.4251	1.12E-06	0.00205072	No	NA
ENSG00000126062	TMEM115	3	50392180	50392978	ENSG00000103023	PRSS54	16	58318309	58328951	0.025366	17.958	1.04E-23	2.01E-17	NA	NA	NA	NA	No	NA
ENSG00000141150	RASL10B	17	34058668	34070540	ENSG00000158856	DMTN	8	21906506	21940038	0.025836	16.009	1.51E-23	2.90E-17	NA	NA	NA	NA	Yes	No
ENSG00000176095	IP6K1	3	49761727	49823975	ENSG00000100228	RAB36	22	23487513	23506537	0.023108	28.581	1.65E-23	3.14E-17	0.0011185	0.34264	0.7945	1	No	NA
ENSG00000185909	KLHDC8B	3	49209044	49213917	ENSG00000100228	RAB36	22	23487513	23506537	0.02183	35.961	5.61E-23	1.05E-16	0.0072554	1.6755	0.15352	1	No	NA
ENSG00000015413	DPEP1	16	89679716	89704839	ENSG00000175718	RBMXL3	X	114423963	114427431	0.024528	17.35	7.63E-23	1.42E-16	NA	NA	NA	NA	No	NA
ENSG00000006125	AP2B1	17	33913918	34053436	ENSG00000113140	SPARC	5	151040657	151066726	0.024269	17.162	1.41E-22	2.61E-16	0.038528	6.111	2.72E-06	0.00495584	Yes	Yes
ENSG00000185909	KLHDC8B	3	49209044	49213917	ENSG00000103023	PRSS54	16	58318309	58328951	0.021242	34.97	2.37E-22	4.34E-16	NA	NA	NA	NA	No	NA
ENSG00000196381	ZNF781	19	38158652	38169674	ENSG00000111291	GPRC5D	12	13093709	13105081	0.021884	27.033	3.23E-22	5.86E-16	0.036129	8.5929	8.27E-07	0.00151755	No	NA
ENSG00000172046	USP19	3	49145479	49158371	ENSG00000134595	SOX3	X	139585152	139587225	0.021083	34.703	3.49E-22	6.28E-16	NA	NA	NA	NA	No	NA
ENSG00000204991	SPIRE2	16	89884587	89937727	ENSG00000175718	RBMXL3	X	114423963	114427431	0.022534	22.279	3.78E-22	6.74E-16	NA	NA	NA	NA	No	NA
ENSG00000172046	USP19	3	49145479	49158371	ENSG00000103023	PRSS54	16	58318309	58328951	0.02104	34.631	3.88E-22	6.85E-16	NA	NA	NA	NA	No	NA
ENSG00000225399	RP11-3B7.1	3	49297518	49298744	ENSG00000100228	RAB36	22	23487513	23506537	0.020853	34.317	6.13E-22	1.07E-15	0.0053267	1.6387	0.17884	1	No	NA
ENSG00000258839	MC1R	16	89978527	89981576	ENSG00000175718	RBMXL3	X	114423963	114427431	0.027477	9.7334	8.58E-22	1.49E-15	NA	NA	NA	NA	No	NA
ENSG00000114738	MAPKAPK3	3	50649339	50686720	ENSG00000134595	SOX3	X	139585152	139587225	0.022732	18.729	1.18E-21	2.03E-15	NA	NA	NA	NA	No	NA
ENSG00000105993	DNAJB6	7	157128075	157210133	ENSG00000128284	APOL3	22	36536372	36562225	0.022401	18.45	2.60E-21	4.44E-15	0.01209	1.8662	0.08374	1	No	NA
ENSG00000205045	SLFN12L	17	33800708	33864880	ENSG00000079308	TNS1	2	218664512	218867718	0.023034	16.268	2.63E-21	4.46E-15	0.0094923	1.2513	0.27177	1	No	NA
ENSG00000145020	AMT	3	49454211	49460186	ENSG00000103023	PRSS54	16	58318309	58328951	0.019291	47.554	3.53E-21	5.93E-15	NA	NA	NA	NA	No	NA
ENSG00000100239	PPP6R2	22	50781733	50883514	ENSG00000258986	TMEM179	14	104941015	105071984	0.024613	12.18	4.22E-21	7.02E-15	NA	NA	NA	NA	No	NA
ENSG00000006125	AP2B1	17	33913918	34053436	ENSG00000120885	CLU	8	27454434	27472548	0.022828	16.119	4.29E-21	7.03E-15	0.030828	4.8509	6.89E-05	0.1216085	Yes	Yes
ENSG00000007402	CACNA2D2	3	50400233	50541675	ENSG00000103023	PRSS54	16	58318309	58328951	0.020059	32.984	4.26E-21	7.03E-15	NA	NA	NA	NA	No	NA
ENSG00000145020	AMT	3	49454211	49460186	ENSG00000134595	SOX3	X	139585152	139587225	0.018881	46.524	9.70E-21	1.58E-14	NA	NA	NA	NA	No	NA
ENSG00000172123	SLFN12	17	33738079	33760302	ENSG00000158856	DMTN	8	21906506	21940038	0.026889	8.8828	1.03E-20	1.66E-14	NA	NA	NA	NA	Yes	No
ENSG00000068745	IP6K2	3	48725436	48777786	ENSG00000103023	PRSS54	16	58318309	58328951	0.02176	17.91	1.20E-20	1.92E-14	NA	NA	NA	NA	No	NA
ENSG00000175395	ZNF25	10	38238500	38265561	ENSG00000176200	OR4D11	11	59271049	59271984	0.021692	17.853	1.41E-20	2.23E-14	NA	NA	NA	NA	No	NA
ENSG00000172660	TAF15	17	34136459	34174246	ENSG00000022840	RNF10	12	120971283	121015397	0.021679	17.842	1.45E-20	2.29E-14	0.015663	2.4266	0.024741	1	Yes	No

ENSG00000163947	ARHGEF3	3	56761446	57113357	ENSG00000174175	SELP	1	169558087	169599431	0.022911	14.154	1.48E-20	2.31E-14	0.044743	5.3455	1.40E-06	0.0025592	No	NA
ENSG00000004534	RBM6	3	49977440	50114683	ENSG00000120937	NPPB	1	11917521	11918988	0.018708	46.088	1.49E-20	2.31E-14	NA	NA	NA	NA	No	NA
ENSG00000178057	NDUFAF3	3	49057892	49060928	ENSG00000100228	RAB36	22	23487513	23506537	0.020295	25.03	1.52E-20	2.34E-14	0.0094471	1.4544	0.19092	1	No	NA
ENSG00000163947	ARHGEF3	3	56761446	57113357	ENSG00000161911	TREML1	6	41117080	41122075	0.022889	14.14	1.56E-20	2.39E-14	0.041996	5.0029	4.33E-06	0.00785895	No	NA
ENSG00000108733	PEX12	17	33901814	33905882	ENSG00000100614	PPM1A	14	60712470	60765805	0.020937	20.667	1.75E-20	2.66E-14	0.0095301	1.7627	0.11792	1	Yes	No
ENSG00000205045	SLFN12L	17	33800708	33864880	ENSG00000022840	RNF10	12	120971283	121015397	0.022218	15.679	1.81E-20	2.73E-14	0.016902	2.2449	0.028792	1	Yes	Yes
ENSG00000172046	USP19	3	49145479	49158371	ENSG00000100228	RAB36	22	23487513	23506537	0.019447	31.957	1.90E-20	2.84E-14	0.0043261	1.9965	0.1364	1	No	NA
ENSG00000108733	PEX12	17	33901814	33905882	ENSG00000079308	TNS1	2	218664512	218867718	0.020828	20.556	2.28E-20	3.38E-14	0.0094253	1.7432	0.12214	1	No	NA
ENSG00000102908	NFAT5	16	69598997	69738569	ENSG00000255833	TIFAB	5	134787899	134788089	0.021347	17.563	3.19E-20	4.70E-14	0.0059486	0.91259	0.48495	1	Yes	No
ENSG00000006125	AP2B1	17	33913918	34053436	ENSG00000103148	NPRL3	16	138697	188859	0.02194	15.478	3.49E-20	5.11E-14	0.023502	3.6704	0.0013113	1	Yes	Yes
ENSG00000108733	PEX12	17	33901814	33905882	ENSG00000166947	EPB42	15	43398423	43513481	0.020501	20.227	4.98E-20	7.20E-14	0.0076745	1.4168	0.21559	1	Yes	No
ENSG00000217442	SYCE3	22	50989541	51001334	ENSG00000258986	TMEM179	14	104941015	105071984	0.025676	9.0783	4.99E-20	7.20E-14	NA	NA	NA	NA	No	NA
ENSG00000163947	ARHGEF3	3	56761446	57113357	ENSG00000137801	THBS1	15	39873280	39891667	0.02235	13.8	5.50E-20	7.89E-14	0.021884	2.5534	0.0093673	1	No	NA
ENSG00000073169	RP3-402G11.5	22	50639408	50656045	ENSG00000258986	TMEM179	14	104941015	105071984	0.019707	24.29	6.29E-20	8.95E-14	NA	NA	NA	NA	No	NA
ENSG00000007402	CACNA2D2	3	50400233	50541675	ENSG00000134595	SOX3	X	139585152	139587225	0.018937	31.102	6.59E-20	9.32E-14	NA	NA	NA	NA	No	NA
ENSG00000163947	ARHGEF3	3	56761446	57113357	ENSG00000005961	ITGA2B	17	42449548	42466873	0.022263	13.744	6.76E-20	9.48E-14	0.057653	6.9822	5.79E-09	1.08E-05	No	NA
ENSG00000198182	ZNF607	19	38197283	38210691	ENSG00000111291	GPRC5D	12	13093709	13105081	0.020983	17.257	7.58E-20	1.06E-13	0.034943	5.5217	1.24E-05	0.0223076	No	NA
ENSG00000163947	ARHGEF3	3	56761446	57113357	ENSG00000138722	MMRN1	4	90800683	90875780	0.02206	13.617	1.08E-19	1.50E-13	0.055338	6.6854	1.58E-08	2.94E-05	No	NA
ENSG00000187741	FANCA	16	89809208	89883065	ENSG00000175718	RBMXL3	X	114423963	114427431	0.020108	19.832	1.28E-19	1.75E-13	NA	NA	NA	NA	No	NA
ENSG00000108733	PEX12	17	33901814	33905882	ENSG00000029534	ANK1	8	41510739	41754280	0.020054	19.777	1.45E-19	1.98E-13	0.0039326	0.7233	0.60602	1	Yes	No
ENSG00000139370	SLC15A4	12	129277739	129308528	ENSG00000035720	STAP1	4	68424446	68473055	0.021921	13.529	1.50E-19	2.03E-13	0.016652	1.7159	0.081192	1	No	NA
ENSG00000006125	AP2B1	17	33913918	34053436	ENSG00000079308	TNS1	2	218664512	218867718	0.021254	14.984	1.75E-19	2.37E-13	0.017822	2.7672	0.011351	1	No	NA
ENSG00000158805	ZNF276	16	89786808	89800446	ENSG00000175718	RBMXL3	X	114423963	114427431	0.021838	13.476	1.82E-19	2.44E-13	NA	NA	NA	NA	No	NA
ENSG00000134982	APC	5	112043195	112181936	ENSG00000169249	ZRSR2	X	15808595	15841383	0.020564	16.905	2.05E-19	2.73E-13	0.0044462	0.68107	0.66501	1	No	NA
ENSG00000168374	ARF4	3	57557090	57583947	ENSG00000259207	ITGB3	17	45387505	45389182	0.019884	19.606	2.18E-19	2.88E-13	0.047046	9.0444	2.10E-08	3.90E-05	Yes	No
ENSG00000108733	PEX12	17	33901814	33905882	ENSG00000167671	UBXN6	19	4446046	4457819	0.019839	19.56	2.43E-19	3.19E-13	0.025976	4.8858	0.00020333	0.35521751	Yes	No
ENSG00000114383	TUSC2	3	50362338	50365682	ENSG00000103023	PRSS54	16	58318309	58328951	0.01832	30.07	2.96E-19	3.87E-13	NA	NA	NA	NA	No	NA
ENSG00000114738	MAPKAPK3	3	50649339	50686720	ENSG00000100228	RAB36	22	23487513	23506537	0.020379	16.75	3.18E-19	4.12E-13	0.0069121	0.9088	0.49875	1	No	NA
ENSG00000163947	ARHGEF3	3	56761446	57113357	ENSG00000124491	F13A1	6	6144318	6321246	0.021447	13.229	4.54E-19	5.85E-13	0.029867	3.5135	0.00051905	0.8875755	No	NA
ENSG00000188315	C3orf62	3	49306219	49311573	ENSG00000134595	SOX3	X	139585152	139587225	0.018863	23.229	4.82E-19	6.18E-13	NA	NA	NA	NA	No	NA
ENSG00000163946	FAM208A	3	56658507	56717265	ENSG00000259207	ITGB3	17	45387505	45389182	0.021364	13.178	5.49E-19	6.99E-13	0.04809	6.5964	1.27E-07	0.00023444	Yes	No
ENSG00000068028	RASSF1	3	50367219	50378343	ENSG00000103023	PRSS54	16	58318309	58328951	0.019459	19.178	6.03E-19	7.63E-13	NA	NA	NA	NA	No	NA
ENSG00000172716	SLFN11	17	33677324	33700720	ENSG00000158856	DMTN	8	21906506	21940038	0.023488	9.6711	6.70E-19	8.37E-13	NA	NA	NA	NA	Yes	No
ENSG00000164062	APEH	3	49711435	49720579	ENSG00000134595	SOX3	X	139585152	139587225	0.017986	29.512	6.68E-19	8.37E-13	NA	NA	NA	NA	No	NA
ENSG00000154760	SLFN13	17	33762115	33775856	ENSG00000022840	RNF10	12	120971283	121015397	0.022926	10.294	7.18E-19	8.92E-13	0.022092	1.7113	0.059583	1	Yes	No
ENSG00000163947	ARHGEF3	3	56761446	57113357	ENSG00000049323	LTBP1	2	33172039	33624576	0.021132	13.031	9.43E-19	1.16E-12	0.051741	6.2272	7.37E-08	0.00013627	No	NA
ENSG00000006125	AP2B1	17	33913918	34053436	ENSG00000167671	UBXN6	19	4446046	4457819	0.020372	14.349	1.39E-18	1.71E-12	0.028611	4.4916	0.0001707	0.2992371	Yes	Yes
ENSG00000154760	SLFN13	17	33762115	33775856	ENSG00000198574	SH2D1B	1	162365056	162381928	0.022443	10.073	2.15E-18	2.63E-12	0.035117	2.757	0.0010882	1	No	NA
ENSG00000228008	CTD-2330K9.3	3	49943495	49954370	ENSG00000120937	NPPB	1	11917521	11918988	0.017431	28.585	2.58E-18	3.13E-12	NA	NA	NA	NA	No	NA
ENSG00000197863	ZNF790	19	37308330	37341689	ENSG00000111291	GPRC5D	12	13093709	13105081	0.020037	14.108	3.05E-18	3.68E-12	0.035353	4.7853	2.66E-05	0.0475342	No	NA
ENSG00000172660	TAF15	17	34136459	34174246	ENSG00000079308	TNS1	2	218664512	218867718	0.019375	15.909	3.41E-18	4.09E-12	0.019255	2.994	0.0066752	1	No	NA
ENSG00000068745	IP6K2	3	48725436	48777786	ENSG00000134595	SOX3	X	139585152	139587225	0.01936	15.896	3.54E-18	4.22E-12	NA	NA	NA	NA	No	NA
ENSG00000108733	PEX12	17	33901814	33905882	ENSG00000167992	VWCE	11	61025762	61062896	0.018709	18.426	3.60E-18	4.27E-12	0.024282	4.5592	0.00041072	0.70725984	Yes	No
ENSG00000166199	ALKBH3	11	43902361	43941816	ENSG00000129657	SEC14L1	17	75082798	75213179	0.020484	12.624	4.25E-18	5.01E-12	0.011854	1.3691	0.20608	1	No	NA
ENSG00000158792	SPATA2L	16	89767338	89768113	ENSG00000175718	RBMXL3	X	114423963	114427431	0.01924	15.795	4.70E-18	5.51E-12	NA	NA	NA	NA	No	NA
ENSG00000141150	RASL10B	17	34058668	34070540	ENSG00000070182	SPTB	14	65216372	65346601	0.02043	12.589	4.82E-18	5.62E-12	0.011587	1.5307	0.15312	1	No	NA
ENSG00000180776	ZDHHC20	13	21950263	22033509	ENSG00000120942	UBIAD1	1	11333263	11356106	0.021492	10.602	5.51E-18	6.38E-12	0.0068206	0.56813	0.85555	1	No	NA
ENSG00000108733	PEX12	17	33901814	33905882	ENSG00000115649	CNPPD1	2	220036619	220042035	0.018494	18.21	6.02E-18	6.93E-12	NA	NA	NA	NA	Yes	No
ENSG00000006125	AP2B1	17	33913918	34053436	ENSG00000167992	VWCE	11	61025762	61062896	0.019692	13.861	6.84E-18	7.83E-12	0.036926	5.8471	5.38E-06	0.0097378	Yes	Yes
ENSG00000068028	RASSF1	3	50367219	50378343	ENSG00000100228	RAB36	22	23487513	23506537	0.018424	18.139	7.11E-18	8.10E-12	0.0023454	0.53895	0.70716	1	No	NA
ENSG00000178773	CPNE7	16	89642176	89663654	ENSG00000175718	RBMXL3	X	114423963	114427431	0.021342	10.526	7.77E-18	8.80E-12	NA	NA	NA	NA	No	NA
ENSG00000164062	APEH	3	49711435	49720579	ENSG00000103023	PRSS54	16	58318309	58328951	0.016956	27.794	8.17E-18	9.20E-12	NA	NA	NA	NA	No	NA
ENSG00000108733	PEX12	17	33901814	33905882	ENSG00000103148	NPRL3	16	138697	188859	0.018355	18.069	8.39E-18	9.40E-12	0.017675	3.2963	0.0058837	1	Yes	No
ENSG00000172053	QARS	3	49133365	49142553	ENSG00000134595	SOX3	X	139585152	139587225	0.015152	74.405	8.51E-18	9.48E-12	NA	NA	NA	NA	No	NA
ENSG00000172123	SLFN12	17	33738079	33760302	ENSG00000070182	SPTB	14	65216372	65346601	0.02382	7.8443	9.39E-18	1.04E-11	0.0073391	1.1275	0.34412	1	Yes	No
ENSG00000108733	PEX12	17	33901814	33905882	ENSG00000144677	CTDSPL	3	37903451	38025960	0.018274	17.989	1.02E-17	1.12E-11	0.042292	8.0901	1.76E-07	0.00032437	Yes	No
ENSG00000112701	SENP6	6	76311225	76427997	ENSG00000124102	PI3	20	43803517	43805185	0.018887	15.5	1.08E-17	1.19E-11	0.049454	7.9341	2.34E-08	4.35E-05	No	NA
ENSG00000205045	SLFN12L	17	33800708	33864880	ENSG00000115649	CNPPD1	2	220036619	220042035	0.019444	13.682	1.22E-17	1.33E-11	NA	NA	NA	NA	Yes	Yes
ENSG00000163947	ARHGEF3	3	56761446	57113357	ENSG00000185245	GP1BA	17	4835592	4838325	0.019996	12.316	1.32E-17	1.43E-11	0.041227	4.9074	5.92E-06	0.01070336	No	NA
ENSG00000198218	QRICH1	3	49067140	49131796	ENSG00000134595	SOX3	X	139585152	139587225	0.017431	21.434	1.51E-17	1.63E-11	NA	NA	NA	NA	No	NA
ENSG00000131873	CHSY1	15	101715928	101792137	ENSG00000110680	CALCA	11	14988214	14993900	0.020411	11.178	1.86E-17	2.00E-11	NA	NA	NA	NA	No	NA
ENSG00000205045	SLFN12L	17	33800708	33864880	ENSG00000029534	ANK1	8	41510739	41754280	0.019255	13.547	1.90E-17	2.03E-11	0.0036513	0.4785	0.85059	1	Yes	Yes
ENSG00000006125	AP2B1	17	33913918	34053436	ENSG00000095303	PTGS1	9	125132824	125157982	0.0192	13.507	2.16E-17	2.29E-11	0.067205	10.987	7.60E-12	1.44E-08	Yes	Yes
ENSG00000006125	AP2B1	17	33913918	34053436	ENSG00000166947	EPB42	15	43398423	43513481	0.019197	13.505	2.18E-17	2.30E-11	0.0096765	1.4901	0.17833	1	Yes	Yes
ENSG00000163946	FAM208A	3	56658507	56717265	ENSG00000161911	TREML1	6	41117080	41122075	0.019753	12.164	2.32E-17	2.43E-11	0.025546	3.4231	0.0012773	1	Yes	No
ENSG00000132139	GAS2L2	17	34071530	34079897	ENSG00000158856	DMTN	8	21906506	21940038	0.019701	12.131	2.62E-17	2.74E-11	NA	NA	NA	NA	Yes	No
ENSG00000172716	SLFN11	17	33677324	33700720	ENSG00000070182	SPTB	14	65216372	65346601	0.021827	8.9723	2.82E-17	2.93E-11	0.013654	1.0486	0.40139	1	Yes	Yes

ENSG00000006125	AP2B1	17	33913918	34053436	ENSG00000115649	CNPPD1	2	220036619	220042035	0.019048	13.398	3.08E-17	3.19E-11	NA	NA	NA	NA	Yes	Yes
ENSG00000163946	FAM208A	3	56658507	56717265	ENSG00000138722	MMRN1	4	90800683	90875780	0.01959	12.062	3.38E-17	3.48E-11	0.034768	4.7032	3.38E-05	0.0601302	Yes	No
ENSG00000006125	AP2B1	17	33913918	34053436	ENSG00000029534	ANK1	8	41510739	41754280	0.018973	13.344	3.67E-17	3.76E-11	0.02106	3.2807	0.003373	1	Yes	Yes
ENSG00000163947	ARHGEF3	3	56761446	57113357	ENSG00000120885	CLU	8	27454434	27472548	0.019514	12.013	4.03E-17	4.11E-11	0.042216	5.0302	3.95E-06	0.00717715	No	NA
ENSG00000108733	PEX12	17	33901814	33905882	ENSG00000158828	PINK1	1	20959948	20978004	0.017688	17.402	4.09E-17	4.15E-11	0.0036093	0.66362	0.65116	1	Yes	No
ENSG00000118495	PLAGL1	6	144261437	144385735	ENSG00000182749	PAQR7	1	26187701	26197744	0.017643	17.356	4.56E-17	4.61E-11	0.10172	20.746	1.13E-19	2.15E-16	No	NA
ENSG00000108733	PEX12	17	33901814	33905882	ENSG00000198876	DCAF12	9	34086385	34127397	0.017631	17.344	4.69E-17	4.71E-11	0.0056093	1.0334	0.39655	1	No	NA
ENSG00000141013	GAS8	16	90087238	90108943	ENSG00000175718	RBMXL3	X	114423963	114427431	0.0188	13.221	5.49E-17	5.49E-11	NA	NA	NA	NA	No	NA
ENSG00000198218	QRICH1	3	49067140	49131796	ENSG00000103023	PRSS54	16	58318309	58328951	0.016864	20.726	5.90E-17	5.87E-11	NA	NA	NA	NA	No	NA
ENSG00000108733	PEX12	17	33901814	33905882	ENSG00000113140	SPARC	5	151040657	151066726	0.017487	17.2	6.61E-17	6.54E-11	0.029101	5.4911	5.46E-05	0.096642	Yes	No
ENSG00000198105	ZNF248	10	38091751	38147034	ENSG00000176200	OR4D11	11	59271049	59271984	0.018091	14.835	7.04E-17	6.94E-11	NA	NA	NA	NA	No	NA
ENSG00000163947	ARHGEF3	3	56761446	57113357	ENSG00000085733	CTTN	11	70244510	70282690	0.019261	11.855	7.22E-17	7.08E-11	0.038043	4.5133	2.14E-05	0.0383488	No	NA
ENSG00000004534	RBM6	3	49977440	50114683	ENSG00000204290	BTNL2	6	32361740	32374905	0.01525	37.438	7.34E-17	7.16E-11	NA	NA	NA	NA	No	NA
ENSG00000006125	AP2B1	17	33913918	34053436	ENSG00000144677	CTDSPL	3	37903451	38025960	0.018652	13.114	7.76E-17	7.54E-11	0.053393	8.6016	4.06E-09	7.59E-06	Yes	Yes
ENSG00000108733	PEX12	17	33901814	33905882	ENSG00000238243	OR2W3	1	248058859	248060449	0.01736	17.073	8.93E-17	8.55E-11	0.011473	2.1262	0.060214	1	Yes	No
ENSG00000130489	SCO2	22	50961997	50964035	ENSG00000258986	TMEM179	14	104941015	105071984	0.020274	9.9888	8.84E-17	8.55E-11	NA	NA	NA	NA	No	NA
ENSG00000108733	PEX12	17	33901814	33905882	ENSG00000162722	TRIM58	1	248020501	248031169	0.01736	17.073	8.93E-17	8.55E-11	0.0019578	0.35938	0.87632	1	No	NA
ENSG00000205045	SLFN12L	17	33800708	33864880	ENSG00000167671	UBXN6	19	4446046	4457819	0.018549	13.041	9.85E-17	9.39E-11	0.023147	3.094	0.0031412	1	Yes	Yes
ENSG00000154760	SLFN13	17	33762115	33775856	ENSG00000079308	TNS1	2	218664512	218867718	0.020746	9.2945	1.00E-16	9.51E-11	0.016294	1.2547	0.24062	1	No	NA
ENSG00000188227	ZNF793	19	37997841	38039664	ENSG00000111291	GPRC5D	12	13093709	13105081	0.017914	14.687	1.07E-16	1.01E-10	0.038251	6.0653	3.06E-06	0.00556614	No	NA
ENSG00000132330	SCLY	2	238969530	238982488	ENSG00000088854	C20orf194	20	3229951	3388272	0.017906	14.68	1.09E-16	1.02E-10	0.007422	0.85337	0.55575	1	No	NA
ENSG00000007402	CACNA2D2	3	50400233	50541675	ENSG00000100228	RAB36	22	23487513	23506537	0.015887	26.012	1.10E-16	1.03E-10	0.0042237	0.97239	0.42172	1	No	NA
ENSG00000075399	VPS9D1	16	89773542	89785777	ENSG00000175718	RBMXL3	X	114423963	114427431	0.017266	16.979	1.12E-16	1.04E-10	NA	NA	NA	NA	No	NA
ENSG00000172053	QARS	3	49133365	49142553	ENSG00000103023	PRSS54	16	58318309	58328951	0.014109	69.208	1.14E-16	1.06E-10	NA	NA	NA	NA	No	NA
ENSG00000168374	ARF4	3	57557090	57583947	ENSG00000166086	JAM3	11	133938820	134018713	0.017199	16.912	1.31E-16	1.21E-10	0.021647	4.0534	0.0012078	1	Yes	No
ENSG00000188315	C3orf62	3	49306219	49311573	ENSG00000103023	PRSS54	16	58318309	58328951	0.016464	20.226	1.54E-16	1.42E-10	NA	NA	NA	NA	No	NA
ENSG00000172660	TAF15	17	34136459	34174246	ENSG00000103148	NPRL3	16	138697	188859	0.017748	14.548	1.58E-16	1.45E-10	0.013403	2.0718	0.054126	1	Yes	No
ENSG00000132437	DDC	7	50526134	50633154	ENSG00000017483	SLC38A5	X	48316920	48328644	0.017695	14.504	1.79E-16	1.63E-10	0.051834	8.3368	8.15E-09	1.52E-05	No	NA
ENSG00000205045	SLFN12L	17	33800708	33864880	ENSG00000146535	GNA12	7	2767746	2883958	0.018269	12.84	1.89E-16	1.71E-10	0.014015	1.8559	0.073703	1	Yes	Yes
ENSG00000179979	CRIPAK	4	1385340	1389780	ENSG00000132740	IGHMBP2	11	68671310	68708067	0.016375	20.114	1.91E-16	1.72E-10	0.0033891	0.7796	0.53853	1	No	NA
ENSG00000068028	RASSF1	3	50367219	50378343	ENSG00000134595	SOX3	X	139585152	139587225	0.017028	16.741	1.96E-16	1.76E-10	NA	NA	NA	NA	No	NA
ENSG00000132436	FIGNL1	7	50511831	50518088	ENSG00000017483	SLC38A5	X	48316920	48328644	0.013877	68.055	2.03E-16	1.82E-10	0.051201	49.647	3.61E-12	6.82E-09	Yes	Yes
ENSG00000006125	AP2B1	17	33913918	34053436	ENSG00000158828	PINK1	1	20959948	20978004	0.018207	12.796	2.18E-16	1.95E-10	0.0078507	1.2067	0.30032	1	Yes	Yes
ENSG00000205045	SLFN12L	17	33800708	33864880	ENSG00000113140	SPARC	5	151040657	151066726	0.018167	12.767	2.40E-16	2.13E-10	0.016255	2.1575	0.035743	1	Yes	Yes
ENSG00000181666	HKR1	19	37803739	37860267	ENSG00000111291	GPRC5D	12	13093709	13105081	0.018699	11.502	2.64E-16	2.34E-10	0.03306	3.902	0.00015284	0.26808136	No	NA
ENSG00000178149	DALRD3	3	49053591	49056010	ENSG00000134595	SOX3	X	139585152	139587225	0.016158	19.843	3.21E-16	2.83E-10	NA	NA	NA	NA	No	NA
ENSG00000171817	ZNF540	19	38042308	38092058	ENSG00000111291	GPRC5D	12	13093709	13105081	0.016137	19.817	3.38E-16	2.96E-10	0.038355	9.1436	3.03E-07	0.00055752	No	NA
ENSG00000188511	C22orf34	22	49808176	50051190	ENSG00000179921	GPBAR1	2	219124219	219128582	0.016794	16.506	3.42E-16	2.97E-10	0.0063791	0.97906	0.4381	1	No	NA
ENSG00000164078	MST1R	3	49924435	49936696	ENSG00000120937	NPPB	1	11917521	11918988	0.016133	19.812	3.41E-16	2.97E-10	NA	NA	NA	NA	No	NA
ENSG00000164062	APEH	3	49711435	49720579	ENSG00000100228	RAB36	22	23487513	23506537	0.015397	25.197	3.60E-16	3.11E-10	0.0042251	0.77732	0.56613	1	No	NA
ENSG00000006125	AP2B1	17	33913918	34053436	ENSG00000238243	OR2W3	1	248058859	248060449	0.017974	12.629	3.75E-16	3.22E-10	0.012355	1.9078	0.076762	1	No	NA
ENSG00000006125	AP2B1	17	33913918	34053436	ENSG00000162722	TRIM58	1	248020501	248031169	0.017974	12.629	3.75E-16	3.22E-10	0.006253	0.95958	0.45154	1	No	NA
ENSG00000167136	ENDOG	9	131580753	131581464	ENSG00000164821	DEFA4	8	6793344	6795860	0.016071	19.735	3.96E-16	3.38E-10	0.019786	2.6356	0.010626	1	Yes	Yes
ENSG00000008300	CELSR3	3	48673902	48700348	ENSG00000134595	SOX3	X	139585152	139587225	0.017305	14.179	4.46E-16	3.80E-10	NA	NA	NA	NA	No	NA
ENSG00000222012	AC005481.5	7	157406715	157411457	ENSG00000128284	APOL3	22	36536372	36562225	0.016006	19.654	4.62E-16	3.90E-10	0.0031435	0.72292	0.57635	1	No	NA
ENSG00000163947	ARHGEF3	3	56761446	57113357	ENSG00000172572	PDE3A	12	20522179	20837315	0.018456	11.35	4.63E-16	3.90E-10	NA	NA	NA	NA	No	NA
ENSG00000168872	DDX19A	16	70380732	70407286	ENSG00000255833	TIFAB	5	134787899	134788089	0.017275	14.154	4.79E-16	4.02E-10	0.004347	0.66582	0.67736	1	No	NA
ENSG00000205045	SLFN12L	17	33800708	33864880	ENSG00000167992	VWCE	11	61025762	61062896	0.017858	12.546	4.92E-16	4.12E-10	0.026359	3.5349	0.00093718	1	Yes	Yes
ENSG00000228008	CTD-2330K9.3	3	49943495	49954370	ENSG00000204290	BTNL2	6	32361740	32374905	0.015175	24.83	6.15E-16	5.12E-10	NA	NA	NA	NA	No	NA
ENSG00000172660	TAF15	17	34136459	34174246	ENSG00000029534	ANK1	8	41510739	41754280	0.017143	14.044	6.53E-16	5.42E-10	0.012248	1.8909	0.079527	1	Yes	No
ENSG00000205045	SLFN12L	17	33800708	33864880	ENSG00000120885	CLU	8	27454434	27472548	0.017708	12.439	6.97E-16	5.76E-10	0.018045	2.3995	0.019513	1	Yes	Yes
ENSG00000092871	RFFL	17	33341759	33416338	ENSG00000158856	DMTN	8	21906506	21940038	0.017091	14	7.37E-16	6.07E-10	NA	NA	NA	NA	Yes	No
ENSG00000132139	GAS2L2	17	34071530	34079897	ENSG00000070182	SPTB	14	65216372	65346601	0.018238	11.213	7.63E-16	6.26E-10	0.010397	1.199	0.29627	1	No	NA
ENSG00000205045	SLFN12L	17	33800708	33864880	ENSG00000144677	CTDSPL	3	37903451	38025960	0.017633	12.385	8.30E-16	6.78E-10	0.029205	3.9281	0.00031123	0.5384279	Yes	Yes
ENSG00000163947	ARHGEF3	3	56761446	57113357	ENSG00000166086	JAM3	11	133938820	134018713	0.018105	11.13	1.04E-15	8.42E-10	0.018748	2.1805	0.02679	1	No	NA
ENSG00000068745	IP6K2	3	48725436	48777786	ENSG00000100228	RAB36	22	23487513	23506537	0.016898	13.84	1.16E-15	9.39E-10	0.011099	2.0562	0.068698	1	No	NA
ENSG00000172660	TAF15	17	34136459	34174246	ENSG00000238243	OR2W3	1	248058859	248060449	0.016879	13.824	1.21E-15	9.73E-10	0.005008	0.76756	0.59553	1	Yes	No
ENSG00000172660	TAF15	17	34136459	34174246	ENSG00000162722	TRIM58	1	248020501	248031169	0.016879	13.824	1.21E-15	9.73E-10	0.0027661	0.423	0.86396	1	No	NA
ENSG00000198917	C9orf114	9	131586026	131592100	ENSG00000164821	DEFA4	8	6793344	6795860	0.015581	19.124	1.28E-15	1.02E-09	0.015112	4.6953	0.0029236	1	Yes	No
ENSG00000164078	MST1R	3	49924435	49936696	ENSG00000204290	BTNL2	6	32361740	32374905	0.015577	19.119	1.29E-15	1.03E-09	NA	NA	NA	NA	No	NA
ENSG00000006125	AP2B1	17	33913918	34053436	ENSG00000184481	FOXO4	X	70316047	70323385	0.017387	12.209	1.47E-15	1.17E-09	0.017359	2.6939	0.013449	1	No	NA
ENSG00000107742	SPOCK2	10	73818793	73848790	ENSG00000162931	TRIM17	1	228595641	228604562	0.017357	12.188	1.57E-15	1.24E-09	0.0012389	0.16196	0.99232	1	No	NA
ENSG00000205045	SLFN12L	17	33800708	33864880	ENSG00000138867	GUCD1	22	24936406	24951284	0.017279	12.132	1.88E-15	1.49E-09	NA	NA	NA	NA	Yes	Yes
ENSG00000066422	ZBTB11	3	101367733	101391057	ENSG00000196724	ZNF418	19	58433252	58446761	0.016053	15.767	1.97E-15	1.55E-09	0.058131	11.307	1.34E-10	2.52E-07	No	NA
ENSG00000108733	PEX12	17	33901814	33905882	ENSG00000183597	TANGO2	22	20004537	20053449	0.016024	15.737	2.12E-15	1.66E-09	NA	NA	NA	NA	No	NA
ENSG00000108733	PEX12	17	33901814	33905882	ENSG00000120885	CLU	8	27454434	27472548	0.016017	15.731	2.15E-15	1.68E-09	0.02249	4.2149	0.00085725	1	Yes	No
ENSG00000163947	ARHGEF3	3	56761446	57113357	ENSG00000188677	PARVB	22	44395091	44565106	0.017759	10.913	2.29E-15	1.78E-09	0.032718	3.8602	0.00017449	0.30553199	No	NA

ENSG00000196586	MYO6	6	76458909	76629254	ENSG00000124102	PI3	20	43803517	43805185	0.017192	12.07	2.31E-15	1.78E-09	0.051321	7.0636	3.13E-08	5.80E-05	No	NA
ENSG00000006125	AP2B1	17	33913918	34053436	ENSG00000128266	GNAZ	22	23412540	23464889	0.017097	12.002	2.87E-15	2.21E-09	0.014381	2.225	0.03876	1	Yes	Yes
ENSG00000067560	RHOA	3	49396578	49449635	ENSG00000134595	SOX3	X	139585152	139587225	0.01521	18.662	3.10E-15	2.38E-09	NA	NA	NA	NA	No	NA
ENSG00000114302	PRKAR2A	3	48782030	48885279	ENSG00000103023	PRSS54	16	58318309	58328951	0.014439	23.607	3.66E-15	2.80E-09	NA	NA	NA	NA	No	NA
ENSG00000205045	SLFN12L	17	33800708	33864880	ENSG00000238243	OR2W3	1	248058859	248060449	0.016989	11.925	3.68E-15	2.81E-09	0.005623	0.73835	0.63947	1	Yes	Yes
ENSG00000006125	AP2B1	17	33913918	34053436	ENSG00000100614	PPM1A	14	60712470	60765805	0.016916	11.873	4.36E-15	3.31E-09	0.014985	2.32	0.031413	1	Yes	Yes
ENSG00000103043	VAC14	16	70721342	70835064	ENSG00000255833	TIFAB	5	134787899	134788089	0.015682	15.396	4.75E-15	3.59E-09	0.0016633	0.30522	0.90989	1	No	NA
ENSG00000142082	SIRT3	11	215458	236431	ENSG00000259207	ITGB3	17	45387505	45389182	0.01431	23.393	5.00E-15	3.76E-09	0.009518	2.203	0.066856	1	Yes	No
ENSG00000164076	CAMKV	3	49895422	49907655	ENSG00000120937	NPPB	1	11917521	11918988	0.01429	23.36	5.24E-15	3.93E-09	NA	NA	NA	NA	No	NA
ENSG00000105738	SIPA1L3	19	38397868	38699012	ENSG00000111291	GPRC5D	12	13093709	13105081	0.017383	10.679	5.42E-15	4.06E-09	0.037358	3.9325	6.32E-05	0.1116112	No	NA
ENSG00000106004	HOXA5	7	27180671	27183287	ENSG00000176358	TAC4	17	47915671	47925379	0.016785	11.78	5.90E-15	4.40E-09	0.0069504	0.91387	0.49477	1	Yes	Yes
ENSG00000205045	SLFN12L	17	33800708	33864880	ENSG00000164068	RNF123	3	49728563	49753910	0.016781	11.776	5.97E-15	4.43E-09	0.019392	2.5821	0.012211	1	Yes	Yes
ENSG00000141150	RASL10B	17	34058668	34070540	ENSG00000022840	RNF10	12	120971283	121015397	0.017298	10.625	6.59E-15	4.88E-09	0.018721	2.4911	0.015443	1	Yes	No
ENSG00000172660	TAF15	17	34136459	34174246	ENSG00000115649	CNPPD1	2	220036619	220042035	0.016152	13.219	6.62E-15	4.88E-09	NA	NA	NA	NA	Yes	No
ENSG00000157350	ST3GAL2	16	70413338	70473140	ENSG00000255833	TIFAB	5	134787899	134788089	0.014174	23.168	6.93E-15	5.09E-09	0.0029271	0.89834	0.4415	1	No	NA
ENSG00000025770	NCAPH2	22	50946645	50961901	ENSG00000258986	TMEM179	14	104941015	105071984	0.01552	15.235	6.95E-15	5.09E-09	NA	NA	NA	NA	No	NA
ENSG00000154760	SLFN13	17	33762115	33775856	ENSG00000103148	NPRL3	16	138697	188859	0.018845	8.4266	7.07E-15	5.16E-09	0.030891	2.4146	0.0043955	1	Yes	No
ENSG00000205045	SLFN12L	17	33800708	33864880	ENSG00000095303	PTGS1	9	125132824	125157982	0.016692	11.713	7.33E-15	5.33E-09	0.034687	4.6918	3.49E-05	0.0620522	Yes	Yes
ENSG00000163947	ARHGEF3	3	56761446	57113357	ENSG00000095303	PTGS1	9	125132824	125157982	0.017231	10.584	7.68E-15	5.56E-09	0.042974	5.1247	2.90E-06	0.0052809	No	NA
ENSG00000172660	TAF15	17	34136459	34174246	ENSG00000166947	EPB42	15	43398423	43513481	0.016065	13.146	8.13E-15	5.87E-09	0.010195	1.5708	0.15244	1	Yes	No
ENSG00000172660	TAF15	17	34136459	34174246	ENSG00000158828	PINK1	1	20959948	20978004	0.01604	13.125	8.61E-15	6.19E-09	0.012027	1.8564	0.085476	1	Yes	No
ENSG00000001617	SEMA3F	3	50192478	50226508	ENSG00000120937	NPPB	1	11917521	11918988	0.015418	15.133	8.85E-15	6.34E-09	NA	NA	NA	NA	No	NA
ENSG00000196967	ZNF585A	19	37597636	37663643	ENSG00000111291	GPRC5D	12	13093709	13105081	0.015997	13.09	9.51E-15	6.79E-09	0.023408	3.6553	0.0013606	1	No	NA
ENSG00000114383	TUSC2	3	50362338	50365682	ENSG00000134595	SOX3	X	139585152	139587225	0.013979	22.844	1.11E-14	7.91E-09	NA	NA	NA	NA	No	NA
ENSG00000163947	ARHGEF3	3	56761446	57113357	ENSG00000140479	PCSK6	15	101840818	102065405	0.017064	10.479	1.12E-14	7.98E-09	0.083458	10.392	5.30E-14	1.01E-10	No	NA
ENSG00000092871	RFFL	17	33341759	33416338	ENSG00000070182	SPTB	14	65216372	65346601	0.015913	13.02	1.16E-14	8.18E-09	0.011585	1.3376	0.22088	1	No	NA
ENSG00000154760	SLFN13	17	33762115	33775856	ENSG00000173068	BNC2	9	16409501	16870841	0.018571	8.3019	1.30E-14	9.15E-09	0.045493	3.6103	2.69E-05	0.0480434	Yes	Yes
ENSG00000163947	ARHGEF3	3	56761446	57113357	ENSG00000198478	SH3BGRL2	6	80341000	80413372	0.01699	10.433	1.33E-14	9.36E-09	0.057135	6.9157	7.25E-09	1.35E-05	No	NA
ENSG00000177963	RIC8A	11	207511	215113	ENSG00000138722	MMRN1	4	90800683	90875780	0.015237	14.953	1.36E-14	9.46E-09	0.0074385	0.85528	0.55409	1	Yes	Yes
ENSG00000006125	AP2B1	17	33913918	34053436	ENSG00000154146	NRGN	11	124609829	124615878	0.016425	11.522	1.36E-14	9.46E-09	0.022245	3.4696	0.0021385	1	Yes	Yes
ENSG00000154760	SLFN13	17	33762115	33775856	ENSG00000115649	CNPPD1	2	220036619	220042035	0.018548	8.2914	1.37E-14	9.50E-09	NA	NA	NA	NA	Yes	No
ENSG00000154760	SLFN13	17	33762115	33775856	ENSG00000238243	OR2W3	1	248058859	248060449	0.018544	8.2896	1.38E-14	9.55E-09	0.012282	0.9419	0.50384	1	Yes	No
ENSG00000004534	RBM6	3	49977440	50114683	ENSG00000182156	ENPP7	17	77704681	77716021	0.013104	32.1	1.42E-14	9.76E-09	NA	NA	NA	NA	No	NA
ENSG00000172660	TAF15	17	34136459	34174246	ENSG00000167671	UBXN6	19	4446046	4457819	0.015752	12.886	1.69E-14	1.16E-08	0.026814	4.2019	0.00035265	0.60902655	Yes	No
ENSG00000006125	AP2B1	17	33913918	34053436	ENSG00000138867	GUCD1	22	24936406	24951284	0.016305	11.437	1.79E-14	1.23E-08	NA	NA	NA	NA	Yes	Yes
ENSG00000205045	SLFN12L	17	33800708	33864880	ENSG00000204463	BAG6	6	31606805	31620170	0.016302	11.435	1.80E-14	1.23E-08	0.031185	4.2029	0.00014253	0.25014015	Yes	Yes
ENSG00000011295	TTC19	17	15905229	15948329	ENSG00000169429	IL8	4	74606223	74609433	0.014446	17.71	1.92E-14	1.31E-08	0.02295	3.5821	0.0016267	1	Yes	Yes
ENSG00000205045	SLFN12L	17	33800708	33864880	ENSG00000118046	STK11	19	1189406	1226662	0.016275	11.415	1.92E-14	1.31E-08	0.0052143	0.68441	0.6854	1	Yes	Yes
ENSG00000136819	C9orf78	9	132589569	132598142	ENSG00000128294	TPST2	22	26921458	26992681	0.012932	31.673	2.16E-14	1.46E-08	0.010912	2.5292	0.039229	1	No	NA
ENSG00000164077	MON1A	3	49946302	49967606	ENSG00000120937	NPPB	1	11917521	11918988	0.013686	22.359	2.25E-14	1.52E-08	NA	NA	NA	NA	No	NA
ENSG00000139531	SUOX	12	56390964	56400425	ENSG00000105085	MED26	19	16698215	16739873	0.013676	22.342	2.31E-14	1.55E-08	0.0019339	0.59293	0.61973	1	Yes	Yes
ENSG00000174885	NLRP6	11	278365	285359	ENSG00000138722	MMRN1	4	90800683	90875780	0.01619	11.355	2.33E-14	1.56E-08	0.019026	1.9653	0.040297	1	Yes	Yes
ENSG00000108733	PEX12	17	33901814	33905882	ENSG00000166169	POLL	10	103338639	103346957	0.014987	14.704	2.45E-14	1.63E-08	0.018163	3.389	0.004859	1	Yes	No
ENSG00000205045	SLFN12L	17	33800708	33864880	ENSG00000140564	FURIN	15	91411822	91426688	0.01616	11.334	2.50E-14	1.66E-08	0.013682	1.8112	0.081766	1	Yes	Yes
ENSG00000181007	ZFP82	19	36874022	36909558	ENSG00000116962	NID1	1	236139130	236228462	0.015575	12.739	2.54E-14	1.69E-08	0.060348	9.7942	1.76E-10	3.31E-07	No	NA
ENSG00000108733	PEX12	17	33901814	33905882	ENSG00000095303	PTGS1	9	125132824	125157982	0.014951	14.668	2.66E-14	1.76E-08	0.053532	10.362	1.11E-09	2.08E-06	Yes	No
ENSG00000168374	ARF4	3	57557090	57583947	ENSG00000120885	CLU	8	27454434	27472548	0.01495	14.667	2.67E-14	1.76E-08	0.019569	3.6567	0.0027844	1	Yes	No
ENSG00000006125	AP2B1	17	33913918	34053436	ENSG00000149260	CAPN5	11	76777979	76837201	0.016113	11.3	2.78E-14	1.83E-08	0.0087916	1.3526	0.23104	1	Yes	Yes
ENSG00000154760	SLFN13	17	33762115	33775856	ENSG00000029534	ANK1	8	41510739	41754280	0.018206	8.1356	2.92E-14	1.91E-08	0.010725	0.82126	0.62861	1	Yes	No
ENSG00000006125	AP2B1	17	33913918	34053436	ENSG00000198876	DCAF12	9	34086385	34127397	0.016091	11.285	2.93E-14	1.91E-08	0.0058053	0.89048	0.50113	1	No	NA
ENSG00000108733	PEX12	17	33901814	33905882	ENSG00000165406	8-Mar	10	45950035	46090354	0.014889	14.606	3.08E-14	2.00E-08	0.0015396	0.2825	0.92281	1	No	NA
ENSG00000172123	SLFN12	17	33738079	33760302	ENSG00000115649	CNPPD1	2	220036619	220042035	0.019974	6.552	4.01E-14	2.60E-08	NA	NA	NA	NA	Yes	No
ENSG00000174885	NLRP6	11	278365	285359	ENSG00000259207	ITGB3	17	45387505	45389182	0.015949	11.183	4.07E-14	2.63E-08	0.025639	2.6665	0.004665	1	Yes	Yes
ENSG00000108474	PIGL	17	16120523	16250988	ENSG00000169429	IL8	4	74606223	74609433	0.01476	14.478	4.17E-14	2.69E-08	0.02372	5.5699	0.00019702	0.34458798	Yes	Yes
ENSG00000108733	PEX12	17	33901814	33905882	ENSG00000196914	ARHGEF12	11	120207787	120360645	0.014756	14.474	4.21E-14	2.71E-08	0.015257	2.8384	0.014947	1	No	NA
ENSG00000126062	TMEM115	3	50392180	50392978	ENSG00000100228	RAB36	22	23487513	23506537	0.015918	11.161	4.37E-14	2.80E-08	0.0075547	1.1609	0.32513	1	No	NA
ENSG00000205045	SLFN12L	17	33800708	33864880	ENSG00000166169	POLL	10	103338639	103346957	0.01591	11.155	4.44E-14	2.84E-08	0.029136	3.9185	0.00031981	0.55295149	Yes	Yes
ENSG00000114302	PRKAR2A	3	48782030	48885279	ENSG00000134595	SOX3	X	139585152	139587225	0.013396	21.879	4.54E-14	2.89E-08	NA	NA	NA	NA	No	NA
ENSG00000136827	TOR1A	9	132575223	132586413	ENSG00000128294	TPST2	22	26921458	26992681	0.014081	17.256	4.59E-14	2.92E-08	0.0081715	1.0758	0.37682	1	No	NA
ENSG00000140995	DEF8	16	90014333	90034468	ENSG00000175718	RBMXL3	X	114423963	114427431	0.015873	11.129	4.84E-14	3.06E-08	NA	NA	NA	NA	No	NA
ENSG00000172123	SLFN12	17	33738079	33760302	ENSG00000022840	RNF10	12	120971283	121015397	0.019841	6.5075	5.33E-14	3.36E-08	0.026038	4.0769	0.00048132	0.82498248	Yes	No
ENSG00000174885	NLRP6	11	278365	285359	ENSG00000205038	PKHD1L1	8	110374706	110542559	0.015814	11.087	5.54E-14	3.49E-08	0.011579	1.187	0.29959	1	Yes	Yes
ENSG00000177963	RIC8A	11	207511	215113	ENSG00000259207	ITGB3	17	45387505	45389182	0.014629	14.348	5.68E-14	3.55E-08	0.019068	2.2184	0.024137	1	Yes	Yes
ENSG00000006125	AP2B1	17	33913918	34053436	ENSG00000101335	MYL9	20	35169887	35178228	0.015805	11.08	5.66E-14	3.55E-08	0.047868	7.6669	4.72E-08	8.74E-05	Yes	Yes
ENSG00000196437	ZNF569	19	37902057	37958363	ENSG00000111291	GPRC5D	12	13093709	13105081	0.013274	21.677	6.09E-14	3.80E-08	0.019362	6.0416	0.00044872	0.77045224	No	NA
ENSG00000177963	RIC8A	11	207511	215113	ENSG00000205038	PKHD1L1	8	110374706	110542559	0.014564	14.282	6.62E-14	4.11E-08	0.0082959	0.95469	0.47035	1	Yes	Yes
ENSG00000169020	ATP5I	4	666225	667190	ENSG00000154263	ABCA10	17	67143355	67225001	0.013911	17.045	6.88E-14	4.26E-08	0.0071703	1.6557	0.15823	1	No	NA

ENSG00000251247	ZNF345	19	37341263	37403931	ENSG00000116962	NID1	1	236139130	236228462	0.014537	14.256	7.04E-14	4.35E-08	0.048037	9.2445	1.34E-08	2.49E-05	No	NA
ENSG00000108733	PEX12	17	33901814	33905882	ENSG00000184481	FOXO4	X	70316047	70323385	0.014535	14.253	7.09E-14	4.36E-08	0.015248	2.8366	0.015002	1	No	NA
ENSG00000003249	DBNDD1	16	90071273	90085881	ENSG00000175718	RBMXL3	X	114423963	114427431	0.016772	9.1506	7.35E-14	4.51E-08	NA	NA	NA	NA	No	NA
ENSG00000168374	ARF4	3	57557090	57583947	ENSG00000137801	THBS1	15	39873280	39891667	0.014485	14.204	7.96E-14	4.87E-08	0.023273	4.3653	0.00062212	1	Yes	No
ENSG00000163946	FAM208A	3	56658507	56717265	ENSG00000174175	SELP	1	169558087	169599431	0.0162	9.94	8.06E-14	4.92E-08	0.035909	4.8634	2.12E-05	0.0380116	Yes	No
ENSG00000108733	PEX12	17	33901814	33905882	ENSG00000138867	GUCD1	22	24936406	24951284	0.014459	14.178	8.47E-14	5.15E-08	NA	NA	NA	NA	Yes	No
ENSG00000025708	TYMP	22	50964181	50968485	ENSG00000258986	TMEM179	14	104941015	105071984	0.017206	8.4508	8.81E-14	5.34E-08	NA	NA	NA	NA	No	NA
ENSG00000228008	CTD-2330K9.3	3	49943495	49954370	ENSG00000182156	ENPP7	17	77704681	77716021	0.013119	21.42	8.87E-14	5.36E-08	NA	NA	NA	NA	No	NA
ENSG00000168374	ARF4	3	57557090	57583947	ENSG00000005961	ITGA2B	17	42449548	42466873	0.014433	14.152	9.01E-14	5.43E-08	0.030245	5.7138	3.36E-05	0.059808	Yes	No
ENSG00000006125	AP2B1	17	33913918	34053436	ENSG00000130300	PLVAP	19	17462257	17488159	0.015591	10.928	9.25E-14	5.56E-08	0.017937	2.7853	0.010882	1	Yes	Yes
ENSG00000168374	ARF4	3	57557090	57583947	ENSG00000161911	TREML1	6	41117080	41122075	0.014402	14.121	9.69E-14	5.79E-08	0.020584	3.8502	0.0018553	1	Yes	No
ENSG00000163946	FAM208A	3	56658507	56717265	ENSG00000049323	LTBP1	2	33172039	33624576	0.016121	9.8903	9.66E-14	5.79E-08	0.027958	3.7556	0.00050601	0.86628912	Yes	No
ENSG00000168374	ARF4	3	57557090	57583947	ENSG00000085733	CTTN	11	70244510	70282690	0.014373	14.093	1.04E-13	6.16E-08	0.019969	3.7328	0.0023743	1	Yes	No
ENSG00000141150	RASL10B	17	34058668	34070540	ENSG00000079308	TNS1	2	218664512	218867718	0.016091	9.8716	1.03E-13	6.16E-08	0.01288	1.7037	0.10453	1	No	NA
ENSG00000168374	ARF4	3	57557090	57583947	ENSG00000188677	PARVB	22	44395091	44565106	0.014356	14.076	1.08E-13	6.37E-08	0.034938	6.6324	4.46E-06	0.00808152	Yes	No
ENSG00000185379	RAD51D	17	33426811	33447063	ENSG00000070182	SPTB	14	65216372	65346601	0.013038	21.285	1.08E-13	6.37E-08	0.0072558	2.2365	0.082509	1	No	NA
ENSG00000177951	BET1L	11	167784	207428	ENSG00000049323	LTBP1	2	33172039	33624576	0.016058	9.8513	1.11E-13	6.54E-08	0.021628	1.8288	0.045403	1	Yes	Yes
ENSG00000178149	DALRD3	3	49053591	49056010	ENSG00000100228	RAB36	22	23487513	23506537	0.013708	16.793	1.11E-13	6.54E-08	0.0061148	1.4104	0.22849	1	No	NA
ENSG00000185379	RAD51D	17	33426811	33447063	ENSG00000158856	DMTN	8	21906506	21940038	0.013021	21.259	1.12E-13	6.57E-08	NA	NA	NA	NA	No	NA
ENSG00000205045	SLFN12L	17	33800708	33864880	ENSG00000101335	MYL9	20	35169887	35178228	0.015465	10.838	1.24E-13	7.22E-08	0.029251	3.9344	0.00030573	0.52952436	Yes	Yes
ENSG00000006125	AP2B1	17	33913918	34053436	ENSG00000166169	POLL	10	103338639	103346957	0.015434	10.816	1.33E-13	7.73E-08	0.031626	4.9805	4.96E-05	0.0879408	Yes	Yes
ENSG00000163946	FAM208A	3	56658507	56717265	ENSG00000122786	CALD1	7	134429003	134655479	0.015968	9.795	1.37E-13	7.93E-08	0.027496	3.6916	0.00060533	1	Yes	No
ENSG00000145022	TCTA	3	49452253	49453908	ENSG00000120937	NPPB	1	11917521	11918988	0.013552	16.599	1.62E-13	9.36E-08	NA	NA	NA	NA	No	NA
ENSG00000172660	TAF15	17	34136459	34174246	ENSG00000100614	PPM1A	14	60712470	60765805	0.014771	12.071	1.65E-13	9.53E-08	0.015191	2.3524	0.029223	1	Yes	No
ENSG00000142082	SIRT3	11	215458	236431	ENSG00000205038	PKHD1L1	8	110374706	110542559	0.012853	20.981	1.68E-13	9.68E-08	0.0021415	0.49199	0.74165	1	Yes	Yes
ENSG00000008300	CELSR3	3	48673902	48700348	ENSG00000103023	PRSS54	16	58318309	58328951	0.014739	12.045	1.78E-13	1.02E-07	NA	NA	NA	NA	No	NA
ENSG00000163946	FAM208A	3	56658507	56717265	ENSG00000198478	SH3BGRL2	6	80341000	80413372	0.015843	9.7169	1.82E-13	1.04E-07	0.037645	5.1077	1.04E-05	0.0187304	Yes	No
ENSG00000177951	BET1L	11	167784	207428	ENSG00000122786	CALD1	7	134429003	134655479	0.015829	9.7082	1.87E-13	1.07E-07	0.022356	1.8918	0.036883	1	Yes	Yes
ENSG00000141150	RASL10B	17	34058668	34070540	ENSG00000198574	SH2D1B	1	162365056	162381928	0.015818	9.7018	1.92E-13	1.09E-07	0.021882	2.9211	0.0049999	1	No	NA
ENSG00000205045	SLFN12L	17	33800708	33864880	ENSG00000158828	PINK1	1	20959948	20978004	0.015267	10.697	1.95E-13	1.11E-07	0.0061024	0.8017	0.58595	1	Yes	Yes
ENSG00000136816	TOR1B	9	132565432	132573560	ENSG00000128294	TPST2	22	26921458	26992681	0.01343	16.448	2.16E-13	1.22E-07	0.0079201	1.2175	0.29472	1	No	NA
ENSG00000149308	NPAT	11	108027942	108079311	ENSG00000197409	HIST1H3D	6	26197068	26197497	0.015212	10.659	2.21E-13	1.25E-07	0.032501	4.3862	8.43E-05	0.1485366	No	NA
ENSG00000006125	AP2B1	17	33913918	34053436	ENSG00000204420	C6orf25	6	31691121	31694491	0.015199	10.649	2.27E-13	1.28E-07	0.027822	4.3643	0.00023498	0.4088652	No	NA
ENSG00000006125	AP2B1	17	33913918	34053436	ENSG00000165406	8-Mar	10	45950035	46090354	0.015178	10.634	2.38E-13	1.34E-07	0.0046898	0.71856	0.63472	1	No	NA
ENSG00000154768	C17orf50	17	34087916	34092098	ENSG00000070182	SPTB	14	65216372	65346601	0.014564	11.9	2.67E-13	1.49E-07	0.009996	1.3184	0.23811	1	No	NA
ENSG00000142065	ZFP14	19	36827162	36870101	ENSG00000116962	NID1	1	236139130	236228462	0.014559	11.895	2.70E-13	1.51E-07	0.057479	11.172	1.81E-10	3.40E-07	No	NA
ENSG00000114378	HYAL1	3	50337320	50349812	ENSG00000120937	NPPB	1	11917521	11918988	0.013321	16.312	2.80E-13	1.56E-07	NA	NA	NA	NA	No	NA
ENSG00000163947	ARHGEF3	3	56761446	57113357	ENSG00000205038	PKHD1L1	8	110374706	110542559	0.015644	9.593	2.85E-13	1.58E-07	0.04072	4.8445	7.28E-06	0.01314768	No	NA
ENSG00000142082	SIRT3	11	215458	236431	ENSG00000138722	MMRN1	4	90800683	90875780	0.012625	20.604	2.92E-13	1.61E-07	0.0051068	1.1767	0.31949	1	Yes	No
ENSG00000099849	RASSF7	11	560404	564021	ENSG00000138722	MMRN1	4	90800683	90875780	0.015039	10.535	3.28E-13	1.81E-07	0.0086378	1.1377	0.33694	1	Yes	No
ENSG00000132139	GAS2L2	17	34071530	34079897	ENSG00000115649	CNPPD1	2	220036619	220042035	0.015584	9.5555	3.27E-13	1.81E-07	NA	NA	NA	NA	Yes	No
ENSG00000173402	DAG1	3	49506146	49573048	ENSG00000120937	NPPB	1	11917521	11918988	0.013243	16.215	3.37E-13	1.85E-07	NA	NA	NA	NA	No	NA
ENSG00000205045	SLFN12L	17	33800708	33864880	ENSG00000198876	DCAF12	9	34086385	34127397	0.01501	10.515	3.51E-13	1.92E-07	0.009805	1.2929	0.25047	1	No	NA
ENSG00000154768	C17orf50	17	34087916	34092098	ENSG00000022840	RNF10	12	120971283	121015397	0.014431	11.79	3.63E-13	1.98E-07	0.01572	2.0854	0.042643	1	Yes	No
ENSG00000106006	HOXA6	7	27185015	27190222	ENSG00000176358	TAC4	17	47915671	47925379	0.012512	20.417	3.83E-13	2.09E-07	0.0031365	0.9628	0.40963	1	No	NA
ENSG00000168374	ARF4	3	57557090	57583947	ENSG00000124491	F13A1	6	6144318	6321246	0.013779	13.502	4.18E-13	2.27E-07	0.011502	2.1317	0.059587	1	Yes	No
ENSG00000168374	ARF4	3	57557090	57583947	ENSG00000049323	LTBP1	2	33172039	33624576	0.013766	13.489	4.31E-13	2.33E-07	0.02566	4.8247	0.000232	0.403912	No	NA
ENSG00000172053	QARS	3	49133365	49142553	ENSG00000100228	RAB36	22	23487513	23506537	0.010778	52.689	4.53E-13	2.45E-07	0.00051481	0.47387	0.49139	1	No	NA
ENSG00000164076	CAMKV	3	49895422	49907655	ENSG00000204290	BTNL2	6	32361740	32374905	0.012416	20.257	4.83E-13	2.60E-07	NA	NA	NA	NA	No	NA
ENSG00000205045	SLFN12L	17	33800708	33864880	ENSG00000196961	AP2A1	19	50270225	50309510	0.01486	10.408	4.94E-13	2.66E-07	0.0043149	0.56584	0.78401	1	Yes	Yes
ENSG00000183763	TRAIP	3	49866034	49894007	ENSG00000120937	NPPB	1	11917521	11918988	0.013081	16.014	4.95E-13	2.66E-07	NA	NA	NA	NA	No	NA
ENSG00000108733	PEX12	17	33901814	33905882	ENSG00000198959	TGM2	20	36756863	36794980	0.013634	13.358	5.87E-13	3.14E-07	0.0044937	0.82695	0.53054	1	Yes	No
ENSG00000142082	SIRT3	11	215458	236431	ENSG00000049323	LTBP1	2	33172039	33624576	0.012332	20.12	5.90E-13	3.15E-07	0.012937	3.0046	0.0177	1	Yes	Yes
ENSG00000164631	ZNF12	7	6728064	6746554	ENSG00000124102	PI3	20	43803517	43805185	0.013623	13.347	6.02E-13	3.20E-07	0.021242	3.9759	0.001423	1	No	NA
ENSG00000108733	PEX12	17	33901814	33905882	ENSG00000123908	AGO2	8	141541264	141645718	0.013603	13.327	6.32E-13	3.35E-07	NA	NA	NA	NA	Yes	No
ENSG00000177951	BET1L	11	167784	207428	ENSG00000259207	ITGB3	17	45387505	45389182	0.015282	9.3678	6.46E-13	3.42E-07	0.03348	2.8656	0.0010463	1	Yes	No
ENSG00000177963	RIC8A	11	207511	215113	ENSG00000049323	LTBP1	2	33172039	33624576	0.013572	13.296	6.79E-13	3.58E-07	0.019478	2.267	0.021096	1	Yes	Yes
ENSG00000006125	AP2B1	17	33913918	34053436	ENSG00000153162	BMP6	6	7727030	7880334	0.014722	10.31	6.78E-13	3.58E-07	0.016673	2.5858	0.017242	1	Yes	Yes
ENSG00000006125	AP2B1	17	33913918	34053436	ENSG00000007968	E2F2	1	23832922	23857712	0.014717	10.306	6.86E-13	3.61E-07	0.0046421	0.71122	0.64064	1	No	NA
ENSG00000103047	TANGO6	16	68877509	69119083	ENSG00000255833	TIFAB	5	134787899	134788089	0.014713	10.304	6.92E-13	3.63E-07	0.005586	0.73347	0.64363	1	No	NA
ENSG00000067560	RHOA	3	49396578	49449635	ENSG00000100228	RAB36	22	23487513	23506537	0.01293	15.827	7.09E-13	3.70E-07	0.0068913	1.5908	0.17462	1	No	NA
ENSG00000163947	ARHGEF3	3	56761446	57113357	ENSG00000128266	GNAZ	22	23412540	23464889	0.015228	9.3341	7.31E-13	3.81E-07	0.025492	2.9854	0.0026195	1	No	NA
ENSG00000141150	RASL10B	17	34058668	34070540	ENSG00000167992	VWCE	11	61025762	61062896	0.015226	9.3326	7.35E-13	3.82E-07	0.021252	2.8352	0.0062857	1	Yes	No
ENSG00000174885	NLRP6	11	278365	285359	ENSG00000049323	LTBP1	2	33172039	33624576	0.014672	10.274	7.60E-13	3.94E-07	0.023447	2.433	0.009807	1	Yes	Yes
ENSG00000006125	AP2B1	17	33913918	34053436	ENSG00000140564	FURIN	15	91411822	91426688	0.014659	10.265	7.83E-13	4.05E-07	0.0044699	0.68472	0.66205	1	Yes	Yes
ENSG00000141150	RASL10B	17	34058668	34070540	ENSG00000130300	PLVAP	19	17462257	17488159	0.015195	9.3135	7.87E-13	4.06E-07	0.024106	3.2253	0.0021985	1	Yes	No
ENSG00000168374	ARF4	3	57557090	57583947	ENSG00000174175	SELP	1	169558087	169599431	0.013507	13.232	7.90E-13	4.07E-07	0.026246	4.9379	0.00018166	0.317905	Yes	No

ENSG00000174885	NLRP6	11	278365	285359	ENSG00000122786	CALD1	7	134429003	134655479	0.01465	10.259	7.99E-13	4.10E-07	0.015145	1.5583	0.12335	1	Yes	Yes
ENSG00000006125	AP2B1	17	33913918	34053436	ENSG00000204463	BAG6	6	31606805	31620170	0.014647	10.257	8.05E-13	4.12E-07	0.045988	7.3513	1.08E-07	0.00019948	Yes	Yes
ENSG00000076003	MCM6	2	136597196	136633996	ENSG00000204385	SLC44A4	6	31830969	31846823	0.015691	8.5513	8.32E-13	4.25E-07	NA	NA	NA	NA	No	NA
ENSG00000006125	AP2B1	17	33913918	34053436	ENSG00000183597	TANGO2	22	20004537	20053449	0.014625	10.241	8.47E-13	4.32E-07	NA	NA	NA	NA	No	NA
ENSG00000135404	CD63	12	56119107	56123491	ENSG00000105085	MED26	19	16698215	16739873	0.012853	15.731	8.51E-13	4.33E-07	0.010413	2.4123	0.047549	1	No	NA
ENSG00000136878	USP20	9	132597696	132644107	ENSG00000128294	TPST2	22	26921458	26992681	0.014046	11.47	8.85E-13	4.49E-07	0.00771	1.0145	0.4192	1	No	NA
ENSG00000163947	ARHGEF3	3	56761446	57113357	ENSG00000101335	MYL9	20	35169887	35178228	0.015108	9.2592	9.59E-13	4.85E-07	0.066836	8.174	1.02E-10	1.92E-07	No	NA
ENSG00000172660	TAF15	17	34136459	34174246	ENSG00000138867	GUCD1	22	24936406	24951284	0.013986	11.421	1.02E-12	5.13E-07	NA	NA	NA	NA	Yes	No
ENSG00000163947	ARHGEF3	3	56761446	57113357	ENSG00000122786	CALD1	7	134429003	134655479	0.015063	9.2316	1.06E-12	5.34E-07	0.06187	7.5266	9.16E-10	1.72E-06	No	NA
ENSG00000172660	TAF15	17	34136459	34174246	ENSG00000017483	SLC38A5	X	48316920	48328644	0.013954	11.394	1.09E-12	5.50E-07	0.021526	3.355	0.0028206	1	Yes	No
ENSG00000136371	MTHFS	15	80125927	80189721	ENSG00000243725	TTC4	1	55181495	55181816	0.01205	19.653	1.17E-12	5.83E-07	0.00033404	0.10225	0.95873	1	No	NA
ENSG00000136371	MTHFS	15	80125927	80189721	ENSG00000271723	MROH7	1	55124788	55181283	0.01205	19.653	1.17E-12	5.83E-07	NA	NA	NA	NA	No	NA
ENSG00000163947	ARHGEF3	3	56761446	57113357	ENSG00000061918	GUCY1B3	4	156680144	156728743	0.015018	9.2036	1.17E-12	5.85E-07	0.029695	3.4927	0.00055376	0.94637584	No	NA
ENSG00000006125	AP2B1	17	33913918	34053436	ENSG00000161911	TREML1	6	41117080	41122075	0.014474	10.134	1.19E-12	5.94E-07	0.016227	2.5154	0.020242	1	Yes	Yes
ENSG00000182179	UBA7	3	49842640	49851379	ENSG00000120937	NPPB	1	11917521	11918988	0.012708	15.552	1.20E-12	5.95E-07	NA	NA	NA	NA	No	NA
ENSG00000267260	CTD-2162K18.4	19	37264055	37264411	ENSG00000116962	NID1	1	236139130	236228462	0.014466	10.128	1.22E-12	6.02E-07	0.044459	6.0752	5.99E-07	0.00110036	No	NA
ENSG00000168374	ARF4	3	57557090	57583947	ENSG00000140479	PCSK6	15	101840818	102065405	0.013309	13.035	1.26E-12	6.21E-07	0.052812	10.215	1.54E-09	2.88E-06	Yes	No
ENSG00000172660	TAF15	17	34136459	34174246	ENSG00000160445	ZER1	9	131492065	131534693	0.013882	11.335	1.29E-12	6.36E-07	0.010705	1.6501	0.13028	1	Yes	No
ENSG00000177352	CCDC71	3	49199968	49203754	ENSG00000134595	SOX3	X	139585152	139587225	0.012006	19.581	1.29E-12	6.36E-07	NA	NA	NA	NA	No	NA
ENSG00000172716	SLFN11	17	33677324	33700720	ENSG00000022840	RNF10	12	120971283	121015397	0.016955	6.9347	1.32E-12	6.46E-07	0.029648	2.3145	0.0065259	1	Yes	No
ENSG00000092871	RFFL	17	33341759	33416338	ENSG00000022840	RNF10	12	120971283	121015397	0.013849	11.307	1.40E-12	6.82E-07	0.0063534	0.72971	0.66535	1	Yes	No
ENSG00000006125	AP2B1	17	33913918	34053436	ENSG00000160445	ZER1	9	131492065	131534693	0.014389	10.073	1.45E-12	7.08E-07	0.0075822	1.1651	0.32276	1	Yes	Yes
ENSG00000154760	SLFN13	17	33762115	33775856	ENSG00000164068	RNF123	3	49728563	49753910	0.016405	7.3175	1.54E-12	7.50E-07	0.012507	0.95938	0.48637	1	Yes	No
ENSG00000075239	ACAT1	11	107992243	108018503	ENSG00000197409	HIST1H3D	6	26197068	26197497	0.015402	8.3916	1.58E-12	7.70E-07	0.051207	5.469	2.42E-07	0.00044576	Yes	Yes
ENSG00000172660	TAF15	17	34136459	34174246	ENSG00000184481	FOXO4	X	70316047	70323385	0.013785	11.254	1.62E-12	7.83E-07	0.015601	2.4168	0.025293	1	No	NA
ENSG00000132436	FIGNL1	7	50511831	50518088	ENSG00000187699	C2orf88	2	190744335	191068210	0.010264	50.152	1.63E-12	7.87E-07	0.019555	18.35	2.03E-05	0.0364182	Yes	Yes
ENSG00000108733	PEX12	17	33901814	33905882	ENSG00000160445	ZER1	9	131492065	131534693	0.013178	12.906	1.71E-12	8.22E-07	0.0042708	0.78576	0.56	1	Yes	No
ENSG00000114316	USP4	3	49316247	49378145	ENSG00000103023	PRSS54	16	58318309	58328951	0.01315	12.878	1.82E-12	8.76E-07	NA	NA	NA	NA	No	NA
ENSG00000099849	RASSF7	11	560404	564021	ENSG00000205038	PKHD1L1	8	110374706	110542559	0.014266	9.9859	1.92E-12	9.21E-07	0.0055165	0.72429	0.65144	1	Yes	No
ENSG00000162086	ZNF75A	16	3358313	3368852	ENSG00000159882	ZNF230	19	44507100	44518078	0.01371	11.192	1.92E-12	9.21E-07	0.072705	11.957	5.92E-13	1.12E-09	No	NA
ENSG00000163947	ARHGEF3	3	56761446	57113357	ENSG00000148498	PARD3	10	34398488	35104253	0.014792	9.0629	1.95E-12	9.34E-07	0.017783	2.0663	0.036549	1	No	NA
ENSG00000205045	SLFN12L	17	33800708	33864880	ENSG00000198959	TGM2	20	36756863	36794980	0.014256	9.9788	1.96E-12	9.36E-07	0.005445	0.71485	0.65948	1	Yes	Yes
ENSG00000050405	LIMA1	12	50569571	50677329	ENSG00000181904	C5orf24	5	134181370	134182553	0.014789	9.061	1.97E-12	9.36E-07	0.013454	1.5564	0.13393	1	No	NA
ENSG00000163946	FAM208A	3	56658507	56717265	ENSG00000164116	GUCY1A3	4	156587863	156653501	0.014779	9.0548	2.01E-12	9.55E-07	0.0099012	1.3057	0.24418	1	Yes	No
ENSG00000205045	SLFN12L	17	33800708	33864880	ENSG00000103257	SLC7A5	16	87863629	87903094	0.014241	9.9683	2.03E-12	9.62E-07	0.0063025	0.82814	0.56394	1	Yes	Yes
ENSG00000112584	FAM120B	6	170599791	170716153	ENSG00000155269	GPR78	4	8560452	8589477	0.013617	11.116	2.38E-12	1.12E-06	NA	NA	NA	NA	No	NA
ENSG00000108733	PEX12	17	33901814	33905882	ENSG00000140564	FURIN	15	91411822	91426688	0.013017	12.745	2.49E-12	1.17E-06	0.0018462	0.33885	0.88947	1	Yes	No
ENSG00000145022	TCTA	3	49452253	49453908	ENSG00000182156	ENPP7	17	77704681	77716021	0.012393	15.162	2.53E-12	1.19E-06	NA	NA	NA	NA	No	NA
ENSG00000146576	C7orf26	7	6629648	6648357	ENSG00000124102	PI3	20	43803517	43805185	0.011726	19.118	2.54E-12	1.19E-06	0.020896	6.5307	0.00022634	0.39428428	No	NA
ENSG00000168374	ARF4	3	57557090	57583947	ENSG00000128266	GNAZ	22	23412540	23464889	0.012982	12.711	2.70E-12	1.26E-06	0.013944	2.5907	0.024492	1	Yes	No
ENSG00000163947	ARHGEF3	3	56761446	57113357	ENSG00000204420	C6orf25	6	31691121	31694491	0.014634	8.9644	2.79E-12	1.30E-06	0.027978	3.2849	0.0010532	1	No	NA
ENSG00000132139	GAS2L2	17	34071530	34079897	ENSG00000167671	UBXN6	19	4446046	4457819	0.014623	8.9579	2.86E-12	1.33E-06	0.0095475	1.1001	0.36054	1	Yes	No
ENSG00000171747	LGALS4	19	39292311	39304004	ENSG00000111291	GPRC5D	12	13093709	13105081	0.015612	7.6553	3.02E-12	1.40E-06	0.027749	2.6001	0.0040932	1	No	NA
ENSG00000154760	SLFN13	17	33762115	33775856	ENSG00000198876	DCAF12	9	34086385	34127397	0.016096	7.1773	3.03E-12	1.40E-06	0.011097	0.85005	0.59847	1	No	NA
ENSG00000186075	ZPBP2	17	38024417	38034149	ENSG00000162551	ALPL	1	21835858	21904905	0.0099868	48.783	3.25E-12	1.50E-06	0.00099116	0.91277	0.33963	1	Yes	No
ENSG00000006125	AP2B1	17	33913918	34053436	ENSG00000141084	RANBP10	16	67757005	67840555	0.014032	9.8198	3.27E-12	1.51E-06	0.01076	1.6587	0.12805	1	Yes	Yes
ENSG00000185379	RAD51D	17	33426811	33447063	ENSG00000113140	SPARC	5	151040657	151066726	0.011608	18.925	3.37E-12	1.55E-06	0.0073083	2.2528	0.08076	1	No	NA
ENSG00000121900	TMEM54	1	33360906	33367039	ENSG00000205364	MT1M	16	56667252	56667898	0.012886	12.616	3.37E-12	1.55E-06	NA	NA	NA	NA	No	NA
ENSG00000142082	SIRT3	11	215458	236431	ENSG00000151693	ASAP2	2	9346894	9541525	0.011599	18.909	3.45E-12	1.58E-06	0.0019676	0.45197	0.77101	1	Yes	Yes
ENSG00000172660	TAF15	17	34136459	34174246	ENSG00000198876	DCAF12	9	34086385	34127397	0.013457	10.983	3.44E-12	1.58E-06	0.0064221	0.9857	0.43357	1	Yes	No
ENSG00000092871	RFFL	17	33341759	33416338	ENSG00000079308	TNS1	2	218664512	218867718	0.013454	10.981	3.46E-12	1.58E-06	0.011659	1.3463	0.21671	1	No	NA
ENSG00000006125	AP2B1	17	33913918	34053436	ENSG00000164068	RNF123	3	49728563	49753910	0.01399	9.7901	3.60E-12	1.64E-06	0.01837	2.8539	0.0092761	1	Yes	Yes
ENSG00000141027	NCOR1	17	15932471	16119405	ENSG00000169429	IL8	4	74606223	74609433	0.013985	9.7868	3.64E-12	1.65E-06	0.042825	5.1061	3.08E-06	0.00559944	Yes	Yes
ENSG00000164077	MON1A	3	49946302	49967606	ENSG00000204290	BTNL2	6	32361740	32374905	0.011573	18.866	3.67E-12	1.66E-06	NA	NA	NA	NA	No	NA
ENSG00000168993	CPLX1	4	778745	819986	ENSG00000154263	ABCA10	17	67143355	67225001	0.012235	14.966	3.68E-12	1.67E-06	0.011271	2.0885	0.064656	1	No	NA
ENSG00000099849	RASSF7	11	560404	564021	ENSG00000259207	ITGB3	17	45387505	45389182	0.013976	9.78	3.72E-12	1.68E-06	0.012808	1.694	0.10684	1	Yes	No
ENSG00000205045	SLFN12L	17	33800708	33864880	ENSG00000161911	TREML1	6	41117080	41122075	0.013972	9.7771	3.75E-12	1.69E-06	0.0084875	1.1177	0.34947	1	Yes	Yes
ENSG00000177951	BET1L	11	167784	207428	ENSG00000205038	PKHD1L1	8	110374706	110542559	0.014491	8.8758	3.85E-12	1.73E-06	0.013569	1.1379	0.32773	1	Yes	Yes
ENSG00000197050	ZNF420	19	37498759	37621216	ENSG00000111291	GPRC5D	12	13093709	13105081	0.012212	14.937	3.89E-12	1.74E-06	0.0094458	2.1861	0.068704	1	No	NA
ENSG00000172660	TAF15	17	34136459	34174246	ENSG00000007968	E2F2	1	23832922	23857712	0.013403	10.938	3.90E-12	1.75E-06	0.0041589	0.63687	0.7008	1	No	NA
ENSG00000108733	PEX12	17	33901814	33905882	ENSG00000149260	CAPN5	11	76777979	76837201	0.012817	12.548	3.96E-12	1.77E-06	0.0083499	1.5426	0.17398	1	Yes	Yes
ENSG00000006125	AP2B1	17	33913918	34053436	ENSG00000164849	GPR146	7	1084212	1098897	0.013941	9.7553	4.03E-12	1.79E-06	0.01032	1.5902	0.14674	1	No	NA
ENSG00000172716	SLFN11	17	33677324	33700720	ENSG00000079308	TNS1	2	218664512	218867718	0.01643	6.7165	4.10E-12	1.82E-06	0.017231	1.3282	0.19649	1	No	NA
ENSG00000168374	ARF4	3	57557090	57583947	ENSG00000081377	CDC14B	9	99258489	99382112	0.012799	12.53	4.13E-12	1.83E-06	0.018089	3.3749	0.0050027	1	Yes	No
ENSG00000108733	PEX12	17	33901814	33905882	ENSG00000134779	TPGS2	18	34361762	34403834	0.0128	12.53	4.13E-12	1.83E-06	NA	NA	NA	NA	Yes	No
ENSG00000163947	ARHGEF3	3	56761446	57113357	ENSG00000035403	VCL	10	75757872	75879918	0.014456	8.8537	4.17E-12	1.84E-06	0.014708	1.7037	0.093598	1	No	NA
ENSG00000006125	AP2B1	17	33913918	34053436	ENSG00000174175	SELP	1	169558087	169599431	0.013921	9.7411	4.21E-12	1.85E-06	0.0065238	1.0014	0.42298	1	Yes	Yes

ENSG00000135211	TMEM60	7	77423045	77427897	ENSG00000010932	FMO1	1	171217638	171255117	0.011518	18.775	4.19E-12	1.85E-06	NA	NA	NA	NA	No	NA
ENSG00000205045	SLFN12L	17	33800708	33864880	ENSG00000017483	SLC38A5	X	48316920	48328644	0.013917	9.738	4.25E-12	1.87E-06	0.025374	3.3994	0.0013634	1	Yes	Yes
ENSG00000172660	TAF15	17	34136459	34174246	ENSG00000140564	FURIN	15	91411822	91426688	0.013356	10.9	4.34E-12	1.90E-06	0.0027118	0.41468	0.86957	1	Yes	No
ENSG00000205045	SLFN12L	17	33800708	33864880	ENSG00000130300	PLVAP	19	17462257	17488159	0.013907	9.7313	4.35E-12	1.90E-06	0.020377	2.716	0.0086107	1	Yes	Yes
ENSG00000205045	SLFN12L	17	33800708	33864880	ENSG00000128266	GNAZ	22	23412540	23464889	0.013884	9.7149	4.58E-12	2.00E-06	0.0088941	1.1717	0.31632	1	Yes	Yes
ENSG00000172716	SLFN11	17	33677324	33700720	ENSG00000115649	CNPPD1	2	220036619	220042035	0.016375	6.6937	4.62E-12	2.01E-06	NA	NA	NA	NA	Yes	No
ENSG00000164078	MST1R	3	49924435	49936696	ENSG00000182156	ENPP7	17	77704681	77716021	0.012136	14.844	4.65E-12	2.02E-06	NA	NA	NA	NA	No	NA
ENSG00000163947	ARHGEF3	3	56761446	57113357	ENSG00000119862	LGALSL	2	64681103	64688515	0.014393	8.8147	4.80E-12	2.08E-06	NA	NA	NA	NA	No	NA
ENSG00000154760	SLFN13	17	33762115	33775856	ENSG00000158828	PINK1	1	20959948	20978004	0.015867	7.0734	4.99E-12	2.16E-06	0.014013	1.0766	0.37647	1	Yes	No
ENSG00000004534	RBM6	3	49977440	50114683	ENSG00000131059	BPIFA3	20	31805116	31815564	0.010703	26.154	5.04E-12	2.17E-06	NA	NA	NA	NA	No	NA
ENSG00000135899	SP110	2	231033634	231090444	ENSG00000179909	ZNF154	19	58208735	58220579	0.015364	7.5319	5.21E-12	2.24E-06	0.023321	2.4196	0.010226	1	No	NA
ENSG00000154760	SLFN13	17	33762115	33775856	ENSG00000196961	AP2A1	19	50270225	50309510	0.01583	7.0567	5.41E-12	2.32E-06	0.013405	1.0292	0.41917	1	Yes	No
ENSG00000167914	GSDMA	17	38119226	38134019	ENSG00000162551	ALPL	1	21835858	21904905	0.010669	26.072	5.47E-12	2.34E-06	0.0076425	3.5388	0.029446	1	Yes	Yes
ENSG00000006125	AP2B1	17	33913918	34053436	ENSG00000017483	SLC38A5	X	48316920	48328644	0.013798	9.6535	5.58E-12	2.39E-06	0.03856	6.1162	2.68E-06	0.00488564	Yes	Yes
ENSG00000178105	DDX10	11	108535752	108811657	ENSG00000165480	SKA3	13	21727734	21750741	0.011371	18.533	5.96E-12	2.55E-06	0.0015469	0.47409	0.7004	1	No	NA
ENSG00000108592	FTSJ3	17	61896793	61906562	ENSG00000171611	PTCRA	6	42883727	42893573	0.0097399	47.565	6.00E-12	2.56E-06	1.28E-06	0.0011733	0.97268	1	No	NA
ENSG00000204991	SPIRE2	16	89884587	89937727	ENSG00000133250	ZNF414	19	8575462	8579044	0.012637	12.368	6.04E-12	2.57E-06	0.013449	1.78	0.087872	1	No	NA
ENSG00000172123	SLFN12	17	33738079	33760302	ENSG00000079308	TNS1	2	218664512	218867718	0.017605	5.7609	6.08E-12	2.58E-06	0.011005	1.6969	0.11859	1	No	NA
ENSG00000154768	C17orf50	17	34087916	34092098	ENSG00000158856	DMTN	8	21906506	21940038	0.013208	10.777	6.11E-12	2.59E-06	NA	NA	NA	NA	Yes	No
ENSG00000132139	GAS2L2	17	34071530	34079897	ENSG00000017483	SLC38A5	X	48316920	48328644	0.014277	8.7426	6.23E-12	2.63E-06	0.01357	1.57	0.12966	1	Yes	No
ENSG00000205045	SLFN12L	17	33800708	33864880	ENSG00000184481	FOXO4	X	70316047	70323385	0.013741	9.6136	6.34E-12	2.67E-06	0.012361	1.6341	0.12213	1	No	NA
ENSG00000160447	PKN3	9	131464802	131482562	ENSG00000164821	DEFA4	8	6793344	6795860	0.013188	10.76	6.40E-12	2.69E-06	0.053058	17.145	7.70E-11	1.45E-07	Yes	Yes
ENSG00000108733	PEX12	17	33901814	33905882	ENSG00000171552	BCL2L1	20	30252255	30311792	0.012611	12.342	6.42E-12	2.70E-06	0.0041799	0.76897	0.57222	1	No	NA
ENSG00000205045	SLFN12L	17	33800708	33864880	ENSG00000197256	KANK2	19	11274943	11308243	0.013725	9.6019	6.58E-12	2.76E-06	0.011422	1.5086	0.16055	1	No	NA
ENSG00000163946	FAM208A	3	56658507	56717265	ENSG00000085733	CTTN	11	70244510	70282690	0.014232	8.7149	6.89E-12	2.88E-06	0.015996	2.1225	0.038946	1	Yes	No
ENSG00000163946	FAM208A	3	56658507	56717265	ENSG00000061918	GUCY1B3	4	156680144	156728743	0.01423	8.7133	6.93E-12	2.89E-06	0.03291	4.4433	7.16E-05	0.1262308	Yes	No
ENSG00000108733	PEX12	17	33901814	33905882	ENSG00000130300	PLVAP	19	17462257	17488159	0.012574	12.306	6.99E-12	2.91E-06	0.015939	2.9673	0.011525	1	Yes	No
ENSG00000205045	SLFN12L	17	33800708	33864880	ENSG00000183597	TANGO2	22	20004537	20053449	0.013689	9.5765	7.14E-12	2.97E-06	NA	NA	NA	NA	No	NA
ENSG00000108733	PEX12	17	33901814	33905882	ENSG00000118046	STK11	19	1189406	1226662	0.012553	12.285	7.35E-12	3.05E-06	0.0016903	0.31018	0.90698	1	Yes	No
ENSG00000205045	SLFN12L	17	33800708	33864880	ENSG00000090674	MCOLN1	19	7587512	7595387	0.013671	9.5638	7.43E-12	3.08E-06	0.0033832	0.44324	0.87503	1	Yes	Yes
ENSG00000135423	GLS2	12	56865557	56882198	ENSG00000105085	MED26	19	16698215	16739873	0.014193	8.6906	7.52E-12	3.11E-06	0.0089268	0.91273	0.51321	1	No	NA
ENSG00000108733	PEX12	17	33901814	33905882	ENSG00000141084	RANBP10	16	67757005	67840555	0.01254	12.272	7.57E-12	3.12E-06	0.00287	0.5273	0.75574	1	Yes	No
ENSG00000186792	HYAL3	3	50331063	50333204	ENSG00000120937	NPPB	1	11917521	11918988	0.015179	7.4399	7.81E-12	3.21E-06	NA	NA	NA	NA	No	NA
ENSG00000132139	GAS2L2	17	34071530	34079897	ENSG00000079308	TNS1	2	218664512	218867718	0.014172	8.6773	7.89E-12	3.24E-06	0.0088359	1.0174	0.42091	1	No	NA
ENSG00000075407	ZNF37A	10	38383264	38412276	ENSG00000141194	OR4D1	17	56232494	56233517	0.013095	10.684	7.92E-12	3.25E-06	NA	NA	NA	NA	No	NA
ENSG00000149311	ATM	11	108094805	108239829	ENSG00000197409	HIST1H3D	6	26197068	26197497	0.013636	9.5388	8.05E-12	3.29E-06	0.05302	7.3106	1.49E-08	2.77E-05	Yes	No
ENSG00000164068	RNF123	3	49728563	49753910	ENSG00000120937	NPPB	1	11917521	11918988	0.011236	18.311	8.24E-12	3.37E-06	NA	NA	NA	NA	No	NA
ENSG00000142082	SIRT3	11	215458	236431	ENSG00000122786	CALD1	7	134429003	134655479	0.011216	18.278	8.64E-12	3.52E-06	0.013429	3.1206	0.014538	1	Yes	Yes
ENSG00000001617	SEMA3F	3	50192478	50226508	ENSG00000182156	ENPP7	17	77704681	77716021	0.012469	12.203	8.92E-12	3.63E-06	NA	NA	NA	NA	No	NA
ENSG00000172660	TAF15	17	34136459	34174246	ENSG00000165406	8-Mar	10	45950035	46090354	0.013039	10.638	9.01E-12	3.65E-06	0.0099465	1.5321	0.16442	1	No	NA
ENSG00000006125	AP2B1	17	33913918	34053436	ENSG00000011105	TSPAN9	12	3186521	3395730	0.013552	9.4791	9.75E-12	3.95E-06	0.05853	9.4808	4.02E-10	7.55E-07	Yes	Yes
ENSG00000163946	FAM208A	3	56658507	56717265	ENSG00000124491	F13A1	6	6144318	6321246	0.014067	8.6121	9.98E-12	4.04E-06	0.017681	2.3502	0.022108	1	Yes	No
ENSG00000006125	AP2B1	17	33913918	34053436	ENSG00000198959	TGM2	20	36756863	36794980	0.013536	9.4682	1.01E-11	4.07E-06	0.0068371	1.0498	0.39138	1	Yes	Yes
ENSG00000154760	SLFN13	17	33762115	33775856	ENSG00000100614	PPM1A	14	60712470	60765805	0.015539	6.925	1.02E-11	4.09E-06	0.025942	2.0175	0.020191	1	Yes	No
ENSG00000108733	PEX12	17	33901814	33905882	ENSG00000164068	RNF123	3	49728563	49753910	0.012412	12.146	1.02E-11	4.10E-06	0.011741	2.1765	0.054728	1	Yes	No
ENSG00000154760	SLFN13	17	33762115	33775856	ENSG00000149260	CAPN5	11	76777979	76837201	0.015493	6.9041	1.12E-11	4.51E-06	0.015019	1.155	0.31161	1	Yes	No
ENSG00000108733	PEX12	17	33901814	33905882	ENSG00000163554	SPTA1	1	158580278	158656488	0.012366	12.101	1.13E-11	4.54E-06	0.0057717	1.0635	0.37913	1	Yes	No
ENSG00000154760	SLFN13	17	33762115	33775856	ENSG00000167671	UBXN6	19	4446046	4457819	0.01548	6.8984	1.15E-11	4.60E-06	0.029395	2.2941	0.007066	1	Yes	No
ENSG00000006125	AP2B1	17	33913918	34053436	ENSG00000137193	PIM1	6	37137979	37143202	0.013477	9.4263	1.15E-11	4.60E-06	0.025033	3.9156	0.00071763	1	No	NA
ENSG00000163946	FAM208A	3	56658507	56717265	ENSG00000172572	PDE3A	12	20522179	20837315	0.013998	8.5694	1.16E-11	4.63E-06	NA	NA	NA	NA	Yes	No
ENSG00000171827	ZNF570	19	37958487	37976260	ENSG00000111291	GPRC5D	12	13093709	13105081	0.011741	14.354	1.18E-11	4.71E-06	0.021939	5.1424	0.00042312	0.72734328	No	NA
ENSG00000185379	RAD51D	17	33426811	33447063	ENSG00000120885	CLU	8	27454434	27472548	0.011077	18.048	1.21E-11	4.79E-06	0.0058722	1.8075	0.14415	1	No	NA
ENSG00000108733	PEX12	17	33901814	33905882	ENSG00000039068	CDH1	16	68771128	68869451	0.012324	12.059	1.25E-11	4.95E-06	0.012032	2.2311	0.049309	1	No	NA
ENSG00000177951	BET1L	11	167784	207428	ENSG00000138722	MMRN1	4	90800683	90875780	0.013954	8.5423	1.28E-11	5.06E-06	0.01076	0.89979	0.54005	1	Yes	No
ENSG00000178449	COX14	12	50505762	50514240	ENSG00000181904	C5orf24	5	134181370	134182553	0.011052	18.008	1.28E-11	5.06E-06	0.0028693	0.88052	0.45066	1	No	NA
ENSG00000178149	DALRD3	3	49053591	49056010	ENSG00000103023	PRSS54	16	58318309	58328951	0.011688	14.289	1.34E-11	5.28E-06	NA	NA	NA	NA	No	NA
ENSG00000006125	AP2B1	17	33913918	34053436	ENSG00000118046	STK11	19	1189406	1226662	0.013405	9.3752	1.36E-11	5.34E-06	0.0037843	0.57931	0.74705	1	No	NA
ENSG00000108278	ZNHIT3	17	34842473	34849850	ENSG00000070182	SPTB	14	65216372	65346601	0.012279	12.014	1.39E-11	5.45E-06	0.0059208	1.0912	0.36363	1	No	NA
ENSG00000163946	FAM208A	3	56658507	56717265	ENSG00000005961	ITGA2B	17	42449548	42466873	0.013905	8.5117	1.43E-11	5.61E-06	0.035493	4.8049	2.51E-05	0.0448788	Yes	No
ENSG00000266173	STRADA	17	61787851	61819330	ENSG00000171611	PTCRA	6	42883727	42893573	0.01225	11.986	1.49E-11	5.80E-06	0.0016327	0.2996	0.91316	1	No	NA
ENSG00000100201	DDX17	22	38883878	38903665	ENSG00000157502	MUM1L1	X	105412298	105452949	0.012245	11.98	1.50E-11	5.86E-06	NA	NA	NA	NA	No	NA
ENSG00000184886	PIGW	17	34892943	34895159	ENSG00000158856	DMTN	8	21906506	21940038	0.011624	14.21	1.56E-11	6.07E-06	NA	NA	NA	NA	Yes	No
ENSG00000172660	TAF15	17	34136459	34174246	ENSG00000183597	TANGO2	22	20004537	20053449	0.012789	10.431	1.60E-11	6.21E-06	NA	NA	NA	NA	No	NA
ENSG00000172123	SLFN12	17	33738079	33760302	ENSG00000238243	OR2W3	1	248058859	248060449	0.017133	5.6036	1.63E-11	6.30E-06	0.011293	1.7419	0.10823	1	Yes	No
ENSG00000172123	SLFN12	17	33738079	33760302	ENSG00000162722	TRIM58	1	248020501	248031169	0.017133	5.6036	1.63E-11	6.30E-06	0.012018	1.855	0.085716	1	Yes	No
ENSG00000002822	MAD1L1	7	1855429	2272878	ENSG00000150636	CCDC102B	18	66383765	66722426	0.013831	8.4659	1.69E-11	6.53E-06	0.012657	1.2991	0.23306	1	No	NA
ENSG00000088543	C3orf18	3	50595462	50605182	ENSG00000120937	NPPB	1	11917521	11918988	0.012175	11.911	1.77E-11	6.83E-06	NA	NA	NA	NA	No	NA

ENSG00000205045	SLFN12L	17	33800708	33864880	ENSG00000144567	FAM134A	2	220042939	220050201	0.013285	9.2898	1.79E-11	6.87E-06	0.012604	1.6668	0.11358	1	Yes	Yes
ENSG00000100211	CBY1	22	39052645	39069859	ENSG00000157502	MUM1L1	X	105412298	105452949	0.0093007	45.4	1.79E-11	6.88E-06	NA	NA	NA	NA	No	NA
ENSG00000186868	MAPT	17	43971748	44105700	ENSG00000183150	GPR19	12	12813825	12849141	0.01018	24.863	1.81E-11	6.93E-06	0.0039165	1.8067	0.16478	1	No	NA
ENSG00000142102	ATHL1	11	289138	296107	ENSG00000049323	LTBP1	2	33172039	33624576	0.018774	4.8519	1.82E-11	6.94E-06	0.00023728	0.10906	0.89669	1	Yes	Yes
ENSG00000100226	GTPBP1	22	39101728	39130402	ENSG00000157502	MUM1L1	X	105412298	105452949	0.01155	14.118	1.86E-11	7.10E-06	NA	NA	NA	NA	No	NA
ENSG00000177963	RIC8A	11	207511	215113	ENSG00000122786	CALD1	7	134429003	134655479	0.012152	11.888	1.87E-11	7.12E-06	0.017842	2.0733	0.035867	1	Yes	Yes
ENSG00000006125	AP2B1	17	33913918	34053436	ENSG00000124491	F13A1	6	6144318	6321246	0.013264	9.2749	1.87E-11	7.12E-06	0.0051386	0.78769	0.57963	1	Yes	Yes
ENSG00000172660	TAF15	17	34136459	34174246	ENSG00000141084	RANBP10	16	67757005	67840555	0.012713	10.368	1.91E-11	7.23E-06	0.0080412	1.2362	0.28513	1	Yes	No
ENSG00000172660	TAF15	17	34136459	34174246	ENSG00000166169	POLL	10	103338639	103346957	0.012711	10.367	1.91E-11	7.24E-06	0.03479	5.4967	1.33E-05	0.0239001	Yes	No
ENSG00000172660	TAF15	17	34136459	34174246	ENSG00000164068	RNF123	3	49728563	49753910	0.012711	10.366	1.92E-11	7.24E-06	0.013116	2.0267	0.059632	1	Yes	No
ENSG00000103051	COG4	16	70514471	70557468	ENSG00000255833	TIFAB	5	134787899	134788089	0.011528	14.091	1.96E-11	7.38E-06	0.0037387	0.68751	0.63299	1	No	NA
ENSG00000006125	AP2B1	17	33913918	34053436	ENSG00000196961	AP2A1	19	50270225	50309510	0.013235	9.2545	2.00E-11	7.53E-06	0.0046575	0.7136	0.63872	1	Yes	Yes
ENSG00000108733	PEX12	17	33901814	33905882	ENSG00000130830	MPP1	X	154006959	154049282	0.012116	11.853	2.03E-11	7.64E-06	0.0062449	1.1513	0.33156	1	No	NA
ENSG00000159110	IFNAR2	21	34602206	34637980	ENSG00000165949	IFI27	14	94571182	94583033	0.014249	7.7542	2.05E-11	7.70E-06	0.037114	4.3989	3.10E-05	0.055273	No	NA
ENSG00000141150	RASL10B	17	34058668	34070540	ENSG00000115649	CNPPD1	2	220036619	220042035	0.013721	8.3976	2.16E-11	8.11E-06	NA	NA	NA	NA	Yes	No
ENSG00000154760	SLFN13	17	33762115	33775856	ENSG00000164849	GPR146	7	1084212	1098897	0.015186	6.7652	2.19E-11	8.17E-06	0.026816	2.0873	0.015581	1	Yes	No
ENSG00000161298	ZNF382	19	37098454	37119499	ENSG00000116962	NID1	1	236139130	236228462	0.011475	14.025	2.22E-11	8.28E-06	0.036048	8.573	8.57E-07	0.00157174	No	NA
ENSG00000075399	VPS9D1	16	89773542	89785777	ENSG00000133250	ZNF414	19	8575462	8579044	0.012078	11.814	2.22E-11	8.28E-06	0.0086602	1.3322	0.23987	1	No	NA
ENSG00000168374	ARF4	3	57557090	57583947	ENSG00000138722	MMRN1	4	90800683	90875780	0.012055	11.792	2.34E-11	8.71E-06	0.029187	5.5078	5.27E-05	0.0933317	Yes	No
ENSG00000170425	ADORA2B	17	15848231	15879060	ENSG00000169429	IL8	4	74606223	74609433	0.010072	24.597	2.35E-11	8.72E-06	0.0087854	4.0727	0.01734	1	No	NA
ENSG00000221819	C16orf3	16	90095316	90096309	ENSG00000175718	RBMXL3	X	114423963	114427431	0.013163	9.2039	2.35E-11	8.72E-06	NA	NA	NA	NA	No	NA
ENSG00000197928	ZNF677	19	53731577	53758151	ENSG00000137942	FNBP1L	1	93913688	94020218	0.01079	17.575	2.41E-11	8.90E-06	0.0091548	1.6926	0.13368	1	No	NA
ENSG00000006125	AP2B1	17	33913918	34053436	ENSG00000140479	PCSK6	15	101840818	102065405	0.013143	9.1895	2.46E-11	9.09E-06	0.034305	5.4173	1.62E-05	0.0290952	Yes	Yes
ENSG00000006125	AP2B1	17	33913918	34053436	ENSG00000134779	TPGS2	18	34361762	34403834	0.013135	9.1839	2.51E-11	9.23E-06	NA	NA	NA	NA	Yes	Yes
ENSG00000214941	ZSWIM7	17	15879874	15897144	ENSG00000169429	IL8	4	74606223	74609433	0.010044	24.527	2.52E-11	9.28E-06	0.0099991	4.641	0.0098754	1	No	NA
ENSG00000108733	PEX12	17	33901814	33905882	ENSG00000184792	OSBP2	22	31089769	31303811	0.012011	11.749	2.59E-11	9.50E-06	0.017577	3.2776	0.0061144	1	Yes	No
ENSG00000168209	DDIT4	10	74033678	74035794	ENSG00000162931	TRIM17	1	228595641	228604562	0.012012	11.749	2.59E-11	9.50E-06	0.00072567	0.13304	0.98478	1	No	NA
ENSG00000146918	NCAPG2	7	158424003	158497520	ENSG00000121905	HPCA	1	33351595	33359230	0.012573	10.252	2.63E-11	9.62E-06	NA	NA	NA	NA	No	NA
ENSG00000108733	PEX12	17	33901814	33905882	ENSG00000007968	E2F2	1	23832922	23857712	0.01199	11.728	2.72E-11	9.94E-06	0.0056741	1.0454	0.38953	1	No	NA
ENSG00000157500	APPL1	3	57261765	57301820	ENSG00000138722	MMRN1	4	90800683	90875780	0.011384	13.913	2.75E-11	1.00E-05	0.0010103	0.30946	0.81856	1	Yes	No
ENSG00000108733	PEX12	17	33901814	33905882	ENSG00000154146	NRGN	11	124609829	124615878	0.011961	11.699	2.91E-11	1.06E-05	0.024775	4.6541	0.00033502	0.57891456	Yes	No
ENSG00000174885	NLRP6	11	278365	285359	ENSG00000151693	ASAP2	2	9346894	9541525	0.013071	9.1382	2.90E-11	1.06E-05	0.0094413	0.96584	0.46689	1	Yes	Yes
ENSG00000205045	SLFN12L	17	33800708	33864880	ENSG00000153162	BMP6	6	7727030	7880334	0.013063	9.1329	2.95E-11	1.07E-05	0.0044057	0.5778	0.77438	1	Yes	Yes
ENSG00000092871	RFFL	17	33341759	33416338	ENSG00000158828	PINK1	1	20959948	20978004	0.012518	10.207	2.98E-11	1.08E-05	0.014613	1.6924	0.096239	1	Yes	No
ENSG00000177963	RIC8A	11	207511	215113	ENSG00000138798	EGF	4	110834040	110933422	0.011947	11.685	3.01E-11	1.09E-05	0.005914	0.67895	0.71042	1	Yes	Yes
ENSG00000164068	RNF123	3	49728563	49753910	ENSG00000204290	BTNL2	6	32361740	32374905	0.010691	17.413	3.05E-11	1.10E-05	NA	NA	NA	NA	No	NA
ENSG00000172123	SLFN12	17	33738079	33760302	ENSG00000100828	NPRL3	16	138697	188859	0.016828	5.5022	3.07E-11	1.11E-05	0.011846	1.8282	0.090639	1	Yes	No
ENSG00000008300	CELSR3	3	48673902	48700348	ENSG00000100228	RAB36	22	23487513	23506537	0.012504	10.195	3.08E-11	1.11E-05	0.0093985	1.4469	0.19367	1	No	NA
ENSG00000198453	ZNF568	19	37407231	37488834	ENSG00000111291	GPRC5D	12	13093709	13105081	0.013022	9.104	3.23E-11	1.16E-05	0.041663	4.9615	4.96E-06	0.00898256	No	NA
ENSG00000142082	SIRT3	11	215458	236431	ENSG00000138798	EGF	4	110834040	110933422	0.010651	17.348	3.35E-11	1.20E-05	0.0028181	0.64787	0.62848	1	Yes	Yes
ENSG00000108733	PEX12	17	33901814	33905882	ENSG00000103342	GSPT1	16	11961985	12009939	0.011902	11.641	3.34E-11	1.20E-05	0.0061047	1.1252	0.34516	1	No	NA
ENSG00000163947	ARHGEF3	3	56761446	57113357	ENSG00000166091	CMTM5	14	23846017	23848981	0.013522	8.2744	3.37E-11	1.20E-05	0.069409	8.5122	3.21E-11	6.05E-08	No	NA
ENSG00000154760	SLFN13	17	33762115	33775856	ENSG00000144677	CTDSPL	3	37903451	38025960	0.014975	6.67	3.44E-11	1.22E-05	0.030671	2.3969	0.0047161	1	Yes	No
ENSG00000198231	DDX42	17	61851223	61896677	ENSG00000171611	PTCRA	6	42883727	42893573	0.010644	17.335	3.42E-11	1.22E-05	0.0031693	0.9729	0.40481	1	No	NA
ENSG00000168374	ARF4	3	57557090	57583947	ENSG00000172572	PDE3A	12	20522179	20837315	0.011886	11.625	3.47E-11	1.23E-05	NA	NA	NA	NA	No	NA
ENSG00000167077	MEI1	22	42095503	42195460	ENSG00000175556	LONRF3	X	118108581	118156888	0.0099092	24.195	3.50E-11	1.24E-05	0.0095183	2.9406	0.032298	1	No	NA
ENSG00000117868	ESYT2	7	158523686	158622944	ENSG00000121905	HPCA	1	33351595	33359230	0.014006	7.6199	3.51E-11	1.24E-05	NA	NA	NA	NA	No	NA
ENSG00000142102	ATHL1	11	289138	296107	ENSG00000259207	ITGB3	17	45387505	45389182	0.018403	4.7541	3.83E-11	1.35E-05	0.0010488	0.48244	0.61743	1	Yes	Yes
ENSG00000214706	IFRD2	3	50325840	50329487	ENSG00000120937	NPPB	1	11917521	11918988	0.01124	13.735	3.87E-11	1.36E-05	NA	NA	NA	NA	No	NA
ENSG00000144224	UBXN4	2	136499189	136542625	ENSG00000204385	SLC44A4	6	31830969	31846823	0.012403	10.112	3.87E-11	1.36E-05	NA	NA	NA	NA	No	NA
ENSG00000183763	TRAIP	3	49866034	49894007	ENSG00000182156	ENPP7	17	77704681	77716021	0.011231	13.724	3.95E-11	1.39E-05	NA	NA	NA	NA	No	NA
ENSG00000157368	IL34	16	70613798	70694585	ENSG00000255833	TIFAB	5	134787899	134788089	0.011827	11.567	3.97E-11	1.39E-05	0.0046216	0.70806	0.64318	1	No	NA
ENSG00000163947	ARHGEF3	3	56761446	57113357	ENSG00000163736	PPBP	4	74852755	74853914	0.013448	8.228	3.99E-11	1.40E-05	0.080599	10.005	1.98E-13	3.75E-10	No	NA
ENSG00000108733	PEX12	17	33901814	33905882	ENSG00000103257	SLC7A5	16	87863629	87903094	0.011808	11.547	4.16E-11	1.45E-05	0.0044014	0.8099	0.54264	1	No	NA
ENSG00000099849	RASSF7	11	560404	564021	ENSG00000049323	LTBP1	2	33172039	33624576	0.012892	9.0114	4.35E-11	1.51E-05	0.022829	3.0504	0.0035339	1	Yes	No
ENSG00000189164	ZNF527	19	37861963	37883968	ENSG00000111291	GPRC5D	12	13093709	13105081	0.012894	9.0128	4.33E-11	1.51E-05	0.029909	4.0256	0.00023611	0.41059529	No	NA
ENSG00000174885	NLRP6	11	278365	285359	ENSG00000144677	CTDSPL	3	37903451	38025960	0.01289	9.0101	4.36E-11	1.52E-05	0.032482	3.402	0.00040077	0.69052671	Yes	Yes
ENSG00000132139	GAS2L2	17	34071530	34079897	ENSG00000022840	RNF10	12	120971283	121015397	0.013399	8.1977	4.45E-11	1.54E-05	0.017504	2.0333	0.039932	1	Yes	No
ENSG00000172057	ORMDL3	17	38077294	38083854	ENSG00000162551	ALPL	1	21835858	21904905	0.011779	11.519	4.44E-11	1.54E-05	0.011743	1.8121	0.093712	1	Yes	Yes
ENSG00000163946	FAM208A	3	56658507	56717265	ENSG00000120885	CLU	8	27454434	27472548	0.013395	8.1953	4.48E-11	1.55E-05	0.030335	4.0848	0.00019958	0.34886584	Yes	No
ENSG00000185379	RAD51D	17	33426811	33447063	ENSG00000079308	TNS1	2	218664512	218867718	0.010531	17.15	4.47E-11	1.55E-05	0.0016892	0.51776	0.67014	1	No	NA
ENSG00000108733	PEX12	17	33901814	33905882	ENSG00000017483	SLC38A5	X	48316920	48328644	0.011762	11.502	4.62E-11	1.60E-05	0.031756	6.0084	1.76E-05	0.031592	Yes	No
ENSG00000073598	FNDC8	17	33448598	33454436	ENSG00000158856	DMTN	8	21906506	21940038	0.010493	17.088	4.90E-11	1.69E-05	NA	NA	NA	NA	No	NA
ENSG00000154760	SLFN13	17	33762115	33775856	ENSG00000140564	FURIN	15	91411822	91426688	0.014804	6.5925	4.98E-11	1.71E-05	0.0072972	0.55683	0.87715	1	Yes	No
ENSG00000132139	GAS2L2	17	34071530	34079897	ENSG00000164068	RNF123	3	49728563	49753910	0.013342	8.1626	5.04E-11	1.73E-05	0.015068	1.746	0.084212	1	Yes	No
ENSG00000163946	FAM208A	3	56658507	56717265	ENSG00000205038	PKHD1L1	8	110374706	110542559	0.013323	8.1508	5.26E-11	1.80E-05	0.01243	1.6434	0.11964	1	Yes	No
ENSG00000185614	FAM212A	3	49840687	49842463	ENSG00000120937	NPPB	1	11917521	11918988	0.010454	17.023	5.38E-11	1.84E-05	NA	NA	NA	NA	No	NA

ENSG00000006125	AP2B1	17	33913918	34053436	ENSG00000130159	ECSIT	19	11618272	11639989	0.012791	8.9403	5.45E-11	1.86E-05	0.014343	2.2191	0.039269	1	Yes	Yes
ENSG00000185379	RAD51D	17	33426811	33447063	ENSG00000144677	CTDSPL	3	37903451	38025960	0.010438	16.997	5.59E-11	1.91E-05	0.0047563	1.4624	0.22333	1	No	NA
ENSG00000177963	RIC8A	11	207511	215113	ENSG00000172572	PDE3A	12	20522179	20837315	0.011671	11.412	5.72E-11	1.95E-05	NA	NA	NA	NA	Yes	Yes
ENSG00000168374	ARF4	3	57557090	57583947	ENSG00000035403	VCL	10	75757872	75879918	0.011654	11.395	5.94E-11	2.02E-05	0.012255	2.273	0.045506	1	Yes	No
ENSG00000129595	EPB41L4A	5	111478138	111755013	ENSG00000169249	ZRSR2	X	15808595	15841383	0.012752	8.9122	5.96E-11	2.02E-05	0.0090504	1.1925	0.30419	1	No	NA
ENSG00000108733	PEX12	17	33901814	33905882	ENSG00000102145	GATA1	X	48644962	48652718	0.011642	11.384	6.10E-11	2.07E-05	0.01675	3.1209	0.0084324	1	Yes	Yes
ENSG00000129007	CALML4	15	68491879	68498417	ENSG00000180423	HARBI1	11	46624411	46638777	0.014708	6.5494	6.12E-11	2.07E-05	0.021104	1.7835	0.052591	1	No	NA
ENSG00000171097	CCBL1	9	131595221	131644773	ENSG00000128294	TPST2	22	26921458	26992681	0.012741	8.9044	6.11E-11	2.07E-05	0.0056008	0.85893	0.52468	1	No	NA
ENSG00000185379	RAD51D	17	33426811	33447063	ENSG00000022840	RNF10	12	120971283	121015397	0.010394	16.925	6.21E-11	2.09E-05	0.0056797	1.7479	0.1556	1	No	NA
ENSG00000163946	FAM208A	3	56658507	56717265	ENSG00000101335	MYL9	20	35169887	35178228	0.013247	8.1036	6.23E-11	2.10E-05	0.033233	4.4885	6.28E-05	0.1109676	Yes	No
ENSG00000205045	SLFN12L	17	33800708	33864880	ENSG00000140479	PCSK6	15	101840818	102065405	0.0127	8.8754	6.70E-11	2.25E-05	0.016746	2.2238	0.030339	1	Yes	Yes
ENSG00000172660	TAF15	17	34136459	34174246	ENSG00000198959	TGM2	20	36756863	36794980	0.012163	9.9134	6.72E-11	2.26E-05	0.0056453	0.86579	0.51951	1	Yes	No
ENSG00000163947	ARHGEF3	3	56761446	57113357	ENSG00000185052	SLC24A3	20	19193290	19703581	0.013208	8.0794	6.80E-11	2.28E-05	0.025877	3.0316	0.0022785	1	No	NA
ENSG00000108733	PEX12	17	33901814	33905882	ENSG00000101335	MYL9	20	35169887	35178228	0.011593	11.335	6.85E-11	2.29E-05	0.04265	8.1615	1.50E-07	0.00027675	Yes	No
ENSG00000205045	SLFN12L	17	33800708	33864880	ENSG00000102145	GATA1	X	48644962	48652718	0.012689	8.8681	6.86E-11	2.29E-05	0.018317	2.4363	0.017768	1	Yes	Yes
ENSG00000145088	EAF2	3	121554030	121605373	ENSG00000158022	TRIM63	1	26377795	26394927	0.012151	9.9042	6.90E-11	2.30E-05	NA	NA	NA	NA	No	NA
ENSG00000145022	TCTA	3	49452253	49453908	ENSG00000204290	BTNL2	6	32361740	32374905	0.010979	13.413	7.14E-11	2.38E-05	NA	NA	NA	NA	No	NA
ENSG00000066422	ZBTB11	3	101367733	101391057	ENSG00000179909	ZNF154	19	58208735	58220579	0.011559	11.302	7.40E-11	2.46E-05	0.044136	8.459	7.73E-08	0.00014285	No	NA
ENSG00000164818	HEATR2	7	769300	829190	ENSG00000129514	FOXA1	14	38059189	38069245	0.010948	13.374	7.69E-11	2.55E-05	NA	NA	NA	NA	No	NA
ENSG00000092871	RFFL	17	33341759	33416338	ENSG00000167671	UBXN6	19	4446046	4457819	0.012101	9.8626	7.74E-11	2.56E-05	0.010697	1.234	0.2756	1	Yes	No
ENSG00000108733	PEX12	17	33901814	33905882	ENSG00000130159	ECSIT	19	11618272	11639989	0.011531	11.274	7.90E-11	2.61E-05	0.011616	2.153	0.05723	1	Yes	No
ENSG00000163946	FAM208A	3	56658507	56717265	ENSG00000166086	JAM3	11	133938820	134018713	0.013131	8.0318	8.07E-11	2.66E-05	0.0067532	0.88777	0.51542	1	Yes	No
ENSG00000006125	AP2B1	17	33913918	34053436	ENSG00000104904	OAZ1	19	2271369	2273487	0.01261	8.8119	8.21E-11	2.70E-05	0.0082234	1.2645	0.27116	1	Yes	Yes
ENSG00000006125	AP2B1	17	33913918	34053436	ENSG00000171552	BCL2L1	20	30252255	30311792	0.012595	8.8011	8.49E-11	2.79E-05	0.0048268	0.73966	0.61777	1	No	NA
ENSG00000141150	RASL10B	17	34058668	34070540	ENSG00000167671	UBXN6	19	4446046	4457819	0.013104	8.0148	8.58E-11	2.82E-05	0.028605	3.845	0.00039349	0.67837676	Yes	No
ENSG00000255284	AP006621.5	11	777578	784297	ENSG00000165621	OXGR1	13	97637973	97646984	0.0086579	42.235	8.91E-11	2.92E-05	NA	NA	NA	NA	No	NA
ENSG00000243477	NAT6	3	50333833	50335514	ENSG00000120937	NPPB	1	11917521	11918988	0.012035	9.8079	9.01E-11	2.95E-05	NA	NA	NA	NA	No	NA
ENSG00000185379	RAD51D	17	33426811	33447063	ENSG00000161911	TREML1	6	41117080	41122075	0.010236	16.664	9.08E-11	2.97E-05	0.0026659	0.81794	0.48405	1	No	NA
ENSG00000205045	SLFN12L	17	33800708	33864880	ENSG00000141084	RANBP10	16	67757005	67840555	0.01256	8.7763	9.19E-11	3.00E-05	0.00645	0.84766	0.54788	1	Yes	Yes
ENSG00000172660	TAF15	17	34136459	34174246	ENSG00000196961	AP2A1	19	50270225	50309510	0.012022	9.7973	9.27E-11	3.02E-05	0.0075048	1.1531	0.32945	1	Yes	No
ENSG00000084093	REST	4	57774075	57802010	ENSG00000104381	GDAP1	8	75233749	75401107	0.010867	13.274	9.31E-11	3.03E-05	0.035626	8.469	1.04E-06	0.00190528	Yes	Yes
ENSG00000172660	TAF15	17	34136459	34174246	ENSG00000164849	GPR146	7	1084212	1098897	0.011998	9.7778	9.79E-11	3.18E-05	0.0094232	1.4507	0.19227	1	Yes	No
ENSG00000154760	SLFN13	17	33762115	33775856	ENSG00000204463	BAG6	6	31606805	31620170	0.014479	6.4456	1.00E-10	3.24E-05	0.042096	3.3289	9.36E-05	0.164736	Yes	No
ENSG00000187741	FANCA	16	89809208	89883065	ENSG00000133250	ZNF414	19	8575462	8579044	0.011429	11.173	1.00E-10	3.24E-05	0.0071624	1.1001	0.36028	1	No	NA
ENSG00000141150	RASL10B	17	34058668	34070540	ENSG00000149260	CAPN5	11	76777979	76837201	0.013033	7.971	1.00E-10	3.24E-05	0.012447	1.6457	0.11905	1	No	NA
ENSG00000136490	LIMD2	17	61776159	61778532	ENSG00000171611	PTCRA	6	42883727	42893573	0.013026	7.9665	1.02E-10	3.29E-05	0.0091923	1.0588	0.38996	1	No	NA
ENSG00000171298	GAA	17	78075355	78093678	ENSG00000187173	LCE2A	1	152670840	152671918	0.010187	16.584	1.02E-10	3.29E-05	NA	NA	NA	NA	No	NA
ENSG00000163947	ARHGEF3	3	56761446	57113357	ENSG00000019102	VSIG2	11	124618002	124621476	0.013022	7.9644	1.03E-10	3.31E-05	0.048355	5.7989	3.09E-07	0.00056825	No	NA
ENSG00000108733	PEX12	17	33901814	33905882	ENSG00000137198	GMPR	6	16238811	16295780	0.011415	11.159	1.03E-10	3.32E-05	0.0038068	0.70006	0.62348	1	No	NA
ENSG00000142102	ATHL1	11	289138	296107	ENSG00000122786	CALD1	7	134429003	134655479	0.017891	4.6193	1.07E-10	3.42E-05	0.0060712	2.8067	0.060919	1	Yes	Yes
ENSG00000157423	HYDIN	16	70841281	71264625	ENSG00000255833	TIFAB	5	134787899	134788089	0.010794	13.184	1.10E-10	3.53E-05	0.0014248	0.32709	0.85987	1	No	NA
ENSG00000184949	FAM227A	22	38974125	39052634	ENSG00000157502	MUM1L1	X	105412298	105452949	0.010791	13.181	1.11E-10	3.55E-05	NA	NA	NA	NA	No	NA
ENSG00000132139	GAS2L2	17	34071530	34079897	ENSG00000177830	CHID1	11	867357	915058	0.012978	7.9369	1.13E-10	3.62E-05	0.0043958	0.50388	0.85392	1	No	NA
ENSG00000006125	AP2B1	17	33913918	34053436	ENSG00000103257	SLC7A5	16	87863629	87903094	0.012464	8.709	1.14E-10	3.63E-05	0.017552	2.7245	0.012533	1	No	NA
ENSG00000114268	PFKFB4	3	48555117	48598605	ENSG00000100228	RAB36	22	23487513	23506537	0.012461	8.7063	1.15E-10	3.66E-05	0.010683	1.4099	0.19762	1	No	NA
ENSG00000185379	RAD51D	17	33426811	33447063	ENSG00000095303	PTGS1	9	125132824	125157982	0.01013	16.491	1.17E-10	3.71E-05	0.013123	4.0691	0.0069393	1	No	NA
ENSG00000267260	CTD-2162K18.4	19	37264055	37264411	ENSG00000111291	GPRC5D	12	13093709	13105081	0.012454	8.7014	1.17E-10	3.71E-05	0.014348	1.9008	0.06635	1	No	NA
ENSG00000182179	UBA7	3	49842640	49851379	ENSG00000204290	BTNL2	6	32361740	32374905	0.010744	13.122	1.24E-10	3.94E-05	NA	NA	NA	NA	No	NA
ENSG00000006125	AP2B1	17	33913918	34053436	ENSG00000100325	ASCC2	22	30184597	30234265	0.012419	8.6772	1.26E-10	3.99E-05	0.0079764	1.2262	0.29023	1	No	NA
ENSG00000172716	SLFN11	17	33677324	33700720	ENSG00000158828	PINK1	1	20959948	20978004	0.014807	6.0433	1.32E-10	4.17E-05	0.011655	0.89328	0.55347	1	Yes	No
ENSG00000168374	ARF4	3	57557090	57583947	ENSG00000061918	GUCY1B3	4	156680144	156728743	0.011305	11.05	1.34E-10	4.21E-05	0.018291	3.4133	0.0046209	1	Yes	No
ENSG00000183763	TRAIP	3	49866034	49894007	ENSG00000204290	BTNL2	6	32361740	32374905	0.010696	13.063	1.39E-10	4.38E-05	NA	NA	NA	NA	No	NA
ENSG00000144488	ESPNL	2	239008798	239041928	ENSG00000088854	C20orf194	20	3229951	3388272	0.0093427	22.799	1.40E-10	4.39E-05	0.00072571	0.33371	0.71635	1	No	NA
ENSG00000164053	ATRIP	3	48488114	48506061	ENSG00000134595	SOX3	X	139585152	139587225	0.013374	7.2716	1.41E-10	4.42E-05	NA	NA	NA	NA	No	NA
ENSG00000243477	NAT6	3	50333833	50335514	ENSG00000182156	ENPP7	17	77704681	77716021	0.011835	9.6435	1.42E-10	4.45E-05	NA	NA	NA	NA	No	NA
ENSG00000006125	AP2B1	17	33913918	34053436	ENSG00000102145	GATA1	X	48644962	48652718	0.012363	8.6376	1.43E-10	4.47E-05	0.023502	3.6704	0.0013114	1	Yes	Yes
ENSG00000006125	AP2B1	17	33913918	34053436	ENSG00000090674	MCOLN1	19	7587512	7595387	0.012363	8.6371	1.43E-10	4.47E-05	0.005334	0.8178	0.55614	1	Yes	Yes
ENSG00000177946	CENPBD1	16	90036206	90038942	ENSG00000175718	RBMXL3	X	114423963	114427431	0.012868	7.8685	1.45E-10	4.52E-05	NA	NA	NA	NA	No	NA
ENSG00000177963	RIC8A	11	207511	215113	ENSG00000061918	GUCY1B3	4	156680144	156728743	0.011259	11.004	1.49E-10	4.63E-05	0.015691	1.8193	0.069923	1	Yes	Yes
ENSG00000177963	RIC8A	11	207511	215113	ENSG00000151693	ASAP2	2	9346894	9541525	0.011248	10.993	1.52E-10	4.74E-05	0.0074723	0.85919	0.55069	1	Yes	Yes
ENSG00000108733	PEX12	17	33901814	33905882	ENSG00000196961	AP2A1	19	50270225	50309510	0.011241	10.987	1.55E-10	4.81E-05	0.0023418	0.43002	0.82787	1	Yes	No
ENSG00000196071	OR2L13	1	248100493	248264224	ENSG00000166086	JAM3	11	133938820	134018713	0.013308	7.2355	1.63E-10	5.05E-05	0.010779	1.1042	0.35684	1	Yes	No
ENSG00000006125	AP2B1	17	33913918	34053436	ENSG00000085733	CTTN	11	70244510	70282690	0.012293	8.5875	1.68E-10	5.19E-05	0.0064859	0.99556	0.4269	1	Yes	Yes
ENSG00000189042	ZNF567	19	37178530	37218603	ENSG00000111291	GPRC5D	12	13093709	13105081	0.0099732	16.232	1.70E-10	5.26E-05	0.0044802	1.0317	0.38974	1	No	NA
ENSG00000172660	TAF15	17	34136459	34174246	ENSG00000103257	SLC7A5	16	87863629	87903094	0.011747	9.5706	1.74E-10	5.36E-05	0.0091353	1.406	0.20921	1	No	NA
ENSG00000108733	PEX12	17	33901814	33905882	ENSG00000124491	F13A1	6	6144318	6321246	0.011183	10.93	1.77E-10	5.45E-05	0.0053819	0.9913	0.42186	1	Yes	No
ENSG00000108733	PEX12	17	33901814	33905882	ENSG00000204463	BAG6	6	31606805	31620170	0.011173	10.92	1.81E-10	5.57E-05	0.036329	6.9064	2.43E-06	0.00443232	Yes	No

ENSG00000172716	SLFN11	17	33677324	33700720	ENSG00000164068	RNF123	3	49728563	49753910	0.014657	5.9808	1.82E-10	5.58E-05	0.01308	1.004	0.44293	1	Yes	No
ENSG00000114735	HEMK1	3	50606583	50622366	ENSG00000103023	PRSS54	16	58318309	58328951	0.0092323	22.527	1.83E-10	5.61E-05	NA	NA	NA	NA	No	NA
ENSG00000168374	ARF4	3	57557090	57583947	ENSG00000185245	GP1BA	17	4835592	4838325	0.011166	10.913	1.84E-10	5.64E-05	0.028782	5.4292	6.25E-05	0.1105	Yes	No
ENSG00000177352	CCDC71	3	49199968	49203754	ENSG00000103023	PRSS54	16	58318309	58328951	0.0099338	16.167	1.87E-10	5.72E-05	NA	NA	NA	NA	No	NA
ENSG00000188895	MSL1	17	38278551	38291643	ENSG00000196663	TECPR2	14	102829300	102968818	0.014182	6.3115	1.90E-10	5.79E-05	0.016539	5.146	0.0015642	1	Yes	No
ENSG00000185379	RAD51D	17	33426811	33447063	ENSG00000101335	MYL9	20	35169887	35178228	0.0099233	16.15	1.92E-10	5.85E-05	0.01619	5.0358	0.001823	1	No	NA
ENSG00000108278	ZNHIT3	17	34842473	34849850	ENSG00000158856	DMTN	8	21906506	21940038	0.011134	10.881	1.98E-10	6.03E-05	NA	NA	NA	NA	Yes	No
ENSG00000177951	BET1L	11	167784	207428	ENSG00000172572	PDE3A	12	20522179	20837315	0.012719	7.7765	2.02E-10	6.13E-05	NA	NA	NA	NA	Yes	Yes
ENSG00000073969	NSF	17	44668035	44834830	ENSG00000183150	GPR19	12	12813825	12849141	0.01321	7.1814	2.02E-10	6.13E-05	0.010575	1.63	0.13562	1	No	NA
ENSG00000177963	RIC8A	11	207511	215113	ENSG00000095303	PTGS1	9	125132824	125157982	0.011121	10.868	2.04E-10	6.19E-05	0.010286	1.1861	0.30416	1	Yes	Yes
ENSG00000103044	HAS3	16	69139467	69151571	ENSG00000255833	TIFAB	5	134787899	134788089	0.01052	12.846	2.10E-10	6.36E-05	0.0084438	1.9522	0.099807	1	No	NA
ENSG00000167914	GSDMA	17	38119226	38134019	ENSG00000196663	TECPR2	14	102829300	102968818	0.0091712	22.377	2.12E-10	6.41E-05	0.008228	3.8121	0.022452	1	Yes	Yes
ENSG00000205045	SLFN12L	17	33800708	33864880	ENSG00000160445	ZER1	9	131492065	131534693	0.012184	8.5107	2.14E-10	6.46E-05	0.0024163	0.31626	0.94685	1	Yes	Yes
ENSG00000087191	PSMC5	17	61906854	61909379	ENSG00000171611	PTCRA	6	42883727	42893573	0.011099	10.847	2.15E-10	6.47E-05	0.002803	0.51495	0.76511	1	No	NA
ENSG00000142102	ATHL1	11	289138	296107	ENSG00000138722	MMRN1	4	90800683	90875780	0.017533	4.5253	2.17E-10	6.52E-05	0.0018766	0.86394	0.42184	1	Yes	Yes
ENSG00000154760	SLFN13	17	33762115	33775856	ENSG00000160445	ZER1	9	131492065	131534693	0.014118	6.2828	2.17E-10	6.53E-05	0.015721	1.2099	0.27094	1	Yes	No
ENSG00000114383	TUSC2	3	50362338	50365682	ENSG00000100228	RAB36	22	23487513	23506537	0.0098675	16.058	2.19E-10	6.58E-05	0.00010629	0.048846	0.95233	1	No	NA
ENSG00000163947	ARHGEF3	3	56761446	57113357	ENSG00000011105	TSPAN9	12	3186521	3395730	0.01268	7.7525	2.20E-10	6.58E-05	0.063573	7.7478	4.33E-10	8.13E-07	No	NA
ENSG00000141741	MIEN1	17	37885409	37886014	ENSG00000162551	ALPL	1	21835858	21904905	0.011086	10.833	2.22E-10	6.63E-05	0.0087165	1.6109	0.15446	1	Yes	No
ENSG00000132139	GAS2L2	17	34071530	34079897	ENSG00000118046	STK11	19	1189406	1226662	0.012671	7.7465	2.24E-10	6.70E-05	0.0071328	0.81987	0.58509	1	Yes	No
ENSG00000172660	TAF15	17	34136459	34174246	ENSG00000167992	VWCE	11	61025762	61062896	0.011633	9.4764	2.25E-10	6.72E-05	0.031076	4.8911	6.22E-05	0.1100318	Yes	No
ENSG00000142082	SIRT3	11	215458	236431	ENSG00000204420	C6orf25	6	31691121	31694491	0.0098497	16.029	2.29E-10	6.81E-05	0.004828	1.1122	0.34938	1	Yes	Yes
ENSG00000073605	GSDMB	17	38060848	38074903	ENSG00000162551	ALPL	1	21835858	21904905	0.010473	12.788	2.35E-10	6.99E-05	0.0015799	0.36277	0.8352	1	Yes	Yes
ENSG00000148719	DNAJB12	10	74092588	74114988	ENSG00000162931	TRIM17	1	228595641	228604562	0.012118	8.4643	2.48E-10	7.36E-05	0.0022218	0.33959	0.91601	1	No	NA
ENSG00000168374	ARF4	3	57557090	57583947	ENSG00000095303	PTGS1	9	125132824	125157982	0.011034	10.782	2.50E-10	7.41E-05	0.023837	4.4735	0.00049358	0.84550254	Yes	No
ENSG00000205045	SLFN12L	17	33800708	33864880	ENSG00000171552	BCL2L1	20	30252255	30311792	0.012107	8.4559	2.55E-10	7.54E-05	0.0022468	0.29402	0.95636	1	Yes	Yes
ENSG00000001617	SEMA3F	3	50192478	50226508	ENSG00000204290	BTNL2	6	32361740	32374905	0.01102	10.769	2.58E-10	7.63E-05	NA	NA	NA	NA	No	NA
ENSG00000177963	RIC8A	11	207511	215113	ENSG00000144677	CTDSPL	3	37903451	38025960	0.011019	10.767	2.59E-10	7.64E-05	0.017815	2.07	0.036182	1	Yes	Yes
ENSG00000163947	ARHGEF3	3	56761446	57113357	ENSG00000113140	SPARC	5	151040657	151066726	0.012605	7.7056	2.60E-10	7.66E-05	0.030418	3.5803	0.00042129	0.7246188	No	NA
ENSG00000141150	RASL10B	17	34058668	34070540	ENSG00000029534	ANK1	8	41510739	41754280	0.012603	7.7044	2.61E-10	7.68E-05	0.0062211	0.81738	0.57286	1	Yes	No
ENSG00000228008	CTD-2330K9.3	3	49943495	49954370	ENSG00000131059	BPIFA3	20	31805116	31815564	0.0097874	15.927	2.65E-10	7.80E-05	NA	NA	NA	NA	No	NA
ENSG00000172660	TAF15	17	34136459	34174246	ENSG00000149260	CAPN5	11	76777979	76837201	0.011552	9.4101	2.71E-10	7.95E-05	0.013705	2.119	0.04887	1	No	NA
ENSG00000088543	C3orf18	3	50595462	50605182	ENSG00000204290	BTNL2	6	32361740	32374905	0.010998	10.747	2.71E-10	7.95E-05	NA	NA	NA	NA	No	NA
ENSG00000154760	SLFN13	17	33762115	33775856	ENSG00000184481	FOXO4	X	70316047	70323385	0.014007	6.2323	2.76E-10	8.07E-05	0.027631	2.1525	0.012184	1	Yes	No
ENSG00000108379	WNT3	17	44839872	44910424	ENSG00000183150	GPR19	12	12813825	12849141	0.010392	12.687	2.85E-10	8.32E-05	0.011184	2.0721	0.066678	1	No	NA
ENSG00000114388	NPRL2	3	50384919	50387453	ENSG00000182156	ENPP7	17	77704681	77716021	0.009047	22.071	2.87E-10	8.39E-05	NA	NA	NA	NA	No	NA
ENSG00000114268	PFKFB4	3	48555117	48598605	ENSG00000103023	PRSS54	16	58318309	58328951	0.012051	8.4165	2.89E-10	8.41E-05	NA	NA	NA	NA	No	NA
ENSG00000172660	TAF15	17	34136459	34174246	ENSG00000204463	BAG6	6	31606805	31620170	0.011514	9.379	2.95E-10	8.58E-05	0.024712	3.8641	0.00081491	1	Yes	No
ENSG00000142082	SIRT3	11	215458	236431	ENSG00000144677	CTDSPL	3	37903451	38025960	0.0097378	15.845	2.99E-10	8.69E-05	0.006279	1.4485	0.21601	1	Yes	No
ENSG00000108733	PEX12	17	33901814	33905882	ENSG00000161911	TREML1	6	41117080	41122075	0.010956	10.705	2.99E-10	8.69E-05	0.010332	1.9126	0.089741	1	Yes	No
ENSG00000186792	HYAL3	3	50331063	50333204	ENSG00000204290	BTNL2	6	32361740	32374905	0.013497	6.6041	3.05E-10	8.83E-05	NA	NA	NA	NA	No	NA
ENSG00000185294	SPPL2C	17	43922256	43924438	ENSG00000183150	GPR19	12	12813825	12849141	0.0090122	21.985	3.13E-10	9.05E-05	0.0020269	0.93326	0.39364	1	No	NA
ENSG00000163946	FAM208A	3	56658507	56717265	ENSG00000113140	SPARC	5	151040657	151066726	0.012519	7.6528	3.14E-10	9.07E-05	0.015584	2.0671	0.044578	1	Yes	No
ENSG00000132139	GAS2L2	17	34071530	34079897	ENSG00000158828	PINK1	1	20959948	20978004	0.012518	7.6521	3.15E-10	9.08E-05	0.020433	2.3806	0.015345	1	Yes	No
ENSG00000006125	AP2B1	17	33913918	34053436	ENSG00000039068	CDH1	16	68771128	68869451	0.012003	8.3829	3.21E-10	9.25E-05	0.017991	2.7939	0.010669	1	No	NA
ENSG00000198093	ZNF649	19	52399725	52408293	ENSG00000164659	KIAA1324L	7	86506222	86689015	0.012507	7.6454	3.22E-10	9.26E-05	0.027692	3.2503	0.0011713	1	Yes	No
ENSG00000162722	TRIM58	1	248020501	248031169	ENSG00000166086	JAM3	11	133938820	134018713	0.012508	7.6457	3.22E-10	9.26E-05	0.0060649	0.93055	0.47202	1	Yes	Yes
ENSG00000177951	BET1L	11	167784	207428	ENSG00000151693	ASAP2	2	9346894	9541525	0.012501	7.6417	3.26E-10	9.37E-05	0.010495	0.87742	0.56229	1	Yes	Yes
ENSG00000131771	PPP1R1B	17	37782993	37792879	ENSG00000112053	SLC26A8	6	35911291	35992645	0.012984	7.0571	3.31E-10	9.48E-05	0.021403	2.2162	0.019145	1	No	NA
ENSG00000163947	ARHGEF3	3	56761446	57113357	ENSG00000183785	TUBA8	22	18593097	18593634	0.012495	7.6374	3.32E-10	9.49E-05	0.044748	5.3461	1.39E-06	0.00254231	No	NA
ENSG00000006125	AP2B1	17	33913918	34053436	ENSG00000123908	AGO2	8	141541264	141645718	0.011984	8.3693	3.35E-10	9.58E-05	NA	NA	NA	NA	Yes	Yes
ENSG00000132139	GAS2L2	17	34071530	34079897	ENSG00000138867	GUCD1	22	24936406	24951284	0.012484	7.6311	3.39E-10	9.68E-05	NA	NA	NA	NA	Yes	No
ENSG00000118640	VAMP8	2	85788685	85809154	ENSG00000169439	SDC2	8	97505579	97624000	0.011446	9.3228	3.44E-10	9.82E-05	0.035669	4.8296	2.34E-05	0.041886	Yes	Yes
ENSG00000172660	TAF15	17	34136459	34174246	ENSG00000118046	STK11	19	1189406	1226662	0.011435	9.3135	3.53E-10	0.00010044	0.0081283	1.2497	0.27839	1	Yes	No
ENSG00000108733	PEX12	17	33901814	33905882	ENSG00000197993	KEL	7	142638201	142659768	0.010884	10.634	3.53E-10	0.00010044	0.022602	4.2365	0.00081878	1	Yes	No
ENSG00000177352	CCDC71	3	49199968	49203754	ENSG00000100228	RAB36	22	23487513	23506537	0.0096578	15.714	3.62E-10	0.00010272	0.0080767	2.4916	0.058896	1	No	NA
ENSG00000154760	SLFN13	17	33762115	33775856	ENSG00000198959	TGM2	20	36756863	36794980	0.013878	6.1744	3.63E-10	0.0001028	0.0068024	0.51881	0.90358	1	Yes	No
ENSG00000076344	RGS11	16	320522	325940	ENSG00000167384	ZNF180	19	44979854	45004576	0.011419	9.3007	3.66E-10	0.00010364	0.0052883	0.69418	0.67709	1	No	NA
ENSG00000120088	CRHR1	17	43699267	43913194	ENSG00000183150	GPR19	12	12813825	12849141	0.010282	12.552	3.68E-10	0.00010415	0.0049804	0.91697	0.46913	1	No	NA
ENSG00000140479	PCSK6	15	101840818	102065405	ENSG00000110680	CALCA	11	14988214	14993900	0.013405	6.5587	3.71E-10	0.00010482	NA	NA	NA	NA	No	NA
ENSG00000006125	AP2B1	17	33913918	34053436	ENSG00000184792	OSBP2	22	31089769	31303811	0.011937	8.3361	3.72E-10	0.00010498	0.018771	2.9173	0.0079949	1	Yes	Yes
ENSG00000154760	SLFN13	17	33762115	33775856	ENSG00000166169	POLL	10	103338639	103346957	0.013862	6.1672	3.75E-10	0.00010558	0.031259	2.4443	0.0039046	1	Yes	No
ENSG00000006125	AP2B1	17	33913918	34053436	ENSG00000196914	ARHGEF12	11	120207787	120360645	0.011933	8.3334	3.76E-10	0.00010558	0.026102	4.0872	0.0004691	0.8045065	No	NA
ENSG00000168374	ARF4	3	57557090	57583947	ENSG00000122786	CALD1	7	134429003	134655479	0.010857	10.607	3.76E-10	0.00010563	0.032462	6.1465	1.30E-05	0.023374	Yes	No
ENSG00000168374	ARF4	3	57557090	57583947	ENSG00000183785	TUBA8	22	18593097	18593634	0.010844	10.595	3.87E-10	0.00010858	0.023979	4.5009	0.00046543	0.79867788	No	NA
ENSG00000108733	PEX12	17	33901814	33905882	ENSG00000090674	MCOLN1	19	7587512	7595387	0.010815	10.566	4.15E-10	0.00011606	0.0020194	0.3707	0.86887	1	Yes	No
ENSG00000163946	FAM208A	3	56658507	56717265	ENSG00000166091	CMTM5	14	23846017	23848981	0.012388	7.5712	4.20E-10	0.00011743	0.065831	9.2014	4.97E-11	9.37E-08	Yes	No

ENSG00000167914	GSDMA	17	38119226	38134019	ENSG00000146592	CREB5	7	28338940	28865511	0.0088789	21.657	4.33E-10	0.00012086	0.0027455	1.265	0.28272	1	Yes	Yes
ENSG00000141150	RASL10B	17	34058668	34070540	ENSG00000166169	POLL	10	103338639	103346957	0.012357	7.5524	4.49E-10	0.00012528	0.02176	2.9044	0.005228	1	Yes	No
ENSG00000180353	HCLS1	3	121350246	121379774	ENSG00000158022	TRIM63	1	26377795	26394927	0.013316	6.5144	4.50E-10	0.00012536	NA	NA	NA	NA	No	NA
ENSG00000163946	FAM208A	3	56658507	56717265	ENSG00000163736	PPBP	4	74852755	74853914	0.012349	7.5475	4.57E-10	0.00012715	0.051727	7.1224	2.62E-08	4.86E-05	Yes	No
ENSG00000108733	PEX12	17	33901814	33905882	ENSG00000180354	C7orf41	7	30174426	30202378	0.010766	10.518	4.63E-10	0.00012871	0.0094357	1.7451	0.12172	1	Yes	No
ENSG00000172057	ORMDL3	17	38077294	38083854	ENSG00000196663	TECPR2	14	102829300	102968818	0.010765	10.516	4.65E-10	0.00012903	0.0091823	1.4133	0.20636	1	Yes	Yes
ENSG00000168374	ARF4	3	57557090	57583947	ENSG00000069966	GNB5	15	52416670	52483566	0.010757	10.509	4.74E-10	0.00013118	0.027481	5.1768	0.00010827	0.19033866	No	NA
ENSG00000213171	LINGO4	1	151772740	151775193	ENSG00000124491	F13A1	6	6144318	6321246	0.011303	9.2048	4.77E-10	0.00013193	0.0091299	1.4051	0.20954	1	No	NA
ENSG00000205045	SLFN12L	17	33800708	33864880	ENSG00000154146	NRGN	11	124609829	124615878	0.011824	8.2564	4.80E-10	0.00013246	0.010384	1.3701	0.21448	1	Yes	Yes
ENSG00000114354	TFG	3	100428205	100467810	ENSG00000196724	ZNF418	19	58433252	58446761	0.012809	6.9603	4.85E-10	0.00013388	0.036636	4.34	3.76E-05	0.0667776	No	NA
ENSG00000172716	SLFN11	17	33677324	33700720	ENSG00000166947	EPB42	15	43398423	43513481	0.014189	5.7872	4.88E-10	0.00013419	0.016432	1.2655	0.23367	1	Yes	No
ENSG00000178252	WDR6	3	49045041	49052151	ENSG00000134595	SOX3	X	139585152	139587225	0.0088299	21.537	4.88E-10	0.00013419	NA	NA	NA	NA	No	NA
ENSG00000142102	ATHL1	11	289138	296107	ENSG00000205038	PKHD1L1	8	110374706	110542559	0.01712	4.4168	4.90E-10	0.00013458	0.0014132	0.65027	0.52215	1	Yes	Yes
ENSG00000154760	SLFN13	17	33762115	33775856	ENSG00000167992	VWCE	11	61025762	61062896	0.013706	6.0967	5.24E-10	0.00014365	0.033337	2.6124	0.0019775	1	Yes	No
ENSG00000172123	SLFN12	17	33738079	33760302	ENSG00000007968	E2F2	1	23832922	23857712	0.015445	5.0429	5.30E-10	0.0001453	0.018564	2.8846	0.0086334	1	No	NA
ENSG00000168374	ARF4	3	57557090	57583947	ENSG00000019102	VSIG2	11	124618002	124621476	0.010703	10.455	5.37E-10	0.00014694	0.037344	7.1067	1.56E-06	0.00284856	No	NA
ENSG00000139330	KERA	12	91444268	91451760	ENSG00000178199	ZC3H12D	6	149768794	149806197	0.0087899	21.438	5.38E-10	0.00014697	0.00065936	0.30318	0.73854	1	No	NA
ENSG00000163947	ARHGEF3	3	56761446	57113357	ENSG00000088053	GP6	19	55525073	55549632	0.01227	7.4982	5.45E-10	0.00014877	0.075855	9.3676	1.74E-12	3.29E-09	No	NA
ENSG00000088826	SMOX	20	4101627	4168394	ENSG00000072274	TFRC	3	195754054	195809060	0.012752	6.9292	5.49E-10	0.00014959	0.0053106	0.54101	0.84509	1	No	NA
ENSG00000163946	FAM208A	3	56658507	56717265	ENSG00000095303	PTGS1	9	125132824	125157982	0.012265	7.4955	5.50E-10	0.0001498	0.026523	3.5575	0.00088016	1	Yes	No
ENSG00000172660	TAF15	17	34136459	34174246	ENSG00000090674	MCOLN1	19	7587512	7595387	0.011229	9.1442	5.64E-10	0.00015327	0.010045	1.5475	0.15957	1	Yes	No
ENSG00000163947	ARHGEF3	3	56761446	57113357	ENSG00000164116	GUCY1A3	4	156587863	156653501	0.01225	7.4863	5.69E-10	0.00015438	0.024152	2.8246	0.0042349	1	No	NA
ENSG00000108733	PEX12	17	33901814	33905882	ENSG00000164849	GPR146	7	1084212	1098897	0.010671	10.423	5.78E-10	0.00015669	0.0082286	1.52	0.18089	1	Yes	No
ENSG00000167136	ENDOG	9	131580753	131581464	ENSG00000101425	BPI	20	36888551	36965907	0.010084	12.308	5.86E-10	0.00015867	0.016134	2.1411	0.037211	1	Yes	Yes
ENSG00000168374	ARF4	3	57557090	57583947	ENSG00000113140	SPARC	5	151040657	151066726	0.010661	10.413	5.92E-10	0.00015994	0.018904	3.53	0.0036265	1	Yes	No
ENSG00000154768	C17orf50	17	34087916	34092098	ENSG00000100614	PPM1A	14	60712470	60765805	0.011194	9.1152	6.11E-10	0.00016492	0.011602	1.5327	0.15247	1	Yes	No
ENSG00000108733	PEX12	17	33901814	33905882	ENSG00000130227	XPO7	8	21777180	21864096	0.010644	10.397	6.15E-10	0.0001659	0.0051594	0.9501	0.44766	1	Yes	Yes
ENSG00000249242	TMEM150C	4	83405743	83483510	ENSG00000100448	CTSG	14	25042762	25045466	0.013625	6.06	6.22E-10	0.00016763	0.0051067	0.42463	0.94559	1	Yes	No
ENSG00000172660	TAF15	17	34136459	34174246	ENSG00000134779	TPGS2	18	34361762	34403834	0.011171	9.0963	6.43E-10	0.00017305	NA	NA	NA	NA	Yes	No
ENSG00000008838	MED24	17	38175350	38217468	ENSG00000162551	ALPL	1	21835858	21904905	0.010029	12.241	6.66E-10	0.00017881	0.01362	3.1655	0.013469	1	Yes	No
ENSG00000163947	ARHGEF3	3	56761446	57113357	ENSG00000154146	NRGN	11	124609829	124615878	0.012165	7.4336	6.86E-10	0.00018415	0.04708	5.6385	5.28E-07	0.00097046	No	NA
ENSG00000185379	RAD51D	17	33426811	33447063	ENSG00000204420	C6orf25	6	31691121	31694491	0.0093758	15.251	7.10E-10	0.0001902	0.0018035	0.55288	0.6463	1	No	NA
ENSG00000159692	CTBP1	4	1205236	1243741	ENSG00000132740	IGHMBP2	11	68671310	68708067	0.011646	8.1306	7.14E-10	0.00019084	0.014141	1.8728	0.070847	1	No	NA
ENSG00000205045	SLFN12L	17	33800708	33864880	ENSG00000165406	8-Mar	10	45950035	46090354	0.011646	8.1307	7.14E-10	0.00019084	0.0065209	0.85703	0.54023	1	No	NA
ENSG00000129282	MRM1	17	34958001	34965407	ENSG00000158856	DMTN	8	21906506	21940038	0.01112	9.0545	7.22E-10	0.00019269	NA	NA	NA	NA	No	NA
ENSG00000172716	SLFN11	17	33677324	33700720	ENSG00000029534	ANK1	8	41510739	41754280	0.014	5.7093	7.25E-10	0.00019319	0.02571	1.9989	0.021614	1	Yes	No
ENSG00000099849	RASSF7	11	560404	564021	ENSG00000122786	CALD1	7	134429003	134655479	0.011636	8.1231	7.31E-10	0.00019468	0.010468	1.3813	0.20964	1	Yes	No
ENSG00000129270	MMP28	17	34083268	34122711	ENSG00000158856	DMTN	8	21906506	21940038	0.011112	9.0474	7.36E-10	0.00019575	NA	NA	NA	NA	No	NA
ENSG00000172660	TAF15	17	34136459	34174246	ENSG00000196914	ARHGEF12	11	120207787	120360645	0.011109	9.045	7.41E-10	0.00019652	0.027478	4.3089	0.00026995	0.46836325	No	NA
ENSG00000131165	CHMP1A	16	89710839	89720331	ENSG00000175718	RBMXL3	X	114423963	114427431	0.013085	6.3998	7.41E-10	0.00019652	NA	NA	NA	NA	No	NA
ENSG00000141150	RASL10B	17	34058668	34070540	ENSG00000011105	TSPAN9	12	3186521	3395730	0.012121	7.4062	7.57E-10	0.00019993	0.051227	7.0499	3.26E-08	6.04E-05	Yes	No
ENSG00000142082	SIRT3	11	215458	236431	ENSG00000172572	PDE3A	12	20522179	20837315	0.0093494	15.207	7.56E-10	0.00019993	NA	NA	NA	NA	Yes	Yes
ENSG00000154768	C17orf50	17	34087916	34092098	ENSG00000079308	TNS1	2	218664512	218867718	0.0111	9.0375	7.57E-10	0.00019993	0.017117	2.2739	0.026777	1	No	NA
ENSG00000177963	RIC8A	11	207511	215113	ENSG00000185532	PRKG1	10	52750945	54058110	0.010542	10.297	7.77E-10	0.00020501	NA	NA	NA	NA	No	NA
ENSG00000154760	SLFN13	17	33762115	33775856	ENSG00000103257	SLC7A5	16	87863629	87903094	0.013517	6.0114	7.83E-10	0.00020617	0.02084	1.6122	0.08271	1	Yes	No
ENSG00000114268	PFKFB4	3	48555117	48598605	ENSG00000134595	SOX3	X	139585152	139587225	0.011604	8.101	7.84E-10	0.00020636	NA	NA	NA	NA	No	NA
ENSG00000166582	CENPV	17	16247911	16256813	ENSG00000169429	IL8	4	74606223	74609433	0.011594	8.094	8.02E-10	0.00021073	0.022771	21.438	4.18E-06	0.00759088	No	NA
ENSG00000132139	GAS2L2	17	34071530	34079897	ENSG00000167992	VWCE	11	61025762	61062896	0.012091	7.3878	8.08E-10	0.00021211	0.010185	1.1743	0.31147	1	Yes	No
ENSG00000114388	NPRL2	3	50384919	50387453	ENSG00000120937	NPPB	1	11917521	11918988	0.0086178	21.015	8.18E-10	0.00021444	NA	NA	NA	NA	No	NA
ENSG00000006125	AP2B1	17	33913918	34053436	ENSG00000137198	GMPR	6	16238811	16295780	0.011578	8.0823	8.32E-10	0.00021783	0.0036579	0.55988	0.76244	1	No	NA
ENSG00000164053	ATRIP	3	48488114	48506061	ENSG00000100228	RAB36	22	23487513	23506537	0.012561	6.8238	8.33E-10	0.00021783	0.0052891	0.60683	0.77268	1	No	NA
ENSG00000205045	SLFN12L	17	33800708	33864880	ENSG00000174175	SELP	1	169558087	169599431	0.011574	8.0797	8.39E-10	0.00021907	0.018241	2.4261	0.018237	1	Yes	Yes
ENSG00000114388	NPRL2	3	50384919	50387453	ENSG00000204290	BTNL2	6	32361740	32374905	0.008602	20.976	8.50E-10	0.00022173	NA	NA	NA	NA	No	NA
ENSG00000154760	SLFN13	17	33762115	33775856	ENSG00000165406	8-Mar	10	45950035	46090354	0.013475	5.9928	8.54E-10	0.00022224	0.0095108	0.72736	0.72543	1	No	NA
ENSG00000187492	CDHR4	3	49828165	49837268	ENSG00000120937	NPPB	1	11917521	11918988	0.0086005	20.972	8.54E-10	0.00022224	NA	NA	NA	NA	No	NA
ENSG00000205045	SLFN12L	17	33800708	33864880	ENSG00000068971	PPP2R5B	11	64685025	64700718	0.011561	8.0701	8.65E-10	0.0002247	0.0063165	0.83	0.5624	1	No	NA
ENSG00000007312	CD79B	17	62006100	62009714	ENSG00000171611	PTCRA	6	42883727	42893573	0.011039	8.9871	8.70E-10	0.00022558	0.011547	1.7815	0.099815	1	No	NA
ENSG00000175283	DOLK	9	131707809	131709898	ENSG00000128294	TPST2	22	26921458	26992681	0.01254	6.8122	8.72E-10	0.000226	0.010917	1.1185	0.34647	1	No	NA
ENSG00000168374	ARF4	3	57557090	57583947	ENSG00000164116	GUCY1A3	4	156587863	156653501	0.010484	10.239	8.88E-10	0.0002299	0.027668	5.213	0.00010009	0.17605831	Yes	No
ENSG00000154760	SLFN13	17	33762115	33775856	ENSG00000120885	CLU	8	27454434	27472548	0.013456	5.9839	8.91E-10	0.0002303	0.0099461	0.76098	0.69126	1	Yes	No
ENSG00000189144	ZNF573	19	38226734	38307940	ENSG00000111291	GPRC5D	12	13093709	13105081	0.011529	8.0481	9.27E-10	0.00023937	0.015004	2.7906	0.016452	1	No	NA
ENSG00000108733	PEX12	17	33901814	33905882	ENSG00000136732	GYPC	2	127413509	127454246	0.010462	10.218	9.35E-10	0.00024097	0.011196	2.0743	0.066402	1	Yes	No
ENSG00000132436	FIGNL1	7	50511831	50518088	ENSG00000152689	RASGRP3	2	33661391	33789817	0.0077115	37.583	9.46E-10	0.00024354	0.001581	1.4568	0.22775	1	No	NA
ENSG00000138468	SENP7	3	101043049	101232085	ENSG00000196247	ZNF107	7	64126511	64171404	0.0092549	15.052	9.48E-10	0.00024371	0.023541	7.3771	6.91E-05	0.1218924	No	NA
ENSG00000185614	FAM212A	3	49840687	49842463	ENSG00000204290	BTNL2	6	32361740	32374905	0.0092456	15.037	9.69E-10	0.00024883	NA	NA	NA	NA	No	NA
ENSG00000006125	AP2B1	17	33913918	34053436	ENSG00000019102	VSIG2	11	124618002	124621476	0.011494	8.0234	1.00E-09	0.00025722	0.015351	2.3775	0.027628	1	Yes	Yes
ENSG00000141150	RASL10B	17	34058668	34070540	ENSG00000113140	SPARC	5	151040657	151066726	0.011986	7.3226	1.02E-09	0.00026125	0.028882	3.8833	0.0003532	0.6096232	Yes	No

ENSG00000137760	ALKBH8	11	107373452	107436472	ENSG00000197409	HIST1H3D	6	26197068	26197497	0.012461	6.7688	1.04E-09	0.00026489	0.020419	2.1122	0.026174	1	No	NA
ENSG00000163946	FAM208A	3	56658507	56717265	ENSG00000140479	PCSK6	15	101840818	102065405	0.011972	7.3139	1.05E-09	0.00026878	0.043582	5.9498	8.69E-07	0.00159288	Yes	No
ENSG00000140993	TIGD7	16	3353759	3355150	ENSG00000159882	ZNF230	19	44507100	44518078	0.011469	8.0055	1.06E-09	0.00027087	0.080606	11.448	5.57E-14	1.06E-10	No	NA
ENSG00000141150	RASL10B	17	34058668	34070540	ENSG00000158828	PINK1	1	20959948	20978004	0.011966	7.3104	1.07E-09	0.00027121	0.015351	2.0356	0.048108	1	Yes	No
ENSG00000100307	CBX7	22	39516172	39548655	ENSG00000157502	MUM1L1	X	105412298	105452949	0.0092059	14.972	1.07E-09	0.00027121	NA	NA	NA	NA	No	NA
ENSG00000168374	ARF4	3	57557090	57583947	ENSG00000166333	ILK	11	6624961	6631511	0.010394	10.151	1.09E-09	0.00027792	0.011751	2.1784	0.054536	1	Yes	No
ENSG00000142082	SIRT3	11	215458	236431	ENSG00000095303	PTGS1	9	125132824	125157982	0.0091945	14.953	1.09E-09	0.00027803	0.002458	0.56489	0.6882	1	Yes	Yes
ENSG00000188511	C22orf34	22	49808176	50051190	ENSG00000149575	SCN2B	11	118032666	118047388	0.010384	10.14	1.12E-09	0.00028413	0.0024048	0.36761	0.89963	1	No	NA
ENSG00000157315	TMED6	16	69377151	69377543	ENSG00000255833	TIFAB	5	134787899	134788089	0.010383	10.139	1.12E-09	0.00028419	0.0072705	1.3417	0.24433	1	Yes	Yes
ENSG00000182179	UBA7	3	49842640	49851379	ENSG00000182156	ENPP7	17	77704681	77716021	0.0098025	11.961	1.13E-09	0.00028669	NA	NA	NA	NA	No	NA
ENSG00000006194	ZNF263	16	3313743	3341460	ENSG00000159882	ZNF230	19	44507100	44518078	0.011437	7.9828	1.14E-09	0.00028808	0.081019	8.0315	1.69E-12	3.20E-09	No	NA
ENSG00000118495	PLAGL1	6	144261437	144385735	ENSG00000135378	PRRG4	11	32851489	32879669	0.010375	10.132	1.14E-09	0.00028826	0.012662	2.3493	0.039274	1	No	NA
ENSG00000145022	TCTA	3	49452253	49453908	ENSG00000131059	BPIFA3	20	31805116	31815564	0.0097856	11.94	1.18E-09	0.00029716	NA	NA	NA	NA	No	NA
ENSG00000174885	NLRP6	11	278365	285359	ENSG00000061918	GUCY1B3	4	156680144	156728743	0.011417	7.969	1.19E-09	0.00029991	0.023814	2.472	0.0086751	1	Yes	Yes
ENSG00000172123	SLFN12	17	33738079	33760302	ENSG00000167671	UBXN6	19	4446046	4457819	0.015045	4.9102	1.20E-09	0.00030116	0.014571	2.2549	0.036292	1	Yes	No
ENSG00000239388	ASB14	3	57310556	57326710	ENSG00000172572	PDE3A	12	20522179	20837315	0.010348	10.105	1.22E-09	0.00030513	NA	NA	NA	NA	Yes	No
ENSG00000205045	SLFN12L	17	33800708	33864880	ENSG00000187017	ESPN	1	6484848	6521040	0.011408	7.9624	1.22E-09	0.00030513	0.0094159	1.2411	0.27718	1	Yes	Yes
ENSG00000090316	MAEA	4	1283639	1333925	ENSG00000132740	IGHMBP2	11	68671310	68708067	0.009146	14.873	1.23E-09	0.00030794	0.003184	0.58517	0.7114	1	No	NA
ENSG00000073598	FNDC8	17	33448598	33454436	ENSG00000070182	SPTB	14	65216372	65346601	0.0091449	14.871	1.23E-09	0.00030839	0.00797	2.4584	0.061547	1	No	NA
ENSG00000168427	KLHL30	2	239047363	239061588	ENSG00000088854	C20orf194	20	3229951	3388272	0.011402	7.9578	1.23E-09	0.00030839	0.0058664	0.67345	0.71526	1	No	NA
ENSG00000205045	SLFN12L	17	33800708	33864880	ENSG00000095321	CRAT	9	131857089	131873083	0.011397	7.9544	1.25E-09	0.00031139	0.011117	1.4679	0.17505	1	Yes	Yes
ENSG00000177951	BET1L	11	167784	207428	ENSG00000144677	CTDSPL	3	37903451	38025960	0.011889	7.2631	1.26E-09	0.00031425	0.026987	2.2944	0.0090618	1	Yes	No
ENSG00000172660	TAF15	17	34136459	34174246	ENSG00000039068	CDH1	16	68771128	68869451	0.010865	8.8442	1.29E-09	0.00032091	0.0032523	0.4976	0.81045	1	No	NA
ENSG00000141150	RASL10B	17	34058668	34070540	ENSG00000173068	BNC2	9	16409501	16870841	0.011875	7.2543	1.30E-09	0.00032336	0.029772	4.0067	0.00024914	0.43300532	No	NA
ENSG00000205045	SLFN12L	17	33800708	33864880	ENSG00000104892	KLC3	19	45836692	45852860	0.011376	7.9399	1.31E-09	0.00032402	0.012181	1.6101	0.12881	1	No	NA
ENSG00000176095	IP6K1	3	49761727	49823975	ENSG00000120937	NPPB	1	11917521	11918988	0.0097424	11.887	1.30E-09	0.00032402	NA	NA	NA	NA	No	NA
ENSG00000141150	RASL10B	17	34058668	34070540	ENSG00000238243	OR2W3	1	248058859	248060449	0.011872	7.2525	1.31E-09	0.00032432	0.0057717	0.758	0.62279	1	Yes	No
ENSG00000163406	SLC15A2	3	121612936	121662949	ENSG00000158022	TRIM63	1	26377795	26394927	0.010305	10.062	1.34E-09	0.00033252	NA	NA	NA	NA	No	NA
ENSG00000085999	RAD54L	1	46713360	46743652	ENSG00000204188	GGNBP1	6	33551515	33556803	0.012331	6.6972	1.37E-09	0.0003396	NA	NA	NA	NA	No	NA
ENSG00000142082	SIRT3	11	215458	236431	ENSG00000174175	SELP	1	169558087	169599431	0.0090958	14.791	1.39E-09	0.00034213	0.0042142	0.9702	0.42293	1	Yes	Yes
ENSG00000153814	JAZF1	7	27886122	28220362	ENSG00000176358	TAC4	17	47915671	47925379	0.011843	7.2345	1.40E-09	0.00034411	0.0068413	0.69803	0.71112	1	No	NA
ENSG00000172716	SLFN11	17	33677324	33700720	ENSG00000167671	UBXN6	19	4446046	4457819	0.013686	5.5793	1.40E-09	0.00034525	0.022533	1.7462	0.052915	1	Yes	No
ENSG00000100242	SUN2	22	39134592	39162338	ENSG00000157502	MUM1L1	X	105412298	105452949	0.0090854	14.774	1.42E-09	0.00034941	NA	NA	NA	NA	No	NA
ENSG00000205045	SLFN12L	17	33800708	33864880	ENSG00000149260	CAPN5	11	76777979	76837201	0.011332	7.909	1.44E-09	0.00035381	0.011317	1.4946	0.16543	1	Yes	Yes
ENSG00000205045	SLFN12L	17	33800708	33864880	ENSG00000137193	PIM1	6	37137979	37143202	0.011326	7.9048	1.46E-09	0.0003581	0.018166	2.4158	0.018719	1	Yes	Yes
ENSG00000100211	CBY1	22	39052645	39069859	ENSG00000163064	EN1	2	119599747	119605254	0.0075342	36.712	1.47E-09	0.00036115	NA	NA	NA	NA	No	NA
ENSG00000130724	CHMP2A	19	59062933	59066491	ENSG00000180537	RNF182	6	13924677	13980533	0.010803	8.793	1.48E-09	0.00036329	0.0048583	0.89438	0.48414	1	No	NA
ENSG00000172660	TAF15	17	34136459	34174246	ENSG00000123908	AGO2	8	141541264	141645718	0.010792	8.7841	1.52E-09	0.00037184	NA	NA	NA	NA	Yes	No
ENSG00000205045	SLFN12L	17	33800708	33864880	ENSG00000177666	PNPLA2	11	818902	825573	0.011302	7.8875	1.54E-09	0.00037645	0.0055846	0.73329	0.64378	1	Yes	Yes
ENSG00000205045	SLFN12L	17	33800708	33864880	ENSG00000161914	ZNF653	19	11594242	11615999	0.011291	7.8796	1.58E-09	0.00038542	0.010244	1.3514	0.22279	1	No	NA
ENSG00000006125	AP2B1	17	33913918	34053436	ENSG00000136732	GYPC	2	127413509	127454246	0.011289	7.8787	1.58E-09	0.00038608	0.012952	2.001	0.062999	1	Yes	Yes
ENSG00000074803	SLC12A1	15	48498498	48596275	ENSG00000144649	FAM198A	3	43020759	43101703	0.0075013	36.551	1.60E-09	0.00038833	0.0052805	4.8839	0.027354	1	No	NA
ENSG00000074803	SLC12A1	15	48498498	48596275	ENSG00000182983	ZNF662	3	42947223	42960825	0.0075013	36.551	1.60E-09	0.00038833	9.30E-05	0.08557	0.76995	1	No	NA
ENSG00000074803	SLC12A1	15	48498498	48596275	ENSG00000240747	KRBOX1	3	42977834	42984284	0.0075013	36.551	1.60E-09	0.00038833	NA	NA	NA	NA	No	NA
ENSG00000100412	ACO2	22	41865129	41921352	ENSG00000175556	LONRF3	X	118108581	118156888	0.01022	9.9783	1.63E-09	0.00039616	0.011942	2.7709	0.026239	1	Yes	No
ENSG00000142082	SIRT3	11	215458	236431	ENSG00000173210	ABLIM3	5	148521046	148640105	0.0090242	14.673	1.64E-09	0.00039673	0.0075026	1.733	0.14054	1	Yes	Yes
ENSG00000127415	IDUA	4	980785	998316	ENSG00000266967	AARSD1	17	41114192	41116515	0.012713	6.2157	1.64E-09	0.00039673	0.0085281	0.98164	0.44873	1	No	NA
ENSG00000127415	IDUA	4	980785	998316	ENSG00000267060	PTGES3L	17	41120105	41121203	0.012713	6.2157	1.64E-09	0.00039673	NA	NA	NA	NA	No	NA
ENSG00000127415	IDUA	4	980785	998316	ENSG00000108825	PTGES3L-AAI	17	41131922	41132020	0.012713	6.2157	1.64E-09	0.00039673	NA	NA	NA	NA	No	NA
ENSG00000163946	FAM208A	3	56658507	56717265	ENSG00000119862	LGALSL	2	64681103	64688515	0.011761	7.184	1.67E-09	0.00040261	NA	NA	NA	NA	Yes	No
ENSG00000132139	GAS2L2	17	34071530	34079897	ENSG00000196961	AP2A1	19	50270225	50309510	0.011757	7.1812	1.69E-09	0.00040616	0.0086585	0.99678	0.43683	1	Yes	No
ENSG00000168374	ARF4	3	57557090	57583947	ENSG00000101335	MYL9	20	35169887	35178228	0.01018	9.9387	1.79E-09	0.00043091	0.024425	4.5867	0.00038721	0.66793725	Yes	No
ENSG00000154760	SLFN13	17	33762115	33775856	ENSG00000137193	PIM1	6	37137979	37143202	0.013124	5.8345	1.80E-09	0.00043214	0.01616	1.2442	0.24747	1	Yes	No
ENSG00000154760	SLFN13	17	33762115	33775856	ENSG00000118046	STK11	19	1189406	1226662	0.013121	5.8331	1.81E-09	0.00043444	0.010337	0.79119	0.66001	1	Yes	No
ENSG00000112592	TBP	6	170863390	170881958	ENSG00000183313	OR52L1	11	6007122	6008215	0.010174	9.9328	1.82E-09	0.00043535	NA	NA	NA	NA	No	NA
ENSG00000122863	CHST3	10	73724123	73773322	ENSG00000162931	TRIM17	1	228595641	228604562	0.011226	7.8338	1.83E-09	0.00043706	0.0055579	0.72976	0.64679	1	No	NA
ENSG00000159314	ARHGAP27	17	43471275	43511787	ENSG00000183150	GPR19	12	12813825	12849141	0.010706	8.7132	1.85E-09	0.00044191	0.0039803	0.60943	0.72295	1	No	NA
ENSG00000188452	CERKL	2	182401403	182521843	ENSG00000096006	CRISP3	6	49695097	49712150	0.0095919	11.702	1.86E-09	0.00044321	0.014399	3.3492	0.009837	1	Yes	Yes
ENSG00000167930	ITFG3	16	284545	316124	ENSG00000167384	ZNF180	19	44979854	45004576	0.010161	9.9206	1.87E-09	0.00044577	0.0035282	0.35879	0.95421	1	No	NA
ENSG00000092871	RFFL	17	33341759	33416338	ENSG00000029534	ANK1	8	41510739	41754280	0.010697	8.7057	1.89E-09	0.00044952	0.0066701	0.76634	0.63264	1	Yes	No
ENSG00000004534	RBM6	3	49977440	50114683	ENSG00000147488	ST18	8	53023399	53322505	0.00827	20.16	1.91E-09	0.00045473	NA	NA	NA	NA	No	NA
ENSG00000177963	RIC8A	11	207511	215113	ENSG00000204420	C6orf25	6	31691121	31694491	0.010143	9.9026	1.95E-09	0.00046324	0.0078442	0.9023	0.51377	1	Yes	Yes
ENSG00000108733	PEX12	17	33901814	33905882	ENSG00000143774	GUK1	1	228327663	228336685	0.010141	9.9011	1.96E-09	0.00046434	0.0074923	1.383	0.22818	1	Yes	No
ENSG00000108733	PEX12	17	33901814	33905882	ENSG00000008441	NFIX	19	13106422	13209610	0.010134	9.894	1.99E-09	0.00047154	0.011024	2.0421	0.07054	1	No	NA
ENSG00000172660	TAF15	17	34136459	34174246	ENSG00000047597	XK	X	37545012	37591383	0.010665	8.6796	2.03E-09	0.00048014	0.0064808	0.99477	0.42744	1	No	NA
ENSG00000158955	WNT9B	17	44910567	44964096	ENSG00000183150	GPR19	12	12813825	12849141	0.011171	7.7949	2.06E-09	0.00048848	0.0026938	0.35268	0.92918	1	No	NA
ENSG00000174885	NLRP6	11	278365	285359	ENSG00000124491	F13A1	6	6144318	6321246	0.01117	7.7941	2.07E-09	0.00048868	0.02017	2.0859	0.028303	1	Yes	Yes

ENSG00000172037	LAMB2	3	49158547	49170551	ENSG00000103023	PRSS54	16	58318309	58328951	0.0082374	20.079	2.07E-09	0.00048868	NA	NA	NA	NA	No	NA
ENSG00000132139	GAS2L2	17	34071530	34079897	ENSG00000144567	FAM134A	2	220042939	220050201	0.011662	7.1225	2.08E-09	0.00048993	0.015569	1.8049	0.072535	1	Yes	No
ENSG00000006125	AP2B1	17	33913918	34053436	ENSG00000168785	TSPAN5	4	99391518	99579780	0.011161	7.7877	2.11E-09	0.0004974	0.0044611	0.68337	0.66314	1	No	NA
ENSG00000100241	SBF1	22	50885184	50913454	ENSG00000258986	TMEM179	14	104941015	105071984	0.0082216	20.041	2.15E-09	0.0005058	NA	NA	NA	NA	No	NA
ENSG00000167914	GSDMA	17	38119226	38134019	ENSG00000110080	ST3GAL4	11	126225535	126310239	0.00822	20.037	2.16E-09	0.00050663	0.01015	4.7117	0.0092075	1	Yes	Yes
ENSG00000167914	GSDMA	17	38119226	38134019	ENSG00000254607	RP11-115C1(	11	126522747	126551999	0.00822	20.037	2.16E-09	0.00050663	NA	NA	NA	NA	No	NA
ENSG00000141150	RASL10B	17	34058668	34070540	ENSG00000120885	CLU	8	27454434	27472548	0.011644	7.1114	2.16E-09	0.00050673	0.024678	3.3038	0.0017736	1	Yes	No
ENSG00000168374	ARF4	3	57557090	57583947	ENSG00000148498	PARD3	10	34398488	35104253	0.010084	9.844	2.23E-09	0.00052321	0.0020973	0.38504	0.85924	1	No	NA
ENSG00000177951	BET1L	11	167784	207428	ENSG00000124491	F13A1	6	6144318	6321246	0.011623	7.0984	2.26E-09	0.00052958	0.024563	2.0832	0.019209	1	Yes	No
ENSG00000108588	CCDC47	17	61822610	61853711	ENSG00000171611	PTCRA	6	42883727	42893573	0.0095061	11.596	2.27E-09	0.00052991	0.0052663	1.2137	0.30333	1	No	NA
ENSG00000115232	ITGA4	2	182321934	182400914	ENSG00000096006	CRISP3	6	49695097	49712150	0.011127	7.7638	2.28E-09	0.00053141	0.019639	2.6156	0.011192	1	Yes	Yes
ENSG00000108733	PEX12	17	33901814	33905882	ENSG00000256269	HMBS	11	118955576	118964259	0.010072	9.833	2.29E-09	0.0005343	0.014248	2.6479	0.02187	1	Yes	No
ENSG00000132139	GAS2L2	17	34071530	34079897	ENSG00000090674	MCOLN1	19	7587512	7595387	0.011606	7.0882	2.35E-09	0.00054662	0.0012935	0.14781	0.99678	1	Yes	No
ENSG00000095319	NUP188	9	131709978	131768647	ENSG00000164821	DEFA4	8	6793344	6795860	0.0081804	19.939	2.38E-09	0.00055295	0.0035392	1.632	0.1961	1	No	NA
ENSG00000154760	SLFN13	17	33762115	33775856	ENSG00000113140	SPARC	5	151040657	151066726	0.012991	5.7743	2.38E-09	0.00055407	0.015452	1.1888	0.28607	1	Yes	No
ENSG00000154760	SLFN13	17	33762115	33775856	ENSG00000017483	SLC38A5	X	48316920	48328644	0.012989	5.7737	2.39E-09	0.000555	0.038829	3.0602	0.00030123	0.52203159	Yes	No
ENSG00000108733	PEX12	17	33901814	33905882	ENSG00000104904	OAZ1	19	2271369	2273487	0.010049	9.8102	2.42E-09	0.00056028	0.0025101	0.46102	0.80539	1	Yes	No
ENSG00000132139	GAS2L2	17	34071530	34079897	ENSG00000166169	POLL	10	103338639	103346957	0.011581	7.0725	2.48E-09	0.00057461	0.015244	1.7666	0.079939	1	Yes	No
ENSG00000183396	TMEM89	3	48658192	48659288	ENSG00000134595	SOX3	X	139585152	139587225	0.008849	14.386	2.50E-09	0.00057738	NA	NA	NA	NA	No	NA
ENSG00000138468	SENP7	3	101043049	101232085	ENSG00000176293	ZNF135	19	58570607	58593375	0.0088479	14.384	2.50E-09	0.00057833	0.080304	26.719	1.43E-16	2.71E-13	No	NA
ENSG00000172123	SLFN12	17	33738079	33760302	ENSG00000164068	RNF123	3	49728563	49753910	0.014677	4.7885	2.52E-09	0.00058101	0.021745	3.3898	0.0025936	1	Yes	No
ENSG00000172660	TAF15	17	34136459	34174246	ENSG00000184792	OSBP2	22	31089769	31303811	0.010568	8.6003	2.52E-09	0.00058181	0.025887	4.0527	0.00051111	0.87450921	Yes	No
ENSG00000167914	GSDMA	17	38119226	38134019	ENSG00000135842	FAM129A	1	184759858	184943682	0.0081521	19.87	2.55E-09	0.00058637	0.0084699	3.9252	0.020071	1	Yes	Yes
ENSG00000189180	ZNF33A	10	38299578	38354016	ENSG00000141194	OR4D1	17	56232494	56233517	0.010556	8.5899	2.59E-09	0.00059639	NA	NA	NA	NA	No	NA
ENSG00000006125	AP2B1	17	33913918	34053436	ENSG00000197993	KEL	7	142638201	142659768	0.01106	7.7171	2.64E-09	0.00060622	0.020397	3.1753	0.0043402	1	Yes	Yes
ENSG00000108733	PEX12	17	33901814	33905882	ENSG00000118816	CCNI	4	77968311	77997158	0.0099991	9.7607	2.71E-09	0.00062245	0.0027042	0.49675	0.77884	1	Yes	No
ENSG00000164828	SUN1	7	855528	914557	ENSG00000129514	FOXA1	14	38059189	38069245	0.0099931	9.7549	2.75E-09	0.00063026	NA	NA	NA	NA	No	NA
ENSG00000132139	GAS2L2	17	34071530	34079897	ENSG00000103257	SLC7A5	16	87863629	87903094	0.011529	7.0402	2.78E-09	0.00063714	0.012327	1.4244	0.18204	1	No	NA
ENSG00000157500	APPL1	3	57261765	57301820	ENSG00000205038	PKHD1L1	8	110374706	110542559	0.0094154	11.484	2.80E-09	0.00064121	0.00085651	0.26232	0.85257	1	Yes	No
ENSG00000154760	SLFN13	17	33762115	33775856	ENSG00000095303	PTGS1	9	125132824	125157982	0.01291	5.7379	2.83E-09	0.00064627	0.034444	2.7022	0.0013662	1	Yes	No
ENSG00000108733	PEX12	17	33901814	33905882	ENSG00000133808	MICALCL	11	12297627	12380691	0.0099757	9.7376	2.86E-09	0.0006524	0.0034141	0.6276	0.67875	1	Yes	No
ENSG00000108733	PEX12	17	33901814	33905882	ENSG00000133816	MICAL2	11	12115543	12285332	0.0099757	9.7376	2.86E-09	0.0006524	0.0045444	0.83633	0.52395	1	Yes	No
ENSG00000205045	SLFN12L	17	33800708	33864880	ENSG00000100614	PPM1A	14	60712470	60765805	0.011011	7.6823	2.94E-09	0.00067051	0.012845	1.699	0.10566	1	Yes	Yes
ENSG00000141748	ARL5C	17	37313147	37323318	ENSG00000162551	ALPL	1	21835858	21904905	0.0080871	19.71	2.98E-09	0.00067875	0.001053	0.48436	0.61625	1	Yes	No
ENSG00000139330	KERA	12	91444268	91451760	ENSG00000162613	FUBP1	1	78409740	78444794	0.0080824	19.698	3.02E-09	0.00068572	0.0022927	1.0559	0.3483	1	No	NA
ENSG00000172123	SLFN12	17	33738079	33760302	ENSG00000029534	ANK1	8	41510739	41754280	0.014587	4.7585	3.02E-09	0.0006863	0.0054583	0.83696	0.54138	1	Yes	No
ENSG00000139330	KERA	12	91444268	91451760	ENSG00000079785	DDX1	2	15731302	15771235	0.008079	19.69	3.04E-09	0.00068994	0.0024839	1.1442	0.31893	1	No	NA
ENSG00000163946	FAM208A	3	56658507	56717265	ENSG00000158457	TSPAN33	7	128784712	128808637	0.011485	7.013	3.06E-09	0.00069389	0.0086431	1.1384	0.33651	1	Yes	No
ENSG00000163947	ARHGEF3	3	56761446	57113357	ENSG00000138798	EGF	4	110834040	110933422	0.011474	7.0062	3.14E-09	0.00071013	0.0202	2.3528	0.016595	1	No	NA
ENSG00000162614	NEXN	1	78354198	78409580	ENSG00000183844	FAM3B	21	42676139	42729358	0.012399	6.0603	3.21E-09	0.00072628	0.025889	2.4211	0.007601	1	No	NA
ENSG00000108733	PEX12	17	33901814	33905882	ENSG00000168785	TSPAN5	4	99391518	99579780	0.0099197	9.6825	3.25E-09	0.00073446	0.0050429	0.92855	0.46156	1	No	NA
ENSG00000132139	GAS2L2	17	34071530	34079897	ENSG00000007968	E2F2	1	23832922	23857712	0.011457	6.9957	3.26E-09	0.00073455	0.0090185	1.0386	0.40488	1	No	NA
ENSG00000123411	IKZF4	12	56401443	56432219	ENSG00000105085	MED26	19	16698215	16739873	0.010961	7.6473	3.29E-09	0.0007405	0.0066049	0.86814	0.5312	1	Yes	No
ENSG00000172346	CSDC2	22	41956767	41972670	ENSG00000175556	LONRF3	X	118108581	118156888	0.0093315	11.381	3.41E-09	0.00076736	0.01797	3.3524	0.0052408	1	Yes	Yes
ENSG00000141150	RASL10B	17	34058668	34070540	ENSG00000164068	RNF123	3	49728563	49753910	0.011435	6.9822	3.42E-09	0.00076823	0.014223	1.8839	0.069025	1	Yes	No
ENSG00000141150	RASL10B	17	34058668	34070540	ENSG00000140564	FURIN	15	91411822	91426688	0.011434	6.9814	3.43E-09	0.00076954	0.0029409	0.38513	0.91136	1	Yes	No
ENSG00000154768	C17orf50	17	34087916	34092098	ENSG00000166947	EPB42	15	43398423	43513481	0.010428	8.4848	3.46E-09	0.00077587	0.0078262	1.0299	0.40826	1	Yes	No
ENSG00000172660	TAF15	17	34136459	34174246	ENSG00000130300	PLVAP	19	17462257	17488159	0.010418	8.4764	3.54E-09	0.00079312	0.020136	3.1338	0.0047918	1	Yes	No
ENSG00000184886	PIGW	17	34892943	34895159	ENSG00000167992	VWCE	11	61025762	61062896	0.0093134	11.359	3.56E-09	0.00079608	0.016359	3.0467	0.0098086	1	Yes	No
ENSG00000073598	FNDC8	17	33448598	33454436	ENSG00000079308	TNS1	2	218664512	218867718	0.0086944	14.132	3.61E-09	0.00080648	0.0080894	2.4956	0.058586	1	No	NA
ENSG00000249242	TMEM150C	4	83405743	83483510	ENSG00000164821	DEFA4	8	6793344	6795860	0.012793	5.6854	3.62E-09	0.00080709	0.0037425	0.31077	0.98376	1	Yes	No
ENSG00000172716	SLFN11	17	33677324	33700720	ENSG00000007968	E2F2	1	23832922	23857712	0.013231	5.3914	3.62E-09	0.00080709	0.0099294	0.75969	0.69259	1	No	NA
ENSG00000008838	MED24	17	38175350	38217468	ENSG00000146592	CREB5	7	28338940	28865511	0.0093022	11.345	3.65E-09	0.00081281	0.0047248	1.0883	0.361	1	Yes	No
ENSG00000099849	RASSF7	11	560404	564021	ENSG00000172572	PDE3A	12	20522179	20837315	0.010915	7.6142	3.65E-09	0.00081281	NA	NA	NA	NA	Yes	No
ENSG00000139330	KERA	12	91444268	91451760	ENSG00000119787	ATL2	2	38522022	38604427	0.0080001	19.496	3.69E-09	0.00081969	0.0062716	2.9	0.055528	1	No	NA
ENSG00000075407	ZNF37A	10	38383264	38412276	ENSG00000169393	ELSPBP1	19	48497908	48528410	0.010399	8.4612	3.69E-09	0.00081969	NA	NA	NA	NA	No	NA
ENSG00000119408	NEK6	9	127019885	127115586	ENSG00000134198	TSPAN2	1	115590632	115632121	0.0092914	11.332	3.75E-09	0.00083073	0.033898	6.4281	7.00E-06	0.012649	No	NA
ENSG00000006125	AP2B1	17	33913918	34053436	ENSG00000130830	MPP1	X	154006959	154049282	0.010896	7.6008	3.81E-09	0.00084331	0.0086596	1.3321	0.23991	1	No	NA
ENSG00000198917	C9orf114	9	131586026	131592100	ENSG00000101425	BPI	20	36888551	36965907	0.0092831	11.321	3.82E-09	0.00084527	0.0093119	2.8762	0.03522	1	Yes	No
ENSG00000177951	BET1L	11	167784	207428	ENSG00000061918	GUCY1B3	4	156680144	156728743	0.01138	6.9483	3.85E-09	0.00085221	0.021613	1.8275	0.045599	1	Yes	Yes
ENSG00000129270	MMP28	17	34083268	34122711	ENSG00000070182	SPTB	14	65216372	65346601	0.01038	8.4449	3.86E-09	0.00085268	0.010891	1.4377	0.18654	1	No	NA
ENSG00000006125	AP2B1	17	33913918	34053436	ENSG00000049323	LTBP1	2	33172039	33624576	0.010885	7.5932	3.90E-09	0.00085986	0.0037409	0.57263	0.75236	1	No	NA
ENSG00000164077	MON1A	3	49946302	49967606	ENSG00000182156	ENPP7	17	77704681	77716021	0.0086608	14.077	3.91E-09	0.00086158	NA	NA	NA	NA	No	NA
ENSG00000092871	RFFL	17	33341759	33416338	ENSG00000164068	RNF123	3	49728563	49753910	0.010372	8.4391	3.92E-09	0.00086358	0.010437	1.2036	0.29346	1	Yes	No
ENSG00000241399	CD302	2	160637946	160654753	ENSG00000146426	TIAM2	6	155181872	155575707	0.010875	7.5861	3.99E-09	0.00087664	0.024231	3.7871	0.00098505	1	No	NA
ENSG00000127418	FGFRL1	4	1003724	1020685	ENSG00000132740	IGHMBP2	11	68671310	68708067	0.010871	7.5838	4.02E-09	0.0008821	0.0082715	0.84517	0.57449	1	No	NA
ENSG00000100413	POLR3H	22	41926691	41940610	ENSG00000175556	LONRF3	X	118108581	118156888	0.0098253	9.5894	4.04E-09	0.00088666	0.020297	3.1594	0.0045084	1	No	NA

ENSG00000172660	TAF15	17	34136459	34174246	ENSG00000118816	CCNI	4	77968311	77997158	0.010351	8.4213	4.12E-09	0.00090287	0.016579	2.571	0.017836	1	Yes	No
ENSG00000092871	RFFL	17	33341759	33416338	ENSG00000167992	VWCE	11	61025762	61062896	0.010346	8.4173	4.16E-09	0.00091185	0.016253	1.8855	0.058957	1	Yes	No
ENSG00000154760	SLFN13	17	33762115	33775856	ENSG00000007350	TKTL1	X	153524024	153558700	0.012719	5.652	4.23E-09	0.00092459	0.015217	1.1705	0.29974	1	No	NA
ENSG00000108733	PEX12	17	33901814	33905882	ENSG00000153162	BMP6	6	7727030	7880334	0.0098051	9.5695	4.23E-09	0.00092474	0.0045688	0.84085	0.52079	1	Yes	No
ENSG00000110934	BIN2	12	51674822	51718452	ENSG00000181904	C5orf24	5	134181370	134182553	0.010338	8.411	4.24E-09	0.00092487	0.0093157	1.434	0.19845	1	No	NA
ENSG00000163947	ARHGEF3	3	56761446	57113357	ENSG00000005249	PRKAR2B	7	106685094	106802256	0.011326	6.9149	4.34E-09	0.00094588	0.05362	6.4661	3.30E-08	6.11E-05	No	NA
ENSG00000132139	GAS2L2	17	34071530	34079897	ENSG00000095321	CRAT	9	131857089	131873083	0.011321	6.9118	4.39E-09	0.00095542	0.008369	0.96317	0.46349	1	Yes	No
ENSG00000088826	SMOX	20	4101627	4168394	ENSG00000112146	FBXO9	6	52916789	52965671	0.011792	6.401	4.41E-09	0.00095926	0.010807	1.1071	0.35474	1	No	NA
ENSG00000174885	NLRP6	11	278365	285359	ENSG00000172572	PDE3A	12	20522179	20837315	0.010825	7.5511	4.45E-09	0.00096741	NA	NA	NA	NA	Yes	Yes
ENSG00000139330	KERA	12	91444268	91451760	ENSG00000167842	MIS12	17	5390247	5394134	0.007919	19.297	4.49E-09	0.0009757	0.0047935	2.2132	0.10993	1	No	NA
ENSG00000142082	SIRT3	11	215458	236431	ENSG00000120885	CLU	8	27454434	27472548	0.0086008	13.979	4.51E-09	0.00097732	0.010469	2.4254	0.046543	1	Yes	Yes
ENSG00000141150	RASL10B	17	34058668	34070540	ENSG00000198876	DCAF12	9	34086385	34127397	0.011308	6.9039	4.51E-09	0.00097732	0.013218	1.749	0.094334	1	No	NA
ENSG00000142082	SIRT3	11	215458	236431	ENSG00000061918	GUCY1B3	4	156680144	156728743	0.0085977	13.974	4.54E-09	0.00098231	0.011294	2.6187	0.033822	1	Yes	Yes
ENSG00000141002	TCF25	16	89940014	89977792	ENSG00000162772	ATF3	1	212738676	212794119	0.0097746	9.5394	4.54E-09	0.00098231	0.0076719	1.179	0.31513	1	No	NA
ENSG00000088826	SMOX	20	4101627	4168394	ENSG00000076770	MBNL3	X	131503345	131623996	0.011776	6.3926	4.56E-09	0.00098412	0.022296	2.3108	0.014337	1	No	NA
ENSG00000115486	GGCX	2	85774743	85788670	ENSG00000141096	DPEP3	16	68009566	68014732	0.0092026	11.222	4.61E-09	0.00099441	0.031163	7.3738	7.59E-06	0.01368477	Yes	Yes
ENSG00000108733	PEX12	17	33901814	33905882	ENSG00000100325	ASCC2	22	30184597	30234265	0.0097659	9.5308	4.63E-09	0.00099798	0.0015054	0.27621	0.92624	1	No	NA
ENSG00000168374	ARF4	3	57557090	57583947	ENSG00000173210	ABLIM3	5	148521046	148640105	0.0097641	9.5291	4.65E-09	0.00100104	0.031242	5.9081	2.19E-05	0.0392229	Yes	No
ENSG00000066117	SMARCD1	12	50478755	50494495	ENSG00000181904	C5orf24	5	134181370	134182553	0.0091936	11.211	4.71E-09	0.00101218	0.0055355	1.0197	0.40465	1	No	NA
ENSG00000172057	ORMDL3	17	38077294	38083854	ENSG00000151726	ACSL1	4	185676749	185747972	0.0097454	9.5106	4.85E-09	0.0010427	0.011653	1.7981	0.096462	1	Yes	Yes
ENSG00000228008	CTD-2330K9.3	3	49943495	49954370	ENSG00000147488	ST18	8	53023399	53322505	0.0085669	13.923	4.89E-09	0.00104923	NA	NA	NA	NA	No	NA
ENSG00000168374	ARF4	3	57557090	57583947	ENSG00000198478	SH3BGRL2	6	80341000	80413372	0.0097401	9.5054	4.91E-09	0.00105328	0.025325	4.7602	0.00026662	0.46285232	Yes	No
ENSG00000141150	RASL10B	17	34058668	34070540	ENSG00000144677	CTDSPL	3	37903451	38025960	0.011254	6.8703	5.08E-09	0.00108805	0.041166	5.6058	2.41E-06	0.00439825	Yes	No
ENSG00000138379	MSTN	2	190920423	190927455	ENSG00000124635	HIST1H2BJ	6	27093676	27100541	0.0097248	9.4904	5.09E-09	0.00108845	0.0083705	1.5464	0.17282	1	No	NA
ENSG00000167914	GSDMA	17	38119226	38134019	ENSG00000182541	LIMK2	22	31608260	31676066	0.0078649	19.164	5.13E-09	0.00109576	0.014788	6.8972	0.0010637	1	Yes	Yes
ENSG00000206013	IFITM5	11	298200	299526	ENSG00000165621	OXGR1	13	97637973	97646984	0.011713	6.3576	5.23E-09	0.00111611	NA	NA	NA	NA	No	NA
ENSG00000173402	DAG1	3	49506146	49573048	ENSG00000182156	ENPP7	17	77704681	77716021	0.0091478	11.155	5.24E-09	0.00111713	NA	NA	NA	NA	No	NA
ENSG00000172057	ORMDL3	17	38077294	38083854	ENSG00000170525	PFKFB3	10	6186881	6277495	0.0097064	9.4722	5.31E-09	0.00113071	0.014755	2.2839	0.034036	1	Yes	Yes
ENSG00000108733	PEX12	17	33901814	33905882	ENSG00000125818	PSMF1	20	1093906	1149022	0.0097046	9.4704	5.33E-09	0.00113422	0.0015193	0.27876	0.92486	1	No	NA
ENSG00000008838	MED24	17	38175350	38217468	ENSG00000196663	TECPR2	14	102829300	102968818	0.0091388	11.144	5.35E-09	0.00113738	0.013454	3.1265	0.014393	1	Yes	No
ENSG00000111540	RAB5B	12	56367697	56388490	ENSG00000105085	MED26	19	16698215	16739873	0.010229	8.321	5.42E-09	0.00115175	0.011433	1.5101	0.16003	1	Yes	Yes
ENSG00000242173	ARHGDIG	16	321881	333003	ENSG00000167384	ZNF180	19	44979854	45004576	0.011218	6.8486	5.49E-09	0.00116405	0.011044	0.92386	0.51641	1	No	NA
ENSG00000172660	TAF15	17	34136459	34174246	ENSG00000113140	SPARC	5	151040657	151066726	0.01022	8.3137	5.53E-09	0.0011727	0.011229	1.7319	0.11047	1	Yes	No
ENSG00000180376	CCDC66	3	56591189	56653929	ENSG00000185052	SLC24A3	20	19193290	19703581	0.0096614	9.4279	5.88E-09	0.00124565	0.01182	2.1913	0.053209	1	Yes	No
ENSG00000132139	GAS2L2	17	34071530	34079897	ENSG00000204463	BAG6	6	31606805	31620170	0.011184	6.8275	5.91E-09	0.00125033	0.01735	2.015	0.041924	1	Yes	No
ENSG00000163946	FAM208A	3	56658507	56717265	ENSG00000183785	TUBA8	22	18593097	18593634	0.011181	6.8253	5.96E-09	0.00125899	0.034877	4.7185	3.23E-05	0.0575586	Yes	No
ENSG00000138315	OIT3	10	74653339	74692791	ENSG00000162931	TRIM17	1	228595641	228604562	0.0078023	19.01	5.97E-09	0.00126065	0.00011563	0.053139	0.94825	1	No	NA
ENSG00000148341	SH3GLB2	9	131771385	131790582	ENSG00000128294	TPST2	22	26921458	26992681	0.012551	5.5766	6.01E-09	0.00126701	0.0034361	0.52581	0.78899	1	No	NA
ENSG00000131771	PPP1R1B	17	37782993	37792879	ENSG00000146592	CREB5	7	28338940	28865511	0.011646	6.3211	6.03E-09	0.00127051	0.0033377	0.33935	0.96184	1	Yes	No
ENSG00000114378	HYAL1	3	50337320	50349812	ENSG00000182156	ENPP7	17	77704681	77716021	0.009083	11.075	6.09E-09	0.00128212	NA	NA	NA	NA	No	NA
ENSG00000172716	SLFN11	17	33677324	33700720	ENSG00000138867	GUCD1	22	24936406	24951284	0.012971	5.2838	6.22E-09	0.00130733	NA	NA	NA	NA	Yes	No
ENSG00000101493	ZNF516	18	74069644	74207146	ENSG00000136834	OR1J1	9	125239220	125240235	0.011623	6.3086	6.34E-09	0.00133033	NA	NA	NA	NA	No	NA
ENSG00000154175	ABI3BP	3	100468000	100712359	ENSG00000196247	ZNF107	7	64126511	64171404	0.012079	5.9019	6.35E-09	0.00133252	0.02694	2.5222	0.0053677	1	No	NA
ENSG00000167074	TEF	22	41763337	41795330	ENSG00000175556	LONRF3	X	118108581	118156888	0.0096254	9.3923	6.39E-09	0.00133912	0.01621	3.0186	0.010387	1	No	NA
ENSG00000172426	RSPH9	6	43612783	43634257	ENSG00000170448	NFXL1	4	47849257	47913011	0.010151	8.2568	6.47E-09	0.0013537	0.010913	2.5295	0.039208	1	No	NA
ENSG00000239388	ASB14	3	57310556	57326710	ENSG00000259207	ITGB3	17	45387505	45389182	0.0096146	9.3818	6.55E-09	0.00136963	0.015797	2.9405	0.012167	1	Yes	No
ENSG00000205045	SLFN12L	17	33800708	33864880	ENSG00000197993	KEL	7	142638201	142659768	0.01065	7.4275	6.56E-09	0.00137	0.021452	2.8625	0.0058465	1	Yes	Yes
ENSG00000095319	NUP188	9	131709978	131768647	ENSG00000169397	RNASE3	14	21359558	21360507	0.0077639	18.916	6.56E-09	0.00137	0.006848	3.1684	0.042532	1	No	NA
ENSG00000177096	FAM109B	22	42470255	42475445	ENSG00000076344	RGS11	16	320522	325940	0.010642	7.4217	6.69E-09	0.00139398	0.0049914	0.65501	0.71033	1	No	NA
ENSG00000141150	RASL10B	17	34058668	34070540	ENSG00000118046	STK11	19	1189406	1226662	0.011116	6.7852	6.86E-09	0.00142872	0.0029437	0.38549	0.91115	1	Yes	No
ENSG00000100138	NHP2L1	22	42069934	42086508	ENSG00000175556	LONRF3	X	118108581	118156888	0.010124	8.2349	6.87E-09	0.00142872	0.01983	3.0852	0.0053784	1	Yes	Yes
ENSG00000177951	BET1L	11	167784	207428	ENSG00000174175	SELP	1	169558087	169599431	0.011113	6.7834	6.91E-09	0.00143636	0.011728	0.98174	0.46114	1	Yes	Yes
ENSG00000177951	BET1L	11	167784	207428	ENSG00000185245	GP1BA	17	4835596	4838325	0.011097	6.7734	7.16E-09	0.00148619	0.019298	1.6279	0.085849	1	Yes	No
ENSG00000177951	BET1L	11	167784	207428	ENSG00000138798	EGF	4	110834040	110933422	0.011082	6.7645	7.39E-09	0.00153254	0.016157	1.3585	0.18732	1	Yes	Yes
ENSG00000076003	MCM6	2	136597196	136633996	ENSG00000132386	SERPINF1	17	1665253	1680868	0.011546	6.2663	7.48E-09	0.00154952	0.025829	2.6867	0.00437	1	No	NA
ENSG00000172660	TAF15	17	34136459	34174246	ENSG00000197993	KEL	7	142638201	142659768	0.01008	8.1991	7.57E-09	0.00156787	0.016831	2.6107	0.016286	1	Yes	No
ENSG00000154760	SLFN13	17	33762115	33775856	ENSG00000104522	TSTA3	8	144694788	144700218	0.01244	5.5264	7.59E-09	0.00157003	0.0032616	0.24788	0.99564	1	No	NA
ENSG00000006125	AP2B1	17	33913918	34053436	ENSG00000110693	SOX6	11	15987995	16761138	0.01058	7.3781	7.67E-09	0.00158431	0.0055758	0.85508	0.52759	1	No	NA
ENSG00000144229	THSD7B	2	137523115	138435287	ENSG00000204385	SLC44A4	6	31830969	31846823	0.012433	5.5233	7.70E-09	0.0015901	NA	NA	NA	NA	No	NA
ENSG00000186075	ZPBP2	17	38024417	38034149	ENSG00000131873	CHSY1	15	101715928	101792137	0.0068721	33.463	7.72E-09	0.00159141	0.0027382	2.5261	0.11232	1	Yes	No
ENSG00000100147	CCDC134	22	42196683	42222303	ENSG00000169313	P2RY12	3	151055168	151102600	0.01007	8.1902	7.76E-09	0.00159848	0.0081758	1.0763	0.37644	1	No	NA
ENSG00000108733	PEX12	17	33901814	33905882	ENSG00000047597	XK	X	37545012	37591383	0.0095391	9.3074	7.79E-09	0.0016019	0.0035937	0.66073	0.65336	1	No	NA
ENSG00000108733	PEX12	17	33901814	33905882	ENSG00000143416	SELENBP1	1	151336778	151345209	0.0095349	9.3032	7.86E-09	0.00161583	0.0011279	0.20687	0.95966	1	No	NA
ENSG00000155099	TMEM55A	8	92006024	92053292	ENSG00000148484	RSU1	10	16632610	16859527	0.0083665	13.595	7.88E-09	0.00161842	0.0071504	2.2038	0.086132	1	No	NA
ENSG00000163933	RFT1	3	53122499	53164478	ENSG00000134809	TIMM10	11	57295936	57298276	0.0076853	18.723	7.94E-09	0.0016294	0.0025048	1.1538	0.31588	1	No	NA
ENSG00000182504	CEP97	3	101442769	101489406	ENSG00000196247	ZNF107	7	64126511	64171404	0.010056	8.1787	8.01E-09	0.00164136	0.010456	1.6114	0.14071	1	No	NA
ENSG00000183508	FAM46C	1	118148556	118170994	ENSG00000109272	PF4V1	4	74718906	74719872	0.011039	6.7376	8.12E-09	0.00166328	0.024278	2.8397	0.0040491	1	No	NA

ENSG00000006125	AP2B1	17	33913918	34053436	ENSG00000008441	NFIX	19	13106422	13209610	0.010551	7.3581	8.16E-09	0.00166976	0.01407	2.1763	0.043131	1	No	NA
ENSG00000251247	ZNF345	19	37341263	37403931	ENSG00000111291	GPRC5D	12	13093709	13105081	0.0095145	9.2831	8.24E-09	0.00168144	0.030595	5.7819	2.89E-05	0.0515865	No	NA
ENSG00000075043	KCNQ2	20	62037542	62103993	ENSG00000049247	UTS2	1	7906752	7913572	0.0095148	9.2834	8.23E-09	0.00168144	0.0074113	0.97493	0.4481	1	No	NA
ENSG00000164050	PLXNB1	3	48445261	48471594	ENSG00000134595	SOX3	X	139585152	139587225	0.0089525	10.915	8.26E-09	0.00168448	NA	NA	NA	NA	No	NA
ENSG00000108306	FBXL20	17	37415384	37558776	ENSG00000162551	ALPL	1	21835858	21904905	0.0095112	9.2799	8.30E-09	0.00169073	0.0078733	1.4538	0.20254	1	Yes	No
ENSG00000167914	GSDMA	17	38119226	38134019	ENSG00000151726	ACSL1	4	185676749	185747972	0.0076655	18.675	8.33E-09	0.0016963	0.01203	5.595	0.0038442	1	Yes	Yes
ENSG00000172660	TAF15	17	34136459	34174246	ENSG00000130159	ECSIT	19	11618272	11639989	0.010032	8.1597	8.44E-09	0.00171365	0.020708	3.2247	0.0038571	1	Yes	No
ENSG00000007384	RHBDF1	16	108058	126354	ENSG00000167384	ZNF180	19	44979854	45004576	0.0089437	10.904	8.43E-09	0.00171365	0.0049926	0.91924	0.46764	1	No	NA
ENSG00000177951	BET1L	11	167784	207428	ENSG00000102362	SYTL4	X	99929488	99987110	0.01101	6.7201	8.64E-09	0.00175015	0.01185	0.99203	0.4516	1	Yes	Yes
ENSG00000134287	ARF3	12	49297286	49351334	ENSG00000213949	ITGA1	5	52120147	52255040	0.010022	8.151	8.64E-09	0.00175015	0.0058696	0.77093	0.61184	1	No	NA
ENSG00000134287	ARF3	12	49297286	49351334	ENSG00000152684	PELO	5	52095719	52099880	0.010022	8.151	8.64E-09	0.00175015	0.0030037	0.39338	0.90653	1	No	NA
ENSG00000139330	KERA	12	91444268	91451760	ENSG00000047315	POLR2B	4	57851426	57896565	0.0076468	18.629	8.72E-09	0.00176501	1.64E-05	0.0075225	0.99251	1	No	NA
ENSG00000188511	C22orf34	22	49808176	50051190	ENSG00000154319	FAM167A	8	11278972	11332224	0.0094877	9.2567	8.76E-09	0.00176998	0.00595	0.91281	0.48479	1	No	NA
ENSG00000141736	ERBB2	17	37844949	37884915	ENSG00000134755	DSC2	18	28645940	28682378	0.010518	7.3345	8.79E-09	0.00177063	0.0036676	0.37302	0.94811	1	Yes	No
ENSG00000154760	SLFN13	17	33762115	33775856	ENSG00000102145	GATA1	X	48644962	48652718	0.01237	5.495	8.79E-09	0.00177063	0.028591	2.2295	0.0090774	1	Yes	No
ENSG00000144224	UBXN4	2	136499189	136542625	ENSG00000132386	SERPINF1	17	1665253	1680868	0.010015	8.1453	8.77E-09	0.00177063	0.018611	2.892	0.0084834	1	No	NA
ENSG00000139330	KERA	12	91444268	91451760	ENSG00000152795	HNRNPDL	4	83343717	83351294	0.0076435	18.62	8.80E-09	0.00177063	NA	NA	NA	NA	No	NA
ENSG00000108733	PEX12	17	33901814	33905882	ENSG00000204420	C6orf25	6	31691121	31694491	0.0094853	9.2543	8.81E-09	0.00177113	0.022589	4.234	0.00082305	1	No	NA
ENSG00000172660	TAF15	17	34136459	34174246	ENSG00000104522	TSTA3	8	144694788	144700218	0.01001	8.1411	8.88E-09	0.0017836	0.014532	2.2488	0.036783	1	No	NA
ENSG00000154760	SLFN13	17	33762115	33775856	ENSG00000068971	PPP2R5B	11	64685025	64700718	0.012358	5.4896	9.01E-09	0.00180855	0.010373	0.79399	0.65709	1	Yes	No
ENSG00000099849	RASSF7	11	560404	564021	ENSG00000151693	ASAP2	2	9346894	9541525	0.010504	7.325	9.06E-09	0.00181646	0.0044269	0.5806	0.77211	1	Yes	No
ENSG00000184348	HIST1H2AK	6	27805658	27806117	ENSG00000049247	UTS2	1	7906752	7913572	0.0089125	10.865	9.07E-09	0.00181667	0.0048234	1.1111	0.34989	1	No	NA
ENSG00000142102	ATHL1	11	289138	296107	ENSG00000151693	ASAP2	2	9346894	9541525	0.015612	4.0216	9.20E-09	0.00184196	0.00066157	0.30419	0.73779	1	Yes	Yes
ENSG00000132139	GAS2L2	17	34071530	34079897	ENSG00000183597	TANGO2	22	20004537	20053449	0.010979	6.7009	9.25E-09	0.00184896	NA	NA	NA	NA	No	NA
ENSG00000114354	TFG	3	100428205	100467810	ENSG00000179909	ZNF154	19	58208735	58220579	0.011442	6.2088	9.36E-09	0.00187023	0.026068	3.0547	0.0021253	1	No	NA
ENSG00000177666	PNPLA2	11	818902	825573	ENSG00000049323	LTBP1	2	33172039	33624576	0.0094573	9.2268	9.39E-09	0.00187351	0.021009	3.7275	0.0034394	1	Yes	No
ENSG00000154760	SLFN13	17	33762115	33775856	ENSG00000011105	TSPAN9	12	3186521	3395730	0.012332	5.4779	9.51E-09	0.00189565	0.022762	1.7644	0.049727	1	Yes	No
ENSG00000177963	RIC8A	11	207511	215113	ENSG00000174175	SELP	1	169558087	169599431	0.0094504	9.22	9.54E-09	0.00189565	0.010383	1.1974	0.29721	1	Yes	Yes
ENSG00000242612	DECR2	16	455513	462487	ENSG00000167384	ZNF180	19	44979854	45004576	0.0099785	8.1154	9.52E-09	0.00189565	0.0018426	0.84823	0.42851	1	No	NA
ENSG00000183657	PP13439	3	171509580	171527714	ENSG00000027869	SH2D2A	1	156776035	156786654	0.0094508	9.2203	9.53E-09	0.00189565	0.00050603	0.092752	0.99337	1	No	NA
ENSG00000172660	TAF15	17	34136459	34174246	ENSG00000168785	TSPAN5	4	99391518	99579780	0.0099767	8.1139	9.56E-09	0.00189907	0.0070487	1.0826	0.37095	1	No	NA
ENSG00000213171	LINGO4	1	151772740	151775193	ENSG00000119862	LGALSL	2	64681103	64688515	0.0099738	8.1115	9.63E-09	0.00190963	NA	NA	NA	NA	No	NA
ENSG00000100221	JOSD1	22	39081548	39097561	ENSG00000157502	MUM1L1	X	105412298	105452949	0.0099733	8.1111	9.64E-09	0.00191009	NA	NA	NA	NA	No	NA
ENSG00000164588	HCN1	5	45259349	45696253	ENSG00000240386	LCE1F	1	152748848	152749445	0.0088851	10.832	9.67E-09	0.00191408	NA	NA	NA	NA	No	NA
ENSG00000008838	MED24	17	38175350	38217468	ENSG00000163421	PROK2	3	71820807	71834357	0.0088793	10.825	9.80E-09	0.00193833	0.011764	2.729	0.028142	1	Yes	Yes
ENSG00000132139	GAS2L2	17	34071530	34079897	ENSG00000103148	NPRL3	16	138697	188859	0.010951	6.6835	9.83E-09	0.00194328	0.010462	1.2066	0.29166	1	Yes	No
ENSG00000155592	ZKSCAN2	16	25247322	25269252	ENSG00000183844	FAM3B	21	42676139	42729358	0.010944	6.679	9.99E-09	0.0019724	0.0075258	0.8654	0.54532	1	No	NA
ENSG00000108604	SMARCD2	17	61909444	61920425	ENSG00000171611	PTCRA	6	42883727	42893573	0.0088673	10.81	1.01E-08	0.00198731	0.0046841	1.0789	0.36567	1	No	NA
ENSG00000092871	RFFL	17	33341759	33416338	ENSG00000130300	PLVAP	19	17462257	17488159	0.0099455	8.0882	1.03E-08	0.00202146	0.0027958	0.31996	0.95866	1	Yes	No
ENSG00000154760	SLFN13	17	33762115	33775856	ENSG00000141084	RANBP10	16	67757005	67840555	0.012294	5.4607	1.03E-08	0.00202897	0.017141	1.3211	0.20046	1	Yes	No
ENSG00000188076	SCGB1C1	11	193080	194573	ENSG00000138722	MMRN1	4	90800683	90875780	0.0067515	32.872	1.04E-08	0.00205318	0.0057822	5.3506	0.020935	1	Yes	Yes
ENSG00000141150	RASL10B	17	34058668	34070540	ENSG00000017483	SLC38A5	X	48316920	48328644	0.010923	6.6661	1.05E-08	0.00205416	0.025397	3.4025	0.0013519	1	Yes	No
ENSG00000006125	AP2B1	17	33913918	34053436	ENSG00000182732	RGS6	14	72399156	73030654	0.010439	7.2788	1.05E-08	0.00205573	0.0051463	0.78887	0.57871	1	No	NA
ENSG00000132139	GAS2L2	17	34071530	34079897	ENSG00000160445	ZER1	9	131492065	131534693	0.010914	6.6607	1.07E-08	0.00208984	0.0029908	0.34235	0.9494	1	Yes	No
ENSG00000006125	AP2B1	17	33913918	34053436	ENSG00000130227	XPO7	8	21777180	21864096	0.010423	7.268	1.08E-08	0.00212272	0.0057807	0.88669	0.50393	1	Yes	Yes
ENSG00000006125	AP2B1	17	33913918	34053436	ENSG00000133816	MICAL2	11	12115543	12285332	0.010422	7.2672	1.09E-08	0.00212376	0.010238	1.5775	0.15047	1	Yes	Yes
ENSG00000006125	AP2B1	17	33913918	34053436	ENSG00000133808	MICALCL	11	12297627	12380691	0.010422	7.2672	1.09E-08	0.00212376	0.0078117	1.2007	0.3035	1	Yes	Yes
ENSG00000167528	ZNF641	12	48733791	48745197	ENSG00000161040	FBXL13	7	102453308	102715286	0.01042	7.2655	1.09E-08	0.00213287	0.021619	2.5218	0.01026	1	No	NA
ENSG00000164053	ATRIP	3	48488114	48506061	ENSG00000103023	PRSS54	16	58318309	58328951	0.011369	6.169	1.09E-08	0.00213532	NA	NA	NA	NA	No	NA
ENSG00000158805	ZNF276	16	89786808	89800446	ENSG00000133250	ZNF414	19	8575462	8579044	0.010902	6.653	1.10E-08	0.00213544	0.0085939	0.98928	0.44271	1	No	NA
ENSG00000121481	RNF2	1	185014496	185071740	ENSG00000140287	HDC	15	50534144	50558223	0.0109	6.6522	1.10E-08	0.00213945	0.0060926	0.80039	0.58704	1	No	NA
ENSG00000185958	FAM186A	12	50720013	50790405	ENSG00000181904	C5orf24	5	134181370	134182553	0.0088295	10.763	1.10E-08	0.0021417	0.016242	3.785	0.0046342	1	No	NA
ENSG00000139330	KERA	12	91444268	91451760	ENSG00000169251	NMD3	3	160874759	160971320	0.0075453	18.38	1.12E-08	0.00217194	0.0018554	0.85414	0.42599	1	No	NA
ENSG00000177963	RIC8A	11	207511	215113	ENSG00000173210	ABLIM3	5	148521046	148640105	0.0093801	9.1508	1.12E-08	0.00217571	0.019075	2.2193	0.024082	1	Yes	Yes
ENSG00000108733	PEX12	17	33901814	33905882	ENSG00000159023	EPB41	1	29213603	29442315	0.0093789	9.1496	1.12E-08	0.00217947	0.0039292	0.72267	0.60649	1	No	NA
ENSG00000132436	FIGNL1	7	50511831	50518088	ENSG00000013583	HEBP1	12	13127798	13153207	0.006721	32.723	1.13E-08	0.00218517	0.0050137	4.6359	0.03157	1	No	NA
ENSG00000092871	RFFL	17	33341759	33416338	ENSG00000102145	GATA1	X	48644962	48652718	0.0098949	8.0467	1.15E-08	0.0022263	0.016274	1.888	0.058578	1	No	NA
ENSG00000172660	TAF15	17	34136459	34174246	ENSG00000068971	PPP2R5B	11	64685025	64700718	0.0098936	8.0456	1.15E-08	0.00223078	0.020256	3.1529	0.004579	1	No	NA
ENSG00000141736	ERBB2	17	37844949	37884915	ENSG00000146592	CREB5	7	28338940	28865511	0.010393	7.2466	1.16E-08	0.00223778	0.0084757	0.86621	0.55519	1	Yes	No
ENSG00000163947	ARHGEF3	3	56761446	57113357	ENSG00000149564	ESAM	11	124623019	124632186	0.010876	6.6373	1.16E-08	0.00223778	0.044722	5.3428	1.41E-06	0.00257607	No	NA
ENSG00000225399	RP11-3B7.1	3	49297518	49298744	ENSG00000182156	ENPP7	17	77704681	77716021	0.0082001	13.322	1.17E-08	0.00225941	NA	NA	NA	NA	No	NA
ENSG00000177666	PNPLA2	11	818902	825573	ENSG00000151693	ASAP2	2	9346894	9541525	0.0093561	9.1272	1.18E-08	0.00228099	0.0039207	0.60026	0.73031	1	Yes	No
ENSG00000114302	PRKAR2A	3	48782030	48885279	ENSG00000120937	NPPB	1	11917521	11918988	0.0081821	13.293	1.22E-08	0.00235416	NA	NA	NA	NA	No	NA
ENSG00000108733	PEX12	17	33901814	33905882	ENSG00000128266	GNAZ	22	23412540	23464889	0.0093398	9.1111	1.23E-08	0.0023609	0.014074	2.6152	0.023334	1	Yes	No
ENSG00000177951	BET1L	11	167784	207428	ENSG00000173210	ABLIM3	5	148521046	148640105	0.010849	6.6205	1.23E-08	0.0023609	0.027892	2.3736	0.0067874	1	Yes	Yes
ENSG00000006125	AP2B1	17	33913918	34053436	ENSG00000180354	C7orf41	7	30174426	30202378	0.010365	7.2271	1.23E-08	0.00236338	0.027393	4.295	0.00027945	0.4845663	Yes	Yes
ENSG00000111846	GCNT2	6	10492456	10629601	ENSG00000145423	SFRP2	4	154701744	154710272	0.0098645	8.0217	1.23E-08	0.00236338	0.006293	0.82689	0.56498	1	No	NA

ENSG00000186075	ZPBP2	17	38024417	38034149	ENSG00000196663	TECPR2	14	102829300	102968818	0.0066843	32.543	1.24E-08	0.00236739	6.36E-05	0.058479	0.80897	1	Yes	No
ENSG00000132139	GAS2L2	17	34071530	34079897	ENSG00000100243	CYB5R3	22	43013846	43045574	0.010846	6.6187	1.24E-08	0.00236739	0.010283	1.1857	0.30439	1	No	NA
ENSG00000186075	ZPBP2	17	38024417	38034149	ENSG00000112053	SLC26A8	6	35911291	35992645	0.0066821	32.532	1.24E-08	0.00237705	0.00048691	0.44818	0.50337	1	No	NA
ENSG00000172123	SLFN12	17	33738079	33760302	ENSG00000184481	FOXO4	X	70316047	70323385	0.013878	4.5242	1.25E-08	0.00239127	0.0083713	1.2874	0.26023	1	Yes	No
ENSG00000170684	ZNF296	19	45574758	45579846	ENSG00000164821	DEFA4	8	6793344	6795860	0.0081712	13.275	1.25E-08	0.00239407	0.0017703	0.40656	0.80401	1	No	NA
ENSG00000186792	HYAL3	3	50331063	50333204	ENSG00000182156	ENPP7	17	77704681	77716021	0.011758	5.7432	1.25E-08	0.00239407	NA	NA	NA	NA	No	NA
ENSG00000114378	HYAL1	3	50337320	50349812	ENSG00000204290	BTNL2	6	32361740	32374905	0.0087658	10.685	1.28E-08	0.0024334	NA	NA	NA	NA	No	NA
ENSG00000163947	ARHGEF3	3	56761446	57113357	ENSG00000166333	ILK	11	6624961	6631511	0.01083	6.609	1.28E-08	0.00243552	0.0096042	1.1067	0.35598	1	No	NA
ENSG00000139330	KERA	12	91444268	91451760	ENSG00000164338	UTP15	5	72861566	72877794	0.0074791	18.217	1.31E-08	0.00249777	0.0032327	1.4902	0.22586	1	No	NA
ENSG00000112619	PRPH2	6	42664340	42690312	ENSG00000182389	CACNB4	2	152689290	152955593	0.0074763	18.21	1.32E-08	0.00251237	0.0035458	1.6351	0.1955	1	Yes	No
ENSG00000158792	SPATA2L	16	89767338	89768113	ENSG00000162772	ATF3	1	212738676	212794119	0.0098291	7.9927	1.33E-08	0.00253037	0.0012415	0.18956	0.97978	1	No	NA
ENSG00000068796	KIF2A	5	61601989	61682210	ENSG00000144820	GPR128	3	100328433	100414323	0.010805	6.5934	1.35E-08	0.0025637	NA	NA	NA	NA	No	NA
ENSG00000177963	RIC8A	11	207511	215113	ENSG00000120885	CLU	8	27454434	27472548	0.0092919	9.0639	1.37E-08	0.00259773	0.016764	1.9458	0.050366	1	Yes	Yes
ENSG00000184886	PIGW	17	34892943	34895159	ENSG00000167671	UBXN6	19	4446046	4457819	0.0087334	10.645	1.38E-08	0.00260707	0.012148	2.2528	0.047301	1	Yes	No
ENSG00000162522	KIAA1522	1	33207486	33240571	ENSG00000205364	MT1M	16	56667252	56667898	0.010314	7.1906	1.38E-08	0.00261299	NA	NA	NA	NA	No	NA
ENSG00000185379	RAD51D	17	33426811	33447063	ENSG00000115649	CNPPD1	2	220036619	220042035	0.0081203	13.192	1.42E-08	0.00267638	NA	NA	NA	NA	No	NA
ENSG00000183695	MRGPRX2	11	19076002	19082228	ENSG00000170448	NFXL1	4	47849257	47913011	0.0092637	9.0361	1.46E-08	0.00276042	0.010105	2.3401	0.053509	1	No	NA
ENSG00000172716	SLFN11	17	33677324	33700720	ENSG00000118046	STK11	19	1189406	1226662	0.012555	5.1122	1.47E-08	0.00277128	0.011226	0.86001	0.58806	1	Yes	Yes
ENSG00000132139	GAS2L2	17	34071530	34079897	ENSG00000068971	PPP2R5B	11	64685025	64700718	0.010763	6.5675	1.48E-08	0.00279116	0.0087835	1.0113	0.42558	1	No	NA
ENSG00000141150	RASL10B	17	34058668	34070540	ENSG00000198959	TGM2	20	36756863	36794980	0.010762	6.567	1.48E-08	0.00279331	0.0048603	0.63771	0.72489	1	Yes	No
ENSG00000006125	AP2B1	17	33913918	34053436	ENSG00000163554	SPTA1	1	158580278	158656488	0.010278	7.1656	1.49E-08	0.0028105	0.010001	1.5405	0.16174	1	Yes	Yes
ENSG00000139330	KERA	12	91444268	91451760	ENSG00000055044	NOP58	2	203130439	203168389	0.0074244	18.083	1.50E-08	0.00281958	0.00011881	0.054601	0.94687	1	No	NA
ENSG00000004534	RBM6	3	49977440	50114683	ENSG00000175691	ZNF77	19	2933216	2944969	0.0074225	18.078	1.51E-08	0.00282996	0.0015672	0.72126	0.48642	1	Yes	Yes
ENSG00000006125	AP2B1	17	33913918	34053436	ENSG00000143774	GUK1	1	228327663	228336685	0.010272	7.1611	1.51E-08	0.00284257	0.00511	0.78328	0.5831	1	Yes	Yes
ENSG00000142102	ATHL1	11	289138	296107	ENSG00000144677	CTDSPL	3	37903451	38025960	0.015343	3.9514	1.54E-08	0.0028846	0.00095943	0.44128	0.64335	1	Yes	Yes
ENSG00000163947	ARHGEF3	3	56761446	57113357	ENSG00000102362	SYTL4	X	99929488	99987110	0.010744	6.556	1.54E-08	0.00288814	0.013695	1.5846	0.12518	1	No	NA
ENSG00000139330	KERA	12	91444268	91451760	ENSG00000196511	TPK1	7	144149034	144533488	0.0074125	18.053	1.54E-08	0.00288943	5.57E-05	0.025603	0.97472	1	No	NA
ENSG00000116874	WARS2	1	119573839	119683294	ENSG00000134597	RBMX2	X	129535943	129547317	0.0074112	18.05	1.55E-08	0.00289596	0.0011391	0.52399	0.59233	1	No	NA
ENSG00000152061	RABGAP1L	1	174128548	174964445	ENSG00000175395	ZNF25	10	38238500	38265561	0.011199	6.0759	1.57E-08	0.0029404	0.0078265	0.90025	0.51551	1	No	NA
ENSG00000141150	RASL10B	17	34058668	34070540	ENSG00000184481	FOXO4	X	70316047	70323385	0.010731	6.5481	1.59E-08	0.00295881	0.012574	1.6627	0.11461	1	No	NA
ENSG00000172123	SLFN12	17	33738079	33760302	ENSG00000140564	FURIN	15	91411822	91426688	0.013757	4.4841	1.59E-08	0.00296862	0.0059865	0.91843	0.48072	1	Yes	No
ENSG00000092871	RFFL	17	33341759	33416338	ENSG00000136732	GYPC	2	127413509	127454246	0.0097478	7.9258	1.60E-08	0.00297915	0.0066751	0.76692	0.63212	1	Yes	No
ENSG00000004534	RBM6	3	49977440	50114683	ENSG00000163357	DCST1	1	155006300	155021740	0.007395	18.011	1.61E-08	0.00299896	0.00054379	0.25001	0.77885	1	No	NA
ENSG00000172123	SLFN12	17	33738079	33760302	ENSG00000160445	ZER1	9	131492065	131534693	0.013746	4.4803	1.63E-08	0.0030286	0.0092501	1.4238	0.20231	1	Yes	No
ENSG00000131771	PPP1R1B	17	37782993	37792879	ENSG00000162551	ALPL	1	21835858	21904905	0.011167	6.0584	1.69E-08	0.00313158	0.017863	1.843	0.057129	1	Yes	No
ENSG00000171804	WDR87	19	38375463	38397317	ENSG00000116962	NID1	1	236139130	236228462	0.008646	10.538	1.69E-08	0.0031319	0.014151	3.2907	0.010875	1	No	NA
ENSG00000008838	MED24	17	38175350	38217468	ENSG00000110080	ST3GAL4	11	126225535	126310239	0.0086417	10.532	1.70E-08	0.00315433	0.011946	2.7718	0.026199	1	Yes	Yes
ENSG00000139330	KERA	12	91444268	91451760	ENSG00000162368	CMPK1	1	47799469	47844511	0.0073725	17.955	1.70E-08	0.00315433	0.0006036	0.27752	0.75772	1	No	NA
ENSG00000008838	MED24	17	38175350	38217468	ENSG00000254607	RP11-115C1(	11	126522747	126551999	0.0086417	10.532	1.70E-08	0.00315433	NA	NA	NA	NA	No	NA
ENSG00000143536	CRNN	1	152381719	152386739	ENSG00000138801	PAPSS1	4	108511433	108641608	0.01116	6.0541	1.71E-08	0.00316998	0.0039619	0.40307	0.93379	1	No	NA
ENSG00000173402	DAG1	3	49506146	49573048	ENSG00000131059	BPIFA3	20	31805116	31815564	0.0086362	10.526	1.73E-08	0.00318985	NA	NA	NA	NA	No	NA
ENSG00000092871	RFFL	17	33341759	33416338	ENSG00000137198	GMPR	6	16238811	16295780	0.0097123	7.8967	1.73E-08	0.00319267	0.0076166	0.87592	0.53625	1	No	NA
ENSG00000173421	CCDC36	3	49235861	49295537	ENSG00000120937	NPPB	1	11917521	11918988	0.0086353	10.524	1.73E-08	0.00319267	NA	NA	NA	NA	No	NA
ENSG00000145103	ILDR1	3	121706170	121741051	ENSG00000158022	TRIM63	1	26377795	26394927	0.0073652	17.937	1.73E-08	0.00319267	NA	NA	NA	NA	No	NA
ENSG00000180376	CCDC66	3	56591189	56653929	ENSG00000174175	SELP	1	169558087	169599431	0.0091881	8.9617	1.74E-08	0.00319772	0.010113	1.8716	0.09676	1	Yes	No
ENSG00000088826	SMOX	20	4101627	4168394	ENSG00000100749	VRK1	14	97263641	97398059	0.011152	6.05	1.74E-08	0.0032035	0.0060256	0.6143	0.78558	1	No	NA
ENSG00000008838	MED24	17	38175350	38217468	ENSG00000072952	MRVI1	11	10594638	10715535	0.0086271	10.514	1.76E-08	0.00324014	0.0073738	1.703	0.14717	1	Yes	Yes
ENSG00000172057	ORMDL3	17	38077294	38083854	ENSG00000182541	LIMK2	22	31608260	31676066	0.0091803	8.9541	1.77E-08	0.00324587	0.010395	1.6019	0.14338	1	Yes	Yes
ENSG00000132139	GAS2L2	17	34071530	34079897	ENSG00000029534	ANK1	8	41510739	41754280	0.010681	6.5166	1.77E-08	0.00324884	0.0058022	0.66605	0.72176	1	Yes	No
ENSG00000205045	SLFN12L	17	33800708	33864880	ENSG00000198892	SHISA4	1	201857808	201861434	0.0102	7.1107	1.77E-08	0.00325161	0.0078994	1.0397	0.40146	1	Yes	Yes
ENSG00000132139	GAS2L2	17	34071530	34079897	ENSG00000238243	OR2W3	1	248058859	248060449	0.010677	6.5146	1.78E-08	0.00325792	0.011913	1.376	0.20295	1	Yes	No
ENSG00000132139	GAS2L2	17	34071530	34079897	ENSG00000162722	TRIM58	1	248020501	248031169	0.010677	6.5146	1.78E-08	0.00325792	0.0092978	1.0711	0.38107	1	No	NA
ENSG00000166228	PCBD1	10	72642037	72648541	ENSG00000116299	KIAA1324	1	109656623	109745853	0.0141	4.3092	1.78E-08	0.00325792	0.016604	1.0198	0.43161	1	No	NA
ENSG00000180776	ZDHHC20	13	21950263	22033509	ENSG00000118137	APOA1	11	116706467	116708666	0.011592	5.661	1.78E-08	0.00325792	0.0092539	0.7727	0.66776	1	No	NA
ENSG00000088826	SMOX	20	4101627	4168394	ENSG00000113811	SELK	3	53918437	53926015	0.01114	6.0436	1.79E-08	0.00325867	0.0029986	0.30477	0.97341	1	No	NA
ENSG00000167552	TUBA1A	12	49578579	49581241	ENSG00000196511	TPK1	7	144149034	144533488	0.0096895	7.878	1.82E-08	0.00332214	0.0081727	1.5096	0.18416	1	No	NA
ENSG00000174885	NLRP6	11	278365	285359	ENSG00000173210	ABLIM3	5	148521046	148640105	0.010185	7.1001	1.83E-08	0.00333941	0.020369	2.107	0.026587	1	Yes	Yes
ENSG00000164050	PLXNB1	3	48445261	48471594	ENSG00000100228	RAB36	22	23487513	23506537	0.0086037	10.486	1.86E-08	0.00338549	0.0020816	0.38214	0.86121	1	No	NA
ENSG00000119048	UBE2B	5	133706870	133727683	ENSG00000128512	DOCK4	7	111366166	111846466	0.0091577	8.9318	1.86E-08	0.00338549	0.0016854	0.30928	0.90751	1	No	NA
ENSG00000139330	KERA	12	91444268	91451760	ENSG00000186350	RXRA	9	137208944	137332431	0.0073318	17.855	1.88E-08	0.0034141	0.0021051	0.96932	0.37973	1	No	NA
ENSG00000139330	KERA	12	91444268	91451760	ENSG00000143401	ANP32E	1	150190717	150208504	0.0073286	17.848	1.89E-08	0.00343776	0.0045056	2.0797	0.12556	1	No	NA
ENSG00000100417	PMM1	22	41973812	41985894	ENSG00000175556	LONRF3	X	118108581	118156888	0.0096717	7.8634	1.90E-08	0.00343944	0.015342	2.3761	0.027712	1	Yes	Yes
ENSG00000139330	KERA	12	91444268	91451760	ENSG00000144161	ZC3H8	2	112973254	113012713	0.0073249	17.839	1.91E-08	0.00346231	0.00077307	0.3555	0.70092	1	No	NA
ENSG00000180376	CCDC66	3	56591189	56653929	ENSG00000124491	F13A1	6	6144318	6321246	0.0091449	8.9192	1.92E-08	0.00346957	0.010385	1.9225	0.088117	1	Yes	No
ENSG00000172123	SLFN12	17	33738079	33760302	ENSG00000118046	STK11	19	1189406	1226662	0.013662	4.4528	1.92E-08	0.00347303	0.0063836	0.97976	0.43762	1	Yes	No
ENSG00000141002	TCF25	16	89940014	89977792	ENSG00000133250	ZNF414	19	8575462	8579044	0.0091441	8.9184	1.92E-08	0.00347303	0.016188	2.5093	0.020526	1	No	NA
ENSG00000132694	ARHGEF11	1	156904632	157015162	ENSG00000119862	LGALSL	2	64681103	64688515	0.010161	7.0831	1.93E-08	0.00349146	NA	NA	NA	NA	No	NA

ENSG00000003756	RBM5	3	50126341	50156454	ENSG00000120937	NPPB	1	11917521	11918988	0.0079886	12.976	1.94E-08	0.00349289	NA	NA	NA	NA	No	NA
ENSG00000118640	VAMP8	2	85788685	85809154	ENSG00000141096	DPEP3	16	68009566	68014732	0.0096572	7.8515	1.96E-08	0.00353128	0.050295	6.9149	4.89E-08	9.05E-05	Yes	Yes
ENSG00000205045	SLFN12L	17	33800708	33864880	ENSG00000143774	GUK1	1	228327663	228336685	0.010153	7.0772	1.97E-08	0.00354727	0.0073013	0.96036	0.45902	1	Yes	Yes
ENSG00000108733	PEX12	17	33901814	33905882	ENSG00000110693	SOX6	11	15987995	16761138	0.0091317	8.9062	1.98E-08	0.00355387	0.006315	1.1643	0.3249	1	No	NA
ENSG00000177096	FAM109B	22	42470255	42475445	ENSG00000171246	NPTX1	17	78440948	78451643	0.010151	7.0759	1.98E-08	0.00355578	0.010635	1.4036	0.20024	1	No	NA
ENSG00000154768	C17orf50	17	34087916	34092098	ENSG00000029534	ANK1	8	41510739	41754280	0.0096435	7.8402	2.02E-08	0.00362529	0.014862	1.9698	0.056334	1	Yes	No
ENSG00000173714	WFIKKN2	17	48912011	48919709	ENSG00000174600	CMKLR1	12	108681821	108733118	0.0096435	7.8402	2.02E-08	0.00362529	0.0052352	0.96413	0.43876	1	No	NA
ENSG00000164076	CAMKV	3	49895422	49907655	ENSG00000182156	ENPP7	17	77704681	77716021	0.0079689	12.944	2.03E-08	0.00363754	NA	NA	NA	NA	No	NA
ENSG00000141150	RASL10B	17	34058668	34070540	ENSG00000095303	PTGS1	9	125132824	125157982	0.010613	6.4748	2.05E-08	0.00367467	0.042609	5.8111	1.31E-06	0.0023973	Yes	No
ENSG00000001617	SEMA3F	3	50192478	50226508	ENSG00000131059	BPIFA3	20	31805116	31815564	0.0091134	8.8881	2.06E-08	0.00368613	NA	NA	NA	NA	No	NA
ENSG00000010539	ZNF200	16	3272325	3286221	ENSG00000159882	ZNF230	19	44507100	44518078	0.011073	6.0066	2.06E-08	0.00368882	0.064015	6.2306	2.88E-09	5.39E-06	No	NA
ENSG00000188076	SCGB1C1	11	193080	194573	ENSG00000122786	CALD1	7	134429003	134655479	0.0064789	31.536	2.07E-08	0.00369221	0.00083638	0.77012	0.38041	1	Yes	Yes
ENSG00000174599	TRAM1L1	4	118004718	118006736	ENSG00000149089	APIP	11	34874641	34918402	0.0072924	17.759	2.07E-08	0.00369221	0.0019887	0.91564	0.40062	1	No	NA
ENSG00000115486	GGCX	2	85774743	85788670	ENSG00000169439	SDC2	8	97505579	97624000	0.0085575	10.429	2.07E-08	0.00369363	0.031167	7.375	7.58E-06	0.01367432	Yes	Yes
ENSG00000136141	LRCH1	13	47127303	47327175	ENSG00000196724	ZNF418	19	58433252	58446761	0.010608	6.4721	2.07E-08	0.00369363	0.011793	1.5582	0.14428	1	No	NA
ENSG00000174885	NLRP6	11	278365	285359	ENSG00000120885	CLU	8	27454434	27472548	0.010124	7.0571	2.10E-08	0.00373476	0.019217	1.9854	0.038013	1	Yes	Yes
ENSG00000163947	ARHGEF3	3	56761446	57113357	ENSG00000151693	ASAP2	2	9346894	9541525	0.0106	6.4671	2.11E-08	0.00375055	0.025212	2.9517	0.0028979	1	No	NA
ENSG00000115386	REG1A	2	79347584	79350545	ENSG00000106565	TMEM176B	7	150488373	150498448	0.010116	7.0511	2.14E-08	0.0037984	0.0072674	0.95586	0.46241	1	No	NA
ENSG00000154768	C17orf50	17	34087916	34092098	ENSG00000198876	DCAF12	9	34086385	34127397	0.009618	7.8193	2.14E-08	0.0037984	0.0058034	0.76218	0.61924	1	No	NA
ENSG00000163947	ARHGEF3	3	56761446	57113357	ENSG00000108846	ABCC3	17	48712138	48769613	0.01059	6.4608	2.15E-08	0.00382332	0.019414	2.2595	0.021541	1	No	NA
ENSG00000122592	HOXA7	7	27193335	27197555	ENSG00000176358	TAC4	17	47915671	47925379	0.0085392	10.406	2.16E-08	0.00383124	0.0022956	0.52748	0.71557	1	Yes	No
ENSG00000177096	FAM109B	22	42470255	42475445	ENSG00000185274	WBSCR17	7	70597155	71178585	0.01011	7.0473	2.16E-08	0.00383124	NA	NA	NA	NA	No	NA
ENSG00000100216	TOMM22	22	39077953	39080818	ENSG00000157502	MUM1L1	X	105412298	105452949	0.0096093	7.8121	2.18E-08	0.00386014	NA	NA	NA	NA	No	NA
ENSG00000178467	P4HTM	3	49027319	49043620	ENSG00000134595	SOX3	X	139585152	139587225	0.0085328	10.398	2.20E-08	0.00388192	NA	NA	NA	NA	No	NA
ENSG00000154760	SLFN13	17	33762115	33775856	ENSG00000158578	ALAS2	X	55039919	55057497	0.01193	5.2971	2.20E-08	0.00388953	0.011396	0.8732	0.57431	1	No	NA
ENSG00000163666	HESX1	3	57231944	57260549	ENSG00000005961	ITGA2B	17	42449548	42466873	0.0079331	12.885	2.21E-08	0.00389959	0.00037708	0.11543	0.95107	1	No	NA
ENSG00000168374	ARF4	3	57557090	57583947	ENSG00000185052	SLC24A3	20	19193290	19703581	0.0090793	8.8546	2.23E-08	0.00392231	0.025362	4.7673	0.00026258	0.45610146	Yes	No
ENSG00000108733	PEX12	17	33901814	33905882	ENSG00000174175	SELP	1	169558087	169599431	0.0090795	8.8548	2.23E-08	0.00392231	0.0039248	0.72186	0.6071	1	Yes	Yes
ENSG00000141150	RASL10B	17	34058668	34070540	ENSG00000102145	GATA1	X	48644962	48652718	0.010572	6.4498	2.24E-08	0.00394109	0.01715	2.2783	0.026486	1	No	NA
ENSG00000173402	DAG1	3	49506146	49573048	ENSG00000204290	BTNL2	6	32361740	32374905	0.0085244	10.388	2.24E-08	0.00394109	NA	NA	NA	NA	No	NA
ENSG00000205045	SLFN12L	17	33800708	33864880	ENSG00000211584	SLC48A1	12	48147699	48172978	0.010093	7.0354	2.24E-08	0.00394509	0.010454	1.3795	0.21041	1	No	NA
ENSG00000154760	SLFN13	17	33762115	33775856	ENSG00000090674	MCOLN1	19	7587512	7595387	0.011919	5.2921	2.25E-08	0.00395576	0.0087485	0.66855	0.78268	1	Yes	No
ENSG00000092871	RFFL	17	33341759	33416338	ENSG00000115649	CNPPD1	2	220036619	220042035	0.0095912	7.7973	2.27E-08	0.00398178	NA	NA	NA	NA	Yes	No
ENSG00000139330	KERA	12	91444268	91451760	ENSG00000241058	NSUN6	10	18834490	18905222	0.007254	17.665	2.27E-08	0.00398178	0.00053979	0.24817	0.78028	1	No	NA
ENSG00000188613	NANOS1	10	120789228	120793854	ENSG00000171790	SLFNL1	1	41481269	41488909	0.011027	5.9812	2.28E-08	0.00398818	0.013305	1.3664	0.19892	1	No	NA
ENSG00000179673	RPRML	17	45055523	45056614	ENSG00000183150	GPR19	12	12813825	12849141	0.0095897	7.7961	2.28E-08	0.00398845	0.0075975	0.99961	0.42996	1	No	NA
ENSG00000139330	KERA	12	91444268	91451760	ENSG00000133706	LARS	5	145492601	145562223	0.0072515	17.659	2.28E-08	0.00399502	0.0029822	1.3744	0.2535	1	No	NA
ENSG00000073598	FNDC8	17	33448598	33454436	ENSG00000022840	RNF10	12	120971283	121015397	0.007914	12.854	2.31E-08	0.00403896	0.014025	4.3526	0.0046959	1	No	NA
ENSG00000100890	KIAA0391	14	35593499	35593616	ENSG00000157017	GHRL	3	10328388	10334631	0.011017	5.9759	2.32E-08	0.00405699	0.060623	6.5396	4.52E-09	8.45E-06	Yes	No
ENSG00000182968	SOX1	13	112721913	112726020	ENSG00000113140	SPARC	5	151040657	151066726	0.0095767	7.7854	2.35E-08	0.00409191	0.0035476	0.54293	0.77572	1	No	NA
ENSG00000185909	KLHDC8B	3	49209044	49213917	ENSG00000120937	NPPB	1	11917521	11918988	0.0079037	12.837	2.37E-08	0.00412851	NA	NA	NA	NA	No	NA
ENSG00000051341	POLQ	3	121150278	121264853	ENSG00000158022	TRIM63	1	26377795	26394927	0.0078999	12.831	2.39E-08	0.00416209	NA	NA	NA	NA	No	NA
ENSG00000212916	MAP10	1	232940643	232946092	ENSG00000165092	ALDH1A1	9	75515578	75695358	0.0095658	7.7765	2.40E-08	0.00418222	0.011274	1.7389	0.1089	1	No	NA
ENSG00000177963	RIC8A	11	207511	215113	ENSG00000124491	F13A1	6	6144318	6321246	0.0090403	8.8162	2.43E-08	0.00422908	0.014257	1.6506	0.10669	1	Yes	Yes
ENSG00000186075	ZPBP2	17	38024417	38034149	ENSG00000146592	CREB5	7	28338940	28865511	0.0064132	31.214	2.44E-08	0.00423297	0.0001964	0.18073	0.67085	1	Yes	No
ENSG00000185379	RAD51D	17	33426811	33447063	ENSG00000167671	UBXN6	19	4446046	4457819	0.0078878	12.811	2.46E-08	0.00426912	0.0044054	1.354	0.25556	1	No	NA
ENSG00000184922	FMNL1	17	43298811	43324687	ENSG00000183150	GPR19	12	12813825	12849141	0.01005	7.0047	2.47E-08	0.00428493	0.0072556	0.9543	0.46359	1	No	NA
ENSG00000139330	KERA	12	91444268	91451760	ENSG00000132300	PTCD3	2	86333305	86369280	0.0072125	17.563	2.51E-08	0.00435286	0.00090411	0.41581	0.65993	1	No	NA
ENSG00000166816	LDHD	16	75145758	75150669	ENSG00000160307	S100B	21	48018875	48025121	0.010037	6.9959	2.54E-08	0.00439609	0.0052268	0.68605	0.684	1	No	NA
ENSG00000185379	RAD51D	17	33426811	33447063	ENSG00000154146	NRGN	11	124609829	124615878	0.0078713	12.784	2.56E-08	0.0044249	0.0021963	0.67355	0.56836	1	No	NA
ENSG00000172716	SLFN11	17	33677324	33700720	ENSG00000238243	OR2W3	1	248058859	248060449	0.012283	5.0003	2.56E-08	0.00442814	0.01322	1.0149	0.43262	1	Yes	No
ENSG00000172716	SLFN11	17	33677324	33700720	ENSG00000162722	TRIM58	1	248020501	248031169	0.012283	5.0003	2.56E-08	0.00442814	0.019777	1.5283	0.10811	1	No	NA
ENSG00000108733	PEX12	17	33901814	33905882	ENSG00000159335	PTMS	12	6874682	6880116	0.0090167	8.7931	2.57E-08	0.00442848	0.012733	2.3627	0.038272	1	Yes	Yes
ENSG00000141741	MIEN1	17	37885409	37886014	ENSG00000196663	TECPR2	14	102829300	102968818	0.009014	8.7904	2.58E-08	0.00444442	0.004185	0.76991	0.57154	1	Yes	No
ENSG00000174839	DENND6A	3	57611184	57678816	ENSG00000259207	ITGB3	17	45387505	45389182	0.0084629	10.313	2.58E-08	0.00444442	0.03238	7.6715	4.42E-06	0.00801346	No	NA
ENSG00000006125	AP2B1	17	33913918	34053436	ENSG00000100243	CYB5R3	22	43013846	43045574	0.01003	6.991	2.58E-08	0.00444442	0.0098745	1.5209	0.16804	1	No	NA
ENSG00000141150	RASL10B	17	34058668	34070540	ENSG00000204463	BAG6	6	31606805	31620170	0.010503	6.4074	2.60E-08	0.00446978	0.034097	4.6092	4.43E-05	0.0785882	Yes	No
ENSG00000136213	CHST12	7	2443223	2474242	ENSG00000150636	CCDC102B	18	66383765	66722426	0.010503	6.4072	2.60E-08	0.00446978	0.0089075	0.91074	0.51499	1	No	NA
ENSG00000142102	ATHL1	11	289138	296107	ENSG00000172572	PDE3A	12	20522179	20837315	0.015065	3.8785	2.62E-08	0.0044882	NA	NA	NA	NA	Yes	Yes
ENSG00000141150	RASL10B	17	34058668	34070540	ENSG00000130733	YIPF2	19	11034171	11039357	0.0105	6.4056	2.62E-08	0.0044882	0.010651	1.4057	0.19937	1	No	NA
ENSG00000141150	RASL10B	17	34058668	34070540	ENSG00000196961	AP2A1	19	50270225	50309510	0.010496	6.4031	2.64E-08	0.00452213	0.0062562	0.82202	0.56901	1	Yes	No
ENSG00000158315	RHBDL2	1	39351479	39407471	ENSG00000100079	LGALS2	22	37966255	37978623	0.010957	5.9431	2.64E-08	0.00452213	0.0057759	0.52924	0.87024	1	No	NA
ENSG00000161298	ZNF382	19	37098454	37119499	ENSG00000111291	GPRC5D	12	13093709	13105081	0.0084506	10.297	2.66E-08	0.00454624	0.026884	6.3334	4.99E-05	0.0884228	No	NA
ENSG00000174885	NLRP6	11	278365	285359	ENSG00000095303	PTGS1	9	125132824	125157982	0.010013	6.979	2.68E-08	0.00457749	0.018988	1.9613	0.040764	1	Yes	Yes
ENSG00000132139	GAS2L2	17	34071530	34079897	ENSG00000198876	DCAF12	9	34086385	34127397	0.010488	6.3978	2.69E-08	0.00459349	0.0053927	0.61878	0.76258	1	No	NA
ENSG00000142082	SIRT3	11	215458	236431	ENSG00000161911	TREML1	6	41117080	41122075	0.0078492	12.748	2.70E-08	0.00460144	0.0084421	1.9518	0.099868	1	Yes	Yes
ENSG00000254004	ZNF260	19	37001597	37019562	ENSG00000111291	GPRC5D	12	13093709	13105081	0.0095095	7.7302	2.73E-08	0.00464995	0.027667	2.8833	0.0022994	1	No	NA

ENSG00000004534	RBM6	3	49977440	50114683	ENSG00000162753	SLC9C2	1	173469603	173572233	0.0071749	17.471	2.75E-08	0.00469106	NA	NA	NA	NA	No	NA
ENSG00000139330	KERA	12	91444268	91451760	ENSG00000155545	MIER3	5	56215429	56267502	0.0071723	17.464	2.77E-08	0.00471695	0.0021329	0.98218	0.37489	1	No	NA
ENSG00000092871	RFFL	17	33341759	33416338	ENSG00000198876	DCAF12	9	34086385	34127397	0.0094959	7.7191	2.81E-08	0.00478107	0.012925	1.4944	0.15497	1	No	NA
ENSG00000151327	FAM177A1	14	35514113	35582336	ENSG00000157017	GHRL	3	10328388	10334631	0.01181	5.2431	2.83E-08	0.00480107	0.063186	5.5798	1.06E-08	1.97E-05	Yes	Yes
ENSG00000168374	ARF4	3	57557090	57583947	ENSG00000158457	TSPAN33	7	128784712	128808637	0.0089738	8.7508	2.83E-08	0.0048066	0.0040383	0.74281	0.59149	1	Yes	No
ENSG00000108733	PEX12	17	33901814	33905882	ENSG00000137193	PIM1	6	37137979	37143202	0.008972	8.7491	2.84E-08	0.00481797	0.014286	2.6551	0.02156	1	No	NA
ENSG00000177096	FAM109B	22	42470255	42475445	ENSG00000172935	MRGPRF	11	68771863	68780877	0.0099865	6.9602	2.84E-08	0.00481797	NA	NA	NA	NA	No	NA
ENSG00000185379	RAD51D	17	33426811	33447063	ENSG00000162722	TRIM58	1	248020501	248031169	0.0078175	12.696	2.91E-08	0.00492019	0.0056859	1.7498	0.15522	1	No	NA
ENSG00000185379	RAD51D	17	33426811	33447063	ENSG00000238243	OR2W3	1	248058859	248060449	0.0078175	12.696	2.91E-08	0.00492019	0.0053417	1.6433	0.17779	1	No	NA
ENSG00000163946	FAM208A	3	56658507	56717265	ENSG00000148498	PARD3	10	34398488	35104253	0.010451	6.3753	2.91E-08	0.00492052	0.0077181	1.0156	0.41844	1	Yes	No
ENSG00000132382	MYBBP1A	17	4443643	4458926	ENSG00000132704	FCRL2	1	157715523	157746922	0.0089581	8.7353	2.93E-08	0.00495752	0.010299	1.1876	0.30319	1	No	NA
ENSG00000154760	SLFN13	17	33762115	33775856	ENSG00000154146	NRGN	11	124609829	124615878	0.011785	5.2322	2.97E-08	0.00501211	0.013775	1.058	0.39292	1	Yes	No
ENSG00000139330	KERA	12	91444268	91451760	ENSG00000075711	DLG1	3	196769431	197026171	0.0071436	17.394	2.97E-08	0.00501211	0.0013255	0.60988	0.54364	1	No	NA
ENSG00000139330	KERA	12	91444268	91451760	ENSG00000015479	MATR3	5	138609441	138667360	0.0071405	17.386	2.99E-08	0.00504143	0.0044962	2.0753	0.1261	1	No	NA
ENSG00000136643	RPS6KC1	1	213224589	213448116	ENSG00000144619	CNTN4	3	2140497	3099645	0.0089495	8.7269	2.99E-08	0.00504143	NA	NA	NA	NA	No	NA
ENSG00000006125	AP2B1	17	33913918	34053436	ENSG00000159335	PTMS	12	6874682	6880116	0.0099578	6.94	3.03E-08	0.00508996	0.016514	2.5606	0.018263	1	Yes	Yes
ENSG00000122548	KIAA0087	7	26572740	26578407	ENSG00000176358	TAC4	17	47915671	47925379	0.0089428	8.7203	3.04E-08	0.0051078	0.0050793	0.77855	0.58684	1	No	NA
ENSG00000099849	RASSF7	11	560404	564021	ENSG00000173210	ABLIM3	5	148521046	148640105	0.0099541	6.9374	3.05E-08	0.0051184	0.0092076	1.2134	0.29235	1	Yes	No
ENSG00000154760	SLFN13	17	33762115	33775856	ENSG00000150045	KLRF1	12	9980077	9997606	0.011773	5.2267	3.05E-08	0.0051184	0.020659	1.598	0.086615	1	No	NA
ENSG00000243708	PLA2G4B	15	42129973	42130157	ENSG00000153157	SYCP2L	6	10887064	10979553	0.0083896	10.222	3.06E-08	0.00513602	0.011817	2.1907	0.05327	1	No	NA
ENSG00000154760	SLFN13	17	33762115	33775856	ENSG00000168785	TSPAN5	4	99391518	99579780	0.01177	5.2251	3.07E-08	0.00514439	0.010476	0.80197	0.64877	1	Yes	No
ENSG00000177096	FAM109B	22	42470255	42475445	ENSG00000179403	VWA1	1	1370241	1378262	0.0099492	6.9339	3.08E-08	0.00516145	NA	NA	NA	NA	No	NA
ENSG00000184886	PIGW	17	34892943	34895159	ENSG00000070182	SPTB	14	65216372	65346601	0.0083851	10.217	3.09E-08	0.00517748	0.0055789	1.0278	0.39988	1	No	NA
ENSG00000115464	USP34	2	61416045	61697904	ENSG00000187017	ESPN	1	6484848	6521040	0.011328	5.5308	3.11E-08	0.00519025	0.081283	8.06	1.50E-12	2.84E-09	Yes	No
ENSG00000073598	FNDC8	17	33448598	33454436	ENSG00000166947	EPB42	15	43398423	43513481	0.0077895	12.65	3.11E-08	0.00519025	0.0032252	0.99011	0.39672	1	No	NA
ENSG00000100226	GTPBP1	22	39101728	39130402	ENSG00000163064	EN1	2	119599747	119605254	0.0083783	10.209	3.14E-08	0.00524611	NA	NA	NA	NA	No	NA
ENSG00000154174	TOMM70A	3	100082275	100105822	ENSG00000196724	ZNF418	19	58433252	58446761	0.010873	5.8967	3.16E-08	0.00527569	0.043923	5.9985	7.53E-07	0.00138251	No	NA
ENSG00000205045	SLFN12L	17	33800708	33864880	ENSG00000179632	MAF1	8	145159402	145162514	0.009936	6.9246	3.17E-08	0.0052874	0.012558	1.6606	0.11515	1	No	NA
ENSG00000186075	ZPBP2	17	38024417	38034149	ENSG00000183762	KREMEN1	22	29469066	29564321	0.0063067	30.693	3.18E-08	0.00529892	0.00063787	0.58721	0.4437	1	No	NA
ENSG00000178035	IMPDH2	3	49061758	49066823	ENSG00000134595	SOX3	X	139585152	139587225	0.0077742	12.625	3.22E-08	0.00536066	NA	NA	NA	NA	No	NA
ENSG00000114383	TUSC2	3	50362338	50365682	ENSG00000120937	NPPB	1	11917521	11918988	0.0077735	12.624	3.23E-08	0.00536396	NA	NA	NA	NA	No	NA
ENSG00000108733	PEX12	17	33901814	33905882	ENSG00000158578	ALAS2	X	55039919	55057497	0.0089138	8.6918	3.25E-08	0.00538934	0.0028122	0.51665	0.76382	1	No	NA
ENSG00000129282	MRM1	17	34958001	34965407	ENSG00000070182	SPTB	14	65216372	65346601	0.0094235	7.6597	3.31E-08	0.00548521	0.0029884	0.4571	0.84016	1	No	NA
ENSG00000175213	ZNF408	11	46722368	46727462	ENSG00000119121	TRPM6	9	77337411	77503010	0.010391	6.3379	3.32E-08	0.00550332	0.0050444	0.662	0.70442	1	No	NA
ENSG00000188895	MSL1	17	38278551	38291643	ENSG00000170525	PFKFB3	10	6186881	6277495	0.011731	5.208	3.32E-08	0.0055045	0.0063267	1.9483	0.12021	1	Yes	No
ENSG00000065621	GSTO2	10	106028631	106059616	ENSG00000114648	KLHL18	3	47328710	47388306	0.0094192	7.6561	3.34E-08	0.00552505	0.01023	1.5762	0.15085	1	No	NA
ENSG00000163946	FAM208A	3	56658507	56717265	ENSG00000127920	GNG11	7	93551011	93555831	0.010384	6.3341	3.36E-08	0.00556426	0.030217	4.0684	0.00020908	0.36505368	Yes	No
ENSG00000177951	BET1L	11	167784	207428	ENSG00000137801	THBS1	15	39873280	39891667	0.010379	6.3306	3.40E-08	0.00562737	0.0078392	0.65364	0.78291	1	Yes	Yes
ENSG00000174885	NLRP6	11	278365	285359	ENSG00000102362	SYTL4	X	99929488	99987110	0.009899	6.8986	3.44E-08	0.0056846	0.022767	2.3608	0.012283	1	Yes	Yes
ENSG00000164078	MST1R	3	49924435	49936696	ENSG00000131059	BPIFA3	20	31805116	31815564	0.0083385	10.16	3.45E-08	0.0056896	NA	NA	NA	NA	No	NA
ENSG00000167914	GSDMA	17	38119226	38134019	ENSG00000170525	PFKFB3	10	6186881	6277495	0.0070819	17.243	3.45E-08	0.00569343	0.010365	4.8128	0.0083314	1	Yes	Yes
ENSG00000008838	MED24	17	38175350	38217468	ENSG00000170525	PFKFB3	10	6186881	6277495	0.0083354	10.156	3.47E-08	0.0057223	0.01195	2.7728	0.026157	1	Yes	No
ENSG00000165949	IFI27	14	94571182	94583033	ENSG00000151846	PABPC3	13	25670300	25673389	0.010365	6.3222	3.51E-08	0.00577301	0.0070278	0.92412	0.48677	1	No	NA
ENSG00000168374	ARF4	3	57557090	57583947	ENSG00000205038	PKHD1L1	8	110374706	110542559	0.0088769	8.6554	3.53E-08	0.00580637	0.02411	4.5262	0.00044089	0.75744902	Yes	No
ENSG00000141150	RASL10B	17	34058668	34070540	ENSG00000134470	IL15RA	10	5990855	6020150	0.010359	6.3182	3.56E-08	0.00584543	0.0091841	1.2103	0.2941	1	No	NA
ENSG00000172738	TMEM217	6	37179956	37225413	ENSG00000049247	UTS2	1	7906752	7913572	0.0098825	6.887	3.57E-08	0.00585157	0.0067922	0.89293	0.51131	1	No	NA
ENSG00000088826	SMOX	20	4101627	4168394	ENSG00000149212	SESN3	11	94898704	94965705	0.010816	5.8659	3.57E-08	0.00585157	0.0070458	0.71904	0.69178	1	No	NA
ENSG00000213171	LINGO4	1	151772740	151775193	ENSG00000049323	LTBP1	2	33172039	33624576	0.0093892	7.6315	3.57E-08	0.00585157	0.0021836	0.33373	0.91928	1	No	NA
ENSG00000149016	TUT1	11	62342517	62350064	ENSG00000171435	KSR2	12	117890817	118406788	0.0088717	8.6503	3.57E-08	0.00585181	NA	NA	NA	NA	No	NA
ENSG00000067560	RHOA	3	49396578	49449635	ENSG00000103023	PRSS54	16	58318309	58328951	0.0083224	10.14	3.58E-08	0.00585925	NA	NA	NA	NA	No	NA
ENSG00000121101	TEX14	17	56634039	56769416	ENSG00000163694	RBM47	4	40425272	40632892	0.009385	7.628	3.60E-08	0.00589252	0.015879	1.8414	0.066068	1	No	NA
ENSG00000119408	NEK6	9	127019885	127115586	ENSG00000213085	CCDC19	1	159842154	159869953	0.0083155	10.131	3.64E-08	0.00594486	0.029671	5.602	4.29E-05	0.0761475	No	NA
ENSG00000131127	ZNF141	4	331603	378653	ENSG00000154263	ABCA10	17	67143355	67225001	0.0088625	8.6413	3.65E-08	0.00595285	0.0042204	0.64633	0.69315	1	No	NA
ENSG00000117481	NSUN4	1	46805849	46830824	ENSG00000204188	GGNBP1	6	33551515	33556803	0.0083142	10.13	3.65E-08	0.00595285	NA	NA	NA	NA	No	NA
ENSG00000100207	TCF20	22	42556019	42739622	ENSG00000175556	LONRF3	X	118108581	118156888	0.0083074	10.121	3.71E-08	0.00604366	0.0020354	0.46756	0.75959	1	No	NA
ENSG00000132139	GAS2L2	17	34071530	34079897	ENSG00000166947	EPB42	15	43398423	43513481	0.010339	6.3059	3.71E-08	0.0060483	0.0083231	0.95784	0.46779	1	Yes	No
ENSG00000006125	AP2B1	17	33913918	34053436	ENSG00000170540	ARL6IP1	16	18802991	18812746	0.0098598	6.871	3.75E-08	0.00610262	0.0085999	1.3229	0.24401	1	No	NA
ENSG00000159674	SPON2	4	1160720	1202750	ENSG00000154263	ABCA10	17	67143355	67225001	0.010793	5.8529	3.75E-08	0.00610262	0.0086617	0.99716	0.43654	1	No	NA
ENSG00000205045	SLFN12L	17	33800708	33864880	ENSG00000135924	DNAJB2	2	220143989	220151622	0.0098564	6.8686	3.78E-08	0.00614329	0.0088541	1.1664	0.31947	1	Yes	Yes
ENSG00000141971	MVB12A	19	17529890	17544533	ENSG00000116106	EPHA4	2	222282747	222438922	0.013316	4.3385	3.81E-08	0.00619039	0.0055644	0.85332	0.52892	1	No	NA
ENSG00000108733	PEX12	17	33901814	33905882	ENSG00000104522	TSTA3	8	144694788	144700218	0.0088403	8.6194	3.84E-08	0.00622573	0.010875	2.0142	0.074329	1	No	NA
ENSG00000163666	HESX1	3	57231944	57260549	ENSG00000259207	ITGB3	17	45387505	45389182	0.0076982	12.501	3.86E-08	0.00625777	0.0041362	1.2709	0.28307	1	No	NA
ENSG00000174885	NLRP6	11	278365	285359	ENSG00000138798	EGF	4	110834040	110933422	0.0098448	6.8604	3.88E-08	0.00628166	0.0082151	0.83936	0.57985	1	Yes	Yes
ENSG00000112706	IMPG1	6	76630832	76782395	ENSG00000124102	PI3	20	43803517	43805185	0.0098419	6.8584	3.90E-08	0.00631653	0.02701	3.6246	0.00073004	1	No	NA
ENSG00000062038	CDH3	16	68670092	68756519	ENSG00000255833	TIFAB	5	134787899	134788089	0.0093474	7.5973	3.92E-08	0.00633178	0.0084909	1.1182	0.34918	1	Yes	No
ENSG00000188511	C22orf34	22	49808176	50051190	ENSG00000197641	SERPINB13	18	61254223	61271873	0.0088314	8.6107	3.92E-08	0.00633178	NA	NA	NA	NA	No	NA
ENSG00000141002	TCF25	16	89940014	89977792	ENSG00000164626	KCNK5	6	39156749	39197226	0.0088289	8.6082	3.94E-08	0.00635876	0.0011502	0.1756	0.98343	1	No	NA

ENSG00000167523	SPATA33	16	89734401	89737680	ENSG00000175718	RBMXL3	X	114423963	114427431	0.0098341	6.8529	3.97E-08	0.00640507	NA	NA	NA	NA	No	NA
ENSG00000006125	AP2B1	17	33913918	34053436	ENSG00000177666	PNPLA2	11	818902	825573	0.0098331	6.8522	3.98E-08	0.00641503	0.0039361	0.60263	0.72841	1	No	NA
ENSG00000172123	SLFN12	17	33738079	33760302	ENSG00000138867	GUCD1	22	24936406	24951284	0.013293	4.3309	3.99E-08	0.00642594	NA	NA	NA	NA	Yes	No
ENSG00000108733	PEX12	17	33901814	33905882	ENSG00000063854	HAGH	16	1845621	1873038	0.008822	8.6015	4.00E-08	0.00643355	0.0031472	0.57839	0.71661	1	No	NA
ENSG00000184163	FAM132A	1	1177833	1182102	ENSG00000184730	APOBR	16	28505970	28510291	0.0062157	30.247	4.00E-08	0.00643355	NA	NA	NA	NA	No	NA
ENSG00000108733	PEX12	17	33901814	33905882	ENSG00000011105	TSPAN9	12	3186521	3395730	0.0088198	8.5993	4.02E-08	0.00645623	0.049826	9.6067	5.97E-09	1.11E-05	Yes	No
ENSG00000108733	PEX12	17	33901814	33905882	ENSG00000170540	ARL6IP1	16	18802991	18812746	0.0088201	8.5996	4.02E-08	0.00645623	0.0070275	1.2965	0.26309	1	No	NA
ENSG00000135535	CD164	6	109687717	109703762	ENSG00000134755	DSC2	18	28645940	28682378	0.010759	5.8344	4.03E-08	0.00646738	0.011696	1.3506	0.2147	1	Yes	Yes
ENSG00000108306	FBXL20	17	37415384	37558776	ENSG00000112053	SLC26A8	6	35911291	35992645	0.0088182	8.5977	4.03E-08	0.00647018	0.0096165	1.7789	0.11454	1	No	NA
ENSG00000172057	ORMDL3	17	38077294	38083854	ENSG00000077238	IL4R	16	27324989	27376099	0.0088153	8.5948	4.06E-08	0.00650758	0.015228	2.3581	0.028851	1	Yes	Yes
ENSG00000172660	TAF15	17	34136459	34174246	ENSG00000130830	MPP1	X	154006959	154049282	0.0093309	7.5837	4.07E-08	0.00650906	0.017038	2.6434	0.01511	1	No	NA
ENSG00000139330	KERA	12	91444268	91451760	ENSG00000018510	AGPS	2	178284997	178408564	0.0070108	17.068	4.11E-08	0.00656894	0.00038479	0.17688	0.83791	1	No	NA
ENSG00000139330	KERA	12	91444268	91451760	ENSG00000085760	MTIF2	2	55463731	55496483	0.0070098	17.066	4.12E-08	0.006579	0.0021806	1.0042	0.36676	1	No	NA
ENSG00000177096	FAM109B	22	42470255	42475445	ENSG00000119596	YLPM1	14	75230069	75302893	0.0098159	6.8401	4.13E-08	0.00659975	0.0047603	0.62453	0.73593	1	No	NA
ENSG00000179546	HTR1D	1	23516993	23521222	ENSG00000106565	TMEM176B	7	150488373	150498448	0.0088039	8.5837	4.17E-08	0.00665175	0.0053628	0.98776	0.42404	1	No	NA
ENSG00000172716	SLFN11	17	33677324	33700720	ENSG00000144567	FAM134A	2	220042939	220050201	0.012041	4.9004	4.21E-08	0.0067193	0.02339	1.8143	0.041853	1	Yes	No
ENSG00000185379	RAD51D	17	33426811	33447063	ENSG00000166947	EPB42	15	43398423	43513481	0.0076581	12.435	4.25E-08	0.00676507	0.0003067	0.093879	0.96342	1	No	NA
ENSG00000163947	ARHGEF3	3	56761446	57113357	ENSG00000010278	CD9	12	6308881	6347425	0.010274	6.2658	4.27E-08	0.0068052	0.015529	1.8002	0.073411	1	No	NA
ENSG00000139330	KERA	12	91444268	91451760	ENSG00000135999	EPC2	2	149402009	149545130	0.0069918	17.022	4.30E-08	0.00684224	0.00146	0.67185	0.51101	1	No	NA
ENSG00000176024	ZNF613	19	52430400	52452012	ENSG00000164659	KIAA1324L	7	86506222	86689015	0.01027	6.2634	4.31E-08	0.0068514	0.026405	4.136	0.00041554	0.71514434	No	NA
ENSG00000141741	MIEN1	17	37885409	37886014	ENSG00000151726	ACSL1	4	185676749	185747972	0.0087836	8.5637	4.36E-08	0.00693096	0.007858	1.451	0.20352	1	Yes	No
ENSG00000164163	ABCE1	4	146025538	146046253	ENSG00000154764	WNT7A	3	13857755	13921618	0.0061807	30.076	4.37E-08	0.00693096	0.0026635	2.457	0.11735	1	No	NA
ENSG00000177096	FAM109B	22	42470255	42475445	ENSG00000131094	C1QL1	17	43037061	43045439	0.0097896	6.8216	4.38E-08	0.00694209	NA	NA	NA	NA	No	NA
ENSG00000164068	RNF123	3	49728563	49753910	ENSG00000182156	ENPP7	17	77704681	77716021	0.0076431	12.41	4.40E-08	0.00697223	NA	NA	NA	NA	No	NA
ENSG00000213719	CLIC1	6	31699994	31707540	ENSG00000101425	BPI	20	36888551	36965907	0.0082329	10.03	4.41E-08	0.00697904	0.015588	3.6302	0.0060611	1	No	NA
ENSG00000088826	SMOX	20	4101627	4168394	ENSG00000100614	PPM1A	14	60712470	60765805	0.010714	5.8095	4.44E-08	0.00702476	0.0090149	0.92182	0.50516	1	No	NA
ENSG00000188076	SCGB1C1	11	193080	194573	ENSG00000049323	LTBP1	2	33172039	33624576	0.0061646	29.997	4.55E-08	0.00718997	0.0038493	3.5551	0.059678	1	Yes	Yes
ENSG00000204296	C6orf10	6	32256303	32339684	ENSG00000163599	CTLA4	2	204732509	204738683	0.0061636	29.992	4.56E-08	0.00719674	0.0082733	7.6749	0.0057121	1	No	NA
ENSG00000008838	MED24	17	38175350	38217468	ENSG00000112053	SLC26A8	6	35911291	35992645	0.0082183	10.012	4.56E-08	0.00719674	0.0095257	2.2048	0.066662	1	No	NA
ENSG00000196260	SFTA2	6	30899130	30899952	ENSG00000169507	SLC38A11	2	165752696	165812035	0.0087618	8.5422	4.59E-08	0.00723648	0.0080368	1.4843	0.19232	1	Yes	No
ENSG00000172057	ORMDL3	17	38077294	38083854	ENSG00000135842	FAM129A	1	184759858	184943682	0.0087583	8.5388	4.62E-08	0.00728568	0.0086324	1.3279	0.24177	1	Yes	Yes
ENSG00000108733	PEX12	17	33901814	33905882	ENSG00000172270	BSG	19	571297	583493	0.0087581	8.5386	4.63E-08	0.00728568	0.0050777	0.93498	0.45738	1	No	NA
ENSG00000139330	KERA	12	91444268	91451760	ENSG00000124275	MTRR	5	7856900	7906138	0.0069605	16.945	4.64E-08	0.00730464	0.0017419	0.80178	0.44884	1	No	NA
ENSG00000172660	TAF15	17	34136459	34174246	ENSG00000133816	MICAL2	11	12115543	12285332	0.0092697	7.5335	4.66E-08	0.00731287	0.013319	2.0585	0.055694	1	Yes	No
ENSG00000172660	TAF15	17	34136459	34174246	ENSG00000133808	MICALCL	11	12297627	12380691	0.0092697	7.5335	4.66E-08	0.00731287	0.015529	2.4055	0.025947	1	Yes	No
ENSG00000185379	RAD51D	17	33426811	33447063	ENSG00000029534	ANK1	8	41510739	41754280	0.0076189	12.371	4.66E-08	0.00731287	0.0080711	2.4898	0.059032	1	No	NA
ENSG00000139330	KERA	12	91444268	91451760	ENSG00000115419	GLS	2	191745553	191819544	0.0069597	16.943	4.65E-08	0.00731287	0.0013053	0.60055	0.54872	1	No	NA
ENSG00000174173	TRMT10C	3	101280706	101285290	ENSG00000196724	ZNF418	19	58433252	58446761	0.0082024	9.9925	4.73E-08	0.00741673	0.02267	5.3177	0.00030941	0.53558871	No	NA
ENSG00000177951	BET1L	11	167784	207428	ENSG00000128266	GNAZ	22	23412540	23464889	0.010226	6.2365	4.74E-08	0.00741983	0.020938	1.7691	0.055087	1	Yes	Yes
ENSG00000172660	TAF15	17	34136459	34174246	ENSG00000136732	GYPC	2	127413509	127454246	0.0092587	7.5245	4.78E-08	0.00747726	0.013927	2.1539	0.045297	1	Yes	No
ENSG00000139330	KERA	12	91444268	91451760	ENSG00000143155	TIPRL	1	168148171	168169950	0.0069447	16.906	4.82E-08	0.00754477	0.0010822	0.4978	0.60803	1	No	NA
ENSG00000114316	USP4	3	49316247	49378145	ENSG00000134595	SOX3	X	139585152	139587225	0.0087384	8.5193	4.84E-08	0.0075609	NA	NA	NA	NA	No	NA
ENSG00000205045	SLFN12L	17	33800708	33864880	ENSG00000186350	RXRA	9	137208944	137332431	0.0097419	6.788	4.86E-08	0.00759153	0.0094777	1.2494	0.2728	1	Yes	Yes
ENSG00000163947	ARHGEF3	3	56761446	57113357	ENSG00000081377	CDC14B	9	99258489	99382112	0.010213	6.2285	4.87E-08	0.00760245	0.025486	2.9847	0.0026247	1	No	NA
ENSG00000205045	SLFN12L	17	33800708	33864880	ENSG00000162873	KLHDC8A	1	205305220	205326218	0.0097392	6.7861	4.89E-08	0.00762427	0.0050473	0.66238	0.70409	1	No	NA
ENSG00000132382	MYBBP1A	17	4443643	4458926	ENSG00000163534	FCRL1	1	157764193	157789895	0.0087332	8.5141	4.90E-08	0.00762907	0.022762	2.6582	0.0069105	1	No	NA
ENSG00000114378	HYAL1	3	50337320	50349812	ENSG00000131059	BPIFA3	20	31805116	31815564	0.0081829	9.9686	4.95E-08	0.00770566	NA	NA	NA	NA	No	NA
ENSG00000139330	KERA	12	91444268	91451760	ENSG00000156976	EIF4A2	3	186500994	186506205	0.0069246	16.857	5.06E-08	0.00788156	0.0023017	1.0601	0.34686	1	No	NA
ENSG00000139330	KERA	12	91444268	91451760	ENSG00000136631	VPS45	1	150039369	150117505	0.0069202	16.846	5.12E-08	0.0079594	0.00032013	0.14715	0.86319	1	No	NA
ENSG00000142082	SIRT3	11	215458	236431	ENSG00000124491	F13A1	6	6144318	6321246	0.0075785	12.305	5.13E-08	0.00796626	0.0075363	1.7408	0.13885	1	Yes	No
ENSG00000088826	SMOX	20	4101627	4168394	ENSG00000143776	CDC42BPA	1	227177566	227506175	0.010644	5.7714	5.15E-08	0.00798848	0.015164	1.5602	0.12274	1	No	NA
ENSG00000163946	FAM208A	3	56658507	56717265	ENSG00000005249	PRKAR2B	7	106685094	106802256	0.010184	6.2105	5.19E-08	0.00804662	0.023448	3.1352	0.0028091	1	Yes	No
ENSG00000008838	MED24	17	38175350	38217468	ENSG00000151726	ACSL1	4	185676749	185747972	0.0081623	9.9433	5.19E-08	0.00804662	0.017086	3.9851	0.0032695	1	Yes	Yes
ENSG00000139330	KERA	12	91444268	91451760	ENSG00000095564	BTAF1	10	93683526	93790082	0.0069111	16.824	5.23E-08	0.00810638	0.0010664	0.49051	0.61247	1	No	NA
ENSG00000186017	ZNF566	19	36936021	36980804	ENSG00000111291	GPRC5D	12	13093709	13105081	0.010633	5.7654	5.27E-08	0.00815304	0.029919	3.1253	0.0010265	1	No	NA
ENSG00000177963	RIC8A	11	207511	215113	ENSG00000161911	TREML1	6	41117080	41122075	0.0086981	8.4796	5.30E-08	0.00820025	0.018157	2.1105	0.032435	1	Yes	Yes
ENSG00000185379	RAD51D	17	33426811	33447063	ENSG00000158828	PINK1	1	20959948	20978004	0.0075616	12.277	5.34E-08	0.0082497	0.0043819	1.3468	0.25785	1	No	NA
ENSG00000139330	KERA	12	91444268	91451760	ENSG00000036549	ZZZ3	1	78028101	78149104	0.0069016	16.801	5.36E-08	0.00827098	0.0046538	2.1484	0.11725	1	No	NA
ENSG00000092020	PPP2R3C	14	35554673	35586382	ENSG00000135929	CYP27A1	2	219646479	219680016	0.0081477	9.9254	5.37E-08	0.00828574	0.0036074	0.83	0.50615	1	Yes	Yes
ENSG00000108733	PEX12	17	33901814	33905882	ENSG00000146535	GNA12	7	2767746	2883958	0.0086898	8.4714	5.40E-08	0.00833086	0.01794	3.3466	0.005304	1	Yes	No
ENSG00000204444	APOM	6	31623248	31625987	ENSG00000100448	CTSG	14	25042762	25045466	0.0092024	7.4783	5.41E-08	0.00833882	0.019931	3.1013	0.0051771	1	Yes	Yes
ENSG00000185379	RAD51D	17	33426811	33447063	ENSG00000140479	PCSK6	15	101840818	102065405	0.0075555	12.267	5.42E-08	0.00833882	0.0028203	0.86545	0.45853	1	No	NA
ENSG00000163946	FAM208A	3	56658507	56717265	ENSG00000119280	C1orf198	1	230972865	231005335	0.010164	6.198	5.42E-08	0.00833917	0.0058666	0.77053	0.61217	1	Yes	No
ENSG00000092871	RFFL	17	33341759	33416338	ENSG00000143416	SELENBP1	1	151336778	151345209	0.0092014	7.4775	5.43E-08	0.00834337	0.0038301	0.43879	0.89802	1	No	NA
ENSG00000132139	GAS2L2	17	34071530	34079897	ENSG00000141084	RANBP10	16	67757005	67840555	0.010162	6.1971	5.44E-08	0.00835187	0.0062712	0.72022	0.67382	1	Yes	No
ENSG00000126062	TMEM115	3	50392180	50392978	ENSG00000131059	BPIFA3	20	31805116	31815564	0.0096888	6.7507	5.46E-08	0.00838076	NA	NA	NA	NA	No	NA
ENSG00000006125	AP2B1	17	33913918	34053436	ENSG00000168497	SDPR	2	192699028	192711981	0.0096877	6.7499	5.47E-08	0.00839474	0.0044911	0.68798	0.65941	1	Yes	Yes

ENSG00000110723	EXPH5	11	108376158	108464465	ENSG00000197409	HIST1H3D	6	26197068	26197497	0.0086819	8.4637	5.50E-08	0.00843077	0.010863	2.012	0.074625	1	No	NA
ENSG00000139330	KERA	12	91444268	91451760	ENSG00000174839	DENND6A	3	57611184	57678816	0.0068888	16.769	5.53E-08	0.00846307	NA	NA	NA	NA	No	NA
ENSG00000243477	NAT6	3	50333833	50335514	ENSG00000204290	BTNL2	6	32361740	32374905	0.0091903	7.4683	5.56E-08	0.00851567	NA	NA	NA	NA	No	NA
ENSG00000132139	GAS2L2	17	34071530	34079897	ENSG00000130300	PLVAP	19	17462257	17488159	0.01015	6.1896	5.58E-08	0.00853486	0.0069538	0.79916	0.60342	1	Yes	No
ENSG00000129993	CBFA2T3	16	88941266	89043612	ENSG00000175718	RBMXL3	X	114423963	114427431	0.009675	6.7409	5.63E-08	0.00859468	NA	NA	NA	NA	No	NA
ENSG00000167768	KRT1	12	53068520	53074191	ENSG00000063515	GSC2	22	19136089	19137796	0.0086718	8.4537	5.63E-08	0.00859468	NA	NA	NA	NA	No	NA
ENSG00000205045	SLFN12L	17	33800708	33864880	ENSG00000013016	EHD3	2	31456880	31492313	0.0096713	6.7383	5.67E-08	0.00865802	0.012647	1.6724	0.11215	1	No	NA
ENSG00000116459	ATP5F1	1	111992304	112005395	ENSG00000158435	CNOT11	2	101874253	101886778	0.0096591	6.7298	5.83E-08	0.00888519	NA	NA	NA	NA	No	NA
ENSG00000142082	SIRT3	11	215458	236431	ENSG00000005961	ITGA2B	17	42449548	42466873	0.007522	12.212	5.86E-08	0.00893374	0.0098338	2.2768	0.059323	1	Yes	No
ENSG00000178035	IMPDH2	3	49061758	49066823	ENSG00000103023	PRSS54	16	58318309	58328951	0.0075213	12.211	5.87E-08	0.00894127	NA	NA	NA	NA	No	NA
ENSG00000006125	AP2B1	17	33913918	34053436	ENSG00000130733	YIPF2	19	11034171	11039357	0.0096549	6.7268	5.88E-08	0.00894665	0.023397	3.6535	0.0013667	1	No	NA
ENSG00000162927	PUS10	2	61169104	61244328	ENSG00000187017	ESPN	1	6484848	6521040	0.0091613	7.4446	5.93E-08	0.00902302	0.052639	10.179	1.66E-09	3.11E-06	No	NA
ENSG00000177963	RIC8A	11	207511	215113	ENSG00000148498	PARD3	10	34398488	35104253	0.0086419	8.4243	6.02E-08	0.00915033	0.018994	2.2096	0.02473	1	No	NA
ENSG00000164078	MST1R	3	49924435	49936696	ENSG00000147488	ST18	8	53023399	53322505	0.0080947	9.8603	6.07E-08	0.00921688	NA	NA	NA	NA	No	NA
ENSG00000225399	RP11-3B7.1	3	49297518	49298744	ENSG00000120937	NPPB	1	11917521	11918988	0.0075069	12.188	6.08E-08	0.00921901	NA	NA	NA	NA	No	NA
ENSG00000120071	KANSL1	17	44107282	44302733	ENSG00000183150	GPR19	12	12813825	12849141	0.010565	5.728	6.09E-08	0.0092278	0.01047	0.96393	0.47347	1	No	NA
ENSG00000118495	PLAGL1	6	144261437	144385735	ENSG00000108405	P2RX1	17	3799886	3819794	0.0086338	8.4164	6.14E-08	0.00928622	0.0067449	1.2441	0.28637	1	No	NA
ENSG00000139330	KERA	12	91444268	91451760	ENSG00000169045	HNRNPH1	5	179041179	179061785	0.0068459	16.664	6.13E-08	0.00928622	0.0015937	0.73349	0.48051	1	No	NA
ENSG00000108733	PEX12	17	33901814	33905882	ENSG00000140479	PCSK6	15	101840818	102065405	0.0086315	8.4141	6.17E-08	0.00932792	0.021886	4.0992	0.0010961	1	Yes	No
ENSG00000108306	FBXL20	17	37415384	37558776	ENSG00000151726	ACSL1	4	185676749	185747972	0.0086297	8.4124	6.19E-08	0.00935666	0.013075	2.427	0.033775	1	Yes	No
ENSG00000243710	WDR65	1	43642792	43720029	ENSG00000133687	TMTC1	12	29653773	29937692	0.0074987	12.174	6.20E-08	0.00935666	0.00074545	0.22828	0.87674	1	No	NA
ENSG00000183657	PP13439	3	171509580	171527714	ENSG00000067225	PKM	15	72491370	72524164	0.0086292	8.4118	6.20E-08	0.00935666	NA	NA	NA	NA	No	NA
ENSG00000167914	GSDMA	17	38119226	38134019	ENSG00000163421	PROK2	3	71820807	71834357	0.0068411	16.652	6.21E-08	0.00935912	0.0057561	2.6602	0.070471	1	Yes	Yes
ENSG00000205045	SLFN12L	17	33800708	33864880	ENSG00000130733	YIPF2	19	11034171	11039357	0.0096299	6.7092	6.21E-08	0.00936068	0.011965	1.5812	0.13723	1	No	NA
ENSG00000139330	KERA	12	91444268	91451760	ENSG00000104626	ERI1	8	8859657	8974256	0.0068332	16.633	6.33E-08	0.00952607	0.0010257	0.47178	0.62404	1	No	NA
ENSG00000174173	TRMT10C	3	101280706	101285290	ENSG00000179909	ZNF154	19	58208735	58220579	0.008076	9.8372	6.34E-08	0.0095421	0.013365	3.1055	0.014917	1	No	NA
ENSG00000177963	RIC8A	11	207511	215113	ENSG00000102362	SYTL4	X	99929488	99987110	0.0086182	8.4011	6.36E-08	0.00955645	0.021614	2.5212	0.010277	1	Yes	Yes
ENSG00000175664	TEX26	13	31506840	31549639	ENSG00000120160	EQTN	9	27284656	27297137	0.008615	8.3979	6.40E-08	0.00962021	NA	NA	NA	NA	No	NA
ENSG00000092871	RFFL	17	33341759	33416338	ENSG00000104904	OAZ1	19	2271369	2273487	0.0091264	7.416	6.41E-08	0.00963073	0.0088593	1.0201	0.41884	1	Yes	No
ENSG00000160716	CHRNB2	1	154540257	154552502	ENSG00000102466	FGF14	13	102378962	103054124	0.009121	7.4115	6.49E-08	0.00974101	NA	NA	NA	NA	No	NA
ENSG00000139330	KERA	12	91444268	91451760	ENSG00000047188	YTHDC2	5	112849380	112930981	0.0068208	16.603	6.52E-08	0.00977511	0.0028794	1.3269	0.2658	1	No	NA
ENSG00000196576	PLXNB2	22	50715058	50746056	ENSG00000258986	TMEM179	14	104941015	105071984	0.0086047	8.3877	6.55E-08	0.00981965	NA	NA	NA	NA	No	NA
ENSG00000073605	GSDMB	17	38060848	38074903	ENSG00000151726	ACSL1	4	185676749	185747972	0.0080582	9.8154	6.61E-08	0.00989317	0.0088393	2.0445	0.086216	1	Yes	Yes
ENSG00000006125	AP2B1	17	33913918	34053436	ENSG00000104522	TSTA3	8	144694788	144700218	0.0096009	6.6888	6.62E-08	0.00990225	0.0069237	1.0632	0.38293	1	No	NA
ENSG00000139330	KERA	12	91444268	91451760	ENSG00000168566	SNRNP48	6	7590432	7612200	0.0068136	16.585	6.63E-08	0.00991575	0.0060114	2.779	0.062626	1	No	NA
ENSG00000004059	ARF5	7	127228399	127231142	ENSG00000125166	GOT2	16	58741035	58757806	0.0091111	7.4034	6.64E-08	0.00991575	0.0056247	0.86261	0.5219	1	No	NA
ENSG00000172123	SLFN12	17	33738079	33760302	ENSG00000017483	SLC38A5	X	48316920	48328644	0.013034	4.2453	6.64E-08	0.00991674	0.012451	1.9228	0.074378	1	Yes	No
ENSG00000104177	MYEF2	15	48441229	48470714	ENSG00000144649	FAM198A	3	43020759	43101703	0.0080548	9.8112	6.66E-08	0.0099272	0.010614	2.4592	0.044021	1	No	NA
ENSG00000104177	MYEF2	15	48441229	48470714	ENSG00000240747	KRBOX1	3	42977834	42984284	0.0080548	9.8112	6.66E-08	0.0099272	NA	NA	NA	NA	No	NA
ENSG00000136371	MTHFS	15	80125927	80189721	ENSG00000214706	IFRD2	3	50325840	50329487	0.0074676	12.123	6.67E-08	0.00993429	0.00095188	0.29155	0.83152	1	No	NA
ENSG00000177096	FAM109B	22	42470255	42475445	ENSG00000088881	EBF4	20	2673480	2740754	0.0095957	6.6852	6.69E-08	0.00996369	0.016474	2.187	0.033236	1	No	NA
ENSG00000139330	KERA	12	91444268	91451760	ENSG00000133997	MED6	14	71047974	71067384	0.0068095	16.575	6.70E-08	0.00996672	0.0019467	0.89623	0.40846	1	No	NA
ENSG00000015532	XYLT2	17	48423453	48440499	ENSG00000107185	RGP1	9	35749284	35752142	0.0095944	6.6843	6.71E-08	0.00997822	0.019015	2.9561	0.007299	1	No	NA
ENSG00000139330	KERA	12	91444268	91451760	ENSG00000163527	STT3B	3	31574130	31679112	0.0068085	16.572	6.72E-08	0.00997826	0.00017153	0.078832	0.9242	1	No	NA
ENSG00000126259	KIRREL2	19	36346763	36355615	ENSG00000116962	NID1	1	236139130	236228462	0.0068075	16.57	6.73E-08	0.00999478	0.022839	10.74	2.45E-05	0.0438305	No	NA
ENSG00000154760	SLFN13	17	33762115	33775856	ENSG00000095321	CRAT	9	131857089	131873083	0.011381	5.0505	6.86E-08	0.01017105	0.014559	1.1191	0.34035	1	Yes	No
ENSG00000172716	SLFN11	17	33677324	33700720	ENSG00000103148	NPRL3	16	138697	188859	0.011801	4.8018	6.86E-08	0.01017105	0.019364	1.4958	0.11962	1	Yes	No
ENSG00000172660	TAF15	17	34136459	34174246	ENSG00000135924	DNAJB2	2	220143989	220151622	0.0090948	7.39	6.88E-08	0.01019243	0.010643	1.6405	0.13281	1	Yes	No
ENSG00000228008	CTD-2330K9.3	3	49943495	49954370	ENSG00000162753	SLC9C2	1	173469603	173572233	0.007454	12.101	6.89E-08	0.01019498	NA	NA	NA	NA	No	NA
ENSG00000163946	FAM208A	3	56658507	56717265	ENSG00000154146	NRGN	11	124609829	124615878	0.010051	6.1289	6.90E-08	0.01020271	0.022519	3.008	0.0039609	1	Yes	No
ENSG00000162063	CCNF	16	2479395	2505565	ENSG00000143552	NUP210L	1	153965161	154127592	0.010049	6.1276	6.93E-08	0.01024323	0.014389	1.6662	0.10269	1	No	NA
ENSG00000203663	OR2L2	1	248201474	248202607	ENSG00000166086	JAM3	11	133938820	134018713	0.009091	7.3869	6.94E-08	0.01025061	0.018173	2.8226	0.0099775	1	Yes	No
ENSG00000139330	KERA	12	91444268	91451760	ENSG00000115524	SF3B1	2	198256698	198299815	0.0067947	16.538	6.95E-08	0.01025253	0.0067185	3.108	0.045159	1	No	NA
ENSG00000131771	PPP1R1B	17	37782993	37792879	ENSG00000128512	DOCK4	7	111366166	111846466	0.010502	5.6935	6.96E-08	0.01025591	0.005075	0.51689	0.86305	1	No	NA
ENSG00000165929	TC2N	14	92251506	92333880	ENSG00000115129	TP53I3	2	24300303	24308731	0.0074494	12.094	6.96E-08	0.01026048	0.0027232	0.62599	0.64405	1	No	NA
ENSG00000204444	APOM	6	31623248	31625987	ENSG00000165617	DACT1	14	59100685	59115039	0.0090873	7.3839	7.00E-08	0.01029558	0.018886	2.9355	0.0076607	1	Yes	No
ENSG00000006125	AP2B1	17	33913918	34053436	ENSG00000197256	KANK2	19	11274943	11308243	0.0095755	6.671	7.00E-08	0.01029558	0.013022	2.012	0.061538	1	No	NA
ENSG00000205045	SLFN12L	17	33800708	33864880	ENSG00000008441	NFIX	19	13106422	13209610	0.0095751	6.6707	7.00E-08	0.01029558	0.0046677	0.61233	0.74608	1	No	NA
ENSG00000183655	KLHL25	15	86302554	86338261	ENSG00000006659	LGALS14	19	40194946	40200084	0.010044	6.1243	7.01E-08	0.01030099	NA	NA	NA	NA	No	NA
ENSG00000169905	TOR1AIP2	1	179809102	179846934	ENSG00000164023	SGMS2	4	108745719	108836203	0.010042	6.1233	7.04E-08	0.01033222	0.0024038	0.31462	0.94758	1	No	NA
ENSG00000196260	SFTA2	6	30899130	30899952	ENSG00000101425	BPI	20	36888551	36965907	0.0085721	8.3557	7.06E-08	0.01035387	0.011261	2.0865	0.064895	1	Yes	No
ENSG00000131849	ZNF132	19	58944181	58951589	ENSG00000071205	ARHGAP10	4	148653214	148993931	0.006787	16.52	7.08E-08	0.01037813	0.022206	10.435	3.30E-05	0.058773	No	NA
ENSG00000139330	KERA	12	91444268	91451760	ENSG00000071246	VASH1	14	77228532	77244414	0.0067837	16.512	7.14E-08	0.01045319	0.00068785	0.31628	0.72893	1	No	NA
ENSG00000139330	KERA	12	91444268	91451760	ENSG00000054282	SDCCAG8	1	243419120	243652442	0.0067828	16.51	7.15E-08	0.0104687	0.00067112	0.30859	0.73456	1	No	NA
ENSG00000172123	SLFN12	17	33738079	33760302	ENSG00000100614	PPM1A	14	60712470	60765805	0.012994	4.2321	7.18E-08	0.0105076	0.0098368	1.515	0.16996	1	Yes	No
ENSG00000129270	MMP28	17	34083268	34122711	ENSG00000022840	RNF10	12	120971283	121015397	0.0090742	7.3731	7.21E-08	0.01053417	0.012234	1.6172	0.1268	1	No	NA
ENSG00000172660	TAF15	17	34136459	34174246	ENSG00000163554	SPTA1	1	158580278	158656488	0.0090704	7.37	7.27E-08	0.01061635	0.0053184	0.8154	0.558	1	Yes	No

ENSG00000154768	C17orf50	17	34087916	34092098	ENSG00000164849	GPR146	7	1084212	1098897	0.0090648	7.3654	7.36E-08	0.01074162	0.0075555	0.99404	0.43401	1	Yes	No
ENSG00000108733	PEX12	17	33901814	33905882	ENSG00000183508	FAM46C	1	118148556	118170994	0.0085526	8.3365	7.38E-08	0.01076184	0.0061962	1.1422	0.33623	1	Yes	Yes
ENSG00000127955	GNAI1	7	79763271	79848718	ENSG00000196867	ZFP28	19	57050317	57068169	0.01134	5.0321	7.46E-08	0.01087925	0.0088524	0.73888	0.70148	1	No	NA
ENSG00000129270	MMP28	17	34083268	34122711	ENSG00000115649	CNPPD1	2	220036619	220042035	0.0090579	7.3598	7.47E-08	0.01088506	NA	NA	NA	NA	No	NA
ENSG00000172716	SLFN11	17	33677324	33700720	ENSG00000130300	PLVAP	19	17462257	17488159	0.011759	4.7843	7.48E-08	0.01089407	0.016379	1.2614	0.23632	1	Yes	No
ENSG00000172716	SLFN11	17	33677324	33700720	ENSG00000140564	FURIN	15	91411822	91426688	0.011757	4.7837	7.51E-08	0.01091848	0.010247	0.78423	0.66724	1	Yes	No
ENSG00000172123	SLFN12	17	33738079	33760302	ENSG00000158828	PINK1	1	20959948	20978004	0.01297	4.2242	7.53E-08	0.01094722	0.007518	1.1552	0.3283	1	Yes	No
ENSG00000006125	AP2B1	17	33913918	34053436	ENSG00000125818	PSMF1	20	1093906	1149022	0.0095412	6.6469	7.54E-08	0.01095515	0.006536	1.0033	0.42172	1	No	NA
ENSG00000099849	RASSF7	11	560404	564021	ENSG00000144677	CTDSPL	3	37903451	38025960	0.0095405	6.6463	7.55E-08	0.01096496	0.009854	1.2995	0.24725	1	Yes	No
ENSG00000139330	KERA	12	91444268	91451760	ENSG00000138069	RAB1A	2	65316073	65357240	0.0067594	16.452	7.57E-08	0.01098113	0.0037095	1.7108	0.18129	1	No	NA
ENSG00000009950	MLXIPL	7	73007524	73038873	ENSG00000140287	HDC	15	50534144	50558223	0.0079991	9.7429	7.58E-08	0.01098293	0.0079291	1.2188	0.294	1	Yes	No
ENSG00000163947	ARHGEF3	3	56761446	57113357	ENSG00000099256	PRTFDC1	10	25137536	25241533	0.010005	6.1005	7.62E-08	0.01103862	0.023957	2.8012	0.0045382	1	No	NA
ENSG00000139330	KERA	12	91444268	91451760	ENSG00000114850	SSR3	3	156257929	156272973	0.0067564	16.445	7.63E-08	0.01103862	0.0069134	3.1988	0.041265	1	No	NA
ENSG00000142102	ATHL1	11	289138	296107	ENSG00000102362	SYTL4	X	99929488	99987110	0.014496	3.7301	7.66E-08	0.01106526	0.0058649	2.7108	0.067015	1	Yes	Yes
ENSG00000241360	PDXP	22	38054734	38061110	ENSG00000103148	NPRL3	16	138697	188859	0.0090472	7.351	7.65E-08	0.01106526	0.0025608	0.39152	0.88473	1	No	NA
ENSG00000188076	SCGB1C1	11	193080	194573	ENSG00000259207	ITGB3	17	45387505	45389182	0.0059557	28.974	7.68E-08	0.01108821	0.0013753	1.267	0.26063	1	Yes	Yes
ENSG00000121101	TEX14	17	56634039	56769416	ENSG00000146592	CREB5	7	28338940	28865511	0.0090454	7.3496	7.68E-08	0.01108821	0.015375	1.782	0.076888	1	No	NA
ENSG00000258405	ZNF578	19	52956829	53015407	ENSG00000164659	KIAA1324L	7	86506222	86689015	0.0079867	9.7276	7.80E-08	0.01124713	0.024585	3.291	0.0018366	1	No	NA
ENSG00000212916	MAP10	1	232940643	232946092	ENSG00000138760	SCARB2	4	77079890	77135046	0.0090366	7.3423	7.83E-08	0.01129197	0.0042525	0.65128	0.68914	1	No	NA
ENSG00000075399	VPS9D1	16	89773542	89785777	ENSG00000165171	WBSCR27	7	73248920	73256865	0.0085247	8.3091	7.86E-08	0.01131918	0.0094775	1.4592	0.1892	1	No	NA
ENSG00000177963	RIC8A	11	207511	215113	ENSG00000185245	GP1BA	17	4835592	4838325	0.0085235	8.3079	7.88E-08	0.01134303	0.013658	1.5803	0.12651	1	Yes	Yes
ENSG00000172057	ORMDL3	17	38077294	38083854	ENSG00000112053	SLC26A8	6	35911291	35992645	0.0085211	8.3056	7.92E-08	0.0113962	0.0077355	1.1889	0.3098	1	No	NA
ENSG00000108349	CASC3	17	38296576	38328436	ENSG00000162551	ALPL	1	21835858	21904905	0.0090287	7.3358	7.98E-08	0.01145119	0.0019891	0.30394	0.935	1	Yes	No
ENSG00000121101	TEX14	17	56634039	56769416	ENSG00000118495	PLAGL1	6	144261437	144385735	0.0090285	7.3356	7.98E-08	0.01145119	0.0052273	0.5997	0.77864	1	No	NA
ENSG00000174885	NLRP6	11	278365	285359	ENSG00000185532	PRKG1	10	52750945	54058110	0.0095158	6.629	7.97E-08	0.01145119	NA	NA	NA	NA	No	NA
ENSG00000180376	CCDC66	3	56591189	56653929	ENSG00000122786	CALD1	7	134429003	134655479	0.0085175	8.302	7.99E-08	0.01145581	0.011127	2.0614	0.068029	1	Yes	No
ENSG00000158805	ZNF276	16	89786808	89800446	ENSG00000165171	WBSCR27	7	73248920	73256865	0.0099832	6.0868	7.99E-08	0.01145581	0.0040937	0.46912	0.87831	1	No	NA
ENSG00000137776	SLTM	15	59171244	59225852	ENSG00000167778	SPRYD3	12	53458388	53473204	0.0090264	7.3339	8.02E-08	0.01147989	0.011109	1.7132	0.11474	1	No	NA
ENSG00000139370	SLC15A4	12	129277739	129308528	ENSG00000244057	LCE3C	1	152573138	152573562	0.0099805	6.0852	8.04E-08	0.01150593	NA	NA	NA	NA	No	NA
ENSG00000006125	AP2B1	17	33913918	34053436	ENSG00000151693	ASAP2	2	9346894	9541525	0.0095103	6.6251	8.07E-08	0.01154067	0.0052255	0.80107	0.56914	1	Yes	Yes
ENSG00000225697	SLC26A6	3	48663156	48672926	ENSG00000134595	SOX3	X	139585152	139587225	0.0079709	9.7082	8.09E-08	0.01156092	NA	NA	NA	NA	No	NA
ENSG00000158966	CACHD1	1	64936428	65158741	ENSG00000151065	DCP1B	12	2055220	2113677	0.0085107	8.2954	8.11E-08	0.01158585	0.0040959	0.75346	0.58362	1	No	NA
ENSG00000100307	CBX7	22	39516172	39548655	ENSG00000163064	EN1	2	119599747	119605254	0.0073845	11.987	8.12E-08	0.01159448	NA	NA	NA	NA	No	NA
ENSG00000099849	RASSF7	11	560404	564021	ENSG00000204420	C6orf25	6	31691121	31694491	0.0095043	6.6209	8.18E-08	0.01165731	0.0096433	1.2714	0.26132	1	Yes	No
ENSG00000118997	DNAH7	2	196602427	196933536	ENSG00000164938	TP53INP1	8	95938200	95961639	0.0073816	11.983	8.18E-08	0.01165731	0.0012238	0.28091	0.89042	1	No	NA
ENSG00000151948	GLT1D1	12	129337972	129469509	ENSG00000244057	LCE3C	1	152573138	152573562	0.010424	5.651	8.20E-08	0.01167955	NA	NA	NA	NA	No	NA
ENSG00000006125	AP2B1	17	33913918	34053436	ENSG00000149218	ENDOD1	11	94822974	94865809	0.0094989	6.6171	8.27E-08	0.01176284	0.015	2.3223	0.031251	1	Yes	Yes
ENSG00000139330	KERA	12	91444268	91451760	ENSG00000124783	SSR1	6	7268539	7347679	0.0067232	16.363	8.27E-08	0.01176284	0.0053831	2.4869	0.083726	1	No	NA
ENSG00000006125	AP2B1	17	33913918	34053436	ENSG00000172270	BSG	19	571297	583493	0.0094988	6.617	8.28E-08	0.01176284	0.0058628	0.89935	0.4946	1	No	NA
ENSG00000139330	KERA	12	91444268	91451760	ENSG00000112237	CCNC	6	99990256	100016849	0.0067181	16.351	8.37E-08	0.01189004	0.0027096	1.2485	0.28743	1	No	NA
ENSG00000009950	MLXIPL	7	73007524	73038873	ENSG00000149534	MS4A2	11	59855734	59863444	0.007955	9.6887	8.39E-08	0.01191147	0.0048994	0.75083	0.60884	1	No	NA
ENSG00000172057	ORMDL3	17	38077294	38083854	ENSG00000173281	PPP1R3B	8	8993765	9009084	0.0084946	8.2796	8.41E-08	0.01193357	0.016821	2.6091	0.016347	1	No	NA
ENSG00000139330	KERA	12	91444268	91451760	ENSG00000047932	GOPC	6	117881432	117923691	0.006713	16.338	8.48E-08	0.01201376	0.0011395	0.52421	0.5922	1	No	NA
ENSG00000139330	KERA	12	91444268	91451760	ENSG00000083896	YTHDC1	4	69176105	69215807	0.00671	16.331	8.54E-08	0.01209256	0.0016013	0.73698	0.47884	1	No	NA
ENSG00000163947	ARHGEF3	3	56761446	57113357	ENSG00000069966	GNB5	15	52416670	52483566	0.0099511	6.0671	8.56E-08	0.0121176	0.033423	3.9463	0.00013273	0.23307388	No	NA
ENSG00000107669	ATE1	10	123499939	123688316	ENSG00000170448	NFXL1	4	47849257	47913011	0.012904	4.2025	8.57E-08	0.0121176	0.028226	1.7544	0.036721	1	No	NA
ENSG00000023909	GCLM	1	94350761	94374966	ENSG00000139182	CLSTN3	12	7282294	7311541	0.010842	5.2907	8.60E-08	0.01215405	0.01476	1.3648	0.19175	1	No	NA
ENSG00000139330	KERA	12	91444268	91451760	ENSG00000152683	SLC30A6	2	32390933	32449448	0.006705	16.319	8.64E-08	0.01220753	0.0013685	0.62967	0.533	1	No	NA
ENSG00000172660	TAF15	17	34136459	34174246	ENSG00000100325	ASCC2	22	30184597	30234265	0.0089909	7.3049	8.67E-08	0.01222628	0.0070306	1.0798	0.37266	1	No	NA
ENSG00000141542	RAB40B	17	80612849	80656604	ENSG00000172322	CLEC12A	12	10124014	10138194	0.0094769	6.6016	8.68E-08	0.01222628	0.0014572	0.44655	0.7198	1	No	NA
ENSG00000166126	AMN	14	103388993	103397529	ENSG00000153157	SYCP2L	6	10887064	10979553	0.011267	4.9993	8.67E-08	0.01222628	0.0044188	0.50654	0.85198	1	No	NA
ENSG00000139330	KERA	12	91444268	91451760	ENSG00000165801	ARHGEF40	14	21538429	21557035	0.0067032	16.314	8.68E-08	0.01222628	0.00024965	0.11474	0.89161	1	No	NA
ENSG00000068745	IP6K2	3	48725436	48777786	ENSG00000120937	NPPB	1	11917521	11918988	0.0089903	7.3044	8.69E-08	0.01222628	NA	NA	NA	NA	No	NA
ENSG00000006125	AP2B1	17	33913918	34053436	ENSG00000118816	CCNI	4	77968311	77997158	0.0094739	6.5995	8.74E-08	0.01229244	0.002338	0.35737	0.90575	1	Yes	Yes
ENSG00000205045	SLFN12L	17	33800708	33864880	ENSG00000011105	TSPAN9	12	3186521	3395730	0.0094717	6.5979	8.78E-08	0.01234389	0.032284	4.356	9.20E-05	0.162012	Yes	Yes
ENSG00000174885	NLRP6	11	278365	285359	ENSG00000185245	GP1BA	17	4835592	4838325	0.0094706	6.5972	8.80E-08	0.0123638	0.021402	2.2162	0.019147	1	Yes	Yes
ENSG00000197980	LEKR1	3	156543270	156763918	ENSG00000135387	CAPRIN1	11	34073230	34122703	0.0094676	6.5951	8.86E-08	0.01243675	0.0081425	0.93689	0.4849	1	No	NA
ENSG00000172057	ORMDL3	17	38077294	38083854	ENSG00000113916	BCL6	3	187439165	187455732	0.0084707	8.256	8.88E-08	0.01246247	0.011653	1.798	0.096482	1	No	NA
ENSG00000092871	RFFL	17	33341759	33416338	ENSG00000238243	OR2W3	1	248058859	248060449	0.0089763	7.2928	8.96E-08	0.01256272	0.0073205	0.84162	0.566	1	Yes	No
ENSG00000161956	SENP3	17	7465192	7475287	ENSG00000178538	CA8	8	61099906	61193971	0.009459	6.589	9.03E-08	0.01264701	0.0095397	1.2576	0.26846	1	No	NA
ENSG00000139330	KERA	12	91444268	91451760	ENSG00000142892	PIGK	1	77554675	77685115	0.0066864	16.273	9.04E-08	0.01266063	0.00036687	0.16864	0.84484	1	No	NA
ENSG00000139330	KERA	12	91444268	91451760	ENSG00000141380	SS18	18	23596578	23671181	0.0066852	16.27	9.07E-08	0.01268878	0.0055724	2.5749	0.076713	1	No	NA
ENSG00000006125	AP2B1	17	33913918	34053436	ENSG00000035403	VCL	10	75757872	75879918	0.0094557	6.5867	9.09E-08	0.01271242	0.0030852	0.47195	0.82944	1	Yes	Yes
ENSG00000180376	CCDC66	3	56591189	56653929	ENSG00000120885	CLU	8	27454434	27472548	0.0084601	8.2456	9.10E-08	0.01271409	0.016292	3.0342	0.010061	1	Yes	No
ENSG00000134255	CEPT1	1	111682848	111727724	ENSG00000128310	GALR3	22	38219389	38221502	0.0084593	8.2448	9.12E-08	0.01272707	NA	NA	NA	NA	No	NA
ENSG00000100147	CCDC134	22	42196683	42222303	ENSG00000175556	LONRF3	X	118108581	118156888	0.0089635	7.2824	9.22E-08	0.01286196	0.011194	1.4782	0.17129	1	No	NA
ENSG00000185379	RAD51D	17	33426811	33447063	ENSG00000174175	SELP	1	169558087	169599431	0.0073308	11.9	9.23E-08	0.01286325	0.003868	1.1882	0.3131	1	No	NA

ENSG00000163947	ARHGEF3	3	56761446	57113357	ENSG00000158457	TSPAN33	7	128784712	128808637	0.009916	6.0455	9.23E-08	0.01286607	0.023037	2.6911	0.006277	1	No	NA
ENSG00000139330	KERA	12	91444268	91451760	ENSG00000143033	MTF2	1	93544792	93604638	0.0066773	16.251	9.25E-08	0.01287223	0.00033408	0.15356	0.85767	1	No	NA
ENSG00000174885	NLRP6	11	278365	285359	ENSG00000174175	SELP	1	169558087	169599431	0.0094471	6.5806	9.27E-08	0.01289271	0.011656	1.1951	0.29442	1	Yes	Yes
ENSG00000006125	AP2B1	17	33913918	34053436	ENSG00000035499	DEPDC1B	5	59892739	59996017	0.009444	6.5785	9.33E-08	0.01297086	0.010356	1.5959	0.14511	1	No	NA
ENSG00000139330	KERA	12	91444268	91451760	ENSG00000114416	FXR1	3	180585929	180700541	0.0066687	16.23	9.44E-08	0.01311963	0.00046835	0.21531	0.80634	1	No	NA
ENSG00000156931	VPS8	3	184529931	184770402	ENSG00000164512	ANKRD55	5	55395507	55529186	0.007902	9.6236	9.49E-08	0.01317305	0.012089	2.2418	0.048314	1	No	NA
ENSG00000171522	PTGER4	5	40679600	40693837	ENSG00000144820	GPR128	3	100328433	100414323	0.0079018	9.6234	9.49E-08	0.01317305	NA	NA	NA	NA	No	NA
ENSG00000157500	APPL1	3	57261765	57301820	ENSG00000259207	ITGB3	17	45387505	45389182	0.0079014	9.6229	9.50E-08	0.01317565	0.015751	4.8969	0.0022106	1	Yes	No
ENSG00000239388	ASB14	3	57310556	57326710	ENSG00000174175	SELP	1	169558087	169599431	0.0084373	8.2232	9.58E-08	0.01327704	0.014952	2.7808	0.016779	1	Yes	No
ENSG00000108306	FBXL20	17	37415384	37558776	ENSG00000113916	BCL6	3	187439165	187455732	0.0084366	8.2225	9.60E-08	0.01329119	0.0028246	0.51894	0.76209	1	No	NA
ENSG00000272325	NUDT3	6	34360041	34360451	ENSG00000171735	CAMTA1	1	6845384	7829766	0.0084356	8.2215	9.62E-08	0.01331156	0.0035979	0.66151	0.65277	1	No	NA
ENSG00000073711	PPP2R3A	3	135684515	135866733	ENSG00000159147	DONSON	21	34949842	34961014	0.0084338	8.2197	9.66E-08	0.01335747	0.0023371	0.42916	0.82848	1	No	NA
ENSG00000213171	LINGO4	1	151772740	151775193	ENSG00000259207	ITGB3	17	45387505	45389182	0.0089415	7.2644	9.68E-08	0.01337956	0.01197	1.8476	0.087055	1	No	NA
ENSG00000139330	KERA	12	91444268	91451760	ENSG00000085491	SLC25A24	1	108676658	108743471	0.0066576	16.203	9.70E-08	0.01339596	0.0078254	3.6241	0.027054	1	No	NA
ENSG00000139330	KERA	12	91444268	91451760	ENSG00000163608	C3orf17	3	112721287	112738708	0.0066559	16.199	9.74E-08	0.01343371	0.0038349	1.7689	0.17109	1	No	NA
ENSG00000015532	XYLT2	17	48423453	48440499	ENSG00000102882	MAPK3	16	30125426	30134827	0.0094239	6.5644	9.75E-08	0.01343371	0.0054386	0.83393	0.54371	1	No	NA
ENSG00000132139	GAS2L2	17	34071530	34079897	ENSG00000172270	BSG	19	571297	583493	0.0098911	6.0301	9.74E-08	0.01343371	0.004993	0.57268	0.80087	1	No	NA
ENSG00000139330	KERA	12	91444268	91451760	ENSG00000146282	RARS2	6	88224096	88299721	0.0066535	16.193	9.80E-08	0.01349451	0.0034246	1.579	0.20674	1	No	NA
ENSG00000205045	SLFN12L	17	33800708	33864880	ENSG00000148341	SH3GLB2	9	131771385	131790582	0.0094186	6.5606	9.86E-08	0.01357188	0.0072217	0.94981	0.46701	1	Yes	Yes
ENSG00000112139	MDGA1	6	37598455	37667082	ENSG00000049247	UTS2	1	7906752	7913572	0.0066494	16.183	9.90E-08	0.01361023	0.00044076	0.20262	0.81663	1	No	NA
ENSG00000139330	KERA	12	91444268	91451760	ENSG00000118007	STAG1	3	136057212	136471220	0.0066491	16.182	9.90E-08	0.01361058	0.000314	0.14433	0.86562	1	No	NA
ENSG00000114395	CYB561D2	3	50389440	50391500	ENSG00000103023	PRSS54	16	58318309	58328951	0.0073005	11.85	9.91E-08	0.01361795	NA	NA	NA	NA	No	NA
ENSG00000139330	KERA	12	91444268	91451760	ENSG00000116001	TIA1	2	70436576	70463307	0.0066477	16.178	9.94E-08	0.01364053	0.00012606	0.057932	0.94372	1	No	NA
ENSG00000139330	KERA	12	91444268	91451760	ENSG00000069275	NUCKS1	1	205688655	205719404	0.0066461	16.174	9.98E-08	0.01368381	0.0010711	0.4927	0.61114	1	No	NA
ENSG00000197724	PHF2	9	96338689	96441869	ENSG00000150269	OR5M9	11	56229945	56230877	0.009413	6.5567	9.98E-08	0.01368562	NA	NA	NA	NA	No	NA
ENSG00000198719	DLL1	6	170591294	170599561	ENSG00000155269	GPR78	4	8560452	8589477	0.010329	5.5989	1.00E-07	0.01373647	NA	NA	NA	NA	No	NA
ENSG00000172716	SLFN11	17	33677324	33700720	ENSG00000103257	SLC7A5	16	87863629	87903094	0.011611	4.7232	1.01E-07	0.01384367	0.018272	1.4098	0.15515	1	Yes	No
ENSG00000139330	KERA	12	91444268	91451760	ENSG00000122068	FYTTD1	3	197464050	197511317	0.0066392	16.157	1.01E-07	0.01387957	0.00018923	0.086967	0.91671	1	No	NA
ENSG00000008838	MED24	17	38175350	38217468	ENSG00000135842	FAM129A	1	184759858	184943682	0.0078695	9.5837	1.02E-07	0.01398789	0.014187	3.2991	0.010721	1	Yes	No
ENSG00000172057	ORMDL3	17	38077294	38083854	ENSG00000128512	DOCK4	7	111366166	111846466	0.0084077	8.1941	1.02E-07	0.01400043	0.0053349	0.81794	0.55603	1	No	NA
ENSG00000132382	MYBBP1A	17	4443643	4458926	ENSG00000137101	CD72	9	35610599	35646807	0.0084073	8.1937	1.03E-07	0.01400476	0.013435	1.5542	0.13463	1	No	NA
ENSG00000189241	TSPYL1	6	116597741	116601066	ENSG00000003393	ALS2	2	202565277	202645912	0.011184	4.9622	1.03E-07	0.01402954	0.0088572	0.90555	0.51962	1	No	NA
ENSG00000177096	FAM109B	22	42470255	42475445	ENSG00000114933	INO80D	2	206858445	206928408	0.0093986	6.5466	1.03E-07	0.01404612	0.0084056	1.1068	0.35642	1	No	NA
ENSG00000186075	ZPBP2	17	38024417	38034149	ENSG00000151726	ACSL1	4	185676749	185747972	0.0058384	28.4	1.03E-07	0.01405313	0.00091958	0.84679	0.3577	1	Yes	No
ENSG00000129282	MRM1	17	34958001	34965407	ENSG00000079308	TNS1	2	218664512	218867718	0.0089096	7.2382	1.04E-07	0.01415273	0.009081	1.3975	0.21254	1	No	NA
ENSG00000139330	KERA	12	91444268	91451760	ENSG00000152700	SAR1B	5	133936834	133968678	0.0066276	16.129	1.04E-07	0.01419913	0.0032836	1.5138	0.22062	1	No	NA
ENSG00000214530	STARD10	11	72465774	72501435	ENSG00000038427	VCAN	5	82767284	82878122	0.012403	4.3265	1.05E-07	0.01431074	0.00871	2.0143	0.090458	1	No	NA
ENSG00000142102	ATHL1	11	289138	296107	ENSG00000095303	PTGS1	9	125132824	125157982	0.014326	3.6855	1.05E-07	0.01432979	0.00080099	0.36835	0.69198	1	Yes	Yes
ENSG00000172123	SLFN12	17	33738079	33760302	ENSG00000204463	BAG6	6	31606805	31620170	0.012796	4.1667	1.06E-07	0.01437596	0.023628	3.6904	0.0012485	1	Yes	No
ENSG00000181744	C3orf58	3	143690640	143767561	ENSG00000134824	FADS2	11	61588853	61634826	0.0089013	7.2314	1.06E-07	0.01437596	0.0058626	0.89933	0.49462	1	No	NA
ENSG00000137776	SLTM	15	59171244	59225852	ENSG00000169313	P2RY12	3	151055168	151102600	0.0088981	7.2287	1.07E-07	0.0144641	0.0027072	0.41397	0.87004	1	No	NA
ENSG00000087589	CASS4	20	54987168	55034396	ENSG00000198467	TPM2	9	35681989	35691017	0.0078511	9.5612	1.07E-07	0.01446945	0.0012267	0.37582	0.77047	1	No	NA
ENSG00000139437	TCHP	12	110338079	110355874	ENSG00000133574	GIMAP4	7	150264365	150271041	0.0093818	6.5347	1.07E-07	0.01447614	0.0027456	0.41986	0.86609	1	No	NA
ENSG00000177096	FAM109B	22	42470255	42475445	ENSG00000182264	IZUMO1	19	49244145	49250166	0.0093784	6.5324	1.08E-07	0.01457352	NA	NA	NA	NA	No	NA
ENSG00000172660	TAF15	17	34136459	34174246	ENSG00000143774	GUK1	1	228327663	228336685	0.0088923	7.224	1.08E-07	0.01459198	0.013334	2.061	0.055402	1	Yes	No
ENSG00000108306	FBXL20	17	37415384	37558776	ENSG00000072952	MRVI1	11	10594638	10715535	0.0083845	8.1713	1.08E-07	0.01459198	0.012282	2.278	0.045068	1	No	NA
ENSG00000139437	TCHP	12	110338079	110355874	ENSG00000079785	DDX1	2	15731302	15771235	0.0093773	6.5316	1.08E-07	0.01459198	0.0076748	1.1795	0.31488	1	No	NA
ENSG00000151327	FAM177A1	14	35514113	35582336	ENSG00000135929	CYP27A1	2	219646479	219680016	0.011159	4.9511	1.08E-07	0.01460397	0.02169	1.8341	0.044618	1	Yes	Yes
ENSG00000100216	TOMM22	22	39077953	39080818	ENSG00000163064	EN1	2	119599747	119605254	0.0088906	7.2227	1.08E-07	0.01462674	NA	NA	NA	NA	No	NA
ENSG00000137310	TCF19	6	31126319	31131992	ENSG00000101425	BPI	20	36888551	36965907	0.0093713	6.5273	1.09E-07	0.01473416	0.015213	2.017	0.050309	1	Yes	No
ENSG00000167914	GSDMA	17	38119226	38134019	ENSG00000125538	IL1B	2	113587328	113594480	0.0066084	16.082	1.09E-07	0.01473416	0.010589	4.9177	0.0075096	1	Yes	Yes
ENSG00000186075	ZPBP2	17	38024417	38034149	ENSG00000072952	MRVI1	11	10594638	10715535	0.0058116	28.269	1.10E-07	0.0148551	0.0016782	1.5466	0.21396	1	Yes	No
ENSG00000073598	FNDC8	17	33448598	33454436	ENSG00000100614	PPM1A	14	60712470	60765805	0.0072549	11.776	1.10E-07	0.01486014	0.013846	4.2963	0.0050753	1	No	NA
ENSG00000139330	KERA	12	91444268	91451760	ENSG00000145388	METTL14	4	119606523	119633806	0.0065978	16.056	1.12E-07	0.01508708	0.0013975	0.64305	0.52593	1	No	NA
ENSG00000163958	ZDHHC19	3	195924320	195938308	ENSG00000204385	SLC44A4	6	31830969	31846823	0.0098249	5.9894	1.12E-07	0.01509465	NA	NA	NA	NA	No	NA
ENSG00000101276	SLC52A3	20	740724	749131	ENSG00000256660	CLEC12B	12	10165384	10171218	0.0098212	5.9871	1.13E-07	0.01520429	0.0056618	0.577	0.81671	1	No	NA
ENSG00000139330	KERA	12	91444268	91451760	ENSG00000146963	C7orf55-LUC	7	139044654	139059217	0.0065922	16.042	1.14E-07	0.01526278	NA	NA	NA	NA	No	NA
ENSG00000139330	KERA	12	91444268	91451760	ENSG00000145833	DDX46	5	134094469	134169313	0.00659	16.037	1.14E-07	0.01533595	0.0016798	0.77314	0.46186	1	No	NA
ENSG00000198917	C9orf114	9	131586026	131592100	ENSG00000179869	ABCA13	7	48211055	48687092	0.0078209	9.524	1.15E-07	0.01534737	0.015737	4.8925	0.0022242	1	Yes	No
ENSG00000015532	XYLT2	17	48423453	48440499	ENSG00000103197	TSC2	16	2098203	2138326	0.0093479	6.5109	1.15E-07	0.01541503	0.01037	1.598	0.14449	1	No	NA
ENSG00000139330	KERA	12	91444268	91451760	ENSG00000109445	ZNF330	4	142142041	142155851	0.0065855	16.026	1.16E-07	0.01547457	0.0071715	3.3191	0.03662	1	No	NA
ENSG00000139437	TCHP	12	110338079	110355874	ENSG00000084733	RAB10	2	26256979	26360323	0.0093451	6.509	1.16E-07	0.01548721	0.0059724	0.91626	0.48229	1	No	NA
ENSG00000172660	TAF15	17	34136459	34174246	ENSG00000137193	PIM1	6	37137979	37143202	0.0088579	7.1958	1.17E-07	0.01558807	0.016303	2.5275	0.019694	1	No	NA
ENSG00000139330	KERA	12	91444268	91451760	ENSG00000164074	C4orf29	4	128904094	128960866	0.0065803	16.013	1.17E-07	0.0156407	0.00025822	0.11868	0.8881	1	No	NA
ENSG00000139330	KERA	12	91444268	91451760	ENSG00000136709	WDR33	2	128466251	128568761	0.00658	16.013	1.17E-07	0.01564252	0.00083463	0.38383	0.68135	1	No	NA
ENSG00000205045	SLFN12L	17	33800708	33864880	ENSG00000184792	OSBP2	22	31089769	31303811	0.0093384	6.5043	1.17E-07	0.01565332	0.012012	1.5875	0.13537	1	Yes	Yes
ENSG00000163606	CD200R1	3	112640056	112693969	ENSG00000270898	ASB3	2	54047280	54047381	0.011119	4.9331	1.17E-07	0.01565332	0.012866	1.1874	0.29532	1	No	NA

ENSG00000163606	CD200R1	3	112640056	112693969	ENSG00000119737	GPR75	2	54080050	54082002	0.011119	4.9331	1.17E-07	0.01565332	0.0096432	0.88705	0.54484	1	No	NA
ENSG00000108278	ZNHIT3	17	34842473	34849850	ENSG00000022840	RNF10	12	120971283	121015397	0.0083456	8.1331	1.18E-07	0.01569953	0.012039	2.2324	0.049193	1	Yes	No
ENSG00000141150	RASL10B	17	34058668	34070540	ENSG00000128274	A4GALT	22	43088127	43117304	0.009802	5.9753	1.18E-07	0.01569953	0.014095	1.8667	0.071868	1	No	NA
ENSG00000228008	CTD-2330K9.3	3	49943495	49954370	ENSG00000175691	ZNF77	19	2933216	2944969	0.0072264	11.729	1.18E-07	0.01571195	0.0037812	0.87012	0.48128	1	Yes	No
ENSG00000130584	ZBTB46	20	62375019	62462597	ENSG00000134755	DSC2	18	28645940	28682378	0.0088504	7.1897	1.19E-07	0.01575626	0.0055019	1.0135	0.40837	1	No	NA
ENSG00000164167	LSM6	4	147096837	147121152	ENSG00000006740	ARHGAP44	17	12692856	12894960	0.011114	4.9307	1.19E-07	0.01576372	0.011256	0.94177	0.49905	1	No	NA
ENSG00000139330	KERA	12	91444268	91451760	ENSG00000115970	THADA	2	43393800	43823185	0.0065746	15.999	1.19E-07	0.01576372	0.00054206	0.24921	0.77947	1	No	NA
ENSG00000177096	FAM109B	22	42470255	42475445	ENSG00000177192	PUS1	12	132416060	132428406	0.009332	6.4997	1.19E-07	0.01580393	0.010863	1.4339	0.188	1	No	NA
ENSG00000115386	REG1A	2	79347584	79350545	ENSG00000002933	TMEM176A	7	150498622	150502208	0.0093294	6.4979	1.20E-07	0.01588252	0.0051683	0.67833	0.69056	1	No	NA
ENSG00000177951	BET1L	11	167784	207428	ENSG00000120885	CLU	8	27454434	27472548	0.009794	5.9704	1.20E-07	0.01590008	0.031343	2.6768	0.0021733	1	Yes	Yes
ENSG00000141736	ERBB2	17	37844949	37884915	ENSG00000128512	DOCK4	7	111366166	111846466	0.0093277	6.4967	1.20E-07	0.01592158	0.0067232	0.6859	0.7222	1	No	NA
ENSG00000188895	MSL1	17	38278551	38291643	ENSG00000162551	ALPL	1	21835858	21904905	0.011107	4.9277	1.20E-07	0.01593115	0.014447	4.4856	0.0039078	1	Yes	No
ENSG00000163946	FAM208A	3	56658507	56717265	ENSG00000173210	ABLIM3	5	148521046	148640105	0.0097907	5.9684	1.21E-07	0.01598037	0.039116	5.3154	5.66E-06	0.01023894	Yes	No
ENSG00000177666	PNPLA2	11	818902	825573	ENSG00000122786	CALD1	7	134429003	134655479	0.0083324	8.1201	1.22E-07	0.01604055	0.010075	1.5521	0.15814	1	Yes	No
ENSG00000205045	SLFN12L	17	33800708	33864880	ENSG00000105221	AKT2	19	40736224	40791443	0.0093229	6.4934	1.22E-07	0.01604055	0.01373	1.8177	0.080547	1	No	NA
ENSG00000139330	KERA	12	91444268	91451760	ENSG00000164941	INTS8	8	95825539	95888717	0.0065653	15.977	1.21E-07	0.01604055	0.0022673	1.0442	0.3524	1	No	NA
ENSG00000151468	CCDC3	10	12938627	13141309	ENSG00000213471	TTLL13	15	90798987	90805901	0.012725	4.1433	1.22E-07	0.01604233	0.015376	1.0117	0.43884	1	No	NA
ENSG00000139330	KERA	12	91444268	91451760	ENSG00000122507	BBS9	7	33168856	33645680	0.0065643	15.974	1.22E-07	0.01604233	0.0015433	0.71025	0.49179	1	No	NA
ENSG00000154760	SLFN13	17	33762115	33775856	ENSG00000171552	BCL2L1	20	30252255	30311792	0.011101	4.9251	1.22E-07	0.01604651	0.0078859	0.6021	0.84162	1	Yes	No
ENSG00000164442	CITED2	6	139693393	139695757	ENSG00000187017	ESPN	1	6484848	6521040	0.0072098	11.702	1.23E-07	0.01617836	0.028178	8.8725	8.44E-06	0.01520888	Yes	No
ENSG00000131844	MCCC2	5	70883115	70954531	ENSG00000137261	KIAA0319	6	24544332	24646383	0.0097821	5.9631	1.23E-07	0.01619428	0.01327	1.5349	0.14094	1	No	NA
ENSG00000182179	UBA7	3	49842640	49851379	ENSG00000147488	ST18	8	53023399	53322505	0.0077878	9.4834	1.24E-07	0.01624963	NA	NA	NA	NA	No	NA
ENSG00000118495	PLAGL1	6	144261437	144385735	ENSG00000128512	DOCK4	7	111366166	111846466	0.0083229	8.1108	1.24E-07	0.01631283	0.015962	2.9717	0.011423	1	No	NA
ENSG00000112305	SMAP1	6	71377479	71562367	ENSG00000078114	NEBL	10	21068902	21463116	0.0083227	8.1105	1.24E-07	0.01631283	0.0061674	1.8989	0.12814	1	No	NA
ENSG00000128581	RABL5	7	100956975	100965104	ENSG00000197893	NRAP	10	115349425	115423886	0.010227	5.5432	1.24E-07	0.0163142	NA	NA	NA	NA	No	NA
ENSG00000139330	KERA	12	91444268	91451760	ENSG00000110075	PPP6R3	11	68228186	68377491	0.0065552	15.952	1.24E-07	0.01631687	0.0025312	1.166	0.31206	1	No	NA
ENSG00000099834	CDHR5	11	616565	626078	ENSG00000144677	CTDSPL	3	37903451	38025960	0.0088228	7.1671	1.26E-07	0.0165161	0.0034665	0.53047	0.78539	1	Yes	No
ENSG00000167914	GSDMA	17	38119226	38134019	ENSG00000128512	DOCK4	7	111366166	111846466	0.006548	15.934	1.27E-07	0.01658674	0.003871	1.7857	0.16827	1	No	NA
ENSG00000139330	KERA	12	91444268	91451760	ENSG00000144048	DUSP11	2	73989311	74007284	0.0065443	15.925	1.28E-07	0.01672272	0.00021971	0.10098	0.90396	1	No	NA
ENSG00000168374	ARF4	3	57557090	57583947	ENSG00000154146	NRGN	11	124609829	124615878	0.0083099	8.0979	1.28E-07	0.01672905	0.02271	4.2572	0.00078341	1	Yes	No
ENSG00000172057	ORMDL3	17	38077294	38083854	ENSG00000183762	KREMEN1	22	29469066	29564321	0.0083095	8.0975	1.28E-07	0.01673276	0.0039575	0.60592	0.72576	1	No	NA
ENSG00000154768	C17orf50	17	34087916	34092098	ENSG00000103148	NPRL3	16	138697	188859	0.0088151	7.1607	1.28E-07	0.01673755	0.0086231	1.1357	0.33815	1	Yes	No
ENSG00000128578	STRIP2	7	129074274	129128240	ENSG00000135314	KHDC1	6	73951037	74020088	0.010213	5.5353	1.28E-07	0.01673755	0.014808	1.5231	0.13501	1	No	NA
ENSG00000156885	COX6A2	16	31439052	31439749	ENSG00000130511	SSBP4	19	18529674	18544652	0.0077683	9.4595	1.29E-07	0.0168691	0.0083679	1.5459	0.17297	1	No	NA
ENSG00000117461	PIK3R3	1	46505812	46642160	ENSG00000204188	GGNBP1	6	33551515	33556803	0.0077676	9.4587	1.30E-07	0.01688574	NA	NA	NA	NA	No	NA
ENSG00000139330	KERA	12	91444268	91451760	ENSG00000127980	PEX1	7	92116334	92157845	0.0065379	15.909	1.30E-07	0.01691019	0.0043072	1.9877	0.1376	1	No	NA
ENSG00000088826	SMOX	20	4101627	4168394	ENSG00000148908	RGS10	10	121259340	121302220	0.010204	5.5305	1.31E-07	0.01698761	0.0078945	0.80634	0.61048	1	No	NA
ENSG00000177096	FAM109B	22	42470255	42475445	ENSG00000128564	VGF	7	100805790	100808874	0.0092901	6.4703	1.31E-07	0.01698761	NA	NA	NA	NA	No	NA
ENSG00000102780	DGKH	13	42614176	42830714	ENSG00000168903	BTNL3	5	180415845	180433727	0.0088056	7.153	1.31E-07	0.01701805	0.0093792	1.7345	0.12405	1	No	NA
ENSG00000102780	DGKH	13	42614176	42830714	ENSG00000113303	BTNL8	5	180326077	180377906	0.0088056	7.153	1.31E-07	0.01701805	0.003973	0.73076	0.60045	1	No	NA
ENSG00000005187	ACSM3	16	20621565	20807446	ENSG00000143344	RGL1	1	183605208	183897666	0.0071824	11.657	1.31E-07	0.01701996	0.0022411	0.68732	0.55989	1	No	NA
ENSG00000088826	SMOX	20	4101627	4168394	ENSG00000101782	RIOK3	18	21032787	21066567	0.010202	5.529	1.31E-07	0.01701996	0.0084231	0.86079	0.56014	1	No	NA
ENSG00000159692	CTBP1	4	1205236	1243741	ENSG00000266967	AARSD1	17	41114192	41116515	0.0092872	6.4682	1.31E-07	0.01701996	0.0029514	0.38651	0.91056	1	No	NA
ENSG00000159692	CTBP1	4	1205236	1243741	ENSG00000267060	PTGES3L	17	41120105	41121203	0.0092872	6.4682	1.31E-07	0.01701996	NA	NA	NA	NA	No	NA
ENSG00000159692	CTBP1	4	1205236	1243741	ENSG00000108825	PTGES3L-AAI	17	41131922	41132020	0.0092872	6.4682	1.31E-07	0.01701996	NA	NA	NA	NA	No	NA
ENSG00000139330	KERA	12	91444268	91451760	ENSG00000157869	RAB28	4	13362978	13485989	0.0065309	15.892	1.32E-07	0.01709466	0.00072152	0.33178	0.71773	1	No	NA
ENSG00000127561	SYNGR3	16	2039661	2044276	ENSG00000049323	LTBP1	2	33172039	33624576	0.0097458	5.9407	1.33E-07	0.01721972	0.014455	1.2134	0.27319	1	No	NA
ENSG00000158856	DMTN	8	21906506	21940038	ENSG00000116688	MFN2	1	12040238	12073571	0.011474	4.6669	1.33E-07	0.01725023	0.0092158	1.4185	0.20435	1	No	NA
ENSG00000185436	IFNLR1	1	24480647	24514449	ENSG00000101335	MYL9	20	35169887	35178228	0.0087968	7.1457	1.34E-07	0.01725616	0.0097197	1.1202	0.34681	1	No	NA
ENSG00000108733	PEX12	17	33901814	33905882	ENSG00000085733	CTTN	11	70244510	70282690	0.0082878	8.0762	1.34E-07	0.01736409	0.012633	2.3439	0.039688	1	Yes	No
ENSG00000108733	PEX12	17	33901814	33905882	ENSG00000112146	FBXO9	6	52916789	52965671	0.0082873	8.0757	1.35E-07	0.01736932	0.0053048	0.97702	0.43069	1	No	NA
ENSG00000132326	PER2	2	239152679	239198743	ENSG00000088854	C20orf194	20	3229951	3388272	0.0077507	9.4379	1.35E-07	0.01736932	0.0013762	0.31592	0.86742	1	No	NA
ENSG00000074706	IPCEF1	6	154475631	154677926	ENSG00000104522	TSTA3	8	144694788	144700218	0.0092751	6.4597	1.35E-07	0.01738675	0.0082618	1.0877	0.36886	1	No	NA
ENSG00000139330	KERA	12	91444268	91451760	ENSG00000175054	ATR	3	142168077	142297668	0.0065217	15.87	1.35E-07	0.01739386	0.0001843	0.084701	0.91879	1	No	NA
ENSG00000173421	CCDC36	3	49235861	49295537	ENSG00000131059	BPIFA3	20	31805116	31815564	0.0077492	9.4361	1.35E-07	0.01739838	NA	NA	NA	NA	No	NA
ENSG00000006125	AP2B1	17	33913918	34053436	ENSG00000187017	ESPN	1	6484848	6521040	0.0092738	6.4588	1.35E-07	0.01740033	0.0046699	0.7155	0.63718	1	Yes	Yes
ENSG00000141150	RASL10B	17	34058668	34070540	ENSG00000187017	ESPN	1	6484848	6521040	0.0097292	5.9305	1.38E-07	0.01770791	0.0047616	0.6247	0.73578	1	Yes	No
ENSG00000132139	GAS2L2	17	34071530	34079897	ENSG00000198959	TGM2	20	36756863	36794980	0.0097295	5.9307	1.38E-07	0.01770791	0.0074489	0.85649	0.55304	1	Yes	No
ENSG00000148426	PROSER2	10	11865338	11914276	ENSG00000171103	TRMT61B	2	29073938	29093167	0.0097294	5.9306	1.38E-07	0.01770791	0.0059101	0.60245	0.79564	1	No	NA
ENSG00000197372	ZNF675	19	23708433	23870022	ENSG00000145476	CYP4V2	4	187112674	187134610	0.010177	5.5154	1.38E-07	0.01773409	0.013787	1.2736	0.24076	1	No	NA
ENSG00000139330	KERA	12	91444268	91451760	ENSG00000113643	RARS	5	167913450	167946304	0.0065118	15.845	1.38E-07	0.01773409	0.0022301	1.027	0.35849	1	No	NA
ENSG00000162739	SLAMF6	1	160454820	160493052	ENSG00000002549	LAP3	4	17578815	17609595	0.0065114	15.844	1.38E-07	0.01773409	0.0013141	0.60463	0.5465	1	No	NA
ENSG00000139330	KERA	12	91444268	91451760	ENSG00000134030	CTIF	18	46065417	46389588	0.0065114	15.844	1.38E-07	0.01773409	NA	NA	NA	NA	No	NA
ENSG00000172660	TAF15	17	34136459	34174246	ENSG00000130227	XPO7	8	21777180	21864096	0.0087802	7.1321	1.39E-07	0.01773709	0.0081755	1.257	0.27479	1	No	NA
ENSG00000239388	ASB14	3	57310556	57326710	ENSG00000049323	LTBP1	2	33172039	33624576	0.0082742	8.0629	1.39E-07	0.01773797	0.0050775	0.93495	0.4574	1	Yes	No
ENSG00000154760	SLFN13	17	33762115	33775856	ENSG00000135924	DNAJB2	2	220143989	220151622	0.011038	4.8967	1.39E-07	0.01773797	0.016154	1.2438	0.24778	1	Yes	No
ENSG00000139330	KERA	12	91444268	91451760	ENSG00000144895	EIF2A	3	150270141	150302029	0.0065075	15.835	1.40E-07	0.01785852	0.0076715	3.5523	0.029053	1	No	NA

ENSG00000072858	SIDT1	3	113251143	113348425	ENSG00000160307	S100B	21	48018875	48025121	0.0082704	8.0592	1.40E-07	0.01785889	0.0019889	0.36508	0.87258	1	No	NA
ENSG00000004059	ARF5	7	127228399	127231142	ENSG00000166197	NOLC1	10	103911933	103923627	0.008773	7.1262	1.41E-07	0.01796772	0.010764	1.6594	0.12788	1	No	NA
ENSG00000140398	NEIL1	15	75639296	75647159	ENSG00000131634	TMEM204	16	1578689	1605581	0.007151	11.606	1.41E-07	0.0180152	0.00098201	0.30079	0.82484	1	No	NA
ENSG00000205045	SLFN12L	17	33800708	33864880	ENSG00000159335	PTMS	12	6874682	6880116	0.0092526	6.4439	1.42E-07	0.01805371	0.01037	1.3682	0.21533	1	Yes	Yes
ENSG00000139330	KERA	12	91444268	91451760	ENSG00000205765	C5orf51	5	41904290	41921738	0.006501	15.819	1.42E-07	0.01808452	0.0034959	1.612	0.20005	1	No	NA
ENSG00000101446	SPINT3	20	44141101	44144264	ENSG00000143106	PSMA5	1	109941653	109969062	0.0064989	15.814	1.43E-07	0.01816876	0.00049165	0.45254	0.5013	1	No	NA
ENSG00000175768	TOMM5	9	37582643	37586163	ENSG00000106565	TMEM176B	7	150488373	150498448	0.0071455	11.597	1.43E-07	0.01820582	0.0031889	0.97892	0.40197	1	No	NA
ENSG00000055950	MRPL43	10	102741133	102747043	ENSG00000171617	ENC1	5	73923234	73933526	0.009711	5.9193	1.43E-07	0.0182314	0.012057	1.3928	0.19549	1	No	NA
ENSG00000186075	ZPBP2	17	38024417	38034149	ENSG00000128512	DOCK4	7	111366166	111846466	0.0057065	27.755	1.44E-07	0.0182544	0.00068593	0.63149	0.42701	1	No	NA
ENSG00000163946	FAM208A	3	56658507	56717265	ENSG00000151693	ASAP2	2	9346894	9541525	0.0097089	5.918	1.44E-07	0.01829007	0.013762	1.822	0.079752	1	Yes	No
ENSG00000015532	XYLT2	17	48423453	48440499	ENSG00000173039	RELA	11	65421067	65430565	0.0092425	6.4368	1.45E-07	0.01836382	0.0045867	0.70269	0.64752	1	No	NA
ENSG00000010256	UQCRC1	3	48636436	48648409	ENSG00000134595	SOX3	X	139585152	139587225	0.0071407	11.589	1.45E-07	0.01836382	NA	NA	NA	NA	No	NA
ENSG00000196923	PDLIM7	5	176910395	176924602	ENSG00000000003	TSPAN6	X	99883667	99894988	0.0077189	9.3989	1.45E-07	0.01837274	0.0067087	1.2373	0.28947	1	No	NA
ENSG00000205045	SLFN12L	17	33800708	33864880	ENSG00000136840	ST6GALNAC4	9	130670165	130679317	0.0092401	6.4352	1.46E-07	0.01842473	0.018652	2.4818	0.015816	1	Yes	Yes
ENSG00000205045	SLFN12L	17	33800708	33864880	ENSG00000160408	ST6GALNAC6	9	130647600	130667687	0.0092401	6.4352	1.46E-07	0.01842473	0.0042496	0.55724	0.79088	1	No	NA
ENSG00000096654	ZNF184	6	27418522	27440897	ENSG00000049247	UTS2	1	7906752	7913572	0.0097014	5.9134	1.46E-07	0.01851836	0.0085473	1.1257	0.34445	1	No	NA
ENSG00000139330	KERA	12	91444268	91451760	ENSG00000196776	CD47	3	107762145	107809872	0.0064875	15.786	1.47E-07	0.01855372	0.0010546	0.4851	0.6158	1	No	NA
ENSG00000139330	KERA	12	91444268	91451760	ENSG00000121892	PDS5A	4	39824483	39979576	0.0064866	15.784	1.47E-07	0.01858019	0.00052233	0.24014	0.78657	1	No	NA
ENSG00000124299	PEPD	19	33877861	34012700	ENSG00000157551	KCNJ15	21	39628663	39673748	0.0092306	6.4284	1.49E-07	0.01876573	0.0085112	1.1209	0.34747	1	No	NA
ENSG00000157870	FAM213B	1	2517930	2520479	ENSG00000091181	IL5RA	3	3111233	3168297	0.0096914	5.9072	1.50E-07	0.0188637	0.018013	1.8588	0.054637	1	No	NA
ENSG00000178363	CALML3	10	5566924	5568225	ENSG00000145244	CORIN	4	47596015	47840123	0.0092277	6.4264	1.50E-07	0.0188637	0.005606	0.73611	0.64138	1	No	NA
ENSG00000139330	KERA	12	91444268	91451760	ENSG00000211460	TSN	2	122494679	122525429	0.0064762	15.758	1.51E-07	0.01901218	0.0055181	2.5496	0.078664	1	No	NA
ENSG00000079999	KEAP1	19	10596796	10614417	ENSG00000237541	HLA-DQA2	6	32709119	32714992	0.0082362	8.0256	1.51E-07	0.01903576	0.0029187	0.53627	0.7489	1	No	NA
ENSG00000108733	PEX12	17	33901814	33905882	ENSG00000035499	DEPDC1B	5	59892739	59996017	0.0082354	8.0248	1.51E-07	0.0190568	0.0046023	0.84705	0.51647	1	No	NA
ENSG00000174885	NLRP6	11	278365	285359	ENSG00000204420	C6orf25	6	31691121	31694491	0.0092213	6.4219	1.52E-07	0.01907578	0.022521	2.3347	0.013317	1	Yes	Yes
ENSG00000177096	FAM109B	22	42470255	42475445	ENSG00000080573	COL5A3	19	10070237	10121147	0.0092208	6.4216	1.52E-07	0.01907578	0.012216	1.6148	0.12748	1	No	NA
ENSG00000177096	FAM109B	22	42470255	42475445	ENSG00000130635	COL5A1	9	137533620	137736686	0.0092211	6.4217	1.52E-07	0.01907578	0.0075529	0.9937	0.43427	1	No	NA
ENSG00000108733	PEX12	17	33901814	33905882	ENSG00000177666	PNPLA2	11	818902	825573	0.0082332	8.0226	1.52E-07	0.01910136	0.0024098	0.44255	0.81885	1	Yes	No
ENSG00000100883	SRP54	14	35451163	35498773	ENSG00000124102	PI3	20	43803517	43805185	0.010132	5.4907	1.52E-07	0.01910136	0.016436	1.6934	0.086307	1	No	NA
ENSG00000180479	ZNF571	19	38045684	38085673	ENSG00000111291	GPRC5D	12	13093709	13105081	0.0096817	5.9013	1.53E-07	0.01914737	0.015868	1.8401	0.066284	1	No	NA
ENSG00000139330	KERA	12	91444268	91451760	ENSG00000163743	RCHY1	4	76404247	76434506	0.0064684	15.739	1.54E-07	0.01923699	0.0026337	1.2134	0.29767	1	No	NA
ENSG00000139330	KERA	12	91444268	91451760	ENSG00000079335	CDC14A	1	100817290	100985833	0.0064686	15.74	1.54E-07	0.01923699	0.0002063	0.094813	0.90955	1	No	NA
ENSG00000197747	S100A10	1	151955391	151966866	ENSG00000153317	ASAP1	8	131064353	131455906	0.0087333	7.0937	1.54E-07	0.01923699	0.0016039	0.29431	0.91619	1	No	NA
ENSG00000177096	FAM109B	22	42470255	42475445	ENSG00000187634	SAMD11	1	860260	878757	0.0092156	6.4179	1.54E-07	0.01923699	NA	NA	NA	NA	No	NA
ENSG00000205045	SLFN12L	17	33800708	33864880	ENSG00000105248	CCDC94	19	4247076	4269087	0.0092145	6.4172	1.54E-07	0.01924401	0.0054229	0.71194	0.66197	1	No	NA
ENSG00000182134	TDRKH	1	151744040	151763892	ENSG00000124659	TBCC	6	42712219	42714558	0.0071144	11.546	1.54E-07	0.01924553	0.0036872	1.1325	0.33491	1	No	NA
ENSG00000139330	KERA	12	91444268	91451760	ENSG00000033867	SLC4A7	3	27414214	27525911	0.0064674	15.737	1.54E-07	0.01924553	0.00014324	0.06583	0.93629	1	No	NA
ENSG00000114388	NPRL2	3	50384919	50387453	ENSG00000175691	ZNF77	19	2933216	2944969	0.0064651	15.731	1.55E-07	0.0193424	0.0033764	1.0367	0.37549	1	No	NA
ENSG00000122574	WIPF3	7	29846102	29956682	ENSG00000198892	SHISA4	1	201857808	201861434	0.0092074	6.4122	1.56E-07	0.01949778	0.0096153	1.2677	0.26323	1	No	NA
ENSG00000002834	LASP1	17	37026112	37078023	ENSG00000162551	ALPL	1	21835858	21904905	0.010118	5.4833	1.57E-07	0.01951833	0.014145	1.6375	0.11016	1	No	NA
ENSG00000158485	CD1B	1	158297741	158301321	ENSG00000180767	CHST13	3	126243126	126262134	0.0071055	11.531	1.57E-07	0.0195999	0.0013368	0.40962	0.74613	1	No	NA
ENSG00000172660	TAF15	17	34136459	34174246	ENSG00000177666	PNPLA2	11	818902	825573	0.0087226	7.085	1.57E-07	0.01960294	0.0017936	0.27401	0.9492	1	Yes	No
ENSG00000108733	PEX12	17	33901814	33905882	ENSG00000162599	NFIA	1	61330931	61928465	0.0082164	8.0061	1.58E-07	0.01966694	0.0030835	0.56664	0.72564	1	No	NA
ENSG00000106635	BCL7B	7	72950686	72972332	ENSG00000140287	HDC	15	50534144	50558223	0.0064536	15.703	1.59E-07	0.01981792	0.0051075	2.3589	0.095092	1	Yes	No
ENSG00000139437	TCHP	12	110338079	110355874	ENSG00000086065	CHMP5	9	33264940	33281977	0.009196	6.4041	1.60E-07	0.01991775	0.012816	1.9798	0.065912	1	No	NA
ENSG00000125734	GPR108	19	6729925	6737614	ENSG00000152894	PTPRK	6	128289924	128841870	0.012581	4.0959	1.61E-07	0.0199889	0.0017224	0.52798	0.66315	1	No	NA
ENSG00000114353	GNAI2	3	50263724	50296787	ENSG00000103023	PRSS54	16	58318309	58328951	0.0056598	27.527	1.62E-07	0.02005376	NA	NA	NA	NA	No	NA
ENSG00000158485	CD1B	1	158297741	158301321	ENSG00000145391	SETD7	4	140417095	140477928	0.0070941	11.513	1.62E-07	0.02005402	0.0007228	0.22134	0.88161	1	No	NA
ENSG00000134222	PSRC1	1	109822178	109825808	ENSG00000145730	PAM	5	102089685	102366809	0.0091913	6.4008	1.62E-07	0.02007165	0.010638	1.404	0.20008	1	No	NA
ENSG00000009950	MLXIPL	7	73007524	73038873	ENSG00000131016	AKAP12	6	151561134	151679692	0.0076707	9.3397	1.62E-07	0.02007934	0.0056775	0.87076	0.51579	1	No	NA
ENSG00000139437	TCHP	12	110338079	110355874	ENSG00000115761	NOL10	2	10710892	10830101	0.0091854	6.3967	1.64E-07	0.02030137	0.002553	0.39033	0.88549	1	No	NA
ENSG00000177096	FAM109B	22	42470255	42475445	ENSG00000149295	DRD2	11	113280318	113346111	0.0091853	6.3966	1.64E-07	0.02030137	NA	NA	NA	NA	No	NA
ENSG00000164619	BMPER	7	33944523	34195484	ENSG00000134755	DSC2	18	28645940	28682378	0.010093	5.4693	1.65E-07	0.02044248	0.014512	1.4922	0.14603	1	No	NA
ENSG00000198682	PAPSS2	10	89419370	89507462	ENSG00000065029	ZNF76	6	35226759	35263762	0.0091794	6.3925	1.66E-07	0.02050677	0.0075175	1.1551	0.32835	1	No	NA
ENSG00000139330	KERA	12	91444268	91451760	ENSG00000163867	ZMYM6	1	35451766	35497569	0.0064366	15.661	1.66E-07	0.02050677	0.0016374	0.75364	0.47094	1	No	NA
ENSG00000139330	KERA	12	91444268	91451760	ENSG00000243749	ZMYM6NB	1	35450799	35450954	0.0064366	15.661	1.66E-07	0.02050677	NA	NA	NA	NA	No	NA
ENSG00000108733	PEX12	17	33901814	33905882	ENSG00000060138	YBX3	12	10851683	10875911	0.0081941	7.9842	1.66E-07	0.02050677	NA	NA	NA	NA	No	NA
ENSG00000100263	RHBDD3	22	29655841	29663967	ENSG00000153208	MERTK	2	112656056	112787138	0.0091732	6.3881	1.68E-07	0.02073953	0.010119	1.3348	0.2304	1	No	NA
ENSG00000023909	GCLM	1	94350761	94374966	ENSG00000149499	EML3	11	62369690	62378819	0.010519	5.1317	1.68E-07	0.02073953	0.0079232	0.72757	0.69891	1	No	NA
ENSG00000137941	TTLL7	1	84330711	84464833	ENSG00000173431	RNASE8	14	21525981	21526614	0.0081884	7.9786	1.68E-07	0.02073953	NA	NA	NA	NA	No	NA
ENSG00000174885	NLRP6	11	278365	285359	ENSG00000005961	ITGA2B	17	42449548	42466873	0.0091719	6.3872	1.69E-07	0.02078738	0.019451	2.0101	0.035374	1	Yes	Yes
ENSG00000185379	RAD51D	17	33426811	33447063	ENSG00000138798	EGF	4	110834040	110933422	0.0070746	11.481	1.69E-07	0.02081285	0.0030292	0.92976	0.42572	1	No	NA
ENSG00000112245	PTP4A1	6	64231666	64293492	ENSG00000198574	SH2D1B	1	162365056	162381928	0.0070745	11.481	1.69E-07	0.02081285	0.0026577	0.81541	0.48544	1	No	NA
ENSG00000127415	IDUA	4	980785	998316	ENSG00000132740	IGHMBP2	11	68671310	68708067	0.010515	5.1295	1.70E-07	0.02086304	0.0073862	0.84922	0.55936	1	No	NA
ENSG00000172716	SLFN11	17	33677324	33700720	ENSG00000102145	GATA1	X	48644962	48652718	0.011353	4.6173	1.70E-07	0.02090457	0.018765	1.4486	0.13818	1	Yes	No
ENSG00000131188	PRR7	5	176873446	176883283	ENSG00000136819	C9orf78	9	132589569	132598142	0.0081823	7.9727	1.71E-07	0.02094727	0.0013303	0.24403	0.94281	1	No	NA
ENSG00000183621	ZNF438	10	31133563	31320866	ENSG00000140022	STON2	14	81727000	81902809	0.010074	5.4593	1.72E-07	0.02105337	0.020863	2.1592	0.022743	1	No	NA

ENSG00000205045	SLFN12L	17	33800708	33864880	ENSG00000049323	LTBP1	2	33172039	33624576	0.0091644	6.3819	1.72E-07	0.02105337	0.011226	1.4825	0.16974		1	No	NA
ENSG00000112041	TULP1	6	35465651	35480715	ENSG00000067141	NEO1	15	73344051	73597547	0.0091635	6.3813	1.72E-07	0.02107141	0.011497	1.5186	0.15714		1	No	NA
ENSG00000132139	GAS2L2	17	34071530	34079897	ENSG00000143774	GUK1	1	228327663	228336685	0.0096256	5.8667	1.72E-07	0.02107841	0.0091645	1.0556	0.39232		1	Yes	No
ENSG00000134283	PPHLN1	12	42632249	42842420	ENSG00000146426	TIAM2	6	155181872	155575707	0.010072	5.4581	1.73E-07	0.0210985	0.015252	1.411	0.17015		1	No	NA
ENSG00000139330	KERA	12	91444268	91451760	ENSG00000113391	FAM172A	5	92953775	93447404	0.006421	15.623	1.73E-07	0.0210985	0.00056566	0.26007	0.77106		1	No	NA
ENSG00000008838	MED24	17	38175350	38217468	ENSG00000158089	GALNT14	2	31133333	31378068	0.0076432	9.306	1.73E-07	0.02110303	0.0054836	1.264	0.28243		1	Yes	Yes
ENSG00000066654	THUMPD1	16	20744986	20753406	ENSG00000186472	PCLO	7	82387442	82792246	0.0091592	6.3782	1.74E-07	0.02120648	NA	NA	NA	NA		No	NA
ENSG00000160716	CHRNB2	1	154540257	154552502	ENSG00000141469	SLC14A1	18	43304092	43332485	0.0086773	7.0478	1.74E-07	0.02124711	0.010711	1.6511	0.13003		1	No	NA
ENSG00000060762	MPC1	6	166778407	166796486	ENSG00000152990	GPR125	4	22346694	22517677	0.0091577	6.3772	1.74E-07	0.02124711	0.0089557	1.0313	0.41036		1	No	NA
ENSG00000139330	KERA	12	91444268	91451760	ENSG00000138663	COPS4	4	83955600	83996971	0.0064141	15.606	1.75E-07	0.02138687	0.00041179	0.1893	0.82757		1	No	NA
ENSG00000154760	SLFN13	17	33762115	33775856	ENSG00000008441	NFIX	19	13106422	13209610	0.010923	4.8451	1.76E-07	0.02139365	0.011126	0.85227	0.59614		1	Yes	No
ENSG00000177096	FAM109B	22	42470255	42475445	ENSG00000115297	TLX2	2	74740590	74744274	0.009153	6.3739	1.76E-07	0.02142843	NA	NA	NA	NA		No	NA
ENSG00000163947	ARHGEF3	3	56761446	57113357	ENSG00000173210	ABLIM3	5	148521046	148640105	0.0096149	5.8601	1.76E-07	0.02143584	0.07076	8.6904	1.75E-11	3.30E-08		No	NA
ENSG00000139330	KERA	12	91444268	91451760	ENSG00000095002	MSH2	2	47630108	47789450	0.0064122	15.602	1.76E-07	0.02143584	0.0016669	0.76724	0.46459		1	No	NA
ENSG00000139437	TCHP	12	110338079	110355874	ENSG00000091527	CDV3	3	133292574	133309105	0.0091514	6.3728	1.77E-07	0.02146445	0.013555	2.0955	0.051419		1	No	NA
ENSG00000132139	GAS2L2	17	34071530	34079897	ENSG00000151474	FRMD4A	10	13693903	14504141	0.0096107	5.8576	1.78E-07	0.02159022	0.0037667	0.4315	0.90252		1	No	NA
ENSG00000112062	MAPK14	6	35995488	36079013	ENSG00000145391	SETD7	4	140417095	140477928	0.010491	5.1176	1.79E-07	0.02167456	0.014942	1.5371	0.13028		1	No	NA
ENSG00000182541	LIMK2	22	31608260	31676066	ENSG00000172817	CYP7B1	8	65500320	65711318	0.008663	7.0361	1.80E-07	0.02180612	NA	NA	NA	NA		No	NA
ENSG00000139330	KERA	12	91444268	91451760	ENSG00000031003	FAM13B	5	137277633	137387650	0.0064037	15.581	1.80E-07	0.02181869	0.0060581	2.8006	0.06129		1	No	NA
ENSG00000269591	AL354808.2	13	20268547	20269240	ENSG00000112320	SOBP	6	107811162	107981357	0.0086619	7.0353	1.80E-07	0.02182881	0.0055328	0.84844	0.53263		1	No	NA
ENSG00000131791	PRKAB2	1	146626685	146644129	ENSG00000132017	DCAF15	19	14063304	14072254	0.0086598	7.0335	1.81E-07	0.02192129	0.0071959	1.1053	0.35718		1	No	NA
ENSG00000139330	KERA	12	91444268	91451760	ENSG00000115091	ACTR3	2	114647537	114720173	0.0064002	15.572	1.81E-07	0.02196766	0.0035701	1.6463	0.19332		1	No	NA
ENSG00000121101	TEX14	17	56634039	56769416	ENSG00000163803	PLB1	2	28680012	28866654	0.0086583	7.0322	1.82E-07	0.021968	0.013089	1.5136	0.14816		1	No	NA
ENSG00000172660	TAF15	17	34136459	34174246	ENSG00000204613	TRIM10	6	30119722	30128711	0.0086574	7.0315	1.82E-07	0.02199977	0.013125	2.0282	0.059438		1	Yes	No
ENSG00000166278	C2	6	31865562	31912806	ENSG00000005471	ABCB4	7	87031013	87109751	0.0086551	7.0296	1.83E-07	0.02209677	0.004918	0.7537	0.60655		1	No	NA
ENSG00000155100	OTUD6B	8	92082424	92099323	ENSG00000174175	SELP	1	169558087	169599431	0.0095971	5.8492	1.83E-07	0.02209945	0.010908	1.2586	0.26176		1	No	NA
ENSG00000142082	SIRT3	11	215458	236431	ENSG00000128266	GNAZ	22	23412540	23464889	0.0070413	11.426	1.83E-07	0.02210695	0.0010385	0.23833	0.91671		1	Yes	Yes
ENSG00000006125	AP2B1	17	33913918	34053436	ENSG00000148337	CIZ1	9	130928343	130966662	0.0091331	6.3599	1.84E-07	0.02214266	0.014945	2.3137	0.031856		1	Yes	Yes
ENSG00000139330	KERA	12	91444268	91451760	ENSG00000117020	AKT3	1	243651535	244014381	0.0063953	15.56	1.84E-07	0.02214266	0.00054474	0.25044	0.77851		1	No	NA
ENSG00000073969	NSF	17	44668035	44834830	ENSG00000134042	MRO	18	48324574	48351772	0.010042	5.4417	1.84E-07	0.02214266	NA	NA	NA	NA		No	NA
ENSG00000174885	NLRP6	11	278365	285359	ENSG00000117400	MPL	1	43803478	43818443	0.0091317	6.3589	1.84E-07	0.02219708	0.017381	1.7925	0.065794		1	Yes	Yes
ENSG00000205045	SLFN12L	17	33800708	33864880	ENSG00000105063	PPP6R1	19	55741981	55770363	0.0091305	6.3581	1.85E-07	0.02224181	0.011241	1.4845	0.16903		1	No	NA
ENSG00000144224	UBXN4	2	136499189	136542625	ENSG00000140968	IRF8	16	85932409	85956215	0.0086489	7.0246	1.85E-07	0.02229852	0.022817	3.5608	0.0017132		1	Yes	No
ENSG00000139330	KERA	12	91444268	91451760	ENSG00000166479	TMX3	18	66340925	66381680	0.0063908	15.549	1.86E-07	0.02230361	0.00095482	0.43916	0.64471		1	No	NA
ENSG00000169905	TOR1AIP2	1	179809102	179846934	ENSG00000038427	VCAN	5	82767284	82878122	0.0095904	5.8451	1.86E-07	0.02230361	0.0041533	0.54456	0.80089		1	No	NA
ENSG00000139330	KERA	12	91444268	91451760	ENSG00000165171	WBSCR27	7	73248920	73256865	0.0063899	15.547	1.86E-07	0.02233977	0.00027916	0.12831	0.8796		1	No	NA
ENSG00000141150	RASL10B	17	34058668	34070540	ENSG00000100614	PPM1A	14	60712470	60765805	0.009586	5.8423	1.87E-07	0.02245758	0.011187	1.4772	0.17167		1	Yes	No
ENSG00000139330	KERA	12	91444268	91451760	ENSG00000148180	GSN	9	123970072	124095121	0.0063873	15.541	1.87E-07	0.02245758	0.0070563	3.2654	0.038625		1	No	NA
ENSG00000168374	ARF4	3	57557090	57583947	ENSG00000110013	SIAE	11	124508438	124539417	0.0081411	7.9322	1.87E-07	0.02245758	0.004353	0.80096	0.54904		1	No	NA
ENSG00000145088	EAF2	3	121554030	121605373	ENSG00000134057	CCNB1	5	68462837	68474072	0.0086428	7.0196	1.88E-07	0.02250674	0.0018528	0.28308	0.94507		1	No	NA
ENSG00000117411	B4GALT2	1	44444615	44456840	ENSG00000063127	SLC6A16	19	49792895	49828482	0.0086419	7.0188	1.88E-07	0.02254268	0.0080044	1.4782	0.19431		1	No	NA
ENSG00000205045	SLFN12L	17	33800708	33864880	ENSG00000196517	SLC6A9	1	44463527	44497139	0.0091209	6.3513	1.89E-07	0.02257857	0.0032494	0.42567	0.88658		1	No	NA
ENSG00000139330	KERA	12	91444268	91451760	ENSG00000164163	ABCE1	4	146025538	146046253	0.0063824	15.529	1.89E-07	0.02265747	0.0060039	2.7755	0.062842		1	No	NA
ENSG00000088543	C3orf18	3	50595462	50605182	ENSG00000131059	BPIFA3	20	31805116	31815564	0.0081349	7.9261	1.90E-07	0.0227064	NA	NA	NA	NA		No	NA
ENSG00000106635	BCL7B	7	72950686	72972332	ENSG00000149534	MS4A2	11	59855734	59863444	0.0063805	15.524	1.90E-07	0.02273974	0.0020718	0.95398	0.38558		1	No	NA
ENSG00000121101	TEX14	17	56634039	56769416	ENSG00000145012	LPP	3	187871072	188608460	0.0086323	7.0109	1.92E-07	0.02296045	0.0068997	0.7929	0.60898		1	No	NA
ENSG00000139330	KERA	12	91444268	91451760	ENSG00000088205	DDX18	2	118572226	118589955	0.0063758	15.512	1.93E-07	0.02297333	0.00031154	0.1432	0.8666		1	No	NA
ENSG00000173638	SLC19A1	21	46918115	46964325	ENSG00000170312	CDK1	10	62538089	62554610	0.010017	5.4279	1.94E-07	0.02310661	0.014736	1.3625	0.19285		1	No	NA
ENSG00000103091	WDR59	16	74907468	75031071	ENSG00000256660	CLEC12B	12	10165384	10171218	0.0086267	7.0063	1.95E-07	0.02319288	0.0042769	0.65503	0.68611		1	No	NA
ENSG00000214706	IFRD2	3	50325840	50329487	ENSG00000182156	ENPP7	17	77704681	77716021	0.0075912	9.2422	1.95E-07	0.02319288	NA	NA	NA	NA		No	NA
ENSG00000204310	AGPAT1	6	32137942	32145873	ENSG00000137878	GCOM1	15	57938335	57993912	0.0095664	5.8303	1.95E-07	0.02324447	0.0086643	0.99745	0.43631		1	No	NA
ENSG00000204310	AGPAT1	6	32137942	32145873	ENSG00000263155	MYZAP	15	57891609	57891901	0.0095664	5.8303	1.95E-07	0.02324447	NA	NA	NA	NA		No	NA
ENSG00000073605	GSDMB	17	38060848	38074903	ENSG00000113916	BCL6	3	187439165	187455732	0.007587	9.237	1.97E-07	0.02337483	0.0035598	0.81901	0.5131		1	No	NA
ENSG00000163946	FAM208A	3	56658507	56717265	ENSG00000128266	GNAZ	22	23412540	23464889	0.0095625	5.8279	1.97E-07	0.02340255	0.021893	2.9226	0.0049805		1	Yes	No
ENSG00000100227	POLDIP3	22	42979727	43010968	ENSG00000160593	AMICA1	11	118064455	118095809	0.0075858	9.2356	1.97E-07	0.02340255	0.0074103	1.3677	0.23404		1	No	NA
ENSG00000205413	SAMD9	7	92728829	92747336	ENSG00000105401	CDC37	19	10501810	10530797	0.0070098	11.375	1.97E-07	0.02340255	0.0018844	0.57773	0.62974		1	No	NA
ENSG00000139330	KERA	12	91444268	91451760	ENSG00000078674	PCM1	8	17780349	17885478	0.006366	15.488	1.97E-07	0.02340255	0.00020949	0.096282	0.90822		1	No	NA
ENSG00000125510	OPRL1	20	62711526	62731996	ENSG00000116299	KIAA1324	1	109656623	109745853	0.0090995	6.3363	1.98E-07	0.02341933	0.0027123	1.2497	0.28708		1	No	NA
ENSG00000099330	OCEL1	19	17337013	17340028	ENSG00000171488	LRRC8C	1	90171861	90235462	0.010865	4.8191	1.98E-07	0.02341933	0.0058825	0.77264	0.6104		1	No	NA
ENSG00000143416	SELENBP1	1	151336778	151345209	ENSG00000169397	RNASE3	14	21359558	21360507	0.0086191	7.0002	1.98E-07	0.02344842	0.0020483	0.26799	0.96623		1	No	NA
ENSG00000139330	KERA	12	91444268	91451760	ENSG00000108654	DDX5	17	62495734	62504317	0.0063624	15.48	1.99E-07	0.0235402	2.65E-05	0.012198	0.98788		1	No	NA
ENSG00000197912	SPG7	16	89557325	89624176	ENSG00000175718	RBMXL3	X	114423963	114427431	0.0086164	6.998	1.99E-07	0.02355973	NA	NA	NA	NA		No	NA
ENSG00000205045	SLFN12L	17	33800708	33864880	ENSG00000162367	TAL1	1	47681962	47697892	0.0090935	6.3321	2.00E-07	0.02367142	0.0021328	0.27908	0.96219		1	No	NA
ENSG00000139304	PTPRQ	12	80799774	81074013	ENSG00000182240	BACE2	21	42539128	42648524	0.0081104	7.902	2.01E-07	0.02372275	0.0090693	1.6767	0.13753		1	No	NA
ENSG00000083838	ZNF446	19	58985384	58989548	ENSG00000038945	MSR1	8	15965387	16424999	0.0090912	6.3305	2.01E-07	0.02373182	0.0065623	1.0074	0.419		1	No	NA
ENSG00000006125	AP2B1	17	33913918	34053436	ENSG00000171914	TLN2	15	62682725	63136830	0.0090915	6.3307	2.01E-07	0.02373182	0.0064253	0.9862	0.43323		1	No	NA
ENSG00000134873	CLDN10	13	96085858	96229592	ENSG00000131238	PPT1	1	40538379	40563375	0.009091	6.3304	2.01E-07	0.02373182	0.0058689	0.77083	0.61192		1	No	NA

ENSG00000011275	RNF216	7	5659678	5821370	ENSG00000137106	GRHPR	9	37422663	37436987	0.0095527	5.8219	2.01E-07	0.02373182	0.0040354	0.6179	0.71613	1	No	NA
ENSG00000150526	MIA2	14	39699435	39722170	ENSG00000212710	CTAGE1	18	19993564	19997878	0.0070005	11.36	2.02E-07	0.023754	0.013595	4.2174	0.0056579	1	No	NA
ENSG00000185418	TARSL2	15	102193801	102264807	ENSG00000162851	TFB2M	1	246703862	246729626	0.0095514	5.821	2.02E-07	0.023754	0.016602	1.7108	0.08234	1	No	NA
ENSG00000139330	KERA	12	91444268	91451760	ENSG00000164597	COG5	7	106848293	107198560	0.0063561	15.464	2.02E-07	0.0237686	0.0018017	0.82938	0.43665	1	No	NA
ENSG00000139437	TCHP	12	110338079	110355874	ENSG00000115875	SRSF7	2	38970741	38978636	0.0090886	6.3287	2.02E-07	0.02380199	0.0081921	1.2596	0.27353	1	No	NA
ENSG00000111344	RASAL1	12	113536624	113574044	ENSG00000165376	CLDN2	X	106161590	106174091	0.0063549	15.461	2.03E-07	0.02381067	NA	NA	NA	NA	No	NA
ENSG00000205045	SLFN12L	17	33800708	33864880	ENSG00000164849	GPR146	7	1084212	1098897	0.0090868	6.3274	2.03E-07	0.0238722	0.0083264	1.0963	0.36324	1	No	NA
ENSG00000170684	ZNF296	19	45574758	45579846	ENSG00000148346	LCN2	9	130911350	130915734	0.0069959	11.352	2.04E-07	0.02392112	0.0011337	0.26018	0.90347	1	No	NA
ENSG00000154768	C17orf50	17	34087916	34092098	ENSG00000165406	8-Mar	10	45950035	46090354	0.0086061	6.9895	2.04E-07	0.02392112	0.0023658	0.30964	0.94978	1	No	NA
ENSG00000132436	FIGNL1	7	50511831	50518088	ENSG00000167992	VWCE	11	61025762	61062896	0.0055652	27.064	2.05E-07	0.02402937	0.0068523	6.3476	0.011922	1	Yes	Yes
ENSG00000119946	CNNM1	10	101088856	101154087	ENSG00000149573	MPZL2	11	118124118	118135251	0.0095412	5.8148	2.06E-07	0.02416679	0.0045831	0.60117	0.75529	1	No	NA
ENSG00000247121	CTD-2260A17.2	5	96149731	96209376	ENSG00000137757	CASP5	11	104864962	104893895	0.0075658	9.211	2.06E-07	0.02418226	0.0036833	0.84751	0.49519	1	No	NA
ENSG00000162636	FAM102B	1	109102711	109187522	ENSG00000042317	SPATA7	14	88851268	88921627	0.0090758	6.3197	2.08E-07	0.02436157	0.015109	2.3395	0.030076	1	No	NA
ENSG00000143248	RGS5	1	163080911	163291577	ENSG00000185294	SPPL2C	17	43922256	43924438	0.0085953	6.9806	2.09E-07	0.02442253	NA	NA	NA	NA	No	NA
ENSG00000205045	SLFN12L	17	33800708	33864880	ENSG00000148337	CIZ1	9	130928343	130966662	0.0090734	6.318	2.09E-07	0.02444843	0.010547	1.3918	0.20515	1	Yes	Yes
ENSG00000205045	SLFN12L	17	33800708	33864880	ENSG00000165661	QSOX2	9	139098179	139137687	0.0090736	6.3181	2.09E-07	0.02444843	0.0035583	0.46628	0.85925	1	No	NA
ENSG00000149043	SYT8	11	1848709	1858751	ENSG00000136770	DNAJC1	10	22045466	22292698	0.0069829	11.331	2.10E-07	0.02455828	0.0047438	1.0927	0.35884	1	No	NA
ENSG00000124782	RREB1	6	7107830	7252213	ENSG00000092067	CEBPE	14	23586513	23588825	0.011245	4.5727	2.12E-07	0.02471353	0.010298	0.7882	0.66312	1	No	NA
ENSG00000135966	TGFBRAP1	2	105885729	105946491	ENSG00000172967	XKR3	22	17264302	17302589	0.0095281	5.8067	2.12E-07	0.02473562	0.0098721	1.3019	0.24607	1	No	NA
ENSG00000166295	ANAPC16	10	73982891	73995618	ENSG00000162931	TRIM17	1	228595641	228604562	0.0069782	11.323	2.13E-07	0.02479267	0.0018042	0.55307	0.64617	1	No	NA
ENSG00000129270	MMP28	17	34083268	34122711	ENSG00000079308	TNS1	2	218664512	218867718	0.0085842	6.9716	2.14E-07	0.02491471	0.0069824	0.91812	0.49145	1	No	NA
ENSG00000139330	KERA	12	91444268	91451760	ENSG00000085719	CPNE3	8	87526656	87573726	0.0063326	15.407	2.14E-07	0.02491471	0.00062397	0.2869	0.75066	1	No	NA
ENSG00000141753	IGFBP4	17	38599702	38613983	ENSG00000133392	MYH11	16	15796992	15950890	0.008081	7.8731	2.15E-07	0.02497853	0.00083136	0.15243	0.97933	1	No	NA
ENSG00000186265	BTLA	3	112182815	112218408	ENSG00000138801	PAPSS1	4	108511433	108641608	0.0063297	15.4	2.15E-07	0.02505392	0.0019787	0.91102	0.40248	1	No	NA
ENSG00000145901	TNIP1	5	150409506	150473138	ENSG00000170448	NFXL1	4	47849257	47913011	0.0085783	6.9667	2.17E-07	0.02520013	0.014515	2.2462	0.036997	1	No	NA
ENSG00000139330	KERA	12	91444268	91451760	ENSG00000151292	CSNK1G3	5	122847793	122952739	0.0063263	15.391	2.17E-07	0.02522266	0.0012989	0.5976	0.55034	1	No	NA
ENSG00000176984	AP000679.2	11	120039685	120042387	ENSG00000203757	OR6K3	1	158686958	158687953	0.0055423	26.952	2.17E-07	0.02522266	NA	NA	NA	NA	No	NA
ENSG00000205089	CCNI2	5	132083137	132089856	ENSG00000165192	ASB11	X	15298053	15333778	0.0063253	15.389	2.18E-07	0.02526757	NA	NA	NA	NA	No	NA
ENSG00000131771	PPP1R1B	17	37782993	37792879	ENSG00000125538	IL1B	2	113587328	113594480	0.0099585	5.3959	2.19E-07	0.02540197	0.014533	1.4944	0.14521	1	Yes	No
ENSG00000174586	ZNF497	19	58865723	58874214	ENSG00000196247	ZNF107	7	64126511	64171404	0.0090519	6.3029	2.19E-07	0.02540197	0.010134	1.3367	0.22951	1	No	NA
ENSG00000139330	KERA	12	91444268	91451760	ENSG00000136122	BORA	13	73302061	73327959	0.0063229	15.383	2.19E-07	0.02540197	NA	NA	NA	NA	No	NA
ENSG00000185379	RAD51D	17	33426811	33447063	ENSG00000151693	ASAP2	2	9346894	9541525	0.0069621	11.297	2.21E-07	0.02558099	0.005396	1.6601	0.17403	1	No	NA
ENSG00000155099	TMEM55A	8	92006024	92053292	ENSG00000198478	SH3BGRL2	6	80341000	80413372	0.0069614	11.296	2.21E-07	0.02559144	0.0046603	1.4327	0.23176	1	No	NA
ENSG00000131791	PRKAB2	1	146626685	146644129	ENSG00000142208	AKT1	14	105235686	105262088	0.0085691	6.9592	2.21E-07	0.02559144	0.0033575	0.51375	0.79823	1	No	NA
ENSG00000006125	AP2B1	17	33913918	34053436	ENSG00000143416	SELENBP1	1	151336778	151345209	0.0090473	6.2996	2.21E-07	0.02560012	0.0024559	0.37544	0.89484	1	No	NA
ENSG00000141140	MYO19	17	34856670	34899284	ENSG00000158856	DMTN	8	21906506	21940038	0.0080668	7.8592	2.22E-07	0.02560418	NA	NA	NA	NA	Yes	No
ENSG00000139330	KERA	12	91444268	91451760	ENSG00000146463	ZMYM4	1	35734568	35887659	0.0063174	15.369	2.22E-07	0.02561458	0.0044776	2.0667	0.12719	1	No	NA
ENSG00000146648	EGFR	7	55086714	55324313	ENSG00000188452	CERKL	2	182401403	182521843	0.0099526	5.3927	2.22E-07	0.02561458	0.0051444	0.524	0.85785	1	No	NA
ENSG00000141002	TCF25	16	89940014	89977792	ENSG00000123989	CHPF	2	220403669	220407819	0.0080657	7.8581	2.22E-07	0.02562554	0.0016655	0.25442	0.95757	1	No	NA
ENSG00000141741	MIEN1	17	37885409	37886014	ENSG00000146592	CREB5	7	28338940	28865511	0.0080654	7.8578	2.22E-07	0.02562726	0.0029215	0.53679	0.7485	1	Yes	No
ENSG00000108733	PEX12	17	33901814	33905882	ENSG00000141469	SLC14A1	18	43304092	43332485	0.0080646	7.8569	2.23E-07	0.02566354	0.0058884	1.0851	0.36696	1	No	NA
ENSG00000139330	KERA	12	91444268	91451760	ENSG00000137996	RTCA	1	100731763	100758325	0.0063149	15.363	2.23E-07	0.025709	NA	NA	NA	NA	No	NA
ENSG00000139330	KERA	12	91444268	91451760	ENSG00000143183	TMCO1	1	165696032	165769272	0.0063142	15.362	2.24E-07	0.02574174	0.00067614	0.3109	0.73287	1	No	NA
ENSG00000197372	ZNF675	19	23708433	23870022	ENSG00000175066	GK5	3	141882414	141944449	0.0099482	5.3903	2.24E-07	0.0257549	0.015687	1.4518	0.15274	1	No	NA
ENSG00000139330	KERA	12	91444268	91451760	ENSG00000145725	PPIP5K2	5	102455904	102548500	0.0063132	15.359	2.24E-07	0.02577724	0.0036157	1.6674	0.1893	1	No	NA
ENSG00000169900	PYDC1	16	31227283	31228680	ENSG00000130511	SSBP4	19	18529674	18544652	0.0075283	9.165	2.25E-07	0.02586848	0.015415	3.5891	0.0065075	1	No	NA
ENSG00000140983	RHOT2	16	718086	724174	ENSG00000185499	MUC1	1	155158300	155162707	0.0085603	6.952	2.26E-07	0.02590335	0.019358	2.5775	0.012357	1	No	NA
ENSG00000169905	TOR1AIP2	1	179809102	179846934	ENSG00000105967	TFEC	7	115575202	115799950	0.0094988	5.7887	2.26E-07	0.02590721	0.0099547	1.3129	0.24074	1	No	NA
ENSG00000006125	AP2B1	17	33913918	34053436	ENSG00000095321	CRAT	9	131857089	131873083	0.0090377	6.2929	2.26E-07	0.025934	0.015789	2.4465	0.02366	1	Yes	Yes
ENSG00000267596	CCL15	17	34323476	34323962	ENSG00000198574	SH2D1B	1	162365056	162381928	0.0090358	6.2916	2.27E-07	0.02601842	0.025247	3.3819	0.0014308	1	No	NA
ENSG00000177951	BET1L	11	167784	207428	ENSG00000148498	PARD3	10	34398488	35104253	0.009496	5.787	2.27E-07	0.02601842	0.01315	1.1024	0.35579	1	No	NA
ENSG00000163946	FAM208A	3	56658507	56717265	ENSG00000107438	PDLIM1	10	96997329	97050781	0.0094953	5.7865	2.27E-07	0.0260451	0.017383	2.3099	0.024468	1	Yes	No
ENSG00000151468	CCDC3	10	12938627	13141309	ENSG00000100028	SNRPD3	22	24953625	24978020	0.012402	4.037	2.28E-07	0.02605345	0.026241	1.7459	0.042409	1	No	NA
ENSG00000174840	PDE12	3	57542003	57552571	ENSG00000259207	ITGB3	17	45387505	45389182	0.0075218	9.1571	2.29E-07	0.02614184	0.0091547	2.8272	0.037617	1	Yes	No
ENSG00000139330	KERA	12	91444268	91451760	ENSG00000122435	TRMT13	1	100598706	100613882	0.0063022	15.332	2.30E-07	0.02632386	NA	NA	NA	NA	No	NA
ENSG00000139330	KERA	12	91444268	91451760	ENSG00000113575	PPP2CA	5	133530025	133561245	0.0063019	15.331	2.30E-07	0.02632975	0.00052203	0.24	0.78668	1	No	NA
ENSG00000136379	ABHD17C	15	80972025	81047962	ENSG00000176148	TCP11L1	11	33060963	33102770	0.010367	5.0564	2.31E-07	0.02635047	0.01348	1.2449	0.25803	1	No	NA
ENSG00000172660	TAF15	17	34136459	34174246	ENSG00000183508	FAM46C	1	118148556	118170994	0.0085468	6.941	2.32E-07	0.02652066	0.0041863	0.64109	0.69739	1	No	NA
ENSG00000186075	ZPBP2	17	38024417	38034149	ENSG00000113916	BCL6	3	187439165	187455732	0.0055134	26.811	2.33E-07	0.02662679	0.001057	0.97351	0.32407	1	No	NA
ENSG00000241399	CD302	2	160637946	160654753	ENSG00000116991	SIPA1L2	1	232533711	232697304	0.0090204	6.2807	2.35E-07	0.02675668	0.014283	2.2097	0.040086	1	No	NA
ENSG00000163666	HESX1	3	57231944	57260549	ENSG00000174175	SELP	1	169558087	169599431	0.0069362	11.255	2.35E-07	0.02675668	0.0022286	0.68347	0.56225	1	No	NA
ENSG00000186417	GLDN	15	51633826	51700210	ENSG00000128606	LRRC17	7	102574221	102585396	0.0099248	5.3775	2.35E-07	0.02677249	0.015471	1.5923	0.11291	1	No	NA
ENSG00000109705	NKX3-2	4	13542454	13546674	ENSG00000147081	AKAP4	X	49955406	49965664	0.0075093	9.1417	2.35E-07	0.02677249	NA	NA	NA	NA	No	NA
ENSG00000176532	PRR15	7	29603427	29606911	ENSG00000038945	MSR1	8	15965387	16424999	0.0094789	5.7765	2.36E-07	0.02679289	0.010836	1.4303	0.18942	1	No	NA
ENSG00000131771	PPP1R1B	17	37782993	37792879	ENSG00000135842	FAM129A	1	184759858	184943682	0.0099229	5.3764	2.36E-07	0.02681875	0.012831	1.3171	0.22351	1	Yes	No
ENSG00000188076	SCGB1C1	11	193080	194573	ENSG00000205038	PKHD1L1	8	110374706	110542559	0.0055088	26.788	2.36E-07	0.02681875	0.00045496	0.41875	0.51772	1	Yes	Yes
ENSG00000139330	KERA	12	91444268	91451760	ENSG00000114098	ARMC8	3	137906109	138017231	0.0062922	15.308	2.36E-07	0.02681875	0.0082655	3.8297	0.022064	1	No	NA

ENSG00000139330	KERA	12	91444268	91451760	ENSG00000182952	HMGN4	6	26538633	26546482	0.0062913	15.306	2.36E-07	0.02683681	0.0022425	1.0328	0.35644	1	No	NA
ENSG00000212900	KRTAP3-2	17	39155447	39156138	ENSG00000182979	MTA1	14	105886159	105937066	0.0069326	11.249	2.37E-07	0.0268536	0.0011213	0.34351	0.79387	1	No	NA
ENSG00000212900	KRTAP3-2	17	39155447	39156138	ENSG00000226174	TEX22	14	105864916	105880196	0.0069326	11.249	2.37E-07	0.0268536	NA	NA	NA	NA	No	NA
ENSG00000164163	ABCE1	4	146025538	146046253	ENSG00000154124	FAM105B	5	14664773	14699820	0.0055065	26.777	2.38E-07	0.02691466	0.0010208	0.94011	0.3325	1	No	NA
ENSG00000205045	SLFN12L	17	33800708	33864880	ENSG00000087460	GNAS	20	57414773	57486247	0.0090111	6.2742	2.40E-07	0.02712625	0.010727	1.4158	0.19525	1	Yes	Yes
ENSG00000140836	ZFHX3	16	72816784	73093597	ENSG00000266714	MYO15B	17	73584139	73622308	0.0090101	6.2735	2.40E-07	0.02715738	0.01123	1.2962	0.24164	1	No	NA
ENSG00000172992	DCAKD	17	43100708	43138473	ENSG00000197790	OR52M1	11	4566421	4567374	0.0069269	11.239	2.40E-07	0.02715738	NA	NA	NA	NA	No	NA
ENSG00000205045	SLFN12L	17	33800708	33864880	ENSG00000154917	RAB6B	3	133553419	133614680	0.0090084	6.2723	2.41E-07	0.02723025	0.0056981	0.74828	0.63103	1	Yes	Yes
ENSG00000139547	RDH16	12	57345219	57353158	ENSG00000105085	MED26	19	16698215	16739873	0.0080298	7.8228	2.41E-07	0.02723025	0.0066635	1.2289	0.29338	1	No	NA
ENSG00000125869	LAMP5	20	9495005	9511171	ENSG00000196663	TECPR2	14	102829300	102968818	0.010767	4.775	2.42E-07	0.02727848	0.0051237	0.46917	0.91028	1	No	NA
ENSG00000164167	LSM6	4	147096837	147121152	ENSG00000142409	ZNF787	19	56598732	56632649	0.010765	4.7743	2.42E-07	0.02735375	0.015893	1.336	0.19905	1	No	NA
ENSG00000164163	ABCE1	4	146025538	146046253	ENSG00000129221	AIPL1	17	6297013	6338519	0.0054945	26.718	2.45E-07	0.02762067	NA	NA	NA	NA	No	NA
ENSG00000109113	RAB34	17	27041299	27045447	ENSG00000129559	NEDD8	14	24695811	24697918	0.0085229	6.9213	2.45E-07	0.0276269	0.0084769	1.3038	0.25263	1	No	NA
ENSG00000144867	SRPRB	3	133502877	133540336	ENSG00000137968	SLC44A5	1	75667816	76076801	0.00946	5.7648	2.45E-07	0.02763762	0.0030233	0.34608	0.94776	1	No	NA
ENSG00000163946	FAM208A	3	56658507	56717265	ENSG00000180354	C7orf41	7	30174426	30202378	0.0094592	5.7644	2.46E-07	0.02766523	0.020022	2.6677	0.0097717	1	Yes	No
ENSG00000162814	SPATA17	1	217804666	218045038	ENSG00000144635	DYNC1LI1	3	32567463	32612366	0.00749	9.118	2.46E-07	0.02768605	0.0022956	0.52747	0.71558	1	No	NA
ENSG00000092871	RFFL	17	33341759	33416338	ENSG00000198959	TGM2	20	36756863	36794980	0.0085209	6.9197	2.46E-07	0.02768885	0.0053425	0.61298	0.76749	1	Yes	No
ENSG00000177096	FAM109B	22	42470255	42475445	ENSG00000105219	CNTD2	19	40728115	40732597	0.008998	6.265	2.47E-07	0.02771301	0.0037626	0.49314	0.83998	1	No	NA
ENSG00000092871	RFFL	17	33341759	33416338	ENSG00000090674	MCOLN1	19	7587512	7595387	0.0085186	6.9178	2.47E-07	0.02779606	0.0049879	0.5721	0.80135	1	Yes	No
ENSG00000165899	OTOGL	12	80603233	80772870	ENSG00000180537	RNF182	6	13924677	13980533	0.010333	5.0398	2.48E-07	0.02779606	0.0086653	0.79631	0.63242	1	No	NA
ENSG00000136295	TTYH3	7	2671585	2704436	ENSG00000150636	CCDC102B	18	66383765	66722426	0.007487	9.1144	2.48E-07	0.0277988	0.002847	0.52306	0.75895	1	No	NA
ENSG00000139330	KERA	12	91444268	91451760	ENSG00000132356	PRKAA1	5	40759481	40798476	0.0062715	15.257	2.48E-07	0.02784304	0.0012517	0.57586	0.56242	1	No	NA
ENSG00000142082	SIRT3	11	215458	236431	ENSG00000185245	GP1BA	17	4835592	4838325	0.0069122	11.215	2.49E-07	0.02784713	0.011592	2.6886	0.030106	1	Yes	No
ENSG00000136696	IL36B	2	113779668	113810444	ENSG00000186532	SMYD4	17	1682779	1733928	0.0054882	26.687	2.49E-07	0.02784713	0.0013107	1.2075	0.27212	1	No	NA
ENSG00000170442	KRT86	12	52643084	52702947	ENSG00000115523	GNLY	2	85921414	85925977	0.0098987	5.3632	2.49E-07	0.02784713	0.0071275	0.72744	0.684	1	No	NA
ENSG00000158485	CD1B	1	158297741	158301321	ENSG00000133794	ARNTL	11	13298199	13408813	0.0069121	11.215	2.49E-07	0.02784713	0.0016069	0.4925	0.68756	1	No	NA
ENSG00000108306	FBXL20	17	37415384	37558776	ENSG00000145721	LIX1	5	96427574	96478576	0.0080151	7.8084	2.49E-07	0.02786437	0.0089386	1.6523	0.1436	1	No	NA
ENSG00000004534	RBM6	3	49977440	50114683	ENSG00000166206	GABRB3	15	26788693	27184686	0.0062697	15.253	2.49E-07	0.02786818	NA	NA	NA	NA	No	NA
ENSG00000004059	ARF5	7	127228399	127231142	ENSG00000160818	GPATCH4	1	156564279	156571282	0.0085142	6.9142	2.50E-07	0.02792115	0.0085331	1.3125	0.24866	1	No	NA
ENSG00000146648	EGFR	7	55086714	55324313	ENSG00000144306	SCRN3	2	175260458	175294303	0.0098948	5.3611	2.51E-07	0.02797964	0.0059829	0.60992	0.78931	1	No	NA
ENSG00000241839	PLEKHO2	15	65157098	65160206	ENSG00000146021	KLHL3	5	136953189	137071779	0.0074813	9.1075	2.51E-07	0.02799976	0.001784	0.40972	0.80173	1	No	NA
ENSG00000136371	MTHFS	15	80125927	80189721	ENSG00000142408	CACNG8	19	54466294	54493469	0.0069079	11.208	2.51E-07	0.02799976	0.00084059	0.25744	0.85606	1	No	NA
ENSG00000177096	FAM109B	22	42470255	42475445	ENSG00000112238	PRDM13	6	100054606	100063454	0.0089895	6.259	2.51E-07	0.02799976	NA	NA	NA	NA	No	NA
ENSG00000114654	EFCC1	3	128720472	128759585	ENSG00000149534	MS4A2	11	59855734	59863444	0.0089882	6.2581	2.52E-07	0.02806255	0.0017453	0.22828	0.97861	1	No	NA
ENSG00000108733	PEX12	17	33901814	33905882	ENSG00000130733	YIPF2	19	11034171	11039357	0.0080098	7.8032	2.52E-07	0.02807102	0.011494	2.1302	0.05976	1	No	NA
ENSG00000137288	MNF1	6	33664538	33679504	ENSG00000138161	CUZD1	10	124591665	124605736	0.0089876	6.2577	2.52E-07	0.02807102	0.0093831	1.2368	0.27953	1	No	NA
ENSG00000176194	CIDEA	18	12254318	12277594	ENSG00000090857	PDPR	16	70147529	70195203	0.0094466	5.7566	2.52E-07	0.0280836	0.0090748	1.0451	0.40001	1	No	NA
ENSG00000157500	APPL1	3	57261765	57301820	ENSG00000049323	LTBP1	2	33172039	33624576	0.0074766	9.1017	2.54E-07	0.02820429	0.0018956	0.58117	0.62746	1	Yes	No
ENSG00000177096	FAM109B	22	42470255	42475445	ENSG00000131620	ANO1	11	69924408	70035634	0.0089846	6.2556	2.54E-07	0.02820429	NA	NA	NA	NA	No	NA
ENSG00000143536	CRNN	1	152381719	152386739	ENSG00000166313	APBB1	11	6416355	6440644	0.0098883	5.3575	2.54E-07	0.02821011	0.012993	1.3339	0.21486	1	No	NA
ENSG00000143466	IKBKE	1	206643791	206670223	ENSG00000180537	RNF182	6	13924677	13980533	0.011558	4.3389	2.54E-07	0.02823394	0.0092301	1.2164	0.29068	1	No	NA
ENSG00000164163	ABCE1	4	146025538	146046253	ENSG00000148082	SHC3	9	91628060	91793682	0.0054793	26.644	2.54E-07	0.02823394	0.00062531	0.57565	0.44822	1	No	NA
ENSG00000004059	ARF5	7	127228399	127231142	ENSG00000115241	PPM1G	2	27604061	27632554	0.0085043	6.9061	2.55E-07	0.02831847	0.007471	1.1479	0.33241	1	No	NA
ENSG00000139330	KERA	12	91444268	91451760	ENSG00000118965	WDR35	2	20110021	20189892	0.0062594	15.227	2.56E-07	0.02833419	0.00098935	0.45506	0.63455	1	No	NA
ENSG00000139330	KERA	12	91444268	91451760	ENSG00000118246	FASTKD2	2	207631368	207657233	0.0062584	15.225	2.56E-07	0.02838423	0.00010003	0.045967	0.95508	1	No	NA
ENSG00000198682	PAPSS2	10	89419370	89507462	ENSG00000010327	STAB1	3	52529354	52558229	0.0089797	6.2521	2.57E-07	0.02840211	0.005403	0.82843	0.54793	1	No	NA
ENSG00000243477	NAT6	3	50333833	50335514	ENSG00000131059	BPIFA3	20	31805116	31815564	0.0085017	6.904	2.57E-07	0.02842219	NA	NA	NA	NA	No	NA
ENSG00000163947	ARHGEF3	3	56761446	57113357	ENSG00000105507	CABP5	19	48533210	48547310	0.0094367	5.7505	2.58E-07	0.02849433	0.021387	2.4942	0.011107	1	No	NA
ENSG00000139330	KERA	12	91444268	91451760	ENSG00000119041	GTF3C3	2	197631289	197664449	0.0062561	15.219	2.58E-07	0.02849433	0.00055106	0.25335	0.77625	1	No	NA
ENSG00000007402	CACNA2D2	3	50400233	50541675	ENSG00000120937	NPPB	1	11917521	11918988	0.0068944	11.186	2.59E-07	0.02864249	NA	NA	NA	NA	No	NA
ENSG00000163947	ARHGEF3	3	56761446	57113357	ENSG00000013016	EHD3	2	31456880	31492313	0.0094331	5.7483	2.60E-07	0.02866573	0.020226	2.356	0.016448	1	No	NA
ENSG00000139330	KERA	12	91444268	91451760	ENSG00000163312	HELQ	4	84328496	84377009	0.0062526	15.211	2.60E-07	0.02866573	0.0032593	1.5025	0.22311	1	No	NA
ENSG00000088826	SMOX	20	4101627	4168394	ENSG00000138376	BARD1	2	215590370	215674428	0.0098778	5.3517	2.60E-07	0.02866573	0.01029	1.0536	0.39513	1	No	NA
ENSG00000154760	SLFN13	17	33762115	33775856	ENSG00000196914	ARHGEF12	11	120207787	120360645	0.01073	4.7584	2.61E-07	0.02871866	0.022119	1.7134	0.059157	1	No	NA
ENSG00000139330	KERA	12	91444268	91451760	ENSG00000213999	MEF2B	19	19267834	19281098	0.0062507	15.206	2.61E-07	0.02871971	0.0089058	4.129	0.016399	1	No	NA
ENSG00000139330	KERA	12	91444268	91451760	ENSG00000198917	C9orf114	9	131586026	131592100	0.0062508	15.206	2.61E-07	0.02871971	0.00040545	0.18638	0.82999	1	No	NA
ENSG00000139330	KERA	12	91444268	91451760	ENSG00000254901	MEF2BNB	19	19287712	19288324	0.0062507	15.206	2.61E-07	0.02871971	NA	NA	NA	NA	No	NA
ENSG00000139330	KERA	12	91444268	91451760	ENSG00000064489	MEF2BNB-M	19	19268025	19268092	0.0062507	15.206	2.61E-07	0.02871971	NA	NA	NA	NA	No	NA
ENSG00000129422	MTUS1	8	17504491	17658426	ENSG00000156689	GLYATL2	11	58601542	58671688	0.011139	4.5292	2.62E-07	0.02880215	NA	NA	NA	NA	No	NA
ENSG00000163946	FAM208A	3	56658507	56717265	ENSG00000163737	PF4	4	74846794	74847841	0.0094274	5.7447	2.63E-07	0.02888211	0.055076	7.6105	6.05E-09	1.13E-05	Yes	No
ENSG00000172123	SLFN12	17	33738079	33760302	ENSG00000198876	DCAF12	9	34086385	34127397	0.012327	4.0123	2.63E-07	0.02888211	0.0045658	0.69948	0.65011	1	No	NA
ENSG00000011405	PIK3C2A	11	17108119	17229530	ENSG00000177627	C12orf54	12	48876286	48890295	0.0098719	5.3485	2.63E-07	0.02888211	NA	NA	NA	NA	No	NA
ENSG00000187664	HAPLN4	19	19366450	19373605	ENSG00000108309	RUNDC3A	17	42385927	42396039	0.0062464	15.195	2.64E-07	0.02894674	0.016089	3.7487	0.0049357	1	Yes	No
ENSG00000132139	GAS2L2	17	34071530	34079897	ENSG00000177666	PNPLA2	11	818902	825573	0.0094254	5.7436	2.64E-07	0.02895428	0.0082857	0.95351	0.47131	1	Yes	No
ENSG00000154760	SLFN13	17	33762115	33775856	ENSG00000101335	MYL9	20	35169887	35178228	0.010721	4.7546	2.65E-07	0.02905058	0.016008	1.2323	0.25544	1	Yes	No
ENSG00000177096	FAM109B	22	42470255	42475445	ENSG00000105464	GRIN2D	19	48898132	48948188	0.0089638	6.241	2.66E-07	0.02905438	0.0070596	0.92834	0.48349	1	No	NA
ENSG00000177096	FAM109B	22	42470255	42475445	ENSG00000069696	DRD4	11	637293	640706	0.0089638	6.2409	2.66E-07	0.02905438	0.0058622	0.76994	0.61267	1	No	NA

ENSG00000177096	FAM109B	22	42470255	42475445	ENSG00000166573	GALR1	18	74962505	74980858	0.008964	6.2411	2.65E-07	0.02905438	NA	NA	NA	NA	No	NA
ENSG00000106009	BRAT1	7	2577511	2595361	ENSG00000150636	CCDC102B	18	66383765	66722426	0.0089627	6.2402	2.66E-07	0.0291034	0.0067648	0.88931	0.51419	1	No	NA
ENSG00000163947	ARHGEF3	3	56761446	57113357	ENSG00000198948	MFAP3L	4	170907748	170954182	0.0094207	5.7406	2.67E-07	0.02915893	0.011268	1.3006	0.23937	1	No	NA
ENSG00000138303	ASCC1	10	73856278	73976892	ENSG00000162931	TRIM17	1	228595641	228604562	0.0098627	5.3435	2.68E-07	0.02927667	0.0078808	1.0372	0.40319	1	No	NA
ENSG00000139330	KERA	12	91444268	91451760	ENSG00000134440	NARS	18	55267888	55289445	0.0062397	15.179	2.68E-07	0.02927963	0.00095317	0.4384	0.6452	1	No	NA
ENSG00000141543	EIF4A3	17	78109013	78120982	ENSG00000187173	LCE2A	1	152670840	152671918	0.0098616	5.3429	2.69E-07	0.02931096	NA	NA	NA	NA	No	NA
ENSG00000139330	KERA	12	91444268	91451760	ENSG00000169764	UGP2	2	64068074	64118696	0.0062382	15.176	2.69E-07	0.02935099	0.00025194	0.1158	0.89067	1	No	NA
ENSG00000148334	PTGES2	9	130882972	130887720	ENSG00000164821	DEFA4	8	6793344	6795860	0.0079793	7.7732	2.70E-07	0.02943894	0.007718	1.4249	0.21268	1	Yes	No
ENSG00000132591	ERAL1	17	27183282	27188085	ENSG00000154114	TBCEL	11	120894781	120961484	0.0068745	11.154	2.72E-07	0.02961287	0.00080621	0.2469	0.86357	1	No	NA
ENSG00000205413	SAMD9	7	92728829	92747336	ENSG00000198171	DDRGK1	20	3170996	3185331	0.0068735	11.152	2.72E-07	0.02966574	0.0006276	0.19216	0.90176	1	No	NA
ENSG00000180376	CCDC66	3	56591189	56653929	ENSG00000085733	CTTN	11	70244510	70282690	0.007975	7.769	2.73E-07	0.0296772	0.0035824	0.65866	0.65495	1	Yes	No
ENSG00000108733	PEX12	17	33901814	33905882	ENSG00000124214	STAU1	20	47729878	47804904	0.0079743	7.7683	2.73E-07	0.02969464	0.0043426	0.79903	0.55043	1	No	NA
ENSG00000108924	HLF	17	53342373	53402426	ENSG00000156689	GLYATL2	11	58601542	58671688	0.0074448	9.0627	2.73E-07	0.02969464	NA	NA	NA	NA	No	NA
ENSG00000131467	PSME3	17	40976402	40995774	ENSG00000237541	HLA-DQA2	6	32709119	32714992	0.0068712	11.148	2.74E-07	0.02976148	0.0046855	1.4405	0.22952	1	No	NA
ENSG00000172660	TAF15	17	34136459	34174246	ENSG00000110693	SOX6	11	15987995	16761138	0.0084721	6.8797	2.74E-07	0.02976794	0.0050179	0.76908	0.59432	1	No	NA
ENSG00000139330	KERA	12	91444268	91451760	ENSG00000135040	NAA35	9	88556061	88637213	0.0062306	15.157	2.74E-07	0.02976794	0.0007682	0.35326	0.70249	1	No	NA
ENSG00000139330	KERA	12	91444268	91451760	ENSG00000113441	LNPEP	5	96294155	96373219	0.0062292	15.153	2.75E-07	0.02981333	0.0050171	2.317	0.099145	1	No	NA
ENSG00000139330	KERA	12	91444268	91451760	ENSG00000153898	MCOLN2	1	85391268	85462796	0.006229	15.153	2.75E-07	0.02981333	0.0020254	0.93257	0.39391	1	No	NA
ENSG00000004059	ARF5	7	127228399	127231142	ENSG00000089737	DDX24	14	94517268	94547591	0.0084706	6.8785	2.75E-07	0.02981333	0.0033247	0.50871	0.80206	1	No	NA
ENSG00000115616	SLC9A2	2	103236166	103327777	ENSG00000181541	MAB21L2	4	151503077	151505843	0.0084711	6.8789	2.75E-07	0.02981333	NA	NA	NA	NA	No	NA
ENSG00000108733	PEX12	17	33901814	33905882	ENSG00000187017	ESPN	1	6484848	6521040	0.0079698	7.7639	2.76E-07	0.02985435	0.0063304	1.1671	0.32346	1	Yes	No
ENSG00000108733	PEX12	17	33901814	33905882	ENSG00000171914	TLN2	15	62682725	63136830	0.0079699	7.764	2.76E-07	0.02985435	0.0075636	1.3962	0.22318	1	No	NA
ENSG00000185379	RAD51D	17	33426811	33447063	ENSG00000128266	GNAZ	22	23412540	23464889	0.0068674	11.142	2.76E-07	0.02988942	0.0064913	1.9993	0.11251	1	No	NA
ENSG00000123636	BAZ2B	2	160175490	160473203	ENSG00000146426	TIAM2	6	155181872	155575707	0.010278	5.0129	2.77E-07	0.02994608	0.028432	2.9654	0.0017525	1	No	NA
ENSG00000185379	RAD51D	17	33426811	33447063	ENSG00000153162	BMP6	6	7727030	7880334	0.0068655	11.139	2.78E-07	0.02999296	0.0020328	0.62331	0.60003	1	No	NA
ENSG00000108733	PEX12	17	33901814	33905882	ENSG00000151474	FRMD4A	10	13693903	14504141	0.0079667	7.7609	2.78E-07	0.02999983	0.013771	2.558	0.026124	1	No	NA
ENSG00000176040	TMPRSS7	3	111753690	111800116	ENSG00000078114	NEBL	10	21068902	21463116	0.0068645	11.137	2.78E-07	0.03003044	0.0015174	0.69831	0.49769	1	No	NA
ENSG00000138378	STAT4	2	191894302	192016322	ENSG00000173085	COQ2	4	84182689	84206067	0.0079653	7.7594	2.79E-07	0.03006749	0.0067762	1.0404	0.3974	1	No	NA
ENSG00000117013	KCNQ4	1	41249684	41306124	ENSG00000138829	FBN2	5	127593601	127994878	0.0089407	6.2247	2.79E-07	0.03009803	0.019981	2.6621	0.0099155	1	No	NA
ENSG00000139437	TCHP	12	110338079	110355874	ENSG00000013374	NUB1	7	151038785	151071710	0.0089398	6.2241	2.80E-07	0.03013026	0.01037	1.598	0.14451	1	No	NA
ENSG00000006125	AP2B1	17	33913918	34053436	ENSG00000159023	EPB41	1	29213603	29442315	0.0089394	6.2239	2.80E-07	0.03013026	0.005424	0.83167	0.54544	1	No	NA
ENSG00000233608	TWIST2	2	239756673	239795893	ENSG00000180219	FAM71C	12	100041531	100043824	0.0079635	7.7577	2.80E-07	0.03013026	NA	NA	NA	NA	No	NA
ENSG00000101350	KIF3B	20	30865467	30922814	ENSG00000036448	MYOM2	8	1993155	2113475	0.0079622	7.7564	2.81E-07	0.03019004	0.0047807	0.73256	0.62346	1	No	NA
ENSG00000112539	C6orf118	6	165693153	165723096	ENSG00000197953	AADACL2	3	151451704	151479127	0.0062208	15.133	2.81E-07	0.03019004	NA	NA	NA	NA	No	NA
ENSG00000142082	SIRT3	11	215458	236431	ENSG00000102362	SYTL4	X	99929488	99987110	0.0068598	11.13	2.81E-07	0.03023895	0.0036912	0.84935	0.49405	1	Yes	Yes
ENSG00000090487	SPG21	15	65255362	65282648	ENSG00000103056	SMPD3	16	68397399	68482591	0.0068596	11.129	2.82E-07	0.03023895	2.05E-05	0.0094286	0.99062	1	No	NA
ENSG00000139437	TCHP	12	110338079	110355874	ENSG00000127022	CANX	5	179125407	179157926	0.008936	6.2214	2.82E-07	0.03024725	0.0081247	1.2492	0.27867	1	No	NA
ENSG00000124749	COL21A1	6	55921388	56258892	ENSG00000111653	ING4	12	6759446	6772314	0.0089361	6.2215	2.82E-07	0.03024725	0.0058077	0.76275	0.61876	1	No	NA
ENSG00000205045	SLFN12L	17	33800708	33864880	ENSG00000172270	BSG	19	571297	583493	0.0089363	6.2217	2.82E-07	0.03024725	0.0040337	0.52883	0.81313	1	No	NA
ENSG00000205413	SAMD9	7	92728829	92747336	ENSG00000078061	ARAF	X	47420516	47430411	0.0068569	11.125	2.83E-07	0.03037185	0.003816	1.1722	0.31924	1	No	NA
ENSG00000180376	CCDC66	3	56591189	56653929	ENSG00000259207	ITGB3	17	45387505	45389182	0.0079571	7.7514	2.84E-07	0.03039169	0.018405	3.4351	0.004417	1	Yes	No
ENSG00000178057	NDUFAF3	3	49057892	49060928	ENSG00000120937	NPPB	1	11917521	11918988	0.0074278	9.0418	2.84E-07	0.03039169	NA	NA	NA	NA	No	NA
ENSG00000197134	ZNF257	19	22235254	22274282	ENSG00000100191	SLC5A4	22	32614465	32651328	0.0062161	15.121	2.84E-07	0.03039169	NA	NA	NA	NA	No	NA
ENSG00000143933	CALM2	2	47272677	47403740	ENSG00000106565	TMEM176B	7	150488373	150498448	0.0068549	11.122	2.85E-07	0.03045284	0.00040296	0.12335	0.94633	1	No	NA
ENSG00000101017	CD40	20	44746911	44758502	ENSG00000082293	COL19A1	6	70576463	70919679	0.0089302	6.2174	2.86E-07	0.0305321	0.01222	1.6153	0.12734	1	Yes	Yes
ENSG00000140521	POLG	15	89860607	89878092	ENSG00000181915	ADO	10	64564516	64568238	0.0068486	11.112	2.89E-07	0.03085687	0.0020972	0.64309	0.58742	1	No	NA
ENSG00000121904	CSMD2	1	33979609	34631443	ENSG00000002933	TMEM176A	7	150498622	150502208	0.01109	4.5091	2.89E-07	0.03085687	0.010422	0.79775	0.65317	1	No	NA
ENSG00000089486	CDIP1	16	4560676	4588829	ENSG00000144026	ZNF514	2	95818419	95831158	0.0062087	15.103	2.89E-07	0.03085687	4.82E-05	0.022166	0.97808	1	No	NA
ENSG00000139437	TCHP	12	110338079	110355874	ENSG00000146386	ABRACL	6	139349819	139364439	0.0089244	6.2133	2.89E-07	0.03085687	NA	NA	NA	NA	No	NA
ENSG00000126351	THRA	17	38214543	38248880	ENSG00000134755	DSC2	18	28645940	28682378	0.007948	7.7425	2.90E-07	0.03088446	0.0065521	0.86116	0.53686	1	Yes	No
ENSG00000167395	ZNF646	16	31085743	31086082	ENSG00000130511	SSBP4	19	18529674	18544652	0.0079481	7.7425	2.90E-07	0.03088446	0.01005	1.5481	0.15937	1	No	NA
ENSG00000119778	ATAD2B	2	23971534	24149984	ENSG00000088826	SMOX	20	4101627	4168394	0.006847	11.109	2.90E-07	0.03089077	0.012613	3.9089	0.0086485	1	No	NA
ENSG00000113356	POLR3G	5	89767565	89810370	ENSG00000131873	CHSY1	15	101715928	101792137	0.008922	6.2116	2.91E-07	0.03092138	0.0093212	1.2285	0.284	1	No	NA
ENSG00000088826	SMOX	20	4101627	4168394	ENSG00000145335	SNCA	4	90645250	90759466	0.0098242	5.3225	2.91E-07	0.03092138	0.0097946	1.0023	0.43625	1	No	NA
ENSG00000139330	KERA	12	91444268	91451760	ENSG00000135341	MAP3K7	6	91223292	91296764	0.0062064	15.098	2.91E-07	0.03092138	0.0013571	0.62444	0.53579	1	No	NA
ENSG00000138061	CYP1B1	2	38294652	38337044	ENSG00000136819	C9orf78	9	132589569	132598142	0.0093796	5.7154	2.91E-07	0.03095106	0.008336	0.95934	0.46658	1	No	NA
ENSG00000172716	SLFN11	17	33677324	33700720	ENSG00000204463	BAG6	6	31606805	31620170	0.011084	4.5068	2.92E-07	0.03101653	0.016969	1.3076	0.20818	1	Yes	No
ENSG00000177464	GPR4	19	46093022	46103011	ENSG00000113140	SPARC	5	151040657	151066726	0.0084435	6.8563	2.92E-07	0.03101653	0.0031721	0.41551	0.89303	1	No	NA
ENSG00000154760	SLFN13	17	33762115	33775856	ENSG00000103342	GSPT1	16	11961985	12009939	0.010674	4.7333	2.92E-07	0.03101653	0.0062166	0.47385	0.93046	1	No	NA
ENSG00000140983	RHOT2	16	718086	724174	ENSG00000177098	SCN4B	11	118004092	118023603	0.008443	6.8559	2.92E-07	0.03101957	0.0095857	1.2637	0.26527	1	No	NA
ENSG00000139437	TCHP	12	110338079	110355874	ENSG00000117625	RCOR3	1	211431719	211489727	0.0089183	6.209	2.93E-07	0.03107562	0.0061674	0.94636	0.4608	1	No	NA
ENSG00000130764	LRRC47	1	3696784	3713068	ENSG00000147852	VLDLR	9	2621834	2654480	0.0098182	5.3191	2.94E-07	0.03118142	0.010912	1.259	0.26153	1	No	NA
ENSG00000023909	GCLM	1	94350761	94374966	ENSG00000138738	PRDM5	4	121606074	121844025	0.010248	4.9979	2.95E-07	0.03125189	0.01486	1.3742	0.18718	1	No	NA
ENSG00000139330	KERA	12	91444268	91451760	ENSG00000071073	MGAT4A	2	99235569	99347589	0.0062002	15.082	2.95E-07	0.03125189	0.0012959	0.59623	0.5511	1	No	NA
ENSG00000015532	XYLT2	17	48423453	48440499	ENSG00000104853	CLPTM1	19	45457848	45496599	0.0089145	6.2063	2.96E-07	0.03126644	0.0028224	0.43164	0.85805	1	No	NA
ENSG00000167552	TUBA1A	12	49578579	49581241	ENSG00000115085	ZAP70	2	98330023	98356325	0.0084379	6.8517	2.96E-07	0.0312778	0.006419	1.1835	0.31524	1	No	NA
ENSG00000196376	SLC35F1	6	118228689	118638839	ENSG00000165272	AQP3	9	33441152	33447609	0.0089122	6.2047	2.97E-07	0.03138956	0.0070679	1.0855	0.36913	1	No	NA

ENSG00000139330	KERA	12	91444268	91451760	ENSG00000159593	NAE1	16	66836778	66907159	0.0061972	15.075	2.97E-07	0.03141141	0.0024139	1.1119	0.32938	1	No	NA
ENSG00000172123	SLFN12	17	33738079	33760302	ENSG00000198574	SH2D1B	1	162365056	162381928	0.012263	3.9911	2.98E-07	0.03145753	0.016647	2.5816	0.017406	1	No	NA
ENSG00000139330	KERA	12	91444268	91451760	ENSG00000033178	UBA6	4	68481479	68566897	0.0061956	15.071	2.98E-07	0.03149937	0.0055137	2.5476	0.078822	1	No	NA
ENSG00000130182	ZSCAN10	16	3138891	3149318	ENSG00000159882	ZNF230	19	44507100	44518078	0.0061938	15.067	3.00E-07	0.03162661	0.044528	21.414	8.13E-10	1.53E-06	No	NA
ENSG00000171357	LURAP1	1	46669006	46669296	ENSG00000204188	GGNBP1	6	33551515	33556803	0.0074035	9.012	3.00E-07	0.03165778	NA	NA	NA	NA	No	NA
ENSG00000177963	RIC8A	11	207511	215113	ENSG00000128266	GNAZ	22	23412540	23464889	0.007932	7.7268	3.00E-07	0.03165847	0.013619	1.5757	0.1279	1	Yes	Yes
ENSG00000143390	RFX5	1	151313116	151319833	ENSG00000134765	DSC1	18	28709199	28742819	0.0079316	7.7264	3.01E-07	0.03165847	0.015593	2.902	0.013153	1	No	NA
ENSG00000188511	C22orf34	22	49808176	50051190	ENSG00000176893	OR51G2	11	4935900	4936922	0.0079316	7.7264	3.01E-07	0.03165847	NA	NA	NA	NA	No	NA
ENSG00000100211	CBY1	22	39052645	39069859	ENSG00000146242	TPBG	6	83072923	83080545	0.0054122	26.316	3.01E-07	0.03169272	4.35E-05	0.03999	0.84154	1	No	NA
ENSG00000139330	KERA	12	91444268	91451760	ENSG00000116560	SFPQ	1	35641979	35658749	0.0061912	15.06	3.02E-07	0.03173009	0.0052977	2.4473	0.087093	1	No	NA
ENSG00000139330	KERA	12	91444268	91451760	ENSG00000112249	ASCC3	6	100956070	101329248	0.0061907	15.059	3.02E-07	0.03175165	0.0039766	1.8345	0.16027	1	No	NA
ENSG00000158485	CD1B	1	158297741	158301321	ENSG00000182264	IZUMO1	19	49244145	49250166	0.0068287	11.079	3.03E-07	0.03181311	NA	NA	NA	NA	No	NA
ENSG00000164400	CSF2	5	131409483	131411859	ENSG00000204316	MRPL38	17	73896389	73901494	0.006187	15.05	3.05E-07	0.03198726	0.00021899	0.20151	0.65361	1	No	NA
ENSG00000180438	TPRXL	3	13978756	14124311	ENSG00000135127	CCDC64	12	120427673	120532298	0.0068261	11.075	3.05E-07	0.03198726	0.00075229	0.23038	0.87526	1	No	NA
ENSG00000108733	PEX12	17	33901814	33905882	ENSG00000204613	TRIM10	6	30119722	30128711	0.0079242	7.7191	3.06E-07	0.03207578	0.0097881	1.8109	0.10808	1	Yes	No
ENSG00000001617	SEMA3F	3	50192478	50226508	ENSG00000175691	ZNF77	19	2933216	2944969	0.0079235	7.7184	3.06E-07	0.03210305	0.0063667	1.1739	0.32007	1	Yes	No
ENSG00000154760	SLFN13	17	33762115	33775856	ENSG00000130830	MPP1	X	154006959	154049282	0.01065	4.7226	3.07E-07	0.03210305	0.019661	1.5192	0.11123	1	No	NA
ENSG00000139330	KERA	12	91444268	91451760	ENSG00000082898	XPO1	2	61704984	61765761	0.0061847	15.045	3.07E-07	0.03210305	0.0043382	2.0021	0.13564	1	No	NA
ENSG00000116774	OLFML3	1	114522063	114524876	ENSG00000104671	DCTN6	8	30013813	30041156	0.0097966	5.3073	3.08E-07	0.03222064	0.014329	1.4731	0.15321	1	No	NA
ENSG00000204296	C6orf10	6	32256303	32339684	ENSG00000137878	GCOM1	15	57938335	57993912	0.0054027	26.269	3.09E-07	0.03226095	8.44E-05	0.077623	0.78061	1	No	NA
ENSG00000204296	C6orf10	6	32256303	32339684	ENSG00000263155	MYZAP	15	57891609	57891901	0.0054027	26.269	3.09E-07	0.03226095	NA	NA	NA	NA	No	NA
ENSG00000172660	TAF15	17	34136459	34174246	ENSG00000120885	CLU	8	27454434	27472548	0.008418	6.8354	3.09E-07	0.03229805	0.0097733	1.5051	0.17323	1	Yes	No
ENSG00000163947	ARHGEF3	3	56761446	57113357	ENSG00000041353	RAB27B	18	52385091	52562747	0.0093514	5.698	3.09E-07	0.03229805	0.01013	1.1679	0.31552	1	No	NA
ENSG00000131097	HIGD1B	17	42923721	42926756	ENSG00000126838	PZP	12	9301436	9360966	0.0061791	15.031	3.11E-07	0.03242899	0.0029826	1.3746	0.25346	1	No	NA
ENSG00000163964	PIGX	3	196439229	196462878	ENSG00000100092	SH3BP1	22	38030661	38052124	0.0079172	7.7122	3.11E-07	0.03242899	0.0039583	0.51889	0.82073	1	No	NA
ENSG00000164163	ABCE1	4	146025538	146046253	ENSG00000085491	SLC25A24	1	108676658	108743471	0.0053983	26.248	3.12E-07	0.03254078	0.0022602	2.0841	0.14918	1	No	NA
ENSG00000204444	APOM	6	31623248	31625987	ENSG00000101425	BPI	20	36888551	36965907	0.0084133	6.8316	3.12E-07	0.03255028	0.022829	3.5628	0.0017049	1	Yes	Yes
ENSG00000204516	MICB	6	31462658	31478901	ENSG00000134765	DSC1	18	28709199	28742819	0.0073859	8.9904	3.13E-07	0.03258479	0.0048195	1.4819	0.21795	1	No	NA
ENSG00000123146	CD97	19	14491313	14519537	ENSG00000135314	KHDC1	6	73951037	74020088	0.0084104	6.8292	3.14E-07	0.03272861	0.003882	0.59431	0.73507	1	No	NA
ENSG00000139330	KERA	12	91444268	91451760	ENSG00000117174	ZNHIT6	1	86115106	86174116	0.0061742	15.019	3.14E-07	0.03272968	0.0052858	2.4417	0.087574	1	No	NA
ENSG00000137944	CCBL2	1	89401456	89435150	ENSG00000198673	FAM19A2	12	62102040	62672931	0.0068113	11.051	3.16E-07	0.03282854	0.00095415	0.43885	0.64491	1	No	NA
ENSG00000139330	KERA	12	91444268	91451760	ENSG00000143924	EML4	2	42396490	42559688	0.0061721	15.014	3.16E-07	0.03286907	0.00016577	0.076182	0.92665	1	No	NA
ENSG00000139437	TCHP	12	110338079	110355874	ENSG00000183307	CECR6	22	17597189	17602257	0.0088818	6.1833	3.17E-07	0.03291769	0.007537	1.1581	0.32666	1	No	NA
ENSG00000178184	PARD6G	18	77915115	78005429	ENSG00000083444	PLOD1	1	11994262	12035595	0.0097824	5.2996	3.17E-07	0.03291769	0.010399	0.95735	0.47942	1	No	NA
ENSG00000198682	PAPSS2	10	89419370	89507462	ENSG00000004399	PLXND1	3	129274018	129325661	0.008882	6.1835	3.17E-07	0.03291769	0.0048902	0.74941	0.60997	1	No	NA
ENSG00000176595	KBTBD11	8	1922044	1955102	ENSG00000144820	GPR128	3	100328433	100414323	0.009783	5.2999	3.17E-07	0.03291769	NA	NA	NA	NA	No	NA
ENSG00000184786	TCTE3	6	170140210	170151655	ENSG00000186073	C15orf41	15	36871812	37102449	0.0073794	8.9825	3.17E-07	0.03292385	0.0015962	0.36651	0.83256	1	No	NA
ENSG00000139330	KERA	12	91444268	91451760	ENSG00000134987	WDR36	5	110427414	110466200	0.00617	15.009	3.18E-07	0.03293104	0.0018085	0.8325	0.43529	1	No	NA
ENSG00000225697	SLC26A6	3	48663156	48672926	ENSG00000100228	RAB36	22	23487513	23506537	0.0073789	8.9818	3.18E-07	0.03293304	0.0027591	0.63427	0.63814	1	No	NA
ENSG00000181666	HKR1	19	37803739	37860267	ENSG00000116962	NID1	1	236139130	236228462	0.0093362	5.6887	3.19E-07	0.03307366	0.036692	4.347	3.67E-05	0.0652159	No	NA
ENSG00000139330	KERA	12	91444268	91451760	ENSG00000111880	RNGTT	6	89319985	89673348	0.0061676	15.003	3.20E-07	0.03307366	0.00074787	0.3439	0.70909	1	No	NA
ENSG00000166595	FAM96B	16	66965959	66968303	ENSG00000197766	CFD	19	859453	863453	0.0073752	8.9773	3.21E-07	0.03316353	0.0046601	1.0733	0.36844	1	No	NA
ENSG00000139330	KERA	12	91444268	91451760	ENSG00000017260	ATP2C1	3	130569439	130721994	0.0061656	14.998	3.21E-07	0.03320056	0.0016081	0.74013	0.47733	1	No	NA
ENSG00000006125	AP2B1	17	33913918	34053436	ENSG00000183508	FAM46C	1	118148556	118170994	0.0088751	6.1786	3.22E-07	0.03326444	0.012151	1.8758	0.082085	1	Yes	Yes
ENSG00000139330	KERA	12	91444268	91451760	ENSG00000143643	TTC13	1	231041989	231114621	0.0061643	14.995	3.22E-07	0.03327866	0.0019452	0.89557	0.40873	1	No	NA
ENSG00000139330	KERA	12	91444268	91451760	ENSG00000062194	GPBP1	5	56469775	56560506	0.0061637	14.993	3.23E-07	0.03330319	0.0020342	0.93661	0.39233	1	No	NA
ENSG00000172123	SLFN12	17	33738079	33760302	ENSG00000166169	POLL	10	103338639	103346957	0.012221	3.9774	3.23E-07	0.03333183	0.013958	2.1588	0.044816	1	Yes	No
ENSG00000142444	C19orf52	19	11039409	11044211	ENSG00000002933	TMEM176A	7	150498622	150502208	0.0083974	6.8186	3.23E-07	0.03335087	0.0099595	1.5341	0.16378	1	No	NA
ENSG00000015532	XYLT2	17	48423453	48440499	ENSG00000100982	PCIF1	20	44563267	44576662	0.0088727	6.177	3.24E-07	0.03335087	0.0055909	0.8574	0.52583	1	No	NA
ENSG00000099849	RASSF7	11	560404	564021	ENSG00000185532	PRKG1	10	52750945	54058110	0.0088712	6.1759	3.25E-07	0.03344848	NA	NA	NA	NA	No	NA
ENSG00000228008	CTD-2330K9.3	3	49943495	49954370	ENSG00000162493	PDPN	1	13909960	13944452	0.006796	11.026	3.27E-07	0.03369738	NA	NA	NA	NA	No	NA
ENSG00000175643	RMI2	16	11343506	11445619	ENSG00000237541	HLA-DQA2	6	32709119	32714992	0.0067956	11.025	3.27E-07	0.0337103	0.0053433	1.6438	0.17768	1	No	NA
ENSG00000137757	CASP5	11	104864962	104893895	ENSG00000184611	KCNH7	2	163229980	163695240	0.0083899	6.8125	3.29E-07	0.03383124	0.0047036	0.72069	0.633	1	No	NA
ENSG00000182118	FAM89A	1	231154704	231175992	ENSG00000109861	CTSC	11	88026760	88070955	0.0088615	6.1691	3.31E-07	0.0340878	0.0037854	0.49615	0.83777	1	No	NA
ENSG00000163946	FAM208A	3	56658507	56717265	ENSG00000166333	ILK	11	6624961	6631511	0.0093184	5.6778	3.32E-07	0.03409332	0.01827	2.4299	0.01806	1	Yes	No
ENSG00000145147	SLIT2	4	20254883	20622184	ENSG00000071991	CDH19	18	64168320	64271375	0.01061	4.7049	3.32E-07	0.03411937	NA	NA	NA	NA	No	NA
ENSG00000187257	RSBN1L	7	77325760	77409008	ENSG00000010932	FMO1	1	171217638	171255117	0.0073589	8.9573	3.33E-07	0.03417826	NA	NA	NA	NA	No	NA
ENSG00000139330	KERA	12	91444268	91451760	ENSG00000146802	TMEM168	7	112405718	112430647	0.0061504	14.961	3.33E-07	0.03419501	0.0026459	1.219	0.296	1	No	NA
ENSG00000132139	GAS2L2	17	34071530	34079897	ENSG00000104522	TSTA3	8	144694788	144700218	0.0093145	5.6753	3.35E-07	0.03431227	0.0057597	0.66114	0.72605	1	No	NA
ENSG00000268358	FLJ11235	5	111755280	111756310	ENSG00000169249	ZRSR2	X	15808595	15841383	0.0078836	7.6793	3.35E-07	0.03435457	0.0013568	0.31147	0.8704	1	No	NA
ENSG00000075651	PLD1	3	171318195	171528740	ENSG00000185247	MAGEA11	X	148769894	148798926	0.0093132	5.6745	3.35E-07	0.03437326	NA	NA	NA	NA	No	NA
ENSG00000168995	SIGLEC7	19	51645556	51656783	ENSG00000002933	TMEM176A	7	150498622	150502208	0.0093123	5.674	3.36E-07	0.03442265	0.0046434	0.60912	0.74874	1	No	NA
ENSG00000188163	FAM166A	9	140138623	140142222	ENSG00000157542	KCNJ6	21	38996789	39288749	0.010182	4.9656	3.38E-07	0.03457539	NA	NA	NA	NA	No	NA
ENSG00000148337	CIZ1	9	130928343	130966662	ENSG00000135953	MFSD9	2	103332299	103353347	0.010181	4.9651	3.38E-07	0.0346318	0.0046339	0.85288	0.51242	1	No	NA
ENSG00000142082	SIRT3	11	215458	236431	ENSG00000164116	GUCY1A3	4	156587863	156653501	0.0067812	11.001	3.39E-07	0.03465031	0.025069	5.8949	0.00010995	0.19318215	Yes	Yes
ENSG00000258839	MC1R	16	89978527	89981576	ENSG00000133250	ZNF414	19	8575462	8579044	0.011808	4.1164	3.39E-07	0.03465041	0.013321	1.7628	0.091392	1	No	NA
ENSG00000139330	KERA	12	91444268	91451760	ENSG00000162924	REL	2	61108656	61150645	0.0061423	14.941	3.40E-07	0.03470676	0.0024611	1.1337	0.3223	1	No	NA

ENSG00000136379	ABHD17C	15	80972025	81047962	ENSG00000111653	ING4	12	6759446	6772314	0.010179	4.9641	3.40E-07	0.03470676	0.012054	1.1115	0.3501	1	No	NA
ENSG00000167077	MEI1	22	42095503	42195460	ENSG00000143870	PDIA6	2	10927407	10978103	0.006142	14.94	3.40E-07	0.03471193	0.0029988	0.9204	0.43037	1	No	NA
ENSG00000172992	DCAKD	17	43100708	43138473	ENSG00000120314	WDR55	5	140044261	140048833	0.0067783	10.997	3.41E-07	0.03480279	0.0012319	0.37743	0.7693	1	No	NA
ENSG00000092871	RFFL	17	33341759	33416338	ENSG00000158578	ALAS2	X	55039919	55057497	0.008372	6.7977	3.42E-07	0.03488438	0.0068943	0.79228	0.60953	1	No	NA
ENSG00000158485	CD1B	1	158297741	158301321	ENSG00000065883	CDK13	7	39989636	40136733	0.0067762	10.993	3.43E-07	0.0349261	0.0010643	0.32602	0.80656	1	No	NA
ENSG00000177096	FAM109B	22	42470255	42475445	ENSG00000122863	CHST3	10	73724123	73773322	0.0088461	6.1583	3.43E-07	0.0349261	NA	NA	NA	NA	No	NA
ENSG00000254667	AP000783.1	11	123301058	123306427	ENSG00000123728	RAP2C	X	131337053	131353471	0.011797	4.1125	3.46E-07	0.03525999	0.022196	2.0679	0.024559	1	No	NA
ENSG00000204305	AGER	6	32148745	32150978	ENSG00000089177	KIF16B	20	16252749	16554078	0.0092965	5.6643	3.48E-07	0.0353623	0.018031	1.8607	0.054347	1	No	NA
ENSG00000173714	WFIKKN2	17	48912011	48919709	ENSG00000120708	TGFBI	5	135364584	135399507	0.0083647	6.7918	3.48E-07	0.0353623	0.0031204	0.57344	0.72042	1	No	NA
ENSG00000204444	APOM	6	31623248	31625987	ENSG00000128641	MYO1B	2	192109911	192290115	0.0083643	6.7914	3.48E-07	0.03536762	0.01171	1.807	0.094709	1	No	NA
ENSG00000139330	KERA	12	91444268	91451760	ENSG00000081019	RSBN1	1	114304454	114355098	0.0061323	14.916	3.48E-07	0.03536762	0.004484	2.0697	0.12681	1	No	NA
ENSG00000158485	CD1B	1	158297741	158301321	ENSG00000084676	NCOA1	2	24714783	24993571	0.0067696	10.982	3.48E-07	0.03536762	0.004112	1.2635	0.28568	1	No	NA
ENSG00000165355	FBXO33	14	39866873	39901704	ENSG00000212710	CTAGE1	18	19993564	19997878	0.0088374	6.1521	3.49E-07	0.03545567	0.026096	3.4986	0.0010363	1	No	NA
ENSG00000139330	KERA	12	91444268	91451760	ENSG00000100883	SRP54	14	35451163	35498773	0.0061303	14.912	3.50E-07	0.03547838	0.0067718	3.1328	0.04406	1	No	NA
ENSG00000139330	KERA	12	91444268	91451760	ENSG00000115421	PAPOLG	2	60983365	61029220	0.0061305	14.912	3.50E-07	0.03547838	0.0015592	0.71756	0.48822	1	No	NA
ENSG00000181192	DHTKD1	10	12110971	12165224	ENSG00000163879	DNALI1	1	38022520	38027846	0.0083616	6.7892	3.50E-07	0.03548516	NA	NA	NA	NA	No	NA
ENSG00000168374	ARF4	3	57557090	57583947	ENSG00000166091	CMTM5	14	23846017	23848981	0.0078635	7.6595	3.51E-07	0.03552537	0.056114	10.891	3.39E-10	6.37E-07	Yes	No
ENSG00000139899	CBLN3	14	24895738	24897611	ENSG00000125534	PPDPF	20	62152077	62153559	0.0053497	26.01	3.53E-07	0.03569818	0.0063908	5.9174	0.015182	1	No	NA
ENSG00000099849	RASSF7	11	560404	564021	ENSG00000185245	GP1BA	17	4835592	4838325	0.008831	6.1477	3.54E-07	0.03582479	0.016945	2.2507	0.02838	1	Yes	No
ENSG00000099769	IGFALS	16	1840414	1844972	ENSG00000131504	DIAPH1	5	140894583	140998622	0.0073322	8.9246	3.54E-07	0.03582479	0.0011634	0.17762	0.98293	1	No	NA
ENSG00000132394	EEFSEC	3	127965679	128127485	ENSG00000204420	C6orf25	6	31691121	31694491	0.0083537	6.7828	3.56E-07	0.03599707	0.0074874	1.382	0.22853	1	No	NA
ENSG00000131097	HIGD1B	17	42923721	42926756	ENSG00000148943	LIN7C	11	27516123	27528320	0.0061228	14.893	3.56E-07	0.03599707	0.002382	1.0972	0.33425	1	No	NA
ENSG00000001617	SEMA3F	3	50192478	50226508	ENSG00000162753	SLC9C2	1	173469603	173572233	0.0078563	7.6525	3.56E-07	0.03599707	NA	NA	NA	NA	No	NA
ENSG00000164171	ITGA2	5	52285156	52390609	ENSG00000106565	TMEM176B	7	150488373	150498448	0.010156	4.9525	3.57E-07	0.03601871	0.0074106	0.68015	0.74363	1	No	NA
ENSG00000120137	PANK3	5	167975500	168006605	ENSG00000173085	COQ2	4	84182689	84206067	0.0083515	6.781	3.58E-07	0.03611337	0.0045054	0.82912	0.52901	1	No	NA
ENSG00000174599	TRAM1L1	4	118004718	118006736	ENSG00000146700	SRCRB4D	7	76018651	76039012	0.0061208	14.888	3.58E-07	0.03611337	0.00058362	0.26833	0.76472	1	No	NA
ENSG00000163933	RFT1	3	53122499	53164478	ENSG00000111678	C12orf57	12	7052596	7055166	0.0061202	14.887	3.59E-07	0.03615306	0.0020563	0.94684	0.38834	1	No	NA
ENSG00000008838	MED24	17	38175350	38217468	ENSG00000171236	LRG1	19	4536421	4539196	0.0073262	8.9172	3.59E-07	0.03616026	0.010505	2.4339	0.045896	1	No	NA
ENSG00000122483	CCDC18	1	93648917	93744287	ENSG00000089327	FXYD5	19	35645633	35660786	0.0083492	6.7791	3.60E-07	0.03616026	0.011372	1.502	0.16284	1	No	NA
ENSG00000139437	TCHP	12	110338079	110355874	ENSG00000134371	CDC73	1	193091147	193223031	0.0088242	6.1429	3.59E-07	0.03616026	0.0068503	1.0519	0.39009	1	No	NA
ENSG00000156113	KCNMA1	10	78629359	79398353	ENSG00000165240	ATP7A	X	77166194	77305892	0.010152	4.9506	3.60E-07	0.03616026	0.0086187	0.79199	0.63663	1	No	NA
ENSG00000172660	TAF15	17	34136459	34174246	ENSG00000171552	BCL2L1	20	30252255	30311792	0.0083495	6.7793	3.60E-07	0.03616026	0.003975	0.6086	0.72361	1	No	NA
ENSG00000183726	TMEM50A	1	25664951	25683344	ENSG00000185885	IFITM1	11	313506	314456	0.010152	4.9508	3.59E-07	0.03616026	0.0035287	0.46237	0.86197	1	No	NA
ENSG00000167552	TUBA1A	12	49578759	49581241	ENSG00000000457	SCYL3	1	169823411	169863408	0.0083474	6.7776	3.61E-07	0.03623055	0.011851	2.1971	0.052619	1	No	NA
ENSG00000109919	MTCH2	11	47638867	47664175	ENSG00000162946	DISC1	1	231762561	232177018	0.0067544	10.958	3.61E-07	0.03623055	0.0027302	0.83773	0.47329	1	No	NA
ENSG00000204991	SPIRE2	16	89884587	89937727	ENSG00000165171	WBSCR27	7	73248920	73256865	0.0078503	7.6465	3.61E-07	0.03623055	0.0063803	0.83843	0.55545	1	No	NA
ENSG00000101347	SAMHD1	20	35526225	35580246	ENSG00000160307	S100B	21	48018875	48025121	0.0067538	10.957	3.62E-07	0.03623055	0.0014825	0.45433	0.7143	1	No	NA
ENSG00000161904	LEMD2	6	33738979	33756913	ENSG00000182985	CADM1	11	115039938	115375675	0.0073231	8.9133	3.62E-07	0.03623055	0.0017508	0.3213	0.90031	1	No	NA
ENSG00000109919	MTCH2	11	47638867	47664175	ENSG00000270106	TRAX	1	231752390	231752652	0.0067544	10.958	3.61E-07	0.03623055	NA	NA	NA	NA	No	NA
ENSG00000115866	DARS	2	136664247	136743670	ENSG00000146072	TNFRSF21	6	47199268	47277641	0.0088209	6.1406	3.62E-07	0.03626092	0.032925	5.1921	2.89E-05	0.0515865	No	NA
ENSG00000109743	BST1	4	15704573	15739936	ENSG00000107929	LARP4B	10	855484	977564	0.0088192	6.1394	3.63E-07	0.03636661	0.0077363	1.4283	0.21146	1	No	NA
ENSG00000188368	PRR19	19	42811105	42814973	ENSG00000004838	ZMYND10	3	50378541	50384283	0.0073208	8.9106	3.63E-07	0.03636661	0.0015656	0.35947	0.83751	1	No	NA
ENSG00000177951	BET1L	11	167784	207428	ENSG00000204420	C6orf25	6	31691121	31694491	0.0092744	5.6507	3.64E-07	0.03643888	0.028543	2.4306	0.0054996	1	Yes	Yes
ENSG00000139330	KERA	12	91444268	91451760	ENSG00000120253	NUP43	6	150045451	150067814	0.0061135	14.87	3.65E-07	0.0364411	0.0022029	1.0144	0.36301	1	No	NA
ENSG00000004059	ARF5	7	127228399	127231142	ENSG00000044574	HSPA5	9	127997132	128003609	0.0083421	6.7733	3.66E-07	0.03652225	0.0064538	0.99061	0.43025	1	No	NA
ENSG00000179913	B3GNT3	19	17905637	17923891	ENSG00000170677	SOCS6	18	67956137	67997436	0.0073178	8.907	3.66E-07	0.03654241	0.01072	1.6526	0.12964	1	No	NA
ENSG00000106635	BCL7B	7	72950686	72972332	ENSG00000163751	CPA3	3	148583043	148614983	0.0061112	14.865	3.67E-07	0.0365875	0.0068965	3.191	0.041588	1	Yes	No
ENSG00000108733	PEX12	17	33901814	33905882	ENSG00000182732	RGS6	14	72399156	73030654	0.0078429	7.6393	3.67E-07	0.03665051	0.0048628	0.89522	0.48357	1	No	NA
ENSG00000108306	FBXL20	17	37415384	37558776	ENSG00000137757	CASP5	11	104864962	104893895	0.0078408	7.6372	3.69E-07	0.0367873	0.0082336	1.5209	0.1806	1	Yes	No
ENSG00000171603	CLSTN1	1	9791180	9884584	ENSG00000134489	HRH4	18	22040593	22059921	0.0092685	5.647	3.69E-07	0.0367873	0.0033598	0.51409	0.79797	1	No	NA
ENSG00000108733	PEX12	17	33901814	33905882	ENSG00000211584	SLC48A1	12	48147699	48172978	0.00784	7.6364	3.70E-07	0.03683916	0.0047818	0.88024	0.49367	1	No	NA
ENSG00000241258	CRCP	7	65579591	65615935	ENSG00000237541	HLA-DQA2	6	32709119	32714992	0.0078386	7.6351	3.71E-07	0.03693578	0.0064038	1.1807	0.31664	1	No	NA
ENSG00000177951	BET1L	11	167784	207428	ENSG00000164116	GUCY1A3	4	156587863	156653501	0.0092649	5.6448	3.72E-07	0.03699344	0.030556	2.6074	0.002832	1	Yes	Yes
ENSG00000140506	LMAN1L	15	75105057	75118099	ENSG00000038945	MSR1	8	15965387	16424999	0.0083343	6.7669	3.72E-07	0.03699344	0.0064674	0.9927	0.42883	1	No	NA
ENSG00000205045	SLFN12L	17	33800708	33864880	ENSG00000124491	F13A1	6	6144318	6321246	0.0088072	6.131	3.73E-07	0.03707891	0.0031377	0.41098	0.89586	1	Yes	Yes
ENSG00000197992	CLEC9A	12	10183276	10218565	ENSG00000180537	RNF182	6	13924677	13980533	0.0083326	6.7655	3.73E-07	0.03709562	0.0082293	1.2654	0.27072	1	No	NA
ENSG00000139437	TCHP	12	110338079	110355874	ENSG00000143155	TIPRL	1	168148171	168169950	0.0088065	6.1305	3.73E-07	0.03709562	0.0079886	1.2281	0.28927	1	No	NA
ENSG00000168374	ARF4	3	57557090	57583947	ENSG00000151693	ASAP2	2	9346894	9541525	0.0078345	7.6311	3.74E-07	0.03716701	0.0097046	1.7953	0.11118	1	Yes	No
ENSG00000168404	MLKL	16	74705753	74734858	ENSG00000170448	NFXL1	4	47849257	47913011	0.010961	4.4559	3.75E-07	0.03717878	0.013829	2.569	0.025562	1	No	NA
ENSG00000006125	AP2B1	17	33913918	34053436	ENSG00000179526	SHARPIN	8	145153536	145159172	0.008801	6.1266	3.78E-07	0.03749013	0.017702	2.7482	0.011862	1	Yes	Yes
ENSG00000131791	PRKAB2	1	146626685	146644129	ENSG00000166987	MBD6	12	57914493	57922792	0.0083265	6.7605	3.78E-07	0.0375057	0.0046804	0.71711	0.63589	1	No	NA
ENSG00000163491	NEK10	3	27151576	27410951	ENSG00000078114	NEBL	10	21068902	21463116	0.0083253	6.7596	3.79E-07	0.03758469	0.004119	0.63074	0.70576	1	No	NA
ENSG00000103343	ZNF174	16	3451235	3459370	ENSG00000159882	ZNF230	19	44507100	44518078	0.0060961	14.828	3.80E-07	0.03765035	0.03251	15.44	2.54E-07	0.00046761	No	NA
ENSG00000007312	CD79B	17	62006100	62009714	ENSG00000135951	TSGA10	2	99613724	99771099	0.0083243	6.7587	3.80E-07	0.03765035	0.0091747	1.4121	0.20682	1	No	NA
ENSG00000184949	FAM227A	22	38974125	39052634	ENSG00000163064	EN1	2	119599747	119605254	0.0073005	8.8857	3.81E-07	0.03768462	NA	NA	NA	NA	No	NA
ENSG00000095139	ARCN1	11	118443105	118473748	ENSG00000170448	NFXL1	4	47849257	47913011	0.0083232	6.7578	3.81E-07	0.03768819	0.0068731	1.0554	0.38785	1	No	NA
ENSG00000162814	SPATA17	1	217804666	218045038	ENSG00000136842	TMOD1	9	100263462	100353717	0.0072994	8.8844	3.82E-07	0.03770383	0.0078165	1.806	0.12551	1	No	NA

ENSG00000162814	SPATA17	1	217804666	218045038	ENSG00000136925	TSTD2	9	100366351	100389894	0.0072994	8.8844	3.82E-07	0.03770383	0.0055874	1.2881	0.27288	1	No	NA
ENSG00000170275	CRTAP	3	33155471	33189265	ENSG00000166851	PLK1	16	23690143	23700997	0.0092529	5.6374	3.81E-07	0.03770383	0.0074712	0.85907	0.5508	1	No	NA
ENSG00000205045	SLFN12L	17	33800708	33864880	ENSG00000256269	HMBS	11	118955576	118964259	0.0087956	6.1228	3.82E-07	0.0377204	0.0037144	0.48681	0.8446	1	Yes	Yes
ENSG00000234127	TRIM26	6	30152232	30181204	ENSG00000161381	PLXDC1	17	37219556	37310647	0.0087955	6.1227	3.82E-07	0.0377204	0.0092631	1.2208	0.28825	1	No	NA
ENSG00000111196	MAGOHB	12	10758612	10766222	ENSG00000189221	MAOA	X	43515467	43606068	0.0087956	6.1228	3.82E-07	0.0377204	0.008846	1.1653	0.32012	1	No	NA
ENSG00000164076	CAMKV	3	49895422	49907655	ENSG00000131059	BPIFA3	20	31805116	31815564	0.0067297	10.917	3.83E-07	0.03772296	NA	NA	NA	NA	No	NA
ENSG00000196376	SLC35F1	6	118228689	118638839	ENSG00000090861	AARS	16	70286198	70323446	0.0087937	6.1215	3.84E-07	0.03782799	0.015767	2.443	0.023845	1	No	NA
ENSG00000158485	CD1B	1	158297741	158301321	ENSG00000149927	DOC2A	16	30017917	30034591	0.0067272	10.913	3.85E-07	0.03791519	0.0023345	0.71603	0.54251	1	No	NA
ENSG00000139330	KERA	12	91444268	91451760	ENSG00000119912	IDE	10	94211441	94333833	0.0060905	14.814	3.85E-07	0.03792306	0.0025831	1.19	0.30468	1	No	NA
ENSG00000139330	KERA	12	91444268	91451760	ENSG00000173692	PSMD1	2	231921578	232037541	0.0060905	14.814	3.85E-07	0.03792306	0.00069647	0.32025	0.72605	1	No	NA
ENSG00000139330	KERA	12	91444268	91451760	ENSG00000102858	MGRN1	16	4666494	4740975	0.00609	14.813	3.86E-07	0.03795797	9.65E-05	0.04436	0.95661	1	No	NA
ENSG00000139330	KERA	12	91444268	91451760	ENSG00000135249	RINT1	7	105172532	105204394	0.0060885	14.809	3.87E-07	0.03807049	0.0014482	0.66643	0.51378	1	No	NA
ENSG00000154768	C17orf50	17	34087916	34092098	ENSG00000130830	MPP1	X	154006959	154049282	0.0083148	6.7509	3.88E-07	0.03812043	0.014501	1.9213	0.063217	1	No	NA
ENSG00000139437	TCHP	12	110338079	110355874	ENSG00000152795	HNRNPDL	4	83343717	83351294	0.0087886	6.1179	3.88E-07	0.03812043	NA	NA	NA	NA	No	NA
ENSG00000177946	CENPBD1	16	90036206	90038942	ENSG00000133250	ZNF414	19	8575462	8579044	0.0092439	5.6319	3.89E-07	0.03815714	0.0087588	1.1538	0.32709	1	No	NA
ENSG00000108733	PEX12	17	33901814	33905882	ENSG00000111666	CHPT1	12	102090725	102137918	0.0078158	7.6127	3.90E-07	0.03830369	0.0030655	0.56333	0.72818	1	No	NA
ENSG00000112276	BVES	6	105544697	105585049	ENSG00000166200	COPS2	15	49398268	49437275	0.0072886	8.8712	3.91E-07	0.03837656	0.0052296	0.9631	0.43941	1	No	NA
ENSG00000114554	PLXNA1	3	126707437	126756235	ENSG00000110315	RNF141	11	10533225	10562777	0.0083082	6.7455	3.94E-07	0.0386034	0.0019492	0.29783	0.93804	1	No	NA
ENSG00000139330	KERA	12	91444268	91451760	ENSG00000185658	BRWD1	21	40556102	40693485	0.0060814	14.792	3.94E-07	0.0386034	1.18E-06	0.00054012	0.99946	1	No	NA
ENSG00000139330	KERA	12	91444268	91451760	ENSG00000112697	TMEM30A	6	75962640	75994684	0.0060802	14.789	3.95E-07	0.03870343	0.0009688	0.44559	0.64058	1	No	NA
ENSG00000099849	RASSF7	11	560404	564021	ENSG00000174175	SELP	1	169558087	169599431	0.0087779	6.1104	3.97E-07	0.03872727	0.0029109	0.38119	0.91362	1	Yes	No
ENSG00000186075	ZPBP2	17	38024417	38034149	ENSG00000110080	ST3GAL4	11	126225535	126310239	0.0053032	25.783	3.96E-07	0.03872727	0.0021759	2.0062	0.15699	1	Yes	No
ENSG00000172123	SLFN12	17	33738079	33760302	ENSG00000167992	VWCE	11	61025762	61062896	0.012114	3.9421	3.97E-07	0.03872727	0.0047535	0.72837	0.62683	1	Yes	No
ENSG00000108883	EFTUD2	17	42929078	42973357	ENSG00000198952	SMG5	1	156219015	156252616	0.0060794	14.787	3.96E-07	0.03872727	0.0095046	4.4093	0.012423	1	No	NA
ENSG00000142945	KIF2C	1	45205490	45233439	ENSG00000106305	AIMP2	7	6048876	6057676	0.0087787	6.111	3.97E-07	0.03872727	0.013456	1.7809	0.087693	1	No	NA
ENSG00000137310	TCF19	6	31126319	31131992	ENSG00000160613	PCSK7	11	117077397	117103241	0.0087775	6.1101	3.98E-07	0.03872727	0.011352	1.4992	0.1638	1	No	NA
ENSG00000150628	SPATA4	4	177105789	177116822	ENSG00000157168	NRG1	8	31496902	32622548	0.0087775	6.1101	3.98E-07	0.03872727	0.0094717	1.2486	0.27323	1	No	NA
ENSG00000139330	KERA	12	91444268	91451760	ENSG00000133657	ATP13A3	3	194123401	194219093	0.0060779	14.783	3.97E-07	0.03872727	0.0020657	0.95117	0.38667	1	No	NA
ENSG00000177096	FAM109B	22	42470255	42475445	ENSG00000157388	CACNA1D	3	53528683	53846490	0.0087772	6.1099	3.98E-07	0.03872727	0.0077403	1.0185	0.41634	1	No	NA
ENSG00000205045	SLFN12L	17	33800708	33864880	ENSG00000105610	KLF1	19	12995237	12997995	0.0087786	6.1109	3.97E-07	0.03872727	0.0069298	0.91115	0.49691	1	No	NA
ENSG00000139330	KERA	12	91444268	91451760	ENSG00000156171	DRAM2	1	111659955	111681745	0.0060774	14.782	3.98E-07	0.03872727	0.00029548	0.13581	0.87302	1	No	NA
ENSG00000187492	CDHR4	3	49828165	49837268	ENSG00000204290	BTNL2	6	32361740	32374905	0.0060792	14.786	3.96E-07	0.03872727	NA	NA	NA	NA	No	NA
ENSG00000186075	ZPBP2	17	38024417	38034149	ENSG00000254607	RP11-115C1(	11	126522747	126551999	0.0053032	25.783	3.96E-07	0.03872727	NA	NA	NA	NA	No	NA
ENSG00000156853	ZNF689	16	30613879	30635333	ENSG00000198121	LPAR1	9	113635543	113800981	0.0067107	10.886	4.00E-07	0.03894039	0.0013548	0.41514	0.74217	1	No	NA
ENSG00000177096	FAM109B	22	42470255	42475445	ENSG00000185551	NR2F2	15	96869167	96883492	0.0087739	6.1076	4.01E-07	0.03896275	NA	NA	NA	NA	No	NA
ENSG00000139330	KERA	12	91444268	91451760	ENSG00000136518	ACTL6A	3	179280668	179304420	0.0060741	14.774	4.01E-07	0.03898703	0.00047908	0.22024	0.80237	1	No	NA
ENSG00000180376	CCDC66	3	56591189	56653929	ENSG00000161911	TREML1	6	41117080	41122075	0.0077991	7.5963	4.05E-07	0.03921783	0.012473	2.3139	0.042057	1	Yes	No
ENSG00000118640	VAMP8	2	85788685	85809154	ENSG00000112053	SLC26A8	6	35911291	35992645	0.0082967	6.7361	4.04E-07	0.03921783	0.030191	4.0648	0.00021127	0.36866615	No	NA
ENSG00000185379	RAD51D	17	33426811	33447063	ENSG00000007968	E2F2	1	23832922	23857712	0.0067067	10.88	4.04E-07	0.03921783	0.006729	2.073	0.10221	1	No	NA
ENSG00000108733	PEX12	17	33901814	33905882	ENSG00000049323	LTBP1	2	33172039	33624576	0.0077988	7.596	4.06E-07	0.03921783	0.0084929	1.5692	0.16612	1	No	NA
ENSG00000177096	FAM109B	22	42470255	42475445	ENSG00000168282	MGAT2	14	50087489	50090198	0.0087688	6.104	4.05E-07	0.03921783	0.0098522	1.2992	0.24737	1	No	NA
ENSG00000172139	SLC9C1	3	111859734	112013105	ENSG00000078114	NEBL	10	21068902	21463116	0.0072731	8.8521	4.06E-07	0.03921783	0.0037754	0.86879	0.48209	1	No	NA
ENSG00000153339	TRAPPC8	18	29409136	29533099	ENSG00000112782	CLIC5	6	45868045	46048132	0.0077992	7.5964	4.05E-07	0.03921783	0.0048187	0.88705	0.48906	1	No	NA
ENSG00000167077	MEI1	22	42095503	42195460	ENSG00000163939	PBRM1	3	52579368	52719933	0.0060696	14.763	4.06E-07	0.03921783	0.001588	0.4867	0.69159	1	No	NA
ENSG00000144224	UBXN4	2	136499189	136542625	ENSG00000130511	SSBP4	19	18529674	18544652	0.0082952	6.7349	4.05E-07	0.03921783	0.0022723	0.34732	0.91161	1	No	NA
ENSG00000139330	KERA	12	91444268	91451760	ENSG00000117133	RPF1	1	84944942	84963473	0.0060706	14.765	4.05E-07	0.03921783	0.00014718	0.067639	0.9346	1	No	NA
ENSG00000177096	FAM109B	22	42470255	42475445	ENSG00000188483	IER5L	9	131937835	131940540	0.0087688	6.104	4.05E-07	0.03921783	0.0019915	0.26055	0.96879	1	No	NA
ENSG00000121101	TEX14	17	56634039	56769416	ENSG00000165181	C9orf84	9	114448453	114557288	0.0082943	6.7341	4.06E-07	0.03924336	0.014005	1.621	0.11466	1	No	NA
ENSG00000165966	PDZRN4	12	41582250	41968384	ENSG00000105048	TNNT1	19	55644162	55660722	0.010092	4.9213	4.07E-07	0.03924336	0.0068593	0.6292	0.78965	1	No	NA
ENSG00000139330	KERA	12	91444268	91451760	ENSG00000112081	SRSF3	6	36562145	36573377	0.0060684	14.76	4.07E-07	0.03924336	0.00040844	0.18775	0.82885	1	No	NA
ENSG00000163833	FBXO40	3	121311966	121349139	ENSG00000158022	TRIM63	1	26377795	26394927	0.007272	8.8507	4.07E-07	0.03924336	NA	NA	NA	NA	No	NA
ENSG00000213171	LINGO4	1	151772740	151775193	ENSG00000137801	THBS1	15	39873280	39891667	0.0082927	6.7329	4.08E-07	0.03930494	0.002507	0.38328	0.88995	1	No	NA
ENSG00000134240	HMGCS2	1	120290619	120311528	ENSG00000157551	KCNJ15	21	39628663	39673748	0.0077965	7.5938	4.08E-07	0.03930863	0.0044701	0.8226	0.53362	1	No	NA
ENSG00000142082	SIRT3	11	215458	236431	ENSG00000117400	MPL	1	43803478	43818443	0.0067021	10.872	4.09E-07	0.0393653	0.0038327	0.88203	0.47406	1	Yes	Yes
ENSG00000108733	PEX12	17	33901814	33905882	ENSG00000168497	SDPR	2	192699028	192711981	0.0077936	7.5909	4.10E-07	0.03949075	0.0092658	1.7134	0.12884	1	Yes	No
ENSG00000091164	TXNL1	18	54264439	54314766	ENSG00000006282	SPATA20	17	48623667	48633213	0.0067005	10.87	4.10E-07	0.03949075	0.0051587	1.5867	0.19101	1	No	NA
ENSG00000004534	RBM6	3	49977440	50114683	ENSG00000164270	HTR4	5	147830595	148056798	0.0060648	14.751	4.10E-07	0.03949075	NA	NA	NA	NA	No	NA
ENSG00000103024	NME3	16	1820882	1821731	ENSG00000196465	MYL6B	12	56546040	56551771	0.008762	6.0992	4.11E-07	0.03954531	0.0042861	0.56205	0.78705	1	No	NA
ENSG00000170234	PWWP2A	5	159488808	159546430	ENSG00000115607	IL18RAP	2	103035149	103069025	0.0092167	5.6152	4.12E-07	0.03960078	0.011153	1.2871	0.24638	1	No	NA
ENSG00000165280	VCP	9	35056061	35073246	ENSG00000118495	PLAGL1	6	144261437	144385735	0.0066944	10.86	4.16E-07	0.03997327	0.0059021	1.8168	0.14245	1	No	NA
ENSG00000168010	ATG16L2	11	72525353	72540680	ENSG00000082397	EPB41L3	18	5392383	5630699	0.0077873	7.5848	4.16E-07	0.03998047	0.0033797	0.62127	0.68362	1	No	NA
ENSG00000196214	ZNF766	19	52772824	52795977	ENSG00000115750	TAF1B	2	9983483	10074545	0.0082823	6.7244	4.17E-07	0.04002363	0.0053093	0.97785	0.43017	1	No	NA
ENSG00000228008	CTD-2330K9.3	3	49943495	49954370	ENSG00000163357	DCST1	1	155006300	155021740	0.0066933	10.858	4.17E-07	0.04002363	0.0022404	0.51477	0.7249	1	No	NA
ENSG00000139330	KERA	12	91444268	91451760	ENSG00000131437	KIF3A	5	132028320	132073330	0.006057	14.732	4.18E-07	0.04010751	7.18E-07	0.00033008	0.99967	1	No	NA
ENSG00000182979	MTA1	14	105886159	105937066	ENSG00000138744	NAAA	4	76831809	76861413	0.0077833	7.5808	4.20E-07	0.04026897	0.0068063	1.0451	0.39441	1	No	NA
ENSG00000052850	ALX4	11	44281994	44331716	ENSG00000105755	ETHE1	19	44010871	44031396	0.0082786	6.7213	4.20E-07	0.04028559	0.0035217	0.53896	0.77881	1	No	NA
ENSG00000196935	SRGAP1	12	64238073	64541613	ENSG00000137878	GCOM1	15	57938335	57993912	0.0096473	5.2256	4.21E-07	0.04028721	0.0046736	0.86023	0.50734	1	No	NA

ENSG00000196935	SRGAP1	12	64238073	64541613	ENSG00000263155	MYZAP	15	57891609	57891901	0.0096473	5.2256	4.21E-07	0.04028721	NA	NA	NA	NA	No	NA
ENSG00000092871	RFFL	17	33341759	33416338	ENSG00000143774	GUK1	1	228327663	228336685	0.0082763	6.7194	4.23E-07	0.04043488	0.013902	1.6089	0.11808	1	Yes	No
ENSG00000172123	SLFN12	17	33738079	33760302	ENSG00000123908	AGO2	8	141541264	141645718	0.012081	3.931	4.24E-07	0.04050518	NA	NA	NA	NA	Yes	No
ENSG00000139330	KERA	12	91444268	91451760	ENSG00000164118	CEP44	4	175206585	175254531	0.0060516	14.719	4.24E-07	0.04050518	NA	NA	NA	NA	No	NA
ENSG00000178401	DNAJC22	12	49740700	49751309	ENSG00000181904	C5orf24	5	134181370	134182553	0.0087475	6.089	4.24E-07	0.04054362	0.016477	2.1875	0.0332	1	No	NA
ENSG00000139330	KERA	12	91444268	91451760	ENSG00000169139	UBE2V2	8	48920960	48977268	0.0060507	14.717	4.25E-07	0.04055338	0.0031042	1.4308	0.23964	1	No	NA
ENSG00000172660	TAF15	17	34136459	34174246	ENSG00000159335	PTMS	12	6874682	6880116	0.008273	6.7168	4.26E-07	0.04062899	0.012077	1.8642	0.084096	1	No	NA
ENSG00000139330	KERA	12	91444268	91451760	ENSG00000100567	PSMA3	14	58711549	58738730	0.0060491	14.713	4.26E-07	0.04067591	0.00058532	0.26911	0.76412	1	No	NA
ENSG00000086730	LAT2	7	73613982	73644161	ENSG00000149534	MS4A2	11	59855734	59863444	0.0072501	8.8239	4.28E-07	0.04078573	0.001402	0.21411	0.9724	1	No	NA
ENSG00000139437	TCHP	12	110338079	110355874	ENSG00000162613	FUBP1	1	78409740	78444794	0.0087425	6.0856	4.29E-07	0.04086976	0.012198	1.8832	0.08082	1	No	NA
ENSG00000127947	PTPN12	7	77166592	77269388	ENSG00000010932	FMO1	1	171217638	171255117	0.0087425	6.0855	4.29E-07	0.04086976	NA	NA	NA	NA	No	NA
ENSG00000124019	FAM124B	2	225243415	225266802	ENSG00000196526	AFAP1	4	7760441	7874040	0.0091958	5.6023	4.31E-07	0.04101362	0.0096726	1.2753	0.25932	1	No	NA
ENSG00000147457	CHMP7	8	23101150	23119512	ENSG00000100362	PVALB	22	37196728	37215523	0.010064	4.9073	4.31E-07	0.04102884	0.0069778	0.71206	0.69823	1	No	NA
ENSG00000119778	ATAD2B	2	23971534	24149984	ENSG00000095303	PTGS1	9	125132824	125157982	0.0066782	10.833	4.32E-07	0.04112777	0.0040846	1.255	0.28864	1	No	NA
ENSG00000257218	GATC	12	120884241	120892833	ENSG00000177042	TMEM80	11	695616	705028	0.0072451	8.8178	4.33E-07	0.04114381	0.002785	0.64024	0.63389	1	No	NA
ENSG00000178252	WDR6	3	49045041	49052151	ENSG00000103023	PRSS54	16	58318309	58328951	0.0060429	14.697	4.33E-07	0.04114381	NA	NA	NA	NA	No	NA
ENSG00000172660	TAF15	17	34136459	34174246	ENSG00000144567	FAM134A	2	220042939	220050201	0.0082644	6.7097	4.34E-07	0.04122164	0.0205	3.1917	0.0041736	1	Yes	No
ENSG00000141150	RASL10B	17	34058668	34070540	ENSG00000008441	NFIX	19	13106422	13209610	0.0091919	5.5999	4.34E-07	0.04123578	0.011637	1.5373	0.15096	1	No	NA
ENSG00000006125	AP2B1	17	33913918	34053436	ENSG00000198478	SH3BGRL2	6	80341000	80413372	0.0087359	6.0809	4.35E-07	0.04128584	0.016252	2.5194	0.020058	1	Yes	Yes
ENSG00000004059	ARF5	7	127228399	127231142	ENSG00000134684	YARS	1	33240840	33277248	0.0082632	6.7087	4.35E-07	0.04128584	0.0035475	0.54292	0.77573	1	No	NA
ENSG00000154760	SLFN13	17	33762115	33775856	ENSG00000183508	FAM46C	1	118148556	118170994	0.010477	4.645	4.36E-07	0.0413178	0.0095587	0.73106	0.72172	1	Yes	Yes
ENSG00000139330	KERA	12	91444268	91451760	ENSG00000156831	NSMCE2	8	126106710	126379362	0.00604	14.69	4.36E-07	0.0413178	0.00043322	0.19915	0.81946	1	No	NA
ENSG00000177666	PNPLA2	11	818902	825573	ENSG00000205038	PKHD1L1	8	110374706	110542559	0.0077659	7.5637	4.37E-07	0.04139155	0.010072	1.5516	0.1583	1	Yes	No
ENSG00000139437	TCHP	12	110338079	110355874	ENSG00000166562	SEC11C	18	56806709	56826068	0.0087328	6.0787	4.38E-07	0.04146596	0.0095803	1.4751	0.18352	1	No	NA
ENSG00000127074	RGS13	1	192605275	192629390	ENSG00000185198	PRSS57	19	685521	695460	0.006038	14.686	4.38E-07	0.04146596	NA	NA	NA	NA	No	NA
ENSG00000198453	ZNF568	19	37407231	37488834	ENSG00000116962	NID1	1	236139130	236228462	0.0087304	6.077	4.40E-07	0.04163546	0.016237	1.8837	0.059236	1	No	NA
ENSG00000164258	NDUFS4	5	52856463	52979168	ENSG00000179869	ABCA13	7	48211055	48687092	0.0096257	5.2138	4.40E-07	0.04163546	0.0090889	0.92946	0.49842	1	No	NA
ENSG00000070019	GUCY2C	12	14765576	14849519	ENSG00000184489	PTP4A3	8	142402093	142441620	0.0087303	6.0769	4.40E-07	0.04163546	0.0013496	0.17646	0.99003	1	No	NA
ENSG00000168374	ARF4	3	57557090	57583947	ENSG00000204420	C6orf25	6	31691121	31694491	0.0077619	7.5598	4.41E-07	0.04165595	0.017902	3.3395	0.0053828	1	No	NA
ENSG00000172123	SLFN12	17	33738079	33760302	ENSG00000137193	PIM1	6	37137979	37143202	0.012057	3.9234	4.43E-07	0.04173891	0.015273	2.3653	0.028391	1	Yes	No
ENSG00000168899	VAMP5	2	85811531	85820535	ENSG00000096063	SRPK1	6	35806114	35889119	0.010051	4.9008	4.43E-07	0.04173891	0.03668	3.4688	0.00017132	0.30015264	No	NA
ENSG00000155393	HEATR3	16	50099852	50140298	ENSG00000105426	PTPRS	19	5158506	5340814	0.0052594	25.569	4.43E-07	0.04173891	0.00049631	0.22817	0.79603	1	No	NA
ENSG00000196166	C8orf86	8	38368352	38386180	ENSG00000212900	KRTAP3-2	17	39155447	39156138	0.0066685	10.817	4.42E-07	0.04173891	NA	NA	NA	NA	No	NA
ENSG00000177096	FAM109B	22	42470255	42475445	ENSG00000179528	LBX2	2	74724644	74730443	0.0087282	6.0755	4.42E-07	0.04173891	NA	NA	NA	NA	No	NA
ENSG00000105227	PRX	19	40899675	40919273	ENSG00000144820	GPR128	3	100328433	100414323	0.0082555	6.7024	4.42E-07	0.04173891	NA	NA	NA	NA	No	NA
ENSG00000174885	NLRP6	11	278365	285359	ENSG00000140479	PCSK6	15	101840818	102065405	0.0087272	6.0748	4.43E-07	0.04176023	0.025339	2.6344	0.0051725	1	Yes	Yes
ENSG00000113073	SLC4A9	5	139739787	139754728	ENSG00000185015	CA13	8	86132816	86196302	0.0077591	7.557	4.44E-07	0.04176646	0.0089775	1.6596	0.14176	1	No	NA
ENSG00000158485	CD1B	1	158297741	158301321	ENSG00000055609	KMT2C	7	151832010	152133090	0.0066668	10.815	4.44E-07	0.04178304	NA	NA	NA	NA	No	NA
ENSG00000139330	KERA	12	91444268	91451760	ENSG00000109680	TBC1D19	4	26578059	26756973	0.0060317	14.67	4.45E-07	0.04180488	0.00039018	0.17936	0.83584	1	No	NA
ENSG00000088826	SMOX	20	4101627	4168394	ENSG00000155749	ALS2CR12	2	202152994	202222121	0.009621	5.2113	4.45E-07	0.04180488	0.0038672	0.3934	0.93861	1	No	NA
ENSG00000136193	SCRN1	7	29959719	30029905	ENSG00000198844	ARHGEF15	17	8213559	8225829	0.0072328	8.8027	4.45E-07	0.0418233	NA	NA	NA	NA	No	NA
ENSG00000106635	BCL7B	7	72950686	72972332	ENSG00000131016	AKAP12	6	151561134	151679692	0.0060306	14.668	4.46E-07	0.04187927	0.0061958	2.8647	0.057508	1	No	NA
ENSG00000108946	PRKAR1A	17	66409764	66547460	ENSG00000197008	ZNF138	7	64254766	64294054	0.0082514	6.699	4.46E-07	0.04191172	0.0034284	0.52462	0.7899	1	No	NA
ENSG00000139330	KERA	12	91444268	91451760	ENSG00000116679	IVNS1ABP	1	185265520	185286461	0.0060297	14.665	4.47E-07	0.04193194	0.0021667	0.99774	0.36911	1	No	NA
ENSG00000139626	ITGB7	12	53585102	53601091	ENSG00000117600	LPPR4	1	99729509	99775146	0.0052545	25.545	4.48E-07	0.04202108	NA	NA	NA	NA	No	NA
ENSG00000164050	PLXNB1	3	48445261	48471594	ENSG00000103023	PRSS54	16	58318309	58328951	0.0072298	8.799	4.48E-07	0.04202108	NA	NA	NA	NA	No	NA
ENSG00000072778	ACADVL	17	7121035	7128592	ENSG00000106780	MEGF9	9	123363091	123476748	0.0082493	6.6973	4.48E-07	0.04202717	0.0049664	0.76116	0.60061	1	No	NA
ENSG00000173230	GOLGB1	3	121382046	121468602	ENSG00000158022	TRIM63	1	26377795	26394927	0.006661	10.805	4.50E-07	0.04216907	NA	NA	NA	NA	No	NA
ENSG00000258839	MC1R	16	89978527	89981576	ENSG00000164626	KCNK5	6	39156749	39197226	0.011661	4.0647	4.51E-07	0.04221441	0.00417	0.54676	0.79916	1	No	NA
ENSG00000164946	FREM1	9	14734664	14910993	ENSG00000100027	YPEL1	22	22057659	22090123	0.010866	4.417	4.53E-07	0.04232306	0.020143	1.5572	0.098686	1	No	NA
ENSG00000139330	KERA	12	91444268	91451760	ENSG00000143753	DEGS1	1	224363458	224381143	0.0060245	14.653	4.53E-07	0.04232306	0.00045829	0.21068	0.81007	1	No	NA
ENSG00000139330	KERA	12	91444268	91451760	ENSG00000170606	HSPA4	5	132387654	132442141	0.0060246	14.653	4.52E-07	0.04232306	0.00011386	0.052324	0.94902	1	No	NA
ENSG00000139330	KERA	12	91444268	91451760	ENSG00000069248	NUP133	1	229577045	229644103	0.0060242	14.652	4.53E-07	0.04232338	0.00085774	0.39447	0.67415	1	No	NA
ENSG00000198917	C9orf114	9	131586026	131592100	ENSG00000118113	MMP8	11	102582526	102597781	0.0072244	8.7924	4.54E-07	0.0423508	0.0078067	2.4076	0.065831	1	Yes	No
ENSG00000139330	KERA	12	91444268	91451760	ENSG00000180488	FAM73A	1	78245309	78344106	0.0060232	14.649	4.54E-07	0.0423508	0.0020077	0.92441	0.39714	1	No	NA
ENSG00000173809	TDRD12	19	33210659	33320483	ENSG00000126838	PZP	12	9301436	9360966	0.0087163	6.0671	4.54E-07	0.0423508	0.0040407	0.6187	0.71547	1	No	NA
ENSG00000139330	KERA	12	91444268	91451760	ENSG00000115760	BIRC6	2	32582096	32843966	0.0060235	14.65	4.54E-07	0.0423508	0.0001203	0.055286	0.94622	1	No	NA
ENSG00000139330	KERA	12	91444268	91451760	ENSG00000152219	ARL14EP	11	30344598	30359774	0.0060221	14.647	4.55E-07	0.04244809	NA	NA	NA	NA	No	NA
ENSG00000243317	C7orf73	7	135347244	135373776	ENSG00000070269	TMEM260	14	56955072	57117324	0.0072227	8.7903	4.56E-07	0.04245954	NA	NA	NA	NA	No	NA
ENSG00000164163	ABCE1	4	146025538	146046253	ENSG00000186998	EMID1	22	29601840	29655586	0.0052475	25.511	4.56E-07	0.04249148	0.0023612	2.1774	0.14039	1	No	NA
ENSG00000184840	TMED9	5	177019159	177023125	ENSG00000196562	SULF2	20	46285656	46415360	0.010035	4.8932	4.57E-07	0.04258298	0.0020078	0.61563	0.60497	1	No	NA
ENSG00000167077	MEI1	22	42095503	42195460	ENSG00000166928	MS4A14	11	60165325	60185161	0.00602	14.641	4.58E-07	0.0425897	0.0032706	1.0041	0.39024	1	No	NA
ENSG00000204991	SPIRE2	16	89884587	89937727	ENSG00000146618	FERD3L	7	19184405	19185044	0.007745	7.5432	4.58E-07	0.04260013	NA	NA	NA	NA	No	NA
ENSG00000158485	CD1B	1	158297741	158301321	ENSG00000137478	FCHSD2	11	72547790	72853306	0.0066524	10.791	4.59E-07	0.04272029	0.00072026	0.22056	0.88215	1	No	NA
ENSG00000196586	MYO6	6	76458909	76629254	ENSG00000124107	SLPI	20	43880880	43883205	0.0087105	6.0631	4.60E-07	0.04272601	0.037423	5.0764	1.14E-05	0.02052	No	NA
ENSG00000172123	SLFN12	17	33738079	33760302	ENSG00000120885	CLU	8	27454434	27472548	0.012038	3.9168	4.60E-07	0.04273093	0.018151	2.8191	0.01006	1	Yes	No
ENSG00000172123	SLFN12	17	33738079	33760302	ENSG00000196961	AP2A1	19	50270225	50309510	0.012038	3.9169	4.60E-07	0.04273093	0.009814	1.5115	0.17113	1	Yes	No

ENSG00000139437	TCHP	12	110338079	110355874	ENSG00000117020	AKT3	1	243651535	244014381	0.0087092	6.0621	4.61E-07	0.04278862	0.0056244	0.86258	0.52193	1	No	NA
ENSG00000132382	MYBBP1A	17	4443643	4458926	ENSG00000144218	AFF3	2	100163718	100759201	0.0077409	7.5392	4.62E-07	0.04287873	0.014353	1.6619	0.10376	1	No	NA
ENSG00000139330	KERA	12	91444268	91451760	ENSG00000132485	ZRANB2	1	71528974	71546980	0.0060149	14.629	4.63E-07	0.04292445	0.0049719	2.296	0.10124	1	No	NA
ENSG00000139437	TCHP	12	110338079	110355874	ENSG00000026950	BTN3A1	6	26402465	26415444	0.0087067	6.0604	4.64E-07	0.04292445	0.007391	1.1355	0.33948	1	No	NA
ENSG00000100918	REC8	14	24641062	24648932	ENSG00000125534	PPDPF	20	62152077	62153559	0.0060147	14.628	4.64E-07	0.04292445	0.0022479	1.0352	0.35556	1	No	NA
ENSG00000139437	TCHP	12	110338079	110355874	ENSG00000111801	BTN3A3	6	26440700	26453643	0.0087067	6.0604	4.64E-07	0.04292445	0.0053105	0.81418	0.55894	1	No	NA
ENSG00000139437	TCHP	12	110338079	110355874	ENSG00000113387	SUB1	5	32531739	32604185	0.0087065	6.0602	4.64E-07	0.04292445	0.0038942	0.59619	0.73357	1	No	NA
ENSG00000116774	OLFML3	1	114522063	114524876	ENSG00000165389	SPTSSA	14	34901995	34931562	0.0096007	5.2001	4.64E-07	0.04292635	NA	NA	NA	NA	No	NA
ENSG00000168995	SIGLEC7	19	51645556	51656783	ENSG00000106565	TMEM176B	7	150488373	150498448	0.0091605	5.5806	4.64E-07	0.04292645	0.0030656	0.40151	0.90167	1	No	NA
ENSG00000174915	PTDSS2	11	448268	491387	ENSG00000165621	OXGR1	13	97637973	97646984	0.011646	4.0594	4.64E-07	0.04292645	NA	NA	NA	NA	No	NA
ENSG00000110921	MVK	12	110012612	110035067	ENSG00000042980	ADAM28	8	24151553	24216531	0.0077369	7.5352	4.66E-07	0.04307525	0.00078863	0.14459	0.98163	1	No	NA
ENSG00000139330	KERA	12	91444268	91451760	ENSG00000162971	TYW5	2	200794698	200813195	0.0060122	14.622	4.66E-07	0.04307525	NA	NA	NA	NA	No	NA
ENSG00000008735	MAPK8IP2	22	51039114	51052409	ENSG00000258986	TMEM179	14	104941015	105071984	0.0082312	6.6825	4.67E-07	0.04309554	NA	NA	NA	NA	No	NA
ENSG00000139437	TCHP	12	110338079	110355874	ENSG00000005249	PRKAR2B	7	106685094	106802256	0.0087027	6.0576	4.68E-07	0.04314073	0.0031495	0.48182	0.82219	1	No	NA
ENSG00000149016	TUT1	11	62342517	62350064	ENSG00000105509	HAS1	19	52216365	52227247	0.0077356	7.5339	4.68E-07	0.04314622	0.0018133	0.33279	0.89326	1	No	NA
ENSG00000103502	CDIPT	16	29869678	29875057	ENSG00000164308	ERAP2	5	96211643	96255420	0.0087022	6.0572	4.68E-07	0.04315165	0.013107	1.3459	0.20889	1	No	NA
ENSG00000139330	KERA	12	91444268	91451760	ENSG00000205339	IPO7	11	9406169	9469673	0.0060105	14.618	4.68E-07	0.04315165	0.0010489	0.48247	0.61741	1	No	NA
ENSG00000171793	CTPS1	1	41445007	41478235	ENSG00000151693	ASAP2	2	9346894	9541525	0.01044	4.6287	4.69E-07	0.04315518	0.012593	1.0551	0.39527	1	No	NA
ENSG00000135063	FAM189A2	9	71939488	72007371	ENSG00000165792	METTL17	14	21457929	21465189	0.010024	4.8873	4.69E-07	0.04315518	NA	NA	NA	NA	No	NA
ENSG00000139437	TCHP	12	110338079	110355874	ENSG00000152092	ASTN1	1	176826438	177134109	0.0087015	6.0567	4.69E-07	0.04315518	NA	NA	NA	NA	No	NA
ENSG00000083817	ZNF416	19	58082934	58089466	ENSG00000100079	LGALS2	22	37966255	37978623	0.0072094	8.774	4.70E-07	0.04321216	0.0051686	1.1911	0.31314	1	No	NA
ENSG00000141150	RASL10B	17	34058668	34070540	ENSG00000104892	KLC3	19	45836692	45852860	0.0091538	5.5765	4.71E-07	0.04328655	0.0075935	0.99909	0.43034	1	No	NA
ENSG00000177096	FAM109B	22	42470255	42475445	ENSG00000165300	SLITRK5	13	88324870	88331871	0.0086991	6.0551	4.71E-07	0.04329839	0.0071529	0.9407	0.47397	1	No	NA
ENSG00000196511	TPK1	7	144149034	144533488	ENSG00000075035	WSCD2	12	108523248	108644314	0.0091525	5.5757	4.72E-07	0.0433736	0.0088591	1.0201	0.41885	1	No	NA
ENSG00000165280	VCP	9	35056061	35073246	ENSG00000152492	CCDC50	3	191074877	191116459	0.0066393	10.77	4.74E-07	0.04348894	0.0031999	0.9823	0.40037	1	No	NA
ENSG00000015532	XYLT2	17	48423453	48440499	ENSG00000185033	SEMA4B	15	90703836	90772908	0.0086965	6.0533	4.74E-07	0.04348894	0.0034555	0.52879	0.78669	1	No	NA
ENSG00000141002	TCF25	16	89940014	89977792	ENSG00000146618	FERD3L	7	19184405	19185044	0.0077295	7.528	4.74E-07	0.04350434	NA	NA	NA	NA	No	NA
ENSG00000177096	FAM109B	22	42470255	42475445	ENSG00000171643	S100Z	5	76145826	76217475	0.0086952	6.0523	4.75E-07	0.04355158	0.013214	1.7485	0.094438	1	No	NA
ENSG00000185924	RTN4RL1	17	1839299	1928178	ENSG00000181754	AMIGO1	1	110046797	110052360	0.0091496	5.5739	4.75E-07	0.04355158	0.0030331	0.27716	0.98615	1	No	NA
ENSG00000139330	KERA	12	91444268	91451760	ENSG00000136874	STX17	9	102668915	102732618	0.0060032	14.6	4.77E-07	0.04366496	0.0045353	2.0935	0.12385	1	No	NA
ENSG00000174915	PTDSS2	11	448268	491387	ENSG00000205038	PKHD1L1	8	110374706	110542559	0.011631	4.0541	4.78E-07	0.04378006	0.0041283	0.95035	0.43408	1	Yes	Yes
ENSG00000167118	URM1	9	131133598	131153015	ENSG00000105993	DNAJB6	7	157128075	157210133	0.0091461	5.5718	4.79E-07	0.04378855	0.014707	1.3598	0.19418	1	No	NA
ENSG00000167118	URM1	9	131133598	131153015	ENSG00000128284	APOL3	22	36536372	36562225	0.0091461	5.5718	4.79E-07	0.04378855	0.014399	1.3309	0.20894	1	No	NA
ENSG00000123500	COL10A1	6	116446502	116479910	ENSG00000144820	GPR128	3	100328433	100414323	0.007201	8.7638	4.79E-07	0.04379278	NA	NA	NA	NA	No	NA
ENSG00000134910	STT3A	11	125461607	125492986	ENSG00000165092	ALDH1A1	9	75515578	75695358	0.0091442	5.5706	4.81E-07	0.04390998	0.011644	1.3446	0.21755	1	No	NA
ENSG00000139330	KERA	12	91444268	91451760	ENSG00000055917	PUM2	2	20448452	20551995	0.006	14.592	4.80E-07	0.04390998	0.00099926	0.45962	0.63167	1	No	NA
ENSG00000172053	QARS	3	49133365	49142553	ENSG00000120937	NPPB	1	11917521	11918988	0.0052259	25.405	4.82E-07	0.04398996	NA	NA	NA	NA	No	NA
ENSG00000100890	KIAA0391	14	35593499	35593616	ENSG00000135929	CYP27A1	2	219646479	219680016	0.0095819	5.1899	4.82E-07	0.04404431	0.013665	1.4039	0.18174	1	Yes	No
ENSG00000172530	BANP	16	87982850	88110924	ENSG00000164930	FZD6	8	104310661	104345094	0.007197	8.7588	4.83E-07	0.04408217	0.02353	5.5242	0.00021383	0.37291952	No	NA
ENSG00000108733	PEX12	17	33901814	33905882	ENSG00000197256	KANK2	19	11274943	11308243	0.0077212	7.5199	4.83E-07	0.04408217	0.0092293	1.7066	0.13041	1	No	NA
ENSG00000006125	AP2B1	17	33913918	34053436	ENSG00000151474	FRMD4A	10	13693903	14504141	0.0086871	6.0466	4.84E-07	0.04408217	0.0023286	0.35594	0.9066	1	No	NA
ENSG00000139330	KERA	12	91444268	91451760	ENSG00000155903	RASA2	3	141205889	141334184	0.0059965	14.584	4.84E-07	0.04414732	0.0049505	2.2861	0.10224	1	No	NA
ENSG00000132139	GAS2L2	17	34071530	34079897	ENSG00000007541	PIGQ	16	619947	634136	0.0091394	5.5677	4.85E-07	0.0441955	0.010543	1.2161	0.28605	1	Yes	No
ENSG00000172057	ORMDL3	17	38077294	38083854	ENSG00000160785	SLC25A44	1	156163880	156182587	0.0077192	7.5179	4.85E-07	0.0441955	0.0035622	0.54518	0.77396	1	No	NA
ENSG00000168374	ARF4	3	57557090	57583947	ENSG00000102362	SYTL4	X	99929488	99987110	0.0077155	7.5142	4.89E-07	0.04453716	0.0062024	1.1434	0.33564	1	No	NA
ENSG00000144681	STAC	3	36421836	36589499	ENSG00000196526	AFAP1	4	7760441	7874040	0.0082084	6.6639	4.91E-07	0.04464657	0.0021258	0.32487	0.92411	1	No	NA
ENSG00000172716	SLFN11	17	33677324	33700720	ENSG00000165406	8-Mar	10	45950035	46090354	0.010825	4.4001	4.92E-07	0.04469586	0.013194	1.0128	0.43457	1	No	NA
ENSG00000198917	C9orf114	9	131586026	131592100	ENSG00000102837	OLFM4	13	53602894	53626192	0.0071891	8.7491	4.92E-07	0.04471801	0.013336	4.1359	0.0063306	1	No	NA
ENSG00000139330	KERA	12	91444268	91451760	ENSG00000007384	RHBDF1	16	108058	126354	0.0059901	14.568	4.92E-07	0.04471801	0.00062256	0.28625	0.75114	1	No	NA
ENSG00000137310	TCF19	6	31126319	31131992	ENSG00000128641	MYO1B	2	192109911	192290115	0.0086778	6.0401	4.93E-07	0.04478006	0.013368	1.7691	0.090099	1	No	NA
ENSG00000136379	ABHD17C	15	80972025	81047962	ENSG00000153823	PID1	2	229715242	230136001	0.0099984	4.875	4.93E-07	0.04478006	0.007433	0.68222	0.74171	1	No	NA
ENSG00000078900	TP73	1	3569084	3652765	ENSG00000169252	ADRB2	5	148206156	148208196	0.0091316	5.5628	4.94E-07	0.04478292	0.0071644	0.82353	0.58187	1	No	NA
ENSG00000180287	PLD5	1	242251689	242687998	ENSG00000213928	IRF9	14	24634266	24634392	0.0077092	7.5081	4.96E-07	0.04501157	0.0012024	0.22055	0.95376	1	No	NA
ENSG00000100418	DESI1	22	41994032	42017100	ENSG00000175556	LONRF3	X	118108581	118156888	0.0086728	6.0366	4.99E-07	0.04515021	0.013765	1.5928	0.12275	1	Yes	Yes
ENSG00000154768	C17orf50	17	34087916	34092098	ENSG00000196914	ARHGEF12	11	120207787	120360645	0.0082013	6.658	4.99E-07	0.04515021	0.018524	2.4643	0.016538	1	No	NA
ENSG00000126062	TMEM115	3	50392180	50392978	ENSG00000165029	ABCA1	9	107543283	107690518	0.0086729	6.0366	4.99E-07	0.04515021	0.012003	1.8527	0.086128	1	No	NA
ENSG00000196154	S100A4	1	153516089	153522612	ENSG00000141337	ARSG	17	66255323	66397591	0.0086734	6.037	4.98E-07	0.04515021	0.0047769	0.87933	0.49428	1	No	NA
ENSG00000188368	PRR19	19	42811105	42814973	ENSG00000243477	NAT6	3	50333833	50335514	0.0071813	8.7395	5.01E-07	0.04532742	0.0070752	1.6336	0.16365	1	No	NA
ENSG00000188368	PRR19	19	42811105	42814973	ENSG00000186792	HYAL3	3	50331063	50333204	0.0071813	8.7395	5.01E-07	0.04532742	0.0009201	0.21113	0.93231	1	No	NA
ENSG00000185619	PCGF3	4	699537	764428	ENSG00000075223	SEMA3C	7	80371854	80551675	0.012371	3.7743	5.03E-07	0.04551355	0.0034954	1.0733	0.35949	1	No	NA
ENSG00000172123	SLFN12	17	33738079	33760302	ENSG00000166947	EPB42	15	43398423	43513481	0.01199	3.9011	5.04E-07	0.04558023	0.013899	2.1495	0.045738	1	Yes	No
ENSG00000119661	DNAL1	14	74111578	74164463	ENSG00000130167	TSPAN16	19	11406824	11437672	0.0081951	6.653	5.05E-07	0.04564956	0.0072758	1.1177	0.34985	1	No	NA
ENSG00000213719	CLIC1	6	31699994	31707540	ENSG00000197561	ELANE	19	851014	856242	0.0071773	8.7347	5.06E-07	0.04565358	0.013494	3.1359	0.014166	1	No	NA
ENSG00000163611	SPICE1	3	113161565	113234034	ENSG00000134326	CMPK2	2	6980701	7006766	0.0086662	6.032	5.06E-07	0.04565358	0.0067767	0.89088	0.51294	1	No	NA
ENSG00000173744	AGFG1	2	228336868	228421384	ENSG00000198816	ZNF358	19	7580178	7581135	0.0081938	6.6518	5.07E-07	0.0457282	0.0016296	0.3742	0.82714	1	No	NA
ENSG00000139330	KERA	12	91444268	91451760	ENSG00000211456	SACM1L	3	45730548	45786916	0.0059774	14.537	5.07E-07	0.04573755	0.0053769	2.484	0.083965	1	No	NA
ENSG00000137310	TCF19	6	31126319	31131992	ENSG00000163803	PLB1	2	28680012	28866654	0.0086648	6.031	5.07E-07	0.04573755	0.0097221	1.2819	0.25599	1	No	NA

ENSG00000139437	TCHP	12	110338079	110355874	ENSG00000135521	LTV1	6	144164481	144184949	0.0086638	6.0303	5.09E-07	0.04580091	0.007244	1.1128	0.35276	1	No	NA
ENSG00000177096	FAM109B	22	42470255	42475445	ENSG00000187730	GABRD	1	1950780	1962192	0.0086637	6.0302	5.09E-07	0.04580091	0.0036947	0.48421	0.84649	1	No	NA
ENSG00000177595	PIDD	11	799179	806095	ENSG00000165621	OXGR1	13	97637973	97646984	0.0066088	10.72	5.09E-07	0.0458339	NA	NA	NA	NA	No	NA
ENSG00000129282	MRM1	17	34958001	34965407	ENSG00000113140	SPARC	5	151040657	151066726	0.0081909	6.6495	5.10E-07	0.04590105	0.015049	2.33	0.030723	1	No	NA
ENSG00000109667	SLC2A9	4	9772777	10056560	ENSG00000110448	CD5	11	60869867	60895324	0.0081904	6.6491	5.11E-07	0.04592857	0.0026882	0.41105	0.87199	1	No	NA
ENSG00000132382	MYBBP1A	17	4443643	4458926	ENSG00000091972	CD200	3	112051688	112081659	0.0076955	7.4946	5.12E-07	0.04601629	0.016175	1.8763	0.060384	1	No	NA
ENSG00000108395	TRIM37	17	57059999	57184282	ENSG00000118495	PLAGL1	6	144261437	144385735	0.006606	10.715	5.13E-07	0.0460617	0.0041497	0.76339	0.57631	1	No	NA
ENSG00000257230	RP11-272B17.2	12	59917059	59917965	ENSG00000164053	ATRIP	3	48488114	48506061	0.0052005	25.281	5.13E-07	0.04610798	0.0031604	2.9168	0.087998	1	No	NA
ENSG00000004059	ARF5	7	127228399	127231142	ENSG00000111801	BTN3A3	6	26440700	26453643	0.0081876	6.6468	5.14E-07	0.04610798	0.0056326	0.86384	0.52098	1	No	NA
ENSG00000004059	ARF5	7	127228399	127231142	ENSG00000026950	BTN3A1	6	26402465	26415444	0.0081876	6.6468	5.14E-07	0.04610798	0.0037472	0.5736	0.75158	1	No	NA
ENSG00000110851	PRDM4	12	108126643	108155049	ENSG00000162676	GFI1	1	92940319	92952433	0.0081864	6.6458	5.15E-07	0.04620122	0.0093839	1.4446	0.19451	1	No	NA
ENSG00000214216	IQCJ	3	158680717	158984096	ENSG00000182196	ARL6IP4	12	123466504	123467456	0.005199	25.274	5.15E-07	0.04620122	0.00077137	0.71021	0.39959	1	No	NA
ENSG00000163823	CCR1	3	46243200	46249887	ENSG00000144290	SLC4A10	2	162480845	162841792	0.0081859	6.6454	5.16E-07	0.04621776	0.013532	2.0919	0.051824	1	Yes	Yes
ENSG00000136147	PHF11	13	50069801	50103123	ENSG00000169507	SLC38A11	2	165752696	165812035	0.010393	4.6075	5.16E-07	0.04624325	0.018386	1.7063	0.074825	1	No	NA
ENSG00000172716	SLFN11	17	33677324	33700720	ENSG00000196961	AP2A1	19	50270225	50309510	0.010798	4.3889	5.19E-07	0.04645673	0.011792	0.9039	0.54253	1	Yes	No
ENSG00000173744	AGFG1	2	228336868	228421384	ENSG00000255837	TAS2R20	12	11149094	11150474	0.0081826	6.6427	5.20E-07	0.0464973	0.0064546	1.4893	0.20333	1	No	NA
ENSG00000114378	HYAL1	3	50337320	50349812	ENSG00000175691	ZNF77	19	2933216	2944969	0.0071654	8.7201	5.20E-07	0.04649937	0.010696	1.6487	0.13064	1	No	NA
ENSG00000006125	AP2B1	17	33913918	34053436	ENSG00000204613	TRIM10	6	30119722	30128711	0.0086527	6.0224	5.21E-07	0.0465607	0.0073509	1.1293	0.34307	1	Yes	Yes
ENSG00000159713	TPPP3	16	67423712	67427438	ENSG00000135047	CTSL	9	90340434	90346308	0.0071645	8.7189	5.21E-07	0.0465607	NA	NA	NA	NA	No	NA
ENSG00000139330	KERA	12	91444268	91451760	ENSG00000116406	EDEM3	1	184659365	184724047	0.0059665	14.511	5.21E-07	0.04656185	0.0012036	0.5537	0.57501	1	No	NA
ENSG00000174840	PDE12	3	57542003	57552571	ENSG00000172572	PDE3A	12	20522179	20837315	0.007164	8.7184	5.22E-07	0.04657013	NA	NA	NA	NA	Yes	No
ENSG00000135409	AMHR2	12	53817639	53825318	ENSG00000172728	FUT10	8	33228342	33319245	0.0076864	7.4856	5.23E-07	0.04664357	0.0045866	0.84413	0.5185	1	No	NA
ENSG00000137831	UACA	15	70946893	71055932	ENSG00000105497	ZNF175	19	52074551	52092991	0.00865	6.0206	5.24E-07	0.0467323	0.0093854	1.2371	0.27936	1	No	NA
ENSG00000112276	BVES	6	105544697	105585049	ENSG00000151466	SCLT1	4	129786076	130003528	0.0071619	8.7158	5.24E-07	0.0467323	0.0040278	0.74087	0.59293	1	No	NA
ENSG00000177096	FAM109B	22	42470255	42475445	ENSG00000147883	CDKN2B	9	22002902	22009362	0.008649	6.0199	5.25E-07	0.04680557	0.011162	1.4739	0.17286	1	No	NA
ENSG00000154768	C17orf50	17	34087916	34092098	ENSG00000160445	ZER1	9	131492065	131534693	0.0081756	6.6369	5.28E-07	0.04700549	0.010356	1.3663	0.21615	1	Yes	No
ENSG00000139330	KERA	12	91444268	91451760	ENSG00000138698	RAP1GDS1	4	99182535	99365012	0.0059613	14.498	5.28E-07	0.04700549	0.0032311	1.4895	0.22603	1	No	NA
ENSG00000258429	PDF	16	69363900	69364498	ENSG00000162704	ARPC5	1	183592401	183604892	0.0071584	8.7116	5.28E-07	0.04700549	0.0054181	1.2489	0.2886	1	No	NA
ENSG00000165084	C8orf34	8	69350148	69731257	ENSG00000237541	HLA-DQA2	6	32709119	32714992	0.0086464	6.0181	5.28E-07	0.04700549	0.0060143	0.79004	0.59572	1	No	NA
ENSG00000141741	MIEN1	17	37885409	37886014	ENSG00000112053	SLC26A8	6	35911291	35992645	0.0076806	7.48	5.29E-07	0.04707228	0.0094217	1.7425	0.12229	1	No	NA
ENSG00000243317	C7orf73	7	135347244	135373776	ENSG00000185305	ARL15	5	53179775	53606412	0.0071574	8.7103	5.29E-07	0.04707228	0.0026854	0.82394	0.48077	1	No	NA
ENSG00000112592	TBP	6	170863390	170881958	ENSG00000103642	LACTB	15	63413999	63434260	0.0076802	7.4796	5.30E-07	0.04709098	0.0040047	0.92177	0.4505	1	No	NA
ENSG00000099849	RASSF7	11	560404	564021	ENSG00000108576	SLC6A4	17	28521337	28563020	0.0086444	6.0166	5.30E-07	0.04710167	0.020396	2.7186	0.008552	1	Yes	No
ENSG00000064042	LIMCH1	4	41361624	41702061	ENSG00000038945	MSR1	8	15965387	16424999	0.0086443	6.0166	5.30E-07	0.04710167	0.011401	1.5058	0.16153	1	No	NA
ENSG00000172660	TAF15	17	34136459	34174246	ENSG00000067836	ROGDI	16	4846969	4852951	0.0081717	6.6338	5.32E-07	0.04722946	0.0166	2.5742	0.017705	1	No	NA
ENSG00000184162	NR2C2AP	19	19312867	19314233	ENSG00000183718	TRIM52	5	180681417	180688119	0.00659	10.689	5.32E-07	0.04722946	0.0081192	2.5048	0.05787	1	No	NA
ENSG00000172123	SLFN12	17	33738079	33760302	ENSG00000103257	SLC7A5	16	87863629	87903094	0.011961	3.8916	5.33E-07	0.04726661	0.013208	2.0412	0.057804	1	Yes	No
ENSG00000142065	ZFP14	19	36827162	36870101	ENSG00000111291	GPRC5D	12	13093709	13105081	0.0081708	6.6331	5.33E-07	0.04726661	0.0077769	1.4359	0.20878	1	No	NA
ENSG00000139330	KERA	12	91444268	91451760	ENSG00000086619	ERO1LB	1	236378855	236445319	0.0059569	14.487	5.34E-07	0.04727276	0.0021768	1.0024	0.36739	1	No	NA
ENSG00000183207	RUVBL2	19	49496705	49518943	ENSG00000126353	CCR7	17	38710021	38721724	0.011573	4.0337	5.35E-07	0.04739404	0.0046445	0.53253	0.83253	1	No	NA
ENSG00000152092	ASTN1	1	176826438	177134109	ENSG00000155542	SETD9	5	56205087	56221359	0.0081674	6.6303	5.37E-07	0.0475639	NA	NA	NA	NA	No	NA
ENSG00000144224	UBXN4	2	136499189	136542625	ENSG00000146072	TNFRSF21	6	47199268	47277641	0.0081658	6.629	5.39E-07	0.04770796	0.027961	4.3868	0.00022209	0.38710287	No	NA
ENSG00000139330	KERA	12	91444268	91451760	ENSG00000145349	CAMK2D	4	114372188	114683083	0.0059519	14.475	5.40E-07	0.04774902	0.0014787	0.68048	0.50663	1	No	NA
ENSG00000165219	GAPVD1	9	128024073	128129486	ENSG00000149577	SIDT2	11	117049449	117068160	0.0076718	7.4713	5.40E-07	0.04774902	0.0015898	0.36504	0.8336	1	No	NA
ENSG00000214511	HIGD1C	12	51347705	51364289	ENSG00000156689	GLYATL2	11	58601542	58671688	0.0065837	10.679	5.40E-07	0.04775759	NA	NA	NA	NA	No	NA
ENSG00000159763	PIP	7	142829170	142836839	ENSG00000110002	VWA5A	11	123986069	124018428	0.0059504	14.471	5.42E-07	0.04787128	0.0014234	0.65497	0.5197	1	No	NA
ENSG00000139330	KERA	12	91444268	91451760	ENSG00000122566	HNRNPA2B1	7	26229547	26240366	0.0059485	14.466	5.45E-07	0.04805873	0.0012649	0.58197	0.559	1	No	NA
ENSG00000103876	FAH	15	80444832	80479288	ENSG00000243725	TTC4	1	55181495	55181816	0.011172	4.1925	5.45E-07	0.04805873	0.0080931	0.56988	0.87906	1	No	NA
ENSG00000139330	KERA	12	91444268	91451760	ENSG00000006468	ETV1	7	13930853	14031050	0.0059485	14.466	5.45E-07	0.04805873	NA	NA	NA	NA	No	NA
ENSG00000103876	FAH	15	80444832	80479288	ENSG00000271723	MROH7	1	55124788	55181283	0.011172	4.1925	5.45E-07	0.04805873	NA	NA	NA	NA	No	NA
ENSG00000139437	TCHP	12	110338079	110355874	ENSG00000109606	DHX15	4	24519064	24586173	0.0086312	6.0074	5.46E-07	0.04809977	0.0034391	0.52627	0.78863	1	No	NA
ENSG00000164258	NDUFS4	5	52856463	52979168	ENSG00000096006	CRISP3	6	49695097	49712150	0.0095225	5.1574	5.46E-07	0.04812931	0.012753	1.309	0.22774	1	No	NA
ENSG00000204978	C19orf69	19	41949063	41950670	ENSG00000144820	GPR128	3	100328433	100414323	0.0071433	8.693	5.47E-07	0.04817204	NA	NA	NA	NA	No	NA
ENSG00000117385	LEPRE1	1	43212006	43232755	ENSG00000135407	AVIL	12	58193578	58212487	0.0081568	6.6216	5.50E-07	0.04841193	0.0028717	0.52761	0.7555	1	No	NA
ENSG00000009954	BAZ1B	7	72854728	72936608	ENSG00000163751	CPA3	3	148583043	148614983	0.0076625	7.4622	5.51E-07	0.04851522	0.0065098	1.2004	0.30697	1	Yes	No
ENSG00000162076	FLYWCH2	16	2933187	2949383	ENSG00000196284	SUPT3H	6	44777054	45345690	0.0090788	5.5304	5.52E-07	0.04856476	0.0058348	1.0752	0.37251	1	No	NA
ENSG00000139437	TCHP	12	110338079	110355874	ENSG00000167283	ATP5L	11	118274639	118302211	0.0086251	6.0031	5.53E-07	0.04861603	0.0041738	0.63918	0.69894	1	No	NA
ENSG00000167077	MEI1	22	42095503	42195460	ENSG00000197747	S100A10	1	151955391	151966866	0.0059408	14.448	5.55E-07	0.0487604	0.00022553	0.069029	0.97642	1	No	NA
ENSG00000179979	CRIPAK	4	1385340	1389780	ENSG00000266967	AARSD1	17	41114192	41116515	0.0071366	8.6848	5.55E-07	0.04877306	0.0011786	0.27051	0.89702	1	No	NA
ENSG00000179979	CRIPAK	4	1385340	1389780	ENSG00000108825	PTGES3L-AAI	17	41131922	41132020	0.0071366	8.6848	5.55E-07	0.04877306	NA	NA	NA	NA	No	NA
ENSG00000189164	ZNF527	19	37861963	37883968	ENSG00000116962	NID1	1	236139130	236228462	0.0086222	6.001	5.56E-07	0.04883643	0.021681	2.8937	0.0053798	1	No	NA
ENSG00000139330	KERA	12	91444268	91451760	ENSG00000127995	CASD1	7	94138531	94186331	0.0059394	14.444	5.57E-07	0.04884272	0.0011805	0.54307	0.58115	1	No	NA
ENSG00000198604	BAZ1A	14	35221937	35344853	ENSG00000157017	GHRL	3	10328388	10334631	0.008151	6.6169	5.57E-07	0.04884725	0.038273	6.0689	3.03E-06	0.0055146	No	NA
ENSG00000130584	ZBTB46	20	62375019	62462597	ENSG00000112299	VNN1	6	133002729	133035188	0.0081507	6.6166	5.57E-07	0.04885616	0.0050928	0.93778	0.45557	1	No	NA
ENSG00000073605	GSDMB	17	38060848	38074903	ENSG00000128512	DOCK4	7	111366166	111846466	0.0071332	8.6807	5.60E-07	0.04904469	0.0041198	0.94837	0.4352	1	No	NA
ENSG00000184313	MROH7	1	55129432	55175939	ENSG00000038945	MSR1	8	15965387	16424999	0.0081482	6.6145	5.61E-07	0.04909204	0.0067576	1.2464	0.28529	1	No	NA
ENSG00000139330	KERA	12	91444268	91451760	ENSG00000077147	TM9SF3	10	98277866	98347209	0.0059356	14.435	5.62E-07	0.04918488	0.0011036	0.50766	0.60207	1	No	NA

ENSG00000108733	PEX12	17	33901814	33905882	ENSG00000125257	ABCC4	13	95672083	95953687	0.0076534	7.4533	5.63E-07	0.04924434	0.0027843	0.5115	0.76772	1	Yes	No
ENSG00000183508	FAM46C	1	118148556	118170994	ENSG00000161911	TREML1	6	41117080	41122075	0.0090696	5.5247	5.63E-07	0.04924434	0.040652	4.836	7.48E-06	0.0135014	No	NA
ENSG00000204403	CASP12	11	104756445	104769397	ENSG00000171224	C10orf35	10	71390007	71393352	0.0095078	5.1494	5.63E-07	0.04924434	0.015134	1.7537	0.082585	1	No	NA
ENSG00000175573	C11orf68	11	65684279	65686588	ENSG00000180509	KCNE1	21	35818988	35884573	0.0065648	10.648	5.65E-07	0.04935611	0.00072775	0.22285	0.88054	1	No	NA
ENSG00000175573	C11orf68	11	65684279	65686588	ENSG00000243627	AP000322.53	21	35791013	35796264	0.0065648	10.648	5.65E-07	0.04935611	NA	NA	NA	NA	No	NA
ENSG00000167491	GATAD2A	19	19496639	19619740	ENSG00000108309	RUNDC3A	17	42385927	42396039	0.0065635	10.646	5.67E-07	0.04948615	0.012786	3.9631	0.008028	1	No	NA
ENSG00000197863	ZNF790	19	37308330	37341689	ENSG00000116962	NID1	1	236139130	236228462	0.0086126	5.9943	5.68E-07	0.04958378	0.017244	2.2911	0.025653	1	No	NA
ENSG00000243646	IL10RB	21	34638663	34669539	ENSG00000165949	IFI27	14	94571182	94583033	0.011542	4.0226	5.69E-07	0.04962985	0.0603	6.5025	5.19E-09	9.69E-06	Yes	Yes
ENSG00000188921	PTPLAD2	9	21003620	21031635	ENSG00000105948	TTC26	7	138818490	138876732	0.0090639	5.5212	5.70E-07	0.04969594	0.013346	1.5437	0.13802	1	No	NA
ENSG00000213171	LINGO4	1	151772740	151775193	ENSG00000161911	TREML1	6	41117080	41122075	0.0081394	6.6074	5.71E-07	0.04980966	0.011603	1.7902	0.098043	1	No	NA
ENSG00000116194	ANGPTL1	1	178818840	178840187	ENSG00000129245	FXR2	17	7494548	7518189	0.0090624	5.5203	5.72E-07	0.04980966	0.0081743	0.94058	0.48187	1	No	NA
ENSG00000141748	ARL5C	17	37313147	37323318	ENSG00000198216	CACNA1E	1	181382238	181777219	0.0059283	14.417	5.72E-07	0.04981288	0.0050451	2.33	0.097871	1	No	NA
ENSG00000108733	PEX12	17	33901814	33905882	ENSG00000185630	PBX1	1	164524821	164868533	0.0076454	7.4454	5.73E-07	0.04986543	0.011052	2.0474	0.069844	1	No	NA
ENSG00000139330	KERA	12	91444268	91451760	ENSG00000163655	GMPS	3	155588325	155658457	0.0059277	14.416	5.73E-07	0.04986543	0.001135	0.52211	0.59344	1	No	NA
ENSG00000142102	ATHL1	11	289138	296107	ENSG00000138798	EGF	4	110834040	110933422	0.013411	3.447	5.74E-07	0.04989385	0.00015683	0.072074	0.93047	1	Yes	Yes
