# Supplementary material for: Imputed gene associations identify replicable trans‐acting genes enriched in transcription pathways and complex traits
Source: Genet Epidemiol. 2019 Apr 4;43(6):596–608. doi: 10.1002/gepi.22205 (PMC6687523; doi:10.1002/gepi.22205)
Supplement: Supplementary file 8 — Supplementary Information [file GEPI-43-596-s008.pdf]

predgene	predname	predChr	predS1	predS2	obsgene	obsname	obsChr	obsS1	obsS2	FHS_stat	FHS_beta	FHS_pval	FHS_FDR	DGN_stat	DGN_beta	DGN_pval	rsid	eQTLGen_trans_P	eQTLGen_trans_FDR	eQTLGen_cis_P	eQTLGen_cis_FDR	GTEx_tissue
ENSG00000238243	OR2W3	1	248058859	248060449	ENSG00000166086	JAM3	11	133938820	134018713	0.044029	18.519	7.89E-40	5.22E-33	0.062774	5.0736	3.09E-08	rs3811445	8.19E-246	0	5.14E-105	0	Adipose_Subcutaneous
ENSG00000238243	OR2W3	1	248058859	248060449	ENSG00000166086	JAM3	11	133938820	134018713	0.044029	18.519	7.89E-40	5.22E-33	0.062774	5.0736	3.09E-08	rs3811445	8.19E-246	0	5.14E-105	0	Colon_Sigmoid
ENSG00000238243	OR2W3	1	248058859	248060449	ENSG00000166086	JAM3	11	133938820	134018713	0.044029	18.519	7.89E-40	5.22E-33	0.062774	5.0736	3.09E-08	rs3811445	8.19E-246	0	5.14E-105	0	Colon_Transverse
ENSG00000238243	OR2W3	1	248058859	248060449	ENSG00000166086	JAM3	11	133938820	134018713	0.044029	18.519	7.89E-40	5.22E-33	0.062774	5.0736	3.09E-08	rs3811444	3.27e-310	0	9.28E-96	0	Artery_Tibial
ENSG00000238243	OR2W3	1	248058859	248060449	ENSG00000166086	JAM3	11	133938820	134018713	0.044029	18.519	7.89E-40	5.22E-33	0.062774	5.0736	3.09E-08	rs3811444	3.27e-310	0	9.28E-96	0	Whole_Blood
ENSG00000108733	PEX12	17	33901814	33905882	ENSG00000158856	DMTN	8	21906506	21940038	0.034814	34.857	3.91E-35	1.86E-28	NA	NA	NA	rs9915021	7.02E-65	0	NA	NA	Brain_Caudate_basal_ganglia
ENSG00000108733	PEX12	17	33901814	33905882	ENSG00000158856	DMTN	8	21906506	21940038	0.034814	34.857	3.91E-35	1.86E-28	NA	NA	NA	rs9915021	7.02E-65	0	NA	NA	Breast_Mammary_Tissue
ENSG00000108733	PEX12	17	33901814	33905882	ENSG00000158856	DMTN	8	21906506	21940038	0.034814	34.857	3.91E-35	1.86E-28	NA	NA	NA	rs10512472	1.91E-98	0	1.06E-12	0	Brain_Caudate_basal_ganglia
ENSG00000006125	AP2B1	17	33913918	34053436	ENSG00000158856	DMTN	8	21906506	21940038	0.035769	25.596	1.35E-34	6.00E-28	NA	NA	NA	rs10512472	1.91E-98	0	3.62E-14	0	Heart_Left_Ventricle
ENSG00000205045	SLFN12L	17	33800708	33864880	ENSG00000158856	DMTN	8	21906506	21940038	0.03533	25.27	3.93E-34	1.64E-27	NA	NA	NA	rs8082605	2.91E-102	0	2.49E-31	0	Adipose_Subcutaneous
ENSG00000205045	SLFN12L	17	33800708	33864880	ENSG00000158856	DMTN	8	21906506	21940038	0.03533	25.27	3.93E-34	1.64E-27	NA	NA	NA	rs8082605	2.91E-102	0	2.49E-31	0	Adipose_Visceral_Omentum
ENSG00000205045	SLFN12L	17	33800708	33864880	ENSG00000158856	DMTN	8	21906506	21940038	0.03533	25.27	3.93E-34	1.64E-27	NA	NA	NA	rs8082605	2.91E-102	0	2.49E-31	0	Heart_Left_Ventricle
ENSG00000205045	SLFN12L	17	33800708	33864880	ENSG00000158856	DMTN	8	21906506	21940038	0.03533	25.27	3.93E-34	1.64E-27	NA	NA	NA	rs8082605	2.91E-102	0	2.49E-31	0	Nerve_Tibial
ENSG00000205045	SLFN12L	17	33800708	33864880	ENSG00000158856	DMTN	8	21906506	21940038	0.03533	25.27	3.93E-34	1.64E-27	NA	NA	NA	rs10512472	1.91E-98	0	4.96E-30	0	Pancreas
ENSG00000108733	PEX12	17	33901814	33905882	ENSG00000022840	RNF10	12	120971283	121015397	0.028189	28.032	4.30E-28	1.21E-21	0.010127	1.8742	0.096301	rs9915021	1.07E-57	0	NA	NA	Brain_Caudate_basal_ganglia
ENSG00000108733	PEX12	17	33901814	33905882	ENSG00000022840	RNF10	12	120971283	121015397	0.028189	28.032	4.30E-28	1.21E-21	0.010127	1.8742	0.096301	rs9915021	1.07E-57	0	NA	NA	Breast_Mammary_Tissue
ENSG00000108733	PEX12	17	33901814	33905882	ENSG00000022840	RNF10	12	120971283	121015397	0.028189	28.032	4.30E-28	1.21E-21	0.010127	1.8742	0.096301	rs10512472	2.97E-71	0	1.06E-12	0	Brain_Caudate_basal_ganglia
ENSG00000172660	TAF15	17	34136459	34174246	ENSG00000158856	DMTN	8	21906506	21940038	0.028606	23.711	8.80E-28	2.40E-21	NA	NA	NA	rs9915021	7.02E-65	0	NA	NA	Skin_Not_Sun_Exposed_Suprapubic
ENSG00000006125	AP2B1	17	33913918	34053436	ENSG00000022840	RNF10	12	120971283	121015397	0.027739	19.686	3.59E-26	8.76E-20	0.020938	3.2613	0.0035335	rs10512472	2.97E-71	0	3.62E-14	0	Heart_Left_Ventricle
ENSG00000141076	CIRH1A	16	69165194	69265033	ENSG00000255833	TIFAB	5	134787899	134788089	0.026103	25.902	6.82E-26	1.63E-19	0.0022297	0.4094	0.84246	rs929843	1.75E-20	0	NA	NA	Lung
ENSG00000154760	SLFN13	17	33762115	33775856	ENSG00000158856	DMTN	8	21906506	21940038	0.029526	13.348	1.74E-25	3.96E-19	NA	NA	NA	rs225245	4.74E-222	0	NA	NA	Liver
ENSG00000154760	SLFN13	17	33762115	33775856	ENSG00000158856	DMTN	8	21906506	21940038	0.029526	13.348	1.74E-25	3.96E-19	NA	NA	NA	rs8082605	2.91E-102	0	2.08E-19	0	Artery_Tibial
ENSG00000154760	SLFN13	17	33762115	33775856	ENSG00000158856	DMTN	8	21906506	21940038	0.029526	13.348	1.74E-25	3.96E-19	NA	NA	NA	rs8082605	2.91E-102	0	2.08E-19	0	Esophagus_Mucosa
ENSG00000154760	SLFN13	17	33762115	33775856	ENSG00000158856	DMTN	8	21906506	21940038	0.029526	13.348	1.74E-25	3.96E-19	NA	NA	NA	rs8082605	2.91E-102	0	2.08E-19	0	Nerve_Tibial
ENSG00000154760	SLFN13	17	33762115	33775856	ENSG00000158856	DMTN	8	21906506	21940038	0.029526	13.348	1.74E-25	3.96E-19	NA	NA	NA	rs8082605	2.91E-102	0	2.08E-19	0	Pituitary
ENSG00000154760	SLFN13	17	33762115	33775856	ENSG00000158856	DMTN	8	21906506	21940038	0.029526	13.348	1.74E-25	3.96E-19	NA	NA	NA	rs10512472	1.91E-98	0	6.34E-38	0	Uterus
ENSG00000154760	SLFN13	17	33762115	33775856	ENSG00000070182	SPTB	14	65216372	65346601	0.0284	12.824	2.40E-24	4.87E-18	0.01803	1.3908	0.16408	rs225245	8.72E-09	0.00014844	NA	NA	Liver
ENSG00000141150	RASL10B	17	34058668	34070540	ENSG00000158856	DMTN	8	21906506	21940038	0.025836	16.009	1.51E-23	2.90E-17	NA	NA	NA	rs9915021	7.02E-65	0	NA	NA	Cells_Transformed_fibroblasts
ENSG00000006125	AP2B1	17	33913918	34053436	ENSG00000113140	SPARC	5	151040657	151066726	0.024269	17.162	1.41E-22	2.61E-16	0.038528	6.111	2.72E-06	rs10512472	7.03E-109	0	3.62E-14	0	Heart_Left_Ventricle
ENSG00000006125	AP2B1	17	33913918	34053436	ENSG00000120885	CLU	8	27454434	27472548	0.022828	16.119	4.29E-21	7.03E-15	0.030828	4.8509	6.89E-05	rs10512472	1.27E-27	0	3.62E-14	0	Heart_Left_Ventricle
ENSG00000172123	SLFN12	17	33738079	33760302	ENSG00000158856	DMTN	8	21906506	21940038	0.026889	8.8828	1.03E-20	1.66E-14	NA	NA	NA	rs225245	4.74E-222	0	NA	NA	Cells_EBV-transformed_lymphocytes
ENSG00000172123	SLFN12	17	33738079	33760302	ENSG00000158856	DMTN	8	21906506	21940038	0.026889	8.8828	1.03E-20	1.66E-14	NA	NA	NA	rs9915021	7.02E-65	0	NA	NA	Breast_Mammary_Tissue
ENSG00000172660	TAF15	17	34136459	34174246	ENSG00000022840	RNF10	12	120971283	121015397	0.021679	17.842	1.45E-20	2.29E-14	0.015663	2.4266	0.024741	rs9915021	1.07E-57	0	NA	NA	Skin_Not_Sun_Exposed_Suprapubic
ENSG00000108733	PEX12	17	33901814	33905882	ENSG00000100614	PPM1A	14	60712470	60765805	0.020937	20.667	1.75E-20	2.66E-14	0.0095301	1.7627	0.11792	rs9915021	6.10E-09	0.00013559	NA	NA	Brain_Caudate_basal_ganglia
ENSG00000108733	PEX12	17	33901814	33905882	ENSG00000100614	PPM1A	14	60712470	60765805	0.020937	20.667	1.75E-20	2.66E-14	0.0095301	1.7627	0.11792	rs9915021	6.10E-09	0.00013559	NA	NA	Breast_Mammary_Tissue
ENSG00000108733	PEX12	17	33901814	33905882	ENSG00000100614	PPM1A	14	60712470	60765805	0.020937	20.667	1.75E-20	2.66E-14	0.0095301	1.7627	0.11792	rs10512472	4.22E-11	0	1.06E-12	0	Brain_Caudate_basal_ganglia
ENSG00000205045	SLFN12L	17	33800708	33864880	ENSG00000022840	RNF10	12	120971283	121015397	0.022218	15.679	1.81E-20	2.73E-14	0.016902	2.2449	0.028792	rs8082605	1.03E-82	0	2.49E-31	0	Adipose_Subcutaneous
ENSG00000205045	SLFN12L	17	33800708	33864880	ENSG00000022840	RNF10	12	120971283	121015397	0.022218	15.679	1.81E-20	2.73E-14	0.016902	2.2449	0.028792	rs8082605	1.03E-82	0	2.49E-31	0	Adipose_Visceral_Omentum
ENSG00000205045	SLFN12L	17	33800708	33864880	ENSG00000022840	RNF10	12	120971283	121015397	0.022218	15.679	1.81E-20	2.73E-14	0.016902	2.2449	0.028792	rs8082605	1.03E-82	0	2.49E-31	0	Heart_Left_Ventricle
ENSG00000205045	SLFN12L	17	33800708	33864880	ENSG00000022840	RNF10	12	120971283	121015397	0.022218	15.679	1.81E-20	2.73E-14	0.016902	2.2449	0.028792	rs8082605	1.03E-82	0	2.49E-31	0	Nerve_Tibial
ENSG00000205045	SLFN12L	17	33800708	33864880	ENSG00000022840	RNF10	12	120971283	121015397	0.022218	15.679	1.81E-20	2.73E-14	0.016902	2.2449	0.028792	rs10512472	2.97E-71	0	4.96E-30	0	Pancreas
ENSG00000102908	NFAT5	16	69598997	69738569	ENSG00000255833	TIFAB	5	134787899	134788089	0.021347	17.563	3.19E-20	4.70E-14	0.0059486	0.91259	0.48495	rs929843	1.75E-20	0	NA	NA	Esophagus_Muscularis
ENSG00000102908	NFAT5	16	69598997	69738569	ENSG00000255833	TIFAB	5	134787899	134788089	0.021347	17.563	3.19E-20	4.70E-14	0.0059486	0.91259	0.48495	rs1364063	1.89E-12	0	5.08E-08	0.000202365	Pancreas
ENSG00000006125	AP2B1	17	33913918	34053436	ENSG00000103148	NPRL3	16	138697	188859	0.02194	15.478	3.49E-20	5.11E-14	0.023502	3.6704	0.0013113	rs10512472	6.78E-42	0	3.62E-14	0	Heart_Left_Ventricle
ENSG00000108733	PEX12	17	33901814	33905882	ENSG00000166947	EPB42	15	43398423	43513481	0.020501	20.227	4.98E-20	7.20E-14	0.0076745	1.4168	0.21559	rs9915021	3.48E-18	0	NA	NA	Brain_Caudate_basal_ganglia
ENSG00000108733	PEX12	17	33901814	33905882	ENSG00000166947	EPB42	15	43398423	43513481	0.020501	20.227	4.98E-20	7.20E-14	0.0076745	1.4168	0.21559	rs9915021	3.48E-18	0	NA	NA	Breast_Mammary_Tissue
ENSG00000108733	PEX12	17	33901814	33905882	ENSG00000166947	EPB42	15	43398423	43513481	0.020501	20.227	4.98E-20	7.20E-14	0.0076745	1.4168	0.21559	rs10512472	2.95E-21	0	1.06E-12	0	Brain_Caudate_basal_ganglia
ENSG00000108733	PEX12	17	33901814	33905882	ENSG00000029534	ANK1	8	41510739	41754280	0.020054	19.777	1.45E-19	1.98E-13	0.0039326	0.7233	0.60602	rs9915021	6.17E-12	0	NA	NA	Brain_Caudate_basal_ganglia
ENSG00000108733	PEX12	17	33901814	33905882	ENSG00000029534	ANK1	8	41510739	41754280	0.020054	19.777	1.45E-19	1.98E-13	0.0039326	0.7233	0.60602	rs9915021	6.17E-12	0	NA	NA	Breast_Mammary_Tissue
ENSG00000108733	PEX12	17	33901814	33905882	ENSG00000029534	ANK1	8	41510739	41754280	0.020054	19.777	1.45E-19	1.98E-13	0.0039326	0.7233	0.60602	rs10512472	4.67E-16	0	1.06E-12	0	Brain_Caudate_basal_ganglia
ENSG00000168374	ARF4	3	57557090	57583947	ENSG00000259207	ITGB3	17	45387505	45389182	0.019884	19.606	2.18E-19	2.88E-13	0.047046	9.0444	2.10E-08	rs17825630	4.73E-14	0	NA	NA	Artery_Tibial
ENSG00000168374	ARF4	3	57557090	57583947	ENSG00000259207	ITGB3	17	45387505	45389182	0.019884	19.606	2.18E-19	2.88E-13	0.047046	9.0444	2.10E-08	rs17825630	4.73E-14	0	NA	NA	Heart_Atrial_Appendage
ENSG00000108733	PEX12	17	33901814	33905882	ENSG00000167671	UBXN6	19	4446046	4457819	0.019839	19.56	2.43E-19	3.19E-13	0.025976	4.8858	0.00020333	rs9915021	2.43E-46	0	NA	NA	Brain_Caudate_basal_ganglia
ENSG00000108733	PEX12	17	33901814	33905882	ENSG00000167671	UBXN6	19	4446046	4457819	0.019839	19.56	2.43E-19	3.19E-13	0.025976	4.8858	0.00020333	rs9915021	2.43E-46	0	NA	NA	Breast_Mammary_Tissue
ENSG00000108733	PEX12	17	33901814	33905882	ENSG00000167671	UBXN6	19	4446046	4457819	0.019839	19.56	2.43E-19	3.19E-13	0.025976	4.8858	0.00020333	rs10512472	4.51E-73	0	1.06E-12	0	Brain_Caudate_basal_ganglia
ENSG00000163946	FAM208A	3	56658507	56717265	ENSG00000259207	ITGB3	17	45387505	45389182	0.021364	13.178	5.49E-19	6.99E-13	0.04809	6.5964	1.27E-07	rs12485738	2.40E-225	0	NA	NA	Esophagus_Mucosa
ENSG00000163946	FAM208A	3	56658507	56717265	ENSG00000259207	ITGB3	17	45387505	45389182	0.021364	13.178	5.49E-19	6.99E-13	0.04809	6.5964	1.27E-07	rs1354034	3.27170000000001e-	0	NA	NA	Heart_Atrial_Appendage
ENSG00000172716	SLFN11	17	33677324	33700720	ENSG00000158856	DMTN	8	21906506	21940038	0.023488	9.6711	6.70E-19	8.37E-13	NA	NA	NA	rs225245	4.74E-222	0	5.77E-07	0.001884469	Cells_Transformed_fibroblasts
ENSG00000172716	SLFN11	17	33677324	33700720	ENSG00000158856	DMTN	8	21906506	21940038	0.023488	9.6711	6.70E-19	8.37E-13	NA	NA	NA	rs8082605	2.91E-102	0	NA	NA	Adrenal_Gland
ENSG00000154760	SLFN13	17	33762115	33775856	ENSG00000022840	RNF10	12	120971283	121015397	0.022926	10.294	7.18E-19	8.92E-13	0.022092	1.7113	0.059583	rs225245	1.95E-178	0	NA	NA	Liver
ENSG00000154760	SLFN13	17	33762115	33775856	ENSG00000022840	RNF10	12	120971283	121015397	0.022926	10.294	7.18E-19	8.92E-13	0.022092	1.7113	0.059583	rs8082605	1.03E-82	0	2.08E-19	0	Artery_Tibial
ENSG00000154760	SLFN13	17	33762115	33775856	ENSG00000022840	RNF10	12	120971283	121015397	0.022926	10.294	7.18E-19	8.92E-13	0.022092	1.7113	0.059583	rs8082605	1.03E-82	0	2.08E-19	0	Esophagus_Mucosa
ENSG00000154760	SLFN13	17	33762115	33775856	ENSG00000022840	RNF10	12	120971283	121015397	0.022926	10.294	7.18E-19	8.92E-13	0.022092	1.7113	0.059583	rs8082605	1.03E-82	0	2.08E-19	0	Nerve_Tibial
ENSG00000154760	SLFN13	17	33762115	33775856	ENSG00000022840	RNF10	12	120971283	121015397	0.022926	10.294	7.18E-19	8.92E-13	0.022092	1.7113	0.059583	rs8082605	1.03E-82	0	2.08E-19	0	Pituitary
ENSG00000154760	SLFN13	17	33762115	33775856	ENSG00000022840	RNF10	12	120971283	121015397	0.022926	10.294	7.18E-19	8.92E-13	0.022092	1.7113	0.059583	rs10512472	2.97E-71	0	6.34E-38	0	Uterus
ENSG00000006125	AP2B1	17	33913918	34053436	ENSG00000167671	UBXN6	19	4446046	4457819	0.020372	14.349	1.39E-18	1.71E-12	0.028611	4.4916	0.0001707	rs10512472	4.51E-73	0	3.62E-14	0	Heart_Left_Ventricle
ENSG00000108733	PEX12	17	33901814	33905882	ENSG00000167992	VWCE	11	61025762	61062896	0.018709	18.426	3.60E-18	4.27E-12	0.024282	4.5592	0.00041072	rs9915021	1.45E-41	0	NA	NA	Brain_Caudate_basal_ganglia
ENSG00000108733	PEX12	17	33901814	33905882	ENSG00000167992	VWCE	11	61025762	61062896	0.018709	18.426	3.60E-18	4.27E-12	0.024282	4.5592	0.00041072	rs9915021	1.45E-41	0	NA	NA	Breast_Mammary_Tissue
ENSG00000108733	PEX12	17	33901814	33905882	ENSG00000167992	VWCE	11	61025762	61062896	0.018709	18.426	3.60E-18	4.27E-12	0.024282	4.5592	0.00041072	rs10512472	2.21E-55	0	1.06E-12	0	Brain_Caudate_basal_ganglia
ENSG00000108733	PEX12	17	33901814	33905882	ENSG00000115649	CNPPD1	2	220036619	220042035	0.018494	18.21	6.02E-18	6.93E-12	NA	NA	NA	rs9915021	3.39E-19	0	NA	NA	Brain_Caudate_basal_ganglia
ENSG00000108733	PEX12	17	33901814	33905882	ENSG00000115649	CNPPD1	2	220036619	220042035	0.018494	18.21	6.02E-18	6.93E-12	NA	NA	NA	rs9915021	3.39E-19	0	NA	NA	Breast_Mammary_Tissue
ENSG00000108733	PEX12	17	33901814	33905882	ENSG00000115649	CNPPD1	2	220036619	220042035	0.018494	18.21	6.02E-18	6.93E-12	NA	NA	NA	rs10512472	2.37E-29	0	1.06E-12	0	Brain_Caudate_basal_ganglia
ENSG00000006125	AP2B1	17	33913918	34053436	ENSG00000167992	VWCE	11	61025762	61062896	0.019692	13.861	6.84E-18	7.83E-12	0.036926	5.8471	5.38E-06	rs10512472	2.21E-55	0	3.62E-14	0	Heart_Left_Ventricle
ENSG00000108733	PEX12	17	33901814	33905882	ENSG00000103148	NPRL3	16	138697	188859	0.018355	18.069	8.39E-18	9.40E-12	0.017675	3.2963	0.0058837	rs9915021	1.32E-25	0	NA	NA	Brain_Caudate_basal_ganglia
ENSG00000108733	PEX12	17	33901814	33905882	ENSG00000103148	NPRL3	16	138697	188859	0.018355	18.069	8.39E-18	9.40E-12	0.017675	3.2963	0.0058837	rs9915021	1.32E-25	0	NA	NA	Breast_Mammary_Tissue
ENSG00000108733	PEX12	17	33901814	33905882	ENSG00000103148	NPRL3	16	138697	188859	0.018355	18.069	8.39E-18	9.40E-12	0.017675	3.2963	0.0058837	rs10512472	6.78E-42	0	1.06E-12	0	Brain_Caudate_basal_ganglia
ENSG00000172123	SLFN12	17	33738079	33760302	ENSG00000070182	SPTB	14	65216372	65346601	0.02382	7.8443	9.39E-18	1.04E-11	0.0073391	1.1275	0.34412	rs225245	8.72E-09	0.00014844	NA	NA	Cells_EBV-transformed_lymphocytes
ENSG00000108733	PEX12	17	33901814	33905882	ENSG00000144677	CTDSPL	3	37903451	38025960	0.018274	17.989	1.02E-17	1.12E-11	0.042292	8.0901	1.76E-07	rs9915021	1.38E-80	0	NA	NA	Brain_Caudate_basal_ganglia
ENSG00000108733	PEX12	17	33901814	33905882	ENSG00000144677	CTDSPL	3	37903451	38025960	0.018274	17.989	1.02E-17	1.12E-11	0.042292	8.0901	1.76E-07	rs9915021	1.38E-80	0	NA	NA	Breast_Mammary_Tissue

ENSG00000108733	PEX12	17	33901814	33905882	ENSG00000144677	CTDSPL	3	37903451	38025960	0.018274	17.989	1.02E-17	1.12E-11	0.042292	8.0901	1.76E-07	rs10512472	7.69E-165	0	1.06E-12	0	Brain_Caudate_basal_ganglia
ENSG00000205045	SLFN12L	17	33800708	33864880	ENSG00000115649	CNPPD1	2	220036619	220042035	0.019444	13.682	1.22E-17	1.33E-11	NA	NA	NA	rs8082605	6.08E-30	0	2.49E-31	0	Adipose_Subcutaneous
ENSG00000205045	SLFN12L	17	33800708	33864880	ENSG00000115649	CNPPD1	2	220036619	220042035	0.019444	13.682	1.22E-17	1.33E-11	NA	NA	NA	rs8082605	6.08E-30	0	2.49E-31	0	Adipose_Visceral_Omentum
ENSG00000205045	SLFN12L	17	33800708	33864880	ENSG00000115649	CNPPD1	2	220036619	220042035	0.019444	13.682	1.22E-17	1.33E-11	NA	NA	NA	rs8082605	6.08E-30	0	2.49E-31	0	Heart_Left_Ventricle
ENSG00000205045	SLFN12L	17	33800708	33864880	ENSG00000115649	CNPPD1	2	220036619	220042035	0.019444	13.682	1.22E-17	1.33E-11	NA	NA	NA	rs8082605	6.08E-30	0	2.49E-31	0	Nerve_Tibial
ENSG00000205045	SLFN12L	17	33800708	33864880	ENSG00000115649	CNPPD1	2	220036619	220042035	0.019444	13.682	1.22E-17	1.33E-11	NA	NA	NA	rs10512472	2.37E-29	0	4.96E-30	0	Pancreas
ENSG00000205045	SLFN12L	17	33800708	33864880	ENSG00000029534	ANK1	8	41510739	41754280	0.019255	13.547	1.90E-17	2.03E-11	0.0036513	0.4785	0.85059	rs8082605	4.69E-14	0	2.49E-31	0	Adipose_Subcutaneous
ENSG00000205045	SLFN12L	17	33800708	33864880	ENSG00000029534	ANK1	8	41510739	41754280	0.019255	13.547	1.90E-17	2.03E-11	0.0036513	0.4785	0.85059	rs8082605	4.69E-14	0	2.49E-31	0	Adipose_Visceral_Omentum
ENSG00000205045	SLFN12L	17	33800708	33864880	ENSG00000029534	ANK1	8	41510739	41754280	0.019255	13.547	1.90E-17	2.03E-11	0.0036513	0.4785	0.85059	rs8082605	4.69E-14	0	2.49E-31	0	Heart_Left_Ventricle
ENSG00000205045	SLFN12L	17	33800708	33864880	ENSG00000029534	ANK1	8	41510739	41754280	0.019255	13.547	1.90E-17	2.03E-11	0.0036513	0.4785	0.85059	rs8082605	4.69E-14	0	2.49E-31	0	Nerve_Tibial
ENSG00000205045	SLFN12L	17	33800708	33864880	ENSG00000029534	ANK1	8	41510739	41754280	0.019255	13.547	1.90E-17	2.03E-11	0.0036513	0.4785	0.85059	rs10512472	4.67E-16	0	4.96E-30	0	Pancreas
ENSG00000006125	AP2B1	17	33913918	34053436	ENSG00000095303	PTGS1	9	125132824	125157982	0.0192	13.507	2.16E-17	2.29E-11	0.067205	10.987	7.60E-12	rs10512472	8.41E-119	0	3.62E-14	0	Heart_Left_Ventricle
ENSG00000006125	AP2B1	17	33913918	34053436	ENSG00000166947	EPB42	15	43398423	43513481	0.019197	13.505	2.18E-17	2.30E-11	0.0096765	1.4901	0.17833	rs10512472	2.95E-21	0	3.62E-14	0	Heart_Left_Ventricle
ENSG00000163946	FAM208A	3	56658507	56717265	ENSG00000161911	TREML1	6	41117080	41122075	0.019753	12.164	2.32E-17	2.43E-11	0.025546	3.4231	0.0012773	rs12485738	3.76E-194	0	NA	NA	Esophagus_Mucosa
ENSG00000163946	FAM208A	3	56658507	56717265	ENSG00000161911	TREML1	6	41117080	41122075	0.019753	12.164	2.32E-17	2.43E-11	0.025546	3.4231	0.0012773	rs1354034	3.27170000000001e-	0	NA	NA	Heart_Atrial_Appendage
ENSG00000132139	GAS2L2	17	34071530	34079897	ENSG00000158856	DMTN	8	21906506	21940038	0.019701	12.131	2.62E-17	2.74E-11	NA	NA	NA	rs9915021	7.02E-65	0	NA	NA	Small_Intestine_Terminal_Ileum
ENSG00000172716	SLFN11	17	33677324	33700720	ENSG00000070182	SPTB	14	65216372	65346601	0.021827	8.9723	2.82E-17	2.93E-11	0.013654	1.0486	0.40139	rs225245	8.72E-09	0.00014844	5.77E-07	0.001884469	Cells_Transformed_fibroblasts
ENSG00000006125	AP2B1	17	33913918	34053436	ENSG00000115649	CNPPD1	2	220036619	220042035	0.019048	13.398	3.08E-17	3.19E-11	NA	NA	NA	rs10512472	2.37E-29	0	3.62E-14	0	Heart_Left_Ventricle
ENSG00000163946	FAM208A	3	56658507	56717265	ENSG00000138722	MMRN1	4	90800683	90875780	0.01959	12.062	3.38E-17	3.48E-11	0.034768	4.7032	3.38E-05	rs12485738	9.40E-304	0	NA	NA	Esophagus_Mucosa
ENSG00000163946	FAM208A	3	56658507	56717265	ENSG00000138722	MMRN1	4	90800683	90875780	0.01959	12.062	3.38E-17	3.48E-11	0.034768	4.7032	3.38E-05	rs1354034	3.27170000000001e-	0	NA	NA	Heart_Atrial_Appendage
ENSG00000006125	AP2B1	17	33913918	34053436	ENSG00000029534	ANK1	8	41510739	41754280	0.018973	13.344	3.67E-17	3.76E-11	0.02106	3.2807	0.003373	rs10512472	4.67E-16	0	3.62E-14	0	Heart_Left_Ventricle
ENSG00000108733	PEX12	17	33901814	33905882	ENSG00000158828	PINK1	1	20959948	20978004	0.017688	17.402	4.09E-17	4.15E-11	0.0036093	0.66362	0.65116	rs9915021	5.07E-27	0	NA	NA	Brain_Caudate_basal_ganglia
ENSG00000108733	PEX12	17	33901814	33905882	ENSG00000158828	PINK1	1	20959948	20978004	0.017688	17.402	4.09E-17	4.15E-11	0.0036093	0.66362	0.65116	rs9915021	5.07E-27	0	NA	NA	Breast_Mammary_Tissue
ENSG00000108733	PEX12	17	33901814	33905882	ENSG00000158828	PINK1	1	20959948	20978004	0.017688	17.402	4.09E-17	4.15E-11	0.0036093	0.66362	0.65116	rs10512472	1.16E-16	0	1.06E-12	0	Brain_Caudate_basal_ganglia
ENSG00000108733	PEX12	17	33901814	33905882	ENSG00000113140	SPARC	5	151040657	151066726	0.017487	17.2	6.61E-17	6.54E-11	0.029101	5.4911	5.46E-05	rs9915021	8.30E-44	0	NA	NA	Brain_Caudate_basal_ganglia
ENSG00000108733	PEX12	17	33901814	33905882	ENSG00000113140	SPARC	5	151040657	151066726	0.017487	17.2	6.61E-17	6.54E-11	0.029101	5.4911	5.46E-05	rs9915021	8.30E-44	0	NA	NA	Breast_Mammary_Tissue
ENSG00000108733	PEX12	17	33901814	33905882	ENSG00000113140	SPARC	5	151040657	151066726	0.017487	17.2	6.61E-17	6.54E-11	0.029101	5.4911	5.46E-05	rs10512472	7.03E-109	0	1.06E-12	0	Brain_Caudate_basal_ganglia
ENSG00000006125	AP2B1	17	33913918	34053436	ENSG00000144677	CTDSPL	3	37903451	38025960	0.018652	13.114	7.76E-17	7.54E-11	0.053393	8.6016	4.06E-09	rs10512472	7.69E-165	0	3.62E-14	0	Heart_Left_Ventricle
ENSG00000108733	PEX12	17	33901814	33905882	ENSG00000238243	OR2W3	1	248058859	248060449	0.01736	17.073	8.93E-17	8.55E-11	0.011473	2.1262	0.060214	rs9915021	7.68E-06	0.04691593	NA	NA	Brain_Caudate_basal_ganglia
ENSG00000108733	PEX12	17	33901814	33905882	ENSG00000238243	OR2W3	1	248058859	248060449	0.01736	17.073	8.93E-17	8.55E-11	0.011473	2.1262	0.060214	rs9915021	7.68E-06	0.04691593	NA	NA	Breast_Mammary_Tissue
ENSG00000205045	SLFN12L	17	33800708	33864880	ENSG00000167671	UBXN6	19	4446046	4457819	0.018549	13.041	9.85E-17	9.39E-11	0.023147	3.094	0.0031412	rs8082605	3.08E-60	0	2.49E-31	0	Adipose_Subcutaneous
ENSG00000205045	SLFN12L	17	33800708	33864880	ENSG00000167671	UBXN6	19	4446046	4457819	0.018549	13.041	9.85E-17	9.39E-11	0.023147	3.094	0.0031412	rs8082605	3.08E-60	0	2.49E-31	0	Adipose_Visceral_Omentum
ENSG00000205045	SLFN12L	17	33800708	33864880	ENSG00000167671	UBXN6	19	4446046	4457819	0.018549	13.041	9.85E-17	9.39E-11	0.023147	3.094	0.0031412	rs8082605	3.08E-60	0	2.49E-31	0	Heart_Left_Ventricle
ENSG00000205045	SLFN12L	17	33800708	33864880	ENSG00000167671	UBXN6	19	4446046	4457819	0.018549	13.041	9.85E-17	9.39E-11	0.023147	3.094	0.0031412	rs8082605	3.08E-60	0	2.49E-31	0	Nerve_Tibial
ENSG00000205045	SLFN12L	17	33800708	33864880	ENSG00000167671	UBXN6	19	4446046	4457819	0.018549	13.041	9.85E-17	9.39E-11	0.023147	3.094	0.0031412	rs10512472	4.51E-73	0	4.96E-30	0	Pancreas
ENSG00000168374	ARF4	3	57557090	57583947	ENSG00000166086	JAM3	11	133938820	134018713	0.017199	16.912	1.31E-16	1.21E-10	0.021647	4.0534	0.0012078	rs17825630	4.26E-10	2.12E-05	NA	NA	Artery_Tibial
ENSG00000168374	ARF4	3	57557090	57583947	ENSG00000166086	JAM3	11	133938820	134018713	0.017199	16.912	1.31E-16	1.21E-10	0.021647	4.0534	0.0012078	rs17825630	4.26E-10	2.12E-05	NA	NA	Heart_Atrial_Appendage
ENSG00000172660	TAF15	17	34136459	34174246	ENSG00000103148	NPRL3	16	138697	188859	0.017748	14.548	1.58E-16	1.45E-10	0.013403	2.0718	0.054126	rs9915021	1.32E-25	0	NA	NA	Skin_Not_Sun_Exposed_Suprapubic
ENSG00000205045	SLFN12L	17	33800708	33864880	ENSG00000146535	GNA12	7	2767746	2883958	0.018269	12.84	1.89E-16	1.71E-10	0.014015	1.8559	0.073703	rs8082605	3.00E-24	0	2.49E-31	0	Adipose_Subcutaneous
ENSG00000205045	SLFN12L	17	33800708	33864880	ENSG00000146535	GNA12	7	2767746	2883958	0.018269	12.84	1.89E-16	1.71E-10	0.014015	1.8559	0.073703	rs8082605	3.00E-24	0	2.49E-31	0	Adipose_Visceral_Omentum
ENSG00000205045	SLFN12L	17	33800708	33864880	ENSG00000146535	GNA12	7	2767746	2883958	0.018269	12.84	1.89E-16	1.71E-10	0.014015	1.8559	0.073703	rs8082605	3.00E-24	0	2.49E-31	0	Heart_Left_Ventricle
ENSG00000205045	SLFN12L	17	33800708	33864880	ENSG00000146535	GNA12	7	2767746	2883958	0.018269	12.84	1.89E-16	1.71E-10	0.014015	1.8559	0.073703	rs8082605	3.00E-24	0	2.49E-31	0	Nerve_Tibial
ENSG00000205045	SLFN12L	17	33800708	33864880	ENSG00000146535	GNA12	7	2767746	2883958	0.018269	12.84	1.89E-16	1.71E-10	0.014015	1.8559	0.073703	rs10512472	1.14E-18	0	4.96E-30	0	Pancreas
ENSG00000132436	FIGNL1	7	50511831	50518088	ENSG00000017483	SLC38A5	X	48316920	48328644	0.013877	68.055	2.03E-16	1.82E-10	0.051201	49.647	3.61E-12	rs12718598	2.44E-239	0	2.35E-199	0	Adipose_Visceral_Omentum
ENSG00000132436	FIGNL1	7	50511831	50518088	ENSG00000017483	SLC38A5	X	48316920	48328644	0.013877	68.055	2.03E-16	1.82E-10	0.051201	49.647	3.61E-12	rs12718598	2.44E-239	0	2.35E-199	0	Artery_Coronary
ENSG00000132436	FIGNL1	7	50511831	50518088	ENSG00000017483	SLC38A5	X	48316920	48328644	0.013877	68.055	2.03E-16	1.82E-10	0.051201	49.647	3.61E-12	rs12718598	2.44E-239	0	2.35E-199	0	Colon_Sigmoid
ENSG00000132436	FIGNL1	7	50511831	50518088	ENSG00000017483	SLC38A5	X	48316920	48328644	0.013877	68.055	2.03E-16	1.82E-10	0.051201	49.647	3.61E-12	rs12718598	2.44E-239	0	2.35E-199	0	Lung
ENSG00000132436	FIGNL1	7	50511831	50518088	ENSG00000017483	SLC38A5	X	48316920	48328644	0.013877	68.055	2.03E-16	1.82E-10	0.051201	49.647	3.61E-12	rs12718598	2.44E-239	0	2.35E-199	0	Pituitary
ENSG00000132436	FIGNL1	7	50511831	50518088	ENSG00000017483	SLC38A5	X	48316920	48328644	0.013877	68.055	2.03E-16	1.82E-10	0.051201	49.647	3.61E-12	rs12718598	2.44E-239	0	2.35E-199	0	Spleen
ENSG00000132436	FIGNL1	7	50511831	50518088	ENSG00000017483	SLC38A5	X	48316920	48328644	0.013877	68.055	2.03E-16	1.82E-10	0.051201	49.647	3.61E-12	rs12718598	2.44E-239	0	2.35E-199	0	Vagina
ENSG00000132436	FIGNL1	7	50511831	50518088	ENSG00000017483	SLC38A5	X	48316920	48328644	0.013877	68.055	2.03E-16	1.82E-10	0.051201	49.647	3.61E-12	rs12718597	8.58E-146	0	1.76E-281	0	Brain_Hippocampus
ENSG00000132436	FIGNL1	7	50511831	50518088	ENSG00000017483	SLC38A5	X	48316920	48328644	0.013877	68.055	2.03E-16	1.82E-10	0.051201	49.647	3.61E-12	rs12718597	8.58E-146	0	1.76E-281	0	Vagina
ENSG00000006125	AP2B1	17	33913918	34053436	ENSG00000158828	PINK1	1	20959948	20978004	0.018207	12.796	2.18E-16	1.95E-10	0.0078507	1.2067	0.30032	rs10512472	1.16E-16	0	3.62E-14	0	Heart_Left_Ventricle
ENSG00000205045	SLFN12L	17	33800708	33864880	ENSG00000113140	SPARC	5	151040657	151066726	0.018167	12.767	2.40E-16	2.13E-10	0.016255	2.1575	0.035743	rs8082605	8.71E-47	0	2.49E-31	0	Adipose_Subcutaneous
ENSG00000205045	SLFN12L	17	33800708	33864880	ENSG00000113140	SPARC	5	151040657	151066726	0.018167	12.767	2.40E-16	2.13E-10	0.016255	2.1575	0.035743	rs8082605	8.71E-47	0	2.49E-31	0	Adipose_Visceral_Omentum
ENSG00000205045	SLFN12L	17	33800708	33864880	ENSG00000113140	SPARC	5	151040657	151066726	0.018167	12.767	2.40E-16	2.13E-10	0.016255	2.1575	0.035743	rs8082605	8.71E-47	0	2.49E-31	0	Heart_Left_Ventricle
ENSG00000205045	SLFN12L	17	33800708	33864880	ENSG00000113140	SPARC	5	151040657	151066726	0.018167	12.767	2.40E-16	2.13E-10	0.016255	2.1575	0.035743	rs8082605	8.71E-47	0	2.49E-31	0	Nerve_Tibial
ENSG00000205045	SLFN12L	17	33800708	33864880	ENSG00000113140	SPARC	5	151040657	151066726	0.018167	12.767	2.40E-16	2.13E-10	0.016255	2.1575	0.035743	rs10512472	7.03E-109	0	4.96E-30	0	Pancreas
ENSG00000167136	ENDOG	9	131580753	131581464	ENSG00000164821	DEFA4	8	6793344	6795860	0.016071	19.735	3.96E-16	3.38E-10	0.019786	2.6356	0.010626	rs13289095	1.50E-34	0	1.10E-185	0	Skin_Not_Sun_Exposed_Suprapubic
ENSG00000167136	ENDOG	9	131580753	131581464	ENSG00000164821	DEFA4	8	6793344	6795860	0.016071	19.735	3.96E-16	3.38E-10	0.019786	2.6356	0.010626	rs15676	1.50E-19	0	3.2717e-310	0	Cells_Transformed_fibroblasts
ENSG00000167136	ENDOG	9	131580753	131581464	ENSG00000164821	DEFA4	8	6793344	6795860	0.016071	19.735	3.96E-16	3.38E-10	0.019786	2.6356	0.010626	rs15676	1.50E-19	0	3.2717e-310	0	Esophagus_Mucosa
ENSG00000167136	ENDOG	9	131580753	131581464	ENSG00000164821	DEFA4	8	6793344	6795860	0.016071	19.735	3.96E-16	3.38E-10	0.019786	2.6356	0.010626	rs15676	1.50E-19	0	3.2717e-310	0	Ovary
ENSG00000167136	ENDOG	9	131580753	131581464	ENSG00000164821	DEFA4	8	6793344	6795860	0.016071	19.735	3.96E-16	3.38E-10	0.019786	2.6356	0.010626	rs15676	1.50E-19	0	3.2717e-310	0	Thyroid
ENSG00000205045	SLFN12L	17	33800708	33864880	ENSG00000167992	VWCE	11	61025762	61062896	0.017858	12.546	4.92E-16	4.12E-10	0.026359	3.5349	0.00093718	rs8082605	8.92E-35	0	2.49E-31	0	Adipose_Subcutaneous
ENSG00000205045	SLFN12L	17	33800708	33864880	ENSG00000167992	VWCE	11	61025762	61062896	0.017858	12.546	4.92E-16	4.12E-10	0.026359	3.5349	0.00093718	rs8082605	8.92E-35	0	2.49E-31	0	Adipose_Visceral_Omentum
ENSG00000205045	SLFN12L	17	33800708	33864880	ENSG00000167992	VWCE	11	61025762	61062896	0.017858	12.546	4.92E-16	4.12E-10	0.026359	3.5349	0.00093718	rs8082605	8.92E-35	0	2.49E-31	0	Heart_Left_Ventricle
ENSG00000205045	SLFN12L	17	33800708	33864880	ENSG00000167992	VWCE	11	61025762	61062896	0.017858	12.546	4.92E-16	4.12E-10	0.026359	3.5349	0.00093718	rs8082605	8.92E-35	0	2.49E-31	0	Nerve_Tibial
ENSG00000205045	SLFN12L	17	33800708	33864880	ENSG00000167992	VWCE	11	61025762	61062896	0.017858	12.546	4.92E-16	4.12E-10	0.026359	3.5349	0.00093718	rs10512472	2.21E-55	0	4.96E-30	0	Pancreas
ENSG00000172660	TAF15	17	34136459	34174246	ENSG00000029534	ANK1	8	41510739	41754280	0.017143	14.044	6.53E-16	5.42E-10	0.012248	1.8909	0.079527	rs9915021	6.17E-12	0	NA	NA	Skin_Not_Sun_Exposed_Suprapubic
ENSG00000205045	SLFN12L	17	33800708	33864880	ENSG00000120885	CLU	8	27454434	27472548	0.017708	12.439	6.97E-16	5.76E-10	0.018045	2.3995	0.019513	rs8082605	4.14E-10	2.12E-05	2.49E-31	0	Adipose_Subcutaneous
ENSG00000205045	SLFN12L	17	33800708	33864880	ENSG00000120885	CLU	8	27454434	27472548	0.017708	12.439	6.97E-16	5.76E-10	0.018045	2.3995	0.019513	rs8082605	4.14E-10	2.12E-05	2.49E-31	0	Adipose_Visceral_Omentum
ENSG00000205045	SLFN12L	17	33800708	33864880	ENSG00000120885	CLU	8	27454434	27472548	0.017708	12.439	6.97E-16	5.76E-10	0.018045	2.3995	0.019513	rs8082605	4.14E-10	2.12E-05	2.49E-31	0	Heart_Left_Ventricle
ENSG00000205045	SLFN12L	17	33800708	33864880	ENSG00000120885	CLU	8	27454434	27472548	0.017708	12.439	6.97E-16	5.76E-10	0.018045	2.3995	0.019513	rs8082605	4.14E-10	2.12E-05	2.49E-31	0	Nerve_Tibial
ENSG00000205045	SLFN12L	17	33800708	33864880	ENSG00000120885	CLU	8	27454434	27472548	0.017708	12.439	6.97E-16	5.76E-10	0.018045	2.3995	0.019513	rs10512472	1.27E-27	0	4.96E-30	0	Pancreas
ENSG00000092871	RFFL	17	33341759	33416338	ENSG00000158856	DMTN	8	21906506	21940038	0.017091	14	7.37E-16	6.07E-10	NA	NA	NA	rs9915021	7.02E-65	0	NA	NA	Adipose_Subcutaneous
ENSG00000092871	RFFL	17	33341759	33416338	ENSG00000158856	DMTN	8	21906506	21940038	0.017091	14	7.37E-16	6.07E-10	NA	NA	NA	rs9915021	7.02E-65	0	NA	NA	Artery_Tibial
ENSG00000205045	SLFN12L	17	33800708	33864880	ENSG00000144677	CTDSPL	3	37903451	38025960	0.017633	12.385	8.30E-16	6.78E-10	0.029205	3.9281	0.00031123	rs8082605	1.17E-78	0	2.49E-31	0	Adipose_Subcutaneous
ENSG00000205045	SLFN12L	17	33800708	33864880	ENSG00000144677	CTDSPL	3	37903451	38025960	0.017633	12.385	8.30E-16	6.78E-10	0.029205	3.9281	0.00031123	rs8082605	1.17E-78	0	2.49E-31	0	Adipose_Visceral_Omentum
ENSG00000205045	SLFN12L	17	33800708	33864880	ENSG00000144677	CTDSPL	3	37903451	38025960	0.017633	12.385	8.30E-16	6.78E-10	0.029205	3.9281	0.00031123	rs8082605	1.17E-78	0	2.49E-31	0	Heart_Left_Ventricle
ENSG00000205045	SLFN12L	17	33800708	33864880	ENSG00000144677	CTDSPL	3	37903451	38025960	0.017633	12.385	8.30E-16	6.78E-10	0.029205	3.9281	0.00031123	rs8082605	1.17E-78	0	2.49E-31	0	Nerve_Tibial
ENSG00000205045	SLFN12L	17	33800708	33864880	ENSG00000144677	CTDSPL	3	37903451	38025960	0.017633	12.385	8.30E-16	6.78E-10	0.029205	3.9281	0.00031123	rs10512472	7.69E-165	0	4.96E-30	0	Pancreas
ENSG00000172660	TAF15	17	34136459	34174246	ENSG00000238243	OR2W3	1	248058859	248060449	0.016879	13.824	1.21E-15	9.73E-10	0.005008	0.76756	0.59553	rs9915021	7.68E-06	0.04691593	NA	NA	Skin_Not_Sun_Exposed_Suprapubic

ENSG00000198917	C9orf114	9	131586026	131592100	ENSG00000164821	DEFA4	8	6793344	6795860	0.015581	19.124	1.28E-15	1.02E-09	0.015112	4.6953	0.0029236	rs15676	1.50E-19	0	NA	NA	Breast_Mammary_Tissue
ENSG00000205045	SLFN12L	17	33800708	33864880	ENSG00000138867	GUCD1	22	24936406	24951284	0.017279	12.132	1.88E-15	1.49E-09	NA	NA	NA	rs8082605	2.66E-13	0	2.49E-31	0	Adipose_Subcutaneous
ENSG00000205045	SLFN12L	17	33800708	33864880	ENSG00000138867	GUCD1	22	24936406	24951284	0.017279	12.132	1.88E-15	1.49E-09	NA	NA	NA	rs8082605	2.66E-13	0	2.49E-31	0	Adipose_Visceral_Omentum
ENSG00000205045	SLFN12L	17	33800708	33864880	ENSG00000138867	GUCD1	22	24936406	24951284	0.017279	12.132	1.88E-15	1.49E-09	NA	NA	NA	rs8082605	2.66E-13	0	2.49E-31	0	Heart_Left_Ventricle
ENSG00000205045	SLFN12L	17	33800708	33864880	ENSG00000138867	GUCD1	22	24936406	24951284	0.017279	12.132	1.88E-15	1.49E-09	NA	NA	NA	rs8082605	2.66E-13	0	2.49E-31	0	Nerve_Tibial
ENSG00000205045	SLFN12L	17	33800708	33864880	ENSG00000138867	GUCD1	22	24936406	24951284	0.017279	12.132	1.88E-15	1.49E-09	NA	NA	NA	rs10512472	1.08E-10	0	4.96E-30	0	Pancreas
ENSG00000108733	PEX12	17	33901814	33905882	ENSG00000120885	CLU	8	27454434	27472548	0.016017	15.731	2.15E-15	1.68E-09	0.02249	4.2149	0.00085725	rs9915021	2.20E-17	0	NA	NA	Brain_Caudate_basal_ganglia
ENSG00000108733	PEX12	17	33901814	33905882	ENSG00000120885	CLU	8	27454434	27472548	0.016017	15.731	2.15E-15	1.68E-09	0.02249	4.2149	0.00085725	rs9915021	2.20E-17	0	NA	NA	Breast_Mammary_Tissue
ENSG00000108733	PEX12	17	33901814	33905882	ENSG00000120885	CLU	8	27454434	27472548	0.016017	15.731	2.15E-15	1.68E-09	0.02249	4.2149	0.00085725	rs10512472	1.27E-27	0	1.06E-12	0	Brain_Caudate_basal_ganglia
ENSG00000006125	AP2B1	17	33913918	34053436	ENSG00000128266	GNAZ	22	23412540	23464889	0.017097	12.002	2.87E-15	2.21E-09	0.014381	2.225	0.03876	rs10512472	4.37E-21	0	3.62E-14	0	Heart_Left_Ventricle
ENSG00000205045	SLFN12L	17	33800708	33864880	ENSG00000238243	OR2W3	1	248058859	248060449	0.016989	11.925	3.68E-15	2.81E-09	0.005623	0.73835	0.63947	rs8082605	1.50E-06	0.01173846	2.49E-31	0	Adipose_Subcutaneous
ENSG00000205045	SLFN12L	17	33800708	33864880	ENSG00000238243	OR2W3	1	248058859	248060449	0.016989	11.925	3.68E-15	2.81E-09	0.005623	0.73835	0.63947	rs8082605	1.50E-06	0.01173846	2.49E-31	0	Adipose_Visceral_Omentum
ENSG00000205045	SLFN12L	17	33800708	33864880	ENSG00000238243	OR2W3	1	248058859	248060449	0.016989	11.925	3.68E-15	2.81E-09	0.005623	0.73835	0.63947	rs8082605	1.50E-06	0.01173846	2.49E-31	0	Heart_Left_Ventricle
ENSG00000205045	SLFN12L	17	33800708	33864880	ENSG00000238243	OR2W3	1	248058859	248060449	0.016989	11.925	3.68E-15	2.81E-09	0.005623	0.73835	0.63947	rs8082605	1.50E-06	0.01173846	2.49E-31	0	Nerve_Tibial
ENSG00000006125	AP2B1	17	33913918	34053436	ENSG00000100614	PPM1A	14	60712470	60765805	0.016916	11.873	4.36E-15	3.31E-09	0.014985	2.32	0.031413	rs10512472	4.22E-11	0	3.62E-14	0	Heart_Left_Ventricle
ENSG00000142082	SIRT3	11	215458	236431	ENSG00000259207	ITGB3	17	45387505	45389182	0.01431	23.393	5.00E-15	3.76E-09	0.009518	2.203	0.066856	rs11602954	1.90E-32	0	1.39E-80	0	Adipose_Subcutaneous
ENSG00000142082	SIRT3	11	215458	236431	ENSG00000259207	ITGB3	17	45387505	45389182	0.01431	23.393	5.00E-15	3.76E-09	0.009518	2.203	0.066856	rs11602954	1.90E-32	0	1.39E-80	0	Artery_Tibial
ENSG00000142082	SIRT3	11	215458	236431	ENSG00000259207	ITGB3	17	45387505	45389182	0.01431	23.393	5.00E-15	3.76E-09	0.009518	2.203	0.066856	rs11602954	1.90E-32	0	1.39E-80	0	Breast_Mammary_Tissue
ENSG00000142082	SIRT3	11	215458	236431	ENSG00000259207	ITGB3	17	45387505	45389182	0.01431	23.393	5.00E-15	3.76E-09	0.009518	2.203	0.066856	rs11602954	1.90E-32	0	1.39E-80	0	Cells_Transformed_fibroblasts
ENSG00000142082	SIRT3	11	215458	236431	ENSG00000259207	ITGB3	17	45387505	45389182	0.01431	23.393	5.00E-15	3.76E-09	0.009518	2.203	0.066856	rs11602954	1.90E-32	0	1.39E-80	0	Nerve_Tibial
ENSG00000142082	SIRT3	11	215458	236431	ENSG00000259207	ITGB3	17	45387505	45389182	0.01431	23.393	5.00E-15	3.76E-09	0.009518	2.203	0.066856	rs505404	5.02E-41	0	1.05E-94	0	Adrenal_Gland
ENSG00000142082	SIRT3	11	215458	236431	ENSG00000259207	ITGB3	17	45387505	45389182	0.01431	23.393	5.00E-15	3.76E-09	0.009518	2.203	0.066856	rs505404	5.02E-41	0	1.05E-94	0	Artery_Aorta
ENSG00000142082	SIRT3	11	215458	236431	ENSG00000259207	ITGB3	17	45387505	45389182	0.01431	23.393	5.00E-15	3.76E-09	0.009518	2.203	0.066856	rs505404	5.02E-41	0	1.05E-94	0	Lung
ENSG00000142082	SIRT3	11	215458	236431	ENSG00000259207	ITGB3	17	45387505	45389182	0.01431	23.393	5.00E-15	3.76E-09	0.009518	2.203	0.066856	rs505404	5.02E-41	0	1.05E-94	0	Thyroid
ENSG00000142082	SIRT3	11	215458	236431	ENSG00000259207	ITGB3	17	45387505	45389182	0.01431	23.393	5.00E-15	3.76E-09	0.009518	2.203	0.066856	rs3782123	1.71E-09	5.69E-05	NA	NA	Brain_Frontal_Cortex_BA9
ENSG00000106004	HOXA5	7	27180671	27183287	ENSG00000176358	TAC4	17	47915671	47925379	0.016785	11.78	5.90E-15	4.40E-09	0.0069504	0.91387	0.49477	rs706015	3.17E-13	0	2.87E-09	2.60E-05	Skin_Not_Sun_Exposed_Suprapubic
ENSG00000205045	SLFN12L	17	33800708	33864880	ENSG00000164068	RNF123	3	49728563	49753910	0.016781	11.776	5.97E-15	4.43E-09	0.019392	2.5821	0.012211	rs8082605	5.08E-22	0	2.49E-31	0	Adipose_Subcutaneous
ENSG00000205045	SLFN12L	17	33800708	33864880	ENSG00000164068	RNF123	3	49728563	49753910	0.016781	11.776	5.97E-15	4.43E-09	0.019392	2.5821	0.012211	rs8082605	5.08E-22	0	2.49E-31	0	Adipose_Visceral_Omentum
ENSG00000205045	SLFN12L	17	33800708	33864880	ENSG00000164068	RNF123	3	49728563	49753910	0.016781	11.776	5.97E-15	4.43E-09	0.019392	2.5821	0.012211	rs8082605	5.08E-22	0	2.49E-31	0	Heart_Left_Ventricle
ENSG00000205045	SLFN12L	17	33800708	33864880	ENSG00000164068	RNF123	3	49728563	49753910	0.016781	11.776	5.97E-15	4.43E-09	0.019392	2.5821	0.012211	rs8082605	5.08E-22	0	2.49E-31	0	Nerve_Tibial
ENSG00000205045	SLFN12L	17	33800708	33864880	ENSG00000164068	RNF123	3	49728563	49753910	0.016781	11.776	5.97E-15	4.43E-09	0.019392	2.5821	0.012211	rs10512472	3.54E-12	0	4.96E-30	0	Pancreas
ENSG00000141150	RASL10B	17	34058668	34070540	ENSG00000022840	RNF10	12	120971283	121015397	0.017298	10.625	6.59E-15	4.88E-09	0.018721	2.4911	0.015443	rs9915021	1.07E-57	0	NA	NA	Cells_Transformed_fibroblasts
ENSG00000172660	TAF15	17	34136459	34174246	ENSG00000115649	CNPPD1	2	220036619	220042035	0.016152	13.219	6.62E-15	4.88E-09	NA	NA	NA	rs9915021	3.39E-19	0	NA	NA	Skin_Not_Sun_Exposed_Suprapubic
ENSG00000154760	SLFN13	17	33762115	33775856	ENSG00000103148	NPRL3	16	138697	188859	0.018845	8.4266	7.07E-15	5.16E-09	0.030891	2.4146	0.0043955	rs225245	1.20E-79	0	NA	NA	Liver
ENSG00000154760	SLFN13	17	33762115	33775856	ENSG00000103148	NPRL3	16	138697	188859	0.018845	8.4266	7.07E-15	5.16E-09	0.030891	2.4146	0.0043955	rs8082605	5.54E-34	0	2.08E-19	0	Artery_Tibial
ENSG00000154760	SLFN13	17	33762115	33775856	ENSG00000103148	NPRL3	16	138697	188859	0.018845	8.4266	7.07E-15	5.16E-09	0.030891	2.4146	0.0043955	rs8082605	5.54E-34	0	2.08E-19	0	Esophagus_Mucosa
ENSG00000154760	SLFN13	17	33762115	33775856	ENSG00000103148	NPRL3	16	138697	188859	0.018845	8.4266	7.07E-15	5.16E-09	0.030891	2.4146	0.0043955	rs8082605	5.54E-34	0	2.08E-19	0	Nerve_Tibial
ENSG00000154760	SLFN13	17	33762115	33775856	ENSG00000103148	NPRL3	16	138697	188859	0.018845	8.4266	7.07E-15	5.16E-09	0.030891	2.4146	0.0043955	rs8082605	5.54E-34	0	2.08E-19	0	Pituitary
ENSG00000154760	SLFN13	17	33762115	33775856	ENSG00000103148	NPRL3	16	138697	188859	0.018845	8.4266	7.07E-15	5.16E-09	0.030891	2.4146	0.0043955	rs10512472	6.78E-42	0	6.34E-38	0	Uterus
ENSG00000205045	SLFN12L	17	33800708	33864880	ENSG00000095303	PTGS1	9	125132824	125157982	0.016692	11.713	7.33E-15	5.33E-09	0.034687	4.6918	3.49E-05	rs8082605	6.10E-57	0	2.49E-31	0	Adipose_Subcutaneous
ENSG00000205045	SLFN12L	17	33800708	33864880	ENSG00000095303	PTGS1	9	125132824	125157982	0.016692	11.713	7.33E-15	5.33E-09	0.034687	4.6918	3.49E-05	rs8082605	6.10E-57	0	2.49E-31	0	Adipose_Visceral_Omentum
ENSG00000205045	SLFN12L	17	33800708	33864880	ENSG00000095303	PTGS1	9	125132824	125157982	0.016692	11.713	7.33E-15	5.33E-09	0.034687	4.6918	3.49E-05	rs8082605	6.10E-57	0	2.49E-31	0	Heart_Left_Ventricle
ENSG00000205045	SLFN12L	17	33800708	33864880	ENSG00000095303	PTGS1	9	125132824	125157982	0.016692	11.713	7.33E-15	5.33E-09	0.034687	4.6918	3.49E-05	rs8082605	6.10E-57	0	2.49E-31	0	Nerve_Tibial
ENSG00000205045	SLFN12L	17	33800708	33864880	ENSG00000095303	PTGS1	9	125132824	125157982	0.016692	11.713	7.33E-15	5.33E-09	0.034687	4.6918	3.49E-05	rs10512472	8.41E-119	0	4.96E-30	0	Pancreas
ENSG00000172660	TAF15	17	34136459	34174246	ENSG00000166947	EPB42	15	43398423	43513481	0.016065	13.146	8.13E-15	5.87E-09	0.010195	1.5708	0.15244	rs9915021	3.48E-18	0	NA	NA	Skin_Not_Sun_Exposed_Suprapubic
ENSG00000172660	TAF15	17	34136459	34174246	ENSG00000158828	PINK1	1	20959948	20978004	0.01604	13.125	8.61E-15	6.19E-09	0.012027	1.8564	0.085476	rs9915021	5.07E-27	0	NA	NA	Skin_Not_Sun_Exposed_Suprapubic
ENSG00000154760	SLFN13	17	33762115	33775856	ENSG00000173068	BNC2	9	16409501	16870841	0.018571	8.3019	1.30E-14	9.15E-09	0.045493	3.6103	2.69E-05	rs10512472	7.97E-06	0.04836275	6.34E-38	0	Uterus
ENSG00000006125	AP2B1	17	33913918	34053436	ENSG00000154146	NRGN	11	124609829	124615878	0.016425	11.522	1.36E-14	9.46E-09	0.022245	3.4696	0.0021385	rs10512472	4.87E-94	0	3.62E-14	0	Heart_Left_Ventricle
ENSG00000177963	RIC8A	11	207511	215113	ENSG00000138722	MMRN1	4	90800683	90875780	0.015237	14.953	1.36E-14	9.46E-09	0.0074385	0.85528	0.55409	rs11602954	5.94E-65	0	7.55E-62	0	Adipose_Subcutaneous
ENSG00000177963	RIC8A	11	207511	215113	ENSG00000138722	MMRN1	4	90800683	90875780	0.015237	14.953	1.36E-14	9.46E-09	0.0074385	0.85528	0.55409	rs11602954	5.94E-65	0	7.55E-62	0	Artery_Aorta
ENSG00000177963	RIC8A	11	207511	215113	ENSG00000138722	MMRN1	4	90800683	90875780	0.015237	14.953	1.36E-14	9.46E-09	0.0074385	0.85528	0.55409	rs11602954	5.94E-65	0	7.55E-62	0	Artery_Tibial
ENSG00000177963	RIC8A	11	207511	215113	ENSG00000138722	MMRN1	4	90800683	90875780	0.015237	14.953	1.36E-14	9.46E-09	0.0074385	0.85528	0.55409	rs11602954	5.94E-65	0	7.55E-62	0	Brain_Caudate_basal_ganglia
ENSG00000177963	RIC8A	11	207511	215113	ENSG00000138722	MMRN1	4	90800683	90875780	0.015237	14.953	1.36E-14	9.46E-09	0.0074385	0.85528	0.55409	rs11602954	5.94E-65	0	7.55E-62	0	Brain_Frontal_Cortex_BA9
ENSG00000177963	RIC8A	11	207511	215113	ENSG00000138722	MMRN1	4	90800683	90875780	0.015237	14.953	1.36E-14	9.46E-09	0.0074385	0.85528	0.55409	rs11602954	5.94E-65	0	7.55E-62	0	Colon_Sigmoid
ENSG00000177963	RIC8A	11	207511	215113	ENSG00000138722	MMRN1	4	90800683	90875780	0.015237	14.953	1.36E-14	9.46E-09	0.0074385	0.85528	0.55409	rs11602954	5.94E-65	0	7.55E-62	0	Colon_Transverse
ENSG00000177963	RIC8A	11	207511	215113	ENSG00000138722	MMRN1	4	90800683	90875780	0.015237	14.953	1.36E-14	9.46E-09	0.0074385	0.85528	0.55409	rs11602954	5.94E-65	0	7.55E-62	0	Esophagus_Gastroesophageal_Junction
ENSG00000177963	RIC8A	11	207511	215113	ENSG00000138722	MMRN1	4	90800683	90875780	0.015237	14.953	1.36E-14	9.46E-09	0.0074385	0.85528	0.55409	rs11602954	5.94E-65	0	7.55E-62	0	Esophagus_Mucosa
ENSG00000177963	RIC8A	11	207511	215113	ENSG00000138722	MMRN1	4	90800683	90875780	0.015237	14.953	1.36E-14	9.46E-09	0.0074385	0.85528	0.55409	rs11602954	5.94E-65	0	7.55E-62	0	Esophagus_Muscularis
ENSG00000177963	RIC8A	11	207511	215113	ENSG00000138722	MMRN1	4	90800683	90875780	0.015237	14.953	1.36E-14	9.46E-09	0.0074385	0.85528	0.55409	rs11602954	5.94E-65	0	7.55E-62	0	Lung
ENSG00000177963	RIC8A	11	207511	215113	ENSG00000138722	MMRN1	4	90800683	90875780	0.015237	14.953	1.36E-14	9.46E-09	0.0074385	0.85528	0.55409	rs11602954	5.94E-65	0	7.55E-62	0	Muscle_Skeletal
ENSG00000177963	RIC8A	11	207511	215113	ENSG00000138722	MMRN1	4	90800683	90875780	0.015237	14.953	1.36E-14	9.46E-09	0.0074385	0.85528	0.55409	rs11602954	5.94E-65	0	7.55E-62	0	Nerve_Tibial
ENSG00000177963	RIC8A	11	207511	215113	ENSG00000138722	MMRN1	4	90800683	90875780	0.015237	14.953	1.36E-14	9.46E-09	0.0074385	0.85528	0.55409	rs11602954	5.94E-65	0	7.55E-62	0	Skin_Not_Sun_Exposed_Suprapubic
ENSG00000177963	RIC8A	11	207511	215113	ENSG00000138722	MMRN1	4	90800683	90875780	0.015237	14.953	1.36E-14	9.46E-09	0.0074385	0.85528	0.55409	rs11602954	5.94E-65	0	7.55E-62	0	Skin_Sun_Exposed_Lower_leg
ENSG00000177963	RIC8A	11	207511	215113	ENSG00000138722	MMRN1	4	90800683	90875780	0.015237	14.953	1.36E-14	9.46E-09	0.0074385	0.85528	0.55409	rs11602954	5.94E-65	0	7.55E-62	0	Thyroid
ENSG00000177963	RIC8A	11	207511	215113	ENSG00000138722	MMRN1	4	90800683	90875780	0.015237	14.953	1.36E-14	9.46E-09	0.0074385	0.85528	0.55409	rs17655730	6.29E-64	0	5.10E-36	0	Adipose_Subcutaneous
ENSG00000177963	RIC8A	11	207511	215113	ENSG00000138722	MMRN1	4	90800683	90875780	0.015237	14.953	1.36E-14	9.46E-09	0.0074385	0.85528	0.55409	rs17655730	6.29E-64	0	5.10E-36	0	Esophagus_Mucosa
ENSG00000177963	RIC8A	11	207511	215113	ENSG00000138722	MMRN1	4	90800683	90875780	0.015237	14.953	1.36E-14	9.46E-09	0.0074385	0.85528	0.55409	rs17655730	6.29E-64	0	5.10E-36	0	Esophagus_Muscularis
ENSG00000177963	RIC8A	11	207511	215113	ENSG00000138722	MMRN1	4	90800683	90875780	0.015237	14.953	1.36E-14	9.46E-09	0.0074385	0.85528	0.55409	rs17655730	6.29E-64	0	5.10E-36	0	Heart_Left_Ventricle
ENSG00000177963	RIC8A	11	207511	215113	ENSG00000138722	MMRN1	4	90800683	90875780	0.015237	14.953	1.36E-14	9.46E-09	0.0074385	0.85528	0.55409	rs17655730	6.29E-64	0	5.10E-36	0	Ovary
ENSG00000177963	RIC8A	11	207511	215113	ENSG00000138722	MMRN1	4	90800683	90875780	0.015237	14.953	1.36E-14	9.46E-09	0.0074385	0.85528	0.55409	rs17655730	6.29E-64	0	5.10E-36	0	Pancreas
ENSG00000177963	RIC8A	11	207511	215113	ENSG00000138722	MMRN1	4	90800683	90875780	0.015237	14.953	1.36E-14	9.46E-09	0.0074385	0.85528	0.55409	rs17655730	6.29E-64	0	5.10E-36	0	Skin_Not_Sun_Exposed_Suprapubic
ENSG00000177963	RIC8A	11	207511	215113	ENSG00000138722	MMRN1	4	90800683	90875780	0.015237	14.953	1.36E-14	9.46E-09	0.0074385	0.85528	0.55409	rs7104764	2.04E-08	0.00024398	1.68E-167	0	Nerve_Tibial
ENSG00000177963	RIC8A	11	207511	215113	ENSG00000138722	MMRN1	4	90800683	90875780	0.015237	14.953	1.36E-14	9.46E-09	0.0074385	0.85528	0.55409	rs505404	2.01E-74	0	3.66E-47	0	Artery_Aorta
ENSG00000177963	RIC8A	11	207511	215113	ENSG00000138722	MMRN1	4	90800683	90875780	0.015237	14.953	1.36E-14	9.46E-09	0.0074385	0.85528	0.55409	rs505404	2.01E-74	0	3.66E-47	0	Thyroid
ENSG00000154760	SLFN13	17	33762115	33775856	ENSG00000115649	CNPPD1	2	220036619	220042035	0.018548	8.2914	1.37E-14	9.50E-09	NA	NA	NA	rs225245	1.17E-55	0	NA	NA	Liver
ENSG00000154760	SLFN13	17	33762115	33775856	ENSG00000115649	CNPPD1	2	220036619	220042035	0.018548	8.2914	1.37E-14	9.50E-09	NA	NA	NA	rs8082605	6.08E-30	0	2.08E-19	0	Artery_Tibial
ENSG00000154760	SLFN13	17	33762115	33775856	ENSG00000115649	CNPPD1	2	220036619	220042035	0.018548	8.2914	1.37E-14	9.50E-09	NA	NA	NA	rs8082605	6.08E-30	0	2.08E-19	0	Esophagus_Mucosa
ENSG00000154760	SLFN13	17	33762115	33775856	ENSG00000115649	CNPPD1	2	220036619	220042035	0.018548	8.2914	1.37E-14	9.50E-09	NA	NA	NA	rs8082605	6.08E-30	0	2.08E-19	0	Nerve_Tibial
ENSG00000154760	SLFN13	17	33762115	33775856	ENSG00000115649	CNPPD1	2	220036619	220042035	0.018548	8.2914	1.37E-14	9.50E-09	NA	NA	NA	rs8082605	6.08E-30	0	2.08E-19	0	Pituitary
ENSG00000154760	SLFN13	17	33762115	33775856	ENSG00000115649	CNPPD1	2	220036619	220042035	0.018548	8.2914	1.37E-14	9.50E-09	NA	NA	NA	rs10512472	2.37E-29	0	6.34E-38	0	Uterus
ENSG00000154760	SLFN13	17	33762115	33775856	ENSG00000238243	OR2W3	1	248058859	248060449	0.018544	8.2896	1.38E-14	9.55E-09	0.012282	0.9419	0.50384	rs225245	4.87E-16	0	NA	NA	Liver
ENSG00000154760	SLFN13	17	33762115	33775856	ENSG00000238243	OR2W3	1	248058859	248060449	0.018544	8.2896	1.38E-14	9.55E-09	0.012282	0.9419	0.50384	rs8082605	1.50E-06	0.01173846	2.08E-19	0	Artery_Tibial

ENSG00000154760	SLFN13	17	33762115	33775856	ENSG00000238243	OR2W3	1	248058859	248060449	0.018544	8.2896	1.38E-14	9.55E-09	0.012282	0.9419	0.50384	rs8082605	1.50E-06	0.01173846	2.08E-19	0	Esophagus_Mucosa
ENSG00000154760	SLFN13	17	33762115	33775856	ENSG00000238243	OR2W3	1	248058859	248060449	0.018544	8.2896	1.38E-14	9.55E-09	0.012282	0.9419	0.50384	rs8082605	1.50E-06	0.01173846	2.08E-19	0	Nerve_Tibial
ENSG00000154760	SLFN13	17	33762115	33775856	ENSG00000238243	OR2W3	1	248058859	248060449	0.018544	8.2896	1.38E-14	9.55E-09	0.012282	0.9419	0.50384	rs8082605	1.50E-06	0.01173846	2.08E-19	0	Pituitary
ENSG00000172660	TAF15	17	34136459	34174246	ENSG00000167671	UBXN6	19	4446046	4457819	0.015752	12.886	1.69E-14	1.16E-08	0.026814	4.2019	0.00035265	rs9915021	2.43E-46	0	NA	NA	Skin_Not_Sun_Exposed_Suprapubic
ENSG00000006125	AP2B1	17	33913918	34053436	ENSG00000138867	GUCD1	22	24936406	24951284	0.016305	11.437	1.79E-14	1.23E-08	NA	NA	NA	rs10512472	1.08E-10	0	3.62E-14	0	Heart_Left_Ventricle
ENSG00000205045	SLFN12L	17	33800708	33864880	ENSG00000204463	BAG6	6	31606805	31620170	0.016302	11.435	1.80E-14	1.23E-08	0.031185	4.2029	0.00014253	rs8082605	6.38E-11	0	2.49E-31	0	Adipose_Subcutaneous
ENSG00000205045	SLFN12L	17	33800708	33864880	ENSG00000204463	BAG6	6	31606805	31620170	0.016302	11.435	1.80E-14	1.23E-08	0.031185	4.2029	0.00014253	rs8082605	6.38E-11	0	2.49E-31	0	Adipose_Visceral_Omentum
ENSG00000205045	SLFN12L	17	33800708	33864880	ENSG00000204463	BAG6	6	31606805	31620170	0.016302	11.435	1.80E-14	1.23E-08	0.031185	4.2029	0.00014253	rs8082605	6.38E-11	0	2.49E-31	0	Heart_Left_Ventricle
ENSG00000205045	SLFN12L	17	33800708	33864880	ENSG00000204463	BAG6	6	31606805	31620170	0.016302	11.435	1.80E-14	1.23E-08	0.031185	4.2029	0.00014253	rs8082605	6.38E-11	0	2.49E-31	0	Nerve_Tibial
ENSG00000205045	SLFN12L	17	33800708	33864880	ENSG00000204463	BAG6	6	31606805	31620170	0.016302	11.435	1.80E-14	1.23E-08	0.031185	4.2029	0.00014253	rs10512472	7.83E-17	0	4.96E-30	0	Pancreas
ENSG00000011295	TTC19	17	15905229	15948329	ENSG00000169429	IL8	4	74606223	74609433	0.014446	17.71	1.92E-14	1.31E-08	0.02295	3.5821	0.0016267	rs7210990	1.42E-95	0	3.2717e-310	0	Adipose_Visceral_Omentum
ENSG00000011295	TTC19	17	15905229	15948329	ENSG00000169429	IL8	4	74606223	74609433	0.014446	17.71	1.92E-14	1.31E-08	0.02295	3.5821	0.0016267	rs7210990	1.42E-95	0	3.2717e-310	0	Colon_Transverse
ENSG00000011295	TTC19	17	15905229	15948329	ENSG00000169429	IL8	4	74606223	74609433	0.014446	17.71	1.92E-14	1.31E-08	0.02295	3.5821	0.0016267	rs7210990	1.42E-95	0	3.2717e-310	0	Small_Intestine_Terminal_Ileum
ENSG00000139531	SUOX	12	56390964	56400425	ENSG00000105085	MED26	19	16698215	16739873	0.013676	22.342	2.31E-14	1.55E-08	0.0019339	0.59293	0.61973	rs10876864	6.01E-06	0.03846264	3.2717e-310	0	Adipose_Subcutaneous
ENSG00000139531	SUOX	12	56390964	56400425	ENSG00000105085	MED26	19	16698215	16739873	0.013676	22.342	2.31E-14	1.55E-08	0.0019339	0.59293	0.61973	rs10876864	6.01E-06	0.03846264	3.2717e-310	0	Artery_Aorta
ENSG00000139531	SUOX	12	56390964	56400425	ENSG00000105085	MED26	19	16698215	16739873	0.013676	22.342	2.31E-14	1.55E-08	0.0019339	0.59293	0.61973	rs10876864	6.01E-06	0.03846264	3.2717e-310	0	Artery_Tibial
ENSG00000139531	SUOX	12	56390964	56400425	ENSG00000105085	MED26	19	16698215	16739873	0.013676	22.342	2.31E-14	1.55E-08	0.0019339	0.59293	0.61973	rs10876864	6.01E-06	0.03846264	3.2717e-310	0	Brain_Caudate_basal_ganglia
ENSG00000139531	SUOX	12	56390964	56400425	ENSG00000105085	MED26	19	16698215	16739873	0.013676	22.342	2.31E-14	1.55E-08	0.0019339	0.59293	0.61973	rs10876864	6.01E-06	0.03846264	3.2717e-310	0	Brain_Putamen_basal_ganglia
ENSG00000139531	SUOX	12	56390964	56400425	ENSG00000105085	MED26	19	16698215	16739873	0.013676	22.342	2.31E-14	1.55E-08	0.0019339	0.59293	0.61973	rs10876864	6.01E-06	0.03846264	3.2717e-310	0	Breast_Mammary_Tissue
ENSG00000139531	SUOX	12	56390964	56400425	ENSG00000105085	MED26	19	16698215	16739873	0.013676	22.342	2.31E-14	1.55E-08	0.0019339	0.59293	0.61973	rs10876864	6.01E-06	0.03846264	3.2717e-310	0	Colon_Sigmoid
ENSG00000139531	SUOX	12	56390964	56400425	ENSG00000105085	MED26	19	16698215	16739873	0.013676	22.342	2.31E-14	1.55E-08	0.0019339	0.59293	0.61973	rs10876864	6.01E-06	0.03846264	3.2717e-310	0	Esophagus_Gastroesophageal_Junction
ENSG00000139531	SUOX	12	56390964	56400425	ENSG00000105085	MED26	19	16698215	16739873	0.013676	22.342	2.31E-14	1.55E-08	0.0019339	0.59293	0.61973	rs10876864	6.01E-06	0.03846264	3.2717e-310	0	Esophagus_Muscularis
ENSG00000139531	SUOX	12	56390964	56400425	ENSG00000105085	MED26	19	16698215	16739873	0.013676	22.342	2.31E-14	1.55E-08	0.0019339	0.59293	0.61973	rs10876864	6.01E-06	0.03846264	3.2717e-310	0	Heart_Atrial_Appendage
ENSG00000139531	SUOX	12	56390964	56400425	ENSG00000105085	MED26	19	16698215	16739873	0.013676	22.342	2.31E-14	1.55E-08	0.0019339	0.59293	0.61973	rs10876864	6.01E-06	0.03846264	3.2717e-310	0	Nerve_Tibial
ENSG00000139531	SUOX	12	56390964	56400425	ENSG00000105085	MED26	19	16698215	16739873	0.013676	22.342	2.31E-14	1.55E-08	0.0019339	0.59293	0.61973	rs10876864	6.01E-06	0.03846264	3.2717e-310	0	Ovary
ENSG00000139531	SUOX	12	56390964	56400425	ENSG00000105085	MED26	19	16698215	16739873	0.013676	22.342	2.31E-14	1.55E-08	0.0019339	0.59293	0.61973	rs10876864	6.01E-06	0.03846264	3.2717e-310	0	Prostate
ENSG00000139531	SUOX	12	56390964	56400425	ENSG00000105085	MED26	19	16698215	16739873	0.013676	22.342	2.31E-14	1.55E-08	0.0019339	0.59293	0.61973	rs10876864	6.01E-06	0.03846264	3.2717e-310	0	Skin_Not_Sun_Exposed_Suprapubic
ENSG00000139531	SUOX	12	56390964	56400425	ENSG00000105085	MED26	19	16698215	16739873	0.013676	22.342	2.31E-14	1.55E-08	0.0019339	0.59293	0.61973	rs10876864	6.01E-06	0.03846264	3.2717e-310	0	Skin_Sun_Exposed_Lower_leg
ENSG00000139531	SUOX	12	56390964	56400425	ENSG00000105085	MED26	19	16698215	16739873	0.013676	22.342	2.31E-14	1.55E-08	0.0019339	0.59293	0.61973	rs10876864	6.01E-06	0.03846264	3.2717e-310	0	Spleen
ENSG00000139531	SUOX	12	56390964	56400425	ENSG00000105085	MED26	19	16698215	16739873	0.013676	22.342	2.31E-14	1.55E-08	0.0019339	0.59293	0.61973	rs10876864	6.01E-06	0.03846264	3.2717e-310	0	Testis
ENSG00000139531	SUOX	12	56390964	56400425	ENSG00000105085	MED26	19	16698215	16739873	0.013676	22.342	2.31E-14	1.55E-08	0.0019339	0.59293	0.61973	rs10876864	6.01E-06	0.03846264	3.2717e-310	0	Thyroid
ENSG00000139531	SUOX	12	56390964	56400425	ENSG00000105085	MED26	19	16698215	16739873	0.013676	22.342	2.31E-14	1.55E-08	0.0019339	0.59293	0.61973	rs10876864	6.01E-06	0.03846264	3.2717e-310	0	Whole_Blood
ENSG00000174885	NLRP6	11	278365	285359	ENSG00000138722	MMRN1	4	90800683	90875780	0.01619	11.355	2.33E-14	1.56E-08	0.019026	1.9653	0.040297	rs11602954	5.94E-65	0	2.12E-70	0	Nerve_Tibial
ENSG00000174885	NLRP6	11	278365	285359	ENSG00000138722	MMRN1	4	90800683	90875780	0.01619	11.355	2.33E-14	1.56E-08	0.019026	1.9653	0.040297	rs17655730	6.29E-64	0	5.43E-117	0	Artery_Aorta
ENSG00000174885	NLRP6	11	278365	285359	ENSG00000138722	MMRN1	4	90800683	90875780	0.01619	11.355	2.33E-14	1.56E-08	0.019026	1.9653	0.040297	rs17655730	6.29E-64	0	5.43E-117	0	Nerve_Tibial
ENSG00000174885	NLRP6	11	278365	285359	ENSG00000138722	MMRN1	4	90800683	90875780	0.01619	11.355	2.33E-14	1.56E-08	0.019026	1.9653	0.040297	rs17655730	6.29E-64	0	5.43E-117	0	Skin_Sun_Exposed_Lower_leg
ENSG00000174885	NLRP6	11	278365	285359	ENSG00000138722	MMRN1	4	90800683	90875780	0.01619	11.355	2.33E-14	1.56E-08	0.019026	1.9653	0.040297	rs505404	2.01E-74	0	2.65E-88	0	Nerve_Tibial
ENSG00000174885	NLRP6	11	278365	285359	ENSG00000138722	MMRN1	4	90800683	90875780	0.01619	11.355	2.33E-14	1.56E-08	0.019026	1.9653	0.040297	rs505404	2.01E-74	0	2.65E-88	0	Prostate
ENSG00000108733	PEX12	17	33901814	33905882	ENSG00000166169	POLL	10	103338639	103346957	0.014987	14.704	2.45E-14	1.63E-08	0.018163	3.389	0.004859	rs9915021	3.09E-30	0	NA	NA	Brain_Caudate_basal_ganglia
ENSG00000108733	PEX12	17	33901814	33905882	ENSG00000166169	POLL	10	103338639	103346957	0.014987	14.704	2.45E-14	1.63E-08	0.018163	3.389	0.004859	rs9915021	3.09E-30	0	NA	NA	Breast_Mammary_Tissue
ENSG00000108733	PEX12	17	33901814	33905882	ENSG00000166169	POLL	10	103338639	103346957	0.014987	14.704	2.45E-14	1.63E-08	0.018163	3.389	0.004859	rs10512472	1.87E-33	0	1.06E-12	0	Brain_Caudate_basal_ganglia
ENSG00000205045	SLFN12L	17	33800708	33864880	ENSG00000140564	FURIN	15	91411822	91426688	0.01616	11.334	2.50E-14	1.66E-08	0.013682	1.8112	0.081766	rs8082605	2.70E-18	0	2.49E-31	0	Adipose_Subcutaneous
ENSG00000205045	SLFN12L	17	33800708	33864880	ENSG00000140564	FURIN	15	91411822	91426688	0.01616	11.334	2.50E-14	1.66E-08	0.013682	1.8112	0.081766	rs8082605	2.70E-18	0	2.49E-31	0	Adipose_Visceral_Omentum
ENSG00000205045	SLFN12L	17	33800708	33864880	ENSG00000140564	FURIN	15	91411822	91426688	0.01616	11.334	2.50E-14	1.66E-08	0.013682	1.8112	0.081766	rs8082605	2.70E-18	0	2.49E-31	0	Heart_Left_Ventricle
ENSG00000205045	SLFN12L	17	33800708	33864880	ENSG00000140564	FURIN	15	91411822	91426688	0.01616	11.334	2.50E-14	1.66E-08	0.013682	1.8112	0.081766	rs8082605	2.70E-18	0	2.49E-31	0	Nerve_Tibial
ENSG00000205045	SLFN12L	17	33800708	33864880	ENSG00000140564	FURIN	15	91411822	91426688	0.01616	11.334	2.50E-14	1.66E-08	0.013682	1.8112	0.081766	rs10512472	1.96E-14	0	4.96E-30	0	Pancreas
ENSG00000108733	PEX12	17	33901814	33905882	ENSG00000095303	PTGS1	9	125132824	125157982	0.014951	14.668	2.66E-14	1.76E-08	0.053532	10.362	1.11E-09	rs9915021	5.42E-64	0	NA	NA	Brain_Caudate_basal_ganglia
ENSG00000108733	PEX12	17	33901814	33905882	ENSG00000095303	PTGS1	9	125132824	125157982	0.014951	14.668	2.66E-14	1.76E-08	0.053532	10.362	1.11E-09	rs9915021	5.42E-64	0	NA	NA	Breast_Mammary_Tissue
ENSG00000108733	PEX12	17	33901814	33905882	ENSG00000095303	PTGS1	9	125132824	125157982	0.014951	14.668	2.66E-14	1.76E-08	0.053532	10.362	1.11E-09	rs10512472	8.41E-119	0	1.06E-12	0	Brain_Caudate_basal_ganglia
ENSG00000168374	ARF4	3	57557090	57583947	ENSG00000120885	CLU	8	27454434	27472548	0.01495	14.667	2.67E-14	1.76E-08	0.019569	3.6567	0.0027844	rs17825630	4.19E-07	0.00376578	NA	NA	Artery_Tibial
ENSG00000168374	ARF4	3	57557090	57583947	ENSG00000120885	CLU	8	27454434	27472548	0.01495	14.667	2.67E-14	1.76E-08	0.019569	3.6567	0.0027844	rs17825630	4.19E-07	0.00376578	NA	NA	Heart_Atrial_Appendage
ENSG00000006125	AP2B1	17	33913918	34053436	ENSG00000149260	CAPN5	11	76777979	76837201	0.016113	11.3	2.78E-14	1.83E-08	0.0087916	1.3526	0.23104	rs10512472	1.11E-06	0.00889928	3.62E-14	0	Heart_Left_Ventricle
ENSG00000154760	SLFN13	17	33762115	33775856	ENSG00000029534	ANK1	8	41510739	41754280	0.018206	8.1356	2.92E-14	1.91E-08	0.010725	0.82126	0.62861	rs225245	8.61E-22	0	NA	NA	Liver
ENSG00000154760	SLFN13	17	33762115	33775856	ENSG00000029534	ANK1	8	41510739	41754280	0.018206	8.1356	2.92E-14	1.91E-08	0.010725	0.82126	0.62861	rs8082605	4.69E-14	0	2.08E-19	0	Artery_Tibial
ENSG00000154760	SLFN13	17	33762115	33775856	ENSG00000029534	ANK1	8	41510739	41754280	0.018206	8.1356	2.92E-14	1.91E-08	0.010725	0.82126	0.62861	rs8082605	4.69E-14	0	2.08E-19	0	Esophagus_Mucosa
ENSG00000154760	SLFN13	17	33762115	33775856	ENSG00000029534	ANK1	8	41510739	41754280	0.018206	8.1356	2.92E-14	1.91E-08	0.010725	0.82126	0.62861	rs8082605	4.69E-14	0	2.08E-19	0	Nerve_Tibial
ENSG00000154760	SLFN13	17	33762115	33775856	ENSG00000029534	ANK1	8	41510739	41754280	0.018206	8.1356	2.92E-14	1.91E-08	0.010725	0.82126	0.62861	rs8082605	4.69E-14	0	2.08E-19	0	Pituitary
ENSG00000154760	SLFN13	17	33762115	33775856	ENSG00000029534	ANK1	8	41510739	41754280	0.018206	8.1356	2.92E-14	1.91E-08	0.010725	0.82126	0.62861	rs10512472	4.67E-16	0	6.34E-38	0	Uterus
ENSG00000172123	SLFN12	17	33738079	33760302	ENSG00000115649	CNPPD1	2	220036619	220042035	0.019974	6.552	4.01E-14	2.60E-08	NA	NA	NA	rs225245	1.17E-55	0	NA	NA	Cells_EBV-transformed_lymphocytes
ENSG00000172123	SLFN12	17	33738079	33760302	ENSG00000115649	CNPPD1	2	220036619	220042035	0.019974	6.552	4.01E-14	2.60E-08	NA	NA	NA	rs9915021	3.39E-19	0	NA	NA	Breast_Mammary_Tissue
ENSG00000174885	NLRP6	11	278365	285359	ENSG00000259207	ITGB3	17	45387505	45389182	0.015949	11.183	4.07E-14	2.63E-08	0.025639	2.6665	0.004665	rs11602954	1.90E-32	0	2.12E-70	0	Nerve_Tibial
ENSG00000174885	NLRP6	11	278365	285359	ENSG00000259207	ITGB3	17	45387505	45389182	0.015949	11.183	4.07E-14	2.63E-08	0.025639	2.6665	0.004665	rs17655730	6.92E-36	0	5.43E-117	0	Artery_Aorta
ENSG00000174885	NLRP6	11	278365	285359	ENSG00000259207	ITGB3	17	45387505	45389182	0.015949	11.183	4.07E-14	2.63E-08	0.025639	2.6665	0.004665	rs17655730	6.92E-36	0	5.43E-117	0	Nerve_Tibial
ENSG00000174885	NLRP6	11	278365	285359	ENSG00000259207	ITGB3	17	45387505	45389182	0.015949	11.183	4.07E-14	2.63E-08	0.025639	2.6665	0.004665	rs17655730	6.92E-36	0	5.43E-117	0	Skin_Sun_Exposed_Lower_leg
ENSG00000174885	NLRP6	11	278365	285359	ENSG00000259207	ITGB3	17	45387505	45389182	0.015949	11.183	4.07E-14	2.63E-08	0.025639	2.6665	0.004665	rs505404	5.02E-41	0	2.65E-88	0	Nerve_Tibial
ENSG00000174885	NLRP6	11	278365	285359	ENSG00000259207	ITGB3	17	45387505	45389182	0.015949	11.183	4.07E-14	2.63E-08	0.025639	2.6665	0.004665	rs505404	5.02E-41	0	2.65E-88	0	Prostate
ENSG00000108474	PIGL	17	16120523	16250988	ENSG00000169429	IL8	4	74606223	74609433	0.01476	14.478	4.17E-14	2.69E-08	0.02372	5.5699	0.00019702	rs7210990	1.42E-95	0	1.35E-124	0	Testis
ENSG00000205045	SLFN12L	17	33800708	33864880	ENSG00000166169	POLL	10	103338639	103346957	0.01591	11.155	4.44E-14	2.84E-08	0.029136	3.9185	0.00031981	rs8082605	2.02E-34	0	2.49E-31	0	Adipose_Subcutaneous
ENSG00000205045	SLFN12L	17	33800708	33864880	ENSG00000166169	POLL	10	103338639	103346957	0.01591	11.155	4.44E-14	2.84E-08	0.029136	3.9185	0.00031981	rs8082605	2.02E-34	0	2.49E-31	0	Adipose_Visceral_Omentum
ENSG00000205045	SLFN12L	17	33800708	33864880	ENSG00000166169	POLL	10	103338639	103346957	0.01591	11.155	4.44E-14	2.84E-08	0.029136	3.9185	0.00031981	rs8082605	2.02E-34	0	2.49E-31	0	Heart_Left_Ventricle
ENSG00000205045	SLFN12L	17	33800708	33864880	ENSG00000166169	POLL	10	103338639	103346957	0.01591	11.155	4.44E-14	2.84E-08	0.029136	3.9185	0.00031981	rs8082605	2.02E-34	0	2.49E-31	0	Nerve_Tibial
ENSG00000205045	SLFN12L	17	33800708	33864880	ENSG00000166169	POLL	10	103338639	103346957	0.01591	11.155	4.44E-14	2.84E-08	0.029136	3.9185	0.00031981	rs10512472	1.87E-33	0	4.96E-30	0	Pancreas
ENSG00000172123	SLFN12	17	33738079	33760302	ENSG00000022840	RNF10	12	120971283	121015397	0.019841	6.5075	5.33E-14	3.36E-08	0.026038	4.0769	0.00048132	rs225245	1.95E-178	0	NA	NA	Cells_EBV-transformed_lymphocytes
ENSG00000172123	SLFN12	17	33738079	33760302	ENSG00000022840	RNF10	12	120971283	121015397	0.019841	6.5075	5.33E-14	3.36E-08	0.026038	4.0769	0.00048132	rs9915021	1.07E-57	0	NA	NA	Breast_Mammary_Tissue
ENSG00000174885	NLRP6	11	278365	285359	ENSG00000205038	PKHD1L1	8	110374706	110542559	0.015814	11.087	5.54E-14	3.49E-08	0.011579	1.187	0.29959	rs11602954	3.21E-08	0.00042188	2.12E-70	0	Nerve_Tibial
ENSG00000174885	NLRP6	11	278365	285359	ENSG00000205038	PKHD1L1	8	110374706	110542559	0.015814	11.087	5.54E-14	3.49E-08	0.011579	1.187	0.29959	rs17655730	2.44E-13	0	5.43E-117	0	Artery_Aorta
ENSG00000174885	NLRP6	11	278365	285359	ENSG00000205038	PKHD1L1	8	110374706	110542559	0.015814	11.087	5.54E-14	3.49E-08	0.011579	1.187	0.29959	rs17655730	2.44E-13	0	5.43E-117	0	Nerve_Tibial
ENSG00000174885	NLRP6	11	278365	285359	ENSG00000205038	PKHD1L1	8	110374706	110542559	0.015814	11.087	5.54E-14	3.49E-08	0.011579	1.187	0.29959	rs17655730	2.44E-13	0	5.43E-117	0	Skin_Sun_Exposed_Lower_leg
ENSG00000174885	NLRP6	11	278365	285359	ENSG00000205038	PKHD1L1	8	110374706	110542559	0.015814	11.087	5.54E-14	3.49E-08	0.011579	1.187	0.29959	rs505404	1.11E-15	0	2.65E-88	0	Nerve_Tibial
ENSG00000174885	NLRP6	11	278365	285359	ENSG00000205038	PKHD1L1	8	110374706	110542559	0.015814	11.087	5.54E-14	3.49E-08	0.011579	1.187	0.29959	rs505404	1.11E-15	0	2.65E-88	0	Prostate
ENSG00000006125	AP2B1	17	33913918	34053436	ENSG00000101335	MYL9	20	35169887	35178228	0.015805	11.08	5.66E-14	3.55E-08	0.047868	7.6669	4.72E-08	rs10512472	4.66E-133	0	3.62E-14	0	Heart_Left_Ventricle
ENSG00000177963	RIC8A	11	207511	215113	ENSG00000259207	ITGB3	17	45387505	45389182	0.014629	14.348	5.68E-14	3.55E-08	0.019068	2.2184	0.024137	rs11602954	1.90E-32	0	7.55E-62	0	Adipose_Subcutaneous

ENSG00000177963	RIC8A	11	207511	215113	ENSG00000259207	ITGB3	17	45387505	45389182	0.014629	14.348	5.68E-14	3.55E-08	0.019068	2.2184	0.024137	rs11602954	1.90E-32	0	7.55E-62	0	Artery_Aorta
ENSG00000177963	RIC8A	11	207511	215113	ENSG00000259207	ITGB3	17	45387505	45389182	0.014629	14.348	5.68E-14	3.55E-08	0.019068	2.2184	0.024137	rs11602954	1.90E-32	0	7.55E-62	0	Artery_Tibial
ENSG00000177963	RIC8A	11	207511	215113	ENSG00000259207	ITGB3	17	45387505	45389182	0.014629	14.348	5.68E-14	3.55E-08	0.019068	2.2184	0.024137	rs11602954	1.90E-32	0	7.55E-62	0	Brain_Caudate_basal_ganglia
ENSG00000177963	RIC8A	11	207511	215113	ENSG00000259207	ITGB3	17	45387505	45389182	0.014629	14.348	5.68E-14	3.55E-08	0.019068	2.2184	0.024137	rs11602954	1.90E-32	0	7.55E-62	0	Brain_Frontal_Cortex_BA9
ENSG00000177963	RIC8A	11	207511	215113	ENSG00000259207	ITGB3	17	45387505	45389182	0.014629	14.348	5.68E-14	3.55E-08	0.019068	2.2184	0.024137	rs11602954	1.90E-32	0	7.55E-62	0	Colon_Sigmoid
ENSG00000177963	RIC8A	11	207511	215113	ENSG00000259207	ITGB3	17	45387505	45389182	0.014629	14.348	5.68E-14	3.55E-08	0.019068	2.2184	0.024137	rs11602954	1.90E-32	0	7.55E-62	0	Colon_Transverse
ENSG00000177963	RIC8A	11	207511	215113	ENSG00000259207	ITGB3	17	45387505	45389182	0.014629	14.348	5.68E-14	3.55E-08	0.019068	2.2184	0.024137	rs11602954	1.90E-32	0	7.55E-62	0	Esophagus_Gastroesophageal_Junction
ENSG00000177963	RIC8A	11	207511	215113	ENSG00000259207	ITGB3	17	45387505	45389182	0.014629	14.348	5.68E-14	3.55E-08	0.019068	2.2184	0.024137	rs11602954	1.90E-32	0	7.55E-62	0	Esophagus_Mucosa
ENSG00000177963	RIC8A	11	207511	215113	ENSG00000259207	ITGB3	17	45387505	45389182	0.014629	14.348	5.68E-14	3.55E-08	0.019068	2.2184	0.024137	rs11602954	1.90E-32	0	7.55E-62	0	Esophagus_Muscularis
ENSG00000177963	RIC8A	11	207511	215113	ENSG00000259207	ITGB3	17	45387505	45389182	0.014629	14.348	5.68E-14	3.55E-08	0.019068	2.2184	0.024137	rs11602954	1.90E-32	0	7.55E-62	0	Lung
ENSG00000177963	RIC8A	11	207511	215113	ENSG00000259207	ITGB3	17	45387505	45389182	0.014629	14.348	5.68E-14	3.55E-08	0.019068	2.2184	0.024137	rs11602954	1.90E-32	0	7.55E-62	0	Muscle_Skeletal
ENSG00000177963	RIC8A	11	207511	215113	ENSG00000259207	ITGB3	17	45387505	45389182	0.014629	14.348	5.68E-14	3.55E-08	0.019068	2.2184	0.024137	rs11602954	1.90E-32	0	7.55E-62	0	Nerve_Tibial
ENSG00000177963	RIC8A	11	207511	215113	ENSG00000259207	ITGB3	17	45387505	45389182	0.014629	14.348	5.68E-14	3.55E-08	0.019068	2.2184	0.024137	rs11602954	1.90E-32	0	7.55E-62	0	Skin_Not_Sun_Exposed_Suprapubic
ENSG00000177963	RIC8A	11	207511	215113	ENSG00000259207	ITGB3	17	45387505	45389182	0.014629	14.348	5.68E-14	3.55E-08	0.019068	2.2184	0.024137	rs11602954	1.90E-32	0	7.55E-62	0	Skin_Sun_Exposed_Lower_leg
ENSG00000177963	RIC8A	11	207511	215113	ENSG00000259207	ITGB3	17	45387505	45389182	0.014629	14.348	5.68E-14	3.55E-08	0.019068	2.2184	0.024137	rs11602954	1.90E-32	0	7.55E-62	0	Thyroid
ENSG00000177963	RIC8A	11	207511	215113	ENSG00000259207	ITGB3	17	45387505	45389182	0.014629	14.348	5.68E-14	3.55E-08	0.019068	2.2184	0.024137	rs17655730	6.92E-36	0	5.10E-36	0	Adipose_Subcutaneous
ENSG00000177963	RIC8A	11	207511	215113	ENSG00000259207	ITGB3	17	45387505	45389182	0.014629	14.348	5.68E-14	3.55E-08	0.019068	2.2184	0.024137	rs17655730	6.92E-36	0	5.10E-36	0	Esophagus_Mucosa
ENSG00000177963	RIC8A	11	207511	215113	ENSG00000259207	ITGB3	17	45387505	45389182	0.014629	14.348	5.68E-14	3.55E-08	0.019068	2.2184	0.024137	rs17655730	6.92E-36	0	5.10E-36	0	Esophagus_Muscularis
ENSG00000177963	RIC8A	11	207511	215113	ENSG00000259207	ITGB3	17	45387505	45389182	0.014629	14.348	5.68E-14	3.55E-08	0.019068	2.2184	0.024137	rs17655730	6.92E-36	0	5.10E-36	0	Heart_Left_Ventricle
ENSG00000177963	RIC8A	11	207511	215113	ENSG00000259207	ITGB3	17	45387505	45389182	0.014629	14.348	5.68E-14	3.55E-08	0.019068	2.2184	0.024137	rs17655730	6.92E-36	0	5.10E-36	0	Ovary
ENSG00000177963	RIC8A	11	207511	215113	ENSG00000259207	ITGB3	17	45387505	45389182	0.014629	14.348	5.68E-14	3.55E-08	0.019068	2.2184	0.024137	rs17655730	6.92E-36	0	5.10E-36	0	Pancreas
ENSG00000177963	RIC8A	11	207511	215113	ENSG00000259207	ITGB3	17	45387505	45389182	0.014629	14.348	5.68E-14	3.55E-08	0.019068	2.2184	0.024137	rs17655730	6.92E-36	0	5.10E-36	0	Skin_Not_Sun_Exposed_Suprapubic
ENSG00000177963	RIC8A	11	207511	215113	ENSG00000259207	ITGB3	17	45387505	45389182	0.014629	14.348	5.68E-14	3.55E-08	0.019068	2.2184	0.024137	rs505404	5.02E-41	0	3.66E-47	0	Artery_Aorta
ENSG00000177963	RIC8A	11	207511	215113	ENSG00000259207	ITGB3	17	45387505	45389182	0.014629	14.348	5.68E-14	3.55E-08	0.019068	2.2184	0.024137	rs505404	5.02E-41	0	3.66E-47	0	Thyroid
ENSG00000177963	RIC8A	11	207511	215113	ENSG00000205038	PKHD1L1	8	110374706	110542559	0.014564	14.282	6.62E-14	4.11E-08	0.0082959	0.95469	0.47035	rs11602954	3.21E-08	0.00042188	7.55E-62	0	Adipose_Subcutaneous
ENSG00000177963	RIC8A	11	207511	215113	ENSG00000205038	PKHD1L1	8	110374706	110542559	0.014564	14.282	6.62E-14	4.11E-08	0.0082959	0.95469	0.47035	rs11602954	3.21E-08	0.00042188	7.55E-62	0	Artery_Aorta
ENSG00000177963	RIC8A	11	207511	215113	ENSG00000205038	PKHD1L1	8	110374706	110542559	0.014564	14.282	6.62E-14	4.11E-08	0.0082959	0.95469	0.47035	rs11602954	3.21E-08	0.00042188	7.55E-62	0	Artery_Tibial
ENSG00000177963	RIC8A	11	207511	215113	ENSG00000205038	PKHD1L1	8	110374706	110542559	0.014564	14.282	6.62E-14	4.11E-08	0.0082959	0.95469	0.47035	rs11602954	3.21E-08	0.00042188	7.55E-62	0	Brain_Caudate_basal_ganglia
ENSG00000177963	RIC8A	11	207511	215113	ENSG00000205038	PKHD1L1	8	110374706	110542559	0.014564	14.282	6.62E-14	4.11E-08	0.0082959	0.95469	0.47035	rs11602954	3.21E-08	0.00042188	7.55E-62	0	Brain_Frontal_Cortex_BA9
ENSG00000177963	RIC8A	11	207511	215113	ENSG00000205038	PKHD1L1	8	110374706	110542559	0.014564	14.282	6.62E-14	4.11E-08	0.0082959	0.95469	0.47035	rs11602954	3.21E-08	0.00042188	7.55E-62	0	Colon_Sigmoid
ENSG00000177963	RIC8A	11	207511	215113	ENSG00000205038	PKHD1L1	8	110374706	110542559	0.014564	14.282	6.62E-14	4.11E-08	0.0082959	0.95469	0.47035	rs11602954	3.21E-08	0.00042188	7.55E-62	0	Colon_Transverse
ENSG00000177963	RIC8A	11	207511	215113	ENSG00000205038	PKHD1L1	8	110374706	110542559	0.014564	14.282	6.62E-14	4.11E-08	0.0082959	0.95469	0.47035	rs11602954	3.21E-08	0.00042188	7.55E-62	0	Esophagus_Gastroesophageal_Junction
ENSG00000177963	RIC8A	11	207511	215113	ENSG00000205038	PKHD1L1	8	110374706	110542559	0.014564	14.282	6.62E-14	4.11E-08	0.0082959	0.95469	0.47035	rs11602954	3.21E-08	0.00042188	7.55E-62	0	Esophagus_Mucosa
ENSG00000177963	RIC8A	11	207511	215113	ENSG00000205038	PKHD1L1	8	110374706	110542559	0.014564	14.282	6.62E-14	4.11E-08	0.0082959	0.95469	0.47035	rs11602954	3.21E-08	0.00042188	7.55E-62	0	Esophagus_Muscularis
ENSG00000177963	RIC8A	11	207511	215113	ENSG00000205038	PKHD1L1	8	110374706	110542559	0.014564	14.282	6.62E-14	4.11E-08	0.0082959	0.95469	0.47035	rs11602954	3.21E-08	0.00042188	7.55E-62	0	Lung
ENSG00000177963	RIC8A	11	207511	215113	ENSG00000205038	PKHD1L1	8	110374706	110542559	0.014564	14.282	6.62E-14	4.11E-08	0.0082959	0.95469	0.47035	rs11602954	3.21E-08	0.00042188	7.55E-62	0	Muscle_Skeletal
ENSG00000177963	RIC8A	11	207511	215113	ENSG00000205038	PKHD1L1	8	110374706	110542559	0.014564	14.282	6.62E-14	4.11E-08	0.0082959	0.95469	0.47035	rs11602954	3.21E-08	0.00042188	7.55E-62	0	Nerve_Tibial
ENSG00000177963	RIC8A	11	207511	215113	ENSG00000205038	PKHD1L1	8	110374706	110542559	0.014564	14.282	6.62E-14	4.11E-08	0.0082959	0.95469	0.47035	rs11602954	3.21E-08	0.00042188	7.55E-62	0	Skin_Not_Sun_Exposed_Suprapubic
ENSG00000177963	RIC8A	11	207511	215113	ENSG00000205038	PKHD1L1	8	110374706	110542559	0.014564	14.282	6.62E-14	4.11E-08	0.0082959	0.95469	0.47035	rs11602954	3.21E-08	0.00042188	7.55E-62	0	Skin_Sun_Exposed_Lower_leg
ENSG00000177963	RIC8A	11	207511	215113	ENSG00000205038	PKHD1L1	8	110374706	110542559	0.014564	14.282	6.62E-14	4.11E-08	0.0082959	0.95469	0.47035	rs11602954	3.21E-08	0.00042188	7.55E-62	0	Thyroid
ENSG00000177963	RIC8A	11	207511	215113	ENSG00000205038	PKHD1L1	8	110374706	110542559	0.014564	14.282	6.62E-14	4.11E-08	0.0082959	0.95469	0.47035	rs17655730	2.44E-13	0	5.10E-36	0	Adipose_Subcutaneous
ENSG00000177963	RIC8A	11	207511	215113	ENSG00000205038	PKHD1L1	8	110374706	110542559	0.014564	14.282	6.62E-14	4.11E-08	0.0082959	0.95469	0.47035	rs17655730	2.44E-13	0	5.10E-36	0	Esophagus_Mucosa
ENSG00000177963	RIC8A	11	207511	215113	ENSG00000205038	PKHD1L1	8	110374706	110542559	0.014564	14.282	6.62E-14	4.11E-08	0.0082959	0.95469	0.47035	rs17655730	2.44E-13	0	5.10E-36	0	Esophagus_Muscularis
ENSG00000177963	RIC8A	11	207511	215113	ENSG00000205038	PKHD1L1	8	110374706	110542559	0.014564	14.282	6.62E-14	4.11E-08	0.0082959	0.95469	0.47035	rs17655730	2.44E-13	0	5.10E-36	0	Heart_Left_Ventricle
ENSG00000177963	RIC8A	11	207511	215113	ENSG00000205038	PKHD1L1	8	110374706	110542559	0.014564	14.282	6.62E-14	4.11E-08	0.0082959	0.95469	0.47035	rs17655730	2.44E-13	0	5.10E-36	0	Ovary
ENSG00000177963	RIC8A	11	207511	215113	ENSG00000205038	PKHD1L1	8	110374706	110542559	0.014564	14.282	6.62E-14	4.11E-08	0.0082959	0.95469	0.47035	rs17655730	2.44E-13	0	5.10E-36	0	Pancreas
ENSG00000177963	RIC8A	11	207511	215113	ENSG00000205038	PKHD1L1	8	110374706	110542559	0.014564	14.282	6.62E-14	4.11E-08	0.0082959	0.95469	0.47035	rs17655730	2.44E-13	0	5.10E-36	0	Skin_Not_Sun_Exposed_Suprapubic
ENSG00000177963	RIC8A	11	207511	215113	ENSG00000205038	PKHD1L1	8	110374706	110542559	0.014564	14.282	6.62E-14	4.11E-08	0.0082959	0.95469	0.47035	rs505404	1.11E-15	0	3.66E-47	0	Artery_Aorta
ENSG00000177963	RIC8A	11	207511	215113	ENSG00000205038	PKHD1L1	8	110374706	110542559	0.014564	14.282	6.62E-14	4.11E-08	0.0082959	0.95469	0.47035	rs505404	1.11E-15	0	3.66E-47	0	Thyroid
ENSG00000168374	ARF4	3	57557090	57583947	ENSG00000137801	THBS1	15	39873280	39891667	0.014485	14.204	7.96E-14	4.87E-08	0.023273	4.3653	0.00062212	rs17825630	4.86E-09	0.00012136	NA	NA	Artery_Tibial
ENSG00000168374	ARF4	3	57557090	57583947	ENSG00000137801	THBS1	15	39873280	39891667	0.014485	14.204	7.96E-14	4.87E-08	0.023273	4.3653	0.00062212	rs17825630	4.86E-09	0.00012136	NA	NA	Heart_Atrial_Appendage
ENSG00000163946	FAM208A	3	56658507	56717265	ENSG00000174175	SELP	1	169558087	169599431	0.0162	9.94	8.06E-14	4.92E-08	0.035909	4.8634	2.12E-05	rs12485738	4.31E-202	0	NA	NA	Esophagus_Mucosa
ENSG00000163946	FAM208A	3	56658507	56717265	ENSG00000174175	SELP	1	169558087	169599431	0.0162	9.94	8.06E-14	4.92E-08	0.035909	4.8634	2.12E-05	rs1354034	3.27170000000001e-	0	NA	NA	Heart_Atrial_Appendage
ENSG00000108733	PEX12	17	33901814	33905882	ENSG00000138867	GUCD1	22	24936406	24951284	0.014459	14.178	8.47E-14	5.15E-08	NA	NA	NA	rs9915021	1.78E-10	0	NA	NA	Brain_Caudate_basal_ganglia
ENSG00000108733	PEX12	17	33901814	33905882	ENSG00000138867	GUCD1	22	24936406	24951284	0.014459	14.178	8.47E-14	5.15E-08	NA	NA	NA	rs9915021	1.78E-10	0	NA	NA	Breast_Mammary_Tissue
ENSG00000108733	PEX12	17	33901814	33905882	ENSG00000138867	GUCD1	22	24936406	24951284	0.014459	14.178	8.47E-14	5.15E-08	NA	NA	NA	rs10512472	1.08E-10	0	1.06E-12	0	Brain_Caudate_basal_ganglia
ENSG00000168374	ARF4	3	57557090	57583947	ENSG00000005961	ITGA2B	17	42449548	42466873	0.014433	14.152	9.01E-14	5.43E-08	0.030245	5.7138	3.36E-05	rs17825630	2.36E-32	0	NA	NA	Artery_Tibial
ENSG00000168374	ARF4	3	57557090	57583947	ENSG00000005961	ITGA2B	17	42449548	42466873	0.014433	14.152	9.01E-14	5.43E-08	0.030245	5.7138	3.36E-05	rs17825630	2.36E-32	0	NA	NA	Heart_Atrial_Appendage
ENSG00000006125	AP2B1	17	33913918	34053436	ENSG00000130300	PLVAP	19	17462257	17488159	0.015591	10.928	9.25E-14	5.56E-08	0.017937	2.7853	0.010882	rs10512472	9.07E-18	0	3.62E-14	0	Heart_Left_Ventricle
ENSG00000163946	FAM208A	3	56658507	56717265	ENSG00000049323	LTBP1	2	33172039	33624576	0.016121	9.8903	9.66E-14	5.79E-08	0.027958	3.7556	0.00050601	rs12485738	1.13E-71	0	NA	NA	Esophagus_Mucosa
ENSG00000163946	FAM208A	3	56658507	56717265	ENSG00000049323	LTBP1	2	33172039	33624576	0.016121	9.8903	9.66E-14	5.79E-08	0.027958	3.7556	0.00050601	rs1354034	1.91E-136	0	NA	NA	Heart_Atrial_Appendage
ENSG00000168374	ARF4	3	57557090	57583947	ENSG00000161911	TREML1	6	41117080	41122075	0.014402	14.121	9.69E-14	5.79E-08	0.020584	3.8502	0.0018553	rs17825630	1.16E-19	0	NA	NA	Artery_Tibial
ENSG00000168374	ARF4	3	57557090	57583947	ENSG00000161911	TREML1	6	41117080	41122075	0.014402	14.121	9.69E-14	5.79E-08	0.020584	3.8502	0.0018553	rs17825630	1.16E-19	0	NA	NA	Heart_Atrial_Appendage
ENSG00000168374	ARF4	3	57557090	57583947	ENSG00000085733	CTTN	11	70244510	70282690	0.014373	14.093	1.04E-13	6.16E-08	0.019969	3.7328	0.0023743	rs17825630	1.56E-19	0	NA	NA	Artery_Tibial
ENSG00000168374	ARF4	3	57557090	57583947	ENSG00000085733	CTTN	11	70244510	70282690	0.014373	14.093	1.04E-13	6.16E-08	0.019969	3.7328	0.0023743	rs17825630	1.56E-19	0	NA	NA	Heart_Atrial_Appendage
ENSG00000168374	ARF4	3	57557090	57583947	ENSG00000188677	PARVB	22	44395091	44565106	0.014356	14.076	1.08E-13	6.37E-08	0.034938	6.6324	4.46E-06	rs17825630	9.99E-28	0	NA	NA	Artery_Tibial
ENSG00000168374	ARF4	3	57557090	57583947	ENSG00000188677	PARVB	22	44395091	44565106	0.014356	14.076	1.08E-13	6.37E-08	0.034938	6.6324	4.46E-06	rs17825630	9.99E-28	0	NA	NA	Heart_Atrial_Appendage
ENSG00000177951	BET1L	11	167784	207428	ENSG00000049323	LTBP1	2	33172039	33624576	0.016058	9.8513	1.11E-13	6.54E-08	0.021628	1.8288	0.045403	rs11602954	4.93E-09	0.00012117	9.12E-206	0	Adipose_Visceral_Omentum
ENSG00000177951	BET1L	11	167784	207428	ENSG00000049323	LTBP1	2	33172039	33624576	0.016058	9.8513	1.11E-13	6.54E-08	0.021628	1.8288	0.045403	rs11602954	4.93E-09	0.00012117	9.12E-206	0	Artery_Aorta
ENSG00000177951	BET1L	11	167784	207428	ENSG00000049323	LTBP1	2	33172039	33624576	0.016058	9.8513	1.11E-13	6.54E-08	0.021628	1.8288	0.045403	rs11602954	4.93E-09	0.00012117	9.12E-206	0	Cells_Transformed_fibroblasts
ENSG00000177951	BET1L	11	167784	207428	ENSG00000049323	LTBP1	2	33172039	33624576	0.016058	9.8513	1.11E-13	6.54E-08	0.021628	1.8288	0.045403	rs11602954	4.93E-09	0.00012117	9.12E-206	0	Pituitary
ENSG00000177951	BET1L	11	167784	207428	ENSG00000049323	LTBP1	2	33172039	33624576	0.016058	9.8513	1.11E-13	6.54E-08	0.021628	1.8288	0.045403	rs11602954	4.93E-09	0.00012117	9.12E-206	0	Prostate
ENSG00000177951	BET1L	11	167784	207428	ENSG00000049323	LTBP1	2	33172039	33624576	0.016058	9.8513	1.11E-13	6.54E-08	0.021628	1.8288	0.045403	rs11602954	4.93E-09	0.00012117	9.12E-206	0	Small_Intestine_Terminal_Ileum
ENSG00000177951	BET1L	11	167784	207428	ENSG00000049323	LTBP1	2	33172039	33624576	0.016058	9.8513	1.11E-13	6.54E-08	0.021628	1.8288	0.045403	rs11602954	4.93E-09	0.00012117	9.12E-206	0	Whole_Blood
ENSG00000177951	BET1L	11	167784	207428	ENSG00000049323	LTBP1	2	33172039	33624576	0.016058	9.8513	1.11E-13	6.54E-08	0.021628	1.8288	0.045403	rs17655730	2.15E-13	0	2.96E-166	0	Prostate
ENSG00000177951	BET1L	11	167784	207428	ENSG00000049323	LTBP1	2	33172039	33624576	0.016058	9.8513	1.11E-13	6.54E-08	0.021628	1.8288	0.045403	rs505404	1.77E-15	0	3.73E-187	0	Small_Intestine_Terminal_Ileum
ENSG00000177951	BET1L	11	167784	207428	ENSG00000049323	LTBP1	2	33172039	33624576	0.016058	9.8513	1.11E-13	6.54E-08	0.021628	1.8288	0.045403	rs505404	1.77E-15	0	3.73E-187	0	Whole_Blood
ENSG00000205045	SLFN12L	17	33800708	33864880	ENSG00000101335	MYL9	20	35169887	35178228	0.015465	10.838	1.24E-13	7.22E-08	0.029251	3.9344	0.00030573	rs8082605	3.70E-82	0	2.49E-31	0	Adipose_Subcutaneous
ENSG00000205045	SLFN12L	17	33800708	33864880	ENSG00000101335	MYL9	20	35169887	35178228	0.015465	10.838	1.24E-13	7.22E-08	0.029251	3.9344	0.00030573	rs8082605	3.70E-82	0	2.49E-31	0	Adipose_Visceral_Omentum
ENSG00000205045	SLFN12L	17	33800708	33864880	ENSG00000101335	MYL9	20	35169887	35178228	0.015465	10.838	1.24E-13	7.22E-08	0.029251	3.9344	0.00030573	rs8082605	3.70E-82	0	2.49E-31	0	Heart_Left_Ventricle
ENSG00000205045	SLFN12L	17	33800708	33864880	ENSG00000101335	MYL9	20	35169887	35178228	0.015465	10.838	1.24E-13	7.22E-08	0.029251	3.9344	0.00030573	rs8082605	3.70E-82	0	2.49E-31	0	Nerve_Tibial
ENSG00000205045	SLFN12L	17	33800708	33864880	ENSG00000101335	MYL9	20	35169887	35178228	0.015465	10.838	1.24E-13	7.22E-08	0.029251	3.9344	0.00030573	rs10512472	4.66E-133	0	4.96E-30	0	Pancreas

ENSG00000006125	AP2B1	17	33913918	34053436	ENSG00000166169	POLL	10	103338639	103346957	0.015434	10.816	1.33E-13	7.73E-08	0.031626	4.9805	4.96E-05	rs10512472	1.87E-33	0	3.62E-14	0	Heart_Left_Ventricle
ENSG00000163946	FAM208A	3	56658507	56717265	ENSG00000122786	CALD1	7	134429003	134655479	0.015968	9.795	1.37E-13	7.93E-08	0.027496	3.6916	0.00060533	rs12485738	6.90E-301	0	NA	NA	Esophagus_Mucosa
ENSG00000163946	FAM208A	3	56658507	56717265	ENSG00000122786	CALD1	7	134429003	134655479	0.015968	9.795	1.37E-13	7.93E-08	0.027496	3.6916	0.00060533	rs1354034	3.27170000000001e-	0	NA	NA	Heart_Atrial_Appendage
ENSG00000172660	TAF15	17	34136459	34174246	ENSG00000100614	PPM1A	14	60712470	60765805	0.014771	12.071	1.65E-13	9.53E-08	0.015191	2.3524	0.029223	rs9915021	6.10E-09	0.00013559	NA	NA	Skin_Not_Sun_Exposed_Suprapubic
ENSG00000142082	SIRT3	11	215458	236431	ENSG00000205038	PKHD1L1	8	110374706	110542559	0.012853	20.981	1.68E-13	9.68E-08	0.0021415	0.49199	0.74165	rs11602954	3.21E-08	0.00042188	1.39E-80	0	Adipose_Subcutaneous
ENSG00000142082	SIRT3	11	215458	236431	ENSG00000205038	PKHD1L1	8	110374706	110542559	0.012853	20.981	1.68E-13	9.68E-08	0.0021415	0.49199	0.74165	rs11602954	3.21E-08	0.00042188	1.39E-80	0	Artery_Tibial
ENSG00000142082	SIRT3	11	215458	236431	ENSG00000205038	PKHD1L1	8	110374706	110542559	0.012853	20.981	1.68E-13	9.68E-08	0.0021415	0.49199	0.74165	rs11602954	3.21E-08	0.00042188	1.39E-80	0	Breast_Mammary_Tissue
ENSG00000142082	SIRT3	11	215458	236431	ENSG00000205038	PKHD1L1	8	110374706	110542559	0.012853	20.981	1.68E-13	9.68E-08	0.0021415	0.49199	0.74165	rs11602954	3.21E-08	0.00042188	1.39E-80	0	Cells_Transformed_fibroblasts
ENSG00000142082	SIRT3	11	215458	236431	ENSG00000205038	PKHD1L1	8	110374706	110542559	0.012853	20.981	1.68E-13	9.68E-08	0.0021415	0.49199	0.74165	rs11602954	3.21E-08	0.00042188	1.39E-80	0	Nerve_Tibial
ENSG00000142082	SIRT3	11	215458	236431	ENSG00000205038	PKHD1L1	8	110374706	110542559	0.012853	20.981	1.68E-13	9.68E-08	0.0021415	0.49199	0.74165	rs505404	1.11E-15	0	1.05E-94	0	Adrenal_Gland
ENSG00000142082	SIRT3	11	215458	236431	ENSG00000205038	PKHD1L1	8	110374706	110542559	0.012853	20.981	1.68E-13	9.68E-08	0.0021415	0.49199	0.74165	rs505404	1.11E-15	0	1.05E-94	0	Artery_Aorta
ENSG00000142082	SIRT3	11	215458	236431	ENSG00000205038	PKHD1L1	8	110374706	110542559	0.012853	20.981	1.68E-13	9.68E-08	0.0021415	0.49199	0.74165	rs505404	1.11E-15	0	1.05E-94	0	Lung
ENSG00000142082	SIRT3	11	215458	236431	ENSG00000205038	PKHD1L1	8	110374706	110542559	0.012853	20.981	1.68E-13	9.68E-08	0.0021415	0.49199	0.74165	rs505404	1.11E-15	0	1.05E-94	0	Thyroid
ENSG00000163946	FAM208A	3	56658507	56717265	ENSG00000198478	SH3BGRL2	6	80341000	80413372	0.015843	9.7169	1.82E-13	1.04E-07	0.037645	5.1077	1.04E-05	rs12485738	3.27170000000001e-	0	NA	NA	Esophagus_Mucosa
ENSG00000163946	FAM208A	3	56658507	56717265	ENSG00000198478	SH3BGRL2	6	80341000	80413372	0.015843	9.7169	1.82E-13	1.04E-07	0.037645	5.1077	1.04E-05	rs1354034	3.27170000000001e-	0	NA	NA	Heart_Atrial_Appendage
ENSG00000177951	BET1L	11	167784	207428	ENSG00000122786	CALD1	7	134429003	134655479	0.015829	9.7082	1.87E-13	1.07E-07	0.022356	1.8918	0.036883	rs11602954	3.38E-37	0	9.12E-206	0	Adipose_Visceral_Omentum
ENSG00000177951	BET1L	11	167784	207428	ENSG00000122786	CALD1	7	134429003	134655479	0.015829	9.7082	1.87E-13	1.07E-07	0.022356	1.8918	0.036883	rs11602954	3.38E-37	0	9.12E-206	0	Artery_Aorta
ENSG00000177951	BET1L	11	167784	207428	ENSG00000122786	CALD1	7	134429003	134655479	0.015829	9.7082	1.87E-13	1.07E-07	0.022356	1.8918	0.036883	rs11602954	3.38E-37	0	9.12E-206	0	Cells_Transformed_fibroblasts
ENSG00000177951	BET1L	11	167784	207428	ENSG00000122786	CALD1	7	134429003	134655479	0.015829	9.7082	1.87E-13	1.07E-07	0.022356	1.8918	0.036883	rs11602954	3.38E-37	0	9.12E-206	0	Pituitary
ENSG00000177951	BET1L	11	167784	207428	ENSG00000122786	CALD1	7	134429003	134655479	0.015829	9.7082	1.87E-13	1.07E-07	0.022356	1.8918	0.036883	rs11602954	3.38E-37	0	9.12E-206	0	Prostate
ENSG00000177951	BET1L	11	167784	207428	ENSG00000122786	CALD1	7	134429003	134655479	0.015829	9.7082	1.87E-13	1.07E-07	0.022356	1.8918	0.036883	rs11602954	3.38E-37	0	9.12E-206	0	Small_Intestine_Terminal_Ileum
ENSG00000177951	BET1L	11	167784	207428	ENSG00000122786	CALD1	7	134429003	134655479	0.015829	9.7082	1.87E-13	1.07E-07	0.022356	1.8918	0.036883	rs11602954	3.38E-37	0	9.12E-206	0	Whole_Blood
ENSG00000177951	BET1L	11	167784	207428	ENSG00000122786	CALD1	7	134429003	134655479	0.015829	9.7082	1.87E-13	1.07E-07	0.022356	1.8918	0.036883	rs17655730	3.56E-40	0	2.96E-166	0	Prostate
ENSG00000177951	BET1L	11	167784	207428	ENSG00000122786	CALD1	7	134429003	134655479	0.015829	9.7082	1.87E-13	1.07E-07	0.022356	1.8918	0.036883	rs505404	1.84E-40	0	3.73E-187	0	Small_Intestine_Terminal_Ileum
ENSG00000177951	BET1L	11	167784	207428	ENSG00000122786	CALD1	7	134429003	134655479	0.015829	9.7082	1.87E-13	1.07E-07	0.022356	1.8918	0.036883	rs505404	1.84E-40	0	3.73E-187	0	Whole_Blood
ENSG00000205045	SLFN12L	17	33800708	33864880	ENSG00000158828	PINK1	1	20959948	20978004	0.015267	10.697	1.95E-13	1.11E-07	0.0061024	0.8017	0.58595	rs8082605	2.28E-16	0	2.49E-31	0	Adipose_Subcutaneous
ENSG00000205045	SLFN12L	17	33800708	33864880	ENSG00000158828	PINK1	1	20959948	20978004	0.015267	10.697	1.95E-13	1.11E-07	0.0061024	0.8017	0.58595	rs8082605	2.28E-16	0	2.49E-31	0	Adipose_Visceral_Omentum
ENSG00000205045	SLFN12L	17	33800708	33864880	ENSG00000158828	PINK1	1	20959948	20978004	0.015267	10.697	1.95E-13	1.11E-07	0.0061024	0.8017	0.58595	rs8082605	2.28E-16	0	2.49E-31	0	Heart_Left_Ventricle
ENSG00000205045	SLFN12L	17	33800708	33864880	ENSG00000158828	PINK1	1	20959948	20978004	0.015267	10.697	1.95E-13	1.11E-07	0.0061024	0.8017	0.58595	rs8082605	2.28E-16	0	2.49E-31	0	Nerve_Tibial
ENSG00000205045	SLFN12L	17	33800708	33864880	ENSG00000158828	PINK1	1	20959948	20978004	0.015267	10.697	1.95E-13	1.11E-07	0.0061024	0.8017	0.58595	rs10512472	1.16E-16	0	4.96E-30	0	Pancreas
ENSG00000142082	SIRT3	11	215458	236431	ENSG00000138722	MMRN1	4	90800683	90875780	0.012625	20.604	2.92E-13	1.61E-07	0.0051068	1.1767	0.31949	rs11602954	5.94E-65	0	1.39E-80	0	Adipose_Subcutaneous
ENSG00000142082	SIRT3	11	215458	236431	ENSG00000138722	MMRN1	4	90800683	90875780	0.012625	20.604	2.92E-13	1.61E-07	0.0051068	1.1767	0.31949	rs11602954	5.94E-65	0	1.39E-80	0	Artery_Tibial
ENSG00000142082	SIRT3	11	215458	236431	ENSG00000138722	MMRN1	4	90800683	90875780	0.012625	20.604	2.92E-13	1.61E-07	0.0051068	1.1767	0.31949	rs11602954	5.94E-65	0	1.39E-80	0	Breast_Mammary_Tissue
ENSG00000142082	SIRT3	11	215458	236431	ENSG00000138722	MMRN1	4	90800683	90875780	0.012625	20.604	2.92E-13	1.61E-07	0.0051068	1.1767	0.31949	rs11602954	5.94E-65	0	1.39E-80	0	Cells_Transformed_fibroblasts
ENSG00000142082	SIRT3	11	215458	236431	ENSG00000138722	MMRN1	4	90800683	90875780	0.012625	20.604	2.92E-13	1.61E-07	0.0051068	1.1767	0.31949	rs11602954	5.94E-65	0	1.39E-80	0	Nerve_Tibial
ENSG00000142082	SIRT3	11	215458	236431	ENSG00000138722	MMRN1	4	90800683	90875780	0.012625	20.604	2.92E-13	1.61E-07	0.0051068	1.1767	0.31949	rs7104764	2.04E-08	0.00024398	1.64E-91	0	Brain_Caudate_basal_ganglia
ENSG00000142082	SIRT3	11	215458	236431	ENSG00000138722	MMRN1	4	90800683	90875780	0.012625	20.604	2.92E-13	1.61E-07	0.0051068	1.1767	0.31949	rs7104764	2.04E-08	0.00024398	1.64E-91	0	Colon_Transverse
ENSG00000142082	SIRT3	11	215458	236431	ENSG00000138722	MMRN1	4	90800683	90875780	0.012625	20.604	2.92E-13	1.61E-07	0.0051068	1.1767	0.31949	rs7104764	2.04E-08	0.00024398	1.64E-91	0	Nerve_Tibial
ENSG00000142082	SIRT3	11	215458	236431	ENSG00000138722	MMRN1	4	90800683	90875780	0.012625	20.604	2.92E-13	1.61E-07	0.0051068	1.1767	0.31949	rs7104764	2.04E-08	0.00024398	1.64E-91	0	Spleen
ENSG00000142082	SIRT3	11	215458	236431	ENSG00000138722	MMRN1	4	90800683	90875780	0.012625	20.604	2.92E-13	1.61E-07	0.0051068	1.1767	0.31949	rs505404	2.01E-74	0	1.05E-94	0	Adrenal_Gland
ENSG00000142082	SIRT3	11	215458	236431	ENSG00000138722	MMRN1	4	90800683	90875780	0.012625	20.604	2.92E-13	1.61E-07	0.0051068	1.1767	0.31949	rs505404	2.01E-74	0	1.05E-94	0	Artery_Aorta
ENSG00000142082	SIRT3	11	215458	236431	ENSG00000138722	MMRN1	4	90800683	90875780	0.012625	20.604	2.92E-13	1.61E-07	0.0051068	1.1767	0.31949	rs505404	2.01E-74	0	1.05E-94	0	Lung
ENSG00000142082	SIRT3	11	215458	236431	ENSG00000138722	MMRN1	4	90800683	90875780	0.012625	20.604	2.92E-13	1.61E-07	0.0051068	1.1767	0.31949	rs505404	2.01E-74	0	1.05E-94	0	Thyroid
ENSG00000142082	SIRT3	11	215458	236431	ENSG00000138722	MMRN1	4	90800683	90875780	0.012625	20.604	2.92E-13	1.61E-07	0.0051068	1.1767	0.31949	rs3782123	3.86E-09	0.00010601	NA	NA	Brain_Frontal_Cortex_BA9
ENSG00000132139	GAS2L2	17	34071530	34079897	ENSG00000115649	CNPPD1	2	220036619	220042035	0.015584	9.5555	3.27E-13	1.81E-07	NA	NA	NA	rs9915021	3.39E-19	0	NA	NA	Small_Intestine_Terminal_Ileum
ENSG00000099849	RASSF7	11	560404	564021	ENSG00000138722	MMRN1	4	90800683	90875780	0.015039	10.535	3.28E-13	1.81E-07	0.0086378	1.1377	0.33694	rs505404	2.01E-74	0	NA	NA	Brain_Frontal_Cortex_BA9
ENSG00000099849	RASSF7	11	560404	564021	ENSG00000138722	MMRN1	4	90800683	90875780	0.015039	10.535	3.28E-13	1.81E-07	0.0086378	1.1377	0.33694	rs3782123	3.86E-09	0.00010601	NA	NA	Whole_Blood
ENSG00000154768	C17orf50	17	34087916	34092098	ENSG00000022840	RNF10	12	120971283	121015397	0.014431	11.79	3.63E-13	1.98E-07	0.01572	2.0854	0.042643	rs9915021	1.07E-57	0	NA	NA	Pituitary
ENSG00000168374	ARF4	3	57557090	57583947	ENSG00000124491	F13A1	6	6144318	6321246	0.013779	13.502	4.18E-13	2.27E-07	0.011502	2.1317	0.059587	rs17825630	8.88E-16	0	NA	NA	Artery_Tibial
ENSG00000168374	ARF4	3	57557090	57583947	ENSG00000124491	F13A1	6	6144318	6321246	0.013779	13.502	4.18E-13	2.27E-07	0.011502	2.1317	0.059587	rs17825630	8.88E-16	0	NA	NA	Heart_Atrial_Appendage
ENSG00000205045	SLFN12L	17	33800708	33864880	ENSG00000196961	AP2A1	19	50270225	50309510	0.01486	10.408	4.94E-13	2.66E-07	0.0043149	0.56584	0.78401	rs8082605	6.57E-08	0.00085447	2.49E-31	0	Adipose_Subcutaneous
ENSG00000205045	SLFN12L	17	33800708	33864880	ENSG00000196961	AP2A1	19	50270225	50309510	0.01486	10.408	4.94E-13	2.66E-07	0.0043149	0.56584	0.78401	rs8082605	6.57E-08	0.00085447	2.49E-31	0	Adipose_Visceral_Omentum
ENSG00000205045	SLFN12L	17	33800708	33864880	ENSG00000196961	AP2A1	19	50270225	50309510	0.01486	10.408	4.94E-13	2.66E-07	0.0043149	0.56584	0.78401	rs8082605	6.57E-08	0.00085447	2.49E-31	0	Heart_Left_Ventricle
ENSG00000205045	SLFN12L	17	33800708	33864880	ENSG00000196961	AP2A1	19	50270225	50309510	0.01486	10.408	4.94E-13	2.66E-07	0.0043149	0.56584	0.78401	rs8082605	6.57E-08	0.00085447	2.49E-31	0	Nerve_Tibial
ENSG00000205045	SLFN12L	17	33800708	33864880	ENSG00000196961	AP2A1	19	50270225	50309510	0.01486	10.408	4.94E-13	2.66E-07	0.0043149	0.56584	0.78401	rs10512472	3.81E-08	0.00049957	4.96E-30	0	Pancreas
ENSG00000108733	PEX12	17	33901814	33905882	ENSG00000198959	TGM2	20	36756863	36794980	0.013634	13.358	5.87E-13	3.14E-07	0.0044937	0.82695	0.53054	rs9915021	2.01E-09	5.61E-05	NA	NA	Brain_Caudate_basal_ganglia
ENSG00000108733	PEX12	17	33901814	33905882	ENSG00000198959	TGM2	20	36756863	36794980	0.013634	13.358	5.87E-13	3.14E-07	0.0044937	0.82695	0.53054	rs9915021	2.01E-09	5.61E-05	NA	NA	Breast_Mammary_Tissue
ENSG00000108733	PEX12	17	33901814	33905882	ENSG00000198959	TGM2	20	36756863	36794980	0.013634	13.358	5.87E-13	3.14E-07	0.0044937	0.82695	0.53054	rs10512472	9.83E-09	0.00016316	1.06E-12	0	Brain_Caudate_basal_ganglia
ENSG00000142082	SIRT3	11	215458	236431	ENSG00000049323	LTBP1	2	33172039	33624576	0.012332	20.12	5.90E-13	3.15E-07	0.012937	3.0046	0.0177	rs11602954	4.93E-09	0.00012117	1.39E-80	0	Adipose_Subcutaneous
ENSG00000142082	SIRT3	11	215458	236431	ENSG00000049323	LTBP1	2	33172039	33624576	0.012332	20.12	5.90E-13	3.15E-07	0.012937	3.0046	0.0177	rs11602954	4.93E-09	0.00012117	1.39E-80	0	Artery_Tibial
ENSG00000142082	SIRT3	11	215458	236431	ENSG00000049323	LTBP1	2	33172039	33624576	0.012332	20.12	5.90E-13	3.15E-07	0.012937	3.0046	0.0177	rs11602954	4.93E-09	0.00012117	1.39E-80	0	Breast_Mammary_Tissue
ENSG00000142082	SIRT3	11	215458	236431	ENSG00000049323	LTBP1	2	33172039	33624576	0.012332	20.12	5.90E-13	3.15E-07	0.012937	3.0046	0.0177	rs11602954	4.93E-09	0.00012117	1.39E-80	0	Cells_Transformed_fibroblasts
ENSG00000142082	SIRT3	11	215458	236431	ENSG00000049323	LTBP1	2	33172039	33624576	0.012332	20.12	5.90E-13	3.15E-07	0.012937	3.0046	0.0177	rs11602954	4.93E-09	0.00012117	1.39E-80	0	Nerve_Tibial
ENSG00000142082	SIRT3	11	215458	236431	ENSG00000049323	LTBP1	2	33172039	33624576	0.012332	20.12	5.90E-13	3.15E-07	0.012937	3.0046	0.0177	rs505404	1.77E-15	0	1.05E-94	0	Adrenal_Gland
ENSG00000142082	SIRT3	11	215458	236431	ENSG00000049323	LTBP1	2	33172039	33624576	0.012332	20.12	5.90E-13	3.15E-07	0.012937	3.0046	0.0177	rs505404	1.77E-15	0	1.05E-94	0	Artery_Aorta
ENSG00000142082	SIRT3	11	215458	236431	ENSG00000049323	LTBP1	2	33172039	33624576	0.012332	20.12	5.90E-13	3.15E-07	0.012937	3.0046	0.0177	rs505404	1.77E-15	0	1.05E-94	0	Lung
ENSG00000142082	SIRT3	11	215458	236431	ENSG00000049323	LTBP1	2	33172039	33624576	0.012332	20.12	5.90E-13	3.15E-07	0.012937	3.0046	0.0177	rs505404	1.77E-15	0	1.05E-94	0	Thyroid
ENSG00000108733	PEX12	17	33901814	33905882	ENSG00000123908	AGO2	8	141541264	141645718	0.013603	13.327	6.32E-13	3.35E-07	NA	NA	NA	rs9915021	4.09E-08	0.00058148	NA	NA	Brain_Caudate_basal_ganglia
ENSG00000108733	PEX12	17	33901814	33905882	ENSG00000123908	AGO2	8	141541264	141645718	0.013603	13.327	6.32E-13	3.35E-07	NA	NA	NA	rs9915021	4.09E-08	0.00058148	NA	NA	Breast_Mammary_Tissue
ENSG00000108733	PEX12	17	33901814	33905882	ENSG00000123908	AGO2	8	141541264	141645718	0.013603	13.327	6.32E-13	3.35E-07	NA	NA	NA	rs10512472	1.33E-11	0	1.06E-12	0	Brain_Caudate_basal_ganglia
ENSG00000177951	BET1L	11	167784	207428	ENSG00000259207	ITGB3	17	45387505	45389182	0.015282	9.3678	6.46E-13	3.42E-07	0.03348	2.8656	0.0010463	rs11602954	1.90E-32	0	9.12E-206	0	Adipose_Visceral_Omentum
ENSG00000177951	BET1L	11	167784	207428	ENSG00000259207	ITGB3	17	45387505	45389182	0.015282	9.3678	6.46E-13	3.42E-07	0.03348	2.8656	0.0010463	rs11602954	1.90E-32	0	9.12E-206	0	Artery_Aorta
ENSG00000177951	BET1L	11	167784	207428	ENSG00000259207	ITGB3	17	45387505	45389182	0.015282	9.3678	6.46E-13	3.42E-07	0.03348	2.8656	0.0010463	rs11602954	1.90E-32	0	9.12E-206	0	Cells_Transformed_fibroblasts
ENSG00000177951	BET1L	11	167784	207428	ENSG00000259207	ITGB3	17	45387505	45389182	0.015282	9.3678	6.46E-13	3.42E-07	0.03348	2.8656	0.0010463	rs11602954	1.90E-32	0	9.12E-206	0	Pituitary
ENSG00000177951	BET1L	11	167784	207428	ENSG00000259207	ITGB3	17	45387505	45389182	0.015282	9.3678	6.46E-13	3.42E-07	0.03348	2.8656	0.0010463	rs11602954	1.90E-32	0	9.12E-206	0	Prostate
ENSG00000177951	BET1L	11	167784	207428	ENSG00000259207	ITGB3	17	45387505	45389182	0.015282	9.3678	6.46E-13	3.42E-07	0.03348	2.8656	0.0010463	rs11602954	1.90E-32	0	9.12E-206	0	Small_Intestine_Terminal_Ileum
ENSG00000177951	BET1L	11	167784	207428	ENSG00000259207	ITGB3	17	45387505	45389182	0.015282	9.3678	6.46E-13	3.42E-07	0.03348	2.8656	0.0010463	rs11602954	1.90E-32	0	9.12E-206	0	Whole_Blood
ENSG00000177951	BET1L	11	167784	207428	ENSG00000259207	ITGB3	17	45387505	45389182	0.015282	9.3678	6.46E-13	3.42E-07	0.03348	2.8656	0.0010463	rs17655730	6.92E-36	0	2.96E-166	0	Prostate
ENSG00000177951	BET1L	11	167784	207428	ENSG00000259207	ITGB3	17	45387505	45389182	0.015282	9.3678	6.46E-13	3.42E-07	0.03348	2.8656	0.0010463	rs505404	5.02E-41	0	3.73E-187	0	Small_Intestine_Terminal_Ileum
ENSG00000177951	BET1L	11	167784	207428	ENSG00000259207	ITGB3	17	45387505	45389182	0.015282	9.3678	6.46E-13	3.42E-07	0.03348	2.8656	0.0010463	rs505404	5.02E-41	0	3.73E-187	0	Whole_Blood
ENSG00000177951	BET1L	11	167784	207428	ENSG00000259207	ITGB3	17	45387505	45389182	0.015282	9.3678	6.46E-13	3.42E-07	0.03348	2.8656	0.0010463	rs3782123	1.71E-09	5.69E-05	NA	NA	Heart_Atrial_Appendage
ENSG00000006125	AP2B1	17	33913918	34053436	ENSG00000153162	BMP6	6	7727030	7880334	0.014722	10.31	6.78E-13	3.58E-07	0.016673	2.5858	0.017242	rs10512472	2.80E-30	0	3.62E-14	0	Heart_Left_Ventricle

ENSG00000177963	RIC8A	11	207511	215113	ENSG00000049323	LTBP1	2	33172039	33624576	0.013572	13.296	6.79E-13	3.58E-07	0.019478	2.267	0.021096	rs11602954	4.93E-09	0.00012117	7.55E-62	0	Adipose_Subcutaneous
ENSG00000177963	RIC8A	11	207511	215113	ENSG00000049323	LTBP1	2	33172039	33624576	0.013572	13.296	6.79E-13	3.58E-07	0.019478	2.267	0.021096	rs11602954	4.93E-09	0.00012117	7.55E-62	0	Artery_Aorta
ENSG00000177963	RIC8A	11	207511	215113	ENSG00000049323	LTBP1	2	33172039	33624576	0.013572	13.296	6.79E-13	3.58E-07	0.019478	2.267	0.021096	rs11602954	4.93E-09	0.00012117	7.55E-62	0	Artery_Tibial
ENSG00000177963	RIC8A	11	207511	215113	ENSG00000049323	LTBP1	2	33172039	33624576	0.013572	13.296	6.79E-13	3.58E-07	0.019478	2.267	0.021096	rs11602954	4.93E-09	0.00012117	7.55E-62	0	Brain_Caudate_basal_ganglia
ENSG00000177963	RIC8A	11	207511	215113	ENSG00000049323	LTBP1	2	33172039	33624576	0.013572	13.296	6.79E-13	3.58E-07	0.019478	2.267	0.021096	rs11602954	4.93E-09	0.00012117	7.55E-62	0	Brain_Frontal_Cortex_BA9
ENSG00000177963	RIC8A	11	207511	215113	ENSG00000049323	LTBP1	2	33172039	33624576	0.013572	13.296	6.79E-13	3.58E-07	0.019478	2.267	0.021096	rs11602954	4.93E-09	0.00012117	7.55E-62	0	Colon_Sigmoid
ENSG00000177963	RIC8A	11	207511	215113	ENSG00000049323	LTBP1	2	33172039	33624576	0.013572	13.296	6.79E-13	3.58E-07	0.019478	2.267	0.021096	rs11602954	4.93E-09	0.00012117	7.55E-62	0	Colon_Transverse
ENSG00000177963	RIC8A	11	207511	215113	ENSG00000049323	LTBP1	2	33172039	33624576	0.013572	13.296	6.79E-13	3.58E-07	0.019478	2.267	0.021096	rs11602954	4.93E-09	0.00012117	7.55E-62	0	Esophagus_Gastroesophageal_Junction
ENSG00000177963	RIC8A	11	207511	215113	ENSG00000049323	LTBP1	2	33172039	33624576	0.013572	13.296	6.79E-13	3.58E-07	0.019478	2.267	0.021096	rs11602954	4.93E-09	0.00012117	7.55E-62	0	Esophagus_Mucosa
ENSG00000177963	RIC8A	11	207511	215113	ENSG00000049323	LTBP1	2	33172039	33624576	0.013572	13.296	6.79E-13	3.58E-07	0.019478	2.267	0.021096	rs11602954	4.93E-09	0.00012117	7.55E-62	0	Esophagus_Muscularis
ENSG00000177963	RIC8A	11	207511	215113	ENSG00000049323	LTBP1	2	33172039	33624576	0.013572	13.296	6.79E-13	3.58E-07	0.019478	2.267	0.021096	rs11602954	4.93E-09	0.00012117	7.55E-62	0	Lung
ENSG00000177963	RIC8A	11	207511	215113	ENSG00000049323	LTBP1	2	33172039	33624576	0.013572	13.296	6.79E-13	3.58E-07	0.019478	2.267	0.021096	rs11602954	4.93E-09	0.00012117	7.55E-62	0	Muscle_Skeletal
ENSG00000177963	RIC8A	11	207511	215113	ENSG00000049323	LTBP1	2	33172039	33624576	0.013572	13.296	6.79E-13	3.58E-07	0.019478	2.267	0.021096	rs11602954	4.93E-09	0.00012117	7.55E-62	0	Nerve_Tibial
ENSG00000177963	RIC8A	11	207511	215113	ENSG00000049323	LTBP1	2	33172039	33624576	0.013572	13.296	6.79E-13	3.58E-07	0.019478	2.267	0.021096	rs11602954	4.93E-09	0.00012117	7.55E-62	0	Skin_Not_Sun_Exposed_Suprapubic
ENSG00000177963	RIC8A	11	207511	215113	ENSG00000049323	LTBP1	2	33172039	33624576	0.013572	13.296	6.79E-13	3.58E-07	0.019478	2.267	0.021096	rs11602954	4.93E-09	0.00012117	7.55E-62	0	Skin_Sun_Exposed_Lower_leg
ENSG00000177963	RIC8A	11	207511	215113	ENSG00000049323	LTBP1	2	33172039	33624576	0.013572	13.296	6.79E-13	3.58E-07	0.019478	2.267	0.021096	rs11602954	4.93E-09	0.00012117	7.55E-62	0	Thyroid
ENSG00000177963	RIC8A	11	207511	215113	ENSG00000049323	LTBP1	2	33172039	33624576	0.013572	13.296	6.79E-13	3.58E-07	0.019478	2.267	0.021096	rs17655730	2.15E-13	0	5.10E-36	0	Adipose_Subcutaneous
ENSG00000177963	RIC8A	11	207511	215113	ENSG00000049323	LTBP1	2	33172039	33624576	0.013572	13.296	6.79E-13	3.58E-07	0.019478	2.267	0.021096	rs17655730	2.15E-13	0	5.10E-36	0	Esophagus_Mucosa
ENSG00000177963	RIC8A	11	207511	215113	ENSG00000049323	LTBP1	2	33172039	33624576	0.013572	13.296	6.79E-13	3.58E-07	0.019478	2.267	0.021096	rs17655730	2.15E-13	0	5.10E-36	0	Esophagus_Muscularis
ENSG00000177963	RIC8A	11	207511	215113	ENSG00000049323	LTBP1	2	33172039	33624576	0.013572	13.296	6.79E-13	3.58E-07	0.019478	2.267	0.021096	rs17655730	2.15E-13	0	5.10E-36	0	Heart_Left_Ventricle
ENSG00000177963	RIC8A	11	207511	215113	ENSG00000049323	LTBP1	2	33172039	33624576	0.013572	13.296	6.79E-13	3.58E-07	0.019478	2.267	0.021096	rs17655730	2.15E-13	0	5.10E-36	0	Ovary
ENSG00000177963	RIC8A	11	207511	215113	ENSG00000049323	LTBP1	2	33172039	33624576	0.013572	13.296	6.79E-13	3.58E-07	0.019478	2.267	0.021096	rs17655730	2.15E-13	0	5.10E-36	0	Pancreas
ENSG00000177963	RIC8A	11	207511	215113	ENSG00000049323	LTBP1	2	33172039	33624576	0.013572	13.296	6.79E-13	3.58E-07	0.019478	2.267	0.021096	rs17655730	2.15E-13	0	5.10E-36	0	Skin_Not_Sun_Exposed_Suprapubic
ENSG00000177963	RIC8A	11	207511	215113	ENSG00000049323	LTBP1	2	33172039	33624576	0.013572	13.296	6.79E-13	3.58E-07	0.019478	2.267	0.021096	rs505404	1.77E-15	0	3.66E-47	0	Artery_Aorta
ENSG00000177963	RIC8A	11	207511	215113	ENSG00000049323	LTBP1	2	33172039	33624576	0.013572	13.296	6.79E-13	3.58E-07	0.019478	2.267	0.021096	rs505404	1.77E-15	0	3.66E-47	0	Thyroid
ENSG00000141150	RASL10B	17	34058668	34070540	ENSG00000167992	VWCE	11	61025762	61062896	0.015226	9.3326	7.35E-13	3.82E-07	0.021252	2.8352	0.0062857	rs9915021	1.45E-41	0	NA	NA	Cells_Transformed_fibroblasts
ENSG00000174885	NLRP6	11	278365	285359	ENSG00000049323	LTBP1	2	33172039	33624576	0.014672	10.274	7.60E-13	3.94E-07	0.023447	2.433	0.009807	rs11602954	4.93E-09	0.00012117	2.12E-70	0	Nerve_Tibial
ENSG00000174885	NLRP6	11	278365	285359	ENSG00000049323	LTBP1	2	33172039	33624576	0.014672	10.274	7.60E-13	3.94E-07	0.023447	2.433	0.009807	rs17655730	2.15E-13	0	5.43E-117	0	Artery_Aorta
ENSG00000174885	NLRP6	11	278365	285359	ENSG00000049323	LTBP1	2	33172039	33624576	0.014672	10.274	7.60E-13	3.94E-07	0.023447	2.433	0.009807	rs17655730	2.15E-13	0	5.43E-117	0	Nerve_Tibial
ENSG00000174885	NLRP6	11	278365	285359	ENSG00000049323	LTBP1	2	33172039	33624576	0.014672	10.274	7.60E-13	3.94E-07	0.023447	2.433	0.009807	rs17655730	2.15E-13	0	5.43E-117	0	Skin_Sun_Exposed_Lower_leg
ENSG00000174885	NLRP6	11	278365	285359	ENSG00000049323	LTBP1	2	33172039	33624576	0.014672	10.274	7.60E-13	3.94E-07	0.023447	2.433	0.009807	rs505404	1.77E-15	0	2.65E-88	0	Nerve_Tibial
ENSG00000174885	NLRP6	11	278365	285359	ENSG00000049323	LTBP1	2	33172039	33624576	0.014672	10.274	7.60E-13	3.94E-07	0.023447	2.433	0.009807	rs505404	1.77E-15	0	2.65E-88	0	Prostate
ENSG00000006125	AP2B1	17	33913918	34053436	ENSG00000140564	FURIN	15	91411822	91426688	0.014659	10.265	7.83E-13	4.05E-07	0.0044699	0.68472	0.66205	rs10512472	1.96E-14	0	3.62E-14	0	Heart_Left_Ventricle
ENSG00000141150	RASL10B	17	34058668	34070540	ENSG00000130300	PLVAP	19	17462257	17488159	0.015195	9.3135	7.87E-13	4.06E-07	0.024106	3.2253	0.0021985	rs9915021	2.45E-12	0	NA	NA	Cells_Transformed_fibroblasts
ENSG00000168374	ARF4	3	57557090	57583947	ENSG00000174175	SELP	1	169558087	169599431	0.013507	13.232	7.90E-13	4.07E-07	0.026246	4.9379	0.00018166	rs17825630	1.73E-18	0	NA	NA	Artery_Tibial
ENSG00000168374	ARF4	3	57557090	57583947	ENSG00000174175	SELP	1	169558087	169599431	0.013507	13.232	7.90E-13	4.07E-07	0.026246	4.9379	0.00018166	rs17825630	1.73E-18	0	NA	NA	Heart_Atrial_Appendage
ENSG00000174885	NLRP6	11	278365	285359	ENSG00000122786	CALD1	7	134429003	134655479	0.01465	10.259	7.99E-13	4.10E-07	0.015145	1.5583	0.12335	rs11602954	3.38E-37	0	2.12E-70	0	Nerve_Tibial
ENSG00000174885	NLRP6	11	278365	285359	ENSG00000122786	CALD1	7	134429003	134655479	0.01465	10.259	7.99E-13	4.10E-07	0.015145	1.5583	0.12335	rs17655730	3.56E-40	0	5.43E-117	0	Artery_Aorta
ENSG00000174885	NLRP6	11	278365	285359	ENSG00000122786	CALD1	7	134429003	134655479	0.01465	10.259	7.99E-13	4.10E-07	0.015145	1.5583	0.12335	rs17655730	3.56E-40	0	5.43E-117	0	Nerve_Tibial
ENSG00000174885	NLRP6	11	278365	285359	ENSG00000122786	CALD1	7	134429003	134655479	0.01465	10.259	7.99E-13	4.10E-07	0.015145	1.5583	0.12335	rs17655730	3.56E-40	0	5.43E-117	0	Skin_Sun_Exposed_Lower_leg
ENSG00000174885	NLRP6	11	278365	285359	ENSG00000122786	CALD1	7	134429003	134655479	0.01465	10.259	7.99E-13	4.10E-07	0.015145	1.5583	0.12335	rs505404	1.84E-40	0	2.65E-88	0	Nerve_Tibial
ENSG00000174885	NLRP6	11	278365	285359	ENSG00000122786	CALD1	7	134429003	134655479	0.01465	10.259	7.99E-13	4.10E-07	0.015145	1.5583	0.12335	rs505404	1.84E-40	0	2.65E-88	0	Prostate
ENSG00000006125	AP2B1	17	33913918	34053436	ENSG00000204463	BAG6	6	31606805	31620170	0.014647	10.257	8.05E-13	4.12E-07	0.045988	7.3513	1.08E-07	rs10512472	7.83E-17	0	3.62E-14	0	Heart_Left_Ventricle
ENSG00000172660	TAF15	17	34136459	34174246	ENSG00000138867	GUCD1	22	24936406	24951284	0.013986	11.421	1.02E-12	5.13E-07	NA	NA	NA	rs9915021	1.78E-10	0	NA	NA	Skin_Not_Sun_Exposed_Suprapubic
ENSG00000172660	TAF15	17	34136459	34174246	ENSG00000017483	SLC38A5	X	48316920	48328644	0.013954	11.394	1.09E-12	5.50E-07	0.021526	3.355	0.0028206	rs9915021	6.46E-06	0.04076003	NA	NA	Skin_Not_Sun_Exposed_Suprapubic
ENSG00000006125	AP2B1	17	33913918	34053436	ENSG00000161911	TREML1	6	41117080	41122075	0.014474	10.134	1.19E-12	5.94E-07	0.016227	2.5154	0.020242	rs10512472	2.34E-44	0	3.62E-14	0	Heart_Left_Ventricle
ENSG00000168374	ARF4	3	57557090	57583947	ENSG00000140479	PCSK6	15	101840818	102065405	0.013309	13.035	1.26E-12	6.21E-07	0.052812	10.215	1.54E-09	rs17825630	7.20E-06	0.04434651	NA	NA	Artery_Tibial
ENSG00000168374	ARF4	3	57557090	57583947	ENSG00000140479	PCSK6	15	101840818	102065405	0.013309	13.035	1.26E-12	6.21E-07	0.052812	10.215	1.54E-09	rs17825630	7.20E-06	0.04434651	NA	NA	Heart_Atrial_Appendage
ENSG00000172660	TAF15	17	34136459	34174246	ENSG00000160445	ZER1	9	131492065	131534693	0.013882	11.335	1.29E-12	6.36E-07	0.010705	1.6501	0.13028	rs9915021	1.28E-09	5.83E-05	NA	NA	Skin_Not_Sun_Exposed_Suprapubic
ENSG00000172716	SLFN11	17	33677324	33700720	ENSG00000022840	RNF10	12	120971283	121015397	0.016955	6.9347	1.32E-12	6.46E-07	0.029648	2.3145	0.0065259	rs225245	1.95E-178	0	5.77E-07	0.001884469	Cells_Transformed_fibroblasts
ENSG00000172716	SLFN11	17	33677324	33700720	ENSG00000022840	RNF10	12	120971283	121015397	0.016955	6.9347	1.32E-12	6.46E-07	0.029648	2.3145	0.0065259	rs8082605	1.03E-82	0	NA	NA	Adrenal_Gland
ENSG00000092871	RFFL	17	33341759	33416338	ENSG00000022840	RNF10	12	120971283	121015397	0.013849	11.307	1.40E-12	6.82E-07	0.0063534	0.72971	0.66535	rs9915021	1.07E-57	0	NA	NA	Adipose_Subcutaneous
ENSG00000092871	RFFL	17	33341759	33416338	ENSG00000022840	RNF10	12	120971283	121015397	0.013849	11.307	1.40E-12	6.82E-07	0.0063534	0.72971	0.66535	rs9915021	1.07E-57	0	NA	NA	Artery_Tibial
ENSG00000006125	AP2B1	17	33913918	34053436	ENSG00000160445	ZER1	9	131492065	131534693	0.014389	10.073	1.45E-12	7.08E-07	0.0075822	1.1651	0.32276	rs10512472	7.27E-06	0.04465132	3.62E-14	0	Heart_Left_Ventricle
ENSG00000154760	SLFN13	17	33762115	33775856	ENSG00000164068	RNF123	3	49728563	49753910	0.016405	7.3175	1.54E-12	7.50E-07	0.012507	0.95938	0.48637	rs225245	4.45E-47	0	NA	NA	Liver
ENSG00000154760	SLFN13	17	33762115	33775856	ENSG00000164068	RNF123	3	49728563	49753910	0.016405	7.3175	1.54E-12	7.50E-07	0.012507	0.95938	0.48637	rs8082605	5.08E-22	0	2.08E-19	0	Artery_Tibial
ENSG00000154760	SLFN13	17	33762115	33775856	ENSG00000164068	RNF123	3	49728563	49753910	0.016405	7.3175	1.54E-12	7.50E-07	0.012507	0.95938	0.48637	rs8082605	5.08E-22	0	2.08E-19	0	Esophagus_Mucosa
ENSG00000154760	SLFN13	17	33762115	33775856	ENSG00000164068	RNF123	3	49728563	49753910	0.016405	7.3175	1.54E-12	7.50E-07	0.012507	0.95938	0.48637	rs8082605	5.08E-22	0	2.08E-19	0	Nerve_Tibial
ENSG00000154760	SLFN13	17	33762115	33775856	ENSG00000164068	RNF123	3	49728563	49753910	0.016405	7.3175	1.54E-12	7.50E-07	0.012507	0.95938	0.48637	rs8082605	5.08E-22	0	2.08E-19	0	Pituitary
ENSG00000154760	SLFN13	17	33762115	33775856	ENSG00000164068	RNF123	3	49728563	49753910	0.016405	7.3175	1.54E-12	7.50E-07	0.012507	0.95938	0.48637	rs10512472	3.54E-12	0	6.34E-38	0	Uterus
ENSG00000075239	ACAT1	11	107992243	108018503	ENSG00000197409	HIST1H3D	6	26197068	26197497	0.015402	8.3916	1.58E-12	7.70E-07	0.051207	5.469	2.42E-07	rs1801516	6.57E-25	0	6.96E-58	0	Brain_Caudate_basal_ganglia
ENSG00000075239	ACAT1	11	107992243	108018503	ENSG00000197409	HIST1H3D	6	26197068	26197497	0.015402	8.3916	1.58E-12	7.70E-07	0.051207	5.469	2.42E-07	rs11212617	5.06E-60	0	6.71E-12	0	Cells_Transformed_fibroblasts
ENSG00000132436	FIGNL1	7	50511831	50518088	ENSG00000187699	C2orf88	2	190744335	191068210	0.010264	50.152	1.63E-12	7.87E-07	0.019555	18.35	2.03E-05	rs12718598	3.27170000000001e-	0	2.35E-199	0	Adipose_Visceral_Omentum
ENSG00000132436	FIGNL1	7	50511831	50518088	ENSG00000187699	C2orf88	2	190744335	191068210	0.010264	50.152	1.63E-12	7.87E-07	0.019555	18.35	2.03E-05	rs12718598	3.27170000000001e-	0	2.35E-199	0	Artery_Coronary
ENSG00000132436	FIGNL1	7	50511831	50518088	ENSG00000187699	C2orf88	2	190744335	191068210	0.010264	50.152	1.63E-12	7.87E-07	0.019555	18.35	2.03E-05	rs12718598	3.27170000000001e-	0	2.35E-199	0	Colon_Sigmoid
ENSG00000132436	FIGNL1	7	50511831	50518088	ENSG00000187699	C2orf88	2	190744335	191068210	0.010264	50.152	1.63E-12	7.87E-07	0.019555	18.35	2.03E-05	rs12718598	3.27170000000001e-	0	2.35E-199	0	Lung
ENSG00000132436	FIGNL1	7	50511831	50518088	ENSG00000187699	C2orf88	2	190744335	191068210	0.010264	50.152	1.63E-12	7.87E-07	0.019555	18.35	2.03E-05	rs12718598	3.27170000000001e-	0	2.35E-199	0	Pituitary
ENSG00000132436	FIGNL1	7	50511831	50518088	ENSG00000187699	C2orf88	2	190744335	191068210	0.010264	50.152	1.63E-12	7.87E-07	0.019555	18.35	2.03E-05	rs12718598	3.27170000000001e-	0	2.35E-199	0	Spleen
ENSG00000132436	FIGNL1	7	50511831	50518088	ENSG00000187699	C2orf88	2	190744335	191068210	0.010264	50.152	1.63E-12	7.87E-07	0.019555	18.35	2.03E-05	rs12718598	3.27170000000001e-	0	2.35E-199	0	Vagina
ENSG00000132436	FIGNL1	7	50511831	50518088	ENSG00000187699	C2orf88	2	190744335	191068210	0.010264	50.152	1.63E-12	7.87E-07	0.019555	18.35	2.03E-05	rs12718597	1.09E-249	0	1.76E-281	0	Brain_Hippocampus
ENSG00000132436	FIGNL1	7	50511831	50518088	ENSG00000187699	C2orf88	2	190744335	191068210	0.010264	50.152	1.63E-12	7.87E-07	0.019555	18.35	2.03E-05	rs12718597	1.09E-249	0	1.76E-281	0	Vagina
ENSG00000108733	PEX12	17	33901814	33905882	ENSG00000160445	ZER1	9	131492065	131534693	0.013178	12.906	1.71E-12	8.22E-07	0.0042708	0.78576	0.56	rs9915021	1.28E-09	5.83E-05	NA	NA	Brain_Caudate_basal_ganglia
ENSG00000108733	PEX12	17	33901814	33905882	ENSG00000160445	ZER1	9	131492065	131534693	0.013178	12.906	1.71E-12	8.22E-07	0.0042708	0.78576	0.56	rs9915021	1.28E-09	5.83E-05	NA	NA	Breast_Mammary_Tissue
ENSG00000108733	PEX12	17	33901814	33905882	ENSG00000160445	ZER1	9	131492065	131534693	0.013178	12.906	1.71E-12	8.22E-07	0.0042708	0.78576	0.56	rs10512472	7.27E-06	0.04465132	1.06E-12	0	Brain_Caudate_basal_ganglia
ENSG00000099849	RASSF7	11	560404	564021	ENSG00000205038	PKHD1L1	8	110374706	110542559	0.014266	9.9859	1.92E-12	9.21E-07	0.0055165	0.72429	0.65144	rs505404	1.11E-15	0	NA	NA	Brain_Frontal_Cortex_BA9
ENSG00000205045	SLFN12L	17	33800708	33864880	ENSG00000198959	TGM2	20	36756863	36794980	0.014256	9.9788	1.96E-12	9.36E-07	0.005445	0.71485	0.65948	rs10512472	9.83E-09	0.00016316	4.96E-30	0	Pancreas
ENSG00000163946	FAM208A	3	56658507	56717265	ENSG00000164116	GUCY1A3	4	156587863	156653501	0.014779	9.0548	2.01E-12	9.55E-07	0.0099012	1.3057	0.24418	rs12485738	1.04E-94	0	NA	NA	Esophagus_Mucosa
ENSG00000163946	FAM208A	3	56658507	56717265	ENSG00000164116	GUCY1A3	4	156587863	156653501	0.014779	9.0548	2.01E-12	9.55E-07	0.0099012	1.3057	0.24418	rs1354034	1.23E-189	0	NA	NA	Heart_Atrial_Appendage
ENSG00000205045	SLFN12L	17	33800708	33864880	ENSG00000103257	SLC7A5	16	87863629	87903094	0.014241	9.9683	2.03E-12	9.62E-07	0.0063025	0.82814	0.56394	rs8082605	1.28E-07	0.00137418	2.49E-31	0	Adipose_Subcutaneous
ENSG00000205045	SLFN12L	17	33800708	33864880	ENSG00000103257	SLC7A5	16	87863629	87903094	0.014241	9.9683	2.03E-12	9.62E-07	0.0063025	0.82814	0.56394	rs8082605	1.28E-07	0.00137418	2.49E-31	0	Adipose_Visceral_Omentum
ENSG00000205045	SLFN12L	17	33800708	33864880	ENSG00000103257	SLC7A5	16	87863629	87903094	0.014241	9.9683	2.03E-12	9.62E-07	0.0063025	0.82814	0.56394	rs8082605	1.28E-07	0.00137418	2.49E-31	0	Heart_Left_Ventricle
ENSG00000205045	SLFN12L	17	33800708	33864880	ENSG00000103257	SLC7A5	16	87863629	87903094	0.014241	9.9683	2.03E-12	9.62E-07	0.0063025	0.82814	0.56394	rs8082605	1.28E-07	0.00137418	2.49E-31	0	Nerve_Tibial

ENSG00000108733	PEX12	17	33901814	33905882	ENSG00000140564	FURIN	15	91411822	91426688	0.013017	12.745	2.49E-12	1.17E-06	0.0018462	0.33885	0.88947	rs9915021	2.81E-16	0	NA	NA	Brain_Caudate_basal_ganglia
ENSG00000108733	PEX12	17	33901814	33905882	ENSG00000140564	FURIN	15	91411822	91426688	0.013017	12.745	2.49E-12	1.17E-06	0.0018462	0.33885	0.88947	rs9915021	2.81E-16	0	NA	NA	Breast_Mammary_Tissue
ENSG00000108733	PEX12	17	33901814	33905882	ENSG00000140564	FURIN	15	91411822	91426688	0.013017	12.745	2.49E-12	1.17E-06	0.0018462	0.33885	0.88947	rs10512472	1.96E-14	0	1.06E-12	0	Brain_Caudate_basal_ganglia
ENSG00000168374	ARF4	3	57557090	57583947	ENSG00000128266	GNAZ	22	23412540	23464889	0.012982	12.711	2.70E-12	1.26E-06	0.013944	2.5907	0.024492	rs17825630	1.04E-07	0.00118756	NA	NA	Artery_Tibial
ENSG00000168374	ARF4	3	57557090	57583947	ENSG00000128266	GNAZ	22	23412540	23464889	0.012982	12.711	2.70E-12	1.26E-06	0.013944	2.5907	0.024492	rs17825630	1.04E-07	0.00118756	NA	NA	Heart_Atrial_Appendage
ENSG00000132139	GAS2L2	17	34071530	34079897	ENSG00000167671	UBXN6	19	4446046	4457819	0.014623	8.9579	2.86E-12	1.33E-06	0.0095475	1.1001	0.36054	rs9915021	2.43E-46	0	NA	NA	Small_Intestine_Terminal_Ileum
ENSG00000186075	ZPBP2	17	38024417	38034149	ENSG00000162551	ALPL	1	21835858	21904905	0.0099868	48.783	3.25E-12	1.50E-06	0.00099116	0.91277	0.33963	rs10445308	1.27E-07	0.0013747	NA	NA	Testis
ENSG00000186075	ZPBP2	17	38024417	38034149	ENSG00000162551	ALPL	1	21835858	21904905	0.0099868	48.783	3.25E-12	1.50E-06	0.00099116	0.91277	0.33963	rs3859192	3.20E-29	0	NA	NA	Testis
ENSG00000186075	ZPBP2	17	38024417	38034149	ENSG00000162551	ALPL	1	21835858	21904905	0.0099868	48.783	3.25E-12	1.50E-06	0.00099116	0.91277	0.33963	rs4794820	1.24E-10	0	NA	NA	Testis
ENSG00000006125	AP2B1	17	33913918	34053436	ENSG00000141084	RANBP10	16	67757005	67840555	0.014032	9.8198	3.27E-12	1.51E-06	0.01076	1.6587	0.12805	rs10512472	1.25E-08	0.00017572	3.62E-14	0	Heart_Left_Ventricle
ENSG00000142082	SIRT3	11	215458	236431	ENSG00000151693	ASAP2	2	9346894	9541525	0.011599	18.909	3.45E-12	1.58E-06	0.0019676	0.45197	0.77101	rs505404	3.11E-08	0.0004087	1.05E-94	0	Adrenal_Gland
ENSG00000142082	SIRT3	11	215458	236431	ENSG00000151693	ASAP2	2	9346894	9541525	0.011599	18.909	3.45E-12	1.58E-06	0.0019676	0.45197	0.77101	rs505404	3.11E-08	0.0004087	1.05E-94	0	Artery_Aorta
ENSG00000142082	SIRT3	11	215458	236431	ENSG00000151693	ASAP2	2	9346894	9541525	0.011599	18.909	3.45E-12	1.58E-06	0.0019676	0.45197	0.77101	rs505404	3.11E-08	0.0004087	1.05E-94	0	Lung
ENSG00000142082	SIRT3	11	215458	236431	ENSG00000151693	ASAP2	2	9346894	9541525	0.011599	18.909	3.45E-12	1.58E-06	0.0019676	0.45197	0.77101	rs505404	3.11E-08	0.0004087	1.05E-94	0	Thyroid
ENSG00000006125	AP2B1	17	33913918	34053436	ENSG00000164068	RNF123	3	49728563	49753910	0.01399	9.7901	3.60E-12	1.64E-06	0.01837	2.8539	0.0092761	rs10512472	3.54E-12	0	3.62E-14	0	Heart_Left_Ventricle
ENSG00000141027	NCOR1	17	15932471	16119405	ENSG00000169429	IL8	4	74606223	74609433	0.013985	9.7868	3.64E-12	1.65E-06	0.042825	5.1061	3.08E-06	rs7210990	1.42E-95	0	3.2717e-310	0	Whole_Blood
ENSG00000099849	RASSF7	11	560404	564021	ENSG00000259207	ITGB3	17	45387505	45389182	0.013976	9.78	3.72E-12	1.68E-06	0.012808	1.694	0.10684	rs505404	5.02E-41	0	NA	NA	Brain_Frontal_Cortex_BA9
ENSG00000099849	RASSF7	11	560404	564021	ENSG00000259207	ITGB3	17	45387505	45389182	0.013976	9.78	3.72E-12	1.68E-06	0.012808	1.694	0.10684	rs3782123	1.71E-09	5.69E-05	NA	NA	Whole_Blood
ENSG00000205045	SLFN12L	17	33800708	33864880	ENSG00000161911	TREML1	6	41117080	41122075	0.013972	9.7771	3.75E-12	1.69E-06	0.0084875	1.1177	0.34947	rs8082605	2.02E-21	0	2.49E-31	0	Adipose_Subcutaneous
ENSG00000205045	SLFN12L	17	33800708	33864880	ENSG00000161911	TREML1	6	41117080	41122075	0.013972	9.7771	3.75E-12	1.69E-06	0.0084875	1.1177	0.34947	rs8082605	2.02E-21	0	2.49E-31	0	Adipose_Visceral_Omentum
ENSG00000205045	SLFN12L	17	33800708	33864880	ENSG00000161911	TREML1	6	41117080	41122075	0.013972	9.7771	3.75E-12	1.69E-06	0.0084875	1.1177	0.34947	rs8082605	2.02E-21	0	2.49E-31	0	Heart_Left_Ventricle
ENSG00000205045	SLFN12L	17	33800708	33864880	ENSG00000161911	TREML1	6	41117080	41122075	0.013972	9.7771	3.75E-12	1.69E-06	0.0084875	1.1177	0.34947	rs8082605	2.02E-21	0	2.49E-31	0	Nerve_Tibial
ENSG00000205045	SLFN12L	17	33800708	33864880	ENSG00000161911	TREML1	6	41117080	41122075	0.013972	9.7771	3.75E-12	1.69E-06	0.0084875	1.1177	0.34947	rs10512472	2.34E-44	0	4.96E-30	0	Pancreas
ENSG00000177951	BET1L	11	167784	207428	ENSG00000205038	PKHD1L1	8	110374706	110542559	0.014491	8.8758	3.85E-12	1.73E-06	0.013569	1.1379	0.32773	rs11602954	3.21E-08	0.00042188	9.12E-206	0	Adipose_Visceral_Omentum
ENSG00000177951	BET1L	11	167784	207428	ENSG00000205038	PKHD1L1	8	110374706	110542559	0.014491	8.8758	3.85E-12	1.73E-06	0.013569	1.1379	0.32773	rs11602954	3.21E-08	0.00042188	9.12E-206	0	Artery_Aorta
ENSG00000177951	BET1L	11	167784	207428	ENSG00000205038	PKHD1L1	8	110374706	110542559	0.014491	8.8758	3.85E-12	1.73E-06	0.013569	1.1379	0.32773	rs11602954	3.21E-08	0.00042188	9.12E-206	0	Cells_Transformed_fibroblasts
ENSG00000177951	BET1L	11	167784	207428	ENSG00000205038	PKHD1L1	8	110374706	110542559	0.014491	8.8758	3.85E-12	1.73E-06	0.013569	1.1379	0.32773	rs11602954	3.21E-08	0.00042188	9.12E-206	0	Pituitary
ENSG00000177951	BET1L	11	167784	207428	ENSG00000205038	PKHD1L1	8	110374706	110542559	0.014491	8.8758	3.85E-12	1.73E-06	0.013569	1.1379	0.32773	rs11602954	3.21E-08	0.00042188	9.12E-206	0	Prostate
ENSG00000177951	BET1L	11	167784	207428	ENSG00000205038	PKHD1L1	8	110374706	110542559	0.014491	8.8758	3.85E-12	1.73E-06	0.013569	1.1379	0.32773	rs11602954	3.21E-08	0.00042188	9.12E-206	0	Small_Intestine_Terminal_Ileum
ENSG00000177951	BET1L	11	167784	207428	ENSG00000205038	PKHD1L1	8	110374706	110542559	0.014491	8.8758	3.85E-12	1.73E-06	0.013569	1.1379	0.32773	rs11602954	3.21E-08	0.00042188	9.12E-206	0	Whole_Blood
ENSG00000177951	BET1L	11	167784	207428	ENSG00000205038	PKHD1L1	8	110374706	110542559	0.014491	8.8758	3.85E-12	1.73E-06	0.013569	1.1379	0.32773	rs17655730	2.44E-13	0	2.96E-166	0	Prostate
ENSG00000177951	BET1L	11	167784	207428	ENSG00000205038	PKHD1L1	8	110374706	110542559	0.014491	8.8758	3.85E-12	1.73E-06	0.013569	1.1379	0.32773	rs505404	1.11E-15	0	3.73E-187	0	Small_Intestine_Terminal_Ileum
ENSG00000177951	BET1L	11	167784	207428	ENSG00000205038	PKHD1L1	8	110374706	110542559	0.014491	8.8758	3.85E-12	1.73E-06	0.013569	1.1379	0.32773	rs505404	1.11E-15	0	3.73E-187	0	Whole_Blood
ENSG00000108733	PEX12	17	33901814	33905882	ENSG00000149260	CAPN5	11	76777979	76837201	0.012817	12.548	3.96E-12	1.77E-06	0.0083499	1.5426	0.17398	rs10512472	1.11E-06	0.00889928	1.06E-12	0	Brain_Caudate_basal_ganglia
ENSG00000168374	ARF4	3	57557090	57583947	ENSG00000081377	CDC14B	9	99258489	99382112	0.012799	12.53	4.13E-12	1.83E-06	0.018089	3.3749	0.0050027	rs17825630	6.08E-17	0	NA	NA	Artery_Tibial
ENSG00000168374	ARF4	3	57557090	57583947	ENSG00000081377	CDC14B	9	99258489	99382112	0.012799	12.53	4.13E-12	1.83E-06	0.018089	3.3749	0.0050027	rs17825630	6.08E-17	0	NA	NA	Heart_Atrial_Appendage
ENSG00000108733	PEX12	17	33901814	33905882	ENSG00000134779	TPGS2	18	34361762	34403834	0.0128	12.53	4.13E-12	1.83E-06	NA	NA	NA	rs9915021	4.75E-06	0.03156441	NA	NA	Brain_Caudate_basal_ganglia
ENSG00000108733	PEX12	17	33901814	33905882	ENSG00000134779	TPGS2	18	34361762	34403834	0.0128	12.53	4.13E-12	1.83E-06	NA	NA	NA	rs9915021	4.75E-06	0.03156441	NA	NA	Breast_Mammary_Tissue
ENSG00000108733	PEX12	17	33901814	33905882	ENSG00000134779	TPGS2	18	34361762	34403834	0.0128	12.53	4.13E-12	1.83E-06	NA	NA	NA	rs10512472	5.55E-06	0.03581884	1.06E-12	0	Brain_Caudate_basal_ganglia
ENSG00000006125	AP2B1	17	33913918	34053436	ENSG00000174175	SELP	1	169558087	169599431	0.013921	9.7411	4.21E-12	1.85E-06	0.0065238	1.0014	0.42298	rs10512472	1.49E-15	0	3.62E-14	0	Heart_Left_Ventricle
ENSG00000205045	SLFN12L	17	33800708	33864880	ENSG00000017483	SLC38A5	X	48316920	48328644	0.013917	9.738	4.25E-12	1.87E-06	0.025374	3.3994	0.0013634	rs8082605	2.72E-07	0.00263795	2.49E-31	0	Adipose_Subcutaneous
ENSG00000205045	SLFN12L	17	33800708	33864880	ENSG00000017483	SLC38A5	X	48316920	48328644	0.013917	9.738	4.25E-12	1.87E-06	0.025374	3.3994	0.0013634	rs8082605	2.72E-07	0.00263795	2.49E-31	0	Adipose_Visceral_Omentum
ENSG00000205045	SLFN12L	17	33800708	33864880	ENSG00000017483	SLC38A5	X	48316920	48328644	0.013917	9.738	4.25E-12	1.87E-06	0.025374	3.3994	0.0013634	rs8082605	2.72E-07	0.00263795	2.49E-31	0	Heart_Left_Ventricle
ENSG00000205045	SLFN12L	17	33800708	33864880	ENSG00000017483	SLC38A5	X	48316920	48328644	0.013917	9.738	4.25E-12	1.87E-06	0.025374	3.3994	0.0013634	rs8082605	2.72E-07	0.00263795	2.49E-31	0	Nerve_Tibial
ENSG00000205045	SLFN12L	17	33800708	33864880	ENSG00000017483	SLC38A5	X	48316920	48328644	0.013917	9.738	4.25E-12	1.87E-06	0.025374	3.3994	0.0013634	rs10512472	1.90E-13	0	4.96E-30	0	Pancreas
ENSG00000205045	SLFN12L	17	33800708	33864880	ENSG00000130300	PLVAP	19	17462257	17488159	0.013907	9.7313	4.35E-12	1.90E-06	0.020377	2.716	0.0086107	rs8082605	1.32E-12	0	2.49E-31	0	Adipose_Subcutaneous
ENSG00000205045	SLFN12L	17	33800708	33864880	ENSG00000130300	PLVAP	19	17462257	17488159	0.013907	9.7313	4.35E-12	1.90E-06	0.020377	2.716	0.0086107	rs8082605	1.32E-12	0	2.49E-31	0	Adipose_Visceral_Omentum
ENSG00000205045	SLFN12L	17	33800708	33864880	ENSG00000130300	PLVAP	19	17462257	17488159	0.013907	9.7313	4.35E-12	1.90E-06	0.020377	2.716	0.0086107	rs8082605	1.32E-12	0	2.49E-31	0	Heart_Left_Ventricle
ENSG00000205045	SLFN12L	17	33800708	33864880	ENSG00000130300	PLVAP	19	17462257	17488159	0.013907	9.7313	4.35E-12	1.90E-06	0.020377	2.716	0.0086107	rs8082605	1.32E-12	0	2.49E-31	0	Nerve_Tibial
ENSG00000205045	SLFN12L	17	33800708	33864880	ENSG00000130300	PLVAP	19	17462257	17488159	0.013907	9.7313	4.35E-12	1.90E-06	0.020377	2.716	0.0086107	rs10512472	9.07E-18	0	4.96E-30	0	Pancreas
ENSG00000172660	TAF15	17	34136459	34174246	ENSG00000140564	FURIN	15	91411822	91426688	0.013356	10.9	4.34E-12	1.90E-06	0.0027118	0.41468	0.86957	rs9915021	2.81E-16	0	NA	NA	Skin_Not_Sun_Exposed_Suprapubic
ENSG00000205045	SLFN12L	17	33800708	33864880	ENSG00000128266	GNAZ	22	23412540	23464889	0.013884	9.7149	4.58E-12	2.00E-06	0.0088941	1.1717	0.31632	rs8082605	3.92E-16	0	2.49E-31	0	Adipose_Subcutaneous
ENSG00000205045	SLFN12L	17	33800708	33864880	ENSG00000128266	GNAZ	22	23412540	23464889	0.013884	9.7149	4.58E-12	2.00E-06	0.0088941	1.1717	0.31632	rs8082605	3.92E-16	0	2.49E-31	0	Adipose_Visceral_Omentum
ENSG00000205045	SLFN12L	17	33800708	33864880	ENSG00000128266	GNAZ	22	23412540	23464889	0.013884	9.7149	4.58E-12	2.00E-06	0.0088941	1.1717	0.31632	rs8082605	3.92E-16	0	2.49E-31	0	Heart_Left_Ventricle
ENSG00000205045	SLFN12L	17	33800708	33864880	ENSG00000128266	GNAZ	22	23412540	23464889	0.013884	9.7149	4.58E-12	2.00E-06	0.0088941	1.1717	0.31632	rs8082605	3.92E-16	0	2.49E-31	0	Nerve_Tibial
ENSG00000205045	SLFN12L	17	33800708	33864880	ENSG00000128266	GNAZ	22	23412540	23464889	0.013884	9.7149	4.58E-12	2.00E-06	0.0088941	1.1717	0.31632	rs10512472	4.37E-21	0	4.96E-30	0	Pancreas
ENSG00000172716	SLFN11	17	33677324	33700720	ENSG00000115649	CNPPD1	2	220036619	220042035	0.016375	6.6937	4.62E-12	2.01E-06	NA	NA	NA	rs225245	1.17E-55	0	5.77E-07	0.001884469	Cells_Transformed_fibroblasts
ENSG00000172716	SLFN11	17	33677324	33700720	ENSG00000115649	CNPPD1	2	220036619	220042035	0.016375	6.6937	4.62E-12	2.01E-06	NA	NA	NA	rs8082605	6.08E-30	0	NA	NA	Adrenal_Gland
ENSG00000154760	SLFN13	17	33762115	33775856	ENSG00000158828	PINK1	1	20959948	20978004	0.015867	7.0734	4.99E-12	2.16E-06	0.014013	1.0766	0.37647	rs225245	1.45E-52	0	NA	NA	Liver
ENSG00000154760	SLFN13	17	33762115	33775856	ENSG00000158828	PINK1	1	20959948	20978004	0.015867	7.0734	4.99E-12	2.16E-06	0.014013	1.0766	0.37647	rs8082605	2.28E-16	0	2.08E-19	0	Artery_Tibial
ENSG00000154760	SLFN13	17	33762115	33775856	ENSG00000158828	PINK1	1	20959948	20978004	0.015867	7.0734	4.99E-12	2.16E-06	0.014013	1.0766	0.37647	rs8082605	2.28E-16	0	2.08E-19	0	Esophagus_Mucosa
ENSG00000154760	SLFN13	17	33762115	33775856	ENSG00000158828	PINK1	1	20959948	20978004	0.015867	7.0734	4.99E-12	2.16E-06	0.014013	1.0766	0.37647	rs8082605	2.28E-16	0	2.08E-19	0	Nerve_Tibial
ENSG00000154760	SLFN13	17	33762115	33775856	ENSG00000158828	PINK1	1	20959948	20978004	0.015867	7.0734	4.99E-12	2.16E-06	0.014013	1.0766	0.37647	rs8082605	2.28E-16	0	2.08E-19	0	Pituitary
ENSG00000154760	SLFN13	17	33762115	33775856	ENSG00000158828	PINK1	1	20959948	20978004	0.015867	7.0734	4.99E-12	2.16E-06	0.014013	1.0766	0.37647	rs10512472	1.16E-16	0	6.34E-38	0	Uterus
ENSG00000154760	SLFN13	17	33762115	33775856	ENSG00000196961	AP2A1	19	50270225	50309510	0.01583	7.0567	5.41E-12	2.32E-06	0.013405	1.0292	0.41917	rs225245	1.75E-29	0	NA	NA	Liver
ENSG00000154760	SLFN13	17	33762115	33775856	ENSG00000196961	AP2A1	19	50270225	50309510	0.01583	7.0567	5.41E-12	2.32E-06	0.013405	1.0292	0.41917	rs8082605	6.57E-08	0.00085447	2.08E-19	0	Artery_Tibial
ENSG00000154760	SLFN13	17	33762115	33775856	ENSG00000196961	AP2A1	19	50270225	50309510	0.01583	7.0567	5.41E-12	2.32E-06	0.013405	1.0292	0.41917	rs8082605	6.57E-08	0.00085447	2.08E-19	0	Esophagus_Mucosa
ENSG00000154760	SLFN13	17	33762115	33775856	ENSG00000196961	AP2A1	19	50270225	50309510	0.01583	7.0567	5.41E-12	2.32E-06	0.013405	1.0292	0.41917	rs8082605	6.57E-08	0.00085447	2.08E-19	0	Nerve_Tibial
ENSG00000154760	SLFN13	17	33762115	33775856	ENSG00000196961	AP2A1	19	50270225	50309510	0.01583	7.0567	5.41E-12	2.32E-06	0.013405	1.0292	0.41917	rs8082605	6.57E-08	0.00085447	2.08E-19	0	Pituitary
ENSG00000154760	SLFN13	17	33762115	33775856	ENSG00000196961	AP2A1	19	50270225	50309510	0.01583	7.0567	5.41E-12	2.32E-06	0.013405	1.0292	0.41917	rs10512472	3.81E-08	0.00049957	6.34E-38	0	Uterus
ENSG00000167914	GSDMA	17	38119226	38134019	ENSG00000162551	ALPL	1	21835858	21904905	0.010669	26.072	5.47E-12	2.34E-06	0.0076425	3.5388	0.029446	rs3859192	3.20E-29	0	6.22E-13	0	Adipose_Subcutaneous
ENSG00000167914	GSDMA	17	38119226	38134019	ENSG00000162551	ALPL	1	21835858	21904905	0.010669	26.072	5.47E-12	2.34E-06	0.0076425	3.5388	0.029446	rs3859192	3.20E-29	0	6.22E-13	0	Adipose_Visceral_Omentum
ENSG00000167914	GSDMA	17	38119226	38134019	ENSG00000162551	ALPL	1	21835858	21904905	0.010669	26.072	5.47E-12	2.34E-06	0.0076425	3.5388	0.029446	rs3859192	3.20E-29	0	6.22E-13	0	Adrenal_Gland
ENSG00000167914	GSDMA	17	38119226	38134019	ENSG00000162551	ALPL	1	21835858	21904905	0.010669	26.072	5.47E-12	2.34E-06	0.0076425	3.5388	0.029446	rs3859192	3.20E-29	0	6.22E-13	0	Artery_Aorta
ENSG00000167914	GSDMA	17	38119226	38134019	ENSG00000162551	ALPL	1	21835858	21904905	0.010669	26.072	5.47E-12	2.34E-06	0.0076425	3.5388	0.029446	rs3859192	3.20E-29	0	6.22E-13	0	Artery_Coronary
ENSG00000167914	GSDMA	17	38119226	38134019	ENSG00000162551	ALPL	1	21835858	21904905	0.010669	26.072	5.47E-12	2.34E-06	0.0076425	3.5388	0.029446	rs3859192	3.20E-29	0	6.22E-13	0	Artery_Tibial
ENSG00000167914	GSDMA	17	38119226	38134019	ENSG00000162551	ALPL	1	21835858	21904905	0.010669	26.072	5.47E-12	2.34E-06	0.0076425	3.5388	0.029446	rs3859192	3.20E-29	0	6.22E-13	0	Breast_Mammary_Tissue
ENSG00000167914	GSDMA	17	38119226	38134019	ENSG00000162551	ALPL	1	21835858	21904905	0.010669	26.072	5.47E-12	2.34E-06	0.0076425	3.5388	0.029446	rs3859192	3.20E-29	0	6.22E-13	0	Cells_Transformed_fibroblasts
ENSG00000167914	GSDMA	17	38119226	38134019	ENSG00000162551	ALPL	1	21835858	21904905	0.010669	26.072	5.47E-12	2.34E-06	0.0076425	3.5388	0.029446	rs3859192	3.20E-29	0	6.22E-13	0	Colon_Sigmoid
ENSG00000167914	GSDMA	17	38119226	38134019	ENSG00000162551	ALPL	1	21835858	21904905	0.010669	26.072	5.47E-12	2.34E-06	0.0076425	3.5388	0.029446	rs3859192	3.20E-29	0	6.22E-13	0	Colon_Transverse
ENSG00000167914	GSDMA	17	38119226	38134019	ENSG00000162551	ALPL	1	21835858	21904905	0.010669	26.072	5.47E-12	2.34E-06	0.0076425	3.5388	0.029446	rs3859192	3.20E-29	0	6.22E-13	0	Esophagus_Gastroesophageal_Junction
ENSG00000167914	GSDMA	17	38119226	38134019	ENSG00000162551	ALPL	1	21835858	21904905	0.010669	26.072	5.47E-12	2.34E-06	0.0076425	3.5388	0.029446	rs3859192	3.20E-29	0	6.22E-13	0	Esophagus_Muscularis

ENSG00000167914	GSDMA	17	38119226	38134019	ENSG00000162551	ALPL	1	21835858	21904905	0.010669	26.072	5.47E-12	2.34E-06	0.0076425	3.5388	0.029446	rs3859192	3.20E-29	0	6.22E-13	0	Heart_Atrial_Appendage
ENSG00000167914	GSDMA	17	38119226	38134019	ENSG00000162551	ALPL	1	21835858	21904905	0.010669	26.072	5.47E-12	2.34E-06	0.0076425	3.5388	0.029446	rs3859192	3.20E-29	0	6.22E-13	0	Heart_Left_Ventricle
ENSG00000167914	GSDMA	17	38119226	38134019	ENSG00000162551	ALPL	1	21835858	21904905	0.010669	26.072	5.47E-12	2.34E-06	0.0076425	3.5388	0.029446	rs3859192	3.20E-29	0	6.22E-13	0	Liver
ENSG00000167914	GSDMA	17	38119226	38134019	ENSG00000162551	ALPL	1	21835858	21904905	0.010669	26.072	5.47E-12	2.34E-06	0.0076425	3.5388	0.029446	rs3859192	3.20E-29	0	6.22E-13	0	Lung
ENSG00000167914	GSDMA	17	38119226	38134019	ENSG00000162551	ALPL	1	21835858	21904905	0.010669	26.072	5.47E-12	2.34E-06	0.0076425	3.5388	0.029446	rs3859192	3.20E-29	0	6.22E-13	0	Muscle_Skeletal
ENSG00000167914	GSDMA	17	38119226	38134019	ENSG00000162551	ALPL	1	21835858	21904905	0.010669	26.072	5.47E-12	2.34E-06	0.0076425	3.5388	0.029446	rs3859192	3.20E-29	0	6.22E-13	0	Nerve_Tibial
ENSG00000167914	GSDMA	17	38119226	38134019	ENSG00000162551	ALPL	1	21835858	21904905	0.010669	26.072	5.47E-12	2.34E-06	0.0076425	3.5388	0.029446	rs3859192	3.20E-29	0	6.22E-13	0	Ovary
ENSG00000167914	GSDMA	17	38119226	38134019	ENSG00000162551	ALPL	1	21835858	21904905	0.010669	26.072	5.47E-12	2.34E-06	0.0076425	3.5388	0.029446	rs3859192	3.20E-29	0	6.22E-13	0	Pancreas
ENSG00000167914	GSDMA	17	38119226	38134019	ENSG00000162551	ALPL	1	21835858	21904905	0.010669	26.072	5.47E-12	2.34E-06	0.0076425	3.5388	0.029446	rs3859192	3.20E-29	0	6.22E-13	0	Small_Intestine_Terminal_Ileum
ENSG00000167914	GSDMA	17	38119226	38134019	ENSG00000162551	ALPL	1	21835858	21904905	0.010669	26.072	5.47E-12	2.34E-06	0.0076425	3.5388	0.029446	rs3859192	3.20E-29	0	6.22E-13	0	Spleen
ENSG00000167914	GSDMA	17	38119226	38134019	ENSG00000162551	ALPL	1	21835858	21904905	0.010669	26.072	5.47E-12	2.34E-06	0.0076425	3.5388	0.029446	rs3859192	3.20E-29	0	6.22E-13	0	Stomach
ENSG00000167914	GSDMA	17	38119226	38134019	ENSG00000162551	ALPL	1	21835858	21904905	0.010669	26.072	5.47E-12	2.34E-06	0.0076425	3.5388	0.029446	rs3859192	3.20E-29	0	6.22E-13	0	Thyroid
ENSG00000167914	GSDMA	17	38119226	38134019	ENSG00000162551	ALPL	1	21835858	21904905	0.010669	26.072	5.47E-12	2.34E-06	0.0076425	3.5388	0.029446	rs3859192	3.20E-29	0	6.22E-13	0	Whole_Blood
ENSG00000167914	GSDMA	17	38119226	38134019	ENSG00000162551	ALPL	1	21835858	21904905	0.010669	26.072	5.47E-12	2.34E-06	0.0076425	3.5388	0.029446	rs17609240	8.77E-13	0	1.53E-144	0	Adipose_Subcutaneous
ENSG00000167914	GSDMA	17	38119226	38134019	ENSG00000162551	ALPL	1	21835858	21904905	0.010669	26.072	5.47E-12	2.34E-06	0.0076425	3.5388	0.029446	rs17609240	8.77E-13	0	1.53E-144	0	Adipose_Visceral_Omentum
ENSG00000167914	GSDMA	17	38119226	38134019	ENSG00000162551	ALPL	1	21835858	21904905	0.010669	26.072	5.47E-12	2.34E-06	0.0076425	3.5388	0.029446	rs17609240	8.77E-13	0	1.53E-144	0	Artery_Aorta
ENSG00000167914	GSDMA	17	38119226	38134019	ENSG00000162551	ALPL	1	21835858	21904905	0.010669	26.072	5.47E-12	2.34E-06	0.0076425	3.5388	0.029446	rs17609240	8.77E-13	0	1.53E-144	0	Breast_Mammary_Tissue
ENSG00000167914	GSDMA	17	38119226	38134019	ENSG00000162551	ALPL	1	21835858	21904905	0.010669	26.072	5.47E-12	2.34E-06	0.0076425	3.5388	0.029446	rs17609240	8.77E-13	0	1.53E-144	0	Colon_Sigmoid
ENSG00000167914	GSDMA	17	38119226	38134019	ENSG00000162551	ALPL	1	21835858	21904905	0.010669	26.072	5.47E-12	2.34E-06	0.0076425	3.5388	0.029446	rs17609240	8.77E-13	0	1.53E-144	0	Esophagus_Gastroesophageal_Junction
ENSG00000167914	GSDMA	17	38119226	38134019	ENSG00000162551	ALPL	1	21835858	21904905	0.010669	26.072	5.47E-12	2.34E-06	0.0076425	3.5388	0.029446	rs17609240	8.77E-13	0	1.53E-144	0	Esophagus_Muscularis
ENSG00000167914	GSDMA	17	38119226	38134019	ENSG00000162551	ALPL	1	21835858	21904905	0.010669	26.072	5.47E-12	2.34E-06	0.0076425	3.5388	0.029446	rs17609240	8.77E-13	0	1.53E-144	0	Heart_Left_Ventricle
ENSG00000167914	GSDMA	17	38119226	38134019	ENSG00000162551	ALPL	1	21835858	21904905	0.010669	26.072	5.47E-12	2.34E-06	0.0076425	3.5388	0.029446	rs17609240	8.77E-13	0	1.53E-144	0	Muscle_Skeletal
ENSG00000167914	GSDMA	17	38119226	38134019	ENSG00000162551	ALPL	1	21835858	21904905	0.010669	26.072	5.47E-12	2.34E-06	0.0076425	3.5388	0.029446	rs3894194	2.28E-14	0	6.08E-29	0	Adipose_Subcutaneous
ENSG00000167914	GSDMA	17	38119226	38134019	ENSG00000162551	ALPL	1	21835858	21904905	0.010669	26.072	5.47E-12	2.34E-06	0.0076425	3.5388	0.029446	rs3894194	2.28E-14	0	6.08E-29	0	Adipose_Visceral_Omentum
ENSG00000167914	GSDMA	17	38119226	38134019	ENSG00000162551	ALPL	1	21835858	21904905	0.010669	26.072	5.47E-12	2.34E-06	0.0076425	3.5388	0.029446	rs3894194	2.28E-14	0	6.08E-29	0	Artery_Tibial
ENSG00000167914	GSDMA	17	38119226	38134019	ENSG00000162551	ALPL	1	21835858	21904905	0.010669	26.072	5.47E-12	2.34E-06	0.0076425	3.5388	0.029446	rs3894194	2.28E-14	0	6.08E-29	0	Colon_Transverse
ENSG00000167914	GSDMA	17	38119226	38134019	ENSG00000162551	ALPL	1	21835858	21904905	0.010669	26.072	5.47E-12	2.34E-06	0.0076425	3.5388	0.029446	rs3894194	2.28E-14	0	6.08E-29	0	Esophagus_Gastroesophageal_Junction
ENSG00000167914	GSDMA	17	38119226	38134019	ENSG00000162551	ALPL	1	21835858	21904905	0.010669	26.072	5.47E-12	2.34E-06	0.0076425	3.5388	0.029446	rs3894194	2.28E-14	0	6.08E-29	0	Heart_Atrial_Appendage
ENSG00000167914	GSDMA	17	38119226	38134019	ENSG00000162551	ALPL	1	21835858	21904905	0.010669	26.072	5.47E-12	2.34E-06	0.0076425	3.5388	0.029446	rs3894194	2.28E-14	0	6.08E-29	0	Muscle_Skeletal
ENSG00000167914	GSDMA	17	38119226	38134019	ENSG00000162551	ALPL	1	21835858	21904905	0.010669	26.072	5.47E-12	2.34E-06	0.0076425	3.5388	0.029446	rs3894194	2.28E-14	0	6.08E-29	0	Whole_Blood
ENSG00000167914	GSDMA	17	38119226	38134019	ENSG00000162551	ALPL	1	21835858	21904905	0.010669	26.072	5.47E-12	2.34E-06	0.0076425	3.5388	0.029446	rs7212938	1.21E-12	0	5.34E-44	0	Adipose_Subcutaneous
ENSG00000167914	GSDMA	17	38119226	38134019	ENSG00000162551	ALPL	1	21835858	21904905	0.010669	26.072	5.47E-12	2.34E-06	0.0076425	3.5388	0.029446	rs7212938	1.21E-12	0	5.34E-44	0	Adipose_Visceral_Omentum
ENSG00000167914	GSDMA	17	38119226	38134019	ENSG00000162551	ALPL	1	21835858	21904905	0.010669	26.072	5.47E-12	2.34E-06	0.0076425	3.5388	0.029446	rs7212938	1.21E-12	0	5.34E-44	0	Artery_Coronary
ENSG00000167914	GSDMA	17	38119226	38134019	ENSG00000162551	ALPL	1	21835858	21904905	0.010669	26.072	5.47E-12	2.34E-06	0.0076425	3.5388	0.029446	rs7212938	1.21E-12	0	5.34E-44	0	Colon_Sigmoid
ENSG00000167914	GSDMA	17	38119226	38134019	ENSG00000162551	ALPL	1	21835858	21904905	0.010669	26.072	5.47E-12	2.34E-06	0.0076425	3.5388	0.029446	rs7212938	1.21E-12	0	5.34E-44	0	Heart_Atrial_Appendage
ENSG00000167914	GSDMA	17	38119226	38134019	ENSG00000162551	ALPL	1	21835858	21904905	0.010669	26.072	5.47E-12	2.34E-06	0.0076425	3.5388	0.029446	rs7212938	1.21E-12	0	5.34E-44	0	Heart_Left_Ventricle
ENSG00000167914	GSDMA	17	38119226	38134019	ENSG00000162551	ALPL	1	21835858	21904905	0.010669	26.072	5.47E-12	2.34E-06	0.0076425	3.5388	0.029446	rs7212938	1.21E-12	0	5.34E-44	0	Thyroid
ENSG00000167914	GSDMA	17	38119226	38134019	ENSG00000162551	ALPL	1	21835858	21904905	0.010669	26.072	5.47E-12	2.34E-06	0.0076425	3.5388	0.029446	rs7212938	1.21E-12	0	5.34E-44	0	Whole_Blood
ENSG00000167914	GSDMA	17	38119226	38134019	ENSG00000162551	ALPL	1	21835858	21904905	0.010669	26.072	5.47E-12	2.34E-06	0.0076425	3.5388	0.029446	rs8078723	3.63E-38	0	6.97E-07	0.002194627	Breast_Mammary_Tissue
ENSG00000167914	GSDMA	17	38119226	38134019	ENSG00000162551	ALPL	1	21835858	21904905	0.010669	26.072	5.47E-12	2.34E-06	0.0076425	3.5388	0.029446	rs8078723	3.63E-38	0	6.97E-07	0.002194627	Esophagus_Gastroesophageal_Junction
ENSG00000167914	GSDMA	17	38119226	38134019	ENSG00000162551	ALPL	1	21835858	21904905	0.010669	26.072	5.47E-12	2.34E-06	0.0076425	3.5388	0.029446	rs4794820	1.24E-10	0	2.26E-66	0	Artery_Tibial
ENSG00000167914	GSDMA	17	38119226	38134019	ENSG00000162551	ALPL	1	21835858	21904905	0.010669	26.072	5.47E-12	2.34E-06	0.0076425	3.5388	0.029446	rs4794820	1.24E-10	0	2.26E-66	0	Cells_Transformed_fibroblasts
ENSG00000167914	GSDMA	17	38119226	38134019	ENSG00000162551	ALPL	1	21835858	21904905	0.010669	26.072	5.47E-12	2.34E-06	0.0076425	3.5388	0.029446	rs4794822	1.31E-36	0	3.02E-06	0.008756123	Breast_Mammary_Tissue
ENSG00000167914	GSDMA	17	38119226	38134019	ENSG00000162551	ALPL	1	21835858	21904905	0.010669	26.072	5.47E-12	2.34E-06	0.0076425	3.5388	0.029446	rs4794822	1.31E-36	0	3.02E-06	0.008756123	Cells_Transformed_fibroblasts
ENSG00000167914	GSDMA	17	38119226	38134019	ENSG00000162551	ALPL	1	21835858	21904905	0.010669	26.072	5.47E-12	2.34E-06	0.0076425	3.5388	0.029446	rs4794822	1.31E-36	0	3.02E-06	0.008756123	Esophagus_Gastroesophageal_Junction
ENSG00000167914	GSDMA	17	38119226	38134019	ENSG00000162551	ALPL	1	21835858	21904905	0.010669	26.072	5.47E-12	2.34E-06	0.0076425	3.5388	0.029446	rs4794822	1.31E-36	0	3.02E-06	0.008756123	Esophagus_Muscularis
ENSG00000167914	GSDMA	17	38119226	38134019	ENSG00000162551	ALPL	1	21835858	21904905	0.010669	26.072	5.47E-12	2.34E-06	0.0076425	3.5388	0.029446	rs4065321	4.43E-35	0	2.52E-12	0	Pancreas
ENSG00000006125	AP2B1	17	33913918	34053436	ENSG00000017483	SLC38A5	X	48316920	48328644	0.013798	9.6535	5.58E-12	2.39E-06	0.03856	6.1162	2.68E-06	rs10512472	1.90E-13	0	3.62E-14	0	Heart_Left_Ventricle
ENSG00000154768	C17orf50	17	34087916	34092098	ENSG00000158856	DMTN	8	21906506	21940038	0.013208	10.777	6.11E-12	2.59E-06	NA	NA	NA	rs9915021	7.02E-65	0	NA	NA	Pituitary
ENSG00000132139	GAS2L2	17	34071530	34079897	ENSG00000017483	SLC38A5	X	48316920	48328644	0.014277	8.7426	6.23E-12	2.63E-06	0.01357	1.57	0.12966	rs9915021	6.46E-06	0.04076003	NA	NA	Small_Intestine_Terminal_Ileum
ENSG00000160447	PKN3	9	131464802	131482562	ENSG00000164821	DEFA4	8	6793344	6795860	0.013188	10.76	6.40E-12	2.69E-06	0.053058	17.145	7.70E-11	rs13289095	1.50E-34	0	3.2717e-310	0	Colon_Transverse
ENSG00000160447	PKN3	9	131464802	131482562	ENSG00000164821	DEFA4	8	6793344	6795860	0.013188	10.76	6.40E-12	2.69E-06	0.053058	17.145	7.70E-11	rs13289095	1.50E-34	0	3.2717e-310	0	Liver
ENSG00000160447	PKN3	9	131464802	131482562	ENSG00000164821	DEFA4	8	6793344	6795860	0.013188	10.76	6.40E-12	2.69E-06	0.053058	17.145	7.70E-11	rs13289095	1.50E-34	0	3.2717e-310	0	Small_Intestine_Terminal_Ileum
ENSG00000160447	PKN3	9	131464802	131482562	ENSG00000164821	DEFA4	8	6793344	6795860	0.013188	10.76	6.40E-12	2.69E-06	0.053058	17.145	7.70E-11	rs13289095	1.50E-34	0	3.2717e-310	0	Stomach
ENSG00000160447	PKN3	9	131464802	131482562	ENSG00000164821	DEFA4	8	6793344	6795860	0.013188	10.76	6.40E-12	2.69E-06	0.053058	17.145	7.70E-11	rs13289095	1.50E-34	0	3.2717e-310	0	Whole_Blood
ENSG00000163946	FAM208A	3	56658507	56717265	ENSG00000085733	CTTN	11	70244510	70282690	0.014232	8.7149	6.89E-12	2.88E-06	0.015996	2.1225	0.038946	rs12485738	2.67E-199	0	NA	NA	Esophagus_Mucosa
ENSG00000163946	FAM208A	3	56658507	56717265	ENSG00000085733	CTTN	11	70244510	70282690	0.014232	8.7149	6.89E-12	2.88E-06	0.015996	2.1225	0.038946	rs1354034	3.27170000000001e-	0	NA	NA	Heart_Atrial_Appendage
ENSG00000163946	FAM208A	3	56658507	56717265	ENSG00000061918	GUCY1B3	4	156680144	156728743	0.01423	8.7133	6.93E-12	2.89E-06	0.03291	4.4433	7.16E-05	rs12485738	5.86E-109	0	NA	NA	Esophagus_Mucosa
ENSG00000163946	FAM208A	3	56658507	56717265	ENSG00000061918	GUCY1B3	4	156680144	156728743	0.01423	8.7133	6.93E-12	2.89E-06	0.03291	4.4433	7.16E-05	rs1354034	1.79E-188	0	NA	NA	Heart_Atrial_Appendage
ENSG00000108733	PEX12	17	33901814	33905882	ENSG00000130300	PLVAP	19	17462257	17488159	0.012574	12.306	6.99E-12	2.91E-06	0.015939	2.9673	0.011525	rs9915021	2.45E-12	0	NA	NA	Brain_Caudate_basal_ganglia
ENSG00000108733	PEX12	17	33901814	33905882	ENSG00000130300	PLVAP	19	17462257	17488159	0.012574	12.306	6.99E-12	2.91E-06	0.015939	2.9673	0.011525	rs9915021	2.45E-12	0	NA	NA	Breast_Mammary_Tissue
ENSG00000108733	PEX12	17	33901814	33905882	ENSG00000130300	PLVAP	19	17462257	17488159	0.012574	12.306	6.99E-12	2.91E-06	0.015939	2.9673	0.011525	rs10512472	9.07E-18	0	1.06E-12	0	Brain_Caudate_basal_ganglia
ENSG00000108733	PEX12	17	33901814	33905882	ENSG00000118046	STK11	19	1189406	1226662	0.012553	12.285	7.35E-12	3.05E-06	0.0016903	0.31018	0.90698	rs9915021	3.14E-08	0.00040834	NA	NA	Brain_Caudate_basal_ganglia
ENSG00000108733	PEX12	17	33901814	33905882	ENSG00000118046	STK11	19	1189406	1226662	0.012553	12.285	7.35E-12	3.05E-06	0.0016903	0.31018	0.90698	rs9915021	3.14E-08	0.00040834	NA	NA	Breast_Mammary_Tissue
ENSG00000205045	SLFN12L	17	33800708	33864880	ENSG00000090674	MCOLN1	19	7587512	7595387	0.013671	9.5638	7.43E-12	3.08E-06	0.0033832	0.44324	0.87503	rs8082605	1.84E-14	0	2.49E-31	0	Adipose_Subcutaneous
ENSG00000205045	SLFN12L	17	33800708	33864880	ENSG00000090674	MCOLN1	19	7587512	7595387	0.013671	9.5638	7.43E-12	3.08E-06	0.0033832	0.44324	0.87503	rs8082605	1.84E-14	0	2.49E-31	0	Adipose_Visceral_Omentum
ENSG00000205045	SLFN12L	17	33800708	33864880	ENSG00000090674	MCOLN1	19	7587512	7595387	0.013671	9.5638	7.43E-12	3.08E-06	0.0033832	0.44324	0.87503	rs8082605	1.84E-14	0	2.49E-31	0	Heart_Left_Ventricle
ENSG00000205045	SLFN12L	17	33800708	33864880	ENSG00000090674	MCOLN1	19	7587512	7595387	0.013671	9.5638	7.43E-12	3.08E-06	0.0033832	0.44324	0.87503	rs8082605	1.84E-14	0	2.49E-31	0	Nerve_Tibial
ENSG00000205045	SLFN12L	17	33800708	33864880	ENSG00000090674	MCOLN1	19	7587512	7595387	0.013671	9.5638	7.43E-12	3.08E-06	0.0033832	0.44324	0.87503	rs10512472	3.45E-14	0	4.96E-30	0	Pancreas
ENSG00000108733	PEX12	17	33901814	33905882	ENSG00000141084	RANBP10	16	67757005	67840555	0.01254	12.272	7.57E-12	3.12E-06	0.00287	0.5273	0.75574	rs9915021	1.13E-06	0.0090001	NA	NA	Brain_Caudate_basal_ganglia
ENSG00000108733	PEX12	17	33901814	33905882	ENSG00000141084	RANBP10	16	67757005	67840555	0.01254	12.272	7.57E-12	3.12E-06	0.00287	0.5273	0.75574	rs9915021	1.13E-06	0.0090001	NA	NA	Breast_Mammary_Tissue
ENSG00000108733	PEX12	17	33901814	33905882	ENSG00000141084	RANBP10	16	67757005	67840555	0.01254	12.272	7.57E-12	3.12E-06	0.00287	0.5273	0.75574	rs10512472	1.25E-08	0.00017572	1.06E-12	0	Brain_Caudate_basal_ganglia
ENSG00000149311	ATM	11	108094805	108239829	ENSG00000197409	HIST1H3D	6	26197068	26197497	0.013636	9.5388	8.05E-12	3.29E-06	0.05302	7.3106	1.49E-08	rs1801516	6.57E-25	0	NA	NA	Thyroid
ENSG00000149311	ATM	11	108094805	108239829	ENSG00000197409	HIST1H3D	6	26197068	26197497	0.013636	9.5388	8.05E-12	3.29E-06	0.05302	7.3106	1.49E-08	rs11212617	5.06E-60	0	NA	NA	Cells_Transformed_fibroblasts
ENSG00000142082	SIRT3	11	215458	236431	ENSG00000122786	CALD1	7	134429003	134655479	0.011216	18.278	8.64E-12	3.52E-06	0.013429	3.1206	0.014538	rs11602954	3.38E-37	0	1.39E-80	0	Adipose_Subcutaneous
ENSG00000142082	SIRT3	11	215458	236431	ENSG00000122786	CALD1	7	134429003	134655479	0.011216	18.278	8.64E-12	3.52E-06	0.013429	3.1206	0.014538	rs11602954	3.38E-37	0	1.39E-80	0	Artery_Tibial
ENSG00000142082	SIRT3	11	215458	236431	ENSG00000122786	CALD1	7	134429003	134655479	0.011216	18.278	8.64E-12	3.52E-06	0.013429	3.1206	0.014538	rs11602954	3.38E-37	0	1.39E-80	0	Breast_Mammary_Tissue
ENSG00000142082	SIRT3	11	215458	236431	ENSG00000122786	CALD1	7	134429003	134655479	0.011216	18.278	8.64E-12	3.52E-06	0.013429	3.1206	0.014538	rs11602954	3.38E-37	0	1.39E-80	0	Cells_Transformed_fibroblasts
ENSG00000142082	SIRT3	11	215458	236431	ENSG00000122786	CALD1	7	134429003	134655479	0.011216	18.278	8.64E-12	3.52E-06	0.013429	3.1206	0.014538	rs11602954	3.38E-37	0	1.39E-80	0	Nerve_Tibial
ENSG00000142082	SIRT3	11	215458	236431	ENSG00000122786	CALD1	7	134429003	134655479	0.011216	18.278	8.64E-12	3.52E-06	0.013429	3.1206	0.014538	rs505404	1.84E-40	0	1.05E-94	0	Adrenal_Gland
ENSG00000142082	SIRT3	11	215458	236431	ENSG00000122786	CALD1	7	134429003	134655479	0.011216	18.278	8.64E-12	3.52E-06	0.013429	3.1206	0.014538	rs505404	1.84E-40	0	1.05E-94	0	Artery_Aorta
ENSG00000142082	SIRT3	11	215458	236431	ENSG00000122786	CALD1	7	134429003	134655479	0.011216	18.278	8.64E-12	3.52E-06	0.013429	3.1206	0.014538	rs505404	1.84E-40	0	1.05E-94	0	Lung

ENSG00000142082	SIRT3	11	215458	236431	ENSG00000122786	CALD1	7	134429003	134655479	0.011216	18.278	8.64E-12	3.52E-06	0.013429	3.1206	0.014538	rs505404	1.84E-40	0	1.05E-94	0	Thyroid
ENSG00000006125	AP2B1	17	33913918	34053436	ENSG00000011105	TSPAN9	12	3186521	3395730	0.013552	9.4791	9.75E-12	3.95E-06	0.05853	9.4808	4.02E-10	rs10512472	5.91E-134	0	3.62E-14	0	Heart_Left_Ventricle
ENSG00000163946	FAM208A	3	56658507	56717265	ENSG00000124491	F13A1	6	6144318	6321246	0.014067	8.6121	9.98E-12	4.04E-06	0.017681	2.3502	0.022108	rs12485738	9.58E-198	0	NA	NA	Esophagus_Mucosa
ENSG00000163946	FAM208A	3	56658507	56717265	ENSG00000124491	F13A1	6	6144318	6321246	0.014067	8.6121	9.98E-12	4.04E-06	0.017681	2.3502	0.022108	rs1354034	3.27170000000001e-	0	NA	NA	Heart_Atrial_Appendage
ENSG00000006125	AP2B1	17	33913918	34053436	ENSG00000198959	TGM2	20	36756863	36794980	0.013536	9.4682	1.01E-11	4.07E-06	0.0068371	1.0498	0.39138	rs10512472	9.83E-09	0.00016316	3.62E-14	0	Heart_Left_Ventricle
ENSG00000154760	SLFN13	17	33762115	33775856	ENSG00000100614	PPM1A	14	60712470	60765805	0.015539	6.925	1.02E-11	4.09E-06	0.025942	2.0175	0.020191	rs225245	1.24E-19	0	NA	NA	Liver
ENSG00000154760	SLFN13	17	33762115	33775856	ENSG00000100614	PPM1A	14	60712470	60765805	0.015539	6.925	1.02E-11	4.09E-06	0.025942	2.0175	0.020191	rs8082605	1.56E-11	0	2.08E-19	0	Artery_Tibial
ENSG00000154760	SLFN13	17	33762115	33775856	ENSG00000100614	PPM1A	14	60712470	60765805	0.015539	6.925	1.02E-11	4.09E-06	0.025942	2.0175	0.020191	rs8082605	1.56E-11	0	2.08E-19	0	Esophagus_Mucosa
ENSG00000154760	SLFN13	17	33762115	33775856	ENSG00000100614	PPM1A	14	60712470	60765805	0.015539	6.925	1.02E-11	4.09E-06	0.025942	2.0175	0.020191	rs8082605	1.56E-11	0	2.08E-19	0	Nerve_Tibial
ENSG00000154760	SLFN13	17	33762115	33775856	ENSG00000100614	PPM1A	14	60712470	60765805	0.015539	6.925	1.02E-11	4.09E-06	0.025942	2.0175	0.020191	rs8082605	1.56E-11	0	2.08E-19	0	Pituitary
ENSG00000154760	SLFN13	17	33762115	33775856	ENSG00000100614	PPM1A	14	60712470	60765805	0.015539	6.925	1.02E-11	4.09E-06	0.025942	2.0175	0.020191	rs10512472	4.22E-11	0	6.34E-38	0	Uterus
ENSG00000108733	PEX12	17	33901814	33905882	ENSG00000164068	RNF123	3	49728563	49753910	0.012412	12.146	1.02E-11	4.10E-06	0.011741	2.1765	0.054728	rs9915021	2.27E-20	0	NA	NA	Brain_Caudate_basal_ganglia
ENSG00000108733	PEX12	17	33901814	33905882	ENSG00000164068	RNF123	3	49728563	49753910	0.012412	12.146	1.02E-11	4.10E-06	0.011741	2.1765	0.054728	rs9915021	2.27E-20	0	NA	NA	Breast_Mammary_Tissue
ENSG00000108733	PEX12	17	33901814	33905882	ENSG00000164068	RNF123	3	49728563	49753910	0.012412	12.146	1.02E-11	4.10E-06	0.011741	2.1765	0.054728	rs10512472	3.54E-12	0	1.06E-12	0	Brain_Caudate_basal_ganglia
ENSG00000154760	SLFN13	17	33762115	33775856	ENSG00000149260	CAPN5	11	76777979	76837201	0.015493	6.9041	1.12E-11	4.51E-06	0.015019	1.155	0.31161	rs225245	2.28E-18	0	NA	NA	Liver
ENSG00000154760	SLFN13	17	33762115	33775856	ENSG00000149260	CAPN5	11	76777979	76837201	0.015493	6.9041	1.12E-11	4.51E-06	0.015019	1.155	0.31161	rs10512472	1.11E-06	0.00889928	6.34E-38	0	Uterus
ENSG00000108733	PEX12	17	33901814	33905882	ENSG00000163554	SPTA1	1	158580278	158656488	0.012366	12.101	1.13E-11	4.54E-06	0.0057717	1.0635	0.37913	rs9915021	5.11E-06	0.03338736	NA	NA	Brain_Caudate_basal_ganglia
ENSG00000108733	PEX12	17	33901814	33905882	ENSG00000163554	SPTA1	1	158580278	158656488	0.012366	12.101	1.13E-11	4.54E-06	0.0057717	1.0635	0.37913	rs9915021	5.11E-06	0.03338736	NA	NA	Breast_Mammary_Tissue
ENSG00000108733	PEX12	17	33901814	33905882	ENSG00000163554	SPTA1	1	158580278	158656488	0.012366	12.101	1.13E-11	4.54E-06	0.0057717	1.0635	0.37913	rs10512472	1.40E-07	0.00152082	1.06E-12	0	Brain_Caudate_basal_ganglia
ENSG00000154760	SLFN13	17	33762115	33775856	ENSG00000167671	UBXN6	19	4446046	4457819	0.01548	6.8984	1.15E-11	4.60E-06	0.029395	2.2941	0.007066	rs225245	6.11E-162	0	NA	NA	Liver
ENSG00000154760	SLFN13	17	33762115	33775856	ENSG00000167671	UBXN6	19	4446046	4457819	0.01548	6.8984	1.15E-11	4.60E-06	0.029395	2.2941	0.007066	rs8082605	3.08E-60	0	2.08E-19	0	Artery_Tibial
ENSG00000154760	SLFN13	17	33762115	33775856	ENSG00000167671	UBXN6	19	4446046	4457819	0.01548	6.8984	1.15E-11	4.60E-06	0.029395	2.2941	0.007066	rs8082605	3.08E-60	0	2.08E-19	0	Esophagus_Mucosa
ENSG00000154760	SLFN13	17	33762115	33775856	ENSG00000167671	UBXN6	19	4446046	4457819	0.01548	6.8984	1.15E-11	4.60E-06	0.029395	2.2941	0.007066	rs8082605	3.08E-60	0	2.08E-19	0	Nerve_Tibial
ENSG00000154760	SLFN13	17	33762115	33775856	ENSG00000167671	UBXN6	19	4446046	4457819	0.01548	6.8984	1.15E-11	4.60E-06	0.029395	2.2941	0.007066	rs8082605	3.08E-60	0	2.08E-19	0	Pituitary
ENSG00000154760	SLFN13	17	33762115	33775856	ENSG00000167671	UBXN6	19	4446046	4457819	0.01548	6.8984	1.15E-11	4.60E-06	0.029395	2.2941	0.007066	rs10512472	4.51E-73	0	6.34E-38	0	Uterus
ENSG00000163946	FAM208A	3	56658507	56717265	ENSG00000172572	PDE3A	12	20522179	20837315	0.013998	8.5694	1.16E-11	4.63E-06	NA	NA	NA	rs12485738	9.24E-27	0	NA	NA	Esophagus_Mucosa
ENSG00000163946	FAM208A	3	56658507	56717265	ENSG00000172572	PDE3A	12	20522179	20837315	0.013998	8.5694	1.16E-11	4.63E-06	NA	NA	NA	rs1354034	5.07E-54	0	NA	NA	Heart_Atrial_Appendage
ENSG00000177951	BET1L	11	167784	207428	ENSG00000138722	MMRN1	4	90800683	90875780	0.013954	8.5423	1.28E-11	5.06E-06	0.01076	0.89979	0.54005	rs11602954	5.94E-65	0	9.12E-206	0	Adipose_Visceral_Omentum
ENSG00000177951	BET1L	11	167784	207428	ENSG00000138722	MMRN1	4	90800683	90875780	0.013954	8.5423	1.28E-11	5.06E-06	0.01076	0.89979	0.54005	rs11602954	5.94E-65	0	9.12E-206	0	Artery_Aorta
ENSG00000177951	BET1L	11	167784	207428	ENSG00000138722	MMRN1	4	90800683	90875780	0.013954	8.5423	1.28E-11	5.06E-06	0.01076	0.89979	0.54005	rs11602954	5.94E-65	0	9.12E-206	0	Cells_Transformed_fibroblasts
ENSG00000177951	BET1L	11	167784	207428	ENSG00000138722	MMRN1	4	90800683	90875780	0.013954	8.5423	1.28E-11	5.06E-06	0.01076	0.89979	0.54005	rs11602954	5.94E-65	0	9.12E-206	0	Pituitary
ENSG00000177951	BET1L	11	167784	207428	ENSG00000138722	MMRN1	4	90800683	90875780	0.013954	8.5423	1.28E-11	5.06E-06	0.01076	0.89979	0.54005	rs11602954	5.94E-65	0	9.12E-206	0	Prostate
ENSG00000177951	BET1L	11	167784	207428	ENSG00000138722	MMRN1	4	90800683	90875780	0.013954	8.5423	1.28E-11	5.06E-06	0.01076	0.89979	0.54005	rs11602954	5.94E-65	0	9.12E-206	0	Small_Intestine_Terminal_Ileum
ENSG00000177951	BET1L	11	167784	207428	ENSG00000138722	MMRN1	4	90800683	90875780	0.013954	8.5423	1.28E-11	5.06E-06	0.01076	0.89979	0.54005	rs11602954	5.94E-65	0	9.12E-206	0	Whole_Blood
ENSG00000177951	BET1L	11	167784	207428	ENSG00000138722	MMRN1	4	90800683	90875780	0.013954	8.5423	1.28E-11	5.06E-06	0.01076	0.89979	0.54005	rs17655730	6.29E-64	0	2.96E-166	0	Prostate
ENSG00000177951	BET1L	11	167784	207428	ENSG00000138722	MMRN1	4	90800683	90875780	0.013954	8.5423	1.28E-11	5.06E-06	0.01076	0.89979	0.54005	rs505404	2.01E-74	0	3.73E-187	0	Small_Intestine_Terminal_Ileum
ENSG00000177951	BET1L	11	167784	207428	ENSG00000138722	MMRN1	4	90800683	90875780	0.013954	8.5423	1.28E-11	5.06E-06	0.01076	0.89979	0.54005	rs505404	2.01E-74	0	3.73E-187	0	Whole_Blood
ENSG00000177951	BET1L	11	167784	207428	ENSG00000138722	MMRN1	4	90800683	90875780	0.013954	8.5423	1.28E-11	5.06E-06	0.01076	0.89979	0.54005	rs3782123	3.86E-09	0.00010601	NA	NA	Heart_Atrial_Appendage
ENSG00000163946	FAM208A	3	56658507	56717265	ENSG00000005961	ITGA2B	17	42449548	42466873	0.013905	8.5117	1.43E-11	5.61E-06	0.035493	4.8049	2.51E-05	rs12485738	3.27170000000001e-	0	NA	NA	Esophagus_Mucosa
ENSG00000163946	FAM208A	3	56658507	56717265	ENSG00000005961	ITGA2B	17	42449548	42466873	0.013905	8.5117	1.43E-11	5.61E-06	0.035493	4.8049	2.51E-05	rs1354034	3.27170000000001e-	0	NA	NA	Heart_Atrial_Appendage
ENSG00000184886	PIGW	17	34892943	34895159	ENSG00000158856	DMTN	8	21906506	21940038	0.011624	14.21	1.56E-11	6.07E-06	NA	NA	NA	rs9915021	7.02E-65	0	NA	NA	Muscle_Skeletal
ENSG00000172123	SLFN12	17	33738079	33760302	ENSG00000238243	OR2W3	1	248058859	248060449	0.017133	5.6036	1.63E-11	6.30E-06	0.011293	1.7419	0.10823	rs225245	4.87E-16	0	NA	NA	Cells_EBV-transformed_lymphocytes
ENSG00000172123	SLFN12	17	33738079	33760302	ENSG00000238243	OR2W3	1	248058859	248060449	0.017133	5.6036	1.63E-11	6.30E-06	0.011293	1.7419	0.10823	rs9915021	7.68E-06	0.04691593	NA	NA	Breast_Mammary_Tissue
ENSG00000205045	SLFN12L	17	33800708	33864880	ENSG00000144567	FAM134A	2	220042939	220050201	0.013285	9.2898	1.79E-11	6.87E-06	0.012604	1.6668	0.11358	rs8082605	2.77E-35	0	2.49E-31	0	Adipose_Subcutaneous
ENSG00000205045	SLFN12L	17	33800708	33864880	ENSG00000144567	FAM134A	2	220042939	220050201	0.013285	9.2898	1.79E-11	6.87E-06	0.012604	1.6668	0.11358	rs8082605	2.77E-35	0	2.49E-31	0	Adipose_Visceral_Omentum
ENSG00000205045	SLFN12L	17	33800708	33864880	ENSG00000144567	FAM134A	2	220042939	220050201	0.013285	9.2898	1.79E-11	6.87E-06	0.012604	1.6668	0.11358	rs8082605	2.77E-35	0	2.49E-31	0	Heart_Left_Ventricle
ENSG00000205045	SLFN12L	17	33800708	33864880	ENSG00000144567	FAM134A	2	220042939	220050201	0.013285	9.2898	1.79E-11	6.87E-06	0.012604	1.6668	0.11358	rs8082605	2.77E-35	0	2.49E-31	0	Nerve_Tibial
ENSG00000205045	SLFN12L	17	33800708	33864880	ENSG00000144567	FAM134A	2	220042939	220050201	0.013285	9.2898	1.79E-11	6.87E-06	0.012604	1.6668	0.11358	rs10512472	6.57E-30	0	4.96E-30	0	Pancreas
ENSG00000142102	ATHL1	11	289138	296107	ENSG00000049323	LTBP1	2	33172039	33624576	0.018774	4.8519	1.82E-11	6.94E-06	0.00023728	0.10906	0.89669	rs11602954	4.93E-09	0.00012117	8.23E-62	0	Thyroid
ENSG00000142102	ATHL1	11	289138	296107	ENSG00000049323	LTBP1	2	33172039	33624576	0.018774	4.8519	1.82E-11	6.94E-06	0.00023728	0.10906	0.89669	rs17655730	2.15E-13	0	1.10E-69	0	Muscle_Skeletal
ENSG00000142102	ATHL1	11	289138	296107	ENSG00000049323	LTBP1	2	33172039	33624576	0.018774	4.8519	1.82E-11	6.94E-06	0.00023728	0.10906	0.89669	rs505404	1.77E-15	0	3.83E-63	0	Lung
ENSG00000177963	RIC8A	11	207511	215113	ENSG00000122786	CALD1	7	134429003	134655479	0.012152	11.888	1.87E-11	7.12E-06	0.017842	2.0733	0.035867	rs11602954	3.38E-37	0	7.55E-62	0	Adipose_Subcutaneous
ENSG00000177963	RIC8A	11	207511	215113	ENSG00000122786	CALD1	7	134429003	134655479	0.012152	11.888	1.87E-11	7.12E-06	0.017842	2.0733	0.035867	rs11602954	3.38E-37	0	7.55E-62	0	Artery_Aorta
ENSG00000177963	RIC8A	11	207511	215113	ENSG00000122786	CALD1	7	134429003	134655479	0.012152	11.888	1.87E-11	7.12E-06	0.017842	2.0733	0.035867	rs11602954	3.38E-37	0	7.55E-62	0	Artery_Tibial
ENSG00000177963	RIC8A	11	207511	215113	ENSG00000122786	CALD1	7	134429003	134655479	0.012152	11.888	1.87E-11	7.12E-06	0.017842	2.0733	0.035867	rs11602954	3.38E-37	0	7.55E-62	0	Brain_Caudate_basal_ganglia
ENSG00000177963	RIC8A	11	207511	215113	ENSG00000122786	CALD1	7	134429003	134655479	0.012152	11.888	1.87E-11	7.12E-06	0.017842	2.0733	0.035867	rs11602954	3.38E-37	0	7.55E-62	0	Brain_Frontal_Cortex_BA9
ENSG00000177963	RIC8A	11	207511	215113	ENSG00000122786	CALD1	7	134429003	134655479	0.012152	11.888	1.87E-11	7.12E-06	0.017842	2.0733	0.035867	rs11602954	3.38E-37	0	7.55E-62	0	Colon_Sigmoid
ENSG00000177963	RIC8A	11	207511	215113	ENSG00000122786	CALD1	7	134429003	134655479	0.012152	11.888	1.87E-11	7.12E-06	0.017842	2.0733	0.035867	rs11602954	3.38E-37	0	7.55E-62	0	Colon_Transverse
ENSG00000177963	RIC8A	11	207511	215113	ENSG00000122786	CALD1	7	134429003	134655479	0.012152	11.888	1.87E-11	7.12E-06	0.017842	2.0733	0.035867	rs11602954	3.38E-37	0	7.55E-62	0	Esophagus_Gastroesophageal_Junction
ENSG00000177963	RIC8A	11	207511	215113	ENSG00000122786	CALD1	7	134429003	134655479	0.012152	11.888	1.87E-11	7.12E-06	0.017842	2.0733	0.035867	rs11602954	3.38E-37	0	7.55E-62	0	Esophagus_Mucosa
ENSG00000177963	RIC8A	11	207511	215113	ENSG00000122786	CALD1	7	134429003	134655479	0.012152	11.888	1.87E-11	7.12E-06	0.017842	2.0733	0.035867	rs11602954	3.38E-37	0	7.55E-62	0	Esophagus_Muscularis
ENSG00000177963	RIC8A	11	207511	215113	ENSG00000122786	CALD1	7	134429003	134655479	0.012152	11.888	1.87E-11	7.12E-06	0.017842	2.0733	0.035867	rs11602954	3.38E-37	0	7.55E-62	0	Lung
ENSG00000177963	RIC8A	11	207511	215113	ENSG00000122786	CALD1	7	134429003	134655479	0.012152	11.888	1.87E-11	7.12E-06	0.017842	2.0733	0.035867	rs11602954	3.38E-37	0	7.55E-62	0	Muscle_Skeletal
ENSG00000177963	RIC8A	11	207511	215113	ENSG00000122786	CALD1	7	134429003	134655479	0.012152	11.888	1.87E-11	7.12E-06	0.017842	2.0733	0.035867	rs11602954	3.38E-37	0	7.55E-62	0	Nerve_Tibial
ENSG00000177963	RIC8A	11	207511	215113	ENSG00000122786	CALD1	7	134429003	134655479	0.012152	11.888	1.87E-11	7.12E-06	0.017842	2.0733	0.035867	rs11602954	3.38E-37	0	7.55E-62	0	Skin_Not_Sun_Exposed_Suprapubic
ENSG00000177963	RIC8A	11	207511	215113	ENSG00000122786	CALD1	7	134429003	134655479	0.012152	11.888	1.87E-11	7.12E-06	0.017842	2.0733	0.035867	rs11602954	3.38E-37	0	7.55E-62	0	Skin_Sun_Exposed_Lower_leg
ENSG00000177963	RIC8A	11	207511	215113	ENSG00000122786	CALD1	7	134429003	134655479	0.012152	11.888	1.87E-11	7.12E-06	0.017842	2.0733	0.035867	rs11602954	3.38E-37	0	7.55E-62	0	Thyroid
ENSG00000177963	RIC8A	11	207511	215113	ENSG00000122786	CALD1	7	134429003	134655479	0.012152	11.888	1.87E-11	7.12E-06	0.017842	2.0733	0.035867	rs17655730	3.56E-40	0	5.10E-36	0	Adipose_Subcutaneous
ENSG00000177963	RIC8A	11	207511	215113	ENSG00000122786	CALD1	7	134429003	134655479	0.012152	11.888	1.87E-11	7.12E-06	0.017842	2.0733	0.035867	rs17655730	3.56E-40	0	5.10E-36	0	Esophagus_Mucosa
ENSG00000177963	RIC8A	11	207511	215113	ENSG00000122786	CALD1	7	134429003	134655479	0.012152	11.888	1.87E-11	7.12E-06	0.017842	2.0733	0.035867	rs17655730	3.56E-40	0	5.10E-36	0	Esophagus_Muscularis
ENSG00000177963	RIC8A	11	207511	215113	ENSG00000122786	CALD1	7	134429003	134655479	0.012152	11.888	1.87E-11	7.12E-06	0.017842	2.0733	0.035867	rs17655730	3.56E-40	0	5.10E-36	0	Heart_Left_Ventricle
ENSG00000177963	RIC8A	11	207511	215113	ENSG00000122786	CALD1	7	134429003	134655479	0.012152	11.888	1.87E-11	7.12E-06	0.017842	2.0733	0.035867	rs17655730	3.56E-40	0	5.10E-36	0	Ovary
ENSG00000177963	RIC8A	11	207511	215113	ENSG00000122786	CALD1	7	134429003	134655479	0.012152	11.888	1.87E-11	7.12E-06	0.017842	2.0733	0.035867	rs17655730	3.56E-40	0	5.10E-36	0	Pancreas
ENSG00000177963	RIC8A	11	207511	215113	ENSG00000122786	CALD1	7	134429003	134655479	0.012152	11.888	1.87E-11	7.12E-06	0.017842	2.0733	0.035867	rs17655730	3.56E-40	0	5.10E-36	0	Skin_Not_Sun_Exposed_Suprapubic
ENSG00000177963	RIC8A	11	207511	215113	ENSG00000122786	CALD1	7	134429003	134655479	0.012152	11.888	1.87E-11	7.12E-06	0.017842	2.0733	0.035867	rs505404	1.84E-40	0	3.66E-47	0	Artery_Aorta
ENSG00000177963	RIC8A	11	207511	215113	ENSG00000122786	CALD1	7	134429003	134655479	0.012152	11.888	1.87E-11	7.12E-06	0.017842	2.0733	0.035867	rs505404	1.84E-40	0	3.66E-47	0	Thyroid
ENSG00000006125	AP2B1	17	33913918	34053436	ENSG00000124491	F13A1	6	6144318	6321246	0.013264	9.2749	1.87E-11	7.12E-06	0.0051386	0.78769	0.57963	rs10512472	3.64E-32	0	3.62E-14	0	Heart_Left_Ventricle
ENSG00000172660	TAF15	17	34136459	34174246	ENSG00000141084	RANBP10	16	67757005	67840555	0.012713	10.368	1.91E-11	7.23E-06	0.0080412	1.2362	0.28513	rs9915021	1.13E-06	0.0090001	NA	NA	Skin_Not_Sun_Exposed_Suprapubic
ENSG00000172660	TAF15	17	34136459	34174246	ENSG00000166169	POLL	10	103338639	103346957	0.012711	10.367	1.91E-11	7.24E-06	0.03479	5.4967	1.33E-05	rs9915021	3.09E-30	0	NA	NA	Skin_Not_Sun_Exposed_Suprapubic
ENSG00000172660	TAF15	17	34136459	34174246	ENSG00000164068	RNF123	3	49728563	49753910	0.012711	10.366	1.92E-11	7.24E-06	0.013116	2.0267	0.059632	rs9915021	2.27E-20	0	NA	NA	Skin_Not_Sun_Exposed_Suprapubic
ENSG00000006125	AP2B1	17	33913918	34053436	ENSG00000196961	AP2A1	19	50270225	50309510	0.013235	9.2545	2.00E-11	7.53E-06	0.0046575	0.7136	0.63872	rs10512472	3.81E-08	0.00049957	3.62E-14	0	Heart_Left_Ventricle
ENSG00000141150	RASL10B	17	34058668	34070540	ENSG00000115649	CNPPD1	2	220036619	220042035	0.013721	8.3976	2.16E-11	8.11E-06	NA	NA	NA	rs9915021	3.39E-19	0	NA	NA	Cells_Transformed_fibroblasts

ENSG00000154760	SLFN13	17	33762115	33775856	ENSG00000164849	GPR146	7	1084212	1098897	0.015186	6.7652	2.19E-11	8.17E-06	0.026816	2.0873	0.015581	rs225245	1.90E-17	0	NA	NA	Liver
ENSG00000168374	ARF4	3	57557090	57583947	ENSG00000138722	MMRN1	4	90800683	90875780	0.012055	11.792	2.34E-11	8.71E-06	0.029187	5.5078	5.27E-05	rs17825630	8.16E-23	0	NA	NA	Artery_Tibial
ENSG00000168374	ARF4	3	57557090	57583947	ENSG00000138722	MMRN1	4	90800683	90875780	0.012055	11.792	2.34E-11	8.71E-06	0.029187	5.5078	5.27E-05	rs17825630	8.16E-23	0	NA	NA	Heart_Atrial_Appendage
ENSG00000006125	AP2B1	17	33913918	34053436	ENSG00000140479	PCSK6	15	101840818	102065405	0.013143	9.1895	2.46E-11	9.09E-06	0.034305	5.4173	1.62E-05	rs10512472	3.03E-22	0	3.62E-14	0	Heart_Left_Ventricle
ENSG00000006125	AP2B1	17	33913918	34053436	ENSG00000134779	TPGS2	18	34361762	34403834	0.013135	9.1839	2.51E-11	9.23E-06	NA	NA	NA	rs10512472	5.55E-06	0.03581884	3.62E-14	0	Heart_Left_Ventricle
ENSG00000108733	PEX12	17	33901814	33905882	ENSG00000184792	OSBP2	22	31089769	31303811	0.012011	11.749	2.59E-11	9.50E-06	0.017577	3.2776	0.0061144	rs9915021	5.31E-07	0.00466469	NA	NA	Brain_Caudate_basal_ganglia
ENSG00000108733	PEX12	17	33901814	33905882	ENSG00000184792	OSBP2	22	31089769	31303811	0.012011	11.749	2.59E-11	9.50E-06	0.017577	3.2776	0.0061144	rs9915021	5.31E-07	0.00466469	NA	NA	Breast_Mammary_Tissue
ENSG00000108733	PEX12	17	33901814	33905882	ENSG00000184792	OSBP2	22	31089769	31303811	0.012011	11.749	2.59E-11	9.50E-06	0.017577	3.2776	0.0061144	rs10512472	2.35E-08	0.00031703	1.06E-12	0	Brain_Caudate_basal_ganglia
ENSG00000157500	APPL1	3	57261765	57301820	ENSG00000138722	MMRN1	4	90800683	90875780	0.011384	13.913	2.75E-11	1.00E-05	0.0010103	0.30946	0.81856	rs12485738	9.40E-304	0	NA	NA	Brain_Anterior_cingulate_cortex_BA24
ENSG00000174885	NLRP6	11	278365	285359	ENSG00000151693	ASAP2	2	9346894	9541525	0.013071	9.1382	2.90E-11	1.06E-05	0.0094413	0.96584	0.46689	rs17655730	6.61E-07	0.00575008	5.43E-117	0	Artery_Aorta
ENSG00000174885	NLRP6	11	278365	285359	ENSG00000151693	ASAP2	2	9346894	9541525	0.013071	9.1382	2.90E-11	1.06E-05	0.0094413	0.96584	0.46689	rs17655730	6.61E-07	0.00575008	5.43E-117	0	Nerve_Tibial
ENSG00000174885	NLRP6	11	278365	285359	ENSG00000151693	ASAP2	2	9346894	9541525	0.013071	9.1382	2.90E-11	1.06E-05	0.0094413	0.96584	0.46689	rs17655730	6.61E-07	0.00575008	5.43E-117	0	Skin_Sun_Exposed_Lower_leg
ENSG00000174885	NLRP6	11	278365	285359	ENSG00000151693	ASAP2	2	9346894	9541525	0.013071	9.1382	2.90E-11	1.06E-05	0.0094413	0.96584	0.46689	rs505404	3.11E-08	0.0004087	2.65E-88	0	Nerve_Tibial
ENSG00000174885	NLRP6	11	278365	285359	ENSG00000151693	ASAP2	2	9346894	9541525	0.013071	9.1382	2.90E-11	1.06E-05	0.0094413	0.96584	0.46689	rs505404	3.11E-08	0.0004087	2.65E-88	0	Prostate
ENSG00000108733	PEX12	17	33901814	33905882	ENSG00000154146	NRGN	11	124609829	124615878	0.011961	11.699	2.91E-11	1.06E-05	0.024775	4.6541	0.00033502	rs9915021	3.58E-48	0	NA	NA	Brain_Caudate_basal_ganglia
ENSG00000108733	PEX12	17	33901814	33905882	ENSG00000154146	NRGN	11	124609829	124615878	0.011961	11.699	2.91E-11	1.06E-05	0.024775	4.6541	0.00033502	rs9915021	3.58E-48	0	NA	NA	Breast_Mammary_Tissue
ENSG00000108733	PEX12	17	33901814	33905882	ENSG00000154146	NRGN	11	124609829	124615878	0.011961	11.699	2.91E-11	1.06E-05	0.024775	4.6541	0.00033502	rs10512472	4.87E-94	0	1.06E-12	0	Brain_Caudate_basal_ganglia
ENSG00000205045	SLFN12L	17	33800708	33864880	ENSG00000153162	BMP6	6	7727030	7880334	0.013063	9.1329	2.95E-11	1.07E-05	0.0044057	0.5778	0.77438	rs8082605	1.24E-17	0	2.49E-31	0	Adipose_Subcutaneous
ENSG00000205045	SLFN12L	17	33800708	33864880	ENSG00000153162	BMP6	6	7727030	7880334	0.013063	9.1329	2.95E-11	1.07E-05	0.0044057	0.5778	0.77438	rs8082605	1.24E-17	0	2.49E-31	0	Adipose_Visceral_Omentum
ENSG00000205045	SLFN12L	17	33800708	33864880	ENSG00000153162	BMP6	6	7727030	7880334	0.013063	9.1329	2.95E-11	1.07E-05	0.0044057	0.5778	0.77438	rs8082605	1.24E-17	0	2.49E-31	0	Heart_Left_Ventricle
ENSG00000205045	SLFN12L	17	33800708	33864880	ENSG00000153162	BMP6	6	7727030	7880334	0.013063	9.1329	2.95E-11	1.07E-05	0.0044057	0.5778	0.77438	rs8082605	1.24E-17	0	2.49E-31	0	Nerve_Tibial
ENSG00000205045	SLFN12L	17	33800708	33864880	ENSG00000153162	BMP6	6	7727030	7880334	0.013063	9.1329	2.95E-11	1.07E-05	0.0044057	0.5778	0.77438	rs10512472	2.80E-30	0	4.96E-30	0	Pancreas
ENSG00000092871	RFFL	17	33341759	33416338	ENSG00000158828	PINK1	1	20959948	20978004	0.012518	10.207	2.98E-11	1.08E-05	0.014613	1.6924	0.096239	rs9915021	5.07E-27	0	NA	NA	Adipose_Subcutaneous
ENSG00000092871	RFFL	17	33341759	33416338	ENSG00000158828	PINK1	1	20959948	20978004	0.012518	10.207	2.98E-11	1.08E-05	0.014613	1.6924	0.096239	rs9915021	5.07E-27	0	NA	NA	Artery_Tibial
ENSG00000177963	RIC8A	11	207511	215113	ENSG00000138798	EGF	4	110834040	110933422	0.011947	11.685	3.01E-11	1.09E-05	0.005914	0.67895	0.71042	rs11602954	3.92E-10	2.13E-05	7.55E-62	0	Adipose_Subcutaneous
ENSG00000177963	RIC8A	11	207511	215113	ENSG00000138798	EGF	4	110834040	110933422	0.011947	11.685	3.01E-11	1.09E-05	0.005914	0.67895	0.71042	rs11602954	3.92E-10	2.13E-05	7.55E-62	0	Artery_Aorta
ENSG00000177963	RIC8A	11	207511	215113	ENSG00000138798	EGF	4	110834040	110933422	0.011947	11.685	3.01E-11	1.09E-05	0.005914	0.67895	0.71042	rs11602954	3.92E-10	2.13E-05	7.55E-62	0	Artery_Tibial
ENSG00000177963	RIC8A	11	207511	215113	ENSG00000138798	EGF	4	110834040	110933422	0.011947	11.685	3.01E-11	1.09E-05	0.005914	0.67895	0.71042	rs11602954	3.92E-10	2.13E-05	7.55E-62	0	Brain_Caudate_basal_ganglia
ENSG00000177963	RIC8A	11	207511	215113	ENSG00000138798	EGF	4	110834040	110933422	0.011947	11.685	3.01E-11	1.09E-05	0.005914	0.67895	0.71042	rs11602954	3.92E-10	2.13E-05	7.55E-62	0	Brain_Frontal_Cortex_BA9
ENSG00000177963	RIC8A	11	207511	215113	ENSG00000138798	EGF	4	110834040	110933422	0.011947	11.685	3.01E-11	1.09E-05	0.005914	0.67895	0.71042	rs11602954	3.92E-10	2.13E-05	7.55E-62	0	Colon_Sigmoid
ENSG00000177963	RIC8A	11	207511	215113	ENSG00000138798	EGF	4	110834040	110933422	0.011947	11.685	3.01E-11	1.09E-05	0.005914	0.67895	0.71042	rs11602954	3.92E-10	2.13E-05	7.55E-62	0	Colon_Transverse
ENSG00000177963	RIC8A	11	207511	215113	ENSG00000138798	EGF	4	110834040	110933422	0.011947	11.685	3.01E-11	1.09E-05	0.005914	0.67895	0.71042	rs11602954	3.92E-10	2.13E-05	7.55E-62	0	Esophagus_Gastroesophageal_Junction
ENSG00000177963	RIC8A	11	207511	215113	ENSG00000138798	EGF	4	110834040	110933422	0.011947	11.685	3.01E-11	1.09E-05	0.005914	0.67895	0.71042	rs11602954	3.92E-10	2.13E-05	7.55E-62	0	Esophagus_Mucosa
ENSG00000177963	RIC8A	11	207511	215113	ENSG00000138798	EGF	4	110834040	110933422	0.011947	11.685	3.01E-11	1.09E-05	0.005914	0.67895	0.71042	rs11602954	3.92E-10	2.13E-05	7.55E-62	0	Esophagus_Muscularis
ENSG00000177963	RIC8A	11	207511	215113	ENSG00000138798	EGF	4	110834040	110933422	0.011947	11.685	3.01E-11	1.09E-05	0.005914	0.67895	0.71042	rs11602954	3.92E-10	2.13E-05	7.55E-62	0	Lung
ENSG00000177963	RIC8A	11	207511	215113	ENSG00000138798	EGF	4	110834040	110933422	0.011947	11.685	3.01E-11	1.09E-05	0.005914	0.67895	0.71042	rs11602954	3.92E-10	2.13E-05	7.55E-62	0	Muscle_Skeletal
ENSG00000177963	RIC8A	11	207511	215113	ENSG00000138798	EGF	4	110834040	110933422	0.011947	11.685	3.01E-11	1.09E-05	0.005914	0.67895	0.71042	rs11602954	3.92E-10	2.13E-05	7.55E-62	0	Nerve_Tibial
ENSG00000177963	RIC8A	11	207511	215113	ENSG00000138798	EGF	4	110834040	110933422	0.011947	11.685	3.01E-11	1.09E-05	0.005914	0.67895	0.71042	rs11602954	3.92E-10	2.13E-05	7.55E-62	0	Skin_Not_Sun_Exposed_Suprapubic
ENSG00000177963	RIC8A	11	207511	215113	ENSG00000138798	EGF	4	110834040	110933422	0.011947	11.685	3.01E-11	1.09E-05	0.005914	0.67895	0.71042	rs11602954	3.92E-10	2.13E-05	7.55E-62	0	Skin_Sun_Exposed_Lower_leg
ENSG00000177963	RIC8A	11	207511	215113	ENSG00000138798	EGF	4	110834040	110933422	0.011947	11.685	3.01E-11	1.09E-05	0.005914	0.67895	0.71042	rs11602954	3.92E-10	2.13E-05	7.55E-62	0	Thyroid
ENSG00000177963	RIC8A	11	207511	215113	ENSG00000138798	EGF	4	110834040	110933422	0.011947	11.685	3.01E-11	1.09E-05	0.005914	0.67895	0.71042	rs17655730	3.08E-11	0	5.10E-36	0	Adipose_Subcutaneous
ENSG00000177963	RIC8A	11	207511	215113	ENSG00000138798	EGF	4	110834040	110933422	0.011947	11.685	3.01E-11	1.09E-05	0.005914	0.67895	0.71042	rs17655730	3.08E-11	0	5.10E-36	0	Esophagus_Mucosa
ENSG00000177963	RIC8A	11	207511	215113	ENSG00000138798	EGF	4	110834040	110933422	0.011947	11.685	3.01E-11	1.09E-05	0.005914	0.67895	0.71042	rs17655730	3.08E-11	0	5.10E-36	0	Esophagus_Muscularis
ENSG00000177963	RIC8A	11	207511	215113	ENSG00000138798	EGF	4	110834040	110933422	0.011947	11.685	3.01E-11	1.09E-05	0.005914	0.67895	0.71042	rs17655730	3.08E-11	0	5.10E-36	0	Heart_Left_Ventricle
ENSG00000177963	RIC8A	11	207511	215113	ENSG00000138798	EGF	4	110834040	110933422	0.011947	11.685	3.01E-11	1.09E-05	0.005914	0.67895	0.71042	rs17655730	3.08E-11	0	5.10E-36	0	Ovary
ENSG00000177963	RIC8A	11	207511	215113	ENSG00000138798	EGF	4	110834040	110933422	0.011947	11.685	3.01E-11	1.09E-05	0.005914	0.67895	0.71042	rs17655730	3.08E-11	0	5.10E-36	0	Pancreas
ENSG00000177963	RIC8A	11	207511	215113	ENSG00000138798	EGF	4	110834040	110933422	0.011947	11.685	3.01E-11	1.09E-05	0.005914	0.67895	0.71042	rs17655730	3.08E-11	0	5.10E-36	0	Skin_Not_Sun_Exposed_Suprapubic
ENSG00000177963	RIC8A	11	207511	215113	ENSG00000138798	EGF	4	110834040	110933422	0.011947	11.685	3.01E-11	1.09E-05	0.005914	0.67895	0.71042	rs505404	2.92E-13	0	3.66E-47	0	Artery_Aorta
ENSG00000177963	RIC8A	11	207511	215113	ENSG00000138798	EGF	4	110834040	110933422	0.011947	11.685	3.01E-11	1.09E-05	0.005914	0.67895	0.71042	rs505404	2.92E-13	0	3.66E-47	0	Thyroid
ENSG00000172123	SLFN12	17	33738079	33760302	ENSG00000103148	NPRL3	16	138697	188859	0.016828	5.5022	3.07E-11	1.11E-05	0.011846	1.8282	0.090639	rs225245	1.20E-79	0	NA	NA	Cells_EBV-transformed_lymphocytes
ENSG00000172123	SLFN12	17	33738079	33760302	ENSG00000103148	NPRL3	16	138697	188859	0.016828	5.5022	3.07E-11	1.11E-05	0.011846	1.8282	0.090639	rs9915021	1.32E-25	0	NA	NA	Breast_Mammary_Tissue
ENSG00000142082	SIRT3	11	215458	236431	ENSG00000138798	EGF	4	110834040	110933422	0.010651	17.348	3.35E-11	1.20E-05	0.0028181	0.64787	0.62848	rs11602954	3.92E-10	2.13E-05	1.39E-80	0	Adipose_Subcutaneous
ENSG00000142082	SIRT3	11	215458	236431	ENSG00000138798	EGF	4	110834040	110933422	0.010651	17.348	3.35E-11	1.20E-05	0.0028181	0.64787	0.62848	rs11602954	3.92E-10	2.13E-05	1.39E-80	0	Artery_Tibial
ENSG00000142082	SIRT3	11	215458	236431	ENSG00000138798	EGF	4	110834040	110933422	0.010651	17.348	3.35E-11	1.20E-05	0.0028181	0.64787	0.62848	rs11602954	3.92E-10	2.13E-05	1.39E-80	0	Breast_Mammary_Tissue
ENSG00000142082	SIRT3	11	215458	236431	ENSG00000138798	EGF	4	110834040	110933422	0.010651	17.348	3.35E-11	1.20E-05	0.0028181	0.64787	0.62848	rs11602954	3.92E-10	2.13E-05	1.39E-80	0	Cells_Transformed_fibroblasts
ENSG00000142082	SIRT3	11	215458	236431	ENSG00000138798	EGF	4	110834040	110933422	0.010651	17.348	3.35E-11	1.20E-05	0.0028181	0.64787	0.62848	rs11602954	3.92E-10	2.13E-05	1.39E-80	0	Nerve_Tibial
ENSG00000142082	SIRT3	11	215458	236431	ENSG00000138798	EGF	4	110834040	110933422	0.010651	17.348	3.35E-11	1.20E-05	0.0028181	0.64787	0.62848	rs505404	2.92E-13	0	1.05E-94	0	Adrenal_Gland
ENSG00000142082	SIRT3	11	215458	236431	ENSG00000138798	EGF	4	110834040	110933422	0.010651	17.348	3.35E-11	1.20E-05	0.0028181	0.64787	0.62848	rs505404	2.92E-13	0	1.05E-94	0	Artery_Aorta
ENSG00000142082	SIRT3	11	215458	236431	ENSG00000138798	EGF	4	110834040	110933422	0.010651	17.348	3.35E-11	1.20E-05	0.0028181	0.64787	0.62848	rs505404	2.92E-13	0	1.05E-94	0	Lung
ENSG00000142082	SIRT3	11	215458	236431	ENSG00000138798	EGF	4	110834040	110933422	0.010651	17.348	3.35E-11	1.20E-05	0.0028181	0.64787	0.62848	rs505404	2.92E-13	0	1.05E-94	0	Thyroid
ENSG00000154760	SLFN13	17	33762115	33775856	ENSG00000144677	CTDSPL	3	37903451	38025960	0.014975	6.67	3.44E-11	1.22E-05	0.030671	2.3969	0.0047161	rs225245	2.72E-40	0	NA	NA	Liver
ENSG00000154760	SLFN13	17	33762115	33775856	ENSG00000144677	CTDSPL	3	37903451	38025960	0.014975	6.67	3.44E-11	1.22E-05	0.030671	2.3969	0.0047161	rs8082605	1.17E-78	0	2.08E-19	0	Artery_Tibial
ENSG00000154760	SLFN13	17	33762115	33775856	ENSG00000144677	CTDSPL	3	37903451	38025960	0.014975	6.67	3.44E-11	1.22E-05	0.030671	2.3969	0.0047161	rs8082605	1.17E-78	0	2.08E-19	0	Esophagus_Mucosa
ENSG00000154760	SLFN13	17	33762115	33775856	ENSG00000144677	CTDSPL	3	37903451	38025960	0.014975	6.67	3.44E-11	1.22E-05	0.030671	2.3969	0.0047161	rs8082605	1.17E-78	0	2.08E-19	0	Nerve_Tibial
ENSG00000154760	SLFN13	17	33762115	33775856	ENSG00000144677	CTDSPL	3	37903451	38025960	0.014975	6.67	3.44E-11	1.22E-05	0.030671	2.3969	0.0047161	rs8082605	1.17E-78	0	2.08E-19	0	Pituitary
ENSG00000154760	SLFN13	17	33762115	33775856	ENSG00000144677	CTDSPL	3	37903451	38025960	0.014975	6.67	3.44E-11	1.22E-05	0.030671	2.3969	0.0047161	rs10512472	7.69E-165	0	6.34E-38	0	Uterus
ENSG00000142102	ATHL1	11	289138	296107	ENSG00000259207	ITGB3	17	45387505	45389182	0.018403	4.7541	3.83E-11	1.35E-05	0.0010488	0.48244	0.61743	rs11602954	1.90E-32	0	8.23E-62	0	Thyroid
ENSG00000142102	ATHL1	11	289138	296107	ENSG00000259207	ITGB3	17	45387505	45389182	0.018403	4.7541	3.83E-11	1.35E-05	0.0010488	0.48244	0.61743	rs17655730	6.92E-36	0	1.10E-69	0	Muscle_Skeletal
ENSG00000142102	ATHL1	11	289138	296107	ENSG00000259207	ITGB3	17	45387505	45389182	0.018403	4.7541	3.83E-11	1.35E-05	0.0010488	0.48244	0.61743	rs505404	5.02E-41	0	3.83E-63	0	Lung
ENSG00000099849	RASSF7	11	560404	564021	ENSG00000049323	LTBP1	2	33172039	33624576	0.012892	9.0114	4.35E-11	1.51E-05	0.022829	3.0504	0.0035339	rs505404	1.77E-15	0	NA	NA	Brain_Frontal_Cortex_BA9
ENSG00000174885	NLRP6	11	278365	285359	ENSG00000144677	CTDSPL	3	37903451	38025960	0.01289	9.0101	4.36E-11	1.52E-05	0.032482	3.402	0.00040077	rs11602954	1.10E-48	0	2.12E-70	0	Nerve_Tibial
ENSG00000174885	NLRP6	11	278365	285359	ENSG00000144677	CTDSPL	3	37903451	38025960	0.01289	9.0101	4.36E-11	1.52E-05	0.032482	3.402	0.00040077	rs17655730	1.49E-41	0	5.43E-117	0	Artery_Aorta
ENSG00000174885	NLRP6	11	278365	285359	ENSG00000144677	CTDSPL	3	37903451	38025960	0.01289	9.0101	4.36E-11	1.52E-05	0.032482	3.402	0.00040077	rs17655730	1.49E-41	0	5.43E-117	0	Nerve_Tibial
ENSG00000174885	NLRP6	11	278365	285359	ENSG00000144677	CTDSPL	3	37903451	38025960	0.01289	9.0101	4.36E-11	1.52E-05	0.032482	3.402	0.00040077	rs17655730	1.49E-41	0	5.43E-117	0	Skin_Sun_Exposed_Lower_leg
ENSG00000174885	NLRP6	11	278365	285359	ENSG00000144677	CTDSPL	3	37903451	38025960	0.01289	9.0101	4.36E-11	1.52E-05	0.032482	3.402	0.00040077	rs505404	1.75E-49	0	2.65E-88	0	Nerve_Tibial
ENSG00000174885	NLRP6	11	278365	285359	ENSG00000144677	CTDSPL	3	37903451	38025960	0.01289	9.0101	4.36E-11	1.52E-05	0.032482	3.402	0.00040077	rs505404	1.75E-49	0	2.65E-88	0	Prostate
ENSG00000132139	GAS2L2	17	34071530	34079897	ENSG00000022840	RNF10	12	120971283	121015397	0.013399	8.1977	4.45E-11	1.54E-05	0.017504	2.0333	0.039932	rs9915021	1.07E-57	0	NA	NA	Small_Intestine_Terminal_Ileum
ENSG00000172057	ORMDL3	17	38077294	38083854	ENSG00000162551	ALPL	1	21835858	21904905	0.011779	11.519	4.44E-11	1.54E-05	0.011743	1.8121	0.093712	rs7216389	1.61E-08	0.00020309	3.2717e-310	0	Adipose_Subcutaneous
ENSG00000172057	ORMDL3	17	38077294	38083854	ENSG00000162551	ALPL	1	21835858	21904905	0.011779	11.519	4.44E-11	1.54E-05	0.011743	1.8121	0.093712	rs4794820	1.24E-10	0	3.2717e-310	0	Spleen
ENSG00000172057	ORMDL3	17	38077294	38083854	ENSG00000162551	ALPL	1	21835858	21904905	0.011779	11.519	4.44E-11	1.54E-05	0.011743	1.8121	0.093712	rs2872507	7.00E-08	0.00087554	3.2717e-310	0	Whole_Blood
ENSG00000172057	ORMDL3	17	38077294	38083854	ENSG00000162551	ALPL	1	21835858	21904905	0.011779	11.519	4.44E-11	1.54E-05	0.011743	1.8121	0.093712	rs8067378	2.19E-07	0.00212414	3.2717e-310	0	Cells_EBV-transformed_lymphocytes
ENSG00000172057	ORMDL3	17	38077294	38083854	ENSG00000162551	ALPL	1	21835858	21904905	0.011779	11.519	4.44E-11	1.54E-05	0.011743	1.8121	0.093712	rs8067378	2.19E-07	0.00212414	3.2717e-310	0	Spleen

ENSG00000172057	ORMDL3	17	38077294	38083854	ENSG00000162551	ALPL	1	21835858	21904905	0.011779	11.519	4.44E-11	1.54E-05	0.011743	1.8121	0.093712	rs8067378	2.19E-07	0.00212414	3.2717e-310	0	Whole_Blood
ENSG00000172057	ORMDL3	17	38077294	38083854	ENSG00000162551	ALPL	1	21835858	21904905	0.011779	11.519	4.44E-11	1.54E-05	0.011743	1.8121	0.093712	rs9303277	3.21E-07	0.00291306	3.2717e-310	0	Cells_EBV-transformed_lymphocytes
ENSG00000172057	ORMDL3	17	38077294	38083854	ENSG00000162551	ALPL	1	21835858	21904905	0.011779	11.519	4.44E-11	1.54E-05	0.011743	1.8121	0.093712	rs9303277	3.21E-07	0.00291306	3.2717e-310	0	Skin_Sun_Exposed_Lower_leg
ENSG00000172057	ORMDL3	17	38077294	38083854	ENSG00000162551	ALPL	1	21835858	21904905	0.011779	11.519	4.44E-11	1.54E-05	0.011743	1.8121	0.093712	rs8069176	1.27E-09	5.83E-05	3.2717e-310	0	Whole_Blood
ENSG00000172057	ORMDL3	17	38077294	38083854	ENSG00000162551	ALPL	1	21835858	21904905	0.011779	11.519	4.44E-11	1.54E-05	0.011743	1.8121	0.093712	rs2290400	1.69E-08	0.00023328	3.2717e-310	0	Cells_EBV-transformed_lymphocytes
ENSG00000172057	ORMDL3	17	38077294	38083854	ENSG00000162551	ALPL	1	21835858	21904905	0.011779	11.519	4.44E-11	1.54E-05	0.011743	1.8121	0.093712	rs2290400	1.69E-08	0.00023328	3.2717e-310	0	Esophagus_Muscularis
ENSG00000172057	ORMDL3	17	38077294	38083854	ENSG00000162551	ALPL	1	21835858	21904905	0.011779	11.519	4.44E-11	1.54E-05	0.011743	1.8121	0.093712	rs2290400	1.69E-08	0.00023328	3.2717e-310	0	Lung
ENSG00000172057	ORMDL3	17	38077294	38083854	ENSG00000162551	ALPL	1	21835858	21904905	0.011779	11.519	4.44E-11	1.54E-05	0.011743	1.8121	0.093712	rs2290400	1.69E-08	0.00023328	3.2717e-310	0	Skin_Sun_Exposed_Lower_leg
ENSG00000163946	FAM208A	3	56658507	56717265	ENSG00000120885	CLU	8	27454434	27472548	0.013395	8.1953	4.48E-11	1.55E-05	0.030335	4.0848	0.00019958	rs12485738	4.93E-49	0	NA	NA	Esophagus_Mucosa
ENSG00000163946	FAM208A	3	56658507	56717265	ENSG00000120885	CLU	8	27454434	27472548	0.013395	8.1953	4.48E-11	1.55E-05	0.030335	4.0848	0.00019958	rs1354034	2.24E-91	0	NA	NA	Heart_Atrial_Appendage
ENSG00000108733	PEX12	17	33901814	33905882	ENSG00000017483	SLC38A5	X	48316920	48328644	0.011762	11.502	4.62E-11	1.60E-05	0.031756	6.0084	1.76E-05	rs9915021	6.46E-06	0.04076003	NA	NA	Brain_Caudate_basal_ganglia
ENSG00000108733	PEX12	17	33901814	33905882	ENSG00000017483	SLC38A5	X	48316920	48328644	0.011762	11.502	4.62E-11	1.60E-05	0.031756	6.0084	1.76E-05	rs9915021	6.46E-06	0.04076003	NA	NA	Breast_Mammary_Tissue
ENSG00000108733	PEX12	17	33901814	33905882	ENSG00000017483	SLC38A5	X	48316920	48328644	0.011762	11.502	4.62E-11	1.60E-05	0.031756	6.0084	1.76E-05	rs10512472	1.90E-13	0	1.06E-12	0	Brain_Caudate_basal_ganglia
ENSG00000154760	SLFN13	17	33762115	33775856	ENSG00000140564	FURIN	15	91411822	91426688	0.014804	6.5925	4.98E-11	1.71E-05	0.0072972	0.55683	0.87715	rs225245	2.51E-30	0	NA	NA	Liver
ENSG00000154760	SLFN13	17	33762115	33775856	ENSG00000140564	FURIN	15	91411822	91426688	0.014804	6.5925	4.98E-11	1.71E-05	0.0072972	0.55683	0.87715	rs8082605	2.70E-18	0	2.08E-19	0	Artery_Tibial
ENSG00000154760	SLFN13	17	33762115	33775856	ENSG00000140564	FURIN	15	91411822	91426688	0.014804	6.5925	4.98E-11	1.71E-05	0.0072972	0.55683	0.87715	rs8082605	2.70E-18	0	2.08E-19	0	Esophagus_Mucosa
ENSG00000154760	SLFN13	17	33762115	33775856	ENSG00000140564	FURIN	15	91411822	91426688	0.014804	6.5925	4.98E-11	1.71E-05	0.0072972	0.55683	0.87715	rs8082605	2.70E-18	0	2.08E-19	0	Nerve_Tibial
ENSG00000154760	SLFN13	17	33762115	33775856	ENSG00000140564	FURIN	15	91411822	91426688	0.014804	6.5925	4.98E-11	1.71E-05	0.0072972	0.55683	0.87715	rs8082605	2.70E-18	0	2.08E-19	0	Pituitary
ENSG00000154760	SLFN13	17	33762115	33775856	ENSG00000140564	FURIN	15	91411822	91426688	0.014804	6.5925	4.98E-11	1.71E-05	0.0072972	0.55683	0.87715	rs10512472	1.96E-14	0	6.34E-38	0	Uterus
ENSG00000132139	GAS2L2	17	34071530	34079897	ENSG00000164068	RNF123	3	49728563	49753910	0.013342	8.1626	5.04E-11	1.73E-05	0.015068	1.746	0.084212	rs9915021	2.27E-20	0	NA	NA	Small_Intestine_Terminal_Ileum
ENSG00000163946	FAM208A	3	56658507	56717265	ENSG00000205038	PKHD1L1	8	110374706	110542559	0.013323	8.1508	5.26E-11	1.80E-05	0.01243	1.6434	0.11964	rs12485738	1.94E-59	0	NA	NA	Esophagus_Mucosa
ENSG00000163946	FAM208A	3	56658507	56717265	ENSG00000205038	PKHD1L1	8	110374706	110542559	0.013323	8.1508	5.26E-11	1.80E-05	0.01243	1.6434	0.11964	rs1354034	6.01E-100	0	NA	NA	Heart_Atrial_Appendage
ENSG00000006125	AP2B1	17	33913918	34053436	ENSG00000130159	ECSIT	19	11618272	11639989	0.012791	8.9403	5.45E-11	1.86E-05	0.014343	2.2191	0.039269	rs10512472	1.20E-17	0	3.62E-14	0	Heart_Left_Ventricle
ENSG00000177963	RIC8A	11	207511	215113	ENSG00000172572	PDE3A	12	20522179	20837315	0.011671	11.412	5.72E-11	1.95E-05	NA	NA	NA	rs17655730	1.27E-06	0.0101111	5.10E-36	0	Adipose_Subcutaneous
ENSG00000177963	RIC8A	11	207511	215113	ENSG00000172572	PDE3A	12	20522179	20837315	0.011671	11.412	5.72E-11	1.95E-05	NA	NA	NA	rs17655730	1.27E-06	0.0101111	5.10E-36	0	Esophagus_Mucosa
ENSG00000177963	RIC8A	11	207511	215113	ENSG00000172572	PDE3A	12	20522179	20837315	0.011671	11.412	5.72E-11	1.95E-05	NA	NA	NA	rs17655730	1.27E-06	0.0101111	5.10E-36	0	Esophagus_Muscularis
ENSG00000177963	RIC8A	11	207511	215113	ENSG00000172572	PDE3A	12	20522179	20837315	0.011671	11.412	5.72E-11	1.95E-05	NA	NA	NA	rs17655730	1.27E-06	0.0101111	5.10E-36	0	Heart_Left_Ventricle
ENSG00000177963	RIC8A	11	207511	215113	ENSG00000172572	PDE3A	12	20522179	20837315	0.011671	11.412	5.72E-11	1.95E-05	NA	NA	NA	rs17655730	1.27E-06	0.0101111	5.10E-36	0	Ovary
ENSG00000177963	RIC8A	11	207511	215113	ENSG00000172572	PDE3A	12	20522179	20837315	0.011671	11.412	5.72E-11	1.95E-05	NA	NA	NA	rs17655730	1.27E-06	0.0101111	5.10E-36	0	Pancreas
ENSG00000177963	RIC8A	11	207511	215113	ENSG00000172572	PDE3A	12	20522179	20837315	0.011671	11.412	5.72E-11	1.95E-05	NA	NA	NA	rs17655730	1.27E-06	0.0101111	5.10E-36	0	Skin_Not_Sun_Exposed_Suprapubic
ENSG00000177963	RIC8A	11	207511	215113	ENSG00000172572	PDE3A	12	20522179	20837315	0.011671	11.412	5.72E-11	1.95E-05	NA	NA	NA	rs505404	3.08E-06	0.02212182	3.66E-47	0	Artery_Aorta
ENSG00000177963	RIC8A	11	207511	215113	ENSG00000172572	PDE3A	12	20522179	20837315	0.011671	11.412	5.72E-11	1.95E-05	NA	NA	NA	rs505404	3.08E-06	0.02212182	3.66E-47	0	Thyroid
ENSG00000168374	ARF4	3	57557090	57583947	ENSG00000035403	VCL	10	75757872	75879918	0.011654	11.395	5.94E-11	2.02E-05	0.012255	2.273	0.045506	rs17825630	8.88E-20	0	NA	NA	Artery_Tibial
ENSG00000168374	ARF4	3	57557090	57583947	ENSG00000035403	VCL	10	75757872	75879918	0.011654	11.395	5.94E-11	2.02E-05	0.012255	2.273	0.045506	rs17825630	8.88E-20	0	NA	NA	Heart_Atrial_Appendage
ENSG00000108733	PEX12	17	33901814	33905882	ENSG00000102145	GATA1	X	48644962	48652718	0.011642	11.384	6.10E-11	2.07E-05	0.01675	3.1209	0.0084324	rs10512472	1.87E-11	0	1.06E-12	0	Brain_Caudate_basal_ganglia
ENSG00000163946	FAM208A	3	56658507	56717265	ENSG00000101335	MYL9	20	35169887	35178228	0.013247	8.1036	6.23E-11	2.10E-05	0.033233	4.4885	6.28E-05	rs12485738	6.62E-243	0	NA	NA	Esophagus_Mucosa
ENSG00000163946	FAM208A	3	56658507	56717265	ENSG00000101335	MYL9	20	35169887	35178228	0.013247	8.1036	6.23E-11	2.10E-05	0.033233	4.4885	6.28E-05	rs1354034	3.27170000000001e-	0	NA	NA	Heart_Atrial_Appendage
ENSG00000205045	SLFN12L	17	33800708	33864880	ENSG00000140479	PCSK6	15	101840818	102065405	0.0127	8.8754	6.70E-11	2.25E-05	0.016746	2.2238	0.030339	rs8082605	1.51E-11	0	2.49E-31	0	Adipose_Subcutaneous
ENSG00000205045	SLFN12L	17	33800708	33864880	ENSG00000140479	PCSK6	15	101840818	102065405	0.0127	8.8754	6.70E-11	2.25E-05	0.016746	2.2238	0.030339	rs8082605	1.51E-11	0	2.49E-31	0	Adipose_Visceral_Omentum
ENSG00000205045	SLFN12L	17	33800708	33864880	ENSG00000140479	PCSK6	15	101840818	102065405	0.0127	8.8754	6.70E-11	2.25E-05	0.016746	2.2238	0.030339	rs8082605	1.51E-11	0	2.49E-31	0	Heart_Left_Ventricle
ENSG00000205045	SLFN12L	17	33800708	33864880	ENSG00000140479	PCSK6	15	101840818	102065405	0.0127	8.8754	6.70E-11	2.25E-05	0.016746	2.2238	0.030339	rs8082605	1.51E-11	0	2.49E-31	0	Nerve_Tibial
ENSG00000205045	SLFN12L	17	33800708	33864880	ENSG00000140479	PCSK6	15	101840818	102065405	0.0127	8.8754	6.70E-11	2.25E-05	0.016746	2.2238	0.030339	rs10512472	3.03E-22	0	4.96E-30	0	Pancreas
ENSG00000172660	TAF15	17	34136459	34174246	ENSG00000198959	TGM2	20	36756863	36794980	0.012163	9.9134	6.72E-11	2.26E-05	0.0056453	0.86579	0.51951	rs9915021	2.01E-09	5.61E-05	NA	NA	Skin_Not_Sun_Exposed_Suprapubic
ENSG00000108733	PEX12	17	33901814	33905882	ENSG00000101335	MYL9	20	35169887	35178228	0.011593	11.335	6.85E-11	2.29E-05	0.04265	8.1615	1.50E-07	rs9915021	1.60E-84	0	NA	NA	Brain_Caudate_basal_ganglia
ENSG00000108733	PEX12	17	33901814	33905882	ENSG00000101335	MYL9	20	35169887	35178228	0.011593	11.335	6.85E-11	2.29E-05	0.04265	8.1615	1.50E-07	rs9915021	1.60E-84	0	NA	NA	Breast_Mammary_Tissue
ENSG00000108733	PEX12	17	33901814	33905882	ENSG00000101335	MYL9	20	35169887	35178228	0.011593	11.335	6.85E-11	2.29E-05	0.04265	8.1615	1.50E-07	rs10512472	4.66E-133	0	1.06E-12	0	Brain_Caudate_basal_ganglia
ENSG00000205045	SLFN12L	17	33800708	33864880	ENSG00000102145	GATA1	X	48644962	48652718	0.012689	8.8681	6.86E-11	2.29E-05	0.018317	2.4363	0.017768	rs8082605	5.92E-08	0.00080711	2.49E-31	0	Adipose_Subcutaneous
ENSG00000205045	SLFN12L	17	33800708	33864880	ENSG00000102145	GATA1	X	48644962	48652718	0.012689	8.8681	6.86E-11	2.29E-05	0.018317	2.4363	0.017768	rs8082605	5.92E-08	0.00080711	2.49E-31	0	Adipose_Visceral_Omentum
ENSG00000205045	SLFN12L	17	33800708	33864880	ENSG00000102145	GATA1	X	48644962	48652718	0.012689	8.8681	6.86E-11	2.29E-05	0.018317	2.4363	0.017768	rs8082605	5.92E-08	0.00080711	2.49E-31	0	Heart_Left_Ventricle
ENSG00000205045	SLFN12L	17	33800708	33864880	ENSG00000102145	GATA1	X	48644962	48652718	0.012689	8.8681	6.86E-11	2.29E-05	0.018317	2.4363	0.017768	rs8082605	5.92E-08	0.00080711	2.49E-31	0	Nerve_Tibial
ENSG00000205045	SLFN12L	17	33800708	33864880	ENSG00000102145	GATA1	X	48644962	48652718	0.012689	8.8681	6.86E-11	2.29E-05	0.018317	2.4363	0.017768	rs10512472	1.87E-11	0	4.96E-30	0	Pancreas
ENSG00000092871	RFFL	17	33341759	33416338	ENSG00000167671	UBXN6	19	4446046	4457819	0.012101	9.8626	7.74E-11	2.56E-05	0.010697	1.234	0.2756	rs9915021	2.43E-46	0	NA	NA	Adipose_Subcutaneous
ENSG00000092871	RFFL	17	33341759	33416338	ENSG00000167671	UBXN6	19	4446046	4457819	0.012101	9.8626	7.74E-11	2.56E-05	0.010697	1.234	0.2756	rs9915021	2.43E-46	0	NA	NA	Artery_Tibial
ENSG00000108733	PEX12	17	33901814	33905882	ENSG00000130159	ECSIT	19	11618272	11639989	0.011531	11.274	7.90E-11	2.61E-05	0.011616	2.153	0.05723	rs9915021	9.36E-14	0	NA	NA	Brain_Caudate_basal_ganglia
ENSG00000108733	PEX12	17	33901814	33905882	ENSG00000130159	ECSIT	19	11618272	11639989	0.011531	11.274	7.90E-11	2.61E-05	0.011616	2.153	0.05723	rs9915021	9.36E-14	0	NA	NA	Breast_Mammary_Tissue
ENSG00000108733	PEX12	17	33901814	33905882	ENSG00000130159	ECSIT	19	11618272	11639989	0.011531	11.274	7.90E-11	2.61E-05	0.011616	2.153	0.05723	rs10512472	1.20E-17	0	1.06E-12	0	Brain_Caudate_basal_ganglia
ENSG00000163946	FAM208A	3	56658507	56717265	ENSG00000166086	JAM3	11	133938820	134018713	0.013131	8.0318	8.07E-11	2.66E-05	0.0067532	0.88777	0.51542	rs12485738	1.88E-208	0	NA	NA	Esophagus_Mucosa
ENSG00000163946	FAM208A	3	56658507	56717265	ENSG00000166086	JAM3	11	133938820	134018713	0.013131	8.0318	8.07E-11	2.66E-05	0.0067532	0.88777	0.51542	rs1354034	3.27170000000001e-	0	NA	NA	Heart_Atrial_Appendage
ENSG00000006125	AP2B1	17	33913918	34053436	ENSG00000104904	OAZ1	19	2271369	2273487	0.01261	8.8119	8.21E-11	2.70E-05	0.0082234	1.2645	0.27116	rs10512472	9.23E-18	0	3.62E-14	0	Heart_Left_Ventricle
ENSG00000141150	RASL10B	17	34058668	34070540	ENSG00000167671	UBXN6	19	4446046	4457819	0.013104	8.0148	8.58E-11	2.82E-05	0.028605	3.845	0.00039349	rs9915021	2.43E-46	0	NA	NA	Cells_Transformed_fibroblasts
ENSG00000205045	SLFN12L	17	33800708	33864880	ENSG00000141084	RANBP10	16	67757005	67840555	0.01256	8.7763	9.19E-11	3.00E-05	0.00645	0.84766	0.54788	rs8082605	4.35E-13	0	2.49E-31	0	Adipose_Subcutaneous
ENSG00000205045	SLFN12L	17	33800708	33864880	ENSG00000141084	RANBP10	16	67757005	67840555	0.01256	8.7763	9.19E-11	3.00E-05	0.00645	0.84766	0.54788	rs8082605	4.35E-13	0	2.49E-31	0	Adipose_Visceral_Omentum
ENSG00000205045	SLFN12L	17	33800708	33864880	ENSG00000141084	RANBP10	16	67757005	67840555	0.01256	8.7763	9.19E-11	3.00E-05	0.00645	0.84766	0.54788	rs8082605	4.35E-13	0	2.49E-31	0	Heart_Left_Ventricle
ENSG00000205045	SLFN12L	17	33800708	33864880	ENSG00000141084	RANBP10	16	67757005	67840555	0.01256	8.7763	9.19E-11	3.00E-05	0.00645	0.84766	0.54788	rs8082605	4.35E-13	0	2.49E-31	0	Nerve_Tibial
ENSG00000205045	SLFN12L	17	33800708	33864880	ENSG00000141084	RANBP10	16	67757005	67840555	0.01256	8.7763	9.19E-11	3.00E-05	0.00645	0.84766	0.54788	rs10512472	1.25E-08	0.00017572	4.96E-30	0	Pancreas
ENSG00000172660	TAF15	17	34136459	34174246	ENSG00000196961	AP2A1	19	50270225	50309510	0.012022	9.7973	9.27E-11	3.02E-05	0.0075048	1.1531	0.32945	rs9915021	1.56E-11	0	NA	NA	Skin_Not_Sun_Exposed_Suprapubic
ENSG00000084093	REST	4	57774075	57802010	ENSG00000104381	GDAP1	8	75233749	75401107	0.010867	13.274	9.31E-11	3.03E-05	0.035626	8.469	1.04E-06	rs17081935	1.23E-37	0	3.02E-09	2.60E-05	Colon_Sigmoid
ENSG00000084093	REST	4	57774075	57802010	ENSG00000104381	GDAP1	8	75233749	75401107	0.010867	13.274	9.31E-11	3.03E-05	0.035626	8.469	1.04E-06	rs17081935	1.23E-37	0	3.02E-09	2.60E-05	Muscle_Skeletal
ENSG00000172660	TAF15	17	34136459	34174246	ENSG00000164849	GPR146	7	1084212	1098897	0.011998	9.7778	9.79E-11	3.18E-05	0.0094232	1.4507	0.19227	rs9915021	1.21E-09	3.90E-05	NA	NA	Skin_Not_Sun_Exposed_Suprapubic
ENSG00000154760	SLFN13	17	33762115	33775856	ENSG00000204463	BAG6	6	31606805	31620170	0.014479	6.4456	1.00E-10	3.24E-05	0.042096	3.3289	9.36E-05	rs225245	2.41E-35	0	NA	NA	Liver
ENSG00000154760	SLFN13	17	33762115	33775856	ENSG00000204463	BAG6	6	31606805	31620170	0.014479	6.4456	1.00E-10	3.24E-05	0.042096	3.3289	9.36E-05	rs8082605	6.38E-11	0	2.08E-19	0	Artery_Tibial
ENSG00000154760	SLFN13	17	33762115	33775856	ENSG00000204463	BAG6	6	31606805	31620170	0.014479	6.4456	1.00E-10	3.24E-05	0.042096	3.3289	9.36E-05	rs8082605	6.38E-11	0	2.08E-19	0	Esophagus_Mucosa
ENSG00000154760	SLFN13	17	33762115	33775856	ENSG00000204463	BAG6	6	31606805	31620170	0.014479	6.4456	1.00E-10	3.24E-05	0.042096	3.3289	9.36E-05	rs8082605	6.38E-11	0	2.08E-19	0	Nerve_Tibial
ENSG00000154760	SLFN13	17	33762115	33775856	ENSG00000204463	BAG6	6	31606805	31620170	0.014479	6.4456	1.00E-10	3.24E-05	0.042096	3.3289	9.36E-05	rs8082605	6.38E-11	0	2.08E-19	0	Pituitary
ENSG00000154760	SLFN13	17	33762115	33775856	ENSG00000204463	BAG6	6	31606805	31620170	0.014479	6.4456	1.00E-10	3.24E-05	0.042096	3.3289	9.36E-05	rs10512472	7.83E-17	0	6.34E-38	0	Uterus
ENSG00000142102	ATHL1	11	289138	296107	ENSG00000122786	CALD1	7	134429003	134655479	0.017891	4.6193	1.07E-10	3.42E-05	0.0060712	2.8067	0.060919	rs11602954	3.38E-37	0	8.23E-62	0	Thyroid
ENSG00000142102	ATHL1	11	289138	296107	ENSG00000122786	CALD1	7	134429003	134655479	0.017891	4.6193	1.07E-10	3.42E-05	0.0060712	2.8067	0.060919	rs17655730	3.56E-40	0	1.10E-69	0	Muscle_Skeletal
ENSG00000142102	ATHL1	11	289138	296107	ENSG00000122786	CALD1	7	134429003	134655479	0.017891	4.6193	1.07E-10	3.42E-05	0.0060712	2.8067	0.060919	rs505404	1.84E-40	0	3.83E-63	0	Lung
ENSG00000172716	SLFN11	17	33677324	33700720	ENSG00000158828	PINK1	1	20959948	20978004	0.014807	6.0433	1.32E-10	4.17E-05	0.011655	0.89328	0.55347	rs225245	1.45E-52	0	5.77E-07	0.001884469	Cells_Transformed_fibroblasts
ENSG00000172716	SLFN11	17	33677324	33700720	ENSG00000158828	PINK1	1	20959948	20978004	0.014807	6.0433	1.32E-10	4.17E-05	0.011655	0.89328	0.55347	rs8082605	2.28E-16	0	NA	NA	Adrenal_Gland
ENSG00000168374	ARF4	3	57557090	57583947	ENSG00000061918	GUCY1B3	4	156680144	156728743	0.011305	11.05	1.34E-10	4.21E-05	0.018291	3.4133	0.0046209	rs17825630	4.20E-07	0.00377502	NA	NA	Artery_Tibial
ENSG00000168374	ARF4	3	57557090	57583947	ENSG00000061918	GUCY1B3	4	156680144	156728743	0.011305	11.05	1.34E-10	4.21E-05	0.018291	3.4133	0.0046209	rs17825630	4.20E-07	0.00377502	NA	NA	Heart_Atrial_Appendage

ENSG00000006125	AP2B1	17	33913918	34053436	ENSG00000102145	GATA1	X	48644962	48652718	0.012363	8.6376	1.43E-10	4.47E-05	0.023502	3.6704	0.0013114	rs10512472	1.87E-11	0	3.62E-14	0	Heart_Left_Ventricle
ENSG00000006125	AP2B1	17	33913918	34053436	ENSG00000090674	MCOLN1	19	7587512	7595387	0.012363	8.6371	1.43E-10	4.47E-05	0.005334	0.8178	0.55614	rs10512472	3.45E-14	0	3.62E-14	0	Heart_Left_Ventricle
ENSG00000177963	RIC8A	11	207511	215113	ENSG00000061918	GUCY1B3	4	156680144	156728743	0.011259	11.004	1.49E-10	4.63E-05	0.015691	1.8193	0.069923	rs11602954	8.40E-28	0	7.55E-62	0	Adipose_Subcutaneous
ENSG00000177963	RIC8A	11	207511	215113	ENSG00000061918	GUCY1B3	4	156680144	156728743	0.011259	11.004	1.49E-10	4.63E-05	0.015691	1.8193	0.069923	rs11602954	8.40E-28	0	7.55E-62	0	Artery_Aorta
ENSG00000177963	RIC8A	11	207511	215113	ENSG00000061918	GUCY1B3	4	156680144	156728743	0.011259	11.004	1.49E-10	4.63E-05	0.015691	1.8193	0.069923	rs11602954	8.40E-28	0	7.55E-62	0	Artery_Tibial
ENSG00000177963	RIC8A	11	207511	215113	ENSG00000061918	GUCY1B3	4	156680144	156728743	0.011259	11.004	1.49E-10	4.63E-05	0.015691	1.8193	0.069923	rs11602954	8.40E-28	0	7.55E-62	0	Brain_Caudate_basal_ganglia
ENSG00000177963	RIC8A	11	207511	215113	ENSG00000061918	GUCY1B3	4	156680144	156728743	0.011259	11.004	1.49E-10	4.63E-05	0.015691	1.8193	0.069923	rs11602954	8.40E-28	0	7.55E-62	0	Brain_Frontal_Cortex_BA9
ENSG00000177963	RIC8A	11	207511	215113	ENSG00000061918	GUCY1B3	4	156680144	156728743	0.011259	11.004	1.49E-10	4.63E-05	0.015691	1.8193	0.069923	rs11602954	8.40E-28	0	7.55E-62	0	Colon_Sigmoid
ENSG00000177963	RIC8A	11	207511	215113	ENSG00000061918	GUCY1B3	4	156680144	156728743	0.011259	11.004	1.49E-10	4.63E-05	0.015691	1.8193	0.069923	rs11602954	8.40E-28	0	7.55E-62	0	Colon_Transverse
ENSG00000177963	RIC8A	11	207511	215113	ENSG00000061918	GUCY1B3	4	156680144	156728743	0.011259	11.004	1.49E-10	4.63E-05	0.015691	1.8193	0.069923	rs11602954	8.40E-28	0	7.55E-62	0	Esophagus_Gastroesophageal_Junction
ENSG00000177963	RIC8A	11	207511	215113	ENSG00000061918	GUCY1B3	4	156680144	156728743	0.011259	11.004	1.49E-10	4.63E-05	0.015691	1.8193	0.069923	rs11602954	8.40E-28	0	7.55E-62	0	Esophagus_Mucosa
ENSG00000177963	RIC8A	11	207511	215113	ENSG00000061918	GUCY1B3	4	156680144	156728743	0.011259	11.004	1.49E-10	4.63E-05	0.015691	1.8193	0.069923	rs11602954	8.40E-28	0	7.55E-62	0	Esophagus_Muscularis
ENSG00000177963	RIC8A	11	207511	215113	ENSG00000061918	GUCY1B3	4	156680144	156728743	0.011259	11.004	1.49E-10	4.63E-05	0.015691	1.8193	0.069923	rs11602954	8.40E-28	0	7.55E-62	0	Lung
ENSG00000177963	RIC8A	11	207511	215113	ENSG00000061918	GUCY1B3	4	156680144	156728743	0.011259	11.004	1.49E-10	4.63E-05	0.015691	1.8193	0.069923	rs11602954	8.40E-28	0	7.55E-62	0	Muscle_Skeletal
ENSG00000177963	RIC8A	11	207511	215113	ENSG00000061918	GUCY1B3	4	156680144	156728743	0.011259	11.004	1.49E-10	4.63E-05	0.015691	1.8193	0.069923	rs11602954	8.40E-28	0	7.55E-62	0	Nerve_Tibial
ENSG00000177963	RIC8A	11	207511	215113	ENSG00000061918	GUCY1B3	4	156680144	156728743	0.011259	11.004	1.49E-10	4.63E-05	0.015691	1.8193	0.069923	rs11602954	8.40E-28	0	7.55E-62	0	Skin_Not_Sun_Exposed_Suprapubic
ENSG00000177963	RIC8A	11	207511	215113	ENSG00000061918	GUCY1B3	4	156680144	156728743	0.011259	11.004	1.49E-10	4.63E-05	0.015691	1.8193	0.069923	rs11602954	8.40E-28	0	7.55E-62	0	Skin_Sun_Exposed_Lower_leg
ENSG00000177963	RIC8A	11	207511	215113	ENSG00000061918	GUCY1B3	4	156680144	156728743	0.011259	11.004	1.49E-10	4.63E-05	0.015691	1.8193	0.069923	rs11602954	8.40E-28	0	7.55E-62	0	Thyroid
ENSG00000177963	RIC8A	11	207511	215113	ENSG00000061918	GUCY1B3	4	156680144	156728743	0.011259	11.004	1.49E-10	4.63E-05	0.015691	1.8193	0.069923	rs17655730	5.01E-29	0	5.10E-36	0	Adipose_Subcutaneous
ENSG00000177963	RIC8A	11	207511	215113	ENSG00000061918	GUCY1B3	4	156680144	156728743	0.011259	11.004	1.49E-10	4.63E-05	0.015691	1.8193	0.069923	rs17655730	5.01E-29	0	5.10E-36	0	Esophagus_Mucosa
ENSG00000177963	RIC8A	11	207511	215113	ENSG00000061918	GUCY1B3	4	156680144	156728743	0.011259	11.004	1.49E-10	4.63E-05	0.015691	1.8193	0.069923	rs17655730	5.01E-29	0	5.10E-36	0	Esophagus_Muscularis
ENSG00000177963	RIC8A	11	207511	215113	ENSG00000061918	GUCY1B3	4	156680144	156728743	0.011259	11.004	1.49E-10	4.63E-05	0.015691	1.8193	0.069923	rs17655730	5.01E-29	0	5.10E-36	0	Heart_Left_Ventricle
ENSG00000177963	RIC8A	11	207511	215113	ENSG00000061918	GUCY1B3	4	156680144	156728743	0.011259	11.004	1.49E-10	4.63E-05	0.015691	1.8193	0.069923	rs17655730	5.01E-29	0	5.10E-36	0	Ovary
ENSG00000177963	RIC8A	11	207511	215113	ENSG00000061918	GUCY1B3	4	156680144	156728743	0.011259	11.004	1.49E-10	4.63E-05	0.015691	1.8193	0.069923	rs17655730	5.01E-29	0	5.10E-36	0	Pancreas
ENSG00000177963	RIC8A	11	207511	215113	ENSG00000061918	GUCY1B3	4	156680144	156728743	0.011259	11.004	1.49E-10	4.63E-05	0.015691	1.8193	0.069923	rs17655730	5.01E-29	0	5.10E-36	0	Skin_Not_Sun_Exposed_Suprapubic
ENSG00000177963	RIC8A	11	207511	215113	ENSG00000061918	GUCY1B3	4	156680144	156728743	0.011259	11.004	1.49E-10	4.63E-05	0.015691	1.8193	0.069923	rs505404	1.76E-34	0	3.66E-47	0	Artery_Aorta
ENSG00000177963	RIC8A	11	207511	215113	ENSG00000061918	GUCY1B3	4	156680144	156728743	0.011259	11.004	1.49E-10	4.63E-05	0.015691	1.8193	0.069923	rs505404	1.76E-34	0	3.66E-47	0	Thyroid
ENSG00000177963	RIC8A	11	207511	215113	ENSG00000151693	ASAP2	2	9346894	9541525	0.011248	10.993	1.52E-10	4.74E-05	0.0074723	0.85919	0.55069	rs17655730	6.61E-07	0.00575008	5.10E-36	0	Adipose_Subcutaneous
ENSG00000177963	RIC8A	11	207511	215113	ENSG00000151693	ASAP2	2	9346894	9541525	0.011248	10.993	1.52E-10	4.74E-05	0.0074723	0.85919	0.55069	rs17655730	6.61E-07	0.00575008	5.10E-36	0	Esophagus_Mucosa
ENSG00000177963	RIC8A	11	207511	215113	ENSG00000151693	ASAP2	2	9346894	9541525	0.011248	10.993	1.52E-10	4.74E-05	0.0074723	0.85919	0.55069	rs17655730	6.61E-07	0.00575008	5.10E-36	0	Esophagus_Muscularis
ENSG00000177963	RIC8A	11	207511	215113	ENSG00000151693	ASAP2	2	9346894	9541525	0.011248	10.993	1.52E-10	4.74E-05	0.0074723	0.85919	0.55069	rs17655730	6.61E-07	0.00575008	5.10E-36	0	Heart_Left_Ventricle
ENSG00000177963	RIC8A	11	207511	215113	ENSG00000151693	ASAP2	2	9346894	9541525	0.011248	10.993	1.52E-10	4.74E-05	0.0074723	0.85919	0.55069	rs17655730	6.61E-07	0.00575008	5.10E-36	0	Ovary
ENSG00000177963	RIC8A	11	207511	215113	ENSG00000151693	ASAP2	2	9346894	9541525	0.011248	10.993	1.52E-10	4.74E-05	0.0074723	0.85919	0.55069	rs17655730	6.61E-07	0.00575008	5.10E-36	0	Pancreas
ENSG00000177963	RIC8A	11	207511	215113	ENSG00000151693	ASAP2	2	9346894	9541525	0.011248	10.993	1.52E-10	4.74E-05	0.0074723	0.85919	0.55069	rs17655730	6.61E-07	0.00575008	5.10E-36	0	Skin_Not_Sun_Exposed_Suprapubic
ENSG00000177963	RIC8A	11	207511	215113	ENSG00000151693	ASAP2	2	9346894	9541525	0.011248	10.993	1.52E-10	4.74E-05	0.0074723	0.85919	0.55069	rs505404	3.11E-08	0.0004087	3.66E-47	0	Artery_Aorta
ENSG00000177963	RIC8A	11	207511	215113	ENSG00000151693	ASAP2	2	9346894	9541525	0.011248	10.993	1.52E-10	4.74E-05	0.0074723	0.85919	0.55069	rs505404	3.11E-08	0.0004087	3.66E-47	0	Thyroid
ENSG00000108733	PEX12	17	33901814	33905882	ENSG00000196961	AP2A1	19	50270225	50309510	0.011241	10.987	1.55E-10	4.81E-05	0.0023418	0.43002	0.82787	rs9915021	1.56E-11	0	NA	NA	Brain_Caudate_basal_ganglia
ENSG00000108733	PEX12	17	33901814	33905882	ENSG00000196961	AP2A1	19	50270225	50309510	0.011241	10.987	1.55E-10	4.81E-05	0.0023418	0.43002	0.82787	rs9915021	1.56E-11	0	NA	NA	Breast_Mammary_Tissue
ENSG00000108733	PEX12	17	33901814	33905882	ENSG00000196961	AP2A1	19	50270225	50309510	0.011241	10.987	1.55E-10	4.81E-05	0.0023418	0.43002	0.82787	rs10512472	3.81E-08	0.00049957	1.06E-12	0	Brain_Caudate_basal_ganglia
ENSG00000196071	OR2L13	1	248100493	248264224	ENSG00000166086	JAM3	11	133938820	134018713	0.013308	7.2355	1.63E-10	5.05E-05	0.010779	1.1042	0.35684	rs3811445	8.19E-246	0	NA	NA	Nerve_Tibial
ENSG00000196071	OR2L13	1	248100493	248264224	ENSG00000166086	JAM3	11	133938820	134018713	0.013308	7.2355	1.63E-10	5.05E-05	0.010779	1.1042	0.35684	rs1339847	1.52E-41	0	NA	NA	Brain_Caudate_basal_ganglia
ENSG00000196071	OR2L13	1	248100493	248264224	ENSG00000166086	JAM3	11	133938820	134018713	0.013308	7.2355	1.63E-10	5.05E-05	0.010779	1.1042	0.35684	rs1339847	1.52E-41	0	NA	NA	Brain_Cortex
ENSG00000006125	AP2B1	17	33913918	34053436	ENSG00000085733	CTTN	11	70244510	70282690	0.012293	8.5875	1.68E-10	5.19E-05	0.0064859	0.99556	0.4269	rs10512472	4.18E-19	0	3.62E-14	0	Heart_Left_Ventricle
ENSG00000108733	PEX12	17	33901814	33905882	ENSG00000124491	F13A1	6	6144318	6321246	0.011183	10.93	1.77E-10	5.45E-05	0.0053819	0.9913	0.42186	rs9915021	1.01E-11	0	NA	NA	Brain_Caudate_basal_ganglia
ENSG00000108733	PEX12	17	33901814	33905882	ENSG00000124491	F13A1	6	6144318	6321246	0.011183	10.93	1.77E-10	5.45E-05	0.0053819	0.9913	0.42186	rs9915021	1.01E-11	0	NA	NA	Breast_Mammary_Tissue
ENSG00000108733	PEX12	17	33901814	33905882	ENSG00000124491	F13A1	6	6144318	6321246	0.011183	10.93	1.77E-10	5.45E-05	0.0053819	0.9913	0.42186	rs10512472	3.64E-32	0	1.06E-12	0	Brain_Caudate_basal_ganglia
ENSG00000108733	PEX12	17	33901814	33905882	ENSG00000204463	BAG6	6	31606805	31620170	0.011173	10.92	1.81E-10	5.57E-05	0.036329	6.9064	2.43E-06	rs9915021	2.40E-12	0	NA	NA	Brain_Caudate_basal_ganglia
ENSG00000108733	PEX12	17	33901814	33905882	ENSG00000204463	BAG6	6	31606805	31620170	0.011173	10.92	1.81E-10	5.57E-05	0.036329	6.9064	2.43E-06	rs9915021	2.40E-12	0	NA	NA	Breast_Mammary_Tissue
ENSG00000108733	PEX12	17	33901814	33905882	ENSG00000204463	BAG6	6	31606805	31620170	0.011173	10.92	1.81E-10	5.57E-05	0.036329	6.9064	2.43E-06	rs10512472	7.83E-17	0	1.06E-12	0	Brain_Caudate_basal_ganglia
ENSG00000172716	SLFN11	17	33677324	33700720	ENSG00000164068	RNF123	3	49728563	49753910	0.014657	5.9808	1.82E-10	5.58E-05	0.01308	1.004	0.44293	rs225245	4.45E-47	0	5.77E-07	0.001884469	Cells_Transformed_fibroblasts
ENSG00000172716	SLFN11	17	33677324	33700720	ENSG00000164068	RNF123	3	49728563	49753910	0.014657	5.9808	1.82E-10	5.58E-05	0.01308	1.004	0.44293	rs8082605	5.08E-22	0	NA	NA	Adrenal_Gland
ENSG00000168374	ARF4	3	57557090	57583947	ENSG00000185245	GP1BA	17	4835592	4838325	0.011166	10.913	1.84E-10	5.64E-05	0.028782	5.4292	6.25E-05	rs17825630	2.78E-14	0	NA	NA	Artery_Tibial
ENSG00000168374	ARF4	3	57557090	57583947	ENSG00000185245	GP1BA	17	4835592	4838325	0.011166	10.913	1.84E-10	5.64E-05	0.028782	5.4292	6.25E-05	rs17825630	2.78E-14	0	NA	NA	Heart_Atrial_Appendage
ENSG00000188895	MSL1	17	38278551	38291643	ENSG00000196663	TECPR2	14	102829300	102968818	0.014182	6.3115	1.90E-10	5.79E-05	0.016539	5.146	0.0015642	rs3894194	7.36E-09	0.0001505	NA	NA	Spleen
ENSG00000188895	MSL1	17	38278551	38291643	ENSG00000196663	TECPR2	14	102829300	102968818	0.014182	6.3115	1.90E-10	5.79E-05	0.016539	5.146	0.0015642	rs7212938	6.67E-07	0.0058136	NA	NA	Thyroid
ENSG00000188895	MSL1	17	38278551	38291643	ENSG00000196663	TECPR2	14	102829300	102968818	0.014182	6.3115	1.90E-10	5.79E-05	0.016539	5.146	0.0015642	rs8078723	1.05E-21	0	NA	NA	Cells_Transformed_fibroblasts
ENSG00000188895	MSL1	17	38278551	38291643	ENSG00000196663	TECPR2	14	102829300	102968818	0.014182	6.3115	1.90E-10	5.79E-05	0.016539	5.146	0.0015642	rs4794820	8.93E-09	0.00016458	1.11E-05	0.031069922	Pancreas
ENSG00000108278	ZNHIT3	17	34842473	34849850	ENSG00000158856	DMTN	8	21906506	21940038	0.011134	10.881	1.98E-10	6.03E-05	NA	NA	NA	rs8082605	2.91E-102	0	NA	NA	Stomach
ENSG00000177951	BET1L	11	167784	207428	ENSG00000172572	PDE3A	12	20522179	20837315	0.012719	7.7765	2.02E-10	6.13E-05	NA	NA	NA	rs17655730	1.27E-06	0.0101111	2.96E-166	0	Prostate
ENSG00000177951	BET1L	11	167784	207428	ENSG00000172572	PDE3A	12	20522179	20837315	0.012719	7.7765	2.02E-10	6.13E-05	NA	NA	NA	rs505404	3.08E-06	0.02212182	3.73E-187	0	Small_Intestine_Terminal_Ileum
ENSG00000177951	BET1L	11	167784	207428	ENSG00000172572	PDE3A	12	20522179	20837315	0.012719	7.7765	2.02E-10	6.13E-05	NA	NA	NA	rs505404	3.08E-06	0.02212182	3.73E-187	0	Whole_Blood
ENSG00000177963	RIC8A	11	207511	215113	ENSG00000095303	PTGS1	9	125132824	125157982	0.011121	10.868	2.04E-10	6.19E-05	0.010286	1.1861	0.30416	rs11602954	3.11E-14	0	7.55E-62	0	Adipose_Subcutaneous
ENSG00000177963	RIC8A	11	207511	215113	ENSG00000095303	PTGS1	9	125132824	125157982	0.011121	10.868	2.04E-10	6.19E-05	0.010286	1.1861	0.30416	rs11602954	3.11E-14	0	7.55E-62	0	Artery_Aorta
ENSG00000177963	RIC8A	11	207511	215113	ENSG00000095303	PTGS1	9	125132824	125157982	0.011121	10.868	2.04E-10	6.19E-05	0.010286	1.1861	0.30416	rs11602954	3.11E-14	0	7.55E-62	0	Artery_Tibial
ENSG00000177963	RIC8A	11	207511	215113	ENSG00000095303	PTGS1	9	125132824	125157982	0.011121	10.868	2.04E-10	6.19E-05	0.010286	1.1861	0.30416	rs11602954	3.11E-14	0	7.55E-62	0	Brain_Caudate_basal_ganglia
ENSG00000177963	RIC8A	11	207511	215113	ENSG00000095303	PTGS1	9	125132824	125157982	0.011121	10.868	2.04E-10	6.19E-05	0.010286	1.1861	0.30416	rs11602954	3.11E-14	0	7.55E-62	0	Brain_Frontal_Cortex_BA9
ENSG00000177963	RIC8A	11	207511	215113	ENSG00000095303	PTGS1	9	125132824	125157982	0.011121	10.868	2.04E-10	6.19E-05	0.010286	1.1861	0.30416	rs11602954	3.11E-14	0	7.55E-62	0	Colon_Sigmoid
ENSG00000177963	RIC8A	11	207511	215113	ENSG00000095303	PTGS1	9	125132824	125157982	0.011121	10.868	2.04E-10	6.19E-05	0.010286	1.1861	0.30416	rs11602954	3.11E-14	0	7.55E-62	0	Colon_Transverse
ENSG00000177963	RIC8A	11	207511	215113	ENSG00000095303	PTGS1	9	125132824	125157982	0.011121	10.868	2.04E-10	6.19E-05	0.010286	1.1861	0.30416	rs11602954	3.11E-14	0	7.55E-62	0	Esophagus_Gastroesophageal_Junction
ENSG00000177963	RIC8A	11	207511	215113	ENSG00000095303	PTGS1	9	125132824	125157982	0.011121	10.868	2.04E-10	6.19E-05	0.010286	1.1861	0.30416	rs11602954	3.11E-14	0	7.55E-62	0	Esophagus_Mucosa
ENSG00000177963	RIC8A	11	207511	215113	ENSG00000095303	PTGS1	9	125132824	125157982	0.011121	10.868	2.04E-10	6.19E-05	0.010286	1.1861	0.30416	rs11602954	3.11E-14	0	7.55E-62	0	Esophagus_Muscularis
ENSG00000177963	RIC8A	11	207511	215113	ENSG00000095303	PTGS1	9	125132824	125157982	0.011121	10.868	2.04E-10	6.19E-05	0.010286	1.1861	0.30416	rs11602954	3.11E-14	0	7.55E-62	0	Lung
ENSG00000177963	RIC8A	11	207511	215113	ENSG00000095303	PTGS1	9	125132824	125157982	0.011121	10.868	2.04E-10	6.19E-05	0.010286	1.1861	0.30416	rs11602954	3.11E-14	0	7.55E-62	0	Muscle_Skeletal
ENSG00000177963	RIC8A	11	207511	215113	ENSG00000095303	PTGS1	9	125132824	125157982	0.011121	10.868	2.04E-10	6.19E-05	0.010286	1.1861	0.30416	rs11602954	3.11E-14	0	7.55E-62	0	Nerve_Tibial
ENSG00000177963	RIC8A	11	207511	215113	ENSG00000095303	PTGS1	9	125132824	125157982	0.011121	10.868	2.04E-10	6.19E-05	0.010286	1.1861	0.30416	rs11602954	3.11E-14	0	7.55E-62	0	Skin_Not_Sun_Exposed_Suprapubic
ENSG00000177963	RIC8A	11	207511	215113	ENSG00000095303	PTGS1	9	125132824	125157982	0.011121	10.868	2.04E-10	6.19E-05	0.010286	1.1861	0.30416	rs11602954	3.11E-14	0	7.55E-62	0	Skin_Sun_Exposed_Lower_leg
ENSG00000177963	RIC8A	11	207511	215113	ENSG00000095303	PTGS1	9	125132824	125157982	0.011121	10.868	2.04E-10	6.19E-05	0.010286	1.1861	0.30416	rs11602954	3.11E-14	0	7.55E-62	0	Thyroid
ENSG00000177963	RIC8A	11	207511	215113	ENSG00000095303	PTGS1	9	125132824	125157982	0.011121	10.868	2.04E-10	6.19E-05	0.010286	1.1861	0.30416	rs17655730	1.75E-16	0	5.10E-36	0	Adipose_Subcutaneous
ENSG00000177963	RIC8A	11	207511	215113	ENSG00000095303	PTGS1	9	125132824	125157982	0.011121	10.868	2.04E-10	6.19E-05	0.010286	1.1861	0.30416	rs17655730	1.75E-16	0	5.10E-36	0	Esophagus_Mucosa
ENSG00000177963	RIC8A	11	207511	215113	ENSG00000095303	PTGS1	9	125132824	125157982	0.011121	10.868	2.04E-10	6.19E-05	0.010286	1.1861	0.30416	rs17655730	1.75E-16	0	5.10E-36	0	Esophagus_Muscularis
ENSG00000177963	RIC8A	11	207511	215113	ENSG00000095303	PTGS1	9	125132824	125157982	0.011121	10.868	2.04E-10	6.19E-05	0.010286	1.1861	0.30416	rs17655730	1.75E-16	0	5.10E-36	0	Heart_Left_Ventricle
ENSG00000177963	RIC8A	11	207511	215113	ENSG00000095303	PTGS1	9	125132824	125157982	0.011121	10.868	2.04E-10	6.19E-05	0.010286	1.1861	0.30416	rs17655730	1.75E-16	0	5.10E-36	0	Ovary

ENSG00000177963	RIC8A	11	207511	215113	ENSG00000095303	PTGS1	9	125132824	125157982	0.011121	10.868	2.04E-10	6.19E-05	0.010286	1.1861	0.30416	rs17655730	1.75E-16	0	5.10E-36	0	Pancreas
ENSG00000177963	RIC8A	11	207511	215113	ENSG00000095303	PTGS1	9	125132824	125157982	0.011121	10.868	2.04E-10	6.19E-05	0.010286	1.1861	0.30416	rs17655730	1.75E-16	0	5.10E-36	0	Skin_Not_Sun_Exposed_Suprapubic
ENSG00000177963	RIC8A	11	207511	215113	ENSG00000095303	PTGS1	9	125132824	125157982	0.011121	10.868	2.04E-10	6.19E-05	0.010286	1.1861	0.30416	rs505404	4.38E-17	0	3.66E-47	0	Artery_Aorta
ENSG00000177963	RIC8A	11	207511	215113	ENSG00000095303	PTGS1	9	125132824	125157982	0.011121	10.868	2.04E-10	6.19E-05	0.010286	1.1861	0.30416	rs505404	4.38E-17	0	3.66E-47	0	Thyroid
ENSG00000167914	GSDMA	17	38119226	38134019	ENSG00000196663	TECPR2	14	102829300	102968818	0.0091712	22.377	2.12E-10	6.41E-05	0.008228	3.8121	0.022452	rs3859192	4.25E-17	0	6.22E-13	0	Adipose_Subcutaneous
ENSG00000167914	GSDMA	17	38119226	38134019	ENSG00000196663	TECPR2	14	102829300	102968818	0.0091712	22.377	2.12E-10	6.41E-05	0.008228	3.8121	0.022452	rs3859192	4.25E-17	0	6.22E-13	0	Adipose_Visceral_Omentum
ENSG00000167914	GSDMA	17	38119226	38134019	ENSG00000196663	TECPR2	14	102829300	102968818	0.0091712	22.377	2.12E-10	6.41E-05	0.008228	3.8121	0.022452	rs3859192	4.25E-17	0	6.22E-13	0	Adrenal_Gland
ENSG00000167914	GSDMA	17	38119226	38134019	ENSG00000196663	TECPR2	14	102829300	102968818	0.0091712	22.377	2.12E-10	6.41E-05	0.008228	3.8121	0.022452	rs3859192	4.25E-17	0	6.22E-13	0	Artery_Aorta
ENSG00000167914	GSDMA	17	38119226	38134019	ENSG00000196663	TECPR2	14	102829300	102968818	0.0091712	22.377	2.12E-10	6.41E-05	0.008228	3.8121	0.022452	rs3859192	4.25E-17	0	6.22E-13	0	Artery_Coronary
ENSG00000167914	GSDMA	17	38119226	38134019	ENSG00000196663	TECPR2	14	102829300	102968818	0.0091712	22.377	2.12E-10	6.41E-05	0.008228	3.8121	0.022452	rs3859192	4.25E-17	0	6.22E-13	0	Artery_Tibial
ENSG00000167914	GSDMA	17	38119226	38134019	ENSG00000196663	TECPR2	14	102829300	102968818	0.0091712	22.377	2.12E-10	6.41E-05	0.008228	3.8121	0.022452	rs3859192	4.25E-17	0	6.22E-13	0	Breast_Mammary_Tissue
ENSG00000167914	GSDMA	17	38119226	38134019	ENSG00000196663	TECPR2	14	102829300	102968818	0.0091712	22.377	2.12E-10	6.41E-05	0.008228	3.8121	0.022452	rs3859192	4.25E-17	0	6.22E-13	0	Cells_Transformed_fibroblasts
ENSG00000167914	GSDMA	17	38119226	38134019	ENSG00000196663	TECPR2	14	102829300	102968818	0.0091712	22.377	2.12E-10	6.41E-05	0.008228	3.8121	0.022452	rs3859192	4.25E-17	0	6.22E-13	0	Colon_Sigmoid
ENSG00000167914	GSDMA	17	38119226	38134019	ENSG00000196663	TECPR2	14	102829300	102968818	0.0091712	22.377	2.12E-10	6.41E-05	0.008228	3.8121	0.022452	rs3859192	4.25E-17	0	6.22E-13	0	Colon_Transverse
ENSG00000167914	GSDMA	17	38119226	38134019	ENSG00000196663	TECPR2	14	102829300	102968818	0.0091712	22.377	2.12E-10	6.41E-05	0.008228	3.8121	0.022452	rs3859192	4.25E-17	0	6.22E-13	0	Esophagus_Gastroesophageal_Junction
ENSG00000167914	GSDMA	17	38119226	38134019	ENSG00000196663	TECPR2	14	102829300	102968818	0.0091712	22.377	2.12E-10	6.41E-05	0.008228	3.8121	0.022452	rs3859192	4.25E-17	0	6.22E-13	0	Esophagus_Muscularis
ENSG00000167914	GSDMA	17	38119226	38134019	ENSG00000196663	TECPR2	14	102829300	102968818	0.0091712	22.377	2.12E-10	6.41E-05	0.008228	3.8121	0.022452	rs3859192	4.25E-17	0	6.22E-13	0	Heart_Atrial_Appendage
ENSG00000167914	GSDMA	17	38119226	38134019	ENSG00000196663	TECPR2	14	102829300	102968818	0.0091712	22.377	2.12E-10	6.41E-05	0.008228	3.8121	0.022452	rs3859192	4.25E-17	0	6.22E-13	0	Heart_Left_Ventricle
ENSG00000167914	GSDMA	17	38119226	38134019	ENSG00000196663	TECPR2	14	102829300	102968818	0.0091712	22.377	2.12E-10	6.41E-05	0.008228	3.8121	0.022452	rs3859192	4.25E-17	0	6.22E-13	0	Liver
ENSG00000167914	GSDMA	17	38119226	38134019	ENSG00000196663	TECPR2	14	102829300	102968818	0.0091712	22.377	2.12E-10	6.41E-05	0.008228	3.8121	0.022452	rs3859192	4.25E-17	0	6.22E-13	0	Lung
ENSG00000167914	GSDMA	17	38119226	38134019	ENSG00000196663	TECPR2	14	102829300	102968818	0.0091712	22.377	2.12E-10	6.41E-05	0.008228	3.8121	0.022452	rs3859192	4.25E-17	0	6.22E-13	0	Muscle_Skeletal
ENSG00000167914	GSDMA	17	38119226	38134019	ENSG00000196663	TECPR2	14	102829300	102968818	0.0091712	22.377	2.12E-10	6.41E-05	0.008228	3.8121	0.022452	rs3859192	4.25E-17	0	6.22E-13	0	Nerve_Tibial
ENSG00000167914	GSDMA	17	38119226	38134019	ENSG00000196663	TECPR2	14	102829300	102968818	0.0091712	22.377	2.12E-10	6.41E-05	0.008228	3.8121	0.022452	rs3859192	4.25E-17	0	6.22E-13	0	Ovary
ENSG00000167914	GSDMA	17	38119226	38134019	ENSG00000196663	TECPR2	14	102829300	102968818	0.0091712	22.377	2.12E-10	6.41E-05	0.008228	3.8121	0.022452	rs3859192	4.25E-17	0	6.22E-13	0	Pancreas
ENSG00000167914	GSDMA	17	38119226	38134019	ENSG00000196663	TECPR2	14	102829300	102968818	0.0091712	22.377	2.12E-10	6.41E-05	0.008228	3.8121	0.022452	rs3859192	4.25E-17	0	6.22E-13	0	Small_Intestine_Terminal_Ileum
ENSG00000167914	GSDMA	17	38119226	38134019	ENSG00000196663	TECPR2	14	102829300	102968818	0.0091712	22.377	2.12E-10	6.41E-05	0.008228	3.8121	0.022452	rs3859192	4.25E-17	0	6.22E-13	0	Spleen
ENSG00000167914	GSDMA	17	38119226	38134019	ENSG00000196663	TECPR2	14	102829300	102968818	0.0091712	22.377	2.12E-10	6.41E-05	0.008228	3.8121	0.022452	rs3859192	4.25E-17	0	6.22E-13	0	Stomach
ENSG00000167914	GSDMA	17	38119226	38134019	ENSG00000196663	TECPR2	14	102829300	102968818	0.0091712	22.377	2.12E-10	6.41E-05	0.008228	3.8121	0.022452	rs3859192	4.25E-17	0	6.22E-13	0	Thyroid
ENSG00000167914	GSDMA	17	38119226	38134019	ENSG00000196663	TECPR2	14	102829300	102968818	0.0091712	22.377	2.12E-10	6.41E-05	0.008228	3.8121	0.022452	rs3859192	4.25E-17	0	6.22E-13	0	Whole_Blood
ENSG00000167914	GSDMA	17	38119226	38134019	ENSG00000196663	TECPR2	14	102829300	102968818	0.0091712	22.377	2.12E-10	6.41E-05	0.008228	3.8121	0.022452	rs17609240	2.64E-12	0	1.53E-144	0	Adipose_Subcutaneous
ENSG00000167914	GSDMA	17	38119226	38134019	ENSG00000196663	TECPR2	14	102829300	102968818	0.0091712	22.377	2.12E-10	6.41E-05	0.008228	3.8121	0.022452	rs17609240	2.64E-12	0	1.53E-144	0	Adipose_Visceral_Omentum
ENSG00000167914	GSDMA	17	38119226	38134019	ENSG00000196663	TECPR2	14	102829300	102968818	0.0091712	22.377	2.12E-10	6.41E-05	0.008228	3.8121	0.022452	rs17609240	2.64E-12	0	1.53E-144	0	Artery_Aorta
ENSG00000167914	GSDMA	17	38119226	38134019	ENSG00000196663	TECPR2	14	102829300	102968818	0.0091712	22.377	2.12E-10	6.41E-05	0.008228	3.8121	0.022452	rs17609240	2.64E-12	0	1.53E-144	0	Breast_Mammary_Tissue
ENSG00000167914	GSDMA	17	38119226	38134019	ENSG00000196663	TECPR2	14	102829300	102968818	0.0091712	22.377	2.12E-10	6.41E-05	0.008228	3.8121	0.022452	rs17609240	2.64E-12	0	1.53E-144	0	Colon_Sigmoid
ENSG00000167914	GSDMA	17	38119226	38134019	ENSG00000196663	TECPR2	14	102829300	102968818	0.0091712	22.377	2.12E-10	6.41E-05	0.008228	3.8121	0.022452	rs17609240	2.64E-12	0	1.53E-144	0	Esophagus_Gastroesophageal_Junction
ENSG00000167914	GSDMA	17	38119226	38134019	ENSG00000196663	TECPR2	14	102829300	102968818	0.0091712	22.377	2.12E-10	6.41E-05	0.008228	3.8121	0.022452	rs17609240	2.64E-12	0	1.53E-144	0	Esophagus_Muscularis
ENSG00000167914	GSDMA	17	38119226	38134019	ENSG00000196663	TECPR2	14	102829300	102968818	0.0091712	22.377	2.12E-10	6.41E-05	0.008228	3.8121	0.022452	rs17609240	2.64E-12	0	1.53E-144	0	Heart_Left_Ventricle
ENSG00000167914	GSDMA	17	38119226	38134019	ENSG00000196663	TECPR2	14	102829300	102968818	0.0091712	22.377	2.12E-10	6.41E-05	0.008228	3.8121	0.022452	rs17609240	2.64E-12	0	1.53E-144	0	Muscle_Skeletal
ENSG00000167914	GSDMA	17	38119226	38134019	ENSG00000196663	TECPR2	14	102829300	102968818	0.0091712	22.377	2.12E-10	6.41E-05	0.008228	3.8121	0.022452	rs3894194	7.36E-09	0.0001505	6.08E-29	0	Adipose_Subcutaneous
ENSG00000167914	GSDMA	17	38119226	38134019	ENSG00000196663	TECPR2	14	102829300	102968818	0.0091712	22.377	2.12E-10	6.41E-05	0.008228	3.8121	0.022452	rs3894194	7.36E-09	0.0001505	6.08E-29	0	Adipose_Visceral_Omentum
ENSG00000167914	GSDMA	17	38119226	38134019	ENSG00000196663	TECPR2	14	102829300	102968818	0.0091712	22.377	2.12E-10	6.41E-05	0.008228	3.8121	0.022452	rs3894194	7.36E-09	0.0001505	6.08E-29	0	Artery_Tibial
ENSG00000167914	GSDMA	17	38119226	38134019	ENSG00000196663	TECPR2	14	102829300	102968818	0.0091712	22.377	2.12E-10	6.41E-05	0.008228	3.8121	0.022452	rs3894194	7.36E-09	0.0001505	6.08E-29	0	Colon_Transverse
ENSG00000167914	GSDMA	17	38119226	38134019	ENSG00000196663	TECPR2	14	102829300	102968818	0.0091712	22.377	2.12E-10	6.41E-05	0.008228	3.8121	0.022452	rs3894194	7.36E-09	0.0001505	6.08E-29	0	Esophagus_Gastroesophageal_Junction
ENSG00000167914	GSDMA	17	38119226	38134019	ENSG00000196663	TECPR2	14	102829300	102968818	0.0091712	22.377	2.12E-10	6.41E-05	0.008228	3.8121	0.022452	rs3894194	7.36E-09	0.0001505	6.08E-29	0	Heart_Atrial_Appendage
ENSG00000167914	GSDMA	17	38119226	38134019	ENSG00000196663	TECPR2	14	102829300	102968818	0.0091712	22.377	2.12E-10	6.41E-05	0.008228	3.8121	0.022452	rs3894194	7.36E-09	0.0001505	6.08E-29	0	Muscle_Skeletal
ENSG00000167914	GSDMA	17	38119226	38134019	ENSG00000196663	TECPR2	14	102829300	102968818	0.0091712	22.377	2.12E-10	6.41E-05	0.008228	3.8121	0.022452	rs3894194	7.36E-09	0.0001505	6.08E-29	0	Whole_Blood
ENSG00000167914	GSDMA	17	38119226	38134019	ENSG00000196663	TECPR2	14	102829300	102968818	0.0091712	22.377	2.12E-10	6.41E-05	0.008228	3.8121	0.022452	rs7212938	6.67E-07	0.0058136	5.34E-44	0	Adipose_Subcutaneous
ENSG00000167914	GSDMA	17	38119226	38134019	ENSG00000196663	TECPR2	14	102829300	102968818	0.0091712	22.377	2.12E-10	6.41E-05	0.008228	3.8121	0.022452	rs7212938	6.67E-07	0.0058136	5.34E-44	0	Adipose_Visceral_Omentum
ENSG00000167914	GSDMA	17	38119226	38134019	ENSG00000196663	TECPR2	14	102829300	102968818	0.0091712	22.377	2.12E-10	6.41E-05	0.008228	3.8121	0.022452	rs7212938	6.67E-07	0.0058136	5.34E-44	0	Artery_Coronary
ENSG00000167914	GSDMA	17	38119226	38134019	ENSG00000196663	TECPR2	14	102829300	102968818	0.0091712	22.377	2.12E-10	6.41E-05	0.008228	3.8121	0.022452	rs7212938	6.67E-07	0.0058136	5.34E-44	0	Colon_Sigmoid
ENSG00000167914	GSDMA	17	38119226	38134019	ENSG00000196663	TECPR2	14	102829300	102968818	0.0091712	22.377	2.12E-10	6.41E-05	0.008228	3.8121	0.022452	rs7212938	6.67E-07	0.0058136	5.34E-44	0	Heart_Atrial_Appendage
ENSG00000167914	GSDMA	17	38119226	38134019	ENSG00000196663	TECPR2	14	102829300	102968818	0.0091712	22.377	2.12E-10	6.41E-05	0.008228	3.8121	0.022452	rs7212938	6.67E-07	0.0058136	5.34E-44	0	Heart_Left_Ventricle
ENSG00000167914	GSDMA	17	38119226	38134019	ENSG00000196663	TECPR2	14	102829300	102968818	0.0091712	22.377	2.12E-10	6.41E-05	0.008228	3.8121	0.022452	rs7212938	6.67E-07	0.0058136	5.34E-44	0	Thyroid
ENSG00000167914	GSDMA	17	38119226	38134019	ENSG00000196663	TECPR2	14	102829300	102968818	0.0091712	22.377	2.12E-10	6.41E-05	0.008228	3.8121	0.022452	rs7212938	6.67E-07	0.0058136	5.34E-44	0	Whole_Blood
ENSG00000167914	GSDMA	17	38119226	38134019	ENSG00000196663	TECPR2	14	102829300	102968818	0.0091712	22.377	2.12E-10	6.41E-05	0.008228	3.8121	0.022452	rs8078723	1.05E-21	0	6.97E-07	0.002194627	Breast_Mammary_Tissue
ENSG00000167914	GSDMA	17	38119226	38134019	ENSG00000196663	TECPR2	14	102829300	102968818	0.0091712	22.377	2.12E-10	6.41E-05	0.008228	3.8121	0.022452	rs8078723	1.05E-21	0	6.97E-07	0.002194627	Esophagus_Gastroesophageal_Junction
ENSG00000167914	GSDMA	17	38119226	38134019	ENSG00000196663	TECPR2	14	102829300	102968818	0.0091712	22.377	2.12E-10	6.41E-05	0.008228	3.8121	0.022452	rs4794820	8.93E-09	0.00016458	2.26E-66	0	Artery_Tibial
ENSG00000167914	GSDMA	17	38119226	38134019	ENSG00000196663	TECPR2	14	102829300	102968818	0.0091712	22.377	2.12E-10	6.41E-05	0.008228	3.8121	0.022452	rs4794820	8.93E-09	0.00016458	2.26E-66	0	Cells_Transformed_fibroblasts
ENSG00000167914	GSDMA	17	38119226	38134019	ENSG00000196663	TECPR2	14	102829300	102968818	0.0091712	22.377	2.12E-10	6.41E-05	0.008228	3.8121	0.022452	rs4794822	2.44E-21	0	3.02E-06	0.008756123	Breast_Mammary_Tissue
ENSG00000167914	GSDMA	17	38119226	38134019	ENSG00000196663	TECPR2	14	102829300	102968818	0.0091712	22.377	2.12E-10	6.41E-05	0.008228	3.8121	0.022452	rs4794822	2.44E-21	0	3.02E-06	0.008756123	Cells_Transformed_fibroblasts
ENSG00000167914	GSDMA	17	38119226	38134019	ENSG00000196663	TECPR2	14	102829300	102968818	0.0091712	22.377	2.12E-10	6.41E-05	0.008228	3.8121	0.022452	rs4794822	2.44E-21	0	3.02E-06	0.008756123	Esophagus_Gastroesophageal_Junction
ENSG00000167914	GSDMA	17	38119226	38134019	ENSG00000196663	TECPR2	14	102829300	102968818	0.0091712	22.377	2.12E-10	6.41E-05	0.008228	3.8121	0.022452	rs4794822	2.44E-21	0	3.02E-06	0.008756123	Esophagus_Muscularis
ENSG00000167914	GSDMA	17	38119226	38134019	ENSG00000196663	TECPR2	14	102829300	102968818	0.0091712	22.377	2.12E-10	6.41E-05	0.008228	3.8121	0.022452	rs4065321	1.47E-17	0	2.52E-12	0	Pancreas
ENSG00000205045	SLFN12L	17	33800708	33864880	ENSG00000160445	ZER1	9	131492065	131534693	0.012184	8.5107	2.14E-10	6.46E-05	0.0024163	0.31626	0.94685	rs8082605	3.84E-07	0.00341109	2.49E-31	0	Adipose_Subcutaneous
ENSG00000205045	SLFN12L	17	33800708	33864880	ENSG00000160445	ZER1	9	131492065	131534693	0.012184	8.5107	2.14E-10	6.46E-05	0.0024163	0.31626	0.94685	rs8082605	3.84E-07	0.00341109	2.49E-31	0	Adipose_Visceral_Omentum
ENSG00000205045	SLFN12L	17	33800708	33864880	ENSG00000160445	ZER1	9	131492065	131534693	0.012184	8.5107	2.14E-10	6.46E-05	0.0024163	0.31626	0.94685	rs8082605	3.84E-07	0.00341109	2.49E-31	0	Heart_Left_Ventricle
ENSG00000205045	SLFN12L	17	33800708	33864880	ENSG00000160445	ZER1	9	131492065	131534693	0.012184	8.5107	2.14E-10	6.46E-05	0.0024163	0.31626	0.94685	rs8082605	3.84E-07	0.00341109	2.49E-31	0	Nerve_Tibial
ENSG00000205045	SLFN12L	17	33800708	33864880	ENSG00000160445	ZER1	9	131492065	131534693	0.012184	8.5107	2.14E-10	6.46E-05	0.0024163	0.31626	0.94685	rs10512472	7.27E-06	0.04465132	4.96E-30	0	Pancreas
ENSG00000142102	ATHL1	11	289138	296107	ENSG00000138722	MMRN1	4	90800683	90875780	0.017533	4.5253	2.17E-10	6.52E-05	0.0018766	0.86394	0.42184	rs11602954	5.94E-65	0	8.23E-62	0	Thyroid
ENSG00000142102	ATHL1	11	289138	296107	ENSG00000138722	MMRN1	4	90800683	90875780	0.017533	4.5253	2.17E-10	6.52E-05	0.0018766	0.86394	0.42184	rs17655730	6.29E-64	0	1.10E-69	0	Muscle_Skeletal
ENSG00000142102	ATHL1	11	289138	296107	ENSG00000138722	MMRN1	4	90800683	90875780	0.017533	4.5253	2.17E-10	6.52E-05	0.0018766	0.86394	0.42184	rs7104764	2.04E-08	0.00024398	1.19E-71	0	Brain_Frontal_Cortex_BA9
ENSG00000142102	ATHL1	11	289138	296107	ENSG00000138722	MMRN1	4	90800683	90875780	0.017533	4.5253	2.17E-10	6.52E-05	0.0018766	0.86394	0.42184	rs505404	2.01E-74	0	3.83E-63	0	Lung
ENSG00000154760	SLFN13	17	33762115	33775856	ENSG00000160445	ZER1	9	131492065	131534693	0.014118	6.2828	2.17E-10	6.53E-05	0.015721	1.2099	0.27094	rs225245	4.35E-13	0	NA	NA	Liver
ENSG00000154760	SLFN13	17	33762115	33775856	ENSG00000160445	ZER1	9	131492065	131534693	0.014118	6.2828	2.17E-10	6.53E-05	0.015721	1.2099	0.27094	rs8082605	3.84E-07	0.00341109	2.08E-19	0	Artery_Tibial
ENSG00000154760	SLFN13	17	33762115	33775856	ENSG00000160445	ZER1	9	131492065	131534693	0.014118	6.2828	2.17E-10	6.53E-05	0.015721	1.2099	0.27094	rs8082605	3.84E-07	0.00341109	2.08E-19	0	Esophagus_Mucosa
ENSG00000154760	SLFN13	17	33762115	33775856	ENSG00000160445	ZER1	9	131492065	131534693	0.014118	6.2828	2.17E-10	6.53E-05	0.015721	1.2099	0.27094	rs8082605	3.84E-07	0.00341109	2.08E-19	0	Nerve_Tibial
ENSG00000154760	SLFN13	17	33762115	33775856	ENSG00000160445	ZER1	9	131492065	131534693	0.014118	6.2828	2.17E-10	6.53E-05	0.015721	1.2099	0.27094	rs8082605	3.84E-07	0.00341109	2.08E-19	0	Pituitary
ENSG00000154760	SLFN13	17	33762115	33775856	ENSG00000160445	ZER1	9	131492065	131534693	0.014118	6.2828	2.17E-10	6.53E-05	0.015721	1.2099	0.27094	rs10512472	7.27E-06	0.04465132	6.34E-38	0	Uterus
ENSG00000141741	MIEN1	17	37885409	37886014	ENSG00000162551	ALPL	1	21835858	21904905	0.011086	10.833	2.22E-10	6.63E-05	0.0087165	1.6109	0.15446	rs7212938	1.21E-12	0	NA	NA	Testis
ENSG00000132139	GAS2L2	17	34071530	34079897	ENSG00000118046	STK11	19	1189406	1226662	0.012671	7.7465	2.24E-10	6.70E-05	0.0071328	0.81987	0.58509	rs9915021	3.14E-08	0.00040834	NA	NA	Small_Intestine_Terminal_Ileum
ENSG00000172660	TAF15	17	34136459	34174246	ENSG00000167992	VWCE	11	61025762	61062896	0.011633	9.4764	2.25E-10	6.72E-05	0.031076	4.8911	6.22E-05	rs9915021	1.45E-41	0	NA	NA	Skin_Not_Sun_Exposed_Suprapubic
ENSG00000142082	SIRT3	11	215458	236431	ENSG00000204420	C6orf25	6	31691121	31694491	0.0098497	16.029	2.29E-10	6.81E-05	0.004828	1.1122	0.34938	rs11602954	2.23E-16	0	1.39E-80	0	Adipose_Subcutaneous

ENSG00000142082	SIRT3	11	215458	236431	ENSG00000204420	C6orf25	6	31691121	31694491	0.0098497	16.029	2.29E-10	6.81E-05	0.004828	1.1122	0.34938	rs11602954	2.23E-16	0	1.39E-80	0	Artery_Tibial
ENSG00000142082	SIRT3	11	215458	236431	ENSG00000204420	C6orf25	6	31691121	31694491	0.0098497	16.029	2.29E-10	6.81E-05	0.004828	1.1122	0.34938	rs11602954	2.23E-16	0	1.39E-80	0	Breast_Mammary_Tissue
ENSG00000142082	SIRT3	11	215458	236431	ENSG00000204420	C6orf25	6	31691121	31694491	0.0098497	16.029	2.29E-10	6.81E-05	0.004828	1.1122	0.34938	rs11602954	2.23E-16	0	1.39E-80	0	Cells_Transformed_fibroblasts
ENSG00000142082	SIRT3	11	215458	236431	ENSG00000204420	C6orf25	6	31691121	31694491	0.0098497	16.029	2.29E-10	6.81E-05	0.004828	1.1122	0.34938	rs11602954	2.23E-16	0	1.39E-80	0	Nerve_Tibial
ENSG00000142082	SIRT3	11	215458	236431	ENSG00000204420	C6orf25	6	31691121	31694491	0.0098497	16.029	2.29E-10	6.81E-05	0.004828	1.1122	0.34938	rs505404	9.44E-22	0	1.05E-94	0	Adrenal_Gland
ENSG00000142082	SIRT3	11	215458	236431	ENSG00000204420	C6orf25	6	31691121	31694491	0.0098497	16.029	2.29E-10	6.81E-05	0.004828	1.1122	0.34938	rs505404	9.44E-22	0	1.05E-94	0	Artery_Aorta
ENSG00000142082	SIRT3	11	215458	236431	ENSG00000204420	C6orf25	6	31691121	31694491	0.0098497	16.029	2.29E-10	6.81E-05	0.004828	1.1122	0.34938	rs505404	9.44E-22	0	1.05E-94	0	Lung
ENSG00000142082	SIRT3	11	215458	236431	ENSG00000204420	C6orf25	6	31691121	31694491	0.0098497	16.029	2.29E-10	6.81E-05	0.004828	1.1122	0.34938	rs505404	9.44E-22	0	1.05E-94	0	Thyroid
ENSG00000073605	GSDMB	17	38060848	38074903	ENSG00000162551	ALPL	1	21835858	21904905	0.010473	12.788	2.35E-10	6.99E-05	0.0015799	0.36277	0.8352	rs12946510	8.70E-07	0.00729639	3.2717e-310	0	Whole_Blood
ENSG00000073605	GSDMB	17	38060848	38074903	ENSG00000162551	ALPL	1	21835858	21904905	0.010473	12.788	2.35E-10	6.99E-05	0.0015799	0.36277	0.8352	rs3859192	3.20E-29	0	3.2717e-310	0	Adrenal_Gland
ENSG00000073605	GSDMB	17	38060848	38074903	ENSG00000162551	ALPL	1	21835858	21904905	0.010473	12.788	2.35E-10	6.99E-05	0.0015799	0.36277	0.8352	rs7216389	1.61E-08	0.00020309	3.2717e-310	0	Adrenal_Gland
ENSG00000073605	GSDMB	17	38060848	38074903	ENSG00000162551	ALPL	1	21835858	21904905	0.010473	12.788	2.35E-10	6.99E-05	0.0015799	0.36277	0.8352	rs7216389	1.61E-08	0.00020309	3.2717e-310	0	Colon_Transverse
ENSG00000073605	GSDMB	17	38060848	38074903	ENSG00000162551	ALPL	1	21835858	21904905	0.010473	12.788	2.35E-10	6.99E-05	0.0015799	0.36277	0.8352	rs7216389	1.61E-08	0.00020309	3.2717e-310	0	Esophagus_Mucosa
ENSG00000073605	GSDMB	17	38060848	38074903	ENSG00000162551	ALPL	1	21835858	21904905	0.010473	12.788	2.35E-10	6.99E-05	0.0015799	0.36277	0.8352	rs7216389	1.61E-08	0.00020309	3.2717e-310	0	Lung
ENSG00000073605	GSDMB	17	38060848	38074903	ENSG00000162551	ALPL	1	21835858	21904905	0.010473	12.788	2.35E-10	6.99E-05	0.0015799	0.36277	0.8352	rs7216389	1.61E-08	0.00020309	3.2717e-310	0	Small_Intestine_Terminal_Ileum
ENSG00000073605	GSDMB	17	38060848	38074903	ENSG00000162551	ALPL	1	21835858	21904905	0.010473	12.788	2.35E-10	6.99E-05	0.0015799	0.36277	0.8352	rs3894194	2.28E-14	0	3.2717e-310	0	Esophagus_Mucosa
ENSG00000073605	GSDMB	17	38060848	38074903	ENSG00000162551	ALPL	1	21835858	21904905	0.010473	12.788	2.35E-10	6.99E-05	0.0015799	0.36277	0.8352	rs3894194	2.28E-14	0	3.2717e-310	0	Ovary
ENSG00000073605	GSDMB	17	38060848	38074903	ENSG00000162551	ALPL	1	21835858	21904905	0.010473	12.788	2.35E-10	6.99E-05	0.0015799	0.36277	0.8352	rs7212938	1.21E-12	0	3.2717e-310	0	Colon_Transverse
ENSG00000073605	GSDMB	17	38060848	38074903	ENSG00000162551	ALPL	1	21835858	21904905	0.010473	12.788	2.35E-10	6.99E-05	0.0015799	0.36277	0.8352	rs4794820	1.24E-10	0	3.2717e-310	0	Spleen
ENSG00000073605	GSDMB	17	38060848	38074903	ENSG00000162551	ALPL	1	21835858	21904905	0.010473	12.788	2.35E-10	6.99E-05	0.0015799	0.36277	0.8352	rs4794820	1.24E-10	0	3.2717e-310	0	Whole_Blood
ENSG00000073605	GSDMB	17	38060848	38074903	ENSG00000162551	ALPL	1	21835858	21904905	0.010473	12.788	2.35E-10	6.99E-05	0.0015799	0.36277	0.8352	rs9303280	4.17E-09	0.00010539	3.2717e-310	0	Colon_Transverse
ENSG00000073605	GSDMB	17	38060848	38074903	ENSG00000162551	ALPL	1	21835858	21904905	0.010473	12.788	2.35E-10	6.99E-05	0.0015799	0.36277	0.8352	rs9303280	4.17E-09	0.00010539	3.2717e-310	0	Lung
ENSG00000073605	GSDMB	17	38060848	38074903	ENSG00000162551	ALPL	1	21835858	21904905	0.010473	12.788	2.35E-10	6.99E-05	0.0015799	0.36277	0.8352	rs9303280	4.17E-09	0.00010539	3.2717e-310	0	Small_Intestine_Terminal_Ileum
ENSG00000073605	GSDMB	17	38060848	38074903	ENSG00000162551	ALPL	1	21835858	21904905	0.010473	12.788	2.35E-10	6.99E-05	0.0015799	0.36277	0.8352	rs4795397	9.75E-08	0.00110548	3.2717e-310	0	Whole_Blood
ENSG00000073605	GSDMB	17	38060848	38074903	ENSG00000162551	ALPL	1	21835858	21904905	0.010473	12.788	2.35E-10	6.99E-05	0.0015799	0.36277	0.8352	rs8067378	2.19E-07	0.00212414	3.2717e-310	0	Cells_EBV-transformed_lymphocytes
ENSG00000073605	GSDMB	17	38060848	38074903	ENSG00000162551	ALPL	1	21835858	21904905	0.010473	12.788	2.35E-10	6.99E-05	0.0015799	0.36277	0.8352	rs8067378	2.19E-07	0.00212414	3.2717e-310	0	Cells_Transformed_fibroblasts
ENSG00000073605	GSDMB	17	38060848	38074903	ENSG00000162551	ALPL	1	21835858	21904905	0.010473	12.788	2.35E-10	6.99E-05	0.0015799	0.36277	0.8352	rs8067378	2.19E-07	0.00212414	3.2717e-310	0	Spleen
ENSG00000073605	GSDMB	17	38060848	38074903	ENSG00000162551	ALPL	1	21835858	21904905	0.010473	12.788	2.35E-10	6.99E-05	0.0015799	0.36277	0.8352	rs8067378	2.19E-07	0.00212414	3.2717e-310	0	Stomach
ENSG00000073605	GSDMB	17	38060848	38074903	ENSG00000162551	ALPL	1	21835858	21904905	0.010473	12.788	2.35E-10	6.99E-05	0.0015799	0.36277	0.8352	rs8067378	2.19E-07	0.00212414	3.2717e-310	0	Whole_Blood
ENSG00000073605	GSDMB	17	38060848	38074903	ENSG00000162551	ALPL	1	21835858	21904905	0.010473	12.788	2.35E-10	6.99E-05	0.0015799	0.36277	0.8352	rs9303277	3.21E-07	0.00291306	3.2717e-310	0	Cells_EBV-transformed_lymphocytes
ENSG00000073605	GSDMB	17	38060848	38074903	ENSG00000162551	ALPL	1	21835858	21904905	0.010473	12.788	2.35E-10	6.99E-05	0.0015799	0.36277	0.8352	rs9303277	3.21E-07	0.00291306	3.2717e-310	0	Stomach
ENSG00000073605	GSDMB	17	38060848	38074903	ENSG00000162551	ALPL	1	21835858	21904905	0.010473	12.788	2.35E-10	6.99E-05	0.0015799	0.36277	0.8352	rs9303277	3.21E-07	0.00291306	3.2717e-310	0	Whole_Blood
ENSG00000073605	GSDMB	17	38060848	38074903	ENSG00000162551	ALPL	1	21835858	21904905	0.010473	12.788	2.35E-10	6.99E-05	0.0015799	0.36277	0.8352	rs8069176	1.27E-09	5.83E-05	3.2717e-310	0	Colon_Transverse
ENSG00000073605	GSDMB	17	38060848	38074903	ENSG00000162551	ALPL	1	21835858	21904905	0.010473	12.788	2.35E-10	6.99E-05	0.0015799	0.36277	0.8352	rs2290400	1.69E-08	0.00023328	3.2717e-310	0	Cells_EBV-transformed_lymphocytes
ENSG00000073605	GSDMB	17	38060848	38074903	ENSG00000162551	ALPL	1	21835858	21904905	0.010473	12.788	2.35E-10	6.99E-05	0.0015799	0.36277	0.8352	rs2305480	6.03E-09	0.00013571	3.2717e-310	0	Small_Intestine_Terminal_Ileum
ENSG00000073605	GSDMB	17	38060848	38074903	ENSG00000162551	ALPL	1	21835858	21904905	0.010473	12.788	2.35E-10	6.99E-05	0.0015799	0.36277	0.8352	rs11078927	2.68E-09	7.31E-05	3.2717e-310	0	Small_Intestine_Terminal_Ileum
ENSG00000168374	ARF4	3	57557090	57583947	ENSG00000095303	PTGS1	9	125132824	125157982	0.011034	10.782	2.50E-10	7.41E-05	0.023837	4.4735	0.00049358	rs17825630	1.02E-08	0.00016263	NA	NA	Artery_Tibial
ENSG00000168374	ARF4	3	57557090	57583947	ENSG00000095303	PTGS1	9	125132824	125157982	0.011034	10.782	2.50E-10	7.41E-05	0.023837	4.4735	0.00049358	rs17825630	1.02E-08	0.00016263	NA	NA	Heart_Atrial_Appendage
ENSG00000205045	SLFN12L	17	33800708	33864880	ENSG00000171552	BCL2L1	20	30252255	30311792	0.012107	8.4559	2.55E-10	7.54E-05	0.0022468	0.29402	0.95636	rs8082605	3.39E-06	0.02394735	2.49E-31	0	Adipose_Subcutaneous
ENSG00000205045	SLFN12L	17	33800708	33864880	ENSG00000171552	BCL2L1	20	30252255	30311792	0.012107	8.4559	2.55E-10	7.54E-05	0.0022468	0.29402	0.95636	rs8082605	3.39E-06	0.02394735	2.49E-31	0	Adipose_Visceral_Omentum
ENSG00000205045	SLFN12L	17	33800708	33864880	ENSG00000171552	BCL2L1	20	30252255	30311792	0.012107	8.4559	2.55E-10	7.54E-05	0.0022468	0.29402	0.95636	rs8082605	3.39E-06	0.02394735	2.49E-31	0	Heart_Left_Ventricle
ENSG00000205045	SLFN12L	17	33800708	33864880	ENSG00000171552	BCL2L1	20	30252255	30311792	0.012107	8.4559	2.55E-10	7.54E-05	0.0022468	0.29402	0.95636	rs8082605	3.39E-06	0.02394735	2.49E-31	0	Nerve_Tibial
ENSG00000177963	RIC8A	11	207511	215113	ENSG00000144677	CTDSPL	3	37903451	38025960	0.011019	10.767	2.59E-10	7.64E-05	0.017815	2.07	0.036182	rs11602954	1.10E-48	0	7.55E-62	0	Adipose_Subcutaneous
ENSG00000177963	RIC8A	11	207511	215113	ENSG00000144677	CTDSPL	3	37903451	38025960	0.011019	10.767	2.59E-10	7.64E-05	0.017815	2.07	0.036182	rs11602954	1.10E-48	0	7.55E-62	0	Artery_Aorta
ENSG00000177963	RIC8A	11	207511	215113	ENSG00000144677	CTDSPL	3	37903451	38025960	0.011019	10.767	2.59E-10	7.64E-05	0.017815	2.07	0.036182	rs11602954	1.10E-48	0	7.55E-62	0	Artery_Tibial
ENSG00000177963	RIC8A	11	207511	215113	ENSG00000144677	CTDSPL	3	37903451	38025960	0.011019	10.767	2.59E-10	7.64E-05	0.017815	2.07	0.036182	rs11602954	1.10E-48	0	7.55E-62	0	Brain_Caudate_basal_ganglia
ENSG00000177963	RIC8A	11	207511	215113	ENSG00000144677	CTDSPL	3	37903451	38025960	0.011019	10.767	2.59E-10	7.64E-05	0.017815	2.07	0.036182	rs11602954	1.10E-48	0	7.55E-62	0	Brain_Frontal_Cortex_BA9
ENSG00000177963	RIC8A	11	207511	215113	ENSG00000144677	CTDSPL	3	37903451	38025960	0.011019	10.767	2.59E-10	7.64E-05	0.017815	2.07	0.036182	rs11602954	1.10E-48	0	7.55E-62	0	Colon_Sigmoid
ENSG00000177963	RIC8A	11	207511	215113	ENSG00000144677	CTDSPL	3	37903451	38025960	0.011019	10.767	2.59E-10	7.64E-05	0.017815	2.07	0.036182	rs11602954	1.10E-48	0	7.55E-62	0	Colon_Transverse
ENSG00000177963	RIC8A	11	207511	215113	ENSG00000144677	CTDSPL	3	37903451	38025960	0.011019	10.767	2.59E-10	7.64E-05	0.017815	2.07	0.036182	rs11602954	1.10E-48	0	7.55E-62	0	Esophagus_Gastroesophageal_Junction
ENSG00000177963	RIC8A	11	207511	215113	ENSG00000144677	CTDSPL	3	37903451	38025960	0.011019	10.767	2.59E-10	7.64E-05	0.017815	2.07	0.036182	rs11602954	1.10E-48	0	7.55E-62	0	Esophagus_Mucosa
ENSG00000177963	RIC8A	11	207511	215113	ENSG00000144677	CTDSPL	3	37903451	38025960	0.011019	10.767	2.59E-10	7.64E-05	0.017815	2.07	0.036182	rs11602954	1.10E-48	0	7.55E-62	0	Esophagus_Muscularis
ENSG00000177963	RIC8A	11	207511	215113	ENSG00000144677	CTDSPL	3	37903451	38025960	0.011019	10.767	2.59E-10	7.64E-05	0.017815	2.07	0.036182	rs11602954	1.10E-48	0	7.55E-62	0	Lung
ENSG00000177963	RIC8A	11	207511	215113	ENSG00000144677	CTDSPL	3	37903451	38025960	0.011019	10.767	2.59E-10	7.64E-05	0.017815	2.07	0.036182	rs11602954	1.10E-48	0	7.55E-62	0	Muscle_Skeletal
ENSG00000177963	RIC8A	11	207511	215113	ENSG00000144677	CTDSPL	3	37903451	38025960	0.011019	10.767	2.59E-10	7.64E-05	0.017815	2.07	0.036182	rs11602954	1.10E-48	0	7.55E-62	0	Nerve_Tibial
ENSG00000177963	RIC8A	11	207511	215113	ENSG00000144677	CTDSPL	3	37903451	38025960	0.011019	10.767	2.59E-10	7.64E-05	0.017815	2.07	0.036182	rs11602954	1.10E-48	0	7.55E-62	0	Skin_Not_Sun_Exposed_Suprapubic
ENSG00000177963	RIC8A	11	207511	215113	ENSG00000144677	CTDSPL	3	37903451	38025960	0.011019	10.767	2.59E-10	7.64E-05	0.017815	2.07	0.036182	rs11602954	1.10E-48	0	7.55E-62	0	Skin_Sun_Exposed_Lower_leg
ENSG00000177963	RIC8A	11	207511	215113	ENSG00000144677	CTDSPL	3	37903451	38025960	0.011019	10.767	2.59E-10	7.64E-05	0.017815	2.07	0.036182	rs11602954	1.10E-48	0	7.55E-62	0	Thyroid
ENSG00000177963	RIC8A	11	207511	215113	ENSG00000144677	CTDSPL	3	37903451	38025960	0.011019	10.767	2.59E-10	7.64E-05	0.017815	2.07	0.036182	rs17655730	1.49E-41	0	5.10E-36	0	Adipose_Subcutaneous
ENSG00000177963	RIC8A	11	207511	215113	ENSG00000144677	CTDSPL	3	37903451	38025960	0.011019	10.767	2.59E-10	7.64E-05	0.017815	2.07	0.036182	rs17655730	1.49E-41	0	5.10E-36	0	Esophagus_Mucosa
ENSG00000177963	RIC8A	11	207511	215113	ENSG00000144677	CTDSPL	3	37903451	38025960	0.011019	10.767	2.59E-10	7.64E-05	0.017815	2.07	0.036182	rs17655730	1.49E-41	0	5.10E-36	0	Esophagus_Muscularis
ENSG00000177963	RIC8A	11	207511	215113	ENSG00000144677	CTDSPL	3	37903451	38025960	0.011019	10.767	2.59E-10	7.64E-05	0.017815	2.07	0.036182	rs17655730	1.49E-41	0	5.10E-36	0	Heart_Left_Ventricle
ENSG00000177963	RIC8A	11	207511	215113	ENSG00000144677	CTDSPL	3	37903451	38025960	0.011019	10.767	2.59E-10	7.64E-05	0.017815	2.07	0.036182	rs17655730	1.49E-41	0	5.10E-36	0	Ovary
ENSG00000177963	RIC8A	11	207511	215113	ENSG00000144677	CTDSPL	3	37903451	38025960	0.011019	10.767	2.59E-10	7.64E-05	0.017815	2.07	0.036182	rs17655730	1.49E-41	0	5.10E-36	0	Pancreas
ENSG00000177963	RIC8A	11	207511	215113	ENSG00000144677	CTDSPL	3	37903451	38025960	0.011019	10.767	2.59E-10	7.64E-05	0.017815	2.07	0.036182	rs17655730	1.49E-41	0	5.10E-36	0	Skin_Not_Sun_Exposed_Suprapubic
ENSG00000177963	RIC8A	11	207511	215113	ENSG00000144677	CTDSPL	3	37903451	38025960	0.011019	10.767	2.59E-10	7.64E-05	0.017815	2.07	0.036182	rs505404	1.75E-49	0	3.66E-47	0	Artery_Aorta
ENSG00000177963	RIC8A	11	207511	215113	ENSG00000144677	CTDSPL	3	37903451	38025960	0.011019	10.767	2.59E-10	7.64E-05	0.017815	2.07	0.036182	rs505404	1.75E-49	0	3.66E-47	0	Thyroid
ENSG00000141150	RASL10B	17	34058668	34070540	ENSG00000029534	ANK1	8	41510739	41754280	0.012603	7.7044	2.61E-10	7.68E-05	0.0062211	0.81738	0.57286	rs9915021	6.17E-12	0	NA	NA	Cells_Transformed_fibroblasts
ENSG00000154760	SLFN13	17	33762115	33775856	ENSG00000184481	FOXO4	X	70316047	70323385	0.014007	6.2323	2.76E-10	8.07E-05	0.027631	2.1525	0.012184	rs225245	1.36E-06	0.01072981	NA	NA	Liver
ENSG00000172660	TAF15	17	34136459	34174246	ENSG00000204463	BAG6	6	31606805	31620170	0.011514	9.379	2.95E-10	8.58E-05	0.024712	3.8641	0.00081491	rs9915021	2.40E-12	0	NA	NA	Skin_Not_Sun_Exposed_Suprapubic
ENSG00000142082	SIRT3	11	215458	236431	ENSG00000144677	CTDSPL	3	37903451	38025960	0.0097378	15.845	2.99E-10	8.69E-05	0.006279	1.4485	0.21601	rs11602954	1.10E-48	0	1.39E-80	0	Adipose_Subcutaneous
ENSG00000142082	SIRT3	11	215458	236431	ENSG00000144677	CTDSPL	3	37903451	38025960	0.0097378	15.845	2.99E-10	8.69E-05	0.006279	1.4485	0.21601	rs11602954	1.10E-48	0	1.39E-80	0	Artery_Tibial
ENSG00000142082	SIRT3	11	215458	236431	ENSG00000144677	CTDSPL	3	37903451	38025960	0.0097378	15.845	2.99E-10	8.69E-05	0.006279	1.4485	0.21601	rs11602954	1.10E-48	0	1.39E-80	0	Breast_Mammary_Tissue
ENSG00000142082	SIRT3	11	215458	236431	ENSG00000144677	CTDSPL	3	37903451	38025960	0.0097378	15.845	2.99E-10	8.69E-05	0.006279	1.4485	0.21601	rs11602954	1.10E-48	0	1.39E-80	0	Cells_Transformed_fibroblasts
ENSG00000142082	SIRT3	11	215458	236431	ENSG00000144677	CTDSPL	3	37903451	38025960	0.0097378	15.845	2.99E-10	8.69E-05	0.006279	1.4485	0.21601	rs11602954	1.10E-48	0	1.39E-80	0	Nerve_Tibial
ENSG00000142082	SIRT3	11	215458	236431	ENSG00000144677	CTDSPL	3	37903451	38025960	0.0097378	15.845	2.99E-10	8.69E-05	0.006279	1.4485	0.21601	rs505404	1.75E-49	0	1.05E-94	0	Adrenal_Gland
ENSG00000142082	SIRT3	11	215458	236431	ENSG00000144677	CTDSPL	3	37903451	38025960	0.0097378	15.845	2.99E-10	8.69E-05	0.006279	1.4485	0.21601	rs505404	1.75E-49	0	1.05E-94	0	Artery_Aorta
ENSG00000142082	SIRT3	11	215458	236431	ENSG00000144677	CTDSPL	3	37903451	38025960	0.0097378	15.845	2.99E-10	8.69E-05	0.006279	1.4485	0.21601	rs505404	1.75E-49	0	1.05E-94	0	Lung
ENSG00000142082	SIRT3	11	215458	236431	ENSG00000144677	CTDSPL	3	37903451	38025960	0.0097378	15.845	2.99E-10	8.69E-05	0.006279	1.4485	0.21601	rs505404	1.75E-49	0	1.05E-94	0	Thyroid
ENSG00000142082	SIRT3	11	215458	236431	ENSG00000144677	CTDSPL	3	37903451	38025960	0.0097378	15.845	2.99E-10	8.69E-05	0.006279	1.4485	0.21601	rs3782123	4.11E-07	0.00366492	NA	NA	Brain_Frontal_Cortex_BA9
ENSG00000108733	PEX12	17	33901814	33905882	ENSG00000161911	TREML1	6	41117080	41122075	0.010956	10.705	2.99E-10	8.69E-05	0.010332	1.9126	0.089741	rs9915021	3.57E-22	0	NA	NA	Brain_Caudate_basal_ganglia
ENSG00000108733	PEX12	17	33901814	33905882	ENSG00000161911	TREML1	6	41117080	41122075	0.010956	10.705	2.99E-10	8.69E-05	0.010332	1.9126	0.089741	rs9915021	3.57E-22	0	NA	NA	Breast_Mammary_Tissue

ENSG00000108733	PEX12	17	33901814	33905882	ENSG00000161911	TREML1	6	41117080	41122075	0.010956	10.705	2.99E-10	8.69E-05	0.010332	1.9126	0.089741	rs10512472	2.34E-44	0	1.06E-12	0	Brain_Caudate_basal_ganglia
ENSG00000163946	FAM208A	3	56658507	56717265	ENSG00000113140	SPARC	5	151040657	151066726	0.012519	7.6528	3.14E-10	9.07E-05	0.015584	2.0671	0.044578	rs12485738	1.78E-153	0	NA	NA	Esophagus_Mucosa
ENSG00000163946	FAM208A	3	56658507	56717265	ENSG00000113140	SPARC	5	151040657	151066726	0.012519	7.6528	3.14E-10	9.07E-05	0.015584	2.0671	0.044578	rs1354034	4.72E-273	0	NA	NA	Heart_Atrial_Appendage
ENSG00000132139	GAS2L2	17	34071530	34079897	ENSG00000158828	PINK1	1	20959948	20978004	0.012518	7.6521	3.15E-10	9.08E-05	0.020433	2.3806	0.015345	rs9915021	5.07E-27	0	NA	NA	Small_Intestine_Terminal_Ileum
ENSG00000198093	ZNF649	19	52399725	52408293	ENSG00000164659	KIAA1324L	7	86506222	86689015	0.012507	7.6454	3.22E-10	9.26E-05	0.027692	3.2503	0.0011713	rs11667325	8.07E-10	2.02E-05	NA	NA	Vagina
ENSG00000162722	TRIM58	1	248020501	248031169	ENSG00000166086	JAM3	11	133938820	134018713	0.012508	7.6457	3.22E-10	9.26E-05	0.0060649	0.93055	0.47202	rs3811445	8.19E-246	0	3.86E-98	0	Esophagus_Muscularis
ENSG00000162722	TRIM58	1	248020501	248031169	ENSG00000166086	JAM3	11	133938820	134018713	0.012508	7.6457	3.22E-10	9.26E-05	0.0060649	0.93055	0.47202	rs1339847	1.52E-41	0	1.69E-13	0	Adipose_Visceral_Omentum
ENSG00000162722	TRIM58	1	248020501	248031169	ENSG00000166086	JAM3	11	133938820	134018713	0.012508	7.6457	3.22E-10	9.26E-05	0.0060649	0.93055	0.47202	rs1339847	1.52E-41	0	1.69E-13	0	Skin_Not_Sun_Exposed_Suprapubic
ENSG00000177951	BET1L	11	167784	207428	ENSG00000151693	ASAP2	2	9346894	9541525	0.012501	7.6417	3.26E-10	9.37E-05	0.010495	0.87742	0.56229	rs17655730	6.61E-07	0.00575008	2.96E-166	0	Prostate
ENSG00000177951	BET1L	11	167784	207428	ENSG00000151693	ASAP2	2	9346894	9541525	0.012501	7.6417	3.26E-10	9.37E-05	0.010495	0.87742	0.56229	rs505404	3.11E-08	0.0004087	3.73E-187	0	Small_Intestine_Terminal_Ileum
ENSG00000177951	BET1L	11	167784	207428	ENSG00000151693	ASAP2	2	9346894	9541525	0.012501	7.6417	3.26E-10	9.37E-05	0.010495	0.87742	0.56229	rs505404	3.11E-08	0.0004087	3.73E-187	0	Whole_Blood
ENSG00000006125	AP2B1	17	33913918	34053436	ENSG00000123908	AGO2	8	141541264	141645718	0.011984	8.3693	3.35E-10	9.58E-05	NA	NA	NA	rs10512472	1.33E-11	0	3.62E-14	0	Heart_Left_Ventricle
ENSG00000132139	GAS2L2	17	34071530	34079897	ENSG00000138867	GUCD1	22	24936406	24951284	0.012484	7.6311	3.39E-10	9.68E-05	NA	NA	NA	rs9915021	1.78E-10	0	NA	NA	Small_Intestine_Terminal_Ileum
ENSG00000118640	VAMP8	2	85788685	85809154	ENSG00000169439	SDC2	8	97505579	97624000	0.011446	9.3228	3.44E-10	9.82E-05	0.035669	4.8296	2.34E-05	rs10187424	7.24E-15	0	3.2717e-310	0	Esophagus_Mucosa
ENSG00000108733	PEX12	17	33901814	33905882	ENSG00000197993	KEL	7	142638201	142659768	0.010884	10.634	3.53E-10	0.00010044	0.022602	4.2365	0.00081878	rs9915021	1.56E-12	0	NA	NA	Brain_Caudate_basal_ganglia
ENSG00000108733	PEX12	17	33901814	33905882	ENSG00000197993	KEL	7	142638201	142659768	0.010884	10.634	3.53E-10	0.00010044	0.022602	4.2365	0.00081878	rs9915021	1.56E-12	0	NA	NA	Breast_Mammary_Tissue
ENSG00000108733	PEX12	17	33901814	33905882	ENSG00000197993	KEL	7	142638201	142659768	0.010884	10.634	3.53E-10	0.00010044	0.022602	4.2365	0.00081878	rs10512472	7.82E-19	0	1.06E-12	0	Brain_Caudate_basal_ganglia
ENSG00000172660	TAF15	17	34136459	34174246	ENSG00000118046	STK11	19	1189406	1226662	0.011435	9.3135	3.53E-10	0.00010044	0.0081283	1.2497	0.27839	rs9915021	3.14E-08	0.00040834	NA	NA	Skin_Not_Sun_Exposed_Suprapubic
ENSG00000154760	SLFN13	17	33762115	33775856	ENSG00000198959	TGM2	20	36756863	36794980	0.013878	6.1744	3.63E-10	0.0001028	0.0068024	0.51881	0.90358	rs225245	1.10E-13	0	NA	NA	Liver
ENSG00000154760	SLFN13	17	33762115	33775856	ENSG00000198959	TGM2	20	36756863	36794980	0.013878	6.1744	3.63E-10	0.0001028	0.0068024	0.51881	0.90358	rs10512472	9.83E-09	0.00016316	6.34E-38	0	Uterus
ENSG00000006125	AP2B1	17	33913918	34053436	ENSG00000184792	OSBP2	22	31089769	31303811	0.011937	8.3361	3.72E-10	0.00010498	0.018771	2.9173	0.0079949	rs10512472	2.35E-08	0.00031703	3.62E-14	0	Heart_Left_Ventricle
ENSG00000154760	SLFN13	17	33762115	33775856	ENSG00000166169	POLL	10	103338639	103346957	0.013862	6.1672	3.75E-10	0.00010558	0.031259	2.4443	0.0039046	rs225245	1.93E-71	0	NA	NA	Liver
ENSG00000154760	SLFN13	17	33762115	33775856	ENSG00000166169	POLL	10	103338639	103346957	0.013862	6.1672	3.75E-10	0.00010558	0.031259	2.4443	0.0039046	rs8082605	2.02E-34	0	2.08E-19	0	Artery_Tibial
ENSG00000154760	SLFN13	17	33762115	33775856	ENSG00000166169	POLL	10	103338639	103346957	0.013862	6.1672	3.75E-10	0.00010558	0.031259	2.4443	0.0039046	rs8082605	2.02E-34	0	2.08E-19	0	Esophagus_Mucosa
ENSG00000154760	SLFN13	17	33762115	33775856	ENSG00000166169	POLL	10	103338639	103346957	0.013862	6.1672	3.75E-10	0.00010558	0.031259	2.4443	0.0039046	rs8082605	2.02E-34	0	2.08E-19	0	Nerve_Tibial
ENSG00000154760	SLFN13	17	33762115	33775856	ENSG00000166169	POLL	10	103338639	103346957	0.013862	6.1672	3.75E-10	0.00010558	0.031259	2.4443	0.0039046	rs8082605	2.02E-34	0	2.08E-19	0	Pituitary
ENSG00000154760	SLFN13	17	33762115	33775856	ENSG00000166169	POLL	10	103338639	103346957	0.013862	6.1672	3.75E-10	0.00010558	0.031259	2.4443	0.0039046	rs10512472	1.87E-33	0	6.34E-38	0	Uterus
ENSG00000168374	ARF4	3	57557090	57583947	ENSG00000122786	CALD1	7	134429003	134655479	0.010857	10.607	3.76E-10	0.00010563	0.032462	6.1465	1.30E-05	rs17825630	2.02E-25	0	NA	NA	Artery_Tibial
ENSG00000168374	ARF4	3	57557090	57583947	ENSG00000122786	CALD1	7	134429003	134655479	0.010857	10.607	3.76E-10	0.00010563	0.032462	6.1465	1.30E-05	rs17825630	2.02E-25	0	NA	NA	Heart_Atrial_Appendage
ENSG00000108733	PEX12	17	33901814	33905882	ENSG00000090674	MCOLN1	19	7587512	7595387	0.010815	10.566	4.15E-10	0.00011606	0.0020194	0.3707	0.86887	rs9915021	9.31E-12	0	NA	NA	Brain_Caudate_basal_ganglia
ENSG00000108733	PEX12	17	33901814	33905882	ENSG00000090674	MCOLN1	19	7587512	7595387	0.010815	10.566	4.15E-10	0.00011606	0.0020194	0.3707	0.86887	rs9915021	9.31E-12	0	NA	NA	Breast_Mammary_Tissue
ENSG00000108733	PEX12	17	33901814	33905882	ENSG00000090674	MCOLN1	19	7587512	7595387	0.010815	10.566	4.15E-10	0.00011606	0.0020194	0.3707	0.86887	rs10512472	3.45E-14	0	1.06E-12	0	Brain_Caudate_basal_ganglia
ENSG00000163946	FAM208A	3	56658507	56717265	ENSG00000166091	CMTM5	14	23846017	23848981	0.012388	7.5712	4.20E-10	0.00011743	0.065831	9.2014	4.97E-11	rs12485738	1.80E-242	0	NA	NA	Esophagus_Mucosa
ENSG00000163946	FAM208A	3	56658507	56717265	ENSG00000166091	CMTM5	14	23846017	23848981	0.012388	7.5712	4.20E-10	0.00011743	0.065831	9.2014	4.97E-11	rs1354034	3.27170000000001e-	0	NA	NA	Heart_Atrial_Appendage
ENSG00000167914	GSDMA	17	38119226	38134019	ENSG00000146592	CREB5	7	28338940	28865511	0.0088789	21.657	4.33E-10	0.00012086	0.0027455	1.265	0.28272	rs3859192	1.81E-15	0	6.22E-13	0	Adipose_Subcutaneous
ENSG00000167914	GSDMA	17	38119226	38134019	ENSG00000146592	CREB5	7	28338940	28865511	0.0088789	21.657	4.33E-10	0.00012086	0.0027455	1.265	0.28272	rs3859192	1.81E-15	0	6.22E-13	0	Adipose_Visceral_Omentum
ENSG00000167914	GSDMA	17	38119226	38134019	ENSG00000146592	CREB5	7	28338940	28865511	0.0088789	21.657	4.33E-10	0.00012086	0.0027455	1.265	0.28272	rs3859192	1.81E-15	0	6.22E-13	0	Adrenal_Gland
ENSG00000167914	GSDMA	17	38119226	38134019	ENSG00000146592	CREB5	7	28338940	28865511	0.0088789	21.657	4.33E-10	0.00012086	0.0027455	1.265	0.28272	rs3859192	1.81E-15	0	6.22E-13	0	Artery_Aorta
ENSG00000167914	GSDMA	17	38119226	38134019	ENSG00000146592	CREB5	7	28338940	28865511	0.0088789	21.657	4.33E-10	0.00012086	0.0027455	1.265	0.28272	rs3859192	1.81E-15	0	6.22E-13	0	Artery_Coronary
ENSG00000167914	GSDMA	17	38119226	38134019	ENSG00000146592	CREB5	7	28338940	28865511	0.0088789	21.657	4.33E-10	0.00012086	0.0027455	1.265	0.28272	rs3859192	1.81E-15	0	6.22E-13	0	Artery_Tibial
ENSG00000167914	GSDMA	17	38119226	38134019	ENSG00000146592	CREB5	7	28338940	28865511	0.0088789	21.657	4.33E-10	0.00012086	0.0027455	1.265	0.28272	rs3859192	1.81E-15	0	6.22E-13	0	Breast_Mammary_Tissue
ENSG00000167914	GSDMA	17	38119226	38134019	ENSG00000146592	CREB5	7	28338940	28865511	0.0088789	21.657	4.33E-10	0.00012086	0.0027455	1.265	0.28272	rs3859192	1.81E-15	0	6.22E-13	0	Cells_Transformed_fibroblasts
ENSG00000167914	GSDMA	17	38119226	38134019	ENSG00000146592	CREB5	7	28338940	28865511	0.0088789	21.657	4.33E-10	0.00012086	0.0027455	1.265	0.28272	rs3859192	1.81E-15	0	6.22E-13	0	Colon_Sigmoid
ENSG00000167914	GSDMA	17	38119226	38134019	ENSG00000146592	CREB5	7	28338940	28865511	0.0088789	21.657	4.33E-10	0.00012086	0.0027455	1.265	0.28272	rs3859192	1.81E-15	0	6.22E-13	0	Colon_Transverse
ENSG00000167914	GSDMA	17	38119226	38134019	ENSG00000146592	CREB5	7	28338940	28865511	0.0088789	21.657	4.33E-10	0.00012086	0.0027455	1.265	0.28272	rs3859192	1.81E-15	0	6.22E-13	0	Esophagus_Gastroesophageal_Junction
ENSG00000167914	GSDMA	17	38119226	38134019	ENSG00000146592	CREB5	7	28338940	28865511	0.0088789	21.657	4.33E-10	0.00012086	0.0027455	1.265	0.28272	rs3859192	1.81E-15	0	6.22E-13	0	Esophagus_Muscularis
ENSG00000167914	GSDMA	17	38119226	38134019	ENSG00000146592	CREB5	7	28338940	28865511	0.0088789	21.657	4.33E-10	0.00012086	0.0027455	1.265	0.28272	rs3859192	1.81E-15	0	6.22E-13	0	Heart_Atrial_Appendage
ENSG00000167914	GSDMA	17	38119226	38134019	ENSG00000146592	CREB5	7	28338940	28865511	0.0088789	21.657	4.33E-10	0.00012086	0.0027455	1.265	0.28272	rs3859192	1.81E-15	0	6.22E-13	0	Heart_Left_Ventricle
ENSG00000167914	GSDMA	17	38119226	38134019	ENSG00000146592	CREB5	7	28338940	28865511	0.0088789	21.657	4.33E-10	0.00012086	0.0027455	1.265	0.28272	rs3859192	1.81E-15	0	6.22E-13	0	Liver
ENSG00000167914	GSDMA	17	38119226	38134019	ENSG00000146592	CREB5	7	28338940	28865511	0.0088789	21.657	4.33E-10	0.00012086	0.0027455	1.265	0.28272	rs3859192	1.81E-15	0	6.22E-13	0	Lung
ENSG00000167914	GSDMA	17	38119226	38134019	ENSG00000146592	CREB5	7	28338940	28865511	0.0088789	21.657	4.33E-10	0.00012086	0.0027455	1.265	0.28272	rs3859192	1.81E-15	0	6.22E-13	0	Muscle_Skeletal
ENSG00000167914	GSDMA	17	38119226	38134019	ENSG00000146592	CREB5	7	28338940	28865511	0.0088789	21.657	4.33E-10	0.00012086	0.0027455	1.265	0.28272	rs3859192	1.81E-15	0	6.22E-13	0	Nerve_Tibial
ENSG00000167914	GSDMA	17	38119226	38134019	ENSG00000146592	CREB5	7	28338940	28865511	0.0088789	21.657	4.33E-10	0.00012086	0.0027455	1.265	0.28272	rs3859192	1.81E-15	0	6.22E-13	0	Ovary
ENSG00000167914	GSDMA	17	38119226	38134019	ENSG00000146592	CREB5	7	28338940	28865511	0.0088789	21.657	4.33E-10	0.00012086	0.0027455	1.265	0.28272	rs3859192	1.81E-15	0	6.22E-13	0	Pancreas
ENSG00000167914	GSDMA	17	38119226	38134019	ENSG00000146592	CREB5	7	28338940	28865511	0.0088789	21.657	4.33E-10	0.00012086	0.0027455	1.265	0.28272	rs3859192	1.81E-15	0	6.22E-13	0	Small_Intestine_Terminal_Ileum
ENSG00000167914	GSDMA	17	38119226	38134019	ENSG00000146592	CREB5	7	28338940	28865511	0.0088789	21.657	4.33E-10	0.00012086	0.0027455	1.265	0.28272	rs3859192	1.81E-15	0	6.22E-13	0	Spleen
ENSG00000167914	GSDMA	17	38119226	38134019	ENSG00000146592	CREB5	7	28338940	28865511	0.0088789	21.657	4.33E-10	0.00012086	0.0027455	1.265	0.28272	rs3859192	1.81E-15	0	6.22E-13	0	Stomach
ENSG00000167914	GSDMA	17	38119226	38134019	ENSG00000146592	CREB5	7	28338940	28865511	0.0088789	21.657	4.33E-10	0.00012086	0.0027455	1.265	0.28272	rs3859192	1.81E-15	0	6.22E-13	0	Thyroid
ENSG00000167914	GSDMA	17	38119226	38134019	ENSG00000146592	CREB5	7	28338940	28865511	0.0088789	21.657	4.33E-10	0.00012086	0.0027455	1.265	0.28272	rs3859192	1.81E-15	0	6.22E-13	0	Whole_Blood
ENSG00000167914	GSDMA	17	38119226	38134019	ENSG00000146592	CREB5	7	28338940	28865511	0.0088789	21.657	4.33E-10	0.00012086	0.0027455	1.265	0.28272	rs17609240	2.81E-09	7.29E-05	1.53E-144	0	Adipose_Subcutaneous
ENSG00000167914	GSDMA	17	38119226	38134019	ENSG00000146592	CREB5	7	28338940	28865511	0.0088789	21.657	4.33E-10	0.00012086	0.0027455	1.265	0.28272	rs17609240	2.81E-09	7.29E-05	1.53E-144	0	Adipose_Visceral_Omentum
ENSG00000167914	GSDMA	17	38119226	38134019	ENSG00000146592	CREB5	7	28338940	28865511	0.0088789	21.657	4.33E-10	0.00012086	0.0027455	1.265	0.28272	rs17609240	2.81E-09	7.29E-05	1.53E-144	0	Artery_Aorta
ENSG00000167914	GSDMA	17	38119226	38134019	ENSG00000146592	CREB5	7	28338940	28865511	0.0088789	21.657	4.33E-10	0.00012086	0.0027455	1.265	0.28272	rs17609240	2.81E-09	7.29E-05	1.53E-144	0	Breast_Mammary_Tissue
ENSG00000167914	GSDMA	17	38119226	38134019	ENSG00000146592	CREB5	7	28338940	28865511	0.0088789	21.657	4.33E-10	0.00012086	0.0027455	1.265	0.28272	rs17609240	2.81E-09	7.29E-05	1.53E-144	0	Colon_Sigmoid
ENSG00000167914	GSDMA	17	38119226	38134019	ENSG00000146592	CREB5	7	28338940	28865511	0.0088789	21.657	4.33E-10	0.00012086	0.0027455	1.265	0.28272	rs17609240	2.81E-09	7.29E-05	1.53E-144	0	Esophagus_Gastroesophageal_Junction
ENSG00000167914	GSDMA	17	38119226	38134019	ENSG00000146592	CREB5	7	28338940	28865511	0.0088789	21.657	4.33E-10	0.00012086	0.0027455	1.265	0.28272	rs17609240	2.81E-09	7.29E-05	1.53E-144	0	Esophagus_Muscularis
ENSG00000167914	GSDMA	17	38119226	38134019	ENSG00000146592	CREB5	7	28338940	28865511	0.0088789	21.657	4.33E-10	0.00012086	0.0027455	1.265	0.28272	rs17609240	2.81E-09	7.29E-05	1.53E-144	0	Heart_Left_Ventricle
ENSG00000167914	GSDMA	17	38119226	38134019	ENSG00000146592	CREB5	7	28338940	28865511	0.0088789	21.657	4.33E-10	0.00012086	0.0027455	1.265	0.28272	rs17609240	2.81E-09	7.29E-05	1.53E-144	0	Muscle_Skeletal
ENSG00000167914	GSDMA	17	38119226	38134019	ENSG00000146592	CREB5	7	28338940	28865511	0.0088789	21.657	4.33E-10	0.00012086	0.0027455	1.265	0.28272	rs3894194	4.69E-11	0	6.08E-29	0	Adipose_Subcutaneous
ENSG00000167914	GSDMA	17	38119226	38134019	ENSG00000146592	CREB5	7	28338940	28865511	0.0088789	21.657	4.33E-10	0.00012086	0.0027455	1.265	0.28272	rs3894194	4.69E-11	0	6.08E-29	0	Adipose_Visceral_Omentum
ENSG00000167914	GSDMA	17	38119226	38134019	ENSG00000146592	CREB5	7	28338940	28865511	0.0088789	21.657	4.33E-10	0.00012086	0.0027455	1.265	0.28272	rs3894194	4.69E-11	0	6.08E-29	0	Artery_Tibial
ENSG00000167914	GSDMA	17	38119226	38134019	ENSG00000146592	CREB5	7	28338940	28865511	0.0088789	21.657	4.33E-10	0.00012086	0.0027455	1.265	0.28272	rs3894194	4.69E-11	0	6.08E-29	0	Colon_Transverse
ENSG00000167914	GSDMA	17	38119226	38134019	ENSG00000146592	CREB5	7	28338940	28865511	0.0088789	21.657	4.33E-10	0.00012086	0.0027455	1.265	0.28272	rs3894194	4.69E-11	0	6.08E-29	0	Esophagus_Gastroesophageal_Junction
ENSG00000167914	GSDMA	17	38119226	38134019	ENSG00000146592	CREB5	7	28338940	28865511	0.0088789	21.657	4.33E-10	0.00012086	0.0027455	1.265	0.28272	rs3894194	4.69E-11	0	6.08E-29	0	Heart_Atrial_Appendage
ENSG00000167914	GSDMA	17	38119226	38134019	ENSG00000146592	CREB5	7	28338940	28865511	0.0088789	21.657	4.33E-10	0.00012086	0.0027455	1.265	0.28272	rs3894194	4.69E-11	0	6.08E-29	0	Muscle_Skeletal
ENSG00000167914	GSDMA	17	38119226	38134019	ENSG00000146592	CREB5	7	28338940	28865511	0.0088789	21.657	4.33E-10	0.00012086	0.0027455	1.265	0.28272	rs3894194	4.69E-11	0	6.08E-29	0	Whole_Blood
ENSG00000167914	GSDMA	17	38119226	38134019	ENSG00000146592	CREB5	7	28338940	28865511	0.0088789	21.657	4.33E-10	0.00012086	0.0027455	1.265	0.28272	rs7212938	2.44E-08	0.00033038	5.34E-44	0	Adipose_Subcutaneous
ENSG00000167914	GSDMA	17	38119226	38134019	ENSG00000146592	CREB5	7	28338940	28865511	0.0088789	21.657	4.33E-10	0.00012086	0.0027455	1.265	0.28272	rs7212938	2.44E-08	0.00033038	5.34E-44	0	Adipose_Visceral_Omentum
ENSG00000167914	GSDMA	17	38119226	38134019	ENSG00000146592	CREB5	7	28338940	28865511	0.0088789	21.657	4.33E-10	0.00012086	0.0027455	1.265	0.28272	rs7212938	2.44E-08	0.00033038	5.34E-44	0	Artery_Coronary
ENSG00000167914	GSDMA	17	38119226	38134019	ENSG00000146592	CREB5	7	28338940	28865511	0.0088789	21.657	4.33E-10	0.00012086	0.0027455	1.265	0.28272	rs7212938	2.44E-08	0.00033038	5.34E-44	0	Colon_Sigmoid
ENSG00000167914	GSDMA	17	38119226	38134019	ENSG00000146592	CREB5	7	28338940	28865511	0.0088789	21.657	4.33E-10	0.00012086	0.0027455	1.265	0.28272	rs7212938	2.44E-08	0.00033038	5.34E-44	0	Heart_Atrial_Appendage
ENSG00000167914	GSDMA	17	38119226	38134019	ENSG00000146592	CREB5	7	28338940	28865511	0.0088789	21.657	4.33E-10	0.00012086	0.0027455	1.265	0.28272	rs7212938	2.44E-08	0.00033038	5.34E-44	0	Heart_Left_Ventricle

ENSG00000167914	GSDMA	17	38119226	38134019	ENSG00000146592	CREB5	7	28338940	28865511	0.0088789	21.657	4.33E-10	0.00012086	0.0027455	1.265	0.28272	rs7212938	2.44E-08	0.00033038	5.34E-44	0	Thyroid
ENSG00000167914	GSDMA	17	38119226	38134019	ENSG00000146592	CREB5	7	28338940	28865511	0.0088789	21.657	4.33E-10	0.00012086	0.0027455	1.265	0.28272	rs7212938	2.44E-08	0.00033038	5.34E-44	0	Whole_Blood
ENSG00000167914	GSDMA	17	38119226	38134019	ENSG00000146592	CREB5	7	28338940	28865511	0.0088789	21.657	4.33E-10	0.00012086	0.0027455	1.265	0.28272	rs8078723	1.90E-17	0	6.97E-07	0.002194627	Breast_Mammary_Tissue
ENSG00000167914	GSDMA	17	38119226	38134019	ENSG00000146592	CREB5	7	28338940	28865511	0.0088789	21.657	4.33E-10	0.00012086	0.0027455	1.265	0.28272	rs8078723	1.90E-17	0	6.97E-07	0.002194627	Esophagus_Gastroesophageal_Junction
ENSG00000167914	GSDMA	17	38119226	38134019	ENSG00000146592	CREB5	7	28338940	28865511	0.0088789	21.657	4.33E-10	0.00012086	0.0027455	1.265	0.28272	rs4794820	2.26E-09	7.41E-05	2.26E-66	0	Artery_Tibial
ENSG00000167914	GSDMA	17	38119226	38134019	ENSG00000146592	CREB5	7	28338940	28865511	0.0088789	21.657	4.33E-10	0.00012086	0.0027455	1.265	0.28272	rs4794820	2.26E-09	7.41E-05	2.26E-66	0	Cells_Transformed_fibroblasts
ENSG00000167914	GSDMA	17	38119226	38134019	ENSG00000146592	CREB5	7	28338940	28865511	0.0088789	21.657	4.33E-10	0.00012086	0.0027455	1.265	0.28272	rs4794822	1.89E-17	0	3.02E-06	0.008756123	Breast_Mammary_Tissue
ENSG00000167914	GSDMA	17	38119226	38134019	ENSG00000146592	CREB5	7	28338940	28865511	0.0088789	21.657	4.33E-10	0.00012086	0.0027455	1.265	0.28272	rs4794822	1.89E-17	0	3.02E-06	0.008756123	Cells_Transformed_fibroblasts
ENSG00000167914	GSDMA	17	38119226	38134019	ENSG00000146592	CREB5	7	28338940	28865511	0.0088789	21.657	4.33E-10	0.00012086	0.0027455	1.265	0.28272	rs4794822	1.89E-17	0	3.02E-06	0.008756123	Esophagus_Gastroesophageal_Junction
ENSG00000167914	GSDMA	17	38119226	38134019	ENSG00000146592	CREB5	7	28338940	28865511	0.0088789	21.657	4.33E-10	0.00012086	0.0027455	1.265	0.28272	rs4794822	1.89E-17	0	3.02E-06	0.008756123	Esophagus_Muscularis
ENSG00000167914	GSDMA	17	38119226	38134019	ENSG00000146592	CREB5	7	28338940	28865511	0.0088789	21.657	4.33E-10	0.00012086	0.0027455	1.265	0.28272	rs4065321	5.35E-14	0	2.52E-12	0	Pancreas
ENSG00000141150	RASL10B	17	34058668	34070540	ENSG00000166169	POLL	10	103338639	103346957	0.012357	7.5524	4.49E-10	0.00012528	0.02176	2.9044	0.005228	rs9915021	3.09E-30	0	NA	NA	Cells_Transformed_fibroblasts
ENSG00000163946	FAM208A	3	56658507	56717265	ENSG00000163736	PPBP	4	74852755	74853914	0.012349	7.5475	4.57E-10	0.00012715	0.051727	7.1224	2.62E-08	rs12485738	3.27170000000001e-	0	NA	NA	Esophagus_Mucosa
ENSG00000163946	FAM208A	3	56658507	56717265	ENSG00000163736	PPBP	4	74852755	74853914	0.012349	7.5475	4.57E-10	0.00012715	0.051727	7.1224	2.62E-08	rs1354034	3.27170000000001e-	0	NA	NA	Heart_Atrial_Appendage
ENSG00000108733	PEX12	17	33901814	33905882	ENSG00000180354	C7orf41	7	30174426	30202378	0.010766	10.518	4.63E-10	0.00012871	0.0094357	1.7451	0.12172	rs9915021	3.46E-60	0	NA	NA	Brain_Caudate_basal_ganglia
ENSG00000108733	PEX12	17	33901814	33905882	ENSG00000180354	C7orf41	7	30174426	30202378	0.010766	10.518	4.63E-10	0.00012871	0.0094357	1.7451	0.12172	rs9915021	3.46E-60	0	NA	NA	Breast_Mammary_Tissue
ENSG00000108733	PEX12	17	33901814	33905882	ENSG00000180354	C7orf41	7	30174426	30202378	0.010766	10.518	4.63E-10	0.00012871	0.0094357	1.7451	0.12172	rs10512472	6.35E-117	0	1.06E-12	0	Brain_Caudate_basal_ganglia
ENSG00000172057	ORMDL3	17	38077294	38083854	ENSG00000196663	TECPR2	14	102829300	102968818	0.010765	10.516	4.65E-10	0.00012903	0.0091823	1.4133	0.20636	rs4794820	8.93E-09	0.00016458	3.2717e-310	0	Spleen
ENSG00000172057	ORMDL3	17	38077294	38083854	ENSG00000196663	TECPR2	14	102829300	102968818	0.010765	10.516	4.65E-10	0.00012903	0.0091823	1.4133	0.20636	rs2872507	1.83E-06	0.01413714	3.2717e-310	0	Whole_Blood
ENSG00000172057	ORMDL3	17	38077294	38083854	ENSG00000196663	TECPR2	14	102829300	102968818	0.010765	10.516	4.65E-10	0.00012903	0.0091823	1.4133	0.20636	rs8069176	3.15E-07	0.00288581	3.2717e-310	0	Whole_Blood
ENSG00000205045	SLFN12L	17	33800708	33864880	ENSG00000154146	NRGN	11	124609829	124615878	0.011824	8.2564	4.80E-10	0.00013246	0.010384	1.3701	0.21448	rs8082605	2.53E-38	0	2.49E-31	0	Adipose_Subcutaneous
ENSG00000205045	SLFN12L	17	33800708	33864880	ENSG00000154146	NRGN	11	124609829	124615878	0.011824	8.2564	4.80E-10	0.00013246	0.010384	1.3701	0.21448	rs8082605	2.53E-38	0	2.49E-31	0	Adipose_Visceral_Omentum
ENSG00000205045	SLFN12L	17	33800708	33864880	ENSG00000154146	NRGN	11	124609829	124615878	0.011824	8.2564	4.80E-10	0.00013246	0.010384	1.3701	0.21448	rs8082605	2.53E-38	0	2.49E-31	0	Heart_Left_Ventricle
ENSG00000205045	SLFN12L	17	33800708	33864880	ENSG00000154146	NRGN	11	124609829	124615878	0.011824	8.2564	4.80E-10	0.00013246	0.010384	1.3701	0.21448	rs8082605	2.53E-38	0	2.49E-31	0	Nerve_Tibial
ENSG00000205045	SLFN12L	17	33800708	33864880	ENSG00000154146	NRGN	11	124609829	124615878	0.011824	8.2564	4.80E-10	0.00013246	0.010384	1.3701	0.21448	rs10512472	4.87E-94	0	4.96E-30	0	Pancreas
ENSG00000172716	SLFN11	17	33677324	33700720	ENSG00000166947	EPB42	15	43398423	43513481	0.014189	5.7872	4.88E-10	0.00013419	0.016432	1.2655	0.23367	rs225245	6.28E-40	0	5.77E-07	0.001884469	Cells_Transformed_fibroblasts
ENSG00000172716	SLFN11	17	33677324	33700720	ENSG00000166947	EPB42	15	43398423	43513481	0.014189	5.7872	4.88E-10	0.00013419	0.016432	1.2655	0.23367	rs8082605	2.72E-16	0	NA	NA	Adrenal_Gland
ENSG00000142102	ATHL1	11	289138	296107	ENSG00000205038	PKHD1L1	8	110374706	110542559	0.01712	4.4168	4.90E-10	0.00013458	0.0014132	0.65027	0.52215	rs11602954	3.21E-08	0.00042188	8.23E-62	0	Thyroid
ENSG00000142102	ATHL1	11	289138	296107	ENSG00000205038	PKHD1L1	8	110374706	110542559	0.01712	4.4168	4.90E-10	0.00013458	0.0014132	0.65027	0.52215	rs17655730	2.44E-13	0	1.10E-69	0	Muscle_Skeletal
ENSG00000142102	ATHL1	11	289138	296107	ENSG00000205038	PKHD1L1	8	110374706	110542559	0.01712	4.4168	4.90E-10	0.00013458	0.0014132	0.65027	0.52215	rs505404	1.11E-15	0	3.83E-63	0	Lung
ENSG00000154760	SLFN13	17	33762115	33775856	ENSG00000167992	VWCE	11	61025762	61062896	0.013706	6.0967	5.24E-10	0.00014365	0.033337	2.6124	0.0019775	rs225245	4.65E-96	0	NA	NA	Liver
ENSG00000154760	SLFN13	17	33762115	33775856	ENSG00000167992	VWCE	11	61025762	61062896	0.013706	6.0967	5.24E-10	0.00014365	0.033337	2.6124	0.0019775	rs8082605	8.92E-35	0	2.08E-19	0	Artery_Tibial
ENSG00000154760	SLFN13	17	33762115	33775856	ENSG00000167992	VWCE	11	61025762	61062896	0.013706	6.0967	5.24E-10	0.00014365	0.033337	2.6124	0.0019775	rs8082605	8.92E-35	0	2.08E-19	0	Esophagus_Mucosa
ENSG00000154760	SLFN13	17	33762115	33775856	ENSG00000167992	VWCE	11	61025762	61062896	0.013706	6.0967	5.24E-10	0.00014365	0.033337	2.6124	0.0019775	rs8082605	8.92E-35	0	2.08E-19	0	Nerve_Tibial
ENSG00000154760	SLFN13	17	33762115	33775856	ENSG00000167992	VWCE	11	61025762	61062896	0.013706	6.0967	5.24E-10	0.00014365	0.033337	2.6124	0.0019775	rs8082605	8.92E-35	0	2.08E-19	0	Pituitary
ENSG00000154760	SLFN13	17	33762115	33775856	ENSG00000167992	VWCE	11	61025762	61062896	0.013706	6.0967	5.24E-10	0.00014365	0.033337	2.6124	0.0019775	rs10512472	2.21E-55	0	6.34E-38	0	Uterus
ENSG00000163946	FAM208A	3	56658507	56717265	ENSG00000095303	PTGS1	9	125132824	125157982	0.012265	7.4955	5.50E-10	0.0001498	0.026523	3.5575	0.00088016	rs12485738	5.39E-139	0	NA	NA	Esophagus_Mucosa
ENSG00000163946	FAM208A	3	56658507	56717265	ENSG00000095303	PTGS1	9	125132824	125157982	0.012265	7.4955	5.50E-10	0.0001498	0.026523	3.5575	0.00088016	rs1354034	7.28E-253	0	NA	NA	Heart_Atrial_Appendage
ENSG00000172660	TAF15	17	34136459	34174246	ENSG00000090674	MCOLN1	19	7587512	7595387	0.011229	9.1442	5.64E-10	0.00015327	0.010045	1.5475	0.15957	rs9915021	9.31E-12	0	NA	NA	Skin_Not_Sun_Exposed_Suprapubic
ENSG00000108733	PEX12	17	33901814	33905882	ENSG00000164849	GPR146	7	1084212	1098897	0.010671	10.423	5.78E-10	0.00015669	0.0082286	1.52	0.18089	rs9915021	1.21E-09	3.90E-05	NA	NA	Brain_Caudate_basal_ganglia
ENSG00000108733	PEX12	17	33901814	33905882	ENSG00000164849	GPR146	7	1084212	1098897	0.010671	10.423	5.78E-10	0.00015669	0.0082286	1.52	0.18089	rs9915021	1.21E-09	3.90E-05	NA	NA	Breast_Mammary_Tissue
ENSG00000167136	ENDOG	9	131580753	131581464	ENSG00000101425	BPI	20	36888551	36965907	0.010084	12.308	5.86E-10	0.00015867	0.016134	2.1411	0.037211	rs13289095	8.54E-51	0	1.10E-185	0	Skin_Not_Sun_Exposed_Suprapubic
ENSG00000167136	ENDOG	9	131580753	131581464	ENSG00000101425	BPI	20	36888551	36965907	0.010084	12.308	5.86E-10	0.00015867	0.016134	2.1411	0.037211	rs15676	1.15E-32	0	3.2717e-310	0	Cells_Transformed_fibroblasts
ENSG00000167136	ENDOG	9	131580753	131581464	ENSG00000101425	BPI	20	36888551	36965907	0.010084	12.308	5.86E-10	0.00015867	0.016134	2.1411	0.037211	rs15676	1.15E-32	0	3.2717e-310	0	Esophagus_Mucosa
ENSG00000167136	ENDOG	9	131580753	131581464	ENSG00000101425	BPI	20	36888551	36965907	0.010084	12.308	5.86E-10	0.00015867	0.016134	2.1411	0.037211	rs15676	1.15E-32	0	3.2717e-310	0	Ovary
ENSG00000167136	ENDOG	9	131580753	131581464	ENSG00000101425	BPI	20	36888551	36965907	0.010084	12.308	5.86E-10	0.00015867	0.016134	2.1411	0.037211	rs15676	1.15E-32	0	3.2717e-310	0	Thyroid
ENSG00000168374	ARF4	3	57557090	57583947	ENSG00000113140	SPARC	5	151040657	151066726	0.010661	10.413	5.92E-10	0.00015994	0.018904	3.53	0.0036265	rs17825630	1.55E-14	0	NA	NA	Artery_Tibial
ENSG00000168374	ARF4	3	57557090	57583947	ENSG00000113140	SPARC	5	151040657	151066726	0.010661	10.413	5.92E-10	0.00015994	0.018904	3.53	0.0036265	rs17825630	1.55E-14	0	NA	NA	Heart_Atrial_Appendage
ENSG00000154768	C17orf50	17	34087916	34092098	ENSG00000100614	PPM1A	14	60712470	60765805	0.011194	9.1152	6.11E-10	0.00016492	0.011602	1.5327	0.15247	rs9915021	6.10E-09	0.00013559	NA	NA	Pituitary
ENSG00000108733	PEX12	17	33901814	33905882	ENSG00000130227	XPO7	8	21777180	21864096	0.010644	10.397	6.15E-10	0.0001659	0.0051594	0.9501	0.44766	rs10512472	5.74E-06	0.03701626	1.06E-12	0	Brain_Caudate_basal_ganglia
ENSG00000249242	TMEM150C	4	83405743	83483510	ENSG00000100448	CTSG	14	25042762	25045466	0.013625	6.06	6.22E-10	0.00016763	0.0051067	0.42463	0.94559	rs17005891	3.43E-21	0	NA	NA	Heart_Left_Ventricle
ENSG00000249242	TMEM150C	4	83405743	83483510	ENSG00000100448	CTSG	14	25042762	25045466	0.013625	6.06	6.22E-10	0.00016763	0.0051067	0.42463	0.94559	rs17005891	3.43E-21	0	NA	NA	Muscle_Skeletal
ENSG00000249242	TMEM150C	4	83405743	83483510	ENSG00000100448	CTSG	14	25042762	25045466	0.013625	6.06	6.22E-10	0.00016763	0.0051067	0.42463	0.94559	rs17005891	3.43E-21	0	NA	NA	Thyroid
ENSG00000172660	TAF15	17	34136459	34174246	ENSG00000134779	TPGS2	18	34361762	34403834	0.011171	9.0963	6.43E-10	0.00017305	NA	NA	NA	rs9915021	4.75E-06	0.03156441	NA	NA	Skin_Not_Sun_Exposed_Suprapubic
ENSG00000008838	MED24	17	38175350	38217468	ENSG00000162551	ALPL	1	21835858	21904905	0.010029	12.241	6.66E-10	0.00017881	0.01362	3.1655	0.013469	rs12946510	8.70E-07	0.00729639	NA	NA	Prostate
ENSG00000008838	MED24	17	38175350	38217468	ENSG00000162551	ALPL	1	21835858	21904905	0.010029	12.241	6.66E-10	0.00017881	0.01362	3.1655	0.013469	rs3859192	3.20E-29	0	1.47E-40	0	Whole_Blood
ENSG00000008838	MED24	17	38175350	38217468	ENSG00000162551	ALPL	1	21835858	21904905	0.010029	12.241	6.66E-10	0.00017881	0.01362	3.1655	0.013469	rs3894194	2.28E-14	0	8.18E-15	0	Thyroid
ENSG00000008838	MED24	17	38175350	38217468	ENSG00000162551	ALPL	1	21835858	21904905	0.010029	12.241	6.66E-10	0.00017881	0.01362	3.1655	0.013469	rs7212938	1.21E-12	0	4.22E-10	0	Testis
ENSG00000008838	MED24	17	38175350	38217468	ENSG00000162551	ALPL	1	21835858	21904905	0.010029	12.241	6.66E-10	0.00017881	0.01362	3.1655	0.013469	rs7212938	1.21E-12	0	4.22E-10	0	Thyroid
ENSG00000008838	MED24	17	38175350	38217468	ENSG00000162551	ALPL	1	21835858	21904905	0.010029	12.241	6.66E-10	0.00017881	0.01362	3.1655	0.013469	rs8078723	3.63E-38	0	5.30E-78	0	Adipose_Subcutaneous
ENSG00000008838	MED24	17	38175350	38217468	ENSG00000162551	ALPL	1	21835858	21904905	0.010029	12.241	6.66E-10	0.00017881	0.01362	3.1655	0.013469	rs8078723	3.63E-38	0	5.30E-78	0	Artery_Tibial
ENSG00000008838	MED24	17	38175350	38217468	ENSG00000162551	ALPL	1	21835858	21904905	0.010029	12.241	6.66E-10	0.00017881	0.01362	3.1655	0.013469	rs8078723	3.63E-38	0	5.30E-78	0	Cells_Transformed_fibroblasts
ENSG00000008838	MED24	17	38175350	38217468	ENSG00000162551	ALPL	1	21835858	21904905	0.010029	12.241	6.66E-10	0.00017881	0.01362	3.1655	0.013469	rs8078723	3.63E-38	0	5.30E-78	0	Lung
ENSG00000008838	MED24	17	38175350	38217468	ENSG00000162551	ALPL	1	21835858	21904905	0.010029	12.241	6.66E-10	0.00017881	0.01362	3.1655	0.013469	rs8078723	3.63E-38	0	5.30E-78	0	Pituitary
ENSG00000008838	MED24	17	38175350	38217468	ENSG00000162551	ALPL	1	21835858	21904905	0.010029	12.241	6.66E-10	0.00017881	0.01362	3.1655	0.013469	rs8078723	3.63E-38	0	5.30E-78	0	Skin_Sun_Exposed_Lower_leg
ENSG00000008838	MED24	17	38175350	38217468	ENSG00000162551	ALPL	1	21835858	21904905	0.010029	12.241	6.66E-10	0.00017881	0.01362	3.1655	0.013469	rs8078723	3.63E-38	0	5.30E-78	0	Spleen
ENSG00000008838	MED24	17	38175350	38217468	ENSG00000162551	ALPL	1	21835858	21904905	0.010029	12.241	6.66E-10	0.00017881	0.01362	3.1655	0.013469	rs8078723	3.63E-38	0	5.30E-78	0	Stomach
ENSG00000008838	MED24	17	38175350	38217468	ENSG00000162551	ALPL	1	21835858	21904905	0.010029	12.241	6.66E-10	0.00017881	0.01362	3.1655	0.013469	rs8078723	3.63E-38	0	5.30E-78	0	Thyroid
ENSG00000008838	MED24	17	38175350	38217468	ENSG00000162551	ALPL	1	21835858	21904905	0.010029	12.241	6.66E-10	0.00017881	0.01362	3.1655	0.013469	rs4794822	1.31E-36	0	1.32E-79	0	Esophagus_Gastroesophageal_Junction
ENSG00000008838	MED24	17	38175350	38217468	ENSG00000162551	ALPL	1	21835858	21904905	0.010029	12.241	6.66E-10	0.00017881	0.01362	3.1655	0.013469	rs4794822	1.31E-36	0	1.32E-79	0	Lung
ENSG00000008838	MED24	17	38175350	38217468	ENSG00000162551	ALPL	1	21835858	21904905	0.010029	12.241	6.66E-10	0.00017881	0.01362	3.1655	0.013469	rs4794822	1.31E-36	0	1.32E-79	0	Spleen
ENSG00000008838	MED24	17	38175350	38217468	ENSG00000162551	ALPL	1	21835858	21904905	0.010029	12.241	6.66E-10	0.00017881	0.01362	3.1655	0.013469	rs4794822	1.31E-36	0	1.32E-79	0	Whole_Blood
ENSG00000008838	MED24	17	38175350	38217468	ENSG00000162551	ALPL	1	21835858	21904905	0.010029	12.241	6.66E-10	0.00017881	0.01362	3.1655	0.013469	rs4065321	4.43E-35	0	1.24E-42	0	Pituitary
ENSG00000008838	MED24	17	38175350	38217468	ENSG00000162551	ALPL	1	21835858	21904905	0.010029	12.241	6.66E-10	0.00017881	0.01362	3.1655	0.013469	rs8067378	2.19E-07	0.00212414	NA	NA	Brain_Hypothalamus
ENSG00000172716	SLFN11	17	33677324	33700720	ENSG00000029534	ANK1	8	41510739	41754280	0.014	5.7093	7.25E-10	0.00019319	0.02571	1.9989	0.021614	rs225245	8.61E-22	0	5.77E-07	0.001884469	Cells_Transformed_fibroblasts
ENSG00000172716	SLFN11	17	33677324	33700720	ENSG00000029534	ANK1	8	41510739	41754280	0.014	5.7093	7.25E-10	0.00019319	0.02571	1.9989	0.021614	rs8082605	4.69E-14	0	NA	NA	Adrenal_Gland
ENSG00000099849	RASSF7	11	560404	564271	ENSG00000122786	CALD1	7	134429003	134655479	0.011636	8.2231	7.31E-10	0.00019468	0.010468	1.3813	0.20964	rs505404	1.84E-40	0	NA	NA	Brain_Frontal_Cortex_BA9
ENSG00000141150	RASL10B	17	34058668	34070540	ENSG00000111105	TSPAN9	12	3186521	3395730	0.012121	7.4062	7.57E-10	0.00019993	0.051227	7.0499	3.26E-08	rs9915021	7.16E-59	0	NA	NA	Cells_Transformed_fibroblasts
ENSG00000142082	SIRT3	11	215458	236431	ENSG00000172572	PDE3A	12	20522179	20837315	0.0093494	15.207	7.56E-10	0.00019993	NA	NA	NA	rs505404	3.08E-06	0.02212182	1.05E-94	0	Adrenal_Gland
ENSG00000142082	SIRT3	11	215458	236431	ENSG00000172572	PDE3A	12	20522179	20837315	0.0093494	15.207	7.56E-10	0.00019993	NA	NA	NA	rs505404	3.08E-06	0.02212182	1.05E-94	0	Artery_Aorta
ENSG00000142082	SIRT3	11	215458	236431	ENSG00000172572	PDE3A	12	20522179	20837315	0.0093494	15.207	7.56E-10	0.00019993	NA	NA	NA	rs505404	3.08E-06	0.02212182	1.05E-94	0	Lung
ENSG00000142082	SIRT3	11	215458	236431	ENSG00000172572	PDE3A	12	20522179	20837315	0.0093494	15.207	7.56E-10	0.00019993	NA	NA	NA	rs505404	3.08E-06	0.02212182	1.05E-94	0	Thyroid

ENSG00000154760	SLFN13	17	33762115	33775856	ENSG00000103257	SLC7A5	16	87863629	87903094	0.013517	6.0114	7.83E-10	0.00020617	0.02084	1.6122	0.08271	rs225245	2.55E-09	7.35E-05	NA	NA	Liver
ENSG00000154760	SLFN13	17	33762115	33775856	ENSG00000103257	SLC7A5	16	87863629	87903094	0.013517	6.0114	7.83E-10	0.00020617	0.02084	1.6122	0.08271	rs8082605	1.28E-07	0.00137418	2.08E-19	0	Artery_Tibial
ENSG00000154760	SLFN13	17	33762115	33775856	ENSG00000103257	SLC7A5	16	87863629	87903094	0.013517	6.0114	7.83E-10	0.00020617	0.02084	1.6122	0.08271	rs8082605	1.28E-07	0.00137418	2.08E-19	0	Esophagus_Mucosa
ENSG00000154760	SLFN13	17	33762115	33775856	ENSG00000103257	SLC7A5	16	87863629	87903094	0.013517	6.0114	7.83E-10	0.00020617	0.02084	1.6122	0.08271	rs8082605	1.28E-07	0.00137418	2.08E-19	0	Nerve_Tibial
ENSG00000154760	SLFN13	17	33762115	33775856	ENSG00000103257	SLC7A5	16	87863629	87903094	0.013517	6.0114	7.83E-10	0.00020617	0.02084	1.6122	0.08271	rs8082605	1.28E-07	0.00137418	2.08E-19	0	Pituitary
ENSG00000132139	GAS2L2	17	34071530	34079897	ENSG00000167992	VWCE	11	61025762	61062896	0.012091	7.3878	8.08E-10	0.00021211	0.010185	1.1743	0.31147	rs9915021	1.45E-41	0	NA	NA	Small_Intestine_Terminal_Ileum
ENSG00000205045	SLFN12L	17	33800708	33864880	ENSG00000174175	SELP	1	169558087	169599431	0.011574	8.0797	8.39E-10	0.00021907	0.018241	2.4261	0.018237	rs8082605	6.09E-06	0.03891064	2.49E-31	0	Adipose_Subcutaneous
ENSG00000205045	SLFN12L	17	33800708	33864880	ENSG00000174175	SELP	1	169558087	169599431	0.011574	8.0797	8.39E-10	0.00021907	0.018241	2.4261	0.018237	rs8082605	6.09E-06	0.03891064	2.49E-31	0	Adipose_Visceral_Omentum
ENSG00000205045	SLFN12L	17	33800708	33864880	ENSG00000174175	SELP	1	169558087	169599431	0.011574	8.0797	8.39E-10	0.00021907	0.018241	2.4261	0.018237	rs8082605	6.09E-06	0.03891064	2.49E-31	0	Heart_Left_Ventricle
ENSG00000205045	SLFN12L	17	33800708	33864880	ENSG00000174175	SELP	1	169558087	169599431	0.011574	8.0797	8.39E-10	0.00021907	0.018241	2.4261	0.018237	rs8082605	6.09E-06	0.03891064	2.49E-31	0	Nerve_Tibial
ENSG00000205045	SLFN12L	17	33800708	33864880	ENSG00000174175	SELP	1	169558087	169599431	0.011574	8.0797	8.39E-10	0.00021907	0.018241	2.4261	0.018237	rs10512472	1.49E-15	0	4.96E-30	0	Pancreas
ENSG00000168374	ARF4	3	57557090	57583947	ENSG00000164116	GUCY1A3	4	156587863	156653501	0.010484	10.239	8.88E-10	0.0002299	0.027668	5.213	0.00010009	rs17825630	7.27E-10	2.03E-05	NA	NA	Artery_Tibial
ENSG00000168374	ARF4	3	57557090	57583947	ENSG00000164116	GUCY1A3	4	156587863	156653501	0.010484	10.239	8.88E-10	0.0002299	0.027668	5.213	0.00010009	rs17825630	7.27E-10	2.03E-05	NA	NA	Heart_Atrial_Appendage
ENSG00000154760	SLFN13	17	33762115	33775856	ENSG00000120885	CLU	8	27454434	27472548	0.013456	5.9839	8.91E-10	0.0002303	0.0099461	0.76098	0.69126	rs225245	1.24E-08	0.00017575	NA	NA	Liver
ENSG00000154760	SLFN13	17	33762115	33775856	ENSG00000120885	CLU	8	27454434	27472548	0.013456	5.9839	8.91E-10	0.0002303	0.0099461	0.76098	0.69126	rs8082605	4.14E-10	2.12E-05	2.08E-19	0	Artery_Tibial
ENSG00000154760	SLFN13	17	33762115	33775856	ENSG00000120885	CLU	8	27454434	27472548	0.013456	5.9839	8.91E-10	0.0002303	0.0099461	0.76098	0.69126	rs8082605	4.14E-10	2.12E-05	2.08E-19	0	Esophagus_Mucosa
ENSG00000154760	SLFN13	17	33762115	33775856	ENSG00000120885	CLU	8	27454434	27472548	0.013456	5.9839	8.91E-10	0.0002303	0.0099461	0.76098	0.69126	rs8082605	4.14E-10	2.12E-05	2.08E-19	0	Nerve_Tibial
ENSG00000154760	SLFN13	17	33762115	33775856	ENSG00000120885	CLU	8	27454434	27472548	0.013456	5.9839	8.91E-10	0.0002303	0.0099461	0.76098	0.69126	rs8082605	4.14E-10	2.12E-05	2.08E-19	0	Pituitary
ENSG00000154760	SLFN13	17	33762115	33775856	ENSG00000120885	CLU	8	27454434	27472548	0.013456	5.9839	8.91E-10	0.0002303	0.0099461	0.76098	0.69126	rs10512472	1.27E-27	0	6.34E-38	0	Uterus
ENSG00000108733	PEX12	17	33901814	33905882	ENSG00000136732	GYPC	2	127413509	127454246	0.010462	10.218	9.35E-10	0.00024097	0.011196	2.0743	0.066402	rs9915021	3.56E-10	2.15E-05	NA	NA	Brain_Caudate_basal_ganglia
ENSG00000108733	PEX12	17	33901814	33905882	ENSG00000136732	GYPC	2	127413509	127454246	0.010462	10.218	9.35E-10	0.00024097	0.011196	2.0743	0.066402	rs9915021	3.56E-10	2.15E-05	NA	NA	Breast_Mammary_Tissue
ENSG00000108733	PEX12	17	33901814	33905882	ENSG00000136732	GYPC	2	127413509	127454246	0.010462	10.218	9.35E-10	0.00024097	0.011196	2.0743	0.066402	rs10512472	1.09E-24	0	1.06E-12	0	Brain_Caudate_basal_ganglia
ENSG00000006125	AP2B1	17	33913918	34053436	ENSG00000019102	VSIG2	11	124618002	124621476	0.011494	8.0234	1.00E-09	0.00025722	0.015351	2.3775	0.027628	rs10512472	3.73E-08	0.00050079	3.62E-14	0	Heart_Left_Ventricle
ENSG00000141150	RASL10B	17	34058668	34070540	ENSG00000113140	SPARC	5	151040657	151066726	0.011986	7.3226	1.02E-09	0.00026125	0.028882	3.8833	0.0003532	rs9915021	8.30E-44	0	NA	NA	Cells_Transformed_fibroblasts
ENSG00000163946	FAM208A	3	56658507	56717265	ENSG00000140479	PCSK6	15	101840818	102065405	0.011972	7.3139	1.05E-09	0.00026878	0.043582	5.9498	8.69E-07	rs12485738	2.49E-52	0	NA	NA	Esophagus_Mucosa
ENSG00000163946	FAM208A	3	56658507	56717265	ENSG00000140479	PCSK6	15	101840818	102065405	0.011972	7.3139	1.05E-09	0.00026878	0.043582	5.9498	8.69E-07	rs1354034	1.83E-109	0	NA	NA	Heart_Atrial_Appendage
ENSG00000141150	RASL10B	17	34058668	34070540	ENSG00000158828	PINK1	1	20959948	20978004	0.011966	7.3104	1.07E-09	0.00027121	0.015351	2.0356	0.048108	rs9915021	5.07E-27	0	NA	NA	Cells_Transformed_fibroblasts
ENSG00000168374	ARF4	3	57557090	57583947	ENSG00000166333	ILK	11	6624961	6631511	0.010394	10.151	1.09E-09	0.00027792	0.011751	2.1784	0.054536	rs17825630	3.06E-09	0.00010842	NA	NA	Artery_Tibial
ENSG00000168374	ARF4	3	57557090	57583947	ENSG00000166333	ILK	11	6624961	6631511	0.010394	10.151	1.09E-09	0.00027792	0.011751	2.1784	0.054536	rs17825630	3.06E-09	0.00010842	NA	NA	Heart_Atrial_Appendage
ENSG00000142082	SIRT3	11	215458	236431	ENSG00000095303	PTGS1	9	125132824	125157982	0.0091945	14.953	1.09E-09	0.00027803	0.002458	0.56489	0.6882	rs11602954	3.11E-14	0	1.39E-80	0	Adipose_Subcutaneous
ENSG00000142082	SIRT3	11	215458	236431	ENSG00000095303	PTGS1	9	125132824	125157982	0.0091945	14.953	1.09E-09	0.00027803	0.002458	0.56489	0.6882	rs11602954	3.11E-14	0	1.39E-80	0	Artery_Tibial
ENSG00000142082	SIRT3	11	215458	236431	ENSG00000095303	PTGS1	9	125132824	125157982	0.0091945	14.953	1.09E-09	0.00027803	0.002458	0.56489	0.6882	rs11602954	3.11E-14	0	1.39E-80	0	Breast_Mammary_Tissue
ENSG00000142082	SIRT3	11	215458	236431	ENSG00000095303	PTGS1	9	125132824	125157982	0.0091945	14.953	1.09E-09	0.00027803	0.002458	0.56489	0.6882	rs11602954	3.11E-14	0	1.39E-80	0	Cells_Transformed_fibroblasts
ENSG00000142082	SIRT3	11	215458	236431	ENSG00000095303	PTGS1	9	125132824	125157982	0.0091945	14.953	1.09E-09	0.00027803	0.002458	0.56489	0.6882	rs11602954	3.11E-14	0	1.39E-80	0	Nerve_Tibial
ENSG00000142082	SIRT3	11	215458	236431	ENSG00000095303	PTGS1	9	125132824	125157982	0.0091945	14.953	1.09E-09	0.00027803	0.002458	0.56489	0.6882	rs505404	4.38E-17	0	1.05E-94	0	Adrenal_Gland
ENSG00000142082	SIRT3	11	215458	236431	ENSG00000095303	PTGS1	9	125132824	125157982	0.0091945	14.953	1.09E-09	0.00027803	0.002458	0.56489	0.6882	rs505404	4.38E-17	0	1.05E-94	0	Artery_Aorta
ENSG00000142082	SIRT3	11	215458	236431	ENSG00000095303	PTGS1	9	125132824	125157982	0.0091945	14.953	1.09E-09	0.00027803	0.002458	0.56489	0.6882	rs505404	4.38E-17	0	1.05E-94	0	Lung
ENSG00000142082	SIRT3	11	215458	236431	ENSG00000095303	PTGS1	9	125132824	125157982	0.0091945	14.953	1.09E-09	0.00027803	0.002458	0.56489	0.6882	rs505404	4.38E-17	0	1.05E-94	0	Thyroid
ENSG00000157315	TMED6	16	69377151	69377543	ENSG00000255833	TIFAB	5	134787899	134788089	0.010383	10.139	1.12E-09	0.00028419	0.0072705	1.3417	0.24433	rs929843	1.75E-20	0	2.59E-07	0.000833178	Skin_Not_Sun_Exposed_Suprapubic
ENSG00000174885	NLRP6	11	278365	285359	ENSG00000061918	GUCY1B3	4	156680144	156728743	0.011417	7.969	1.19E-09	0.00029991	0.023814	2.472	0.0086751	rs11602954	8.40E-28	0	2.12E-70	0	Nerve_Tibial
ENSG00000174885	NLRP6	11	278365	285359	ENSG00000061918	GUCY1B3	4	156680144	156728743	0.011417	7.969	1.19E-09	0.00029991	0.023814	2.472	0.0086751	rs17655730	5.01E-29	0	5.43E-117	0	Artery_Aorta
ENSG00000174885	NLRP6	11	278365	285359	ENSG00000061918	GUCY1B3	4	156680144	156728743	0.011417	7.969	1.19E-09	0.00029991	0.023814	2.472	0.0086751	rs17655730	5.01E-29	0	5.43E-117	0	Nerve_Tibial
ENSG00000174885	NLRP6	11	278365	285359	ENSG00000061918	GUCY1B3	4	156680144	156728743	0.011417	7.969	1.19E-09	0.00029991	0.023814	2.472	0.0086751	rs17655730	5.01E-29	0	5.43E-117	0	Skin_Sun_Exposed_Lower_leg
ENSG00000174885	NLRP6	11	278365	285359	ENSG00000061918	GUCY1B3	4	156680144	156728743	0.011417	7.969	1.19E-09	0.00029991	0.023814	2.472	0.0086751	rs505404	1.76E-34	0	2.65E-88	0	Nerve_Tibial
ENSG00000174885	NLRP6	11	278365	285359	ENSG00000061918	GUCY1B3	4	156680144	156728743	0.011417	7.969	1.19E-09	0.00029991	0.023814	2.472	0.0086751	rs505404	1.76E-34	0	2.65E-88	0	Prostate
ENSG00000172123	SLFN12	17	33738079	33760302	ENSG00000167671	UBXN6	19	4446046	4457819	0.015045	4.9102	1.20E-09	0.00030116	0.014571	2.2549	0.036292	rs225245	6.11E-162	0	NA	NA	Cells_EBV-transformed_lymphocytes
ENSG00000172123	SLFN12	17	33738079	33760302	ENSG00000167671	UBXN6	19	4446046	4457819	0.015045	4.9102	1.20E-09	0.00030116	0.014571	2.2549	0.036292	rs9915021	2.43E-46	0	NA	NA	Breast_Mammary_Tissue
ENSG00000205045	SLFN12L	17	33800708	33864880	ENSG00000187017	ESPN	1	6484848	6521040	0.011408	7.9624	1.22E-09	0.00030513	0.0094159	1.2411	0.27718	rs8082605	9.11E-17	0	2.49E-31	0	Adipose_Subcutaneous
ENSG00000205045	SLFN12L	17	33800708	33864880	ENSG00000187017	ESPN	1	6484848	6521040	0.011408	7.9624	1.22E-09	0.00030513	0.0094159	1.2411	0.27718	rs8082605	9.11E-17	0	2.49E-31	0	Adipose_Visceral_Omentum
ENSG00000205045	SLFN12L	17	33800708	33864880	ENSG00000187017	ESPN	1	6484848	6521040	0.011408	7.9624	1.22E-09	0.00030513	0.0094159	1.2411	0.27718	rs8082605	9.11E-17	0	2.49E-31	0	Heart_Left_Ventricle
ENSG00000205045	SLFN12L	17	33800708	33864880	ENSG00000187017	ESPN	1	6484848	6521040	0.011408	7.9624	1.22E-09	0.00030513	0.0094159	1.2411	0.27718	rs8082605	9.11E-17	0	2.49E-31	0	Nerve_Tibial
ENSG00000205045	SLFN12L	17	33800708	33864880	ENSG00000187017	ESPN	1	6484848	6521040	0.011408	7.9624	1.22E-09	0.00030513	0.0094159	1.2411	0.27718	rs10512472	5.66E-18	0	4.96E-30	0	Pancreas
ENSG00000239388	ASB14	3	57310556	57326710	ENSG00000172572	PDE3A	12	20522179	20837315	0.010348	10.105	1.22E-09	0.00030513	NA	NA	NA	rs12485738	9.24E-27	0	NA	NA	Artery_Tibial
ENSG00000239388	ASB14	3	57310556	57326710	ENSG00000172572	PDE3A	12	20522179	20837315	0.010348	10.105	1.22E-09	0.00030513	NA	NA	NA	rs1354034	5.07E-54	0	NA	NA	Skin_Not_Sun_Exposed_Suprapubic
ENSG00000205045	SLFN12L	17	33800708	33864880	ENSG00000095321	CRAT	9	131857089	131873083	0.011397	7.9544	1.25E-09	0.00031139	0.011117	1.4679	0.17505	rs8082605	2.34E-17	0	2.49E-31	0	Adipose_Subcutaneous
ENSG00000205045	SLFN12L	17	33800708	33864880	ENSG00000095321	CRAT	9	131857089	131873083	0.011397	7.9544	1.25E-09	0.00031139	0.011117	1.4679	0.17505	rs8082605	2.34E-17	0	2.49E-31	0	Adipose_Visceral_Omentum
ENSG00000205045	SLFN12L	17	33800708	33864880	ENSG00000095321	CRAT	9	131857089	131873083	0.011397	7.9544	1.25E-09	0.00031139	0.011117	1.4679	0.17505	rs8082605	2.34E-17	0	2.49E-31	0	Heart_Left_Ventricle
ENSG00000205045	SLFN12L	17	33800708	33864880	ENSG00000095321	CRAT	9	131857089	131873083	0.011397	7.9544	1.25E-09	0.00031139	0.011117	1.4679	0.17505	rs8082605	2.34E-17	0	2.49E-31	0	Nerve_Tibial
ENSG00000205045	SLFN12L	17	33800708	33864880	ENSG00000095321	CRAT	9	131857089	131873083	0.011397	7.9544	1.25E-09	0.00031139	0.011117	1.4679	0.17505	rs10512472	7.64E-26	0	4.96E-30	0	Pancreas
ENSG00000177951	BET1L	11	167784	207428	ENSG00000144677	CTDSPL	3	37903451	38025960	0.011889	7.2631	1.26E-09	0.00031425	0.026987	2.2944	0.0090618	rs11602954	1.10E-48	0	9.12E-206	0	Adipose_Visceral_Omentum
ENSG00000177951	BET1L	11	167784	207428	ENSG00000144677	CTDSPL	3	37903451	38025960	0.011889	7.2631	1.26E-09	0.00031425	0.026987	2.2944	0.0090618	rs11602954	1.10E-48	0	9.12E-206	0	Artery_Aorta
ENSG00000177951	BET1L	11	167784	207428	ENSG00000144677	CTDSPL	3	37903451	38025960	0.011889	7.2631	1.26E-09	0.00031425	0.026987	2.2944	0.0090618	rs11602954	1.10E-48	0	9.12E-206	0	Cells_Transformed_fibroblasts
ENSG00000177951	BET1L	11	167784	207428	ENSG00000144677	CTDSPL	3	37903451	38025960	0.011889	7.2631	1.26E-09	0.00031425	0.026987	2.2944	0.0090618	rs11602954	1.10E-48	0	9.12E-206	0	Pituitary
ENSG00000177951	BET1L	11	167784	207428	ENSG00000144677	CTDSPL	3	37903451	38025960	0.011889	7.2631	1.26E-09	0.00031425	0.026987	2.2944	0.0090618	rs11602954	1.10E-48	0	9.12E-206	0	Prostate
ENSG00000177951	BET1L	11	167784	207428	ENSG00000144677	CTDSPL	3	37903451	38025960	0.011889	7.2631	1.26E-09	0.00031425	0.026987	2.2944	0.0090618	rs11602954	1.10E-48	0	9.12E-206	0	Small_Intestine_Terminal_Ileum
ENSG00000177951	BET1L	11	167784	207428	ENSG00000144677	CTDSPL	3	37903451	38025960	0.011889	7.2631	1.26E-09	0.00031425	0.026987	2.2944	0.0090618	rs11602954	1.10E-48	0	9.12E-206	0	Whole_Blood
ENSG00000177951	BET1L	11	167784	207428	ENSG00000144677	CTDSPL	3	37903451	38025960	0.011889	7.2631	1.26E-09	0.00031425	0.026987	2.2944	0.0090618	rs17655730	1.49E-41	0	2.96E-166	0	Prostate
ENSG00000177951	BET1L	11	167784	207428	ENSG00000144677	CTDSPL	3	37903451	38025960	0.011889	7.2631	1.26E-09	0.00031425	0.026987	2.2944	0.0090618	rs505404	1.75E-49	0	3.73E-187	0	Small_Intestine_Terminal_Ileum
ENSG00000177951	BET1L	11	167784	207428	ENSG00000144677	CTDSPL	3	37903451	38025960	0.011889	7.2631	1.26E-09	0.00031425	0.026987	2.2944	0.0090618	rs505404	1.75E-49	0	3.73E-187	0	Whole_Blood
ENSG00000177951	BET1L	11	167784	207428	ENSG00000144677	CTDSPL	3	37903451	38025960	0.011889	7.2631	1.26E-09	0.00031425	0.026987	2.2944	0.0090618	rs3782123	4.11E-07	0.00366492	NA	NA	Heart_Atrial_Appendage
ENSG00000141150	RASL10B	17	34058668	34070540	ENSG00000238243	OR2W3	1	248058859	248060449	0.011872	7.2525	1.31E-09	0.00032432	0.0057717	0.758	0.62279	rs9915021	7.68E-06	0.04691593	NA	NA	Cells_Transformed_fibroblasts
ENSG00000142082	SIRT3	11	215458	236431	ENSG00000174175	SELP	1	169558087	169599431	0.0090958	14.791	1.39E-09	0.00034213	0.0042142	0.9702	0.42293	rs11602954	2.72E-34	0	1.39E-80	0	Adipose_Subcutaneous
ENSG00000142082	SIRT3	11	215458	236431	ENSG00000174175	SELP	1	169558087	169599431	0.0090958	14.791	1.39E-09	0.00034213	0.0042142	0.9702	0.42293	rs11602954	2.72E-34	0	1.39E-80	0	Artery_Tibial
ENSG00000142082	SIRT3	11	215458	236431	ENSG00000174175	SELP	1	169558087	169599431	0.0090958	14.791	1.39E-09	0.00034213	0.0042142	0.9702	0.42293	rs11602954	2.72E-34	0	1.39E-80	0	Breast_Mammary_Tissue
ENSG00000142082	SIRT3	11	215458	236431	ENSG00000174175	SELP	1	169558087	169599431	0.0090958	14.791	1.39E-09	0.00034213	0.0042142	0.9702	0.42293	rs11602954	2.72E-34	0	1.39E-80	0	Cells_Transformed_fibroblasts
ENSG00000142082	SIRT3	11	215458	236431	ENSG00000174175	SELP	1	169558087	169599431	0.0090958	14.791	1.39E-09	0.00034213	0.0042142	0.9702	0.42293	rs11602954	2.72E-34	0	1.39E-80	0	Nerve_Tibial
ENSG00000142082	SIRT3	11	215458	236431	ENSG00000174175	SELP	1	169558087	169599431	0.0090958	14.791	1.39E-09	0.00034213	0.0042142	0.9702	0.42293	rs505404	2.87E-40	0	1.05E-94	0	Adrenal_Gland
ENSG00000142082	SIRT3	11	215458	236431	ENSG00000174175	SELP	1	169558087	169599431	0.0090958	14.791	1.39E-09	0.00034213	0.0042142	0.9702	0.42293	rs505404	2.87E-40	0	1.05E-94	0	Artery_Aorta
ENSG00000142082	SIRT3	11	215458	236431	ENSG00000174175	SELP	1	169558087	169599431	0.0090958	14.791	1.39E-09	0.00034213	0.0042142	0.9702	0.42293	rs505404	2.87E-40	0	1.05E-94	0	Lung
ENSG00000142082	SIRT3	11	215458	236431	ENSG00000174175	SELP	1	169558087	169599431	0.0090958	14.791	1.39E-09	0.00034213	0.0042142	0.9702	0.42293	rs505404	2.87E-40	0	1.05E-94	0	Thyroid
ENSG00000172716	SLFN11	17	33677324	33700720	ENSG00000167671	UBXN6	19	4446046	4457819	0.013686	5.5793	1.40E-09	0.00034525	0.022533	1.7462	0.052915	rs225245	6.11E-162	0	5.77E-07	0.001884469	Cells_Transformed_fibroblasts
ENSG00000172716	SLFN11	17	33677324	33700720	ENSG00000167671	UBXN6	19	4446046	4457819	0.013686	5.5793	1.40E-09	0.00034525	0.022533	1.7462	0.052915	rs8082605	3.08E-60	0	NA	NA	Adrenal_Gland

ENSG00000205045	SLFN12L	17	33800708	33864880	ENSG00000149260	CAPN5	11	76777979	76837201	0.011332	7.909	1.44E-09	0.00035381	0.011317	1.4946	0.16543	rs10512472	1.11E-06	0.00889928	4.96E-30	0	Pancreas
ENSG00000205045	SLFN12L	17	33800708	33864880	ENSG00000137193	PIM1	6	37137979	37143202	0.011326	7.9048	1.46E-09	0.0003581	0.018166	2.4158	0.018719	rs8082605	2.56E-06	0.01876826	2.49E-31	0	Adipose_Subcutaneous
ENSG00000205045	SLFN12L	17	33800708	33864880	ENSG00000137193	PIM1	6	37137979	37143202	0.011326	7.9048	1.46E-09	0.0003581	0.018166	2.4158	0.018719	rs8082605	2.56E-06	0.01876826	2.49E-31	0	Adipose_Visceral_Omentum
ENSG00000205045	SLFN12L	17	33800708	33864880	ENSG00000137193	PIM1	6	37137979	37143202	0.011326	7.9048	1.46E-09	0.0003581	0.018166	2.4158	0.018719	rs8082605	2.56E-06	0.01876826	2.49E-31	0	Heart_Left_Ventricle
ENSG00000205045	SLFN12L	17	33800708	33864880	ENSG00000137193	PIM1	6	37137979	37143202	0.011326	7.9048	1.46E-09	0.0003581	0.018166	2.4158	0.018719	rs8082605	2.56E-06	0.01876826	2.49E-31	0	Nerve_Tibial
ENSG00000172660	TAF15	17	34136459	34174246	ENSG00000123908	AGO2	8	141541264	141645718	0.010792	8.7841	1.52E-09	0.00037184	NA	NA	NA	rs9915021	4.09E-08	0.00058148	NA	NA	Skin_Not_Sun_Exposed_Suprapubic
ENSG00000205045	SLFN12L	17	33800708	33864880	ENSG00000177666	PNPLA2	11	818902	825573	0.011302	7.8875	1.54E-09	0.00037645	0.0055846	0.73329	0.64378	rs8082605	1.26E-06	0.01004391	2.49E-31	0	Adipose_Subcutaneous
ENSG00000205045	SLFN12L	17	33800708	33864880	ENSG00000177666	PNPLA2	11	818902	825573	0.011302	7.8875	1.54E-09	0.00037645	0.0055846	0.73329	0.64378	rs8082605	1.26E-06	0.01004391	2.49E-31	0	Adipose_Visceral_Omentum
ENSG00000205045	SLFN12L	17	33800708	33864880	ENSG00000177666	PNPLA2	11	818902	825573	0.011302	7.8875	1.54E-09	0.00037645	0.0055846	0.73329	0.64378	rs8082605	1.26E-06	0.01004391	2.49E-31	0	Heart_Left_Ventricle
ENSG00000205045	SLFN12L	17	33800708	33864880	ENSG00000177666	PNPLA2	11	818902	825573	0.011302	7.8875	1.54E-09	0.00037645	0.0055846	0.73329	0.64378	rs8082605	1.26E-06	0.01004391	2.49E-31	0	Nerve_Tibial
ENSG00000006125	AP2B1	17	33913918	34053436	ENSG00000136732	GYPC	2	127413509	127454246	0.011289	7.8787	1.58E-09	0.00038608	0.012952	2.001	0.062999	rs10512472	1.09E-24	0	3.62E-14	0	Heart_Left_Ventricle
ENSG00000100412	ACO2	22	41865129	41921352	ENSG00000175556	LONRF3	X	118108581	118156888	0.01022	9.9783	1.63E-09	0.00039616	0.011942	2.7709	0.026239	rs727563	1.92E-27	0	NA	NA	Ovary
ENSG00000142082	SIRT3	11	215458	236431	ENSG00000173210	ABLIM3	5	148521046	148640105	0.0090242	14.673	1.64E-09	0.00039673	0.0075026	1.733	0.14054	rs11602954	2.06E-35	0	1.39E-80	0	Adipose_Subcutaneous
ENSG00000142082	SIRT3	11	215458	236431	ENSG00000173210	ABLIM3	5	148521046	148640105	0.0090242	14.673	1.64E-09	0.00039673	0.0075026	1.733	0.14054	rs11602954	2.06E-35	0	1.39E-80	0	Artery_Tibial
ENSG00000142082	SIRT3	11	215458	236431	ENSG00000173210	ABLIM3	5	148521046	148640105	0.0090242	14.673	1.64E-09	0.00039673	0.0075026	1.733	0.14054	rs11602954	2.06E-35	0	1.39E-80	0	Breast_Mammary_Tissue
ENSG00000142082	SIRT3	11	215458	236431	ENSG00000173210	ABLIM3	5	148521046	148640105	0.0090242	14.673	1.64E-09	0.00039673	0.0075026	1.733	0.14054	rs11602954	2.06E-35	0	1.39E-80	0	Cells_Transformed_fibroblasts
ENSG00000142082	SIRT3	11	215458	236431	ENSG00000173210	ABLIM3	5	148521046	148640105	0.0090242	14.673	1.64E-09	0.00039673	0.0075026	1.733	0.14054	rs11602954	2.06E-35	0	1.39E-80	0	Nerve_Tibial
ENSG00000142082	SIRT3	11	215458	236431	ENSG00000173210	ABLIM3	5	148521046	148640105	0.0090242	14.673	1.64E-09	0.00039673	0.0075026	1.733	0.14054	rs505404	3.10E-39	0	1.05E-94	0	Adrenal_Gland
ENSG00000142082	SIRT3	11	215458	236431	ENSG00000173210	ABLIM3	5	148521046	148640105	0.0090242	14.673	1.64E-09	0.00039673	0.0075026	1.733	0.14054	rs505404	3.10E-39	0	1.05E-94	0	Artery_Aorta
ENSG00000142082	SIRT3	11	215458	236431	ENSG00000173210	ABLIM3	5	148521046	148640105	0.0090242	14.673	1.64E-09	0.00039673	0.0075026	1.733	0.14054	rs505404	3.10E-39	0	1.05E-94	0	Lung
ENSG00000142082	SIRT3	11	215458	236431	ENSG00000173210	ABLIM3	5	148521046	148640105	0.0090242	14.673	1.64E-09	0.00039673	0.0075026	1.733	0.14054	rs505404	3.10E-39	0	1.05E-94	0	Thyroid
ENSG00000163946	FAM208A	3	56658507	56717265	ENSG00000119862	LGALSL	2	64681103	64688515	0.011761	7.184	1.67E-09	0.00040261	NA	NA	NA	rs12485738	1.17E-118	0	NA	NA	Esophagus_Mucosa
ENSG00000163946	FAM208A	3	56658507	56717265	ENSG00000119862	LGALSL	2	64681103	64688515	0.011761	7.184	1.67E-09	0.00040261	NA	NA	NA	rs1354034	7.05E-227	0	NA	NA	Heart_Atrial_Appendage
ENSG00000132139	GAS2L2	17	34071530	34079897	ENSG00000196961	AP2A1	19	50270225	50309510	0.011757	7.1812	1.69E-09	0.00040616	0.0086585	0.99678	0.43683	rs9915021	1.56E-11	0	NA	NA	Small_Intestine_Terminal_Ileum
ENSG00000168374	ARF4	3	57557090	57583947	ENSG00000101335	MYL9	20	35169887	35178228	0.01018	9.9387	1.79E-09	0.00043091	0.024425	4.5867	0.00038721	rs17825630	9.14E-22	0	NA	NA	Artery_Tibial
ENSG00000168374	ARF4	3	57557090	57583947	ENSG00000101335	MYL9	20	35169887	35178228	0.01018	9.9387	1.79E-09	0.00043091	0.024425	4.5867	0.00038721	rs17825630	9.14E-22	0	NA	NA	Heart_Atrial_Appendage
ENSG00000154760	SLFN13	17	33762115	33775856	ENSG00000137193	PIM1	6	37137979	37143202	0.013124	5.8345	1.80E-09	0.00043214	0.01616	1.2442	0.24747	rs225245	2.86E-14	0	NA	NA	Liver
ENSG00000154760	SLFN13	17	33762115	33775856	ENSG00000137193	PIM1	6	37137979	37143202	0.013124	5.8345	1.80E-09	0.00043214	0.01616	1.2442	0.24747	rs8082605	2.56E-06	0.01876826	2.08E-19	0	Artery_Tibial
ENSG00000154760	SLFN13	17	33762115	33775856	ENSG00000137193	PIM1	6	37137979	37143202	0.013124	5.8345	1.80E-09	0.00043214	0.01616	1.2442	0.24747	rs8082605	2.56E-06	0.01876826	2.08E-19	0	Esophagus_Mucosa
ENSG00000154760	SLFN13	17	33762115	33775856	ENSG00000137193	PIM1	6	37137979	37143202	0.013124	5.8345	1.80E-09	0.00043214	0.01616	1.2442	0.24747	rs8082605	2.56E-06	0.01876826	2.08E-19	0	Nerve_Tibial
ENSG00000154760	SLFN13	17	33762115	33775856	ENSG00000137193	PIM1	6	37137979	37143202	0.013124	5.8345	1.80E-09	0.00043214	0.01616	1.2442	0.24747	rs8082605	2.56E-06	0.01876826	2.08E-19	0	Pituitary
ENSG00000154760	SLFN13	17	33762115	33775856	ENSG00000118046	STK11	19	1189406	1226662	0.013121	5.8331	1.81E-09	0.00043444	0.010337	0.79119	0.66001	rs225245	1.03E-13	0	NA	NA	Liver
ENSG00000188452	CERKL	2	182401403	182521843	ENSG00000096006	CRISP3	6	49695097	49712150	0.0095919	11.702	1.86E-09	0.00044321	0.014399	3.3492	0.009837	rs1375493	1.23E-27	0	3.47E-111	0	Adrenal_Gland
ENSG00000188452	CERKL	2	182401403	182521843	ENSG00000096006	CRISP3	6	49695097	49712150	0.0095919	11.702	1.86E-09	0.00044321	0.014399	3.3492	0.009837	rs1375493	1.23E-27	0	3.47E-111	0	Whole_Blood
ENSG00000188452	CERKL	2	182401403	182521843	ENSG00000096006	CRISP3	6	49695097	49712150	0.0095919	11.702	1.86E-09	0.00044321	0.014399	3.3492	0.009837	rs2124440	8.36E-27	0	6.92E-104	0	Adrenal_Gland
ENSG00000188452	CERKL	2	182401403	182521843	ENSG00000096006	CRISP3	6	49695097	49712150	0.0095919	11.702	1.86E-09	0.00044321	0.014399	3.3492	0.009837	rs2124440	8.36E-27	0	6.92E-104	0	Whole_Blood
ENSG00000188452	CERKL	2	182401403	182521843	ENSG00000096006	CRISP3	6	49695097	49712150	0.0095919	11.702	1.86E-09	0.00044321	0.014399	3.3492	0.009837	rs1449263	6.73E-26	0	1.10E-101	0	Whole_Blood
ENSG00000092871	RFFL	17	33341759	33416338	ENSG00000029534	ANK1	8	41510739	41754280	0.010697	8.7057	1.89E-09	0.00044952	0.0066701	0.76634	0.63264	rs9915021	6.17E-12	0	NA	NA	Adipose_Subcutaneous
ENSG00000092871	RFFL	17	33341759	33416338	ENSG00000029534	ANK1	8	41510739	41754280	0.010697	8.7057	1.89E-09	0.00044952	0.0066701	0.76634	0.63264	rs9915021	6.17E-12	0	NA	NA	Artery_Tibial
ENSG00000177963	RIC8A	11	207511	215113	ENSG00000204420	C6orf25	6	31691121	31694491	0.010143	9.9026	1.95E-09	0.00046324	0.0078442	0.9023	0.51377	rs11602954	2.23E-16	0	7.55E-62	0	Adipose_Subcutaneous
ENSG00000177963	RIC8A	11	207511	215113	ENSG00000204420	C6orf25	6	31691121	31694491	0.010143	9.9026	1.95E-09	0.00046324	0.0078442	0.9023	0.51377	rs11602954	2.23E-16	0	7.55E-62	0	Artery_Aorta
ENSG00000177963	RIC8A	11	207511	215113	ENSG00000204420	C6orf25	6	31691121	31694491	0.010143	9.9026	1.95E-09	0.00046324	0.0078442	0.9023	0.51377	rs11602954	2.23E-16	0	7.55E-62	0	Artery_Tibial
ENSG00000177963	RIC8A	11	207511	215113	ENSG00000204420	C6orf25	6	31691121	31694491	0.010143	9.9026	1.95E-09	0.00046324	0.0078442	0.9023	0.51377	rs11602954	2.23E-16	0	7.55E-62	0	Brain_Caudate_basal_ganglia
ENSG00000177963	RIC8A	11	207511	215113	ENSG00000204420	C6orf25	6	31691121	31694491	0.010143	9.9026	1.95E-09	0.00046324	0.0078442	0.9023	0.51377	rs11602954	2.23E-16	0	7.55E-62	0	Brain_Frontal_Cortex_BA9
ENSG00000177963	RIC8A	11	207511	215113	ENSG00000204420	C6orf25	6	31691121	31694491	0.010143	9.9026	1.95E-09	0.00046324	0.0078442	0.9023	0.51377	rs11602954	2.23E-16	0	7.55E-62	0	Colon_Sigmoid
ENSG00000177963	RIC8A	11	207511	215113	ENSG00000204420	C6orf25	6	31691121	31694491	0.010143	9.9026	1.95E-09	0.00046324	0.0078442	0.9023	0.51377	rs11602954	2.23E-16	0	7.55E-62	0	Colon_Transverse
ENSG00000177963	RIC8A	11	207511	215113	ENSG00000204420	C6orf25	6	31691121	31694491	0.010143	9.9026	1.95E-09	0.00046324	0.0078442	0.9023	0.51377	rs11602954	2.23E-16	0	7.55E-62	0	Esophagus_Gastroesophageal_Junction
ENSG00000177963	RIC8A	11	207511	215113	ENSG00000204420	C6orf25	6	31691121	31694491	0.010143	9.9026	1.95E-09	0.00046324	0.0078442	0.9023	0.51377	rs11602954	2.23E-16	0	7.55E-62	0	Esophagus_Mucosa
ENSG00000177963	RIC8A	11	207511	215113	ENSG00000204420	C6orf25	6	31691121	31694491	0.010143	9.9026	1.95E-09	0.00046324	0.0078442	0.9023	0.51377	rs11602954	2.23E-16	0	7.55E-62	0	Esophagus_Muscularis
ENSG00000177963	RIC8A	11	207511	215113	ENSG00000204420	C6orf25	6	31691121	31694491	0.010143	9.9026	1.95E-09	0.00046324	0.0078442	0.9023	0.51377	rs11602954	2.23E-16	0	7.55E-62	0	Lung
ENSG00000177963	RIC8A	11	207511	215113	ENSG00000204420	C6orf25	6	31691121	31694491	0.010143	9.9026	1.95E-09	0.00046324	0.0078442	0.9023	0.51377	rs11602954	2.23E-16	0	7.55E-62	0	Muscle_Skeletal
ENSG00000177963	RIC8A	11	207511	215113	ENSG00000204420	C6orf25	6	31691121	31694491	0.010143	9.9026	1.95E-09	0.00046324	0.0078442	0.9023	0.51377	rs11602954	2.23E-16	0	7.55E-62	0	Nerve_Tibial
ENSG00000177963	RIC8A	11	207511	215113	ENSG00000204420	C6orf25	6	31691121	31694491	0.010143	9.9026	1.95E-09	0.00046324	0.0078442	0.9023	0.51377	rs11602954	2.23E-16	0	7.55E-62	0	Skin_Not_Sun_Exposed_Suprapubic
ENSG00000177963	RIC8A	11	207511	215113	ENSG00000204420	C6orf25	6	31691121	31694491	0.010143	9.9026	1.95E-09	0.00046324	0.0078442	0.9023	0.51377	rs11602954	2.23E-16	0	7.55E-62	0	Skin_Sun_Exposed_Lower_leg
ENSG00000177963	RIC8A	11	207511	215113	ENSG00000204420	C6orf25	6	31691121	31694491	0.010143	9.9026	1.95E-09	0.00046324	0.0078442	0.9023	0.51377	rs11602954	2.23E-16	0	7.55E-62	0	Thyroid
ENSG00000177963	RIC8A	11	207511	215113	ENSG00000204420	C6orf25	6	31691121	31694491	0.010143	9.9026	1.95E-09	0.00046324	0.0078442	0.9023	0.51377	rs17655730	8.25E-20	0	5.10E-36	0	Adipose_Subcutaneous
ENSG00000177963	RIC8A	11	207511	215113	ENSG00000204420	C6orf25	6	31691121	31694491	0.010143	9.9026	1.95E-09	0.00046324	0.0078442	0.9023	0.51377	rs17655730	8.25E-20	0	5.10E-36	0	Esophagus_Mucosa
ENSG00000177963	RIC8A	11	207511	215113	ENSG00000204420	C6orf25	6	31691121	31694491	0.010143	9.9026	1.95E-09	0.00046324	0.0078442	0.9023	0.51377	rs17655730	8.25E-20	0	5.10E-36	0	Esophagus_Muscularis
ENSG00000177963	RIC8A	11	207511	215113	ENSG00000204420	C6orf25	6	31691121	31694491	0.010143	9.9026	1.95E-09	0.00046324	0.0078442	0.9023	0.51377	rs17655730	8.25E-20	0	5.10E-36	0	Heart_Left_Ventricle
ENSG00000177963	RIC8A	11	207511	215113	ENSG00000204420	C6orf25	6	31691121	31694491	0.010143	9.9026	1.95E-09	0.00046324	0.0078442	0.9023	0.51377	rs17655730	8.25E-20	0	5.10E-36	0	Ovary
ENSG00000177963	RIC8A	11	207511	215113	ENSG00000204420	C6orf25	6	31691121	31694491	0.010143	9.9026	1.95E-09	0.00046324	0.0078442	0.9023	0.51377	rs17655730	8.25E-20	0	5.10E-36	0	Pancreas
ENSG00000177963	RIC8A	11	207511	215113	ENSG00000204420	C6orf25	6	31691121	31694491	0.010143	9.9026	1.95E-09	0.00046324	0.0078442	0.9023	0.51377	rs17655730	8.25E-20	0	5.10E-36	0	Skin_Not_Sun_Exposed_Suprapubic
ENSG00000177963	RIC8A	11	207511	215113	ENSG00000204420	C6orf25	6	31691121	31694491	0.010143	9.9026	1.95E-09	0.00046324	0.0078442	0.9023	0.51377	rs505404	9.44E-22	0	3.66E-47	0	Artery_Aorta
ENSG00000177963	RIC8A	11	207511	215113	ENSG00000204420	C6orf25	6	31691121	31694491	0.010143	9.9026	1.95E-09	0.00046324	0.0078442	0.9023	0.51377	rs505404	9.44E-22	0	3.66E-47	0	Thyroid
ENSG00000108733	PEX12	17	33901814	33905882	ENSG00000143774	GUK1	1	228327663	228336685	0.010141	9.9011	1.96E-09	0.00046434	0.0074923	1.383	0.22818	rs9915021	1.78E-10	0	NA	NA	Brain_Caudate_basal_ganglia
ENSG00000108733	PEX12	17	33901814	33905882	ENSG00000143774	GUK1	1	228327663	228336685	0.010141	9.9011	1.96E-09	0.00046434	0.0074923	1.383	0.22818	rs9915021	1.78E-10	0	NA	NA	Breast_Mammary_Tissue
ENSG00000108733	PEX12	17	33901814	33905882	ENSG00000143774	GUK1	1	228327663	228336685	0.010141	9.9011	1.96E-09	0.00046434	0.0074923	1.383	0.22818	rs10512472	1.38E-20	0	1.06E-12	0	Brain_Caudate_basal_ganglia
ENSG00000174885	NLRP6	11	278365	285359	ENSG00000124491	F13A1	6	6144318	6321246	0.01117	7.7941	2.07E-09	0.00048868	0.02017	2.0859	0.028303	rs11602954	7.25E-48	0	2.12E-70	0	Nerve_Tibial
ENSG00000174885	NLRP6	11	278365	285359	ENSG00000124491	F13A1	6	6144318	6321246	0.01117	7.7941	2.07E-09	0.00048868	0.02017	2.0859	0.028303	rs17655730	3.37E-44	0	5.43E-117	0	Artery_Aorta
ENSG00000174885	NLRP6	11	278365	285359	ENSG00000124491	F13A1	6	6144318	6321246	0.01117	7.7941	2.07E-09	0.00048868	0.02017	2.0859	0.028303	rs17655730	3.37E-44	0	5.43E-117	0	Nerve_Tibial
ENSG00000174885	NLRP6	11	278365	285359	ENSG00000124491	F13A1	6	6144318	6321246	0.01117	7.7941	2.07E-09	0.00048868	0.02017	2.0859	0.028303	rs17655730	3.37E-44	0	5.43E-117	0	Skin_Sun_Exposed_Lower_leg
ENSG00000174885	NLRP6	11	278365	285359	ENSG00000124491	F13A1	6	6144318	6321246	0.01117	7.7941	2.07E-09	0.00048868	0.02017	2.0859	0.028303	rs505404	5.93E-51	0	2.65E-88	0	Nerve_Tibial
ENSG00000174885	NLRP6	11	278365	285359	ENSG00000124491	F13A1	6	6144318	6321246	0.01117	7.7941	2.07E-09	0.00048868	0.02017	2.0859	0.028303	rs505404	5.93E-51	0	2.65E-88	0	Prostate
ENSG00000132139	GAS2L2	17	34071530	34079897	ENSG00000144567	FAM134A	2	220042939	220050201	0.011662	7.1225	2.08E-09	0.00048993	0.015569	1.8049	0.072535	rs9915021	2.69E-24	0	NA	NA	Small_Intestine_Terminal_Ileum
ENSG00000167914	GSDMA	17	38119226	38134019	ENSG00000110080	ST3GAL4	11	126225535	126310239	0.00822	20.037	2.16E-09	0.00050663	0.01015	4.7117	0.0092075	rs3859192	1.04E-10	0	6.22E-13	0	Adipose_Subcutaneous
ENSG00000167914	GSDMA	17	38119226	38134019	ENSG00000110080	ST3GAL4	11	126225535	126310239	0.00822	20.037	2.16E-09	0.00050663	0.01015	4.7117	0.0092075	rs3859192	1.04E-10	0	6.22E-13	0	Adipose_Visceral_Omentum
ENSG00000167914	GSDMA	17	38119226	38134019	ENSG00000110080	ST3GAL4	11	126225535	126310239	0.00822	20.037	2.16E-09	0.00050663	0.01015	4.7117	0.0092075	rs3859192	1.04E-10	0	6.22E-13	0	Adrenal_Gland
ENSG00000167914	GSDMA	17	38119226	38134019	ENSG00000110080	ST3GAL4	11	126225535	126310239	0.00822	20.037	2.16E-09	0.00050663	0.01015	4.7117	0.0092075	rs3859192	1.04E-10	0	6.22E-13	0	Artery_Aorta
ENSG00000167914	GSDMA	17	38119226	38134019	ENSG00000110080	ST3GAL4	11	126225535	126310239	0.00822	20.037	2.16E-09	0.00050663	0.01015	4.7117	0.0092075	rs3859192	1.04E-10	0	6.22E-13	0	Artery_Coronary
ENSG00000167914	GSDMA	17	38119226	38134019	ENSG00000110080	ST3GAL4	11	126225535	126310239	0.00822	20.037	2.16E-09	0.00050663	0.01015	4.7117	0.0092075	rs3859192	1.04E-10	0	6.22E-13	0	Artery_Tibial
ENSG00000167914	GSDMA	17	38119226	38134019	ENSG00000110080	ST3GAL4	11	126225535	126310239	0.00822	20.037	2.16E-09	0.00050663	0.01015	4.7117	0.0092075	rs3859192	1.04E-10	0	6.22E-13	0	Breast_Mammary_Tissue
ENSG00000167914	GSDMA	17	38119226	38134019	ENSG00000110080	ST3GAL4	11	126225535	126310239	0.00822	20.037	2.16E-09	0.00050663	0.01015	4.7117	0.0092075	rs3859192	1.04E-10	0	6.22E-13	0	Cells_Transformed_fibroblasts

ENSG00000167914	GSDMA	17	38119226	38134019	ENSG00000110080	ST3GAL4	11	126225535	126310239	0.00822	20.037	2.16E-09	0.00050663	0.01015	4.7117	0.0092075	rs3859192	1.04E-10	0	6.22E-13	0	Colon_Sigmoid
ENSG00000167914	GSDMA	17	38119226	38134019	ENSG00000110080	ST3GAL4	11	126225535	126310239	0.00822	20.037	2.16E-09	0.00050663	0.01015	4.7117	0.0092075	rs3859192	1.04E-10	0	6.22E-13	0	Colon_Transverse
ENSG00000167914	GSDMA	17	38119226	38134019	ENSG00000110080	ST3GAL4	11	126225535	126310239	0.00822	20.037	2.16E-09	0.00050663	0.01015	4.7117	0.0092075	rs3859192	1.04E-10	0	6.22E-13	0	Esophagus_Gastroesophageal_Junction
ENSG00000167914	GSDMA	17	38119226	38134019	ENSG00000110080	ST3GAL4	11	126225535	126310239	0.00822	20.037	2.16E-09	0.00050663	0.01015	4.7117	0.0092075	rs3859192	1.04E-10	0	6.22E-13	0	Esophagus_Muscularis
ENSG00000167914	GSDMA	17	38119226	38134019	ENSG00000110080	ST3GAL4	11	126225535	126310239	0.00822	20.037	2.16E-09	0.00050663	0.01015	4.7117	0.0092075	rs3859192	1.04E-10	0	6.22E-13	0	Heart_Atrial_Appendage
ENSG00000167914	GSDMA	17	38119226	38134019	ENSG00000110080	ST3GAL4	11	126225535	126310239	0.00822	20.037	2.16E-09	0.00050663	0.01015	4.7117	0.0092075	rs3859192	1.04E-10	0	6.22E-13	0	Heart_Left_Ventricle
ENSG00000167914	GSDMA	17	38119226	38134019	ENSG00000110080	ST3GAL4	11	126225535	126310239	0.00822	20.037	2.16E-09	0.00050663	0.01015	4.7117	0.0092075	rs3859192	1.04E-10	0	6.22E-13	0	Liver
ENSG00000167914	GSDMA	17	38119226	38134019	ENSG00000110080	ST3GAL4	11	126225535	126310239	0.00822	20.037	2.16E-09	0.00050663	0.01015	4.7117	0.0092075	rs3859192	1.04E-10	0	6.22E-13	0	Lung
ENSG00000167914	GSDMA	17	38119226	38134019	ENSG00000110080	ST3GAL4	11	126225535	126310239	0.00822	20.037	2.16E-09	0.00050663	0.01015	4.7117	0.0092075	rs3859192	1.04E-10	0	6.22E-13	0	Muscle_Skeletal
ENSG00000167914	GSDMA	17	38119226	38134019	ENSG00000110080	ST3GAL4	11	126225535	126310239	0.00822	20.037	2.16E-09	0.00050663	0.01015	4.7117	0.0092075	rs3859192	1.04E-10	0	6.22E-13	0	Nerve_Tibial
ENSG00000167914	GSDMA	17	38119226	38134019	ENSG00000110080	ST3GAL4	11	126225535	126310239	0.00822	20.037	2.16E-09	0.00050663	0.01015	4.7117	0.0092075	rs3859192	1.04E-10	0	6.22E-13	0	Ovary
ENSG00000167914	GSDMA	17	38119226	38134019	ENSG00000110080	ST3GAL4	11	126225535	126310239	0.00822	20.037	2.16E-09	0.00050663	0.01015	4.7117	0.0092075	rs3859192	1.04E-10	0	6.22E-13	0	Pancreas
ENSG00000167914	GSDMA	17	38119226	38134019	ENSG00000110080	ST3GAL4	11	126225535	126310239	0.00822	20.037	2.16E-09	0.00050663	0.01015	4.7117	0.0092075	rs3859192	1.04E-10	0	6.22E-13	0	Small_Intestine_Terminal_Ileum
ENSG00000167914	GSDMA	17	38119226	38134019	ENSG00000110080	ST3GAL4	11	126225535	126310239	0.00822	20.037	2.16E-09	0.00050663	0.01015	4.7117	0.0092075	rs3859192	1.04E-10	0	6.22E-13	0	Spleen
ENSG00000167914	GSDMA	17	38119226	38134019	ENSG00000110080	ST3GAL4	11	126225535	126310239	0.00822	20.037	2.16E-09	0.00050663	0.01015	4.7117	0.0092075	rs3859192	1.04E-10	0	6.22E-13	0	Stomach
ENSG00000167914	GSDMA	17	38119226	38134019	ENSG00000110080	ST3GAL4	11	126225535	126310239	0.00822	20.037	2.16E-09	0.00050663	0.01015	4.7117	0.0092075	rs3859192	1.04E-10	0	6.22E-13	0	Thyroid
ENSG00000167914	GSDMA	17	38119226	38134019	ENSG00000110080	ST3GAL4	11	126225535	126310239	0.00822	20.037	2.16E-09	0.00050663	0.01015	4.7117	0.0092075	rs3859192	1.04E-10	0	6.22E-13	0	Whole_Blood
ENSG00000167914	GSDMA	17	38119226	38134019	ENSG00000110080	ST3GAL4	11	126225535	126310239	0.00822	20.037	2.16E-09	0.00050663	0.01015	4.7117	0.0092075	rs8078723	4.29E-14	0	6.97E-07	0.002194627	Breast_Mammary_Tissue
ENSG00000167914	GSDMA	17	38119226	38134019	ENSG00000110080	ST3GAL4	11	126225535	126310239	0.00822	20.037	2.16E-09	0.00050663	0.01015	4.7117	0.0092075	rs8078723	4.29E-14	0	6.97E-07	0.002194627	Esophagus_Gastroesophageal_Junction
ENSG00000167914	GSDMA	17	38119226	38134019	ENSG00000110080	ST3GAL4	11	126225535	126310239	0.00822	20.037	2.16E-09	0.00050663	0.01015	4.7117	0.0092075	rs4794820	6.62E-06	0.04163105	2.26E-66	0	Artery_Tibial
ENSG00000167914	GSDMA	17	38119226	38134019	ENSG00000110080	ST3GAL4	11	126225535	126310239	0.00822	20.037	2.16E-09	0.00050663	0.01015	4.7117	0.0092075	rs4794820	6.62E-06	0.04163105	2.26E-66	0	Cells_Transformed_fibroblasts
ENSG00000167914	GSDMA	17	38119226	38134019	ENSG00000110080	ST3GAL4	11	126225535	126310239	0.00822	20.037	2.16E-09	0.00050663	0.01015	4.7117	0.0092075	rs4794822	2.95E-13	0	3.02E-06	0.008756123	Breast_Mammary_Tissue
ENSG00000167914	GSDMA	17	38119226	38134019	ENSG00000110080	ST3GAL4	11	126225535	126310239	0.00822	20.037	2.16E-09	0.00050663	0.01015	4.7117	0.0092075	rs4794822	2.95E-13	0	3.02E-06	0.008756123	Cells_Transformed_fibroblasts
ENSG00000167914	GSDMA	17	38119226	38134019	ENSG00000110080	ST3GAL4	11	126225535	126310239	0.00822	20.037	2.16E-09	0.00050663	0.01015	4.7117	0.0092075	rs4794822	2.95E-13	0	3.02E-06	0.008756123	Esophagus_Gastroesophageal_Junction
ENSG00000167914	GSDMA	17	38119226	38134019	ENSG00000110080	ST3GAL4	11	126225535	126310239	0.00822	20.037	2.16E-09	0.00050663	0.01015	4.7117	0.0092075	rs4794822	2.95E-13	0	3.02E-06	0.008756123	Esophagus_Muscularis
ENSG00000167914	GSDMA	17	38119226	38134019	ENSG00000110080	ST3GAL4	11	126225535	126310239	0.00822	20.037	2.16E-09	0.00050663	0.01015	4.7117	0.0092075	rs4065321	1.97E-13	0	2.52E-12	0	Pancreas
ENSG00000141150	RASL10B	17	34058668	34070540	ENSG00000120885	CLU	8	27454434	27472548	0.011644	7.1114	2.16E-09	0.00050673	0.024678	3.3038	0.0017736	rs9915021	2.20E-17	0	NA	NA	Cells_Transformed_fibroblasts
ENSG00000177951	BET1L	11	167784	207428	ENSG00000124491	F13A1	6	6144318	6321246	0.011623	7.0984	2.26E-09	0.00052958	0.024563	2.0832	0.019209	rs11602954	7.25E-48	0	9.12E-206	0	Adipose_Visceral_Omentum
ENSG00000177951	BET1L	11	167784	207428	ENSG00000124491	F13A1	6	6144318	6321246	0.011623	7.0984	2.26E-09	0.00052958	0.024563	2.0832	0.019209	rs11602954	7.25E-48	0	9.12E-206	0	Artery_Aorta
ENSG00000177951	BET1L	11	167784	207428	ENSG00000124491	F13A1	6	6144318	6321246	0.011623	7.0984	2.26E-09	0.00052958	0.024563	2.0832	0.019209	rs11602954	7.25E-48	0	9.12E-206	0	Cells_Transformed_fibroblasts
ENSG00000177951	BET1L	11	167784	207428	ENSG00000124491	F13A1	6	6144318	6321246	0.011623	7.0984	2.26E-09	0.00052958	0.024563	2.0832	0.019209	rs11602954	7.25E-48	0	9.12E-206	0	Pituitary
ENSG00000177951	BET1L	11	167784	207428	ENSG00000124491	F13A1	6	6144318	6321246	0.011623	7.0984	2.26E-09	0.00052958	0.024563	2.0832	0.019209	rs11602954	7.25E-48	0	9.12E-206	0	Prostate
ENSG00000177951	BET1L	11	167784	207428	ENSG00000124491	F13A1	6	6144318	6321246	0.011623	7.0984	2.26E-09	0.00052958	0.024563	2.0832	0.019209	rs11602954	7.25E-48	0	9.12E-206	0	Small_Intestine_Terminal_Ileum
ENSG00000177951	BET1L	11	167784	207428	ENSG00000124491	F13A1	6	6144318	6321246	0.011623	7.0984	2.26E-09	0.00052958	0.024563	2.0832	0.019209	rs11602954	7.25E-48	0	9.12E-206	0	Whole_Blood
ENSG00000177951	BET1L	11	167784	207428	ENSG00000124491	F13A1	6	6144318	6321246	0.011623	7.0984	2.26E-09	0.00052958	0.024563	2.0832	0.019209	rs17655730	3.37E-44	0	2.96E-166	0	Prostate
ENSG00000177951	BET1L	11	167784	207428	ENSG00000124491	F13A1	6	6144318	6321246	0.011623	7.0984	2.26E-09	0.00052958	0.024563	2.0832	0.019209	rs505404	5.93E-51	0	3.73E-187	0	Small_Intestine_Terminal_Ileum
ENSG00000177951	BET1L	11	167784	207428	ENSG00000124491	F13A1	6	6144318	6321246	0.011623	7.0984	2.26E-09	0.00052958	0.024563	2.0832	0.019209	rs505404	5.93E-51	0	3.73E-187	0	Whole_Blood
ENSG00000177951	BET1L	11	167784	207428	ENSG00000124491	F13A1	6	6144318	6321246	0.011623	7.0984	2.26E-09	0.00052958	0.024563	2.0832	0.019209	rs3782123	5.12E-07	0.00442957	NA	NA	Heart_Atrial_Appendage
ENSG00000115232	ITGA4	2	182321934	182400914	ENSG00000096006	CRISP3	6	49695097	49712150	0.011127	7.7638	2.28E-09	0.00053141	0.019639	2.6156	0.011192	rs1375493	1.23E-27	0	3.2717e-310	0	Adrenal_Gland
ENSG00000115232	ITGA4	2	182321934	182400914	ENSG00000096006	CRISP3	6	49695097	49712150	0.011127	7.7638	2.28E-09	0.00053141	0.019639	2.6156	0.011192	rs1375493	1.23E-27	0	3.2717e-310	0	Artery_Tibial
ENSG00000115232	ITGA4	2	182321934	182400914	ENSG00000096006	CRISP3	6	49695097	49712150	0.011127	7.7638	2.28E-09	0.00053141	0.019639	2.6156	0.011192	rs1375493	1.23E-27	0	3.2717e-310	0	Whole_Blood
ENSG00000115232	ITGA4	2	182321934	182400914	ENSG00000096006	CRISP3	6	49695097	49712150	0.011127	7.7638	2.28E-09	0.00053141	0.019639	2.6156	0.011192	rs2124440	8.36E-27	0	3.2717e-310	0	Adrenal_Gland
ENSG00000115232	ITGA4	2	182321934	182400914	ENSG00000096006	CRISP3	6	49695097	49712150	0.011127	7.7638	2.28E-09	0.00053141	0.019639	2.6156	0.011192	rs2124440	8.36E-27	0	3.2717e-310	0	Whole_Blood
ENSG00000115232	ITGA4	2	182321934	182400914	ENSG00000096006	CRISP3	6	49695097	49712150	0.011127	7.7638	2.28E-09	0.00053141	0.019639	2.6156	0.011192	rs1449263	6.73E-26	0	3.2717e-310	0	Liver
ENSG00000115232	ITGA4	2	182321934	182400914	ENSG00000096006	CRISP3	6	49695097	49712150	0.011127	7.7638	2.28E-09	0.00053141	0.019639	2.6156	0.011192	rs1449263	6.73E-26	0	3.2717e-310	0	Whole_Blood
ENSG00000108733	PEX12	17	33901814	33905882	ENSG00000256269	HMBS	11	118955576	118964259	0.010072	9.833	2.29E-09	0.0005343	0.014248	2.6479	0.02187	rs9915021	1.14E-11	0	NA	NA	Brain_Caudate_basal_ganglia
ENSG00000108733	PEX12	17	33901814	33905882	ENSG00000256269	HMBS	11	118955576	118964259	0.010072	9.833	2.29E-09	0.0005343	0.014248	2.6479	0.02187	rs9915021	1.14E-11	0	NA	NA	Breast_Mammary_Tissue
ENSG00000108733	PEX12	17	33901814	33905882	ENSG00000256269	HMBS	11	118955576	118964259	0.010072	9.833	2.29E-09	0.0005343	0.014248	2.6479	0.02187	rs10512472	1.67E-17	0	1.06E-12	0	Brain_Caudate_basal_ganglia
ENSG00000132139	GAS2L2	17	34071530	34079897	ENSG00000090674	MCOLN1	19	7587512	7595387	0.011606	7.0882	2.35E-09	0.00054662	0.0012935	0.14781	0.99678	rs9915021	9.31E-12	0	NA	NA	Small_Intestine_Terminal_Ileum
ENSG00000154760	SLFN13	17	33762115	33775856	ENSG00000113140	SPARC	5	151040657	151066726	0.012991	5.7743	2.38E-09	0.00055407	0.015452	1.1888	0.28607	rs225245	1.80E-17	0	NA	NA	Liver
ENSG00000154760	SLFN13	17	33762115	33775856	ENSG00000113140	SPARC	5	151040657	151066726	0.012991	5.7743	2.38E-09	0.00055407	0.015452	1.1888	0.28607	rs8082605	8.71E-47	0	2.08E-19	0	Artery_Tibial
ENSG00000154760	SLFN13	17	33762115	33775856	ENSG00000113140	SPARC	5	151040657	151066726	0.012991	5.7743	2.38E-09	0.00055407	0.015452	1.1888	0.28607	rs8082605	8.71E-47	0	2.08E-19	0	Esophagus_Mucosa
ENSG00000154760	SLFN13	17	33762115	33775856	ENSG00000113140	SPARC	5	151040657	151066726	0.012991	5.7743	2.38E-09	0.00055407	0.015452	1.1888	0.28607	rs8082605	8.71E-47	0	2.08E-19	0	Nerve_Tibial
ENSG00000154760	SLFN13	17	33762115	33775856	ENSG00000113140	SPARC	5	151040657	151066726	0.012991	5.7743	2.38E-09	0.00055407	0.015452	1.1888	0.28607	rs8082605	8.71E-47	0	2.08E-19	0	Pituitary
ENSG00000154760	SLFN13	17	33762115	33775856	ENSG00000113140	SPARC	5	151040657	151066726	0.012991	5.7743	2.38E-09	0.00055407	0.015452	1.1888	0.28607	rs10512472	7.03E-109	0	6.34E-38	0	Uterus
ENSG00000154760	SLFN13	17	33762115	33775856	ENSG00000017483	SLC38A5	X	48316920	48328644	0.012989	5.7737	2.39E-09	0.000555	0.038829	3.0602	0.00030123	rs225245	4.68E-30	0	NA	NA	Liver
ENSG00000154760	SLFN13	17	33762115	33775856	ENSG00000017483	SLC38A5	X	48316920	48328644	0.012989	5.7737	2.39E-09	0.000555	0.038829	3.0602	0.00030123	rs8082605	2.72E-07	0.00263795	2.08E-19	0	Artery_Tibial
ENSG00000154760	SLFN13	17	33762115	33775856	ENSG00000017483	SLC38A5	X	48316920	48328644	0.012989	5.7737	2.39E-09	0.000555	0.038829	3.0602	0.00030123	rs8082605	2.72E-07	0.00263795	2.08E-19	0	Esophagus_Mucosa
ENSG00000154760	SLFN13	17	33762115	33775856	ENSG00000017483	SLC38A5	X	48316920	48328644	0.012989	5.7737	2.39E-09	0.000555	0.038829	3.0602	0.00030123	rs8082605	2.72E-07	0.00263795	2.08E-19	0	Nerve_Tibial
ENSG00000154760	SLFN13	17	33762115	33775856	ENSG00000017483	SLC38A5	X	48316920	48328644	0.012989	5.7737	2.39E-09	0.000555	0.038829	3.0602	0.00030123	rs8082605	2.72E-07	0.00263795	2.08E-19	0	Pituitary
ENSG00000154760	SLFN13	17	33762115	33775856	ENSG00000017483	SLC38A5	X	48316920	48328644	0.012989	5.7737	2.39E-09	0.000555	0.038829	3.0602	0.00030123	rs10512472	1.90E-13	0	6.34E-38	0	Uterus
ENSG00000108733	PEX12	17	33901814	33905882	ENSG00000104904	OAZ1	19	2271369	2273487	0.010049	9.8102	2.42E-09	0.00056028	0.0025101	0.46102	0.80539	rs9915021	1.09E-09	3.93E-05	NA	NA	Brain_Caudate_basal_ganglia
ENSG00000108733	PEX12	17	33901814	33905882	ENSG00000104904	OAZ1	19	2271369	2273487	0.010049	9.8102	2.42E-09	0.00056028	0.0025101	0.46102	0.80539	rs9915021	1.09E-09	3.93E-05	NA	NA	Breast_Mammary_Tissue
ENSG00000108733	PEX12	17	33901814	33905882	ENSG00000104904	OAZ1	19	2271369	2273487	0.010049	9.8102	2.42E-09	0.00056028	0.0025101	0.46102	0.80539	rs10512472	9.23E-18	0	1.06E-12	0	Brain_Caudate_basal_ganglia
ENSG00000132139	GAS2L2	17	34071530	34079897	ENSG00000166169	POLL	10	103338639	103346957	0.011581	7.0725	2.48E-09	0.00057461	0.015244	1.7666	0.079939	rs9915021	3.09E-30	0	NA	NA	Small_Intestine_Terminal_Ileum
ENSG00000172660	TAF15	17	34136459	34174246	ENSG00000184792	OSBP2	22	31089769	31303811	0.010568	8.6003	2.52E-09	0.00058101	0.025887	4.0527	0.00051111	rs9915021	5.31E-07	0.00466469	NA	NA	Skin_Not_Sun_Exposed_Suprapubic
ENSG00000172123	SLFN12	17	33738079	33760302	ENSG00000164068	RNF123	3	49728563	49753910	0.014677	4.7885	2.52E-09	0.00058101	0.021745	3.3898	0.0025936	rs225245	4.45E-47	0	NA	NA	Cells_EBV-transformed_lymphocytes
ENSG00000172123	SLFN12	17	33738079	33760302	ENSG00000164068	RNF123	3	49728563	49753910	0.014677	4.7885	2.52E-09	0.00058101	0.021745	3.3898	0.0025936	rs9915021	2.27E-20	0	NA	NA	Breast_Mammary_Tissue
ENSG00000167914	GSDMA	17	38119226	38134019	ENSG00000135842	FAM129A	1	184759858	184943682	0.0081521	19.87	2.55E-09	0.00058637	0.0084699	3.9252	0.020071	rs3859192	2.10E-34	0	6.22E-13	0	Adipose_Subcutaneous
ENSG00000167914	GSDMA	17	38119226	38134019	ENSG00000135842	FAM129A	1	184759858	184943682	0.0081521	19.87	2.55E-09	0.00058637	0.0084699	3.9252	0.020071	rs3859192	2.10E-34	0	6.22E-13	0	Adipose_Visceral_Omentum
ENSG00000167914	GSDMA	17	38119226	38134019	ENSG00000135842	FAM129A	1	184759858	184943682	0.0081521	19.87	2.55E-09	0.00058637	0.0084699	3.9252	0.020071	rs3859192	2.10E-34	0	6.22E-13	0	Adrenal_Gland
ENSG00000167914	GSDMA	17	38119226	38134019	ENSG00000135842	FAM129A	1	184759858	184943682	0.0081521	19.87	2.55E-09	0.00058637	0.0084699	3.9252	0.020071	rs3859192	2.10E-34	0	6.22E-13	0	Artery_Aorta
ENSG00000167914	GSDMA	17	38119226	38134019	ENSG00000135842	FAM129A	1	184759858	184943682	0.0081521	19.87	2.55E-09	0.00058637	0.0084699	3.9252	0.020071	rs3859192	2.10E-34	0	6.22E-13	0	Artery_Coronary
ENSG00000167914	GSDMA	17	38119226	38134019	ENSG00000135842	FAM129A	1	184759858	184943682	0.0081521	19.87	2.55E-09	0.00058637	0.0084699	3.9252	0.020071	rs3859192	2.10E-34	0	6.22E-13	0	Artery_Tibial
ENSG00000167914	GSDMA	17	38119226	38134019	ENSG00000135842	FAM129A	1	184759858	184943682	0.0081521	19.87	2.55E-09	0.00058637	0.0084699	3.9252	0.020071	rs3859192	2.10E-34	0	6.22E-13	0	Breast_Mammary_Tissue
ENSG00000167914	GSDMA	17	38119226	38134019	ENSG00000135842	FAM129A	1	184759858	184943682	0.0081521	19.87	2.55E-09	0.00058637	0.0084699	3.9252	0.020071	rs3859192	2.10E-34	0	6.22E-13	0	Cells_Transformed_fibroblasts
ENSG00000167914	GSDMA	17	38119226	38134019	ENSG00000135842	FAM129A	1	184759858	184943682	0.0081521	19.87	2.55E-09	0.00058637	0.0084699	3.9252	0.020071	rs3859192	2.10E-34	0	6.22E-13	0	Colon_Sigmoid
ENSG00000167914	GSDMA	17	38119226	38134019	ENSG00000135842	FAM129A	1	184759858	184943682	0.0081521	19.87	2.55E-09	0.00058637	0.0084699	3.9252	0.020071	rs3859192	2.10E-34	0	6.22E-13	0	Colon_Transverse
ENSG00000167914	GSDMA	17	38119226	38134019	ENSG00000135842	FAM129A	1	184759858	184943682	0.0081521	19.87	2.55E-09	0.00058637	0.0084699	3.9252	0.020071	rs3859192	2.10E-34	0	6.22E-13	0	Esophagus_Gastroesophageal_Junction
ENSG00000167914	GSDMA	17	38119226	38134019	ENSG00000135842	FAM129A	1	184759858	184943682	0.0081521	19.87	2.55E-09	0.00058637	0.0084699	3.9252	0.020071	rs3859192	2.10E-34	0	6.22E-13	0	Esophagus_Muscularis
ENSG00000167914	GSDMA	17	38119226	38134019	ENSG00000135842	FAM129A	1	184759858	184943682	0.0081521	19.87	2.55E-09	0.00058637	0.0084699	3.9252	0.020071	rs3859192	2.10E-34	0	6.22E-13	0	Heart_Atrial_Appendage
ENSG00000167914	GSDMA	17	38119226	38134019	ENSG00000135842	FAM129A	1	184759858	184943682	0.0081521	19.87	2.55E-09	0.00058637	0.0084699	3.9252	0.020071	rs3859192	2.10E-34	0	6.22E-13	0	Heart_Left_Ventricle

ENSG00000167914	GSDMA	17	38119226	38134019	ENSG00000135842	FAM129A	1	184759858	184943682	0.0081521	19.87	2.55E-09	0.00058637	0.0084699	3.9252	0.020071	rs3859192	2.10E-34	0	6.22E-13	0	Liver
ENSG00000167914	GSDMA	17	38119226	38134019	ENSG00000135842	FAM129A	1	184759858	184943682	0.0081521	19.87	2.55E-09	0.00058637	0.0084699	3.9252	0.020071	rs3859192	2.10E-34	0	6.22E-13	0	Lung
ENSG00000167914	GSDMA	17	38119226	38134019	ENSG00000135842	FAM129A	1	184759858	184943682	0.0081521	19.87	2.55E-09	0.00058637	0.0084699	3.9252	0.020071	rs3859192	2.10E-34	0	6.22E-13	0	Muscle_Skeletal
ENSG00000167914	GSDMA	17	38119226	38134019	ENSG00000135842	FAM129A	1	184759858	184943682	0.0081521	19.87	2.55E-09	0.00058637	0.0084699	3.9252	0.020071	rs3859192	2.10E-34	0	6.22E-13	0	Nerve_Tibial
ENSG00000167914	GSDMA	17	38119226	38134019	ENSG00000135842	FAM129A	1	184759858	184943682	0.0081521	19.87	2.55E-09	0.00058637	0.0084699	3.9252	0.020071	rs3859192	2.10E-34	0	6.22E-13	0	Ovary
ENSG00000167914	GSDMA	17	38119226	38134019	ENSG00000135842	FAM129A	1	184759858	184943682	0.0081521	19.87	2.55E-09	0.00058637	0.0084699	3.9252	0.020071	rs3859192	2.10E-34	0	6.22E-13	0	Pancreas
ENSG00000167914	GSDMA	17	38119226	38134019	ENSG00000135842	FAM129A	1	184759858	184943682	0.0081521	19.87	2.55E-09	0.00058637	0.0084699	3.9252	0.020071	rs3859192	2.10E-34	0	6.22E-13	0	Small_Intestine_Terminal_Ileum
ENSG00000167914	GSDMA	17	38119226	38134019	ENSG00000135842	FAM129A	1	184759858	184943682	0.0081521	19.87	2.55E-09	0.00058637	0.0084699	3.9252	0.020071	rs3859192	2.10E-34	0	6.22E-13	0	Spleen
ENSG00000167914	GSDMA	17	38119226	38134019	ENSG00000135842	FAM129A	1	184759858	184943682	0.0081521	19.87	2.55E-09	0.00058637	0.0084699	3.9252	0.020071	rs3859192	2.10E-34	0	6.22E-13	0	Stomach
ENSG00000167914	GSDMA	17	38119226	38134019	ENSG00000135842	FAM129A	1	184759858	184943682	0.0081521	19.87	2.55E-09	0.00058637	0.0084699	3.9252	0.020071	rs3859192	2.10E-34	0	6.22E-13	0	Thyroid
ENSG00000167914	GSDMA	17	38119226	38134019	ENSG00000135842	FAM129A	1	184759858	184943682	0.0081521	19.87	2.55E-09	0.00058637	0.0084699	3.9252	0.020071	rs3859192	2.10E-34	0	6.22E-13	0	Whole_Blood
ENSG00000167914	GSDMA	17	38119226	38134019	ENSG00000135842	FAM129A	1	184759858	184943682	0.0081521	19.87	2.55E-09	0.00058637	0.0084699	3.9252	0.020071	rs17609240	1.68E-17	0	1.53E-144	0	Adipose_Subcutaneous
ENSG00000167914	GSDMA	17	38119226	38134019	ENSG00000135842	FAM129A	1	184759858	184943682	0.0081521	19.87	2.55E-09	0.00058637	0.0084699	3.9252	0.020071	rs17609240	1.68E-17	0	1.53E-144	0	Adipose_Visceral_Omentum
ENSG00000167914	GSDMA	17	38119226	38134019	ENSG00000135842	FAM129A	1	184759858	184943682	0.0081521	19.87	2.55E-09	0.00058637	0.0084699	3.9252	0.020071	rs17609240	1.68E-17	0	1.53E-144	0	Artery_Aorta
ENSG00000167914	GSDMA	17	38119226	38134019	ENSG00000135842	FAM129A	1	184759858	184943682	0.0081521	19.87	2.55E-09	0.00058637	0.0084699	3.9252	0.020071	rs17609240	1.68E-17	0	1.53E-144	0	Breast_Mammary_Tissue
ENSG00000167914	GSDMA	17	38119226	38134019	ENSG00000135842	FAM129A	1	184759858	184943682	0.0081521	19.87	2.55E-09	0.00058637	0.0084699	3.9252	0.020071	rs17609240	1.68E-17	0	1.53E-144	0	Colon_Sigmoid
ENSG00000167914	GSDMA	17	38119226	38134019	ENSG00000135842	FAM129A	1	184759858	184943682	0.0081521	19.87	2.55E-09	0.00058637	0.0084699	3.9252	0.020071	rs17609240	1.68E-17	0	1.53E-144	0	Esophagus_Gastroesophageal_Junction
ENSG00000167914	GSDMA	17	38119226	38134019	ENSG00000135842	FAM129A	1	184759858	184943682	0.0081521	19.87	2.55E-09	0.00058637	0.0084699	3.9252	0.020071	rs17609240	1.68E-17	0	1.53E-144	0	Esophagus_Muscularis
ENSG00000167914	GSDMA	17	38119226	38134019	ENSG00000135842	FAM129A	1	184759858	184943682	0.0081521	19.87	2.55E-09	0.00058637	0.0084699	3.9252	0.020071	rs17609240	1.68E-17	0	1.53E-144	0	Heart_Left_Ventricle
ENSG00000167914	GSDMA	17	38119226	38134019	ENSG00000135842	FAM129A	1	184759858	184943682	0.0081521	19.87	2.55E-09	0.00058637	0.0084699	3.9252	0.020071	rs17609240	1.68E-17	0	1.53E-144	0	Muscle_Skeletal
ENSG00000167914	GSDMA	17	38119226	38134019	ENSG00000135842	FAM129A	1	184759858	184943682	0.0081521	19.87	2.55E-09	0.00058637	0.0084699	3.9252	0.020071	rs3894194	3.63E-19	0	6.08E-29	0	Adipose_Subcutaneous
ENSG00000167914	GSDMA	17	38119226	38134019	ENSG00000135842	FAM129A	1	184759858	184943682	0.0081521	19.87	2.55E-09	0.00058637	0.0084699	3.9252	0.020071	rs3894194	3.63E-19	0	6.08E-29	0	Adipose_Visceral_Omentum
ENSG00000167914	GSDMA	17	38119226	38134019	ENSG00000135842	FAM129A	1	184759858	184943682	0.0081521	19.87	2.55E-09	0.00058637	0.0084699	3.9252	0.020071	rs3894194	3.63E-19	0	6.08E-29	0	Artery_Tibial
ENSG00000167914	GSDMA	17	38119226	38134019	ENSG00000135842	FAM129A	1	184759858	184943682	0.0081521	19.87	2.55E-09	0.00058637	0.0084699	3.9252	0.020071	rs3894194	3.63E-19	0	6.08E-29	0	Colon_Transverse
ENSG00000167914	GSDMA	17	38119226	38134019	ENSG00000135842	FAM129A	1	184759858	184943682	0.0081521	19.87	2.55E-09	0.00058637	0.0084699	3.9252	0.020071	rs3894194	3.63E-19	0	6.08E-29	0	Esophagus_Gastroesophageal_Junction
ENSG00000167914	GSDMA	17	38119226	38134019	ENSG00000135842	FAM129A	1	184759858	184943682	0.0081521	19.87	2.55E-09	0.00058637	0.0084699	3.9252	0.020071	rs3894194	3.63E-19	0	6.08E-29	0	Heart_Atrial_Appendage
ENSG00000167914	GSDMA	17	38119226	38134019	ENSG00000135842	FAM129A	1	184759858	184943682	0.0081521	19.87	2.55E-09	0.00058637	0.0084699	3.9252	0.020071	rs3894194	3.63E-19	0	6.08E-29	0	Muscle_Skeletal
ENSG00000167914	GSDMA	17	38119226	38134019	ENSG00000135842	FAM129A	1	184759858	184943682	0.0081521	19.87	2.55E-09	0.00058637	0.0084699	3.9252	0.020071	rs3894194	3.63E-19	0	6.08E-29	0	Whole_Blood
ENSG00000167914	GSDMA	17	38119226	38134019	ENSG00000135842	FAM129A	1	184759858	184943682	0.0081521	19.87	2.55E-09	0.00058637	0.0084699	3.9252	0.020071	rs7212938	1.48E-15	0	5.34E-44	0	Adipose_Subcutaneous
ENSG00000167914	GSDMA	17	38119226	38134019	ENSG00000135842	FAM129A	1	184759858	184943682	0.0081521	19.87	2.55E-09	0.00058637	0.0084699	3.9252	0.020071	rs7212938	1.48E-15	0	5.34E-44	0	Adipose_Visceral_Omentum
ENSG00000167914	GSDMA	17	38119226	38134019	ENSG00000135842	FAM129A	1	184759858	184943682	0.0081521	19.87	2.55E-09	0.00058637	0.0084699	3.9252	0.020071	rs7212938	1.48E-15	0	5.34E-44	0	Artery_Coronary
ENSG00000167914	GSDMA	17	38119226	38134019	ENSG00000135842	FAM129A	1	184759858	184943682	0.0081521	19.87	2.55E-09	0.00058637	0.0084699	3.9252	0.020071	rs7212938	1.48E-15	0	5.34E-44	0	Colon_Sigmoid
ENSG00000167914	GSDMA	17	38119226	38134019	ENSG00000135842	FAM129A	1	184759858	184943682	0.0081521	19.87	2.55E-09	0.00058637	0.0084699	3.9252	0.020071	rs7212938	1.48E-15	0	5.34E-44	0	Heart_Atrial_Appendage
ENSG00000167914	GSDMA	17	38119226	38134019	ENSG00000135842	FAM129A	1	184759858	184943682	0.0081521	19.87	2.55E-09	0.00058637	0.0084699	3.9252	0.020071	rs7212938	1.48E-15	0	5.34E-44	0	Heart_Left_Ventricle
ENSG00000167914	GSDMA	17	38119226	38134019	ENSG00000135842	FAM129A	1	184759858	184943682	0.0081521	19.87	2.55E-09	0.00058637	0.0084699	3.9252	0.020071	rs7212938	1.48E-15	0	5.34E-44	0	Thyroid
ENSG00000167914	GSDMA	17	38119226	38134019	ENSG00000135842	FAM129A	1	184759858	184943682	0.0081521	19.87	2.55E-09	0.00058637	0.0084699	3.9252	0.020071	rs7212938	1.48E-15	0	5.34E-44	0	Whole_Blood
ENSG00000167914	GSDMA	17	38119226	38134019	ENSG00000135842	FAM129A	1	184759858	184943682	0.0081521	19.87	2.55E-09	0.00058637	0.0084699	3.9252	0.020071	rs8078723	1.78E-38	0	6.97E-07	0.002194627	Breast_Mammary_Tissue
ENSG00000167914	GSDMA	17	38119226	38134019	ENSG00000135842	FAM129A	1	184759858	184943682	0.0081521	19.87	2.55E-09	0.00058637	0.0084699	3.9252	0.020071	rs8078723	1.78E-38	0	6.97E-07	0.002194627	Esophagus_Gastroesophageal_Junction
ENSG00000167914	GSDMA	17	38119226	38134019	ENSG00000135842	FAM129A	1	184759858	184943682	0.0081521	19.87	2.55E-09	0.00058637	0.0084699	3.9252	0.020071	rs4794820	1.01E-16	0	2.26E-66	0	Artery_Tibial
ENSG00000167914	GSDMA	17	38119226	38134019	ENSG00000135842	FAM129A	1	184759858	184943682	0.0081521	19.87	2.55E-09	0.00058637	0.0084699	3.9252	0.020071	rs4794820	1.01E-16	0	2.26E-66	0	Cells_Transformed_fibroblasts
ENSG00000167914	GSDMA	17	38119226	38134019	ENSG00000135842	FAM129A	1	184759858	184943682	0.0081521	19.87	2.55E-09	0.00058637	0.0084699	3.9252	0.020071	rs4794822	2.20E-38	0	3.02E-06	0.008756123	Breast_Mammary_Tissue
ENSG00000167914	GSDMA	17	38119226	38134019	ENSG00000135842	FAM129A	1	184759858	184943682	0.0081521	19.87	2.55E-09	0.00058637	0.0084699	3.9252	0.020071	rs4794822	2.20E-38	0	3.02E-06	0.008756123	Cells_Transformed_fibroblasts
ENSG00000167914	GSDMA	17	38119226	38134019	ENSG00000135842	FAM129A	1	184759858	184943682	0.0081521	19.87	2.55E-09	0.00058637	0.0084699	3.9252	0.020071	rs4794822	2.20E-38	0	3.02E-06	0.008756123	Esophagus_Gastroesophageal_Junction
ENSG00000167914	GSDMA	17	38119226	38134019	ENSG00000135842	FAM129A	1	184759858	184943682	0.0081521	19.87	2.55E-09	0.00058637	0.0084699	3.9252	0.020071	rs4794822	2.20E-38	0	3.02E-06	0.008756123	Esophagus_Muscularis
ENSG00000167914	GSDMA	17	38119226	38134019	ENSG00000135842	FAM129A	1	184759858	184943682	0.0081521	19.87	2.55E-09	0.00058637	0.0084699	3.9252	0.020071	rs4065321	7.18E-33	0	2.52E-12	0	Pancreas
ENSG00000006125	AP2B1	17	33913918	34053436	ENSG00000197993	KEL	7	142638201	142659768	0.01106	7.7171	2.64E-09	0.00060622	0.020397	3.1753	0.0043402	rs10512472	7.82E-19	0	3.62E-14	0	Heart_Left_Ventricle
ENSG00000108733	PEX12	17	33901814	33905882	ENSG00000118816	CCNI	4	77968311	77997158	0.0099991	9.7607	2.71E-09	0.00062245	0.0027042	0.49675	0.77884	rs9915021	4.53E-07	0.00406513	NA	NA	Brain_Caudate_basal_ganglia
ENSG00000108733	PEX12	17	33901814	33905882	ENSG00000118816	CCNI	4	77968311	77997158	0.0099991	9.7607	2.71E-09	0.00062245	0.0027042	0.49675	0.77884	rs9915021	4.53E-07	0.00406513	NA	NA	Breast_Mammary_Tissue
ENSG00000108733	PEX12	17	33901814	33905882	ENSG00000118816	CCNI	4	77968311	77997158	0.0099991	9.7607	2.71E-09	0.00062245	0.0027042	0.49675	0.77884	rs10512472	1.80E-07	0.00175269	1.06E-12	0	Brain_Caudate_basal_ganglia
ENSG00000157500	APPL1	3	57261765	57301820	ENSG00000205038	PKHD1L1	8	110374706	110542559	0.0094154	11.484	2.80E-09	0.00064121	0.00085651	0.26232	0.85257	rs12485738	1.94E-59	0	NA	NA	Brain_Anterior_cingulate_cortex_BA24
ENSG00000154760	SLFN13	17	33762115	33775856	ENSG00000095303	PTGS1	9	125132824	125157982	0.01291	5.7379	2.83E-09	0.00064627	0.034444	2.7022	0.0013662	rs225245	4.34E-32	0	NA	NA	Liver
ENSG00000154760	SLFN13	17	33762115	33775856	ENSG00000095303	PTGS1	9	125132824	125157982	0.01291	5.7379	2.83E-09	0.00064627	0.034444	2.7022	0.0013662	rs8082605	6.10E-57	0	2.08E-19	0	Artery_Tibial
ENSG00000154760	SLFN13	17	33762115	33775856	ENSG00000095303	PTGS1	9	125132824	125157982	0.01291	5.7379	2.83E-09	0.00064627	0.034444	2.7022	0.0013662	rs8082605	6.10E-57	0	2.08E-19	0	Esophagus_Mucosa
ENSG00000154760	SLFN13	17	33762115	33775856	ENSG00000095303	PTGS1	9	125132824	125157982	0.01291	5.7379	2.83E-09	0.00064627	0.034444	2.7022	0.0013662	rs8082605	6.10E-57	0	2.08E-19	0	Nerve_Tibial
ENSG00000154760	SLFN13	17	33762115	33775856	ENSG00000095303	PTGS1	9	125132824	125157982	0.01291	5.7379	2.83E-09	0.00064627	0.034444	2.7022	0.0013662	rs8082605	6.10E-57	0	2.08E-19	0	Pituitary
ENSG00000154760	SLFN13	17	33762115	33775856	ENSG00000095303	PTGS1	9	125132824	125157982	0.01291	5.7379	2.83E-09	0.00064627	0.034444	2.7022	0.0013662	rs10512472	8.41E-119	0	6.34E-38	0	Uterus
ENSG00000108733	PEX12	17	33901814	33905882	ENSG00000133816	MICAL2	11	12115543	12285332	0.0099757	9.7376	2.86E-09	0.0006524	0.0045444	0.83633	0.52395	rs9915021	1.31E-21	0	NA	NA	Brain_Caudate_basal_ganglia
ENSG00000108733	PEX12	17	33901814	33905882	ENSG00000133816	MICAL2	11	12115543	12285332	0.0099757	9.7376	2.86E-09	0.0006524	0.0045444	0.83633	0.52395	rs9915021	1.31E-21	0	NA	NA	Breast_Mammary_Tissue
ENSG00000108733	PEX12	17	33901814	33905882	ENSG00000133816	MICAL2	11	12115543	12285332	0.0099757	9.7376	2.86E-09	0.0006524	0.0045444	0.83633	0.52395	rs10512472	2.85E-24	0	1.06E-12	0	Brain_Caudate_basal_ganglia
ENSG00000108733	PEX12	17	33901814	33905882	ENSG00000133808	MICALCL	11	12297627	12380691	0.0099757	9.7376	2.86E-09	0.0006524	0.0034141	0.6276	0.67875	rs9915021	8.15E-10	2.02E-05	NA	NA	Brain_Caudate_basal_ganglia
ENSG00000108733	PEX12	17	33901814	33905882	ENSG00000133808	MICALCL	11	12297627	12380691	0.0099757	9.7376	2.86E-09	0.0006524	0.0034141	0.6276	0.67875	rs9915021	8.15E-10	2.02E-05	NA	NA	Breast_Mammary_Tissue
ENSG00000108733	PEX12	17	33901814	33905882	ENSG00000133808	MICALCL	11	12297627	12380691	0.0099757	9.7376	2.86E-09	0.0006524	0.0034141	0.6276	0.67875	rs10512472	1.86E-09	5.64E-05	1.06E-12	0	Brain_Caudate_basal_ganglia
ENSG00000205045	SLFN12L	17	33800708	33864880	ENSG00000100614	PPM1A	14	60712470	60765805	0.011011	7.6823	2.94E-09	0.00067051	0.012845	1.699	0.10566	rs8082605	1.56E-11	0	2.49E-31	0	Adipose_Subcutaneous
ENSG00000205045	SLFN12L	17	33800708	33864880	ENSG00000100614	PPM1A	14	60712470	60765805	0.011011	7.6823	2.94E-09	0.00067051	0.012845	1.699	0.10566	rs8082605	1.56E-11	0	2.49E-31	0	Adipose_Visceral_Omentum
ENSG00000205045	SLFN12L	17	33800708	33864880	ENSG00000100614	PPM1A	14	60712470	60765805	0.011011	7.6823	2.94E-09	0.00067051	0.012845	1.699	0.10566	rs8082605	1.56E-11	0	2.49E-31	0	Heart_Left_Ventricle
ENSG00000205045	SLFN12L	17	33800708	33864880	ENSG00000100614	PPM1A	14	60712470	60765805	0.011011	7.6823	2.94E-09	0.00067051	0.012845	1.699	0.10566	rs8082605	1.56E-11	0	2.49E-31	0	Nerve_Tibial
ENSG00000205045	SLFN12L	17	33800708	33864880	ENSG00000100614	PPM1A	14	60712470	60765805	0.011011	7.6823	2.94E-09	0.00067051	0.012845	1.699	0.10566	rs10512472	4.22E-11	0	4.96E-30	0	Pancreas
ENSG00000141748	ARL5C	17	37313147	37323318	ENSG00000162551	ALPL	1	21835858	21904905	0.0080871	19.71	2.98E-09	0.00067875	0.001053	0.48436	0.61625	rs12946510	8.70E-07	0.00729639	NA	NA	Adipose_Subcutaneous
ENSG00000141748	ARL5C	17	37313147	37323318	ENSG00000162551	ALPL	1	21835858	21904905	0.0080871	19.71	2.98E-09	0.00067875	0.001053	0.48436	0.61625	rs10445308	1.27E-07	0.0013747	NA	NA	Adipose_Subcutaneous
ENSG00000172123	SLFN12	17	33738079	33760302	ENSG00000029534	ANK1	8	41510739	41754280	0.014587	4.7585	3.02E-09	0.0006863	0.0054583	0.83696	0.54138	rs225245	8.61E-22	0	NA	NA	Cells_EBV-transformed_lymphocytes
ENSG00000172123	SLFN12	17	33738079	33760302	ENSG00000029534	ANK1	8	41510739	41754280	0.014587	4.7585	3.02E-09	0.0006863	0.0054583	0.83696	0.54138	rs9915021	6.17E-12	0	NA	NA	Breast_Mammary_Tissue
ENSG00000163946	FAM208A	3	56658507	56717265	ENSG00000158457	TSPAN33	7	128784712	128808637	0.011485	7.013	3.06E-09	0.00069389	0.0086431	1.1384	0.33651	rs12485738	4.27E-189	0	NA	NA	Esophagus_Mucosa
ENSG00000163946	FAM208A	3	56658507	56717265	ENSG00000158457	TSPAN33	7	128784712	128808637	0.011485	7.013	3.06E-09	0.00069389	0.0086431	1.1384	0.33651	rs1354034	3.27170000000001e-	0	NA	NA	Heart_Atrial_Appendage
ENSG00000123411	IKZF4	12	56401443	56432219	ENSG00000105085	MED26	19	16698215	16739873	0.010961	7.6473	3.29E-09	0.0007405	0.0066049	0.86814	0.5312	rs10876864	6.01E-06	0.03846264	NA	NA	Skin_Sun_Exposed_Lower_leg
ENSG00000172346	CSDC2	22	41956767	41972670	ENSG00000175556	LONRF3	X	118108581	118156888	0.0093315	11.381	3.41E-09	0.00076736	0.01797	3.3524	0.0052408	rs4822024	1.77E-35	0	1.85E-21	0	Brain_Frontal_Cortex_BA9
ENSG00000172346	CSDC2	22	41956767	41972670	ENSG00000175556	LONRF3	X	118108581	118156888	0.0093315	11.381	3.41E-09	0.00076736	0.01797	3.3524	0.0052408	rs4822024	1.77E-35	0	1.85E-21	0	Liver
ENSG00000141150	RASL10B	17	34058668	34070540	ENSG00000164068	RNF123	3	49728563	49753910	0.011435	6.9822	3.42E-09	0.00076823	0.014223	1.8839	0.069025	rs9915021	2.27E-20	0	NA	NA	Cells_Transformed_fibroblasts
ENSG00000141150	RASL10B	17	34058668	34070540	ENSG00000140564	FURIN	15	91411822	91426688	0.011434	6.9814	3.43E-09	0.00076954	0.0029409	0.38513	0.91136	rs9915021	2.81E-16	0	NA	NA	Cells_Transformed_fibroblasts
ENSG00000154768	C17orf50	17	34087916	34092098	ENSG00000166947	EPB42	15	43398423	43513481	0.010428	8.4848	3.46E-09	0.00077587	0.0078262	1.0299	0.40826	rs9915021	3.48E-18	0	NA	NA	Pituitary
ENSG00000172660	TAF15	17	34136459	34174246	ENSG00000130300	PLVAP	19	17462257	17488159	0.010418	8.4764	3.54E-09	0.00079312	0.020136	3.1338	0.0047918	rs9915021	2.45E-12	0	NA	NA	Skin_Not_Sun_Exposed_Suprapubic
ENSG00000184886	PIGW	17	34892943	34895159	ENSG00000167992	VWCE	11	61025762	61062896	0.0093134	11.359	3.56E-09	0.00079608	0.016359	3.0467	0.0098086	rs9915021	1.45E-41	0	NA	NA	Muscle_Skeletal
ENSG00000249242	TMEM150C	4	83405743	83483510	ENSG00000164821	DEFA4	8	6793344	6795860	0.012793	5.6854	3.62E-09	0.00080709	0.0037425	0.31077	0.98376	rs17005891	3.52E-07	0.00324805	NA	NA	Heart_Left_Ventricle

ENSG00000249242	TMEM150C	4	83405743	83483510	ENSG00000164821	DEFA4	8	6793344	6795860	0.012793	5.6854	3.62E-09	0.00080709	0.0037425	0.31077	0.98376	rs17005891	3.52E-07	0.00324805	NA	NA	Muscle_Skeletal
ENSG00000249242	TMEM150C	4	83405743	83483510	ENSG00000164821	DEFA4	8	6793344	6795860	0.012793	5.6854	3.62E-09	0.00080709	0.0037425	0.31077	0.98376	rs17005891	3.52E-07	0.00324805	NA	NA	Thyroid
ENSG00000008838	MED24	17	38175350	38217468	ENSG00000146592	CREB5	7	28338940	28865511	0.0093022	11.345	3.65E-09	0.00081281	0.0047248	1.0883	0.361	rs12946510	5.08E-06	0.03324727	NA	NA	Prostate
ENSG00000008838	MED24	17	38175350	38217468	ENSG00000146592	CREB5	7	28338940	28865511	0.0093022	11.345	3.65E-09	0.00081281	0.0047248	1.0883	0.361	rs3859192	1.81E-15	0	1.47E-40	0	Whole_Blood
ENSG00000008838	MED24	17	38175350	38217468	ENSG00000146592	CREB5	7	28338940	28865511	0.0093022	11.345	3.65E-09	0.00081281	0.0047248	1.0883	0.361	rs3894194	4.69E-11	0	8.18E-15	0	Thyroid
ENSG00000008838	MED24	17	38175350	38217468	ENSG00000146592	CREB5	7	28338940	28865511	0.0093022	11.345	3.65E-09	0.00081281	0.0047248	1.0883	0.361	rs7212938	2.44E-08	0.00033038	4.22E-10	0	Testis
ENSG00000008838	MED24	17	38175350	38217468	ENSG00000146592	CREB5	7	28338940	28865511	0.0093022	11.345	3.65E-09	0.00081281	0.0047248	1.0883	0.361	rs7212938	2.44E-08	0.00033038	4.22E-10	0	Thyroid
ENSG00000008838	MED24	17	38175350	38217468	ENSG00000146592	CREB5	7	28338940	28865511	0.0093022	11.345	3.65E-09	0.00081281	0.0047248	1.0883	0.361	rs8078723	1.90E-17	0	5.30E-78	0	Adipose_Subcutaneous
ENSG00000008838	MED24	17	38175350	38217468	ENSG00000146592	CREB5	7	28338940	28865511	0.0093022	11.345	3.65E-09	0.00081281	0.0047248	1.0883	0.361	rs8078723	1.90E-17	0	5.30E-78	0	Artery_Tibial
ENSG00000008838	MED24	17	38175350	38217468	ENSG00000146592	CREB5	7	28338940	28865511	0.0093022	11.345	3.65E-09	0.00081281	0.0047248	1.0883	0.361	rs8078723	1.90E-17	0	5.30E-78	0	Cells_Transformed_fibroblasts
ENSG00000008838	MED24	17	38175350	38217468	ENSG00000146592	CREB5	7	28338940	28865511	0.0093022	11.345	3.65E-09	0.00081281	0.0047248	1.0883	0.361	rs8078723	1.90E-17	0	5.30E-78	0	Lung
ENSG00000008838	MED24	17	38175350	38217468	ENSG00000146592	CREB5	7	28338940	28865511	0.0093022	11.345	3.65E-09	0.00081281	0.0047248	1.0883	0.361	rs8078723	1.90E-17	0	5.30E-78	0	Pituitary
ENSG00000008838	MED24	17	38175350	38217468	ENSG00000146592	CREB5	7	28338940	28865511	0.0093022	11.345	3.65E-09	0.00081281	0.0047248	1.0883	0.361	rs8078723	1.90E-17	0	5.30E-78	0	Skin_Sun_Exposed_Lower_leg
ENSG00000008838	MED24	17	38175350	38217468	ENSG00000146592	CREB5	7	28338940	28865511	0.0093022	11.345	3.65E-09	0.00081281	0.0047248	1.0883	0.361	rs8078723	1.90E-17	0	5.30E-78	0	Spleen
ENSG00000008838	MED24	17	38175350	38217468	ENSG00000146592	CREB5	7	28338940	28865511	0.0093022	11.345	3.65E-09	0.00081281	0.0047248	1.0883	0.361	rs8078723	1.90E-17	0	5.30E-78	0	Stomach
ENSG00000008838	MED24	17	38175350	38217468	ENSG00000146592	CREB5	7	28338940	28865511	0.0093022	11.345	3.65E-09	0.00081281	0.0047248	1.0883	0.361	rs8078723	1.90E-17	0	5.30E-78	0	Thyroid
ENSG00000008838	MED24	17	38175350	38217468	ENSG00000146592	CREB5	7	28338940	28865511	0.0093022	11.345	3.65E-09	0.00081281	0.0047248	1.0883	0.361	rs4794822	1.89E-17	0	1.32E-79	0	Esophagus_Gastroesophageal_Junction
ENSG00000008838	MED24	17	38175350	38217468	ENSG00000146592	CREB5	7	28338940	28865511	0.0093022	11.345	3.65E-09	0.00081281	0.0047248	1.0883	0.361	rs4794822	1.89E-17	0	1.32E-79	0	Lung
ENSG00000008838	MED24	17	38175350	38217468	ENSG00000146592	CREB5	7	28338940	28865511	0.0093022	11.345	3.65E-09	0.00081281	0.0047248	1.0883	0.361	rs4794822	1.89E-17	0	1.32E-79	0	Spleen
ENSG00000008838	MED24	17	38175350	38217468	ENSG00000146592	CREB5	7	28338940	28865511	0.0093022	11.345	3.65E-09	0.00081281	0.0047248	1.0883	0.361	rs4794822	1.89E-17	0	1.32E-79	0	Whole_Blood
ENSG00000008838	MED24	17	38175350	38217468	ENSG00000146592	CREB5	7	28338940	28865511	0.0093022	11.345	3.65E-09	0.00081281	0.0047248	1.0883	0.361	rs4065321	5.35E-14	0	1.24E-42	0	Pituitary
ENSG00000099849	RASSF7	11	560404	564021	ENSG00000172572	PDE3A	12	20522179	20837315	0.010915	7.6142	3.65E-09	0.00081281	NA	NA	NA	rs505404	3.08E-06	0.02212182	NA	NA	Brain_Frontal_Cortex_BA9
ENSG00000198917	C9orf114	9	131586026	131592100	ENSG00000101425	BPI	20	36888551	36965907	0.0092831	11.321	3.82E-09	0.00084527	0.0093119	2.8762	0.03522	rs15676	1.15E-32	0	NA	NA	Breast_Mammary_Tissue
ENSG00000177951	BET1L	11	167784	207428	ENSG00000061918	GUCY1B3	4	156680144	156728743	0.01138	6.9483	3.85E-09	0.00085221	0.021613	1.8275	0.045599	rs11602954	8.40E-28	0	9.12E-206	0	Adipose_Visceral_Omentum
ENSG00000177951	BET1L	11	167784	207428	ENSG00000061918	GUCY1B3	4	156680144	156728743	0.01138	6.9483	3.85E-09	0.00085221	0.021613	1.8275	0.045599	rs11602954	8.40E-28	0	9.12E-206	0	Artery_Aorta
ENSG00000177951	BET1L	11	167784	207428	ENSG00000061918	GUCY1B3	4	156680144	156728743	0.01138	6.9483	3.85E-09	0.00085221	0.021613	1.8275	0.045599	rs11602954	8.40E-28	0	9.12E-206	0	Cells_Transformed_fibroblasts
ENSG00000177951	BET1L	11	167784	207428	ENSG00000061918	GUCY1B3	4	156680144	156728743	0.01138	6.9483	3.85E-09	0.00085221	0.021613	1.8275	0.045599	rs11602954	8.40E-28	0	9.12E-206	0	Pituitary
ENSG00000177951	BET1L	11	167784	207428	ENSG00000061918	GUCY1B3	4	156680144	156728743	0.01138	6.9483	3.85E-09	0.00085221	0.021613	1.8275	0.045599	rs11602954	8.40E-28	0	9.12E-206	0	Prostate
ENSG00000177951	BET1L	11	167784	207428	ENSG00000061918	GUCY1B3	4	156680144	156728743	0.01138	6.9483	3.85E-09	0.00085221	0.021613	1.8275	0.045599	rs11602954	8.40E-28	0	9.12E-206	0	Small_Intestine_Terminal_Ileum
ENSG00000177951	BET1L	11	167784	207428	ENSG00000061918	GUCY1B3	4	156680144	156728743	0.01138	6.9483	3.85E-09	0.00085221	0.021613	1.8275	0.045599	rs11602954	8.40E-28	0	9.12E-206	0	Whole_Blood
ENSG00000177951	BET1L	11	167784	207428	ENSG00000061918	GUCY1B3	4	156680144	156728743	0.01138	6.9483	3.85E-09	0.00085221	0.021613	1.8275	0.045599	rs17655730	5.01E-29	0	2.96E-166	0	Prostate
ENSG00000177951	BET1L	11	167784	207428	ENSG00000061918	GUCY1B3	4	156680144	156728743	0.01138	6.9483	3.85E-09	0.00085221	0.021613	1.8275	0.045599	rs505404	1.76E-34	0	3.73E-187	0	Small_Intestine_Terminal_Ileum
ENSG00000177951	BET1L	11	167784	207428	ENSG00000061918	GUCY1B3	4	156680144	156728743	0.01138	6.9483	3.85E-09	0.00085221	0.021613	1.8275	0.045599	rs505404	1.76E-34	0	3.73E-187	0	Whole_Blood
ENSG00000092871	RFFL	17	33341759	33416338	ENSG00000164068	RNF123	3	49728563	49753910	0.010372	8.4391	3.92E-09	0.00086358	0.010437	1.2036	0.29346	rs9915021	2.27E-20	0	NA	NA	Adipose_Subcutaneous
ENSG00000092871	RFFL	17	33341759	33416338	ENSG00000164068	RNF123	3	49728563	49753910	0.010372	8.4391	3.92E-09	0.00086358	0.010437	1.2036	0.29346	rs9915021	2.27E-20	0	NA	NA	Artery_Tibial
ENSG00000172660	TAF15	17	34136459	34174246	ENSG00000118816	CCNI	4	77968311	77997158	0.010351	8.4213	4.12E-09	0.00090287	0.016579	2.571	0.017836	rs9915021	4.53E-07	0.00406513	NA	NA	Skin_Not_Sun_Exposed_Suprapubic
ENSG00000092871	RFFL	17	33341759	33416338	ENSG00000167992	VWCE	11	61025762	61062896	0.010346	8.4173	4.16E-09	0.00091185	0.016253	1.8855	0.058957	rs9915021	1.45E-41	0	NA	NA	Adipose_Subcutaneous
ENSG00000092871	RFFL	17	33341759	33416338	ENSG00000167992	VWCE	11	61025762	61062896	0.010346	8.4173	4.16E-09	0.00091185	0.016253	1.8855	0.058957	rs9915021	1.45E-41	0	NA	NA	Artery_Tibial
ENSG00000108733	PEX12	17	33901814	33905882	ENSG00000153162	BMP6	6	7727030	7880334	0.0098051	9.5695	4.23E-09	0.00092474	0.0045688	0.84085	0.52079	rs9915021	4.57E-11	0	NA	NA	Brain_Caudate_basal_ganglia
ENSG00000108733	PEX12	17	33901814	33905882	ENSG00000153162	BMP6	6	7727030	7880334	0.0098051	9.5695	4.23E-09	0.00092474	0.0045688	0.84085	0.52079	rs9915021	4.57E-11	0	NA	NA	Breast_Mammary_Tissue
ENSG00000108733	PEX12	17	33901814	33905882	ENSG00000153162	BMP6	6	7727030	7880334	0.0098051	9.5695	4.23E-09	0.00092474	0.0045688	0.84085	0.52079	rs10512472	2.80E-30	0	1.06E-12	0	Brain_Caudate_basal_ganglia
ENSG00000132139	GAS2L2	17	34071530	34079897	ENSG00000095321	CRAT	9	131857089	131873083	0.011321	6.9118	4.39E-09	0.00095542	0.008369	0.96317	0.46349	rs9915021	1.11E-19	0	NA	NA	Small_Intestine_Terminal_Ileum
ENSG00000174885	NLRP6	11	278365	285359	ENSG00000172572	PDE3A	12	20522179	20837315	0.010825	7.5511	4.45E-09	0.00096741	NA	NA	NA	rs17655730	1.27E-06	0.0101111	5.43E-117	0	Artery_Aorta
ENSG00000174885	NLRP6	11	278365	285359	ENSG00000172572	PDE3A	12	20522179	20837315	0.010825	7.5511	4.45E-09	0.00096741	NA	NA	NA	rs17655730	1.27E-06	0.0101111	5.43E-117	0	Nerve_Tibial
ENSG00000174885	NLRP6	11	278365	285359	ENSG00000172572	PDE3A	12	20522179	20837315	0.010825	7.5511	4.45E-09	0.00096741	NA	NA	NA	rs17655730	1.27E-06	0.0101111	5.43E-117	0	Skin_Sun_Exposed_Lower_leg
ENSG00000174885	NLRP6	11	278365	285359	ENSG00000172572	PDE3A	12	20522179	20837315	0.010825	7.5511	4.45E-09	0.00096741	NA	NA	NA	rs505404	3.08E-06	0.02212182	2.65E-88	0	Nerve_Tibial
ENSG00000174885	NLRP6	11	278365	285359	ENSG00000172572	PDE3A	12	20522179	20837315	0.010825	7.5511	4.45E-09	0.00096741	NA	NA	NA	rs505404	3.08E-06	0.02212182	2.65E-88	0	Prostate
ENSG00000142082	SIRT3	11	215458	236431	ENSG00000120885	CLU	8	27454434	27472548	0.0086008	13.979	4.51E-09	0.00097732	0.010469	2.4254	0.046543	rs11602954	1.24E-10	0	1.39E-80	0	Adipose_Subcutaneous
ENSG00000142082	SIRT3	11	215458	236431	ENSG00000120885	CLU	8	27454434	27472548	0.0086008	13.979	4.51E-09	0.00097732	0.010469	2.4254	0.046543	rs11602954	1.24E-10	0	1.39E-80	0	Artery_Tibial
ENSG00000142082	SIRT3	11	215458	236431	ENSG00000120885	CLU	8	27454434	27472548	0.0086008	13.979	4.51E-09	0.00097732	0.010469	2.4254	0.046543	rs11602954	1.24E-10	0	1.39E-80	0	Breast_Mammary_Tissue
ENSG00000142082	SIRT3	11	215458	236431	ENSG00000120885	CLU	8	27454434	27472548	0.0086008	13.979	4.51E-09	0.00097732	0.010469	2.4254	0.046543	rs11602954	1.24E-10	0	1.39E-80	0	Cells_Transformed_fibroblasts
ENSG00000142082	SIRT3	11	215458	236431	ENSG00000120885	CLU	8	27454434	27472548	0.0086008	13.979	4.51E-09	0.00097732	0.010469	2.4254	0.046543	rs11602954	1.24E-10	0	1.39E-80	0	Nerve_Tibial
ENSG00000142082	SIRT3	11	215458	236431	ENSG00000120885	CLU	8	27454434	27472548	0.0086008	13.979	4.51E-09	0.00097732	0.010469	2.4254	0.046543	rs505404	7.47E-15	0	1.05E-94	0	Adrenal_Gland
ENSG00000142082	SIRT3	11	215458	236431	ENSG00000120885	CLU	8	27454434	27472548	0.0086008	13.979	4.51E-09	0.00097732	0.010469	2.4254	0.046543	rs505404	7.47E-15	0	1.05E-94	0	Artery_Aorta
ENSG00000142082	SIRT3	11	215458	236431	ENSG00000120885	CLU	8	27454434	27472548	0.0086008	13.979	4.51E-09	0.00097732	0.010469	2.4254	0.046543	rs505404	7.47E-15	0	1.05E-94	0	Lung
ENSG00000142082	SIRT3	11	215458	236431	ENSG00000120885	CLU	8	27454434	27472548	0.0086008	13.979	4.51E-09	0.00097732	0.010469	2.4254	0.046543	rs505404	7.47E-15	0	1.05E-94	0	Thyroid
ENSG00000142082	SIRT3	11	215458	236431	ENSG00000061918	GUCY1B3	4	156680144	156728743	0.0085977	13.974	4.54E-09	0.00098231	0.011294	2.6187	0.033822	rs11602954	8.40E-28	0	1.39E-80	0	Adipose_Subcutaneous
ENSG00000142082	SIRT3	11	215458	236431	ENSG00000061918	GUCY1B3	4	156680144	156728743	0.0085977	13.974	4.54E-09	0.00098231	0.011294	2.6187	0.033822	rs11602954	8.40E-28	0	1.39E-80	0	Artery_Tibial
ENSG00000142082	SIRT3	11	215458	236431	ENSG00000061918	GUCY1B3	4	156680144	156728743	0.0085977	13.974	4.54E-09	0.00098231	0.011294	2.6187	0.033822	rs11602954	8.40E-28	0	1.39E-80	0	Breast_Mammary_Tissue
ENSG00000142082	SIRT3	11	215458	236431	ENSG00000061918	GUCY1B3	4	156680144	156728743	0.0085977	13.974	4.54E-09	0.00098231	0.011294	2.6187	0.033822	rs11602954	8.40E-28	0	1.39E-80	0	Cells_Transformed_fibroblasts
ENSG00000142082	SIRT3	11	215458	236431	ENSG00000061918	GUCY1B3	4	156680144	156728743	0.0085977	13.974	4.54E-09	0.00098231	0.011294	2.6187	0.033822	rs11602954	8.40E-28	0	1.39E-80	0	Nerve_Tibial
ENSG00000142082	SIRT3	11	215458	236431	ENSG00000061918	GUCY1B3	4	156680144	156728743	0.0085977	13.974	4.54E-09	0.00098231	0.011294	2.6187	0.033822	rs505404	1.76E-34	0	1.05E-94	0	Adrenal_Gland
ENSG00000142082	SIRT3	11	215458	236431	ENSG00000061918	GUCY1B3	4	156680144	156728743	0.0085977	13.974	4.54E-09	0.00098231	0.011294	2.6187	0.033822	rs505404	1.76E-34	0	1.05E-94	0	Artery_Aorta
ENSG00000142082	SIRT3	11	215458	236431	ENSG00000061918	GUCY1B3	4	156680144	156728743	0.0085977	13.974	4.54E-09	0.00098231	0.011294	2.6187	0.033822	rs505404	1.76E-34	0	1.05E-94	0	Lung
ENSG00000142082	SIRT3	11	215458	236431	ENSG00000061918	GUCY1B3	4	156680144	156728743	0.0085977	13.974	4.54E-09	0.00098231	0.011294	2.6187	0.033822	rs505404	1.76E-34	0	1.05E-94	0	Thyroid
ENSG00000115486	GGCX	2	85774743	85788670	ENSG00000141096	DPEP3	16	68009566	68014732	0.0092026	11.222	4.61E-09	0.00099441	0.031163	7.3738	7.59E-06	rs10187424	6.33E-97	0	3.2717e-310	0	Artery_Aorta
ENSG00000115486	GGCX	2	85774743	85788670	ENSG00000141096	DPEP3	16	68009566	68014732	0.0092026	11.222	4.61E-09	0.00099441	0.031163	7.3738	7.59E-06	rs10187424	6.33E-97	0	3.2717e-310	0	Artery_Tibial
ENSG00000115486	GGCX	2	85774743	85788670	ENSG00000141096	DPEP3	16	68009566	68014732	0.0092026	11.222	4.61E-09	0.00099441	0.031163	7.3738	7.59E-06	rs10187424	6.33E-97	0	3.2717e-310	0	Brain_Cortex
ENSG00000115486	GGCX	2	85774743	85788670	ENSG00000141096	DPEP3	16	68009566	68014732	0.0092026	11.222	4.61E-09	0.00099441	0.031163	7.3738	7.59E-06	rs10187424	6.33E-97	0	3.2717e-310	0	Colon_Transverse
ENSG00000115486	GGCX	2	85774743	85788670	ENSG00000141096	DPEP3	16	68009566	68014732	0.0092026	11.222	4.61E-09	0.00099441	0.031163	7.3738	7.59E-06	rs10187424	6.33E-97	0	3.2717e-310	0	Nerve_Tibial
ENSG00000168374	ARF4	3	57557090	57583947	ENSG00000173210	ABLIM3	5	148521046	148640105	0.0097641	9.5291	4.65E-09	0.00100104	0.031242	5.9081	2.19E-05	rs17825630	1.09E-14	0	NA	NA	Artery_Tibial
ENSG00000168374	ARF4	3	57557090	57583947	ENSG00000173210	ABLIM3	5	148521046	148640105	0.0097641	9.5291	4.65E-09	0.00100104	0.031242	5.9081	2.19E-05	rs17825630	1.09E-14	0	NA	NA	Heart_Atrial_Appendage
ENSG00000172057	ORMDL3	17	38077294	38083854	ENSG00000151726	ACSL1	4	185676749	185747972	0.0097454	9.5106	4.85E-09	0.0010427	0.011653	1.7981	0.096462	rs4794820	5.16E-07	0.00445765	3.2717e-310	0	Spleen
ENSG00000168374	ARF4	3	57557090	57583947	ENSG00000198478	SH3BGRL2	6	80341000	80413372	0.0097401	9.5054	4.91E-09	0.00105328	0.025325	4.7602	0.00026662	rs17825630	2.55E-19	0	NA	NA	Artery_Tibial
ENSG00000168374	ARF4	3	57557090	57583947	ENSG00000198478	SH3BGRL2	6	80341000	80413372	0.0097401	9.5054	4.91E-09	0.00105328	0.025325	4.7602	0.00026662	rs17825630	2.55E-19	0	NA	NA	Heart_Atrial_Appendage
ENSG00000141150	RASL10B	17	34058668	34070540	ENSG00000144677	CTDSPL	3	37903451	38025960	0.011254	6.8703	5.08E-09	0.00108805	0.041166	5.6058	2.41E-06	rs9915021	1.38E-80	0	NA	NA	Cells_Transformed_fibroblasts
ENSG00000167914	GSDMA	17	38119226	38134019	ENSG00000182541	LIMK2	22	31608260	31676066	0.0078649	19.164	5.13E-09	0.00109576	0.014788	6.8972	0.0010637	rs3859192	3.66E-12	0	6.22E-13	0	Adipose_Subcutaneous
ENSG00000167914	GSDMA	17	38119226	38134019	ENSG00000182541	LIMK2	22	31608260	31676066	0.0078649	19.164	5.13E-09	0.00109576	0.014788	6.8972	0.0010637	rs3859192	3.66E-12	0	6.22E-13	0	Adipose_Visceral_Omentum
ENSG00000167914	GSDMA	17	38119226	38134019	ENSG00000182541	LIMK2	22	31608260	31676066	0.0078649	19.164	5.13E-09	0.00109576	0.014788	6.8972	0.0010637	rs3859192	3.66E-12	0	6.22E-13	0	Adrenal_Gland
ENSG00000167914	GSDMA	17	38119226	38134019	ENSG00000182541	LIMK2	22	31608260	31676066	0.0078649	19.164	5.13E-09	0.00109576	0.014788	6.8972	0.0010637	rs3859192	3.66E-12	0	6.22E-13	0	Artery_Aorta
ENSG00000167914	GSDMA	17	38119226	38134019	ENSG00000182541	LIMK2	22	31608260	31676066	0.0078649	19.164	5.13E-09	0.00109576	0.014788	6.8972	0.0010637	rs3859192	3.66E-12	0	6.22E-13	0	Artery_Coronary
ENSG00000167914	GSDMA	17	38119226	38134019	ENSG00000182541	LIMK2	22	31608260	31676066	0.0078649	19.164	5.13E-09	0.00109576	0.014788	6.8972	0.0010637	rs3859192	3.66E-12	0	6.22E-13	0	Artery_Tibial

ENSG00000167914	GSDMA	17	38119226	38134019	ENSG00000182541	LIMK2	22	31608260	31676066	0.0078649	19.164	5.13E-09	0.00109576	0.014788	6.8972	0.0010637	rs3859192	3.66E-12	0	6.22E-13	0	Breast_Mammary_Tissue
ENSG00000167914	GSDMA	17	38119226	38134019	ENSG00000182541	LIMK2	22	31608260	31676066	0.0078649	19.164	5.13E-09	0.00109576	0.014788	6.8972	0.0010637	rs3859192	3.66E-12	0	6.22E-13	0	Cells_Transformed_fibroblasts
ENSG00000167914	GSDMA	17	38119226	38134019	ENSG00000182541	LIMK2	22	31608260	31676066	0.0078649	19.164	5.13E-09	0.00109576	0.014788	6.8972	0.0010637	rs3859192	3.66E-12	0	6.22E-13	0	Colon_Sigmoid
ENSG00000167914	GSDMA	17	38119226	38134019	ENSG00000182541	LIMK2	22	31608260	31676066	0.0078649	19.164	5.13E-09	0.00109576	0.014788	6.8972	0.0010637	rs3859192	3.66E-12	0	6.22E-13	0	Colon_Transverse
ENSG00000167914	GSDMA	17	38119226	38134019	ENSG00000182541	LIMK2	22	31608260	31676066	0.0078649	19.164	5.13E-09	0.00109576	0.014788	6.8972	0.0010637	rs3859192	3.66E-12	0	6.22E-13	0	Esophagus_Gastroesophageal_Junction
ENSG00000167914	GSDMA	17	38119226	38134019	ENSG00000182541	LIMK2	22	31608260	31676066	0.0078649	19.164	5.13E-09	0.00109576	0.014788	6.8972	0.0010637	rs3859192	3.66E-12	0	6.22E-13	0	Esophagus_Muscularis
ENSG00000167914	GSDMA	17	38119226	38134019	ENSG00000182541	LIMK2	22	31608260	31676066	0.0078649	19.164	5.13E-09	0.00109576	0.014788	6.8972	0.0010637	rs3859192	3.66E-12	0	6.22E-13	0	Heart_Atrial_Appendage
ENSG00000167914	GSDMA	17	38119226	38134019	ENSG00000182541	LIMK2	22	31608260	31676066	0.0078649	19.164	5.13E-09	0.00109576	0.014788	6.8972	0.0010637	rs3859192	3.66E-12	0	6.22E-13	0	Heart_Left_Ventricle
ENSG00000167914	GSDMA	17	38119226	38134019	ENSG00000182541	LIMK2	22	31608260	31676066	0.0078649	19.164	5.13E-09	0.00109576	0.014788	6.8972	0.0010637	rs3859192	3.66E-12	0	6.22E-13	0	Liver
ENSG00000167914	GSDMA	17	38119226	38134019	ENSG00000182541	LIMK2	22	31608260	31676066	0.0078649	19.164	5.13E-09	0.00109576	0.014788	6.8972	0.0010637	rs3859192	3.66E-12	0	6.22E-13	0	Lung
ENSG00000167914	GSDMA	17	38119226	38134019	ENSG00000182541	LIMK2	22	31608260	31676066	0.0078649	19.164	5.13E-09	0.00109576	0.014788	6.8972	0.0010637	rs3859192	3.66E-12	0	6.22E-13	0	Muscle_Skeletal
ENSG00000167914	GSDMA	17	38119226	38134019	ENSG00000182541	LIMK2	22	31608260	31676066	0.0078649	19.164	5.13E-09	0.00109576	0.014788	6.8972	0.0010637	rs3859192	3.66E-12	0	6.22E-13	0	Nerve_Tibial
ENSG00000167914	GSDMA	17	38119226	38134019	ENSG00000182541	LIMK2	22	31608260	31676066	0.0078649	19.164	5.13E-09	0.00109576	0.014788	6.8972	0.0010637	rs3859192	3.66E-12	0	6.22E-13	0	Ovary
ENSG00000167914	GSDMA	17	38119226	38134019	ENSG00000182541	LIMK2	22	31608260	31676066	0.0078649	19.164	5.13E-09	0.00109576	0.014788	6.8972	0.0010637	rs3859192	3.66E-12	0	6.22E-13	0	Pancreas
ENSG00000167914	GSDMA	17	38119226	38134019	ENSG00000182541	LIMK2	22	31608260	31676066	0.0078649	19.164	5.13E-09	0.00109576	0.014788	6.8972	0.0010637	rs3859192	3.66E-12	0	6.22E-13	0	Small_Intestine_Terminal_Ileum
ENSG00000167914	GSDMA	17	38119226	38134019	ENSG00000182541	LIMK2	22	31608260	31676066	0.0078649	19.164	5.13E-09	0.00109576	0.014788	6.8972	0.0010637	rs3859192	3.66E-12	0	6.22E-13	0	Spleen
ENSG00000167914	GSDMA	17	38119226	38134019	ENSG00000182541	LIMK2	22	31608260	31676066	0.0078649	19.164	5.13E-09	0.00109576	0.014788	6.8972	0.0010637	rs3859192	3.66E-12	0	6.22E-13	0	Stomach
ENSG00000167914	GSDMA	17	38119226	38134019	ENSG00000182541	LIMK2	22	31608260	31676066	0.0078649	19.164	5.13E-09	0.00109576	0.014788	6.8972	0.0010637	rs3859192	3.66E-12	0	6.22E-13	0	Thyroid
ENSG00000167914	GSDMA	17	38119226	38134019	ENSG00000182541	LIMK2	22	31608260	31676066	0.0078649	19.164	5.13E-09	0.00109576	0.014788	6.8972	0.0010637	rs3859192	3.66E-12	0	6.22E-13	0	Whole_Blood
ENSG00000167914	GSDMA	17	38119226	38134019	ENSG00000182541	LIMK2	22	31608260	31676066	0.0078649	19.164	5.13E-09	0.00109576	0.014788	6.8972	0.0010637	rs17609240	2.35E-07	0.00223653	1.53E-144	0	Adipose_Subcutaneous
ENSG00000167914	GSDMA	17	38119226	38134019	ENSG00000182541	LIMK2	22	31608260	31676066	0.0078649	19.164	5.13E-09	0.00109576	0.014788	6.8972	0.0010637	rs17609240	2.35E-07	0.00223653	1.53E-144	0	Adipose_Visceral_Omentum
ENSG00000167914	GSDMA	17	38119226	38134019	ENSG00000182541	LIMK2	22	31608260	31676066	0.0078649	19.164	5.13E-09	0.00109576	0.014788	6.8972	0.0010637	rs17609240	2.35E-07	0.00223653	1.53E-144	0	Artery_Aorta
ENSG00000167914	GSDMA	17	38119226	38134019	ENSG00000182541	LIMK2	22	31608260	31676066	0.0078649	19.164	5.13E-09	0.00109576	0.014788	6.8972	0.0010637	rs17609240	2.35E-07	0.00223653	1.53E-144	0	Breast_Mammary_Tissue
ENSG00000167914	GSDMA	17	38119226	38134019	ENSG00000182541	LIMK2	22	31608260	31676066	0.0078649	19.164	5.13E-09	0.00109576	0.014788	6.8972	0.0010637	rs17609240	2.35E-07	0.00223653	1.53E-144	0	Colon_Sigmoid
ENSG00000167914	GSDMA	17	38119226	38134019	ENSG00000182541	LIMK2	22	31608260	31676066	0.0078649	19.164	5.13E-09	0.00109576	0.014788	6.8972	0.0010637	rs17609240	2.35E-07	0.00223653	1.53E-144	0	Esophagus_Gastroesophageal_Junction
ENSG00000167914	GSDMA	17	38119226	38134019	ENSG00000182541	LIMK2	22	31608260	31676066	0.0078649	19.164	5.13E-09	0.00109576	0.014788	6.8972	0.0010637	rs17609240	2.35E-07	0.00223653	1.53E-144	0	Esophagus_Muscularis
ENSG00000167914	GSDMA	17	38119226	38134019	ENSG00000182541	LIMK2	22	31608260	31676066	0.0078649	19.164	5.13E-09	0.00109576	0.014788	6.8972	0.0010637	rs17609240	2.35E-07	0.00223653	1.53E-144	0	Heart_Left_Ventricle
ENSG00000167914	GSDMA	17	38119226	38134019	ENSG00000182541	LIMK2	22	31608260	31676066	0.0078649	19.164	5.13E-09	0.00109576	0.014788	6.8972	0.0010637	rs17609240	2.35E-07	0.00223653	1.53E-144	0	Muscle_Skeletal
ENSG00000167914	GSDMA	17	38119226	38134019	ENSG00000182541	LIMK2	22	31608260	31676066	0.0078649	19.164	5.13E-09	0.00109576	0.014788	6.8972	0.0010637	rs3894194	1.30E-06	0.01033327	6.08E-29	0	Adipose_Subcutaneous
ENSG00000167914	GSDMA	17	38119226	38134019	ENSG00000182541	LIMK2	22	31608260	31676066	0.0078649	19.164	5.13E-09	0.00109576	0.014788	6.8972	0.0010637	rs3894194	1.30E-06	0.01033327	6.08E-29	0	Adipose_Visceral_Omentum
ENSG00000167914	GSDMA	17	38119226	38134019	ENSG00000182541	LIMK2	22	31608260	31676066	0.0078649	19.164	5.13E-09	0.00109576	0.014788	6.8972	0.0010637	rs3894194	1.30E-06	0.01033327	6.08E-29	0	Artery_Tibial
ENSG00000167914	GSDMA	17	38119226	38134019	ENSG00000182541	LIMK2	22	31608260	31676066	0.0078649	19.164	5.13E-09	0.00109576	0.014788	6.8972	0.0010637	rs3894194	1.30E-06	0.01033327	6.08E-29	0	Colon_Transverse
ENSG00000167914	GSDMA	17	38119226	38134019	ENSG00000182541	LIMK2	22	31608260	31676066	0.0078649	19.164	5.13E-09	0.00109576	0.014788	6.8972	0.0010637	rs3894194	1.30E-06	0.01033327	6.08E-29	0	Esophagus_Gastroesophageal_Junction
ENSG00000167914	GSDMA	17	38119226	38134019	ENSG00000182541	LIMK2	22	31608260	31676066	0.0078649	19.164	5.13E-09	0.00109576	0.014788	6.8972	0.0010637	rs3894194	1.30E-06	0.01033327	6.08E-29	0	Heart_Atrial_Appendage
ENSG00000167914	GSDMA	17	38119226	38134019	ENSG00000182541	LIMK2	22	31608260	31676066	0.0078649	19.164	5.13E-09	0.00109576	0.014788	6.8972	0.0010637	rs3894194	1.30E-06	0.01033327	6.08E-29	0	Muscle_Skeletal
ENSG00000167914	GSDMA	17	38119226	38134019	ENSG00000182541	LIMK2	22	31608260	31676066	0.0078649	19.164	5.13E-09	0.00109576	0.014788	6.8972	0.0010637	rs3894194	1.30E-06	0.01033327	6.08E-29	0	Whole_Blood
ENSG00000167914	GSDMA	17	38119226	38134019	ENSG00000182541	LIMK2	22	31608260	31676066	0.0078649	19.164	5.13E-09	0.00109576	0.014788	6.8972	0.0010637	rs7212938	8.17E-06	0.04925273	5.34E-44	0	Adipose_Subcutaneous
ENSG00000167914	GSDMA	17	38119226	38134019	ENSG00000182541	LIMK2	22	31608260	31676066	0.0078649	19.164	5.13E-09	0.00109576	0.014788	6.8972	0.0010637	rs7212938	8.17E-06	0.04925273	5.34E-44	0	Adipose_Visceral_Omentum
ENSG00000167914	GSDMA	17	38119226	38134019	ENSG00000182541	LIMK2	22	31608260	31676066	0.0078649	19.164	5.13E-09	0.00109576	0.014788	6.8972	0.0010637	rs7212938	8.17E-06	0.04925273	5.34E-44	0	Artery_Coronary
ENSG00000167914	GSDMA	17	38119226	38134019	ENSG00000182541	LIMK2	22	31608260	31676066	0.0078649	19.164	5.13E-09	0.00109576	0.014788	6.8972	0.0010637	rs7212938	8.17E-06	0.04925273	5.34E-44	0	Colon_Sigmoid
ENSG00000167914	GSDMA	17	38119226	38134019	ENSG00000182541	LIMK2	22	31608260	31676066	0.0078649	19.164	5.13E-09	0.00109576	0.014788	6.8972	0.0010637	rs7212938	8.17E-06	0.04925273	5.34E-44	0	Heart_Atrial_Appendage
ENSG00000167914	GSDMA	17	38119226	38134019	ENSG00000182541	LIMK2	22	31608260	31676066	0.0078649	19.164	5.13E-09	0.00109576	0.014788	6.8972	0.0010637	rs7212938	8.17E-06	0.04925273	5.34E-44	0	Heart_Left_Ventricle
ENSG00000167914	GSDMA	17	38119226	38134019	ENSG00000182541	LIMK2	22	31608260	31676066	0.0078649	19.164	5.13E-09	0.00109576	0.014788	6.8972	0.0010637	rs7212938	8.17E-06	0.04925273	5.34E-44	0	Thyroid
ENSG00000167914	GSDMA	17	38119226	38134019	ENSG00000182541	LIMK2	22	31608260	31676066	0.0078649	19.164	5.13E-09	0.00109576	0.014788	6.8972	0.0010637	rs7212938	8.17E-06	0.04925273	5.34E-44	0	Whole_Blood
ENSG00000167914	GSDMA	17	38119226	38134019	ENSG00000182541	LIMK2	22	31608260	31676066	0.0078649	19.164	5.13E-09	0.00109576	0.014788	6.8972	0.0010637	rs8078723	5.38E-13	0	6.97E-07	0.002194627	Breast_Mammary_Tissue
ENSG00000167914	GSDMA	17	38119226	38134019	ENSG00000182541	LIMK2	22	31608260	31676066	0.0078649	19.164	5.13E-09	0.00109576	0.014788	6.8972	0.0010637	rs8078723	5.38E-13	0	6.97E-07	0.002194627	Esophagus_Gastroesophageal_Junction
ENSG00000167914	GSDMA	17	38119226	38134019	ENSG00000182541	LIMK2	22	31608260	31676066	0.0078649	19.164	5.13E-09	0.00109576	0.014788	6.8972	0.0010637	rs4794820	3.89E-08	0.00052744	2.26E-66	0	Artery_Tibial
ENSG00000167914	GSDMA	17	38119226	38134019	ENSG00000182541	LIMK2	22	31608260	31676066	0.0078649	19.164	5.13E-09	0.00109576	0.014788	6.8972	0.0010637	rs4794820	3.89E-08	0.00052744	2.26E-66	0	Cells_Transformed_fibroblasts
ENSG00000167914	GSDMA	17	38119226	38134019	ENSG00000182541	LIMK2	22	31608260	31676066	0.0078649	19.164	5.13E-09	0.00109576	0.014788	6.8972	0.0010637	rs4794822	1.27E-12	0	3.02E-06	0.008756123	Breast_Mammary_Tissue
ENSG00000167914	GSDMA	17	38119226	38134019	ENSG00000182541	LIMK2	22	31608260	31676066	0.0078649	19.164	5.13E-09	0.00109576	0.014788	6.8972	0.0010637	rs4794822	1.27E-12	0	3.02E-06	0.008756123	Cells_Transformed_fibroblasts
ENSG00000167914	GSDMA	17	38119226	38134019	ENSG00000182541	LIMK2	22	31608260	31676066	0.0078649	19.164	5.13E-09	0.00109576	0.014788	6.8972	0.0010637	rs4794822	1.27E-12	0	3.02E-06	0.008756123	Esophagus_Gastroesophageal_Junction
ENSG00000167914	GSDMA	17	38119226	38134019	ENSG00000182541	LIMK2	22	31608260	31676066	0.0078649	19.164	5.13E-09	0.00109576	0.014788	6.8972	0.0010637	rs4794822	1.27E-12	0	3.02E-06	0.008756123	Esophagus_Muscularis
ENSG00000167914	GSDMA	17	38119226	38134019	ENSG00000182541	LIMK2	22	31608260	31676066	0.0078649	19.164	5.13E-09	0.00109576	0.014788	6.8972	0.0010637	rs4065321	2.17E-09	5.58E-05	2.52E-12	0	Pancreas
ENSG00000172057	ORMDL3	17	38077294	38083854	ENSG00000170525	PFKFB3	10	6186881	6277495	0.0097064	9.4722	5.31E-09	0.00113071	0.014755	2.2839	0.034036	rs7216389	2.87E-07	0.00272062	3.2717e-310	0	Adipose_Subcutaneous
ENSG00000172057	ORMDL3	17	38077294	38083854	ENSG00000170525	PFKFB3	10	6186881	6277495	0.0097064	9.4722	5.31E-09	0.00113071	0.014755	2.2839	0.034036	rs4794820	1.46E-09	5.76E-05	3.2717e-310	0	Spleen
ENSG00000172057	ORMDL3	17	38077294	38083854	ENSG00000170525	PFKFB3	10	6186881	6277495	0.0097064	9.4722	5.31E-09	0.00113071	0.014755	2.2839	0.034036	rs2872507	2.73E-07	0.00263673	3.2717e-310	0	Whole_Blood
ENSG00000172057	ORMDL3	17	38077294	38083854	ENSG00000170525	PFKFB3	10	6186881	6277495	0.0097064	9.4722	5.31E-09	0.00113071	0.014755	2.2839	0.034036	rs8067378	2.67E-07	0.00257357	3.2717e-310	0	Cells_EBV-transformed_lymphocytes
ENSG00000172057	ORMDL3	17	38077294	38083854	ENSG00000170525	PFKFB3	10	6186881	6277495	0.0097064	9.4722	5.31E-09	0.00113071	0.014755	2.2839	0.034036	rs8067378	2.67E-07	0.00257357	3.2717e-310	0	Spleen
ENSG00000172057	ORMDL3	17	38077294	38083854	ENSG00000170525	PFKFB3	10	6186881	6277495	0.0097064	9.4722	5.31E-09	0.00113071	0.014755	2.2839	0.034036	rs8067378	2.67E-07	0.00257357	3.2717e-310	0	Whole_Blood
ENSG00000172057	ORMDL3	17	38077294	38083854	ENSG00000170525	PFKFB3	10	6186881	6277495	0.0097064	9.4722	5.31E-09	0.00113071	0.014755	2.2839	0.034036	rs9303277	2.53E-07	0.00247042	3.2717e-310	0	Cells_EBV-transformed_lymphocytes
ENSG00000172057	ORMDL3	17	38077294	38083854	ENSG00000170525	PFKFB3	10	6186881	6277495	0.0097064	9.4722	5.31E-09	0.00113071	0.014755	2.2839	0.034036	rs9303277	2.53E-07	0.00247042	3.2717e-310	0	Skin_Sun_Exposed_Lower_leg
ENSG00000172057	ORMDL3	17	38077294	38083854	ENSG00000170525	PFKFB3	10	6186881	6277495	0.0097064	9.4722	5.31E-09	0.00113071	0.014755	2.2839	0.034036	rs8069176	3.56E-08	0.00046043	3.2717e-310	0	Whole_Blood
ENSG00000172057	ORMDL3	17	38077294	38083854	ENSG00000170525	PFKFB3	10	6186881	6277495	0.0097064	9.4722	5.31E-09	0.00113071	0.014755	2.2839	0.034036	rs2290400	1.05E-07	0.00119845	3.2717e-310	0	Cells_EBV-transformed_lymphocytes
ENSG00000172057	ORMDL3	17	38077294	38083854	ENSG00000170525	PFKFB3	10	6186881	6277495	0.0097064	9.4722	5.31E-09	0.00113071	0.014755	2.2839	0.034036	rs2290400	1.05E-07	0.00119845	3.2717e-310	0	Esophagus_Muscularis
ENSG00000172057	ORMDL3	17	38077294	38083854	ENSG00000170525	PFKFB3	10	6186881	6277495	0.0097064	9.4722	5.31E-09	0.00113071	0.014755	2.2839	0.034036	rs2290400	1.05E-07	0.00119845	3.2717e-310	0	Lung
ENSG00000172057	ORMDL3	17	38077294	38083854	ENSG00000170525	PFKFB3	10	6186881	6277495	0.0097064	9.4722	5.31E-09	0.00113071	0.014755	2.2839	0.034036	rs2290400	1.05E-07	0.00119845	3.2717e-310	0	Skin_Sun_Exposed_Lower_leg
ENSG00000008838	MED24	17	38175350	38217468	ENSG00000196663	TECPR2	14	102829300	102968818	0.0091388	11.144	5.35E-09	0.00113738	0.013454	3.1265	0.014393	rs12946510	4.63E-07	0.00409677	NA	NA	Prostate
ENSG00000008838	MED24	17	38175350	38217468	ENSG00000196663	TECPR2	14	102829300	102968818	0.0091388	11.144	5.35E-09	0.00113738	0.013454	3.1265	0.014393	rs3859192	4.25E-17	0	1.47E-40	0	Whole_Blood
ENSG00000008838	MED24	17	38175350	38217468	ENSG00000196663	TECPR2	14	102829300	102968818	0.0091388	11.144	5.35E-09	0.00113738	0.013454	3.1265	0.014393	rs3894194	7.36E-09	0.0001505	8.18E-15	0	Thyroid
ENSG00000008838	MED24	17	38175350	38217468	ENSG00000196663	TECPR2	14	102829300	102968818	0.0091388	11.144	5.35E-09	0.00113738	0.013454	3.1265	0.014393	rs7212938	6.67E-07	0.0058136	4.22E-10	0	Testis
ENSG00000008838	MED24	17	38175350	38217468	ENSG00000196663	TECPR2	14	102829300	102968818	0.0091388	11.144	5.35E-09	0.00113738	0.013454	3.1265	0.014393	rs7212938	6.67E-07	0.0058136	4.22E-10	0	Thyroid
ENSG00000008838	MED24	17	38175350	38217468	ENSG00000196663	TECPR2	14	102829300	102968818	0.0091388	11.144	5.35E-09	0.00113738	0.013454	3.1265	0.014393	rs8078723	1.05E-21	0	5.30E-78	0	Adipose_Subcutaneous
ENSG00000008838	MED24	17	38175350	38217468	ENSG00000196663	TECPR2	14	102829300	102968818	0.0091388	11.144	5.35E-09	0.00113738	0.013454	3.1265	0.014393	rs8078723	1.05E-21	0	5.30E-78	0	Artery_Tibial
ENSG00000008838	MED24	17	38175350	38217468	ENSG00000196663	TECPR2	14	102829300	102968818	0.0091388	11.144	5.35E-09	0.00113738	0.013454	3.1265	0.014393	rs8078723	1.05E-21	0	5.30E-78	0	Cells_Transformed_fibroblasts
ENSG00000008838	MED24	17	38175350	38217468	ENSG00000196663	TECPR2	14	102829300	102968818	0.0091388	11.144	5.35E-09	0.00113738	0.013454	3.1265	0.014393	rs8078723	1.05E-21	0	5.30E-78	0	Lung
ENSG00000008838	MED24	17	38175350	38217468	ENSG00000196663	TECPR2	14	102829300	102968818	0.0091388	11.144	5.35E-09	0.00113738	0.013454	3.1265	0.014393	rs8078723	1.05E-21	0	5.30E-78	0	Pituitary
ENSG00000008838	MED24	17	38175350	38217468	ENSG00000196663	TECPR2	14	102829300	102968818	0.0091388	11.144	5.35E-09	0.00113738	0.013454	3.1265	0.014393	rs8078723	1.05E-21	0	5.30E-78	0	Skin_Sun_Exposed_Lower_leg
ENSG00000008838	MED24	17	38175350	38217468	ENSG00000196663	TECPR2	14	102829300	102968818	0.0091388	11.144	5.35E-09	0.00113738	0.013454	3.1265	0.014393	rs8078723	1.05E-21	0	5.30E-78	0	Spleen
ENSG00000008838	MED24	17	38175350	38217468	ENSG00000196663	TECPR2	14	102829300	102968818	0.0091388	11.144	5.35E-09	0.00113738	0.013454	3.1265	0.014393	rs8078723	1.05E-21	0	5.30E-78	0	Stomach
ENSG00000008838	MED24	17	38175350	38217468	ENSG00000196663	TECPR2	14	102829300	102968818	0.0091388	11.144	5.35E-09	0.00113738	0.013454	3.1265	0.014393	rs8078723	1.05E-21	0	5.30E-78	0	Thyroid
ENSG00000008838	MED24	17	38175350	38217468	ENSG00000196663	TECPR2	14	102829300	102968818	0.0091388	11.144	5.35E-09	0.00113738	0.013454	3.1265	0.014393	rs4794822	2.44E-21	0	1.32E-79	0	Esophagus_Gastroesophageal_Junction
ENSG00000008838	MED24	17	38175350	38217468	ENSG00000196663	TECPR2	14	102829300	102968818	0.0091388	11.144	5.35E-09	0.00113738	0.013454	3.1265	0.014393	rs4794822	2.44E-21	0	1.32E-79	0	Lung

ENSG00000008838	MED24	17	38175350	38217468	ENSG00000196663	TECPR2	14	102829300	102968818	0.0091388	11.144	5.35E-09	0.00113738	0.013454	3.1265	0.014393	rs4794822	2.44E-21	0	1.32E-79	0	Spleen
ENSG00000008838	MED24	17	38175350	38217468	ENSG00000196663	TECPR2	14	102829300	102968818	0.0091388	11.144	5.35E-09	0.00113738	0.013454	3.1265	0.014393	rs4794822	2.44E-21	0	1.32E-79	0	Whole_Blood
ENSG00000008838	MED24	17	38175350	38217468	ENSG00000196663	TECPR2	14	102829300	102968818	0.0091388	11.144	5.35E-09	0.00113738	0.013454	3.1265	0.014393	rs4065321	1.47E-17	0	1.24E-42	0	Pituitary
ENSG00000111540	RAB5B	12	56367697	56388490	ENSG00000105085	MED26	19	16698215	16739873	0.010229	8.321	5.42E-09	0.00115175	0.011433	1.5101	0.16003	rs10876864	6.01E-06	0.03846264	5.79E-06	0.016449853	Muscle_Skeletal
ENSG00000172660	TAF15	17	34136459	34174246	ENSG00000113140	SPARC	5	151040657	151066726	0.01022	8.3137	5.53E-09	0.0011727	0.011229	1.7319	0.11047	rs9915021	8.30E-44	0	NA	NA	Skin_Not_Sun_Exposed_Suprapubic
ENSG00000180376	CCDC66	3	56591189	56653929	ENSG00000185052	SLC24A3	20	19193290	19703581	0.0096614	9.4279	5.88E-09	0.00124565	0.01182	2.1913	0.053209	rs17825630	8.36E-20	0	NA	NA	Brain_Anterior_cingulate_cortex_BA24
ENSG00000180376	CCDC66	3	56591189	56653929	ENSG00000185052	SLC24A3	20	19193290	19703581	0.0096614	9.4279	5.88E-09	0.00124565	0.01182	2.1913	0.053209	rs17825630	8.36E-20	0	NA	NA	Cells_EBV-transformed_lymphocytes
ENSG00000180376	CCDC66	3	56591189	56653929	ENSG00000185052	SLC24A3	20	19193290	19703581	0.0096614	9.4279	5.88E-09	0.00124565	0.01182	2.1913	0.053209	rs1354034	3.27170000000001e-	0	NA	NA	Brain_Cerebellar_Hemisphere
ENSG00000180376	CCDC66	3	56591189	56653929	ENSG00000185052	SLC24A3	20	19193290	19703581	0.0096614	9.4279	5.88E-09	0.00124565	0.01182	2.1913	0.053209	rs1354034	3.27170000000001e-	0	NA	NA	Cells_EBV-transformed_lymphocytes
ENSG00000180376	CCDC66	3	56591189	56653929	ENSG00000185052	SLC24A3	20	19193290	19703581	0.0096614	9.4279	5.88E-09	0.00124565	0.01182	2.1913	0.053209	rs1354034	3.27170000000001e-	0	NA	NA	Testis
ENSG00000132139	GAS2L2	17	34071530	34079897	ENSG00000204463	BAG6	6	31606805	31620170	0.011184	6.8275	5.91E-09	0.00125033	0.01735	2.015	0.041924	rs9915021	2.40E-12	0	NA	NA	Small_Intestine_Terminal_Ileum
ENSG00000163946	FAM208A	3	56658507	56717265	ENSG00000183785	TUBA8	22	18593097	18593634	0.011181	6.8253	5.96E-09	0.00125899	0.034877	4.7185	3.23E-05	rs12485738	1.06E-25	0	NA	NA	Esophagus_Mucosa
ENSG00000163946	FAM208A	3	56658507	56717265	ENSG00000183785	TUBA8	22	18593097	18593634	0.011181	6.8253	5.96E-09	0.00125899	0.034877	4.7185	3.23E-05	rs1354034	4.07E-59	0	NA	NA	Heart_Atrial_Appendage
ENSG00000131771	PPP1R1B	17	37782993	37792879	ENSG00000146592	CREB5	7	28338940	28865511	0.011646	6.3211	6.03E-09	0.00127051	0.0033377	0.33935	0.96184	rs3859192	1.81E-15	0	NA	NA	Adipose_Subcutaneous
ENSG00000131771	PPP1R1B	17	37782993	37792879	ENSG00000146592	CREB5	7	28338940	28865511	0.011646	6.3211	6.03E-09	0.00127051	0.0033377	0.33935	0.96184	rs3894194	4.69E-11	0	NA	NA	Heart_Atrial_Appendage
ENSG00000131771	PPP1R1B	17	37782993	37792879	ENSG00000146592	CREB5	7	28338940	28865511	0.011646	6.3211	6.03E-09	0.00127051	0.0033377	0.33935	0.96184	rs4794820	2.26E-09	7.41E-05	NA	NA	Heart_Left_Ventricle
ENSG00000131771	PPP1R1B	17	37782993	37792879	ENSG00000146592	CREB5	7	28338940	28865511	0.011646	6.3211	6.03E-09	0.00127051	0.0033377	0.33935	0.96184	rs9303280	8.57E-07	0.00721415	NA	NA	Heart_Atrial_Appendage
ENSG00000172716	SLFN11	17	33677324	33700720	ENSG00000138867	GUCD1	22	24936406	24951284	0.012971	5.2838	6.22E-09	0.00130733	NA	NA	NA	rs225245	4.32E-38	0	5.77E-07	0.001884469	Cells_Transformed_fibroblasts
ENSG00000172716	SLFN11	17	33677324	33700720	ENSG00000138867	GUCD1	22	24936406	24951284	0.012971	5.2838	6.22E-09	0.00130733	NA	NA	NA	rs8082605	2.66E-13	0	NA	NA	Adrenal_Gland
ENSG00000239388	ASB14	3	57310556	57326710	ENSG00000259207	ITGB3	17	45387505	45389182	0.0096146	9.3818	6.55E-09	0.00136963	0.015797	2.9405	0.012167	rs17825630	4.73E-14	0	NA	NA	Skin_Not_Sun_Exposed_Suprapubic
ENSG00000239388	ASB14	3	57310556	57326710	ENSG00000259207	ITGB3	17	45387505	45389182	0.0096146	9.3818	6.55E-09	0.00136963	0.015797	2.9405	0.012167	rs12485738	2.40E-225	0	NA	NA	Artery_Tibial
ENSG00000239388	ASB14	3	57310556	57326710	ENSG00000259207	ITGB3	17	45387505	45389182	0.0096146	9.3818	6.55E-09	0.00136963	0.015797	2.9405	0.012167	rs1354034	3.27170000000001e-	0	NA	NA	Skin_Not_Sun_Exposed_Suprapubic
ENSG00000205045	SLFN12L	17	33800708	33864880	ENSG00000197993	KEL	7	142638201	142659768	0.01065	7.4275	6.56E-09	0.00137	0.021452	2.8625	0.0058465	rs8082605	1.52E-07	0.00160514	2.49E-31	0	Adipose_Subcutaneous
ENSG00000205045	SLFN12L	17	33800708	33864880	ENSG00000197993	KEL	7	142638201	142659768	0.01065	7.4275	6.56E-09	0.00137	0.021452	2.8625	0.0058465	rs8082605	1.52E-07	0.00160514	2.49E-31	0	Adipose_Visceral_Omentum
ENSG00000205045	SLFN12L	17	33800708	33864880	ENSG00000197993	KEL	7	142638201	142659768	0.01065	7.4275	6.56E-09	0.00137	0.021452	2.8625	0.0058465	rs8082605	1.52E-07	0.00160514	2.49E-31	0	Heart_Left_Ventricle
ENSG00000205045	SLFN12L	17	33800708	33864880	ENSG00000197993	KEL	7	142638201	142659768	0.01065	7.4275	6.56E-09	0.00137	0.021452	2.8625	0.0058465	rs8082605	1.52E-07	0.00160514	2.49E-31	0	Nerve_Tibial
ENSG00000205045	SLFN12L	17	33800708	33864880	ENSG00000197993	KEL	7	142638201	142659768	0.01065	7.4275	6.56E-09	0.00137	0.021452	2.8625	0.0058465	rs10512472	7.82E-19	0	4.96E-30	0	Pancreas
ENSG00000100138	NHP2L1	22	42069934	42086508	ENSG00000175556	LONRF3	X	118108581	118156888	0.010124	8.2349	6.87E-09	0.00142872	0.01983	3.0852	0.0053784	rs4822024	1.77E-35	0	9.12E-84	0	Artery_Aorta
ENSG00000141150	RASL10B	17	34058668	34070540	ENSG00000118046	STK11	19	1189406	1226662	0.011116	6.7852	6.86E-09	0.00142872	0.0029437	0.38549	0.91115	rs9915021	3.14E-08	0.00040834	NA	NA	Cells_Transformed_fibroblasts
ENSG00000177951	BET1L	11	167784	207428	ENSG00000174175	SELP	1	169558087	169599431	0.011113	6.7834	6.91E-09	0.00143636	0.011728	0.98174	0.46114	rs11602954	2.72E-34	0	9.12E-206	0	Adipose_Visceral_Omentum
ENSG00000177951	BET1L	11	167784	207428	ENSG00000174175	SELP	1	169558087	169599431	0.011113	6.7834	6.91E-09	0.00143636	0.011728	0.98174	0.46114	rs11602954	2.72E-34	0	9.12E-206	0	Artery_Aorta
ENSG00000177951	BET1L	11	167784	207428	ENSG00000174175	SELP	1	169558087	169599431	0.011113	6.7834	6.91E-09	0.00143636	0.011728	0.98174	0.46114	rs11602954	2.72E-34	0	9.12E-206	0	Cells_Transformed_fibroblasts
ENSG00000177951	BET1L	11	167784	207428	ENSG00000174175	SELP	1	169558087	169599431	0.011113	6.7834	6.91E-09	0.00143636	0.011728	0.98174	0.46114	rs11602954	2.72E-34	0	9.12E-206	0	Pituitary
ENSG00000177951	BET1L	11	167784	207428	ENSG00000174175	SELP	1	169558087	169599431	0.011113	6.7834	6.91E-09	0.00143636	0.011728	0.98174	0.46114	rs11602954	2.72E-34	0	9.12E-206	0	Prostate
ENSG00000177951	BET1L	11	167784	207428	ENSG00000174175	SELP	1	169558087	169599431	0.011113	6.7834	6.91E-09	0.00143636	0.011728	0.98174	0.46114	rs11602954	2.72E-34	0	9.12E-206	0	Small_Intestine_Terminal_Ileum
ENSG00000177951	BET1L	11	167784	207428	ENSG00000174175	SELP	1	169558087	169599431	0.011113	6.7834	6.91E-09	0.00143636	0.011728	0.98174	0.46114	rs11602954	2.72E-34	0	9.12E-206	0	Whole_Blood
ENSG00000177951	BET1L	11	167784	207428	ENSG00000174175	SELP	1	169558087	169599431	0.011113	6.7834	6.91E-09	0.00143636	0.011728	0.98174	0.46114	rs17655730	4.69E-37	0	2.96E-166	0	Prostate
ENSG00000177951	BET1L	11	167784	207428	ENSG00000174175	SELP	1	169558087	169599431	0.011113	6.7834	6.91E-09	0.00143636	0.011728	0.98174	0.46114	rs505404	2.87E-40	0	3.73E-187	0	Small_Intestine_Terminal_Ileum
ENSG00000177951	BET1L	11	167784	207428	ENSG00000174175	SELP	1	169558087	169599431	0.011113	6.7834	6.91E-09	0.00143636	0.011728	0.98174	0.46114	rs505404	2.87E-40	0	3.73E-187	0	Whole_Blood
ENSG00000177951	BET1L	11	167784	207428	ENSG00000185245	GP1BA	17	4835592	4838325	0.011097	6.7734	7.16E-09	0.00148619	0.019298	1.6279	0.085849	rs11602954	3.88E-45	0	9.12E-206	0	Adipose_Visceral_Omentum
ENSG00000177951	BET1L	11	167784	207428	ENSG00000185245	GP1BA	17	4835592	4838325	0.011097	6.7734	7.16E-09	0.00148619	0.019298	1.6279	0.085849	rs11602954	3.88E-45	0	9.12E-206	0	Artery_Aorta
ENSG00000177951	BET1L	11	167784	207428	ENSG00000185245	GP1BA	17	4835592	4838325	0.011097	6.7734	7.16E-09	0.00148619	0.019298	1.6279	0.085849	rs11602954	3.88E-45	0	9.12E-206	0	Cells_Transformed_fibroblasts
ENSG00000177951	BET1L	11	167784	207428	ENSG00000185245	GP1BA	17	4835592	4838325	0.011097	6.7734	7.16E-09	0.00148619	0.019298	1.6279	0.085849	rs11602954	3.88E-45	0	9.12E-206	0	Pituitary
ENSG00000177951	BET1L	11	167784	207428	ENSG00000185245	GP1BA	17	4835592	4838325	0.011097	6.7734	7.16E-09	0.00148619	0.019298	1.6279	0.085849	rs11602954	3.88E-45	0	9.12E-206	0	Prostate
ENSG00000177951	BET1L	11	167784	207428	ENSG00000185245	GP1BA	17	4835592	4838325	0.011097	6.7734	7.16E-09	0.00148619	0.019298	1.6279	0.085849	rs11602954	3.88E-45	0	9.12E-206	0	Small_Intestine_Terminal_Ileum
ENSG00000177951	BET1L	11	167784	207428	ENSG00000185245	GP1BA	17	4835592	4838325	0.011097	6.7734	7.16E-09	0.00148619	0.019298	1.6279	0.085849	rs11602954	3.88E-45	0	9.12E-206	0	Whole_Blood
ENSG00000177951	BET1L	11	167784	207428	ENSG00000185245	GP1BA	17	4835592	4838325	0.011097	6.7734	7.16E-09	0.00148619	0.019298	1.6279	0.085849	rs17655730	4.78E-41	0	2.96E-166	0	Prostate
ENSG00000177951	BET1L	11	167784	207428	ENSG00000185245	GP1BA	17	4835592	4838325	0.011097	6.7734	7.16E-09	0.00148619	0.019298	1.6279	0.085849	rs505404	2.82E-47	0	3.73E-187	0	Small_Intestine_Terminal_Ileum
ENSG00000177951	BET1L	11	167784	207428	ENSG00000185245	GP1BA	17	4835592	4838325	0.011097	6.7734	7.16E-09	0.00148619	0.019298	1.6279	0.085849	rs505404	2.82E-47	0	3.73E-187	0	Whole_Blood
ENSG00000177951	BET1L	11	167784	207428	ENSG00000185245	GP1BA	17	4835592	4838325	0.011097	6.7734	7.16E-09	0.00148619	0.019298	1.6279	0.085849	rs3782123	3.24E-06	0.02309107	NA	NA	Heart_Atrial_Appendage
ENSG00000177951	BET1L	11	167784	207428	ENSG00000138798	EGF	4	110834040	110933422	0.011082	6.7645	7.39E-09	0.00153254	0.016157	1.3585	0.18732	rs11602954	3.92E-10	2.13E-05	9.12E-206	0	Adipose_Visceral_Omentum
ENSG00000177951	BET1L	11	167784	207428	ENSG00000138798	EGF	4	110834040	110933422	0.011082	6.7645	7.39E-09	0.00153254	0.016157	1.3585	0.18732	rs11602954	3.92E-10	2.13E-05	9.12E-206	0	Artery_Aorta
ENSG00000177951	BET1L	11	167784	207428	ENSG00000138798	EGF	4	110834040	110933422	0.011082	6.7645	7.39E-09	0.00153254	0.016157	1.3585	0.18732	rs11602954	3.92E-10	2.13E-05	9.12E-206	0	Cells_Transformed_fibroblasts
ENSG00000177951	BET1L	11	167784	207428	ENSG00000138798	EGF	4	110834040	110933422	0.011082	6.7645	7.39E-09	0.00153254	0.016157	1.3585	0.18732	rs11602954	3.92E-10	2.13E-05	9.12E-206	0	Pituitary
ENSG00000177951	BET1L	11	167784	207428	ENSG00000138798	EGF	4	110834040	110933422	0.011082	6.7645	7.39E-09	0.00153254	0.016157	1.3585	0.18732	rs11602954	3.92E-10	2.13E-05	9.12E-206	0	Prostate
ENSG00000177951	BET1L	11	167784	207428	ENSG00000138798	EGF	4	110834040	110933422	0.011082	6.7645	7.39E-09	0.00153254	0.016157	1.3585	0.18732	rs11602954	3.92E-10	2.13E-05	9.12E-206	0	Small_Intestine_Terminal_Ileum
ENSG00000177951	BET1L	11	167784	207428	ENSG00000138798	EGF	4	110834040	110933422	0.011082	6.7645	7.39E-09	0.00153254	0.016157	1.3585	0.18732	rs11602954	3.92E-10	2.13E-05	9.12E-206	0	Whole_Blood
ENSG00000177951	BET1L	11	167784	207428	ENSG00000138798	EGF	4	110834040	110933422	0.011082	6.7645	7.39E-09	0.00153254	0.016157	1.3585	0.18732	rs17655730	3.08E-11	0	2.96E-166	0	Prostate
ENSG00000177951	BET1L	11	167784	207428	ENSG00000138798	EGF	4	110834040	110933422	0.011082	6.7645	7.39E-09	0.00153254	0.016157	1.3585	0.18732	rs505404	2.92E-13	0	3.73E-187	0	Small_Intestine_Terminal_Ileum
ENSG00000177951	BET1L	11	167784	207428	ENSG00000138798	EGF	4	110834040	110933422	0.011082	6.7645	7.39E-09	0.00153254	0.016157	1.3585	0.18732	rs505404	2.92E-13	0	3.73E-187	0	Whole_Blood
ENSG00000172660	TAF15	17	34136459	34174246	ENSG00000197993	KEL	7	142638201	142659768	0.01008	8.1991	7.57E-09	0.00156787	0.016831	2.6107	0.016286	rs9915021	1.56E-12	0	NA	NA	Skin_Not_Sun_Exposed_Suprapubic
ENSG00000186075	ZPBP2	17	38024417	38034149	ENSG00000131873	CHSY1	15	101715928	101792137	0.0068721	33.463	7.72E-09	0.00159141	0.0027382	2.5261	0.11232	rs10445308	2.69E-06	0.01978358	NA	NA	Testis
ENSG00000186075	ZPBP2	17	38024417	38034149	ENSG00000131873	CHSY1	15	101715928	101792137	0.0068721	33.463	7.72E-09	0.00159141	0.0027382	2.5261	0.11232	rs3859192	2.59E-12	0	NA	NA	Testis
ENSG00000186075	ZPBP2	17	38024417	38034149	ENSG00000131873	CHSY1	15	101715928	101792137	0.0068721	33.463	7.72E-09	0.00159141	0.0027382	2.5261	0.11232	rs4794820	1.44E-06	0.01124255	NA	NA	Testis
ENSG00000108306	FBXL20	17	37415384	37558776	ENSG00000162551	ALPL	1	21835858	21904905	0.0095112	9.2799	8.30E-09	0.00169073	0.0078733	1.4538	0.20254	rs12946510	8.70E-07	0.00729639	NA	NA	Brain_Nucleus_accumbens_basal_ganglia
ENSG00000108306	FBXL20	17	37415384	37558776	ENSG00000162551	ALPL	1	21835858	21904905	0.0095112	9.2799	8.30E-09	0.00169073	0.0078733	1.4538	0.20254	rs7212938	1.21E-12	0	NA	NA	Brain_Nucleus_accumbens_basal_ganglia
ENSG00000108306	FBXL20	17	37415384	37558776	ENSG00000162551	ALPL	1	21835858	21904905	0.0095112	9.2799	8.30E-09	0.00169073	0.0078733	1.4538	0.20254	rs9303277	3.21E-07	0.00291306	NA	NA	Brain_Nucleus_accumbens_basal_ganglia
ENSG00000167914	GSDMA	17	38119226	38134019	ENSG00000151726	ACSL1	4	185676749	185747972	0.0076655	18.675	8.33E-09	0.0016963	0.01203	5.595	0.0038442	rs3859192	1.10E-13	0	6.22E-13	0	Adipose_Subcutaneous
ENSG00000167914	GSDMA	17	38119226	38134019	ENSG00000151726	ACSL1	4	185676749	185747972	0.0076655	18.675	8.33E-09	0.0016963	0.01203	5.595	0.0038442	rs3859192	1.10E-13	0	6.22E-13	0	Adipose_Visceral_Omentum
ENSG00000167914	GSDMA	17	38119226	38134019	ENSG00000151726	ACSL1	4	185676749	185747972	0.0076655	18.675	8.33E-09	0.0016963	0.01203	5.595	0.0038442	rs3859192	1.10E-13	0	6.22E-13	0	Adrenal_Gland
ENSG00000167914	GSDMA	17	38119226	38134019	ENSG00000151726	ACSL1	4	185676749	185747972	0.0076655	18.675	8.33E-09	0.0016963	0.01203	5.595	0.0038442	rs3859192	1.10E-13	0	6.22E-13	0	Artery_Aorta
ENSG00000167914	GSDMA	17	38119226	38134019	ENSG00000151726	ACSL1	4	185676749	185747972	0.0076655	18.675	8.33E-09	0.0016963	0.01203	5.595	0.0038442	rs3859192	1.10E-13	0	6.22E-13	0	Artery_Coronary
ENSG00000167914	GSDMA	17	38119226	38134019	ENSG00000151726	ACSL1	4	185676749	185747972	0.0076655	18.675	8.33E-09	0.0016963	0.01203	5.595	0.0038442	rs3859192	1.10E-13	0	6.22E-13	0	Artery_Tibial
ENSG00000167914	GSDMA	17	38119226	38134019	ENSG00000151726	ACSL1	4	185676749	185747972	0.0076655	18.675	8.33E-09	0.0016963	0.01203	5.595	0.0038442	rs3859192	1.10E-13	0	6.22E-13	0	Breast_Mammary_Tissue
ENSG00000167914	GSDMA	17	38119226	38134019	ENSG00000151726	ACSL1	4	185676749	185747972	0.0076655	18.675	8.33E-09	0.0016963	0.01203	5.595	0.0038442	rs3859192	1.10E-13	0	6.22E-13	0	Cells_Transformed_fibroblasts
ENSG00000167914	GSDMA	17	38119226	38134019	ENSG00000151726	ACSL1	4	185676749	185747972	0.0076655	18.675	8.33E-09	0.0016963	0.01203	5.595	0.0038442	rs3859192	1.10E-13	0	6.22E-13	0	Colon_Sigmoid
ENSG00000167914	GSDMA	17	38119226	38134019	ENSG00000151726	ACSL1	4	185676749	185747972	0.0076655	18.675	8.33E-09	0.0016963	0.01203	5.595	0.0038442	rs3859192	1.10E-13	0	6.22E-13	0	Colon_Transverse
ENSG00000167914	GSDMA	17	38119226	38134019	ENSG00000151726	ACSL1	4	185676749	185747972	0.0076655	18.675	8.33E-09	0.0016963	0.01203	5.595	0.0038442	rs3859192	1.10E-13	0	6.22E-13	0	Esophagus_Gastroesophageal_Junction
ENSG00000167914	GSDMA	17	38119226	38134019	ENSG00000151726	ACSL1	4	185676749	185747972	0.0076655	18.675	8.33E-09	0.0016963	0.01203	5.595	0.0038442	rs3859192	1.10E-13	0	6.22E-13	0	Esophagus_Muscularis
ENSG00000167914	GSDMA	17	38119226	38134019	ENSG00000151726	ACSL1	4	185676749	185747972	0.0076655	18.675	8.33E-09	0.0016963	0.01203	5.595	0.0038442	rs3859192	1.10E-13	0	6.22E-13	0	Heart_Atrial_Appendage
ENSG00000167914	GSDMA	17	38119226	38134019	ENSG00000151726	ACSL1	4	185676749	185747972	0.0076655	18.675	8.33E-09	0.0016963	0.01203	5.595	0.0038442	rs3859192	1.10E-13	0	6.22E-13	0	Heart_Left_Ventricle
ENSG00000167914	GSDMA	17	38119226	38134019	ENSG00000151726	ACSL1	4	185676749	185747972	0.0076655	18.675	8.33E-09	0.0016963	0.01203	5.595	0.0038442	rs3859192	1.10E-13	0	6.22E-13	0	Liver

ENSG00000167914	GSDMA	17	38119226	38134019	ENSG00000151726	ACSL1	4	185676749	185747972	0.0076655	18.675	8.33E-09	0.0016963	0.01203	5.595	0.0038442	rs3859192	1.10E-13	0	6.22E-13	0	Lung
ENSG00000167914	GSDMA	17	38119226	38134019	ENSG00000151726	ACSL1	4	185676749	185747972	0.0076655	18.675	8.33E-09	0.0016963	0.01203	5.595	0.0038442	rs3859192	1.10E-13	0	6.22E-13	0	Muscle_Skeletal
ENSG00000167914	GSDMA	17	38119226	38134019	ENSG00000151726	ACSL1	4	185676749	185747972	0.0076655	18.675	8.33E-09	0.0016963	0.01203	5.595	0.0038442	rs3859192	1.10E-13	0	6.22E-13	0	Nerve_Tibial
ENSG00000167914	GSDMA	17	38119226	38134019	ENSG00000151726	ACSL1	4	185676749	185747972	0.0076655	18.675	8.33E-09	0.0016963	0.01203	5.595	0.0038442	rs3859192	1.10E-13	0	6.22E-13	0	Ovary
ENSG00000167914	GSDMA	17	38119226	38134019	ENSG00000151726	ACSL1	4	185676749	185747972	0.0076655	18.675	8.33E-09	0.0016963	0.01203	5.595	0.0038442	rs3859192	1.10E-13	0	6.22E-13	0	Pancreas
ENSG00000167914	GSDMA	17	38119226	38134019	ENSG00000151726	ACSL1	4	185676749	185747972	0.0076655	18.675	8.33E-09	0.0016963	0.01203	5.595	0.0038442	rs3859192	1.10E-13	0	6.22E-13	0	Small_Intestine_Terminal_Ileum
ENSG00000167914	GSDMA	17	38119226	38134019	ENSG00000151726	ACSL1	4	185676749	185747972	0.0076655	18.675	8.33E-09	0.0016963	0.01203	5.595	0.0038442	rs3859192	1.10E-13	0	6.22E-13	0	Spleen
ENSG00000167914	GSDMA	17	38119226	38134019	ENSG00000151726	ACSL1	4	185676749	185747972	0.0076655	18.675	8.33E-09	0.0016963	0.01203	5.595	0.0038442	rs3859192	1.10E-13	0	6.22E-13	0	Stomach
ENSG00000167914	GSDMA	17	38119226	38134019	ENSG00000151726	ACSL1	4	185676749	185747972	0.0076655	18.675	8.33E-09	0.0016963	0.01203	5.595	0.0038442	rs3859192	1.10E-13	0	6.22E-13	0	Thyroid
ENSG00000167914	GSDMA	17	38119226	38134019	ENSG00000151726	ACSL1	4	185676749	185747972	0.0076655	18.675	8.33E-09	0.0016963	0.01203	5.595	0.0038442	rs3859192	1.10E-13	0	6.22E-13	0	Whole_Blood
ENSG00000167914	GSDMA	17	38119226	38134019	ENSG00000151726	ACSL1	4	185676749	185747972	0.0076655	18.675	8.33E-09	0.0016963	0.01203	5.595	0.0038442	rs17609240	4.58E-08	0.00063158	1.53E-144	0	Adipose_Subcutaneous
ENSG00000167914	GSDMA	17	38119226	38134019	ENSG00000151726	ACSL1	4	185676749	185747972	0.0076655	18.675	8.33E-09	0.0016963	0.01203	5.595	0.0038442	rs17609240	4.58E-08	0.00063158	1.53E-144	0	Adipose_Visceral_Omentum
ENSG00000167914	GSDMA	17	38119226	38134019	ENSG00000151726	ACSL1	4	185676749	185747972	0.0076655	18.675	8.33E-09	0.0016963	0.01203	5.595	0.0038442	rs17609240	4.58E-08	0.00063158	1.53E-144	0	Artery_Aorta
ENSG00000167914	GSDMA	17	38119226	38134019	ENSG00000151726	ACSL1	4	185676749	185747972	0.0076655	18.675	8.33E-09	0.0016963	0.01203	5.595	0.0038442	rs17609240	4.58E-08	0.00063158	1.53E-144	0	Breast_Mammary_Tissue
ENSG00000167914	GSDMA	17	38119226	38134019	ENSG00000151726	ACSL1	4	185676749	185747972	0.0076655	18.675	8.33E-09	0.0016963	0.01203	5.595	0.0038442	rs17609240	4.58E-08	0.00063158	1.53E-144	0	Colon_Sigmoid
ENSG00000167914	GSDMA	17	38119226	38134019	ENSG00000151726	ACSL1	4	185676749	185747972	0.0076655	18.675	8.33E-09	0.0016963	0.01203	5.595	0.0038442	rs17609240	4.58E-08	0.00063158	1.53E-144	0	Esophagus_Gastroesophageal_Junction
ENSG00000167914	GSDMA	17	38119226	38134019	ENSG00000151726	ACSL1	4	185676749	185747972	0.0076655	18.675	8.33E-09	0.0016963	0.01203	5.595	0.0038442	rs17609240	4.58E-08	0.00063158	1.53E-144	0	Esophagus_Muscularis
ENSG00000167914	GSDMA	17	38119226	38134019	ENSG00000151726	ACSL1	4	185676749	185747972	0.0076655	18.675	8.33E-09	0.0016963	0.01203	5.595	0.0038442	rs17609240	4.58E-08	0.00063158	1.53E-144	0	Heart_Left_Ventricle
ENSG00000167914	GSDMA	17	38119226	38134019	ENSG00000151726	ACSL1	4	185676749	185747972	0.0076655	18.675	8.33E-09	0.0016963	0.01203	5.595	0.0038442	rs17609240	4.58E-08	0.00063158	1.53E-144	0	Muscle_Skeletal
ENSG00000167914	GSDMA	17	38119226	38134019	ENSG00000151726	ACSL1	4	185676749	185747972	0.0076655	18.675	8.33E-09	0.0016963	0.01203	5.595	0.0038442	rs3894194	1.60E-06	0.0125406	6.08E-29	0	Adipose_Subcutaneous
ENSG00000167914	GSDMA	17	38119226	38134019	ENSG00000151726	ACSL1	4	185676749	185747972	0.0076655	18.675	8.33E-09	0.0016963	0.01203	5.595	0.0038442	rs3894194	1.60E-06	0.0125406	6.08E-29	0	Adipose_Visceral_Omentum
ENSG00000167914	GSDMA	17	38119226	38134019	ENSG00000151726	ACSL1	4	185676749	185747972	0.0076655	18.675	8.33E-09	0.0016963	0.01203	5.595	0.0038442	rs3894194	1.60E-06	0.0125406	6.08E-29	0	Artery_Tibial
ENSG00000167914	GSDMA	17	38119226	38134019	ENSG00000151726	ACSL1	4	185676749	185747972	0.0076655	18.675	8.33E-09	0.0016963	0.01203	5.595	0.0038442	rs3894194	1.60E-06	0.0125406	6.08E-29	0	Colon_Transverse
ENSG00000167914	GSDMA	17	38119226	38134019	ENSG00000151726	ACSL1	4	185676749	185747972	0.0076655	18.675	8.33E-09	0.0016963	0.01203	5.595	0.0038442	rs3894194	1.60E-06	0.0125406	6.08E-29	0	Esophagus_Gastroesophageal_Junction
ENSG00000167914	GSDMA	17	38119226	38134019	ENSG00000151726	ACSL1	4	185676749	185747972	0.0076655	18.675	8.33E-09	0.0016963	0.01203	5.595	0.0038442	rs3894194	1.60E-06	0.0125406	6.08E-29	0	Heart_Atrial_Appendage
ENSG00000167914	GSDMA	17	38119226	38134019	ENSG00000151726	ACSL1	4	185676749	185747972	0.0076655	18.675	8.33E-09	0.0016963	0.01203	5.595	0.0038442	rs3894194	1.60E-06	0.0125406	6.08E-29	0	Muscle_Skeletal
ENSG00000167914	GSDMA	17	38119226	38134019	ENSG00000151726	ACSL1	4	185676749	185747972	0.0076655	18.675	8.33E-09	0.0016963	0.01203	5.595	0.0038442	rs3894194	1.60E-06	0.0125406	6.08E-29	0	Whole_Blood
ENSG00000167914	GSDMA	17	38119226	38134019	ENSG00000151726	ACSL1	4	185676749	185747972	0.0076655	18.675	8.33E-09	0.0016963	0.01203	5.595	0.0038442	rs7212938	1.56E-07	0.00161211	5.34E-44	0	Adipose_Subcutaneous
ENSG00000167914	GSDMA	17	38119226	38134019	ENSG00000151726	ACSL1	4	185676749	185747972	0.0076655	18.675	8.33E-09	0.0016963	0.01203	5.595	0.0038442	rs7212938	1.56E-07	0.00161211	5.34E-44	0	Adipose_Visceral_Omentum
ENSG00000167914	GSDMA	17	38119226	38134019	ENSG00000151726	ACSL1	4	185676749	185747972	0.0076655	18.675	8.33E-09	0.0016963	0.01203	5.595	0.0038442	rs7212938	1.56E-07	0.00161211	5.34E-44	0	Artery_Coronary
ENSG00000167914	GSDMA	17	38119226	38134019	ENSG00000151726	ACSL1	4	185676749	185747972	0.0076655	18.675	8.33E-09	0.0016963	0.01203	5.595	0.0038442	rs7212938	1.56E-07	0.00161211	5.34E-44	0	Colon_Sigmoid
ENSG00000167914	GSDMA	17	38119226	38134019	ENSG00000151726	ACSL1	4	185676749	185747972	0.0076655	18.675	8.33E-09	0.0016963	0.01203	5.595	0.0038442	rs7212938	1.56E-07	0.00161211	5.34E-44	0	Heart_Atrial_Appendage
ENSG00000167914	GSDMA	17	38119226	38134019	ENSG00000151726	ACSL1	4	185676749	185747972	0.0076655	18.675	8.33E-09	0.0016963	0.01203	5.595	0.0038442	rs7212938	1.56E-07	0.00161211	5.34E-44	0	Heart_Left_Ventricle
ENSG00000167914	GSDMA	17	38119226	38134019	ENSG00000151726	ACSL1	4	185676749	185747972	0.0076655	18.675	8.33E-09	0.0016963	0.01203	5.595	0.0038442	rs7212938	1.56E-07	0.00161211	5.34E-44	0	Thyroid
ENSG00000167914	GSDMA	17	38119226	38134019	ENSG00000151726	ACSL1	4	185676749	185747972	0.0076655	18.675	8.33E-09	0.0016963	0.01203	5.595	0.0038442	rs7212938	1.56E-07	0.00161211	5.34E-44	0	Whole_Blood
ENSG00000167914	GSDMA	17	38119226	38134019	ENSG00000151726	ACSL1	4	185676749	185747972	0.0076655	18.675	8.33E-09	0.0016963	0.01203	5.595	0.0038442	rs8078723	1.33E-13	0	6.97E-07	0.002194627	Breast_Mammary_Tissue
ENSG00000167914	GSDMA	17	38119226	38134019	ENSG00000151726	ACSL1	4	185676749	185747972	0.0076655	18.675	8.33E-09	0.0016963	0.01203	5.595	0.0038442	rs8078723	1.33E-13	0	6.97E-07	0.002194627	Esophagus_Gastroesophageal_Junction
ENSG00000167914	GSDMA	17	38119226	38134019	ENSG00000151726	ACSL1	4	185676749	185747972	0.0076655	18.675	8.33E-09	0.0016963	0.01203	5.595	0.0038442	rs4794820	5.16E-07	0.00445765	2.26E-66	0	Artery_Tibial
ENSG00000167914	GSDMA	17	38119226	38134019	ENSG00000151726	ACSL1	4	185676749	185747972	0.0076655	18.675	8.33E-09	0.0016963	0.01203	5.595	0.0038442	rs4794820	5.16E-07	0.00445765	2.26E-66	0	Cells_Transformed_fibroblasts
ENSG00000167914	GSDMA	17	38119226	38134019	ENSG00000151726	ACSL1	4	185676749	185747972	0.0076655	18.675	8.33E-09	0.0016963	0.01203	5.595	0.0038442	rs4794822	1.03E-12	0	3.02E-06	0.008756123	Breast_Mammary_Tissue
ENSG00000167914	GSDMA	17	38119226	38134019	ENSG00000151726	ACSL1	4	185676749	185747972	0.0076655	18.675	8.33E-09	0.0016963	0.01203	5.595	0.0038442	rs4794822	1.03E-12	0	3.02E-06	0.008756123	Cells_Transformed_fibroblasts
ENSG00000167914	GSDMA	17	38119226	38134019	ENSG00000151726	ACSL1	4	185676749	185747972	0.0076655	18.675	8.33E-09	0.0016963	0.01203	5.595	0.0038442	rs4794822	1.03E-12	0	3.02E-06	0.008756123	Esophagus_Gastroesophageal_Junction
ENSG00000167914	GSDMA	17	38119226	38134019	ENSG00000151726	ACSL1	4	185676749	185747972	0.0076655	18.675	8.33E-09	0.0016963	0.01203	5.595	0.0038442	rs4794822	1.03E-12	0	3.02E-06	0.008756123	Esophagus_Muscularis
ENSG00000167914	GSDMA	17	38119226	38134019	ENSG00000151726	ACSL1	4	185676749	185747972	0.0076655	18.675	8.33E-09	0.0016963	0.01203	5.595	0.0038442	rs4065321	2.50E-12	0	2.52E-12	0	Pancreas
ENSG00000172660	TAF15	17	34136459	34174246	ENSG00000130159	ECSIT	19	11618272	11639989	0.010032	8.1597	8.44E-09	0.00171365	0.020708	3.2247	0.0038571	rs9915021	9.36E-14	0	NA	NA	Skin_Not_Sun_Exposed_Suprapubic
ENSG00000177951	BET1L	11	167784	207428	ENSG00000102362	SYTL4	X	99929488	99987110	0.01101	6.7201	8.64E-09	0.00175015	0.01185	0.99203	0.4516	rs505404	4.66E-06	0.03095987	3.73E-187	0	Small_Intestine_Terminal_Ileum
ENSG00000177951	BET1L	11	167784	207428	ENSG00000102362	SYTL4	X	99929488	99987110	0.01101	6.7201	8.64E-09	0.00175015	0.01185	0.99203	0.4516	rs505404	4.66E-06	0.03095987	3.73E-187	0	Whole_Blood
ENSG00000154760	SLFN13	17	33762115	33775856	ENSG00000102145	GATA1	X	48644962	48652718	0.01237	5.495	8.79E-09	0.00177063	0.028591	2.2295	0.0090774	rs225245	2.47E-25	0	NA	NA	Liver
ENSG00000154760	SLFN13	17	33762115	33775856	ENSG00000102145	GATA1	X	48644962	48652718	0.01237	5.495	8.79E-09	0.00177063	0.028591	2.2295	0.0090774	rs8082605	5.92E-08	0.00080711	2.08E-19	0	Artery_Tibial
ENSG00000154760	SLFN13	17	33762115	33775856	ENSG00000102145	GATA1	X	48644962	48652718	0.01237	5.495	8.79E-09	0.00177063	0.028591	2.2295	0.0090774	rs8082605	5.92E-08	0.00080711	2.08E-19	0	Esophagus_Mucosa
ENSG00000154760	SLFN13	17	33762115	33775856	ENSG00000102145	GATA1	X	48644962	48652718	0.01237	5.495	8.79E-09	0.00177063	0.028591	2.2295	0.0090774	rs8082605	5.92E-08	0.00080711	2.08E-19	0	Nerve_Tibial
ENSG00000154760	SLFN13	17	33762115	33775856	ENSG00000102145	GATA1	X	48644962	48652718	0.01237	5.495	8.79E-09	0.00177063	0.028591	2.2295	0.0090774	rs8082605	5.92E-08	0.00080711	2.08E-19	0	Pituitary
ENSG00000154760	SLFN13	17	33762115	33775856	ENSG00000102145	GATA1	X	48644962	48652718	0.01237	5.495	8.79E-09	0.00177063	0.028591	2.2295	0.0090774	rs10512472	1.87E-11	0	6.34E-38	0	Uterus
ENSG00000141736	ERBB2	17	37844949	37884915	ENSG00000134755	DSC2	18	28645940	28682378	0.010518	7.3345	8.79E-09	0.00177063	0.0036676	0.37302	0.94811	rs3859192	2.56E-13	0	NA	NA	Thyroid
ENSG00000141736	ERBB2	17	37844949	37884915	ENSG00000134755	DSC2	18	28645940	28682378	0.010518	7.3345	8.79E-09	0.00177063	0.0036676	0.37302	0.94811	rs3894194	3.07E-08	0.00039456	NA	NA	Whole_Blood
ENSG00000154760	SLFN13	17	33762115	33775856	ENSG00000068971	PPP2R5B	11	64685025	64700718	0.012358	5.4896	9.01E-09	0.00180855	0.010373	0.79399	0.65709	rs225245	2.45E-07	0.00237651	NA	NA	Liver
ENSG00000099849	RASSF7	11	560404	564021	ENSG00000151693	ASAP2	2	9346894	9541525	0.010504	7.325	9.06E-09	0.00181646	0.0044269	0.5806	0.77211	rs505404	3.11E-08	0.0004087	NA	NA	Brain_Frontal_Cortex_BA9
ENSG00000142102	ATHL1	11	289138	296107	ENSG00000151693	ASAP2	2	9346894	9541525	0.015612	4.0216	9.20E-09	0.00184196	0.00066157	0.30419	0.73779	rs17655730	6.61E-07	0.00575008	1.10E-69	0	Muscle_Skeletal
ENSG00000142102	ATHL1	11	289138	296107	ENSG00000151693	ASAP2	2	9346894	9541525	0.015612	4.0216	9.20E-09	0.00184196	0.00066157	0.30419	0.73779	rs505404	3.11E-08	0.0004087	3.83E-63	0	Lung
ENSG00000177666	PNPLA2	11	818902	825573	ENSG00000049323	LTBP1	2	33172039	33624576	0.0094573	9.2268	9.39E-09	0.00187351	0.021009	3.2725	0.0034394	rs11602954	4.93E-09	0.00012117	NA	NA	Brain_Frontal_Cortex_BA9
ENSG00000177666	PNPLA2	11	818902	825573	ENSG00000049323	LTBP1	2	33172039	33624576	0.0094573	9.2268	9.39E-09	0.00187351	0.021009	3.2725	0.0034394	rs17655730	2.15E-13	0	NA	NA	Brain_Frontal_Cortex_BA9
ENSG00000177666	PNPLA2	11	818902	825573	ENSG00000049323	LTBP1	2	33172039	33624576	0.0094573	9.2268	9.39E-09	0.00187351	0.021009	3.2725	0.0034394	rs17655730	2.15E-13	0	NA	NA	Esophagus_Muscularis
ENSG00000154760	SLFN13	17	33762115	33775856	ENSG00000011105	TSPAN9	12	3186521	3395730	0.012332	5.4779	9.51E-09	0.00189565	0.022762	1.7644	0.049727	rs225245	2.63E-36	0	NA	NA	Liver
ENSG00000154760	SLFN13	17	33762115	33775856	ENSG00000011105	TSPAN9	12	3186521	3395730	0.012332	5.4779	9.51E-09	0.00189565	0.022762	1.7644	0.049727	rs8082605	5.88E-65	0	2.08E-19	0	Artery_Tibial
ENSG00000154760	SLFN13	17	33762115	33775856	ENSG00000011105	TSPAN9	12	3186521	3395730	0.012332	5.4779	9.51E-09	0.00189565	0.022762	1.7644	0.049727	rs8082605	5.88E-65	0	2.08E-19	0	Esophagus_Mucosa
ENSG00000154760	SLFN13	17	33762115	33775856	ENSG00000011105	TSPAN9	12	3186521	3395730	0.012332	5.4779	9.51E-09	0.00189565	0.022762	1.7644	0.049727	rs8082605	5.88E-65	0	2.08E-19	0	Nerve_Tibial
ENSG00000154760	SLFN13	17	33762115	33775856	ENSG00000011105	TSPAN9	12	3186521	3395730	0.012332	5.4779	9.51E-09	0.00189565	0.022762	1.7644	0.049727	rs8082605	5.88E-65	0	2.08E-19	0	Pituitary
ENSG00000154760	SLFN13	17	33762115	33775856	ENSG00000011105	TSPAN9	12	3186521	3395730	0.012332	5.4779	9.51E-09	0.00189565	0.022762	1.7644	0.049727	rs10512472	5.91E-134	0	6.34E-38	0	Uterus
ENSG00000177963	RIC8A	11	207511	215113	ENSG00000174175	SELP	1	169558087	169599431	0.0094504	9.22	9.54E-09	0.00189565	0.010383	1.1974	0.29721	rs11602954	2.72E-34	0	7.55E-62	0	Adipose_Subcutaneous
ENSG00000177963	RIC8A	11	207511	215113	ENSG00000174175	SELP	1	169558087	169599431	0.0094504	9.22	9.54E-09	0.00189565	0.010383	1.1974	0.29721	rs11602954	2.72E-34	0	7.55E-62	0	Artery_Aorta
ENSG00000177963	RIC8A	11	207511	215113	ENSG00000174175	SELP	1	169558087	169599431	0.0094504	9.22	9.54E-09	0.00189565	0.010383	1.1974	0.29721	rs11602954	2.72E-34	0	7.55E-62	0	Artery_Tibial
ENSG00000177963	RIC8A	11	207511	215113	ENSG00000174175	SELP	1	169558087	169599431	0.0094504	9.22	9.54E-09	0.00189565	0.010383	1.1974	0.29721	rs11602954	2.72E-34	0	7.55E-62	0	Brain_Caudate_basal_ganglia
ENSG00000177963	RIC8A	11	207511	215113	ENSG00000174175	SELP	1	169558087	169599431	0.0094504	9.22	9.54E-09	0.00189565	0.010383	1.1974	0.29721	rs11602954	2.72E-34	0	7.55E-62	0	Brain_Frontal_Cortex_BA9
ENSG00000177963	RIC8A	11	207511	215113	ENSG00000174175	SELP	1	169558087	169599431	0.0094504	9.22	9.54E-09	0.00189565	0.010383	1.1974	0.29721	rs11602954	2.72E-34	0	7.55E-62	0	Colon_Sigmoid
ENSG00000177963	RIC8A	11	207511	215113	ENSG00000174175	SELP	1	169558087	169599431	0.0094504	9.22	9.54E-09	0.00189565	0.010383	1.1974	0.29721	rs11602954	2.72E-34	0	7.55E-62	0	Colon_Transverse
ENSG00000177963	RIC8A	11	207511	215113	ENSG00000174175	SELP	1	169558087	169599431	0.0094504	9.22	9.54E-09	0.00189565	0.010383	1.1974	0.29721	rs11602954	2.72E-34	0	7.55E-62	0	Esophagus_Gastroesophageal_Junction
ENSG00000177963	RIC8A	11	207511	215113	ENSG00000174175	SELP	1	169558087	169599431	0.0094504	9.22	9.54E-09	0.00189565	0.010383	1.1974	0.29721	rs11602954	2.72E-34	0	7.55E-62	0	Esophagus_Mucosa
ENSG00000177963	RIC8A	11	207511	215113	ENSG00000174175	SELP	1	169558087	169599431	0.0094504	9.22	9.54E-09	0.00189565	0.010383	1.1974	0.29721	rs11602954	2.72E-34	0	7.55E-62	0	Esophagus_Muscularis
ENSG00000177963	RIC8A	11	207511	215113	ENSG00000174175	SELP	1	169558087	169599431	0.0094504	9.22	9.54E-09	0.00189565	0.010383	1.1974	0.29721	rs11602954	2.72E-34	0	7.55E-62	0	Lung
ENSG00000177963	RIC8A	11	207511	215113	ENSG00000174175	SELP	1	169558087	169599431	0.0094504	9.22	9.54E-09	0.00189565	0.010383	1.1974	0.29721	rs11602954	2.72E-34	0	7.55E-62	0	Muscle_Skeletal
ENSG00000177963	RIC8A	11	207511	215113	ENSG00000174175	SELP	1	169558087	169599431	0.0094504	9.22	9.54E-09	0.00189565	0.010383	1.1974	0.29721	rs11602954	2.72E-34	0	7.55E-62	0	Nerve_Tibial
ENSG00000177963	RIC8A	11	207511	215113	ENSG00000174175	SELP	1	169558087	169599431	0.0094504	9.22	9.54E-09	0.00189565	0.010383	1.1974	0.29721	rs11602954	2.72E-34	0	7.55E-62	0	Skin_Not_Sun_Exposed_Suprapubic

ENSG00000177963	RIC8A	11	207511	215113	ENSG00000174175	SELP	1	169558087	169599431	0.0094504	9.22	9.54E-09	0.00189565	0.010383	1.1974	0.29721	rs11602954	2.72E-34	0	7.55E-62	0	Skin_Sun_Exposed_Lower_leg
ENSG00000177963	RIC8A	11	207511	215113	ENSG00000174175	SELP	1	169558087	169599431	0.0094504	9.22	9.54E-09	0.00189565	0.010383	1.1974	0.29721	rs11602954	2.72E-34	0	7.55E-62	0	Thyroid
ENSG00000177963	RIC8A	11	207511	215113	ENSG00000174175	SELP	1	169558087	169599431	0.0094504	9.22	9.54E-09	0.00189565	0.010383	1.1974	0.29721	rs17655730	4.69E-37	0	5.10E-36	0	Adipose_Subcutaneous
ENSG00000177963	RIC8A	11	207511	215113	ENSG00000174175	SELP	1	169558087	169599431	0.0094504	9.22	9.54E-09	0.00189565	0.010383	1.1974	0.29721	rs17655730	4.69E-37	0	5.10E-36	0	Esophagus_Mucosa
ENSG00000177963	RIC8A	11	207511	215113	ENSG00000174175	SELP	1	169558087	169599431	0.0094504	9.22	9.54E-09	0.00189565	0.010383	1.1974	0.29721	rs17655730	4.69E-37	0	5.10E-36	0	Esophagus_Muscularis
ENSG00000177963	RIC8A	11	207511	215113	ENSG00000174175	SELP	1	169558087	169599431	0.0094504	9.22	9.54E-09	0.00189565	0.010383	1.1974	0.29721	rs17655730	4.69E-37	0	5.10E-36	0	Heart_Left_Ventricle
ENSG00000177963	RIC8A	11	207511	215113	ENSG00000174175	SELP	1	169558087	169599431	0.0094504	9.22	9.54E-09	0.00189565	0.010383	1.1974	0.29721	rs17655730	4.69E-37	0	5.10E-36	0	Ovary
ENSG00000177963	RIC8A	11	207511	215113	ENSG00000174175	SELP	1	169558087	169599431	0.0094504	9.22	9.54E-09	0.00189565	0.010383	1.1974	0.29721	rs17655730	4.69E-37	0	5.10E-36	0	Pancreas
ENSG00000177963	RIC8A	11	207511	215113	ENSG00000174175	SELP	1	169558087	169599431	0.0094504	9.22	9.54E-09	0.00189565	0.010383	1.1974	0.29721	rs17655730	4.69E-37	0	5.10E-36	0	Skin_Not_Sun_Exposed_Suprapubic
ENSG00000177963	RIC8A	11	207511	215113	ENSG00000174175	SELP	1	169558087	169599431	0.0094504	9.22	9.54E-09	0.00189565	0.010383	1.1974	0.29721	rs505404	2.87E-40	0	3.66E-47	0	Artery_Aorta
ENSG00000177963	RIC8A	11	207511	215113	ENSG00000174175	SELP	1	169558087	169599431	0.0094504	9.22	9.54E-09	0.00189565	0.010383	1.1974	0.29721	rs505404	2.87E-40	0	3.66E-47	0	Thyroid
ENSG00000008838	MED24	17	38175350	38217468	ENSG00000163421	PROK2	3	71820807	71834357	0.0088793	10.825	9.80E-09	0.00193833	0.011764	2.729	0.028142	rs3859192	5.81E-16	0	1.47E-40	0	Whole_Blood
ENSG00000008838	MED24	17	38175350	38217468	ENSG00000163421	PROK2	3	71820807	71834357	0.0088793	10.825	9.80E-09	0.00193833	0.011764	2.729	0.028142	rs3894194	1.54E-06	0.01202721	8.18E-15	0	Thyroid
ENSG00000008838	MED24	17	38175350	38217468	ENSG00000163421	PROK2	3	71820807	71834357	0.0088793	10.825	9.80E-09	0.00193833	0.011764	2.729	0.028142	rs8078723	2.41E-14	0	5.30E-78	0	Adipose_Subcutaneous
ENSG00000008838	MED24	17	38175350	38217468	ENSG00000163421	PROK2	3	71820807	71834357	0.0088793	10.825	9.80E-09	0.00193833	0.011764	2.729	0.028142	rs8078723	2.41E-14	0	5.30E-78	0	Artery_Tibial
ENSG00000008838	MED24	17	38175350	38217468	ENSG00000163421	PROK2	3	71820807	71834357	0.0088793	10.825	9.80E-09	0.00193833	0.011764	2.729	0.028142	rs8078723	2.41E-14	0	5.30E-78	0	Cells_Transformed_fibroblasts
ENSG00000008838	MED24	17	38175350	38217468	ENSG00000163421	PROK2	3	71820807	71834357	0.0088793	10.825	9.80E-09	0.00193833	0.011764	2.729	0.028142	rs8078723	2.41E-14	0	5.30E-78	0	Lung
ENSG00000008838	MED24	17	38175350	38217468	ENSG00000163421	PROK2	3	71820807	71834357	0.0088793	10.825	9.80E-09	0.00193833	0.011764	2.729	0.028142	rs8078723	2.41E-14	0	5.30E-78	0	Pituitary
ENSG00000008838	MED24	17	38175350	38217468	ENSG00000163421	PROK2	3	71820807	71834357	0.0088793	10.825	9.80E-09	0.00193833	0.011764	2.729	0.028142	rs8078723	2.41E-14	0	5.30E-78	0	Skin_Sun_Exposed_Lower_leg
ENSG00000008838	MED24	17	38175350	38217468	ENSG00000163421	PROK2	3	71820807	71834357	0.0088793	10.825	9.80E-09	0.00193833	0.011764	2.729	0.028142	rs8078723	2.41E-14	0	5.30E-78	0	Spleen
ENSG00000008838	MED24	17	38175350	38217468	ENSG00000163421	PROK2	3	71820807	71834357	0.0088793	10.825	9.80E-09	0.00193833	0.011764	2.729	0.028142	rs8078723	2.41E-14	0	5.30E-78	0	Stomach
ENSG00000008838	MED24	17	38175350	38217468	ENSG00000163421	PROK2	3	71820807	71834357	0.0088793	10.825	9.80E-09	0.00193833	0.011764	2.729	0.028142	rs8078723	2.41E-14	0	5.30E-78	0	Thyroid
ENSG00000008838	MED24	17	38175350	38217468	ENSG00000163421	PROK2	3	71820807	71834357	0.0088793	10.825	9.80E-09	0.00193833	0.011764	2.729	0.028142	rs4794822	5.82E-14	0	1.32E-79	0	Esophagus_Gastroesophageal_Junction
ENSG00000008838	MED24	17	38175350	38217468	ENSG00000163421	PROK2	3	71820807	71834357	0.0088793	10.825	9.80E-09	0.00193833	0.011764	2.729	0.028142	rs4794822	5.82E-14	0	1.32E-79	0	Lung
ENSG00000008838	MED24	17	38175350	38217468	ENSG00000163421	PROK2	3	71820807	71834357	0.0088793	10.825	9.80E-09	0.00193833	0.011764	2.729	0.028142	rs4794822	5.82E-14	0	1.32E-79	0	Spleen
ENSG00000008838	MED24	17	38175350	38217468	ENSG00000163421	PROK2	3	71820807	71834357	0.0088793	10.825	9.80E-09	0.00193833	0.011764	2.729	0.028142	rs4794822	5.82E-14	0	1.32E-79	0	Whole_Blood
ENSG00000008838	MED24	17	38175350	38217468	ENSG00000163421	PROK2	3	71820807	71834357	0.0088793	10.825	9.80E-09	0.00193833	0.011764	2.729	0.028142	rs4065321	6.39E-13	0	1.24E-42	0	Pituitary
ENSG00000132139	GAS2L2	17	34071530	34079897	ENSG00000103148	NPRL3	16	138697	188859	0.010951	6.6835	9.83E-09	0.00194328	0.010462	1.2066	0.29166	rs9915021	1.32E-25	0	NA	NA	Small_Intestine_Terminal_Ileum
ENSG00000092871	RFFL	17	33341759	33416338	ENSG00000130300	PLVAP	19	17462257	17488159	0.0099455	8.0882	1.03E-08	0.00202146	0.0027958	0.31996	0.95866	rs9915021	2.45E-12	0	NA	NA	Adipose_Subcutaneous
ENSG00000092871	RFFL	17	33341759	33416338	ENSG00000130300	PLVAP	19	17462257	17488159	0.0099455	8.0882	1.03E-08	0.00202146	0.0027958	0.31996	0.95866	rs9915021	2.45E-12	0	NA	NA	Artery_Tibial
ENSG00000154760	SLFN13	17	33762115	33775856	ENSG00000141084	RANBP10	16	67757005	67840555	0.012294	5.4607	1.03E-08	0.00202897	0.017141	1.3211	0.20046	rs225245	6.94E-17	0	NA	NA	Liver
ENSG00000154760	SLFN13	17	33762115	33775856	ENSG00000141084	RANBP10	16	67757005	67840555	0.012294	5.4607	1.03E-08	0.00202897	0.017141	1.3211	0.20046	rs8082605	4.35E-13	0	2.08E-19	0	Artery_Tibial
ENSG00000154760	SLFN13	17	33762115	33775856	ENSG00000141084	RANBP10	16	67757005	67840555	0.012294	5.4607	1.03E-08	0.00202897	0.017141	1.3211	0.20046	rs8082605	4.35E-13	0	2.08E-19	0	Esophagus_Mucosa
ENSG00000154760	SLFN13	17	33762115	33775856	ENSG00000141084	RANBP10	16	67757005	67840555	0.012294	5.4607	1.03E-08	0.00202897	0.017141	1.3211	0.20046	rs8082605	4.35E-13	0	2.08E-19	0	Nerve_Tibial
ENSG00000154760	SLFN13	17	33762115	33775856	ENSG00000141084	RANBP10	16	67757005	67840555	0.012294	5.4607	1.03E-08	0.00202897	0.017141	1.3211	0.20046	rs8082605	4.35E-13	0	2.08E-19	0	Pituitary
ENSG00000154760	SLFN13	17	33762115	33775856	ENSG00000141084	RANBP10	16	67757005	67840555	0.012294	5.4607	1.03E-08	0.00202897	0.017141	1.3211	0.20046	rs10512472	1.25E-08	0.00017572	6.34E-38	0	Uterus
ENSG00000188076	SCGB1C1	11	193080	194573	ENSG00000138722	MMRN1	4	90800683	90875780	0.0067515	32.872	1.04E-08	0.00205318	0.0057822	5.3506	0.020935	rs11602954	5.94E-65	0	3.14E-203	0	Whole_Blood
ENSG00000188076	SCGB1C1	11	193080	194573	ENSG00000138722	MMRN1	4	90800683	90875780	0.0067515	32.872	1.04E-08	0.00205318	0.0057822	5.3506	0.020935	rs17655730	6.29E-64	0	1.60E-163	0	Whole_Blood
ENSG00000188076	SCGB1C1	11	193080	194573	ENSG00000138722	MMRN1	4	90800683	90875780	0.0067515	32.872	1.04E-08	0.00205318	0.0057822	5.3506	0.020935	rs505404	2.01E-74	0	5.81E-188	0	Whole_Blood
ENSG00000141150	RASL10B	17	34058668	34070540	ENSG00000017483	SLC38A5	X	48316920	48328644	0.010923	6.6661	1.05E-08	0.00205416	0.025397	3.4025	0.0013519	rs9915021	6.46E-06	0.04076003	NA	NA	Cells_Transformed_fibroblasts
ENSG00000132139	GAS2L2	17	34071530	34079897	ENSG00000160445	ZER1	9	131492065	131534693	0.010914	6.6607	1.07E-08	0.00208984	0.0029908	0.34235	0.9494	rs9915021	1.28E-09	5.83E-05	NA	NA	Small_Intestine_Terminal_Ileum
ENSG00000006125	AP2B1	17	33913918	34053436	ENSG00000130227	XPO7	8	21777180	21864096	0.010423	7.268	1.08E-08	0.00212272	0.0057807	0.88669	0.50393	rs10512472	5.74E-06	0.03701626	3.62E-14	0	Heart_Left_Ventricle
ENSG00000006125	AP2B1	17	33913918	34053436	ENSG00000133816	MICAL2	11	12115543	12285332	0.010422	7.2672	1.09E-08	0.00212376	0.010238	1.5775	0.15047	rs10512472	2.85E-24	0	3.62E-14	0	Heart_Left_Ventricle
ENSG00000006125	AP2B1	17	33913918	34053436	ENSG00000133808	MICALCL	11	12297627	12380691	0.010422	7.2672	1.09E-08	0.00212376	0.0078117	1.2007	0.3035	rs10512472	1.86E-09	5.64E-05	3.62E-14	0	Heart_Left_Ventricle
ENSG00000177963	RIC8A	11	207511	215113	ENSG00000173210	ABLIM3	5	148521046	148640105	0.0093801	9.1508	1.12E-08	0.00217571	0.019075	2.2193	0.024082	rs11602954	2.06E-35	0	7.55E-62	0	Adipose_Subcutaneous
ENSG00000177963	RIC8A	11	207511	215113	ENSG00000173210	ABLIM3	5	148521046	148640105	0.0093801	9.1508	1.12E-08	0.00217571	0.019075	2.2193	0.024082	rs11602954	2.06E-35	0	7.55E-62	0	Artery_Aorta
ENSG00000177963	RIC8A	11	207511	215113	ENSG00000173210	ABLIM3	5	148521046	148640105	0.0093801	9.1508	1.12E-08	0.00217571	0.019075	2.2193	0.024082	rs11602954	2.06E-35	0	7.55E-62	0	Artery_Tibial
ENSG00000177963	RIC8A	11	207511	215113	ENSG00000173210	ABLIM3	5	148521046	148640105	0.0093801	9.1508	1.12E-08	0.00217571	0.019075	2.2193	0.024082	rs11602954	2.06E-35	0	7.55E-62	0	Brain_Caudate_basal_ganglia
ENSG00000177963	RIC8A	11	207511	215113	ENSG00000173210	ABLIM3	5	148521046	148640105	0.0093801	9.1508	1.12E-08	0.00217571	0.019075	2.2193	0.024082	rs11602954	2.06E-35	0	7.55E-62	0	Brain_Frontal_Cortex_BA9
ENSG00000177963	RIC8A	11	207511	215113	ENSG00000173210	ABLIM3	5	148521046	148640105	0.0093801	9.1508	1.12E-08	0.00217571	0.019075	2.2193	0.024082	rs11602954	2.06E-35	0	7.55E-62	0	Colon_Sigmoid
ENSG00000177963	RIC8A	11	207511	215113	ENSG00000173210	ABLIM3	5	148521046	148640105	0.0093801	9.1508	1.12E-08	0.00217571	0.019075	2.2193	0.024082	rs11602954	2.06E-35	0	7.55E-62	0	Colon_Transverse
ENSG00000177963	RIC8A	11	207511	215113	ENSG00000173210	ABLIM3	5	148521046	148640105	0.0093801	9.1508	1.12E-08	0.00217571	0.019075	2.2193	0.024082	rs11602954	2.06E-35	0	7.55E-62	0	Esophagus_Gastroesophageal_Junction
ENSG00000177963	RIC8A	11	207511	215113	ENSG00000173210	ABLIM3	5	148521046	148640105	0.0093801	9.1508	1.12E-08	0.00217571	0.019075	2.2193	0.024082	rs11602954	2.06E-35	0	7.55E-62	0	Esophagus_Mucosa
ENSG00000177963	RIC8A	11	207511	215113	ENSG00000173210	ABLIM3	5	148521046	148640105	0.0093801	9.1508	1.12E-08	0.00217571	0.019075	2.2193	0.024082	rs11602954	2.06E-35	0	7.55E-62	0	Esophagus_Muscularis
ENSG00000177963	RIC8A	11	207511	215113	ENSG00000173210	ABLIM3	5	148521046	148640105	0.0093801	9.1508	1.12E-08	0.00217571	0.019075	2.2193	0.024082	rs11602954	2.06E-35	0	7.55E-62	0	Lung
ENSG00000177963	RIC8A	11	207511	215113	ENSG00000173210	ABLIM3	5	148521046	148640105	0.0093801	9.1508	1.12E-08	0.00217571	0.019075	2.2193	0.024082	rs11602954	2.06E-35	0	7.55E-62	0	Muscle_Skeletal
ENSG00000177963	RIC8A	11	207511	215113	ENSG00000173210	ABLIM3	5	148521046	148640105	0.0093801	9.1508	1.12E-08	0.00217571	0.019075	2.2193	0.024082	rs11602954	2.06E-35	0	7.55E-62	0	Nerve_Tibial
ENSG00000177963	RIC8A	11	207511	215113	ENSG00000173210	ABLIM3	5	148521046	148640105	0.0093801	9.1508	1.12E-08	0.00217571	0.019075	2.2193	0.024082	rs11602954	2.06E-35	0	7.55E-62	0	Skin_Not_Sun_Exposed_Suprapubic
ENSG00000177963	RIC8A	11	207511	215113	ENSG00000173210	ABLIM3	5	148521046	148640105	0.0093801	9.1508	1.12E-08	0.00217571	0.019075	2.2193	0.024082	rs11602954	2.06E-35	0	7.55E-62	0	Skin_Sun_Exposed_Lower_leg
ENSG00000177963	RIC8A	11	207511	215113	ENSG00000173210	ABLIM3	5	148521046	148640105	0.0093801	9.1508	1.12E-08	0.00217571	0.019075	2.2193	0.024082	rs11602954	2.06E-35	0	7.55E-62	0	Thyroid
ENSG00000177963	RIC8A	11	207511	215113	ENSG00000173210	ABLIM3	5	148521046	148640105	0.0093801	9.1508	1.12E-08	0.00217571	0.019075	2.2193	0.024082	rs17655730	1.22E-33	0	5.10E-36	0	Adipose_Subcutaneous
ENSG00000177963	RIC8A	11	207511	215113	ENSG00000173210	ABLIM3	5	148521046	148640105	0.0093801	9.1508	1.12E-08	0.00217571	0.019075	2.2193	0.024082	rs17655730	1.22E-33	0	5.10E-36	0	Esophagus_Mucosa
ENSG00000177963	RIC8A	11	207511	215113	ENSG00000173210	ABLIM3	5	148521046	148640105	0.0093801	9.1508	1.12E-08	0.00217571	0.019075	2.2193	0.024082	rs17655730	1.22E-33	0	5.10E-36	0	Esophagus_Muscularis
ENSG00000177963	RIC8A	11	207511	215113	ENSG00000173210	ABLIM3	5	148521046	148640105	0.0093801	9.1508	1.12E-08	0.00217571	0.019075	2.2193	0.024082	rs17655730	1.22E-33	0	5.10E-36	0	Heart_Left_Ventricle
ENSG00000177963	RIC8A	11	207511	215113	ENSG00000173210	ABLIM3	5	148521046	148640105	0.0093801	9.1508	1.12E-08	0.00217571	0.019075	2.2193	0.024082	rs17655730	1.22E-33	0	5.10E-36	0	Ovary
ENSG00000177963	RIC8A	11	207511	215113	ENSG00000173210	ABLIM3	5	148521046	148640105	0.0093801	9.1508	1.12E-08	0.00217571	0.019075	2.2193	0.024082	rs17655730	1.22E-33	0	5.10E-36	0	Pancreas
ENSG00000177963	RIC8A	11	207511	215113	ENSG00000173210	ABLIM3	5	148521046	148640105	0.0093801	9.1508	1.12E-08	0.00217571	0.019075	2.2193	0.024082	rs17655730	1.22E-33	0	5.10E-36	0	Skin_Not_Sun_Exposed_Suprapubic
ENSG00000177963	RIC8A	11	207511	215113	ENSG00000173210	ABLIM3	5	148521046	148640105	0.0093801	9.1508	1.12E-08	0.00217571	0.019075	2.2193	0.024082	rs505404	3.10E-39	0	3.66E-47	0	Artery_Aorta
ENSG00000177963	RIC8A	11	207511	215113	ENSG00000173210	ABLIM3	5	148521046	148640105	0.0093801	9.1508	1.12E-08	0.00217571	0.019075	2.2193	0.024082	rs505404	3.10E-39	0	3.66E-47	0	Thyroid
ENSG00000141736	ERBB2	17	37844949	37884915	ENSG00000146592	CREB5	7	28338940	28865511	0.010393	7.2466	1.16E-08	0.00223778	0.0084757	0.86621	0.55519	rs3859192	1.81E-15	0	NA	NA	Thyroid
ENSG00000141736	ERBB2	17	37844949	37884915	ENSG00000146592	CREB5	7	28338940	28865511	0.010393	7.2466	1.16E-08	0.00223778	0.0084757	0.86621	0.55519	rs3894194	4.69E-11	0	NA	NA	Whole_Blood
ENSG00000141736	ERBB2	17	37844949	37884915	ENSG00000146592	CREB5	7	28338940	28865511	0.010393	7.2466	1.16E-08	0.00223778	0.0084757	0.86621	0.55519	rs9303280	8.57E-07	0.00721415	1.32E-09	1.31E-05	Testis
ENSG00000141736	ERBB2	17	37844949	37884915	ENSG00000146592	CREB5	7	28338940	28865511	0.010393	7.2466	1.16E-08	0.00223778	0.0084757	0.86621	0.55519	rs8069176	5.54E-07	0.00490114	5.55E-13	0	Heart_Atrial_Appendage
ENSG00000177666	PNPLA2	11	818902	825573	ENSG00000151693	ASAP2	2	9346894	9541525	0.0093561	9.1272	1.18E-08	0.00228099	0.0039207	0.60026	0.73031	rs17655730	6.61E-07	0.00575008	NA	NA	Brain_Frontal_Cortex_BA9
ENSG00000177666	PNPLA2	11	818902	825573	ENSG00000151693	ASAP2	2	9346894	9541525	0.0093561	9.1272	1.18E-08	0.00228099	0.0039207	0.60026	0.73031	rs17655730	6.61E-07	0.00575008	NA	NA	Esophagus_Muscularis
ENSG00000177951	BET1L	11	167784	207428	ENSG00000173210	ABLIM3	5	148521046	148640105	0.010849	6.6205	1.23E-08	0.0023609	0.027892	2.3736	0.0067874	rs11602954	2.06E-35	0	9.12E-206	0	Adipose_Visceral_Omentum
ENSG00000177951	BET1L	11	167784	207428	ENSG00000173210	ABLIM3	5	148521046	148640105	0.010849	6.6205	1.23E-08	0.0023609	0.027892	2.3736	0.0067874	rs11602954	2.06E-35	0	9.12E-206	0	Artery_Aorta
ENSG00000177951	BET1L	11	167784	207428	ENSG00000173210	ABLIM3	5	148521046	148640105	0.010849	6.6205	1.23E-08	0.0023609	0.027892	2.3736	0.0067874	rs11602954	2.06E-35	0	9.12E-206	0	Cells_Transformed_fibroblasts
ENSG00000177951	BET1L	11	167784	207428	ENSG00000173210	ABLIM3	5	148521046	148640105	0.010849	6.6205	1.23E-08	0.0023609	0.027892	2.3736	0.0067874	rs11602954	2.06E-35	0	9.12E-206	0	Pituitary
ENSG00000177951	BET1L	11	167784	207428	ENSG00000173210	ABLIM3	5	148521046	148640105	0.010849	6.6205	1.23E-08	0.0023609	0.027892	2.3736	0.0067874	rs11602954	2.06E-35	0	9.12E-206	0	Prostate
ENSG00000177951	BET1L	11	167784	207428	ENSG00000173210	ABLIM3	5	148521046	148640105	0.010849	6.6205	1.23E-08	0.0023609	0.027892	2.3736	0.0067874	rs11602954	2.06E-35	0	9.12E-206	0	Small_Intestine_Terminal_Ileum
ENSG00000177951	BET1L	11	167784	207428	ENSG00000173210	ABLIM3	5	148521046	148640105	0.010849	6.6205	1.23E-08	0.0023609	0.027892	2.3736	0.0067874	rs11602954	2.06E-35	0	9.12E-206	0	Whole_Blood

ENSG00000177951	BET1L	11	167784	207428	ENSG00000173210	ABLIM3	5	148521046	148640105	0.010849	6.6205	1.23E-08	0.0023609	0.027892	2.3736	0.0067874	rs17655730	1.22E-33	0	2.96E-166	0	Prostate
ENSG00000177951	BET1L	11	167784	207428	ENSG00000173210	ABLIM3	5	148521046	148640105	0.010849	6.6205	1.23E-08	0.0023609	0.027892	2.3736	0.0067874	rs505404	3.10E-39	0	3.73E-187	0	Small_Intestine_Terminal_Ileum
ENSG00000177951	BET1L	11	167784	207428	ENSG00000173210	ABLIM3	5	148521046	148640105	0.010849	6.6205	1.23E-08	0.0023609	0.027892	2.3736	0.0067874	rs505404	3.10E-39	0	3.73E-187	0	Whole_Blood
ENSG00000108733	PEX12	17	33901814	33905882	ENSG00000128266	GNAZ	22	23412540	23464889	0.0093398	9.1111	1.23E-08	0.0023609	0.014074	2.6152	0.023334	rs9915021	4.37E-11	0	NA	NA	Brain_Caudate_basal_ganglia
ENSG00000108733	PEX12	17	33901814	33905882	ENSG00000128266	GNAZ	22	23412540	23464889	0.0093398	9.1111	1.23E-08	0.0023609	0.014074	2.6152	0.023334	rs9915021	4.37E-11	0	NA	NA	Breast_Mammary_Tissue
ENSG00000108733	PEX12	17	33901814	33905882	ENSG00000128266	GNAZ	22	23412540	23464889	0.0093398	9.1111	1.23E-08	0.0023609	0.014074	2.6152	0.023334	rs10512472	4.37E-21	0	1.06E-12	0	Brain_Caudate_basal_ganglia
ENSG00000006125	AP2B1	17	33913918	34053436	ENSG00000180354	C7orf41	7	30174426	30202378	0.010365	7.2271	1.23E-08	0.00236338	0.027393	4.295	0.00027945	rs10512472	6.35E-117	0	3.62E-14	0	Heart_Left_Ventricle
ENSG00000186075	ZPBP2	17	38024417	38034149	ENSG00000196663	TECPR2	14	102829300	102968818	0.0066843	32.543	1.24E-08	0.00236739	6.36E-05	0.058479	0.80897	rs10445308	9.54E-07	0.00782177	NA	NA	Testis
ENSG00000186075	ZPBP2	17	38024417	38034149	ENSG00000196663	TECPR2	14	102829300	102968818	0.0066843	32.543	1.24E-08	0.00236739	6.36E-05	0.058479	0.80897	rs3859192	4.25E-17	0	NA	NA	Testis
ENSG00000186075	ZPBP2	17	38024417	38034149	ENSG00000196663	TECPR2	14	102829300	102968818	0.0066843	32.543	1.24E-08	0.00236739	6.36E-05	0.058479	0.80897	rs4794820	8.93E-09	0.00016458	NA	NA	Testis
ENSG00000172123	SLFN12	17	33738079	33760302	ENSG00000184481	FOXO4	X	70316047	70323385	0.013878	4.5242	1.25E-08	0.00239127	0.0083713	1.2874	0.26023	rs225245	1.36E-06	0.01072981	NA	NA	Cells_EBV-transformed_lymphocytes
ENSG00000112619	PRPH2	6	42664340	42690312	ENSG00000182389	CACNB4	2	152689290	152955593	0.0074763	18.21	1.32E-08	0.00251237	0.0035458	1.6351	0.1955	rs16895831	3.45E-25	0	NA	NA	Uterus
ENSG00000177963	RIC8A	11	207511	215113	ENSG00000120885	CLU	8	27454434	27472548	0.0092919	9.0639	1.37E-08	0.00259773	0.016764	1.9458	0.050366	rs11602954	1.24E-10	0	7.55E-62	0	Adipose_Subcutaneous
ENSG00000177963	RIC8A	11	207511	215113	ENSG00000120885	CLU	8	27454434	27472548	0.0092919	9.0639	1.37E-08	0.00259773	0.016764	1.9458	0.050366	rs11602954	1.24E-10	0	7.55E-62	0	Artery_Aorta
ENSG00000177963	RIC8A	11	207511	215113	ENSG00000120885	CLU	8	27454434	27472548	0.0092919	9.0639	1.37E-08	0.00259773	0.016764	1.9458	0.050366	rs11602954	1.24E-10	0	7.55E-62	0	Artery_Tibial
ENSG00000177963	RIC8A	11	207511	215113	ENSG00000120885	CLU	8	27454434	27472548	0.0092919	9.0639	1.37E-08	0.00259773	0.016764	1.9458	0.050366	rs11602954	1.24E-10	0	7.55E-62	0	Brain_Caudate_basal_ganglia
ENSG00000177963	RIC8A	11	207511	215113	ENSG00000120885	CLU	8	27454434	27472548	0.0092919	9.0639	1.37E-08	0.00259773	0.016764	1.9458	0.050366	rs11602954	1.24E-10	0	7.55E-62	0	Brain_Frontal_Cortex_BA9
ENSG00000177963	RIC8A	11	207511	215113	ENSG00000120885	CLU	8	27454434	27472548	0.0092919	9.0639	1.37E-08	0.00259773	0.016764	1.9458	0.050366	rs11602954	1.24E-10	0	7.55E-62	0	Colon_Sigmoid
ENSG00000177963	RIC8A	11	207511	215113	ENSG00000120885	CLU	8	27454434	27472548	0.0092919	9.0639	1.37E-08	0.00259773	0.016764	1.9458	0.050366	rs11602954	1.24E-10	0	7.55E-62	0	Colon_Transverse
ENSG00000177963	RIC8A	11	207511	215113	ENSG00000120885	CLU	8	27454434	27472548	0.0092919	9.0639	1.37E-08	0.00259773	0.016764	1.9458	0.050366	rs11602954	1.24E-10	0	7.55E-62	0	Esophagus_Gastroesophageal_Junction
ENSG00000177963	RIC8A	11	207511	215113	ENSG00000120885	CLU	8	27454434	27472548	0.0092919	9.0639	1.37E-08	0.00259773	0.016764	1.9458	0.050366	rs11602954	1.24E-10	0	7.55E-62	0	Esophagus_Mucosa
ENSG00000177963	RIC8A	11	207511	215113	ENSG00000120885	CLU	8	27454434	27472548	0.0092919	9.0639	1.37E-08	0.00259773	0.016764	1.9458	0.050366	rs11602954	1.24E-10	0	7.55E-62	0	Esophagus_Muscularis
ENSG00000177963	RIC8A	11	207511	215113	ENSG00000120885	CLU	8	27454434	27472548	0.0092919	9.0639	1.37E-08	0.00259773	0.016764	1.9458	0.050366	rs11602954	1.24E-10	0	7.55E-62	0	Lung
ENSG00000177963	RIC8A	11	207511	215113	ENSG00000120885	CLU	8	27454434	27472548	0.0092919	9.0639	1.37E-08	0.00259773	0.016764	1.9458	0.050366	rs11602954	1.24E-10	0	7.55E-62	0	Muscle_Skeletal
ENSG00000177963	RIC8A	11	207511	215113	ENSG00000120885	CLU	8	27454434	27472548	0.0092919	9.0639	1.37E-08	0.00259773	0.016764	1.9458	0.050366	rs11602954	1.24E-10	0	7.55E-62	0	Nerve_Tibial
ENSG00000177963	RIC8A	11	207511	215113	ENSG00000120885	CLU	8	27454434	27472548	0.0092919	9.0639	1.37E-08	0.00259773	0.016764	1.9458	0.050366	rs11602954	1.24E-10	0	7.55E-62	0	Skin_Not_Sun_Exposed_Suprapubic
ENSG00000177963	RIC8A	11	207511	215113	ENSG00000120885	CLU	8	27454434	27472548	0.0092919	9.0639	1.37E-08	0.00259773	0.016764	1.9458	0.050366	rs11602954	1.24E-10	0	7.55E-62	0	Skin_Sun_Exposed_Lower_leg
ENSG00000177963	RIC8A	11	207511	215113	ENSG00000120885	CLU	8	27454434	27472548	0.0092919	9.0639	1.37E-08	0.00259773	0.016764	1.9458	0.050366	rs11602954	1.24E-10	0	7.55E-62	0	Thyroid
ENSG00000177963	RIC8A	11	207511	215113	ENSG00000120885	CLU	8	27454434	27472548	0.0092919	9.0639	1.37E-08	0.00259773	0.016764	1.9458	0.050366	rs17655730	2.73E-13	0	5.10E-36	0	Adipose_Subcutaneous
ENSG00000177963	RIC8A	11	207511	215113	ENSG00000120885	CLU	8	27454434	27472548	0.0092919	9.0639	1.37E-08	0.00259773	0.016764	1.9458	0.050366	rs17655730	2.73E-13	0	5.10E-36	0	Esophagus_Mucosa
ENSG00000177963	RIC8A	11	207511	215113	ENSG00000120885	CLU	8	27454434	27472548	0.0092919	9.0639	1.37E-08	0.00259773	0.016764	1.9458	0.050366	rs17655730	2.73E-13	0	5.10E-36	0	Esophagus_Muscularis
ENSG00000177963	RIC8A	11	207511	215113	ENSG00000120885	CLU	8	27454434	27472548	0.0092919	9.0639	1.37E-08	0.00259773	0.016764	1.9458	0.050366	rs17655730	2.73E-13	0	5.10E-36	0	Heart_Left_Ventricle
ENSG00000177963	RIC8A	11	207511	215113	ENSG00000120885	CLU	8	27454434	27472548	0.0092919	9.0639	1.37E-08	0.00259773	0.016764	1.9458	0.050366	rs17655730	2.73E-13	0	5.10E-36	0	Ovary
ENSG00000177963	RIC8A	11	207511	215113	ENSG00000120885	CLU	8	27454434	27472548	0.0092919	9.0639	1.37E-08	0.00259773	0.016764	1.9458	0.050366	rs17655730	2.73E-13	0	5.10E-36	0	Pancreas
ENSG00000177963	RIC8A	11	207511	215113	ENSG00000120885	CLU	8	27454434	27472548	0.0092919	9.0639	1.37E-08	0.00259773	0.016764	1.9458	0.050366	rs17655730	2.73E-13	0	5.10E-36	0	Skin_Not_Sun_Exposed_Suprapubic
ENSG00000177963	RIC8A	11	207511	215113	ENSG00000120885	CLU	8	27454434	27472548	0.0092919	9.0639	1.37E-08	0.00259773	0.016764	1.9458	0.050366	rs505404	7.47E-15	0	3.66E-47	0	Artery_Aorta
ENSG00000177963	RIC8A	11	207511	215113	ENSG00000120885	CLU	8	27454434	27472548	0.0092919	9.0639	1.37E-08	0.00259773	0.016764	1.9458	0.050366	rs505404	7.47E-15	0	3.66E-47	0	Thyroid
ENSG00000184886	PIGW	17	34892943	34895159	ENSG00000167671	UBXN6	19	4446046	4457819	0.0087334	10.645	1.38E-08	0.00260707	0.012148	2.2528	0.047301	rs9915021	2.43E-46	0	NA	NA	Muscle_Skeletal
ENSG00000172716	SLFN11	17	33677324	33700720	ENSG00000118046	STK11	19	1189406	1226662	0.012555	5.1122	1.47E-08	0.00277128	0.011226	0.86001	0.58806	rs225245	1.03E-13	0	5.77E-07	0.001884469	Cells_Transformed_fibroblasts
ENSG00000141150	RASL10B	17	34058668	34070540	ENSG00000198959	TGM2	20	36756863	36794980	0.010762	6.567	1.48E-08	0.00279331	0.0048603	0.63771	0.72489	rs9915021	2.01E-09	5.61E-05	NA	NA	Cells_Transformed_fibroblasts
ENSG00000006125	AP2B1	17	33913918	34053436	ENSG00000163554	SPTA1	1	158580278	158656488	0.010278	7.1656	1.49E-08	0.0028105	0.010001	1.5405	0.16174	rs10512472	1.40E-07	0.00152082	3.62E-14	0	Heart_Left_Ventricle
ENSG00000004534	RBM6	3	49977440	50114683	ENSG00000175691	ZNF77	19	2933216	2944969	0.0074225	18.078	1.51E-08	0.00282996	0.0015672	0.72126	0.48642	rs2013208	7.61E-06	0.04648663	3.2717e-310	0	Adipose_Subcutaneous
ENSG00000004534	RBM6	3	49977440	50114683	ENSG00000175691	ZNF77	19	2933216	2944969	0.0074225	18.078	1.51E-08	0.00282996	0.0015672	0.72126	0.48642	rs2013208	7.61E-06	0.04648663	3.2717e-310	0	Adipose_Visceral_Omentum
ENSG00000004534	RBM6	3	49977440	50114683	ENSG00000175691	ZNF77	19	2933216	2944969	0.0074225	18.078	1.51E-08	0.00282996	0.0015672	0.72126	0.48642	rs2013208	7.61E-06	0.04648663	3.2717e-310	0	Artery_Tibial
ENSG00000004534	RBM6	3	49977440	50114683	ENSG00000175691	ZNF77	19	2933216	2944969	0.0074225	18.078	1.51E-08	0.00282996	0.0015672	0.72126	0.48642	rs2013208	7.61E-06	0.04648663	3.2717e-310	0	Brain_Cortex
ENSG00000004534	RBM6	3	49977440	50114683	ENSG00000175691	ZNF77	19	2933216	2944969	0.0074225	18.078	1.51E-08	0.00282996	0.0015672	0.72126	0.48642	rs2013208	7.61E-06	0.04648663	3.2717e-310	0	Breast_Mammary_Tissue
ENSG00000004534	RBM6	3	49977440	50114683	ENSG00000175691	ZNF77	19	2933216	2944969	0.0074225	18.078	1.51E-08	0.00282996	0.0015672	0.72126	0.48642	rs2013208	7.61E-06	0.04648663	3.2717e-310	0	Cells_EBV-transformed_lymphocytes
ENSG00000004534	RBM6	3	49977440	50114683	ENSG00000175691	ZNF77	19	2933216	2944969	0.0074225	18.078	1.51E-08	0.00282996	0.0015672	0.72126	0.48642	rs2013208	7.61E-06	0.04648663	3.2717e-310	0	Esophagus_Gastroesophageal_Junction
ENSG00000004534	RBM6	3	49977440	50114683	ENSG00000175691	ZNF77	19	2933216	2944969	0.0074225	18.078	1.51E-08	0.00282996	0.0015672	0.72126	0.48642	rs2013208	7.61E-06	0.04648663	3.2717e-310	0	Esophagus_Mucosa
ENSG00000004534	RBM6	3	49977440	50114683	ENSG00000175691	ZNF77	19	2933216	2944969	0.0074225	18.078	1.51E-08	0.00282996	0.0015672	0.72126	0.48642	rs2013208	7.61E-06	0.04648663	3.2717e-310	0	Heart_Left_Ventricle
ENSG00000004534	RBM6	3	49977440	50114683	ENSG00000175691	ZNF77	19	2933216	2944969	0.0074225	18.078	1.51E-08	0.00282996	0.0015672	0.72126	0.48642	rs2013208	7.61E-06	0.04648663	3.2717e-310	0	Muscle_Skeletal
ENSG00000004534	RBM6	3	49977440	50114683	ENSG00000175691	ZNF77	19	2933216	2944969	0.0074225	18.078	1.51E-08	0.00282996	0.0015672	0.72126	0.48642	rs2013208	7.61E-06	0.04648663	3.2717e-310	0	Nerve_Tibial
ENSG00000004534	RBM6	3	49977440	50114683	ENSG00000175691	ZNF77	19	2933216	2944969	0.0074225	18.078	1.51E-08	0.00282996	0.0015672	0.72126	0.48642	rs2013208	7.61E-06	0.04648663	3.2717e-310	0	Ovary
ENSG00000004534	RBM6	3	49977440	50114683	ENSG00000175691	ZNF77	19	2933216	2944969	0.0074225	18.078	1.51E-08	0.00282996	0.0015672	0.72126	0.48642	rs2013208	7.61E-06	0.04648663	3.2717e-310	0	Pancreas
ENSG00000004534	RBM6	3	49977440	50114683	ENSG00000175691	ZNF77	19	2933216	2944969	0.0074225	18.078	1.51E-08	0.00282996	0.0015672	0.72126	0.48642	rs2013208	7.61E-06	0.04648663	3.2717e-310	0	Prostate
ENSG00000004534	RBM6	3	49977440	50114683	ENSG00000175691	ZNF77	19	2933216	2944969	0.0074225	18.078	1.51E-08	0.00282996	0.0015672	0.72126	0.48642	rs2013208	7.61E-06	0.04648663	3.2717e-310	0	Skin_Sun_Exposed_Lower_leg
ENSG00000004534	RBM6	3	49977440	50114683	ENSG00000175691	ZNF77	19	2933216	2944969	0.0074225	18.078	1.51E-08	0.00282996	0.0015672	0.72126	0.48642	rs2013208	7.61E-06	0.04648663	3.2717e-310	0	Small_Intestine_Terminal_Ileum
ENSG00000004534	RBM6	3	49977440	50114683	ENSG00000175691	ZNF77	19	2933216	2944969	0.0074225	18.078	1.51E-08	0.00282996	0.0015672	0.72126	0.48642	rs2013208	7.61E-06	0.04648663	3.2717e-310	0	Spleen
ENSG00000004534	RBM6	3	49977440	50114683	ENSG00000175691	ZNF77	19	2933216	2944969	0.0074225	18.078	1.51E-08	0.00282996	0.0015672	0.72126	0.48642	rs2013208	7.61E-06	0.04648663	3.2717e-310	0	Uterus
ENSG00000006125	AP2B1	17	33913918	34053436	ENSG00000143774	GUK1	1	228327663	228336685	0.010272	7.1611	1.51E-08	0.00284257	0.00511	0.78328	0.5831	rs10512472	1.38E-20	0	3.62E-14	0	Heart_Left_Ventricle
ENSG00000142102	ATHL1	11	289138	296107	ENSG00000144677	CTDSPL	3	37903451	38025960	0.015343	3.9514	1.54E-08	0.0028846	0.00095943	0.44128	0.64335	rs11602954	1.10E-48	0	8.23E-62	0	Thyroid
ENSG00000142102	ATHL1	11	289138	296107	ENSG00000144677	CTDSPL	3	37903451	38025960	0.015343	3.9514	1.54E-08	0.0028846	0.00095943	0.44128	0.64335	rs17655730	1.49E-41	0	1.10E-69	0	Muscle_Skeletal
ENSG00000142102	ATHL1	11	289138	296107	ENSG00000144677	CTDSPL	3	37903451	38025960	0.015343	3.9514	1.54E-08	0.0028846	0.00095943	0.44128	0.64335	rs505404	1.75E-49	0	3.83E-63	0	Lung
ENSG00000172123	SLFN12	17	33738079	33760302	ENSG00000140564	FURIN	15	91411822	91426688	0.013757	4.4841	1.59E-08	0.00296862	0.0059865	0.91843	0.48072	rs225245	2.51E-30	0	NA	NA	Cells_EBV-transformed_lymphocytes
ENSG00000172123	SLFN12	17	33738079	33760302	ENSG00000140564	FURIN	15	91411822	91426688	0.013757	4.4841	1.59E-08	0.00296862	0.0059865	0.91843	0.48072	rs9915021	2.81E-16	0	NA	NA	Breast_Mammary_Tissue
ENSG00000092871	RFFL	17	33341759	33416338	ENSG00000136732	GYPC	2	127413509	127454246	0.0097478	7.9258	1.60E-08	0.00297915	0.0066751	0.76692	0.63212	rs9915021	3.56E-10	2.15E-05	NA	NA	Adipose_Subcutaneous
ENSG00000092871	RFFL	17	33341759	33416338	ENSG00000136732	GYPC	2	127413509	127454246	0.0097478	7.9258	1.60E-08	0.00297915	0.0066751	0.76692	0.63212	rs9915021	3.56E-10	2.15E-05	NA	NA	Artery_Tibial
ENSG00000172123	SLFN12	17	33738079	33760302	ENSG00000160445	ZER1	9	131492065	131534693	0.013746	4.4803	1.63E-08	0.0030286	0.0092501	1.4238	0.20231	rs225245	4.35E-13	0	NA	NA	Cells_EBV-transformed_lymphocytes
ENSG00000172123	SLFN12	17	33738079	33760302	ENSG00000160445	ZER1	9	131492065	131534693	0.013746	4.4803	1.63E-08	0.0030286	0.0092501	1.4238	0.20231	rs9915021	1.28E-09	5.83E-05	NA	NA	Breast_Mammary_Tissue
ENSG00000131771	PPP1R1B	17	37782993	37792879	ENSG00000162551	ALPL	1	21835858	21904905	0.011167	6.0584	1.69E-08	0.00313158	0.017863	1.843	0.057129	rs3859192	3.20E-29	0	NA	NA	Adipose_Subcutaneous
ENSG00000131771	PPP1R1B	17	37782993	37792879	ENSG00000162551	ALPL	1	21835858	21904905	0.011167	6.0584	1.69E-08	0.00313158	0.017863	1.843	0.057129	rs3894194	2.28E-14	0	NA	NA	Heart_Atrial_Appendage
ENSG00000131771	PPP1R1B	17	37782993	37792879	ENSG00000162551	ALPL	1	21835858	21904905	0.011167	6.0584	1.69E-08	0.00313158	0.017863	1.843	0.057129	rs4794820	1.24E-10	0	NA	NA	Heart_Left_Ventricle
ENSG00000131771	PPP1R1B	17	37782993	37792879	ENSG00000162551	ALPL	1	21835858	21904905	0.011167	6.0584	1.69E-08	0.00313158	0.017863	1.843	0.057129	rs9303280	4.17E-09	0.00010539	NA	NA	Heart_Atrial_Appendage
ENSG00000008838	MED24	17	38175350	38217468	ENSG00000110080	ST3GAL4	11	126225535	126310239	0.0086417	10.532	1.70E-08	0.00315433	0.011946	2.7718	0.026199	rs3859192	1.04E-10	0	1.47E-40	0	Whole_Blood
ENSG00000008838	MED24	17	38175350	38217468	ENSG00000110080	ST3GAL4	11	126225535	126310239	0.0086417	10.532	1.70E-08	0.00315433	0.011946	2.7718	0.026199	rs8078723	4.29E-14	0	5.30E-78	0	Adipose_Subcutaneous
ENSG00000008838	MED24	17	38175350	38217468	ENSG00000110080	ST3GAL4	11	126225535	126310239	0.0086417	10.532	1.70E-08	0.00315433	0.011946	2.7718	0.026199	rs8078723	4.29E-14	0	5.30E-78	0	Artery_Tibial
ENSG00000008838	MED24	17	38175350	38217468	ENSG00000110080	ST3GAL4	11	126225535	126310239	0.0086417	10.532	1.70E-08	0.00315433	0.011946	2.7718	0.026199	rs8078723	4.29E-14	0	5.30E-78	0	Cells_Transformed_fibroblasts
ENSG00000008838	MED24	17	38175350	38217468	ENSG00000110080	ST3GAL4	11	126225535	126310239	0.0086417	10.532	1.70E-08	0.00315433	0.011946	2.7718	0.026199	rs8078723	4.29E-14	0	5.30E-78	0	Lung
ENSG00000008838	MED24	17	38175350	38217468	ENSG00000110080	ST3GAL4	11	126225535	126310239	0.0086417	10.532	1.70E-08	0.00315433	0.011946	2.7718	0.026199	rs8078723	4.29E-14	0	5.30E-78	0	Pituitary
ENSG00000008838	MED24	17	38175350	38217468	ENSG00000110080	ST3GAL4	11	126225535	126310239	0.0086417	10.532	1.70E-08	0.00315433	0.011946	2.7718	0.026199	rs8078723	4.29E-14	0	5.30E-78	0	Skin_Sun_Exposed_Lower_leg
ENSG00000008838	MED24	17	38175350	38217468	ENSG00000110080	ST3GAL4	11	126225535	126310239	0.0086417	10.532	1.70E-08	0.00315433	0.011946	2.7718	0.026199	rs8078723	4.29E-14	0	5.30E-78	0	Spleen
ENSG00000008838	MED24	17	38175350	38217468	ENSG00000110080	ST3GAL4	11	126225535	126310239	0.0086417	10.532	1.70E-08	0.00315433	0.011946	2.7718	0.026199	rs8078723	4.29E-14	0	5.30E-78	0	Stomach

ENSG00000008838	MED24	17	38175350	38217468	ENSG00000110080	ST3GAL4	11	126225535	126310239	0.0086417	10.532	1.70E-08	0.00315433	0.011946	2.7718	0.026199	rs8078723	4.29E-14	0	5.30E-78	0	Thyroid
ENSG00000008838	MED24	17	38175350	38217468	ENSG00000110080	ST3GAL4	11	126225535	126310239	0.0086417	10.532	1.70E-08	0.00315433	0.011946	2.7718	0.026199	rs4794822	2.95E-13	0	1.32E-79	0	Esophagus_Gastroesophageal_Junction
ENSG00000008838	MED24	17	38175350	38217468	ENSG00000110080	ST3GAL4	11	126225535	126310239	0.0086417	10.532	1.70E-08	0.00315433	0.011946	2.7718	0.026199	rs4794822	2.95E-13	0	1.32E-79	0	Lung
ENSG00000008838	MED24	17	38175350	38217468	ENSG00000110080	ST3GAL4	11	126225535	126310239	0.0086417	10.532	1.70E-08	0.00315433	0.011946	2.7718	0.026199	rs4794822	2.95E-13	0	1.32E-79	0	Spleen
ENSG00000008838	MED24	17	38175350	38217468	ENSG00000110080	ST3GAL4	11	126225535	126310239	0.0086417	10.532	1.70E-08	0.00315433	0.011946	2.7718	0.026199	rs4794822	2.95E-13	0	1.32E-79	0	Whole_Blood
ENSG00000008838	MED24	17	38175350	38217468	ENSG00000110080	ST3GAL4	11	126225535	126310239	0.0086417	10.532	1.70E-08	0.00315433	0.011946	2.7718	0.026199	rs4065321	1.97E-13	0	1.24E-42	0	Pituitary
ENSG00000180376	CCDC66	3	56591189	56653929	ENSG00000174175	SELP	1	169558087	169599431	0.0091881	8.9617	1.74E-08	0.00319772	0.010113	1.8716	0.09676	rs17825630	1.73E-18	0	NA	NA	Brain_Anterior_cingulate_cortex_BA24
ENSG00000180376	CCDC66	3	56591189	56653929	ENSG00000174175	SELP	1	169558087	169599431	0.0091881	8.9617	1.74E-08	0.00319772	0.010113	1.8716	0.09676	rs17825630	1.73E-18	0	NA	NA	Cells_EBV-transformed_lymphocytes
ENSG00000180376	CCDC66	3	56591189	56653929	ENSG00000174175	SELP	1	169558087	169599431	0.0091881	8.9617	1.74E-08	0.00319772	0.010113	1.8716	0.09676	rs1354034	3.27170000000001e-	0	NA	NA	Brain_Cerebellar_Hemisphere
ENSG00000180376	CCDC66	3	56591189	56653929	ENSG00000174175	SELP	1	169558087	169599431	0.0091881	8.9617	1.74E-08	0.00319772	0.010113	1.8716	0.09676	rs1354034	3.27170000000001e-	0	NA	NA	Cells_EBV-transformed_lymphocytes
ENSG00000180376	CCDC66	3	56591189	56653929	ENSG00000174175	SELP	1	169558087	169599431	0.0091881	8.9617	1.74E-08	0.00319772	0.010113	1.8716	0.09676	rs1354034	3.27170000000001e-	0	NA	NA	Testis
ENSG00000008838	MED24	17	38175350	38217468	ENSG00000072952	MRVI1	11	10594638	10715535	0.0086271	10.514	1.76E-08	0.00324014	0.0073738	1.703	0.14717	rs3859192	5.47E-07	0.00481306	1.47E-40	0	Whole_Blood
ENSG00000172057	ORMDL3	17	38077294	38083854	ENSG00000182541	LIMK2	22	31608260	31676066	0.0091803	8.9541	1.77E-08	0.00324587	0.010395	1.6019	0.14338	rs4794820	3.89E-08	0.00052744	3.2717e-310	0	Spleen
ENSG00000172057	ORMDL3	17	38077294	38083854	ENSG00000182541	LIMK2	22	31608260	31676066	0.0091803	8.9541	1.77E-08	0.00324587	0.010395	1.6019	0.14338	rs2872507	5.75E-06	0.03701626	3.2717e-310	0	Whole_Blood
ENSG00000172057	ORMDL3	17	38077294	38083854	ENSG00000182541	LIMK2	22	31608260	31676066	0.0091803	8.9541	1.77E-08	0.00324587	0.010395	1.6019	0.14338	rs8069176	8.90E-07	0.00743469	3.2717e-310	0	Whole_Blood
ENSG00000132139	GAS2L2	17	34071530	34079897	ENSG00000029534	ANK1	8	41510739	41754280	0.010681	6.5166	1.77E-08	0.00324884	0.0058022	0.66605	0.72176	rs9915021	6.17E-12	0	NA	NA	Small_Intestine_Terminal_Ileum
ENSG00000205045	SLFN12L	17	33800708	33864880	ENSG00000198892	SHISA4	1	201857808	201861434	0.0102	7.1107	1.77E-08	0.00325161	0.0078994	1.0397	0.40146	rs8082605	5.00E-06	0.03292855	2.49E-31	0	Adipose_Subcutaneous
ENSG00000205045	SLFN12L	17	33800708	33864880	ENSG00000198892	SHISA4	1	201857808	201861434	0.0102	7.1107	1.77E-08	0.00325161	0.0078994	1.0397	0.40146	rs8082605	5.00E-06	0.03292855	2.49E-31	0	Adipose_Visceral_Omentum
ENSG00000205045	SLFN12L	17	33800708	33864880	ENSG00000198892	SHISA4	1	201857808	201861434	0.0102	7.1107	1.77E-08	0.00325161	0.0078994	1.0397	0.40146	rs8082605	5.00E-06	0.03292855	2.49E-31	0	Heart_Left_Ventricle
ENSG00000205045	SLFN12L	17	33800708	33864880	ENSG00000198892	SHISA4	1	201857808	201861434	0.0102	7.1107	1.77E-08	0.00325161	0.0078994	1.0397	0.40146	rs8082605	5.00E-06	0.03292855	2.49E-31	0	Nerve_Tibial
ENSG00000205045	SLFN12L	17	33800708	33864880	ENSG00000198892	SHISA4	1	201857808	201861434	0.0102	7.1107	1.77E-08	0.00325161	0.0078994	1.0397	0.40146	rs10512472	2.30E-06	0.01730851	4.96E-30	0	Pancreas
ENSG00000132139	GAS2L2	17	34071530	34079897	ENSG00000238243	OR2W3	1	248058859	248060449	0.010677	6.5146	1.78E-08	0.00325792	0.011913	1.376	0.20295	rs9915021	7.68E-06	0.04691593	NA	NA	Small_Intestine_Terminal_Ileum
ENSG00000174885	NLRP6	11	278365	285359	ENSG00000173210	ABLIM3	5	148521046	148640105	0.010185	7.1001	1.83E-08	0.00333941	0.020369	2.107	0.026587	rs11602954	2.06E-35	0	2.12E-70	0	Nerve_Tibial
ENSG00000174885	NLRP6	11	278365	285359	ENSG00000173210	ABLIM3	5	148521046	148640105	0.010185	7.1001	1.83E-08	0.00333941	0.020369	2.107	0.026587	rs17655730	1.22E-33	0	5.43E-117	0	Artery_Aorta
ENSG00000174885	NLRP6	11	278365	285359	ENSG00000173210	ABLIM3	5	148521046	148640105	0.010185	7.1001	1.83E-08	0.00333941	0.020369	2.107	0.026587	rs17655730	1.22E-33	0	5.43E-117	0	Nerve_Tibial
ENSG00000174885	NLRP6	11	278365	285359	ENSG00000173210	ABLIM3	5	148521046	148640105	0.010185	7.1001	1.83E-08	0.00333941	0.020369	2.107	0.026587	rs17655730	1.22E-33	0	5.43E-117	0	Skin_Sun_Exposed_Lower_leg
ENSG00000174885	NLRP6	11	278365	285359	ENSG00000173210	ABLIM3	5	148521046	148640105	0.010185	7.1001	1.83E-08	0.00333941	0.020369	2.107	0.026587	rs505404	3.10E-39	0	2.65E-88	0	Nerve_Tibial
ENSG00000174885	NLRP6	11	278365	285359	ENSG00000173210	ABLIM3	5	148521046	148640105	0.010185	7.1001	1.83E-08	0.00333941	0.020369	2.107	0.026587	rs505404	3.10E-39	0	2.65E-88	0	Prostate
ENSG00000100417	PMM1	22	41973812	41985894	ENSG00000175556	LONRF3	X	118108581	118156888	0.0096717	7.8634	1.90E-08	0.00343944	0.015342	2.3761	0.027712	rs4822024	1.77E-35	0	6.14E-122	0	Thyroid
ENSG00000180376	CCDC66	3	56591189	56653929	ENSG00000124491	F13A1	6	6144318	6321246	0.0091449	8.9192	1.92E-08	0.00346957	0.010385	1.9225	0.088117	rs17825630	8.88E-16	0	NA	NA	Brain_Anterior_cingulate_cortex_BA24
ENSG00000180376	CCDC66	3	56591189	56653929	ENSG00000124491	F13A1	6	6144318	6321246	0.0091449	8.9192	1.92E-08	0.00346957	0.010385	1.9225	0.088117	rs17825630	8.88E-16	0	NA	NA	Cells_EBV-transformed_lymphocytes
ENSG00000180376	CCDC66	3	56591189	56653929	ENSG00000124491	F13A1	6	6144318	6321246	0.0091449	8.9192	1.92E-08	0.00346957	0.010385	1.9225	0.088117	rs1354034	3.27170000000001e-	0	NA	NA	Brain_Cerebellar_Hemisphere
ENSG00000180376	CCDC66	3	56591189	56653929	ENSG00000124491	F13A1	6	6144318	6321246	0.0091449	8.9192	1.92E-08	0.00346957	0.010385	1.9225	0.088117	rs1354034	3.27170000000001e-	0	NA	NA	Cells_EBV-transformed_lymphocytes
ENSG00000180376	CCDC66	3	56591189	56653929	ENSG00000124491	F13A1	6	6144318	6321246	0.0091449	8.9192	1.92E-08	0.00346957	0.010385	1.9225	0.088117	rs1354034	3.27170000000001e-	0	NA	NA	Testis
ENSG00000172123	SLFN12	17	33738079	33760302	ENSG00000118046	STK11	19	1189406	1226662	0.013662	4.4528	1.92E-08	0.00347303	0.0063836	0.97976	0.43762	rs225245	1.03E-13	0	NA	NA	Cells_EBV-transformed_lymphocytes
ENSG00000172123	SLFN12	17	33738079	33760302	ENSG00000118046	STK11	19	1189406	1226662	0.013662	4.4528	1.92E-08	0.00347303	0.0063836	0.97976	0.43762	rs9915021	3.14E-08	0.00040834	NA	NA	Breast_Mammary_Tissue
ENSG00000118640	VAMP8	2	85788685	85809154	ENSG00000141096	DPEP3	16	68009566	68014732	0.0096572	7.8515	1.96E-08	0.00353128	0.050295	6.9149	4.89E-08	rs10187424	6.33E-97	0	3.2717e-310	0	Esophagus_Mucosa
ENSG00000205045	SLFN12L	17	33800708	33864880	ENSG00000143774	GUK1	1	228327663	228336685	0.010153	7.0772	1.97E-08	0.00354727	0.0073013	0.96036	0.45902	rs8082605	3.85E-15	0	2.49E-31	0	Adipose_Subcutaneous
ENSG00000205045	SLFN12L	17	33800708	33864880	ENSG00000143774	GUK1	1	228327663	228336685	0.010153	7.0772	1.97E-08	0.00354727	0.0073013	0.96036	0.45902	rs8082605	3.85E-15	0	2.49E-31	0	Adipose_Visceral_Omentum
ENSG00000205045	SLFN12L	17	33800708	33864880	ENSG00000143774	GUK1	1	228327663	228336685	0.010153	7.0772	1.97E-08	0.00354727	0.0073013	0.96036	0.45902	rs8082605	3.85E-15	0	2.49E-31	0	Heart_Left_Ventricle
ENSG00000205045	SLFN12L	17	33800708	33864880	ENSG00000143774	GUK1	1	228327663	228336685	0.010153	7.0772	1.97E-08	0.00354727	0.0073013	0.96036	0.45902	rs8082605	3.85E-15	0	2.49E-31	0	Nerve_Tibial
ENSG00000205045	SLFN12L	17	33800708	33864880	ENSG00000143774	GUK1	1	228327663	228336685	0.010153	7.0772	1.97E-08	0.00354727	0.0073013	0.96036	0.45902	rs10512472	1.38E-20	0	4.96E-30	0	Pancreas
ENSG00000154768	C17orf50	17	34087916	34092098	ENSG00000029534	ANK1	8	41510739	41754280	0.0096435	7.8402	2.02E-08	0.00362529	0.014862	1.9698	0.056334	rs9915021	6.17E-12	0	NA	NA	Pituitary
ENSG00000141150	RASL10B	17	34058668	34070540	ENSG00000095303	PTGS1	9	125132824	125157982	0.010613	6.4748	2.05E-08	0.00367467	0.042609	5.8111	1.31E-06	rs9915021	5.42E-64	0	NA	NA	Cells_Transformed_fibroblasts
ENSG00000188076	SCGB1C1	11	193080	194573	ENSG00000122786	CALD1	7	134429003	134655479	0.0064789	31.536	2.07E-08	0.00369221	0.00083638	0.77012	0.38041	rs11602954	3.38E-37	0	3.14E-203	0	Whole_Blood
ENSG00000188076	SCGB1C1	11	193080	194573	ENSG00000122786	CALD1	7	134429003	134655479	0.0064789	31.536	2.07E-08	0.00369221	0.00083638	0.77012	0.38041	rs17655730	3.56E-40	0	1.60E-163	0	Whole_Blood
ENSG00000188076	SCGB1C1	11	193080	194573	ENSG00000122786	CALD1	7	134429003	134655479	0.0064789	31.536	2.07E-08	0.00369221	0.00083638	0.77012	0.38041	rs505404	1.84E-40	0	5.81E-188	0	Whole_Blood
ENSG00000115486	GGCX	2	85774743	85788670	ENSG00000169439	SDC2	8	97505579	97624000	0.0085575	10.429	2.07E-08	0.00369363	0.031167	7.375	7.58E-06	rs10187424	7.24E-15	0	3.2717e-310	0	Artery_Aorta
ENSG00000115486	GGCX	2	85774743	85788670	ENSG00000169439	SDC2	8	97505579	97624000	0.0085575	10.429	2.07E-08	0.00369363	0.031167	7.375	7.58E-06	rs10187424	7.24E-15	0	3.2717e-310	0	Artery_Tibial
ENSG00000115486	GGCX	2	85774743	85788670	ENSG00000169439	SDC2	8	97505579	97624000	0.0085575	10.429	2.07E-08	0.00369363	0.031167	7.375	7.58E-06	rs10187424	7.24E-15	0	3.2717e-310	0	Brain_Cortex
ENSG00000115486	GGCX	2	85774743	85788670	ENSG00000169439	SDC2	8	97505579	97624000	0.0085575	10.429	2.07E-08	0.00369363	0.031167	7.375	7.58E-06	rs10187424	7.24E-15	0	3.2717e-310	0	Colon_Transverse
ENSG00000115486	GGCX	2	85774743	85788670	ENSG00000169439	SDC2	8	97505579	97624000	0.0085575	10.429	2.07E-08	0.00369363	0.031167	7.375	7.58E-06	rs10187424	7.24E-15	0	3.2717e-310	0	Nerve_Tibial
ENSG00000174885	NLRP6	11	278365	285359	ENSG00000120885	CLU	8	27454434	27472548	0.010124	7.0571	2.10E-08	0.00373476	0.019217	1.9854	0.038013	rs11602954	1.24E-10	0	2.12E-70	0	Nerve_Tibial
ENSG00000174885	NLRP6	11	278365	285359	ENSG00000120885	CLU	8	27454434	27472548	0.010124	7.0571	2.10E-08	0.00373476	0.019217	1.9854	0.038013	rs17655730	2.73E-13	0	5.43E-117	0	Artery_Aorta
ENSG00000174885	NLRP6	11	278365	285359	ENSG00000120885	CLU	8	27454434	27472548	0.010124	7.0571	2.10E-08	0.00373476	0.019217	1.9854	0.038013	rs17655730	2.73E-13	0	5.43E-117	0	Nerve_Tibial
ENSG00000174885	NLRP6	11	278365	285359	ENSG00000120885	CLU	8	27454434	27472548	0.010124	7.0571	2.10E-08	0.00373476	0.019217	1.9854	0.038013	rs17655730	2.73E-13	0	5.43E-117	0	Skin_Sun_Exposed_Lower_leg
ENSG00000174885	NLRP6	11	278365	285359	ENSG00000120885	CLU	8	27454434	27472548	0.010124	7.0571	2.10E-08	0.00373476	0.019217	1.9854	0.038013	rs505404	7.47E-15	0	2.65E-88	0	Nerve_Tibial
ENSG00000174885	NLRP6	11	278365	285359	ENSG00000120885	CLU	8	27454434	27472548	0.010124	7.0571	2.10E-08	0.00373476	0.019217	1.9854	0.038013	rs505404	7.47E-15	0	2.65E-88	0	Prostate
ENSG00000122592	HOXA7	7	27193335	27197555	ENSG00000176358	TAC4	17	47915671	47925379	0.0085392	10.406	2.16E-08	0.00383124	0.0022956	0.52748	0.71557	rs706015	3.17E-13	0	NA	NA	Prostate
ENSG00000168374	ARF4	3	57557090	57583947	ENSG00000185052	SLC24A3	20	19193290	19703581	0.0090793	8.8546	2.23E-08	0.00392231	0.025362	4.7673	0.00026258	rs17825630	8.36E-20	0	NA	NA	Artery_Tibial
ENSG00000168374	ARF4	3	57557090	57583947	ENSG00000185052	SLC24A3	20	19193290	19703581	0.0090793	8.8546	2.23E-08	0.00392231	0.025362	4.7673	0.00026258	rs17825630	8.36E-20	0	NA	NA	Heart_Atrial_Appendage
ENSG00000108733	PEX12	17	33901814	33905882	ENSG00000174175	SELP	1	169558087	169599431	0.0090795	8.8548	2.23E-08	0.00392231	0.0039248	0.72186	0.6071	rs10512472	1.49E-15	0	1.06E-12	0	Brain_Caudate_basal_ganglia
ENSG00000154760	SLFN13	17	33762115	33775856	ENSG00000090674	MCOLN1	19	7587512	7595387	0.011919	5.2921	2.25E-08	0.00395576	0.0087485	0.66855	0.78268	rs225245	6.87E-23	0	NA	NA	Liver
ENSG00000154760	SLFN13	17	33762115	33775856	ENSG00000090674	MCOLN1	19	7587512	7595387	0.011919	5.2921	2.25E-08	0.00395576	0.0087485	0.66855	0.78268	rs8082605	1.84E-14	0	2.08E-19	0	Artery_Tibial
ENSG00000154760	SLFN13	17	33762115	33775856	ENSG00000090674	MCOLN1	19	7587512	7595387	0.011919	5.2921	2.25E-08	0.00395576	0.0087485	0.66855	0.78268	rs8082605	1.84E-14	0	2.08E-19	0	Esophagus_Mucosa
ENSG00000154760	SLFN13	17	33762115	33775856	ENSG00000090674	MCOLN1	19	7587512	7595387	0.011919	5.2921	2.25E-08	0.00395576	0.0087485	0.66855	0.78268	rs8082605	1.84E-14	0	2.08E-19	0	Nerve_Tibial
ENSG00000154760	SLFN13	17	33762115	33775856	ENSG00000090674	MCOLN1	19	7587512	7595387	0.011919	5.2921	2.25E-08	0.00395576	0.0087485	0.66855	0.78268	rs8082605	1.84E-14	0	2.08E-19	0	Pituitary
ENSG00000154760	SLFN13	17	33762115	33775856	ENSG00000090674	MCOLN1	19	7587512	7595387	0.011919	5.2921	2.25E-08	0.00395576	0.0087485	0.66855	0.78268	rs10512472	3.45E-14	0	6.34E-38	0	Uterus
ENSG00000092871	RFFL	17	33341759	33416338	ENSG00000115649	CNPPD1	2	220036619	220042035	0.0095912	7.7973	2.27E-08	0.00398178	NA	NA	NA	rs9915021	3.39E-19	0	NA	NA	Adipose_Subcutaneous
ENSG00000092871	RFFL	17	33341759	33416338	ENSG00000115649	CNPPD1	2	220036619	220042035	0.0095912	7.7973	2.27E-08	0.00398178	NA	NA	NA	rs9915021	3.39E-19	0	NA	NA	Artery_Tibial
ENSG00000100890	KIAA0391	14	35593499	35593616	ENSG00000157017	GHRL	3	10328388	10334631	0.011017	5.9759	2.32E-08	0.00405699	0.060623	6.5396	4.52E-09	rs2143950	2.92E-22	0	NA	NA	Artery_Tibial
ENSG00000100890	KIAA0391	14	35593499	35593616	ENSG00000157017	GHRL	3	10328388	10334631	0.011017	5.9759	2.32E-08	0.00405699	0.060623	6.5396	4.52E-09	rs2143950	2.92E-22	0	NA	NA	Skin_Sun_Exposed_Lower_leg
ENSG00000100890	KIAA0391	14	35593499	35593616	ENSG00000157017	GHRL	3	10328388	10334631	0.011017	5.9759	2.32E-08	0.00405699	0.060623	6.5396	4.52E-09	rs2143950	2.92E-22	0	NA	NA	Spleen
ENSG00000177963	RIC8A	11	207511	215113	ENSG00000124491	F13A1	6	6144318	6321246	0.0090403	8.8162	2.43E-08	0.00422908	0.014257	1.6506	0.10669	rs11602954	7.25E-48	0	7.55E-62	0	Adipose_Subcutaneous
ENSG00000177963	RIC8A	11	207511	215113	ENSG00000124491	F13A1	6	6144318	6321246	0.0090403	8.8162	2.43E-08	0.00422908	0.014257	1.6506	0.10669	rs11602954	7.25E-48	0	7.55E-62	0	Artery_Aorta
ENSG00000177963	RIC8A	11	207511	215113	ENSG00000124491	F13A1	6	6144318	6321246	0.0090403	8.8162	2.43E-08	0.00422908	0.014257	1.6506	0.10669	rs11602954	7.25E-48	0	7.55E-62	0	Artery_Tibial
ENSG00000177963	RIC8A	11	207511	215113	ENSG00000124491	F13A1	6	6144318	6321246	0.0090403	8.8162	2.43E-08	0.00422908	0.014257	1.6506	0.10669	rs11602954	7.25E-48	0	7.55E-62	0	Brain_Caudate_basal_ganglia
ENSG00000177963	RIC8A	11	207511	215113	ENSG00000124491	F13A1	6	6144318	6321246	0.0090403	8.8162	2.43E-08	0.00422908	0.014257	1.6506	0.10669	rs11602954	7.25E-48	0	7.55E-62	0	Brain_Frontal_Cortex_BA9
ENSG00000177963	RIC8A	11	207511	215113	ENSG00000124491	F13A1	6	6144318	6321246	0.0090403	8.8162	2.43E-08	0.00422908	0.014257	1.6506	0.10669	rs11602954	7.25E-48	0	7.55E-62	0	Colon_Sigmoid
ENSG00000177963	RIC8A	11	207511	215113	ENSG00000124491	F13A1	6	6144318	6321246	0.0090403	8.8162	2.43E-08	0.00422908	0.014257	1.6506	0.10669	rs11602954	7.25E-48	0	7.55E-62	0	Colon_Transverse
ENSG00000177963	RIC8A	11	207511	215113	ENSG00000124491	F13A1	6	6144318	6321246	0.0090403	8.8162	2.43E-08	0.00422908	0.014257	1.6506	0.10669	rs11602954	7.25E-48	0	7.55E-62	0	Esophagus_Gastroesophageal_Junction
ENSG00000177963	RIC8A	11	207511	215113	ENSG00000124491	F13A1	6	6144318	6321246	0.0090403	8.8162	2.43E-08	0.00422908	0.014257	1.6506	0.10669	rs11602954	7.25E-48	0	7.55E-62	0	Esophagus_Mucosa

ENSG00000177963	RIC8A	11	207511	215113	ENSG00000124491	F13A1	6	6144318	6321246	0.0090403	8.8162	2.43E-08	0.00422908	0.014257	1.6506	0.10669	rs11602954	7.25E-48	0	7.55E-62	0	Esophagus_Muscularis
ENSG00000177963	RIC8A	11	207511	215113	ENSG00000124491	F13A1	6	6144318	6321246	0.0090403	8.8162	2.43E-08	0.00422908	0.014257	1.6506	0.10669	rs11602954	7.25E-48	0	7.55E-62	0	Lung
ENSG00000177963	RIC8A	11	207511	215113	ENSG00000124491	F13A1	6	6144318	6321246	0.0090403	8.8162	2.43E-08	0.00422908	0.014257	1.6506	0.10669	rs11602954	7.25E-48	0	7.55E-62	0	Muscle_Skeletal
ENSG00000177963	RIC8A	11	207511	215113	ENSG00000124491	F13A1	6	6144318	6321246	0.0090403	8.8162	2.43E-08	0.00422908	0.014257	1.6506	0.10669	rs11602954	7.25E-48	0	7.55E-62	0	Nerve_Tibial
ENSG00000177963	RIC8A	11	207511	215113	ENSG00000124491	F13A1	6	6144318	6321246	0.0090403	8.8162	2.43E-08	0.00422908	0.014257	1.6506	0.10669	rs11602954	7.25E-48	0	7.55E-62	0	Skin_Not_Sun_Exposed_Suprapubic
ENSG00000177963	RIC8A	11	207511	215113	ENSG00000124491	F13A1	6	6144318	6321246	0.0090403	8.8162	2.43E-08	0.00422908	0.014257	1.6506	0.10669	rs11602954	7.25E-48	0	7.55E-62	0	Skin_Sun_Exposed_Lower_leg
ENSG00000177963	RIC8A	11	207511	215113	ENSG00000124491	F13A1	6	6144318	6321246	0.0090403	8.8162	2.43E-08	0.00422908	0.014257	1.6506	0.10669	rs11602954	7.25E-48	0	7.55E-62	0	Thyroid
ENSG00000177963	RIC8A	11	207511	215113	ENSG00000124491	F13A1	6	6144318	6321246	0.0090403	8.8162	2.43E-08	0.00422908	0.014257	1.6506	0.10669	rs17655730	3.37E-44	0	5.10E-36	0	Adipose_Subcutaneous
ENSG00000177963	RIC8A	11	207511	215113	ENSG00000124491	F13A1	6	6144318	6321246	0.0090403	8.8162	2.43E-08	0.00422908	0.014257	1.6506	0.10669	rs17655730	3.37E-44	0	5.10E-36	0	Esophagus_Mucosa
ENSG00000177963	RIC8A	11	207511	215113	ENSG00000124491	F13A1	6	6144318	6321246	0.0090403	8.8162	2.43E-08	0.00422908	0.014257	1.6506	0.10669	rs17655730	3.37E-44	0	5.10E-36	0	Esophagus_Muscularis
ENSG00000177963	RIC8A	11	207511	215113	ENSG00000124491	F13A1	6	6144318	6321246	0.0090403	8.8162	2.43E-08	0.00422908	0.014257	1.6506	0.10669	rs17655730	3.37E-44	0	5.10E-36	0	Heart_Left_Ventricle
ENSG00000177963	RIC8A	11	207511	215113	ENSG00000124491	F13A1	6	6144318	6321246	0.0090403	8.8162	2.43E-08	0.00422908	0.014257	1.6506	0.10669	rs17655730	3.37E-44	0	5.10E-36	0	Ovary
ENSG00000177963	RIC8A	11	207511	215113	ENSG00000124491	F13A1	6	6144318	6321246	0.0090403	8.8162	2.43E-08	0.00422908	0.014257	1.6506	0.10669	rs17655730	3.37E-44	0	5.10E-36	0	Pancreas
ENSG00000177963	RIC8A	11	207511	215113	ENSG00000124491	F13A1	6	6144318	6321246	0.0090403	8.8162	2.43E-08	0.00422908	0.014257	1.6506	0.10669	rs17655730	3.37E-44	0	5.10E-36	0	Skin_Not_Sun_Exposed_Suprapubic
ENSG00000177963	RIC8A	11	207511	215113	ENSG00000124491	F13A1	6	6144318	6321246	0.0090403	8.8162	2.43E-08	0.00422908	0.014257	1.6506	0.10669	rs505404	5.93E-51	0	3.66E-47	0	Artery_Aorta
ENSG00000177963	RIC8A	11	207511	215113	ENSG00000124491	F13A1	6	6144318	6321246	0.0090403	8.8162	2.43E-08	0.00422908	0.014257	1.6506	0.10669	rs505404	5.93E-51	0	3.66E-47	0	Thyroid
ENSG00000186075	ZPBP2	17	38024417	38034149	ENSG00000146592	CREB5	7	28338940	28865511	0.0064132	31.214	2.44E-08	0.00423297	0.0001964	0.18073	0.67085	rs10445308	2.63E-06	0.01942416	NA	NA	Testis
ENSG00000186075	ZPBP2	17	38024417	38034149	ENSG00000146592	CREB5	7	28338940	28865511	0.0064132	31.214	2.44E-08	0.00423297	0.0001964	0.18073	0.67085	rs3859192	1.81E-15	0	NA	NA	Testis
ENSG00000186075	ZPBP2	17	38024417	38034149	ENSG00000146592	CREB5	7	28338940	28865511	0.0064132	31.214	2.44E-08	0.00423297	0.0001964	0.18073	0.67085	rs4794820	2.26E-09	7.41E-05	NA	NA	Testis
ENSG00000172716	SLFN11	17	33677324	33700720	ENSG00000238243	OR2W3	1	248058859	248060449	0.012283	5.0003	2.56E-08	0.00442814	0.01322	1.0149	0.43262	rs225245	4.87E-16	0	5.77E-07	0.001884469	Cells_Transformed_fibroblasts
ENSG00000172716	SLFN11	17	33677324	33700720	ENSG00000238243	OR2W3	1	248058859	248060449	0.012283	5.0003	2.56E-08	0.00442814	0.01322	1.0149	0.43262	rs8082605	1.50E-06	0.01173846	NA	NA	Adrenal_Gland
ENSG00000108733	PEX12	17	33901814	33905882	ENSG00000159335	PTMS	12	6874682	6880116	0.0090167	8.7931	2.57E-08	0.00442838	0.012733	2.3627	0.038272	rs10512472	3.56E-10	2.15E-05	1.06E-12	0	Brain_Caudate_basal_ganglia
ENSG00000141741	MIEN1	17	37885409	37886014	ENSG00000196663	TECPR2	14	102829300	102968818	0.009014	8.7904	2.58E-08	0.00444442	0.004185	0.76991	0.57154	rs7212938	6.67E-07	0.0058136	NA	NA	Testis
ENSG00000141150	RASL10B	17	34058668	34070540	ENSG00000204463	BAG6	6	31606805	31620170	0.010503	6.4074	2.60E-08	0.00446978	0.034097	4.6092	4.43E-05	rs9915021	2.40E-12	0	NA	NA	Cells_Transformed_fibroblasts
ENSG00000142102	ATHL1	11	289138	296107	ENSG00000172572	PDE3A	12	20522179	20837315	0.015065	3.8785	2.62E-08	0.0044882	NA	NA	NA	rs17655730	1.27E-06	0.0101111	1.10E-69	0	Muscle_Skeletal
ENSG00000142102	ATHL1	11	289138	296107	ENSG00000172572	PDE3A	12	20522179	20837315	0.015065	3.8785	2.62E-08	0.0044882	NA	NA	NA	rs505404	3.08E-06	0.02212182	3.83E-63	0	Lung
ENSG00000141150	RASL10B	17	34058668	34070540	ENSG00000196961	AP2A1	19	50270225	50309510	0.010496	6.4031	2.64E-08	0.00452213	0.0062562	0.82202	0.56901	rs9915021	1.56E-11	0	NA	NA	Cells_Transformed_fibroblasts
ENSG00000174885	NLRP6	11	278365	285359	ENSG00000095303	PTGS1	9	125132824	125157982	0.010013	6.979	2.68E-08	0.00457749	0.018988	1.9613	0.040764	rs11602954	3.11E-14	0	2.12E-70	0	Nerve_Tibial
ENSG00000174885	NLRP6	11	278365	285359	ENSG00000095303	PTGS1	9	125132824	125157982	0.010013	6.979	2.68E-08	0.00457749	0.018988	1.9613	0.040764	rs17655730	1.75E-16	0	5.43E-117	0	Artery_Aorta
ENSG00000174885	NLRP6	11	278365	285359	ENSG00000095303	PTGS1	9	125132824	125157982	0.010013	6.979	2.68E-08	0.00457749	0.018988	1.9613	0.040764	rs17655730	1.75E-16	0	5.43E-117	0	Nerve_Tibial
ENSG00000174885	NLRP6	11	278365	285359	ENSG00000095303	PTGS1	9	125132824	125157982	0.010013	6.979	2.68E-08	0.00457749	0.018988	1.9613	0.040764	rs17655730	1.75E-16	0	5.43E-117	0	Skin_Sun_Exposed_Lower_leg
ENSG00000174885	NLRP6	11	278365	285359	ENSG00000095303	PTGS1	9	125132824	125157982	0.010013	6.979	2.68E-08	0.00457749	0.018988	1.9613	0.040764	rs505404	4.38E-17	0	2.65E-88	0	Nerve_Tibial
ENSG00000174885	NLRP6	11	278365	285359	ENSG00000095303	PTGS1	9	125132824	125157982	0.010013	6.979	2.68E-08	0.00457749	0.018988	1.9613	0.040764	rs505404	4.38E-17	0	2.65E-88	0	Prostate
ENSG00000142082	SIRT3	11	215458	236431	ENSG00000161911	TREML1	6	41117080	41122075	0.0078492	12.748	2.70E-08	0.00460144	0.0084421	1.9518	0.099868	rs11602954	8.99E-32	0	1.39E-80	0	Adipose_Subcutaneous
ENSG00000142082	SIRT3	11	215458	236431	ENSG00000161911	TREML1	6	41117080	41122075	0.0078492	12.748	2.70E-08	0.00460144	0.0084421	1.9518	0.099868	rs11602954	8.99E-32	0	1.39E-80	0	Artery_Tibial
ENSG00000142082	SIRT3	11	215458	236431	ENSG00000161911	TREML1	6	41117080	41122075	0.0078492	12.748	2.70E-08	0.00460144	0.0084421	1.9518	0.099868	rs11602954	8.99E-32	0	1.39E-80	0	Breast_Mammary_Tissue
ENSG00000142082	SIRT3	11	215458	236431	ENSG00000161911	TREML1	6	41117080	41122075	0.0078492	12.748	2.70E-08	0.00460144	0.0084421	1.9518	0.099868	rs11602954	8.99E-32	0	1.39E-80	0	Cells_Transformed_fibroblasts
ENSG00000142082	SIRT3	11	215458	236431	ENSG00000161911	TREML1	6	41117080	41122075	0.0078492	12.748	2.70E-08	0.00460144	0.0084421	1.9518	0.099868	rs11602954	8.99E-32	0	1.39E-80	0	Nerve_Tibial
ENSG00000142082	SIRT3	11	215458	236431	ENSG00000161911	TREML1	6	41117080	41122075	0.0078492	12.748	2.70E-08	0.00460144	0.0084421	1.9518	0.099868	rs505404	1.33E-35	0	1.05E-94	0	Adrenal_Gland
ENSG00000142082	SIRT3	11	215458	236431	ENSG00000161911	TREML1	6	41117080	41122075	0.0078492	12.748	2.70E-08	0.00460144	0.0084421	1.9518	0.099868	rs505404	1.33E-35	0	1.05E-94	0	Artery_Aorta
ENSG00000142082	SIRT3	11	215458	236431	ENSG00000161911	TREML1	6	41117080	41122075	0.0078492	12.748	2.70E-08	0.00460144	0.0084421	1.9518	0.099868	rs505404	1.33E-35	0	1.05E-94	0	Lung
ENSG00000142082	SIRT3	11	215458	236431	ENSG00000161911	TREML1	6	41117080	41122075	0.0078492	12.748	2.70E-08	0.00460144	0.0084421	1.9518	0.099868	rs505404	1.33E-35	0	1.05E-94	0	Thyroid
ENSG00000151327	FAM177A1	14	35514113	35582336	ENSG00000157017	GHRL	3	10328388	10334631	0.01181	5.2431	2.83E-08	0.00480107	0.063186	5.5798	1.06E-08	rs2143950	2.92E-22	0	1.11E-25	0	Adrenal_Gland
ENSG00000151327	FAM177A1	14	35514113	35582336	ENSG00000157017	GHRL	3	10328388	10334631	0.01181	5.2431	2.83E-08	0.00480107	0.063186	5.5798	1.06E-08	rs2143950	2.92E-22	0	1.11E-25	0	Skin_Sun_Exposed_Lower_leg
ENSG00000168374	ARF4	3	57557090	57583947	ENSG00000158457	TSPAN33	7	128784712	128808637	0.0089738	8.7508	2.83E-08	0.0048066	0.0040383	0.74281	0.59149	rs17825630	2.42E-15	0	NA	NA	Artery_Tibial
ENSG00000168374	ARF4	3	57557090	57583947	ENSG00000158457	TSPAN33	7	128784712	128808637	0.0089738	8.7508	2.83E-08	0.0048066	0.0040383	0.74281	0.59149	rs17825630	2.42E-15	0	NA	NA	Heart_Atrial_Appendage
ENSG00000163946	FAM208A	3	56658507	56717265	ENSG00000148498	PARD3	10	34398488	35104253	0.010451	6.3753	2.91E-08	0.00492052	0.0077181	1.0156	0.41844	rs12485738	3.83E-11	0	NA	NA	Esophagus_Mucosa
ENSG00000163946	FAM208A	3	56658507	56717265	ENSG00000148498	PARD3	10	34398488	35104253	0.010451	6.3753	2.91E-08	0.00492052	0.0077181	1.0156	0.41844	rs1354034	9.52E-18	0	NA	NA	Heart_Atrial_Appendage
ENSG00000154760	SLFN13	17	33762115	33775856	ENSG00000154146	NRGN	11	124609829	124615878	0.011785	5.2322	2.97E-08	0.00501211	0.013775	1.058	0.39292	rs225245	1.78E-13	0	NA	NA	Liver
ENSG00000154760	SLFN13	17	33762115	33775856	ENSG00000154146	NRGN	11	124609829	124615878	0.011785	5.2322	2.97E-08	0.00501211	0.013775	1.058	0.39292	rs8082605	2.53E-38	0	2.08E-19	0	Artery_Tibial
ENSG00000154760	SLFN13	17	33762115	33775856	ENSG00000154146	NRGN	11	124609829	124615878	0.011785	5.2322	2.97E-08	0.00501211	0.013775	1.058	0.39292	rs8082605	2.53E-38	0	2.08E-19	0	Esophagus_Mucosa
ENSG00000154760	SLFN13	17	33762115	33775856	ENSG00000154146	NRGN	11	124609829	124615878	0.011785	5.2322	2.97E-08	0.00501211	0.013775	1.058	0.39292	rs8082605	2.53E-38	0	2.08E-19	0	Nerve_Tibial
ENSG00000154760	SLFN13	17	33762115	33775856	ENSG00000154146	NRGN	11	124609829	124615878	0.011785	5.2322	2.97E-08	0.00501211	0.013775	1.058	0.39292	rs8082605	2.53E-38	0	2.08E-19	0	Pituitary
ENSG00000154760	SLFN13	17	33762115	33775856	ENSG00000154146	NRGN	11	124609829	124615878	0.011785	5.2322	2.97E-08	0.00501211	0.013775	1.058	0.39292	rs10512472	4.87E-94	0	6.34E-38	0	Uterus
ENSG00000006125	AP2B1	17	33913918	34053436	ENSG00000159335	PTMS	12	6874682	6880116	0.0099578	6.94	3.03E-08	0.00508996	0.016514	2.5606	0.018263	rs10512472	3.56E-10	2.15E-05	3.62E-14	0	Heart_Left_Ventricle
ENSG00000099849	RASSF7	11	560404	564021	ENSG00000173210	ABLIM3	5	148521046	148640105	0.0099541	6.9374	3.05E-08	0.0051184	0.0092076	1.2134	0.29235	rs505404	3.10E-39	0	NA	NA	Brain_Frontal_Cortex_BA9
ENSG00000154760	SLFN13	17	33762115	33775856	ENSG00000168785	TSPAN5	4	99391518	99579780	0.01177	5.2251	3.07E-08	0.00514439	0.010476	0.80197	0.64877	rs225245	5.57E-19	0	NA	NA	Liver
ENSG00000154760	SLFN13	17	33762115	33775856	ENSG00000168785	TSPAN5	4	99391518	99579780	0.01177	5.2251	3.07E-08	0.00514439	0.010476	0.80197	0.64877	rs8082605	5.30E-09	0.00012019	2.08E-19	0	Artery_Tibial
ENSG00000154760	SLFN13	17	33762115	33775856	ENSG00000168785	TSPAN5	4	99391518	99579780	0.01177	5.2251	3.07E-08	0.00514439	0.010476	0.80197	0.64877	rs8082605	5.30E-09	0.00012019	2.08E-19	0	Esophagus_Mucosa
ENSG00000154760	SLFN13	17	33762115	33775856	ENSG00000168785	TSPAN5	4	99391518	99579780	0.01177	5.2251	3.07E-08	0.00514439	0.010476	0.80197	0.64877	rs8082605	5.30E-09	0.00012019	2.08E-19	0	Nerve_Tibial
ENSG00000154760	SLFN13	17	33762115	33775856	ENSG00000168785	TSPAN5	4	99391518	99579780	0.01177	5.2251	3.07E-08	0.00514439	0.010476	0.80197	0.64877	rs8082605	5.30E-09	0.00012019	2.08E-19	0	Pituitary
ENSG00000115464	USP34	2	61416045	61697904	ENSG00000187017	ESPN	1	6484848	6521040	0.011328	5.5308	3.11E-08	0.00519025	0.081283	8.06	1.50E-12	rs766432	3.27170000000001e-	0	NA	NA	Artery_Aorta
ENSG00000115464	USP34	2	61416045	61697904	ENSG00000187017	ESPN	1	6484848	6521040	0.011328	5.5308	3.11E-08	0.00519025	0.081283	8.06	1.50E-12	rs4671393	3.27170000000001e-	0	NA	NA	Artery_Aorta
ENSG00000115464	USP34	2	61416045	61697904	ENSG00000187017	ESPN	1	6484848	6521040	0.011328	5.5308	3.11E-08	0.00519025	0.081283	8.06	1.50E-12	rs1427407	3.27170000000001e-	0	NA	NA	Pancreas
ENSG00000188895	MSL1	17	38278551	38291643	ENSG00000170525	PFKFB3	10	6186881	6277495	0.011731	5.208	3.32E-08	0.0055045	0.0063267	1.9483	0.12021	rs3894194	8.91E-09	0.00016464	NA	NA	Spleen
ENSG00000188895	MSL1	17	38278551	38291643	ENSG00000170525	PFKFB3	10	6186881	6277495	0.011731	5.208	3.32E-08	0.0055045	0.0063267	1.9483	0.12021	rs7212938	5.45E-08	0.00074524	NA	NA	Thyroid
ENSG00000188895	MSL1	17	38278551	38291643	ENSG00000170525	PFKFB3	10	6186881	6277495	0.011731	5.208	3.32E-08	0.0055045	0.0063267	1.9483	0.12021	rs8078723	5.30E-15	0	NA	NA	Cells_Transformed_fibroblasts
ENSG00000188895	MSL1	17	38278551	38291643	ENSG00000170525	PFKFB3	10	6186881	6277495	0.011731	5.208	3.32E-08	0.0055045	0.0063267	1.9483	0.12021	rs4794820	1.46E-09	5.76E-05	1.11E-05	0.031069922	Pancreas
ENSG00000163946	FAM208A	3	56658507	56717265	ENSG00000127920	GNG11	7	93551011	93555831	0.010384	6.3341	3.36E-08	0.00556426	0.030217	4.0684	0.00020908	rs12485738	3.89E-243	0	NA	NA	Esophagus_Mucosa
ENSG00000163946	FAM208A	3	56658507	56717265	ENSG00000127920	GNG11	7	93551011	93555831	0.010384	6.3341	3.36E-08	0.00556426	0.030217	4.0684	0.00020908	rs1354034	3.27170000000001e-	0	NA	NA	Heart_Atrial_Appendage
ENSG00000177951	BET1L	11	167784	207428	ENSG00000137801	THBS1	15	39873280	39891667	0.010379	6.3306	3.40E-08	0.00562737	0.0078392	0.65364	0.78291	rs11602954	2.10E-25	0	9.12E-206	0	Adipose_Visceral_Omentum
ENSG00000177951	BET1L	11	167784	207428	ENSG00000137801	THBS1	15	39873280	39891667	0.010379	6.3306	3.40E-08	0.00562737	0.0078392	0.65364	0.78291	rs11602954	2.10E-25	0	9.12E-206	0	Artery_Aorta
ENSG00000177951	BET1L	11	167784	207428	ENSG00000137801	THBS1	15	39873280	39891667	0.010379	6.3306	3.40E-08	0.00562737	0.0078392	0.65364	0.78291	rs11602954	2.10E-25	0	9.12E-206	0	Cells_Transformed_fibroblasts
ENSG00000177951	BET1L	11	167784	207428	ENSG00000137801	THBS1	15	39873280	39891667	0.010379	6.3306	3.40E-08	0.00562737	0.0078392	0.65364	0.78291	rs11602954	2.10E-25	0	9.12E-206	0	Pituitary
ENSG00000177951	BET1L	11	167784	207428	ENSG00000137801	THBS1	15	39873280	39891667	0.010379	6.3306	3.40E-08	0.00562737	0.0078392	0.65364	0.78291	rs11602954	2.10E-25	0	9.12E-206	0	Prostate
ENSG00000177951	BET1L	11	167784	207428	ENSG00000137801	THBS1	15	39873280	39891667	0.010379	6.3306	3.40E-08	0.00562737	0.0078392	0.65364	0.78291	rs11602954	2.10E-25	0	9.12E-206	0	Small_Intestine_Terminal_Ileum
ENSG00000177951	BET1L	11	167784	207428	ENSG00000137801	THBS1	15	39873280	39891667	0.010379	6.3306	3.40E-08	0.00562737	0.0078392	0.65364	0.78291	rs11602954	2.10E-25	0	9.12E-206	0	Whole_Blood
ENSG00000177951	BET1L	11	167784	207428	ENSG00000137801	THBS1	15	39873280	39891667	0.010379	6.3306	3.40E-08	0.00562737	0.0078392	0.65364	0.78291	rs17655730	3.05E-29	0	2.96E-166	0	Prostate
ENSG00000177951	BET1L	11	167784	207428	ENSG00000137801	THBS1	15	39873280	39891667	0.010379	6.3306	3.40E-08	0.00562737	0.0078392	0.65364	0.78291	rs505404	9.69E-32	0	3.73E-187	0	Small_Intestine_Terminal_Ileum
ENSG00000177951	BET1L	11	167784	207428	ENSG00000137801	THBS1	15	39873280	39891667	0.010379	6.3306	3.40E-08	0.00562737	0.0078392	0.65364	0.78291	rs505404	9.69E-32	0	3.73E-187	0	Whole_Blood
ENSG00000174885	NLRP6	11	278365	285359	ENSG00000102362	SYTL4	X	99929488	99987110	0.009899	6.8986	3.44E-08	0.0056846	0.022767	2.3608	0.012283	rs505404	4.66E-06	0.03095987	2.65E-88	0	Nerve_Tibial
ENSG00000174885	NLRP6	11	278365	285359	ENSG00000102362	SYTL4	X	99929488	99987110	0.009899	6.8986	3.44E-08	0.0056846	0.022767	2.3608	0.012283	rs505404	4.66E-06	0.03095987	2.65E-88	0	Prostate

ENSG00000167914	GSDMA	17	38119226	38134019	ENSG00000170525	PFKFB3	10	6186881	6277495	0.0070819	17.243	3.45E-08	0.00569343	0.010365	4.8128	0.0083314	rs3859192	5.54E-15	0	6.22E-13	0	Adipose_Subcutaneous
ENSG00000167914	GSDMA	17	38119226	38134019	ENSG00000170525	PFKFB3	10	6186881	6277495	0.0070819	17.243	3.45E-08	0.00569343	0.010365	4.8128	0.0083314	rs3859192	5.54E-15	0	6.22E-13	0	Adipose_Visceral_Omentum
ENSG00000167914	GSDMA	17	38119226	38134019	ENSG00000170525	PFKFB3	10	6186881	6277495	0.0070819	17.243	3.45E-08	0.00569343	0.010365	4.8128	0.0083314	rs3859192	5.54E-15	0	6.22E-13	0	Adrenal_Gland
ENSG00000167914	GSDMA	17	38119226	38134019	ENSG00000170525	PFKFB3	10	6186881	6277495	0.0070819	17.243	3.45E-08	0.00569343	0.010365	4.8128	0.0083314	rs3859192	5.54E-15	0	6.22E-13	0	Artery_Aorta
ENSG00000167914	GSDMA	17	38119226	38134019	ENSG00000170525	PFKFB3	10	6186881	6277495	0.0070819	17.243	3.45E-08	0.00569343	0.010365	4.8128	0.0083314	rs3859192	5.54E-15	0	6.22E-13	0	Artery_Coronary
ENSG00000167914	GSDMA	17	38119226	38134019	ENSG00000170525	PFKFB3	10	6186881	6277495	0.0070819	17.243	3.45E-08	0.00569343	0.010365	4.8128	0.0083314	rs3859192	5.54E-15	0	6.22E-13	0	Artery_Tibial
ENSG00000167914	GSDMA	17	38119226	38134019	ENSG00000170525	PFKFB3	10	6186881	6277495	0.0070819	17.243	3.45E-08	0.00569343	0.010365	4.8128	0.0083314	rs3859192	5.54E-15	0	6.22E-13	0	Breast_Mammary_Tissue
ENSG00000167914	GSDMA	17	38119226	38134019	ENSG00000170525	PFKFB3	10	6186881	6277495	0.0070819	17.243	3.45E-08	0.00569343	0.010365	4.8128	0.0083314	rs3859192	5.54E-15	0	6.22E-13	0	Cells_Transformed_fibroblasts
ENSG00000167914	GSDMA	17	38119226	38134019	ENSG00000170525	PFKFB3	10	6186881	6277495	0.0070819	17.243	3.45E-08	0.00569343	0.010365	4.8128	0.0083314	rs3859192	5.54E-15	0	6.22E-13	0	Colon_Sigmoid
ENSG00000167914	GSDMA	17	38119226	38134019	ENSG00000170525	PFKFB3	10	6186881	6277495	0.0070819	17.243	3.45E-08	0.00569343	0.010365	4.8128	0.0083314	rs3859192	5.54E-15	0	6.22E-13	0	Colon_Transverse
ENSG00000167914	GSDMA	17	38119226	38134019	ENSG00000170525	PFKFB3	10	6186881	6277495	0.0070819	17.243	3.45E-08	0.00569343	0.010365	4.8128	0.0083314	rs3859192	5.54E-15	0	6.22E-13	0	Esophagus_Gastroesophageal_Junction
ENSG00000167914	GSDMA	17	38119226	38134019	ENSG00000170525	PFKFB3	10	6186881	6277495	0.0070819	17.243	3.45E-08	0.00569343	0.010365	4.8128	0.0083314	rs3859192	5.54E-15	0	6.22E-13	0	Esophagus_Muscularis
ENSG00000167914	GSDMA	17	38119226	38134019	ENSG00000170525	PFKFB3	10	6186881	6277495	0.0070819	17.243	3.45E-08	0.00569343	0.010365	4.8128	0.0083314	rs3859192	5.54E-15	0	6.22E-13	0	Heart_Atrial_Appendage
ENSG00000167914	GSDMA	17	38119226	38134019	ENSG00000170525	PFKFB3	10	6186881	6277495	0.0070819	17.243	3.45E-08	0.00569343	0.010365	4.8128	0.0083314	rs3859192	5.54E-15	0	6.22E-13	0	Heart_Left_Ventricle
ENSG00000167914	GSDMA	17	38119226	38134019	ENSG00000170525	PFKFB3	10	6186881	6277495	0.0070819	17.243	3.45E-08	0.00569343	0.010365	4.8128	0.0083314	rs3859192	5.54E-15	0	6.22E-13	0	Liver
ENSG00000167914	GSDMA	17	38119226	38134019	ENSG00000170525	PFKFB3	10	6186881	6277495	0.0070819	17.243	3.45E-08	0.00569343	0.010365	4.8128	0.0083314	rs3859192	5.54E-15	0	6.22E-13	0	Lung
ENSG00000167914	GSDMA	17	38119226	38134019	ENSG00000170525	PFKFB3	10	6186881	6277495	0.0070819	17.243	3.45E-08	0.00569343	0.010365	4.8128	0.0083314	rs3859192	5.54E-15	0	6.22E-13	0	Muscle_Skeletal
ENSG00000167914	GSDMA	17	38119226	38134019	ENSG00000170525	PFKFB3	10	6186881	6277495	0.0070819	17.243	3.45E-08	0.00569343	0.010365	4.8128	0.0083314	rs3859192	5.54E-15	0	6.22E-13	0	Nerve_Tibial
ENSG00000167914	GSDMA	17	38119226	38134019	ENSG00000170525	PFKFB3	10	6186881	6277495	0.0070819	17.243	3.45E-08	0.00569343	0.010365	4.8128	0.0083314	rs3859192	5.54E-15	0	6.22E-13	0	Ovary
ENSG00000167914	GSDMA	17	38119226	38134019	ENSG00000170525	PFKFB3	10	6186881	6277495	0.0070819	17.243	3.45E-08	0.00569343	0.010365	4.8128	0.0083314	rs3859192	5.54E-15	0	6.22E-13	0	Pancreas
ENSG00000167914	GSDMA	17	38119226	38134019	ENSG00000170525	PFKFB3	10	6186881	6277495	0.0070819	17.243	3.45E-08	0.00569343	0.010365	4.8128	0.0083314	rs3859192	5.54E-15	0	6.22E-13	0	Small_Intestine_Terminal_Ileum
ENSG00000167914	GSDMA	17	38119226	38134019	ENSG00000170525	PFKFB3	10	6186881	6277495	0.0070819	17.243	3.45E-08	0.00569343	0.010365	4.8128	0.0083314	rs3859192	5.54E-15	0	6.22E-13	0	Spleen
ENSG00000167914	GSDMA	17	38119226	38134019	ENSG00000170525	PFKFB3	10	6186881	6277495	0.0070819	17.243	3.45E-08	0.00569343	0.010365	4.8128	0.0083314	rs3859192	5.54E-15	0	6.22E-13	0	Stomach
ENSG00000167914	GSDMA	17	38119226	38134019	ENSG00000170525	PFKFB3	10	6186881	6277495	0.0070819	17.243	3.45E-08	0.00569343	0.010365	4.8128	0.0083314	rs3859192	5.54E-15	0	6.22E-13	0	Thyroid
ENSG00000167914	GSDMA	17	38119226	38134019	ENSG00000170525	PFKFB3	10	6186881	6277495	0.0070819	17.243	3.45E-08	0.00569343	0.010365	4.8128	0.0083314	rs3859192	5.54E-15	0	6.22E-13	0	Whole_Blood
ENSG00000167914	GSDMA	17	38119226	38134019	ENSG00000170525	PFKFB3	10	6186881	6277495	0.0070819	17.243	3.45E-08	0.00569343	0.010365	4.8128	0.0083314	rs3894194	8.91E-09	0.00016464	6.08E-29	0	Adipose_Subcutaneous
ENSG00000167914	GSDMA	17	38119226	38134019	ENSG00000170525	PFKFB3	10	6186881	6277495	0.0070819	17.243	3.45E-08	0.00569343	0.010365	4.8128	0.0083314	rs3894194	8.91E-09	0.00016464	6.08E-29	0	Adipose_Visceral_Omentum
ENSG00000167914	GSDMA	17	38119226	38134019	ENSG00000170525	PFKFB3	10	6186881	6277495	0.0070819	17.243	3.45E-08	0.00569343	0.010365	4.8128	0.0083314	rs3894194	8.91E-09	0.00016464	6.08E-29	0	Artery_Tibial
ENSG00000167914	GSDMA	17	38119226	38134019	ENSG00000170525	PFKFB3	10	6186881	6277495	0.0070819	17.243	3.45E-08	0.00569343	0.010365	4.8128	0.0083314	rs3894194	8.91E-09	0.00016464	6.08E-29	0	Colon_Transverse
ENSG00000167914	GSDMA	17	38119226	38134019	ENSG00000170525	PFKFB3	10	6186881	6277495	0.0070819	17.243	3.45E-08	0.00569343	0.010365	4.8128	0.0083314	rs3894194	8.91E-09	0.00016464	6.08E-29	0	Esophagus_Gastroesophageal_Junction
ENSG00000167914	GSDMA	17	38119226	38134019	ENSG00000170525	PFKFB3	10	6186881	6277495	0.0070819	17.243	3.45E-08	0.00569343	0.010365	4.8128	0.0083314	rs3894194	8.91E-09	0.00016464	6.08E-29	0	Heart_Atrial_Appendage
ENSG00000167914	GSDMA	17	38119226	38134019	ENSG00000170525	PFKFB3	10	6186881	6277495	0.0070819	17.243	3.45E-08	0.00569343	0.010365	4.8128	0.0083314	rs3894194	8.91E-09	0.00016464	6.08E-29	0	Muscle_Skeletal
ENSG00000167914	GSDMA	17	38119226	38134019	ENSG00000170525	PFKFB3	10	6186881	6277495	0.0070819	17.243	3.45E-08	0.00569343	0.010365	4.8128	0.0083314	rs3894194	8.91E-09	0.00016464	6.08E-29	0	Whole_Blood
ENSG00000167914	GSDMA	17	38119226	38134019	ENSG00000170525	PFKFB3	10	6186881	6277495	0.0070819	17.243	3.45E-08	0.00569343	0.010365	4.8128	0.0083314	rs7212938	5.45E-08	0.00074524	5.34E-44	0	Adipose_Subcutaneous
ENSG00000167914	GSDMA	17	38119226	38134019	ENSG00000170525	PFKFB3	10	6186881	6277495	0.0070819	17.243	3.45E-08	0.00569343	0.010365	4.8128	0.0083314	rs7212938	5.45E-08	0.00074524	5.34E-44	0	Adipose_Visceral_Omentum
ENSG00000167914	GSDMA	17	38119226	38134019	ENSG00000170525	PFKFB3	10	6186881	6277495	0.0070819	17.243	3.45E-08	0.00569343	0.010365	4.8128	0.0083314	rs7212938	5.45E-08	0.00074524	5.34E-44	0	Artery_Coronary
ENSG00000167914	GSDMA	17	38119226	38134019	ENSG00000170525	PFKFB3	10	6186881	6277495	0.0070819	17.243	3.45E-08	0.00569343	0.010365	4.8128	0.0083314	rs7212938	5.45E-08	0.00074524	5.34E-44	0	Colon_Sigmoid
ENSG00000167914	GSDMA	17	38119226	38134019	ENSG00000170525	PFKFB3	10	6186881	6277495	0.0070819	17.243	3.45E-08	0.00569343	0.010365	4.8128	0.0083314	rs7212938	5.45E-08	0.00074524	5.34E-44	0	Heart_Atrial_Appendage
ENSG00000167914	GSDMA	17	38119226	38134019	ENSG00000170525	PFKFB3	10	6186881	6277495	0.0070819	17.243	3.45E-08	0.00569343	0.010365	4.8128	0.0083314	rs7212938	5.45E-08	0.00074524	5.34E-44	0	Heart_Left_Ventricle
ENSG00000167914	GSDMA	17	38119226	38134019	ENSG00000170525	PFKFB3	10	6186881	6277495	0.0070819	17.243	3.45E-08	0.00569343	0.010365	4.8128	0.0083314	rs7212938	5.45E-08	0.00074524	5.34E-44	0	Thyroid
ENSG00000167914	GSDMA	17	38119226	38134019	ENSG00000170525	PFKFB3	10	6186881	6277495	0.0070819	17.243	3.45E-08	0.00569343	0.010365	4.8128	0.0083314	rs7212938	5.45E-08	0.00074524	5.34E-44	0	Whole_Blood
ENSG00000167914	GSDMA	17	38119226	38134019	ENSG00000170525	PFKFB3	10	6186881	6277495	0.0070819	17.243	3.45E-08	0.00569343	0.010365	4.8128	0.0083314	rs8078723	5.30E-15	0	6.97E-07	0.002194627	Breast_Mammary_Tissue
ENSG00000167914	GSDMA	17	38119226	38134019	ENSG00000170525	PFKFB3	10	6186881	6277495	0.0070819	17.243	3.45E-08	0.00569343	0.010365	4.8128	0.0083314	rs8078723	5.30E-15	0	6.97E-07	0.002194627	Esophagus_Gastroesophageal_Junction
ENSG00000167914	GSDMA	17	38119226	38134019	ENSG00000170525	PFKFB3	10	6186881	6277495	0.0070819	17.243	3.45E-08	0.00569343	0.010365	4.8128	0.0083314	rs4794820	1.46E-09	5.76E-05	2.26E-66	0	Artery_Tibial
ENSG00000167914	GSDMA	17	38119226	38134019	ENSG00000170525	PFKFB3	10	6186881	6277495	0.0070819	17.243	3.45E-08	0.00569343	0.010365	4.8128	0.0083314	rs4794820	1.46E-09	5.76E-05	2.26E-66	0	Cells_Transformed_fibroblasts
ENSG00000167914	GSDMA	17	38119226	38134019	ENSG00000170525	PFKFB3	10	6186881	6277495	0.0070819	17.243	3.45E-08	0.00569343	0.010365	4.8128	0.0083314	rs4794822	7.48E-14	0	3.02E-06	0.008756123	Breast_Mammary_Tissue
ENSG00000167914	GSDMA	17	38119226	38134019	ENSG00000170525	PFKFB3	10	6186881	6277495	0.0070819	17.243	3.45E-08	0.00569343	0.010365	4.8128	0.0083314	rs4794822	7.48E-14	0	3.02E-06	0.008756123	Cells_Transformed_fibroblasts
ENSG00000167914	GSDMA	17	38119226	38134019	ENSG00000170525	PFKFB3	10	6186881	6277495	0.0070819	17.243	3.45E-08	0.00569343	0.010365	4.8128	0.0083314	rs4794822	7.48E-14	0	3.02E-06	0.008756123	Esophagus_Gastroesophageal_Junction
ENSG00000167914	GSDMA	17	38119226	38134019	ENSG00000170525	PFKFB3	10	6186881	6277495	0.0070819	17.243	3.45E-08	0.00569343	0.010365	4.8128	0.0083314	rs4794822	7.48E-14	0	3.02E-06	0.008756123	Esophagus_Muscularis
ENSG00000167914	GSDMA	17	38119226	38134019	ENSG00000170525	PFKFB3	10	6186881	6277495	0.0070819	17.243	3.45E-08	0.00569343	0.010365	4.8128	0.0083314	rs4065321	1.52E-11	0	2.52E-12	0	Pancreas
ENSG00000008838	MED24	17	38175350	38217468	ENSG00000170525	PFKFB3	10	6186881	6277495	0.0083354	10.156	3.47E-08	0.0057223	0.01195	2.7728	0.026157	rs12946510	2.03E-07	0.00201119	NA	NA	Prostate
ENSG00000008838	MED24	17	38175350	38217468	ENSG00000170525	PFKFB3	10	6186881	6277495	0.0083354	10.156	3.47E-08	0.0057223	0.01195	2.7728	0.026157	rs3859192	5.54E-15	0	1.47E-40	0	Whole_Blood
ENSG00000008838	MED24	17	38175350	38217468	ENSG00000170525	PFKFB3	10	6186881	6277495	0.0083354	10.156	3.47E-08	0.0057223	0.01195	2.7728	0.026157	rs3894194	8.91E-09	0.00016464	8.18E-15	0	Thyroid
ENSG00000008838	MED24	17	38175350	38217468	ENSG00000170525	PFKFB3	10	6186881	6277495	0.0083354	10.156	3.47E-08	0.0057223	0.01195	2.7728	0.026157	rs7212938	5.45E-08	0.00074524	4.22E-10	0	Testis
ENSG00000008838	MED24	17	38175350	38217468	ENSG00000170525	PFKFB3	10	6186881	6277495	0.0083354	10.156	3.47E-08	0.0057223	0.01195	2.7728	0.026157	rs7212938	5.45E-08	0.00074524	4.22E-10	0	Thyroid
ENSG00000008838	MED24	17	38175350	38217468	ENSG00000170525	PFKFB3	10	6186881	6277495	0.0083354	10.156	3.47E-08	0.0057223	0.01195	2.7728	0.026157	rs8078723	5.30E-15	0	5.30E-78	0	Adipose_Subcutaneous
ENSG00000008838	MED24	17	38175350	38217468	ENSG00000170525	PFKFB3	10	6186881	6277495	0.0083354	10.156	3.47E-08	0.0057223	0.01195	2.7728	0.026157	rs8078723	5.30E-15	0	5.30E-78	0	Artery_Tibial
ENSG00000008838	MED24	17	38175350	38217468	ENSG00000170525	PFKFB3	10	6186881	6277495	0.0083354	10.156	3.47E-08	0.0057223	0.01195	2.7728	0.026157	rs8078723	5.30E-15	0	5.30E-78	0	Cells_Transformed_fibroblasts
ENSG00000008838	MED24	17	38175350	38217468	ENSG00000170525	PFKFB3	10	6186881	6277495	0.0083354	10.156	3.47E-08	0.0057223	0.01195	2.7728	0.026157	rs8078723	5.30E-15	0	5.30E-78	0	Lung
ENSG00000008838	MED24	17	38175350	38217468	ENSG00000170525	PFKFB3	10	6186881	6277495	0.0083354	10.156	3.47E-08	0.0057223	0.01195	2.7728	0.026157	rs8078723	5.30E-15	0	5.30E-78	0	Pituitary
ENSG00000008838	MED24	17	38175350	38217468	ENSG00000170525	PFKFB3	10	6186881	6277495	0.0083354	10.156	3.47E-08	0.0057223	0.01195	2.7728	0.026157	rs8078723	5.30E-15	0	5.30E-78	0	Skin_Sun_Exposed_Lower_leg
ENSG00000008838	MED24	17	38175350	38217468	ENSG00000170525	PFKFB3	10	6186881	6277495	0.0083354	10.156	3.47E-08	0.0057223	0.01195	2.7728	0.026157	rs8078723	5.30E-15	0	5.30E-78	0	Spleen
ENSG00000008838	MED24	17	38175350	38217468	ENSG00000170525	PFKFB3	10	6186881	6277495	0.0083354	10.156	3.47E-08	0.0057223	0.01195	2.7728	0.026157	rs8078723	5.30E-15	0	5.30E-78	0	Stomach
ENSG00000008838	MED24	17	38175350	38217468	ENSG00000170525	PFKFB3	10	6186881	6277495	0.0083354	10.156	3.47E-08	0.0057223	0.01195	2.7728	0.026157	rs8078723	5.30E-15	0	5.30E-78	0	Thyroid
ENSG00000008838	MED24	17	38175350	38217468	ENSG00000170525	PFKFB3	10	6186881	6277495	0.0083354	10.156	3.47E-08	0.0057223	0.01195	2.7728	0.026157	rs4794822	7.48E-14	0	1.32E-79	0	Esophagus_Gastroesophageal_Junction
ENSG00000008838	MED24	17	38175350	38217468	ENSG00000170525	PFKFB3	10	6186881	6277495	0.0083354	10.156	3.47E-08	0.0057223	0.01195	2.7728	0.026157	rs4794822	7.48E-14	0	1.32E-79	0	Lung
ENSG00000008838	MED24	17	38175350	38217468	ENSG00000170525	PFKFB3	10	6186881	6277495	0.0083354	10.156	3.47E-08	0.0057223	0.01195	2.7728	0.026157	rs4794822	7.48E-14	0	1.32E-79	0	Spleen
ENSG00000008838	MED24	17	38175350	38217468	ENSG00000170525	PFKFB3	10	6186881	6277495	0.0083354	10.156	3.47E-08	0.0057223	0.01195	2.7728	0.026157	rs4794822	7.48E-14	0	1.32E-79	0	Whole_Blood
ENSG00000008838	MED24	17	38175350	38217468	ENSG00000170525	PFKFB3	10	6186881	6277495	0.0083354	10.156	3.47E-08	0.0057223	0.01195	2.7728	0.026157	rs4065321	1.52E-11	0	1.24E-42	0	Pituitary
ENSG00000008838	MED24	17	38175350	38217468	ENSG00000170525	PFKFB3	10	6186881	6277495	0.0083354	10.156	3.47E-08	0.0057223	0.01195	2.7728	0.026157	rs8067378	2.67E-07	0.00257357	NA	NA	Brain_Hypothalamus
ENSG00000168374	ARF4	3	57557090	57583947	ENSG00000205038	PKHD1L1	8	110374706	110542559	0.0088769	8.6554	3.53E-08	0.00580637	0.02411	4.5262	0.00044089	rs17825630	2.04E-06	0.0156941	NA	NA	Artery_Tibial
ENSG00000168374	ARF4	3	57557090	57583947	ENSG00000205038	PKHD1L1	8	110374706	110542559	0.0088769	8.6554	3.53E-08	0.00580637	0.02411	4.5262	0.00044089	rs17825630	2.04E-06	0.0156941	NA	NA	Heart_Atrial_Appendage
ENSG00000132139	GAS2L2	17	34071530	34079897	ENSG00000166947	EPB42	15	43398423	43513481	0.010339	6.3059	3.71E-08	0.0060483	0.0083231	0.95784	0.46779	rs9915021	3.48E-18	0	NA	NA	Small_Intestine_Terminal_Ileum
ENSG00000205045	SLFN12L	17	33800708	33864880	ENSG00000135924	DNAJB2	2	220143989	220151622	0.0098564	6.8686	3.78E-08	0.00614329	0.0088541	1.1664	0.31947	rs8082605	2.06E-11	0	2.49E-31	0	Adipose_Subcutaneous
ENSG00000205045	SLFN12L	17	33800708	33864880	ENSG00000135924	DNAJB2	2	220143989	220151622	0.0098564	6.8686	3.78E-08	0.00614329	0.0088541	1.1664	0.31947	rs8082605	2.06E-11	0	2.49E-31	0	Adipose_Visceral_Omentum
ENSG00000205045	SLFN12L	17	33800708	33864880	ENSG00000135924	DNAJB2	2	220143989	220151622	0.0098564	6.8686	3.78E-08	0.00614329	0.0088541	1.1664	0.31947	rs8082605	2.06E-11	0	2.49E-31	0	Heart_Left_Ventricle
ENSG00000205045	SLFN12L	17	33800708	33864880	ENSG00000135924	DNAJB2	2	220143989	220151622	0.0098564	6.8686	3.78E-08	0.00614329	0.0088541	1.1664	0.31947	rs8082605	2.06E-11	0	2.49E-31	0	Nerve_Tibial
ENSG00000205045	SLFN12L	17	33800708	33864880	ENSG00000135924	DNAJB2	2	220143989	220151622	0.0098564	6.8686	3.78E-08	0.00614329	0.0088541	1.1664	0.31947	rs10512472	6.15E-09	0.00013543	4.96E-30	0	Pancreas
ENSG00000174885	NLRP6	11	278365	285359	ENSG00000138798	EGF	4	110834040	110933422	0.0098448	6.8604	3.88E-08	0.00628166	0.0082151	0.83936	0.57985	rs11602954	3.92E-10	2.13E-05	2.12E-70	0	Nerve_Tibial
ENSG00000174885	NLRP6	11	278365	285359	ENSG00000138798	EGF	4	110834040	110933422	0.0098448	6.8604	3.88E-08	0.00628166	0.0082151	0.83936	0.57985	rs17655730	3.08E-11	0	5.43E-117	0	Artery_Aorta
ENSG00000174885	NLRP6	11	278365	285359	ENSG00000138798	EGF	4	110834040	110933422	0.0098448	6.8604	3.88E-08	0.00628166	0.0082151	0.83936	0.57985	rs17655730	3.08E-11	0	5.43E-117	0	Nerve_Tibial
ENSG00000174885	NLRP6	11	278365	285359	ENSG00000138798	EGF	4	110834040	110933422	0.0098448	6.8604	3.88E-08	0.00628166	0.0082151	0.83936	0.57985	rs17655730	3.08E-11	0	5.43E-117	0	Skin_Sun_Exposed_Lower_leg

ENSG00000174885	NLRP6	11	278365	285359	ENSG00000138798	EGF	4	110834040	110933422	0.0098448	6.8604	3.88E-08	0.00628166	0.0082151	0.83936	0.57985	rs505404	2.92E-13	0	2.65E-88	0	Nerve_Tibial
ENSG00000174885	NLRP6	11	278365	285359	ENSG00000138798	EGF	4	110834040	110933422	0.0098448	6.8604	3.88E-08	0.00628166	0.0082151	0.83936	0.57985	rs505404	2.92E-13	0	2.65E-88	0	Prostate
ENSG00000062038	CDH3	16	68670092	68756519	ENSG00000255833	TIFAB	5	134787899	134788089	0.0093474	7.5973	3.92E-08	0.00633178	0.0084909	1.1182	0.34918	rs1364063	1.89E-12	0	NA	NA	Ovary
ENSG00000172123	SLFN12	17	33738079	33760302	ENSG00000138867	GUCD1	22	24936406	24951284	0.013293	4.3309	3.99E-08	0.00642594	NA	NA	NA	rs225245	4.32E-38	0	NA	NA	Cells_EBV-transformed_lymphocytes
ENSG00000172123	SLFN12	17	33738079	33760302	ENSG00000138867	GUCD1	22	24936406	24951284	0.013293	4.3309	3.99E-08	0.00642594	NA	NA	NA	rs9915021	1.78E-10	0	NA	NA	Breast_Mammary_Tissue
ENSG00000108733	PEX12	17	33901814	33905882	ENSG00000011105	TSPAN9	12	3186521	3395730	0.0088198	8.5993	4.02E-08	0.00645623	0.049826	9.6067	5.97E-09	rs9915021	7.16E-59	0	NA	NA	Brain_Caudate_basal_ganglia
ENSG00000108733	PEX12	17	33901814	33905882	ENSG00000011105	TSPAN9	12	3186521	3395730	0.0088198	8.5993	4.02E-08	0.00645623	0.049826	9.6067	5.97E-09	rs9915021	7.16E-59	0	NA	NA	Breast_Mammary_Tissue
ENSG00000108733	PEX12	17	33901814	33905882	ENSG00000011105	TSPAN9	12	3186521	3395730	0.0088198	8.5993	4.02E-08	0.00645623	0.049826	9.6067	5.97E-09	rs10512472	5.91E-134	0	1.06E-12	0	Brain_Caudate_basal_ganglia
ENSG00000135535	CD164	6	109687717	109703762	ENSG00000134755	DSC2	18	28645940	28682378	0.010759	5.8344	4.03E-08	0.00646738	0.011696	1.3506	0.2147	rs1008084	3.03E-27	0	1.62E-80	0	Brain_Anterior_cingulate_cortex_BA24
ENSG00000135535	CD164	6	109687717	109703762	ENSG00000134755	DSC2	18	28645940	28682378	0.010759	5.8344	4.03E-08	0.00646738	0.011696	1.3506	0.2147	rs1008084	3.03E-27	0	1.62E-80	0	Nerve_Tibial
ENSG00000135535	CD164	6	109687717	109703762	ENSG00000134755	DSC2	18	28645940	28682378	0.010759	5.8344	4.03E-08	0.00646738	0.011696	1.3506	0.2147	rs884366	2.14E-19	0	5.36E-100	0	Muscle_Skeletal
ENSG00000135535	CD164	6	109687717	109703762	ENSG00000134755	DSC2	18	28645940	28682378	0.010759	5.8344	4.03E-08	0.00646738	0.011696	1.3506	0.2147	rs9374080	2.07E-28	0	2.82E-83	0	Whole_Blood
ENSG00000172057	ORMDL3	17	38077294	38083854	ENSG00000077238	IL4R	16	27324989	27376099	0.0088153	8.5948	4.06E-08	0.00650758	0.015228	2.3581	0.028851	rs7216389	2.24E-13	0	3.2717e-310	0	Adipose_Subcutaneous
ENSG00000172057	ORMDL3	17	38077294	38083854	ENSG00000077238	IL4R	16	27324989	27376099	0.0088153	8.5948	4.06E-08	0.00650758	0.015228	2.3581	0.028851	rs4794820	2.61E-09	7.33E-05	3.2717e-310	0	Spleen
ENSG00000172057	ORMDL3	17	38077294	38083854	ENSG00000077238	IL4R	16	27324989	27376099	0.0088153	8.5948	4.06E-08	0.00650758	0.015228	2.3581	0.028851	rs2872507	8.15E-10	2.02E-05	3.2717e-310	0	Whole_Blood
ENSG00000172057	ORMDL3	17	38077294	38083854	ENSG00000077238	IL4R	16	27324989	27376099	0.0088153	8.5948	4.06E-08	0.00650758	0.015228	2.3581	0.028851	rs8067378	1.55E-13	0	3.2717e-310	0	Cells_EBV-transformed_lymphocytes
ENSG00000172057	ORMDL3	17	38077294	38083854	ENSG00000077238	IL4R	16	27324989	27376099	0.0088153	8.5948	4.06E-08	0.00650758	0.015228	2.3581	0.028851	rs8067378	1.55E-13	0	3.2717e-310	0	Spleen
ENSG00000172057	ORMDL3	17	38077294	38083854	ENSG00000077238	IL4R	16	27324989	27376099	0.0088153	8.5948	4.06E-08	0.00650758	0.015228	2.3581	0.028851	rs8067378	1.55E-13	0	3.2717e-310	0	Whole_Blood
ENSG00000172057	ORMDL3	17	38077294	38083854	ENSG00000077238	IL4R	16	27324989	27376099	0.0088153	8.5948	4.06E-08	0.00650758	0.015228	2.3581	0.028851	rs9303277	2.79E-13	0	3.2717e-310	0	Cells_EBV-transformed_lymphocytes
ENSG00000172057	ORMDL3	17	38077294	38083854	ENSG00000077238	IL4R	16	27324989	27376099	0.0088153	8.5948	4.06E-08	0.00650758	0.015228	2.3581	0.028851	rs9303277	2.79E-13	0	3.2717e-310	0	Skin_Sun_Exposed_Lower_leg
ENSG00000172057	ORMDL3	17	38077294	38083854	ENSG00000077238	IL4R	16	27324989	27376099	0.0088153	8.5948	4.06E-08	0.00650758	0.015228	2.3581	0.028851	rs8069176	1.14E-10	0	3.2717e-310	0	Whole_Blood
ENSG00000172057	ORMDL3	17	38077294	38083854	ENSG00000077238	IL4R	16	27324989	27376099	0.0088153	8.5948	4.06E-08	0.00650758	0.015228	2.3581	0.028851	rs2290400	7.33E-15	0	3.2717e-310	0	Cells_EBV-transformed_lymphocytes
ENSG00000172057	ORMDL3	17	38077294	38083854	ENSG00000077238	IL4R	16	27324989	27376099	0.0088153	8.5948	4.06E-08	0.00650758	0.015228	2.3581	0.028851	rs2290400	7.33E-15	0	3.2717e-310	0	Esophagus_Muscularis
ENSG00000172057	ORMDL3	17	38077294	38083854	ENSG00000077238	IL4R	16	27324989	27376099	0.0088153	8.5948	4.06E-08	0.00650758	0.015228	2.3581	0.028851	rs2290400	7.33E-15	0	3.2717e-310	0	Lung
ENSG00000172057	ORMDL3	17	38077294	38083854	ENSG00000077238	IL4R	16	27324989	27376099	0.0088153	8.5948	4.06E-08	0.00650758	0.015228	2.3581	0.028851	rs2290400	7.33E-15	0	3.2717e-310	0	Skin_Sun_Exposed_Lower_leg
ENSG00000172716	SLFN11	17	33677324	33700720	ENSG00000144567	FAM134A	2	220042939	220050201	0.012041	4.9004	4.21E-08	0.0067193	0.02339	1.8143	0.041853	rs225245	2.89E-60	0	5.77E-07	0.001884469	Cells_Transformed_fibroblasts
ENSG00000172716	SLFN11	17	33677324	33700720	ENSG00000144567	FAM134A	2	220042939	220050201	0.012041	4.9004	4.21E-08	0.0067193	0.02339	1.8143	0.041853	rs8082605	2.77E-35	0	NA	NA	Adrenal_Gland
ENSG00000141741	MIEN1	17	37885409	37886014	ENSG00000151726	ACSL1	4	185676749	185747972	0.0087836	8.5637	4.36E-08	0.00693096	0.007858	1.451	0.20352	rs7212938	1.56E-07	0.00161211	NA	NA	Testis
ENSG00000188076	SCGB1C1	11	193080	194573	ENSG00000049323	LTBP1	2	33172039	33624576	0.0061646	29.997	4.55E-08	0.00718997	0.0038493	3.5551	0.059678	rs11602954	4.93E-09	0.00012117	3.14E-203	0	Whole_Blood
ENSG00000188076	SCGB1C1	11	193080	194573	ENSG00000049323	LTBP1	2	33172039	33624576	0.0061646	29.997	4.55E-08	0.00718997	0.0038493	3.5551	0.059678	rs17655730	2.15E-13	0	1.60E-163	0	Whole_Blood
ENSG00000188076	SCGB1C1	11	193080	194573	ENSG00000049323	LTBP1	2	33172039	33624576	0.0061646	29.997	4.55E-08	0.00718997	0.0038493	3.5551	0.059678	rs505404	1.77E-15	0	5.81E-188	0	Whole_Blood
ENSG00000196260	SFTA2	6	30899130	30899952	ENSG00000169507	SLC38A11	2	165752696	165812035	0.0087618	8.5422	4.59E-08	0.00723648	0.0080368	1.4843	0.19232	rs2442719	1.12E-07	0.00126582	NA	NA	Whole_Blood
ENSG00000196260	SFTA2	6	30899130	30899952	ENSG00000169507	SLC38A11	2	165752696	165812035	0.0087618	8.5422	4.59E-08	0.00723648	0.0080368	1.4843	0.19232	rs2233956	9.05E-12	0	NA	NA	Esophagus_Mucosa
ENSG00000172057	ORMDL3	17	38077294	38083854	ENSG00000135842	FAM129A	1	184759858	184943682	0.0087583	8.5388	4.62E-08	0.00728568	0.0086324	1.3279	0.24177	rs7216389	8.34E-12	0	3.2717e-310	0	Adipose_Subcutaneous
ENSG00000172057	ORMDL3	17	38077294	38083854	ENSG00000135842	FAM129A	1	184759858	184943682	0.0087583	8.5388	4.62E-08	0.00728568	0.0086324	1.3279	0.24177	rs4794820	1.01E-16	0	3.2717e-310	0	Spleen
ENSG00000172057	ORMDL3	17	38077294	38083854	ENSG00000135842	FAM129A	1	184759858	184943682	0.0087583	8.5388	4.62E-08	0.00728568	0.0086324	1.3279	0.24177	rs2872507	9.46E-10	3.98E-05	3.2717e-310	0	Whole_Blood
ENSG00000172057	ORMDL3	17	38077294	38083854	ENSG00000135842	FAM129A	1	184759858	184943682	0.0087583	8.5388	4.62E-08	0.00728568	0.0086324	1.3279	0.24177	rs8067378	3.15E-09	0.00010811	3.2717e-310	0	Cells_EBV-transformed_lymphocytes
ENSG00000172057	ORMDL3	17	38077294	38083854	ENSG00000135842	FAM129A	1	184759858	184943682	0.0087583	8.5388	4.62E-08	0.00728568	0.0086324	1.3279	0.24177	rs8067378	3.15E-09	0.00010811	3.2717e-310	0	Spleen
ENSG00000172057	ORMDL3	17	38077294	38083854	ENSG00000135842	FAM129A	1	184759858	184943682	0.0087583	8.5388	4.62E-08	0.00728568	0.0086324	1.3279	0.24177	rs8067378	3.15E-09	0.00010811	3.2717e-310	0	Whole_Blood
ENSG00000172057	ORMDL3	17	38077294	38083854	ENSG00000135842	FAM129A	1	184759858	184943682	0.0087583	8.5388	4.62E-08	0.00728568	0.0086324	1.3279	0.24177	rs9303277	2.02E-08	0.00024439	3.2717e-310	0	Cells_EBV-transformed_lymphocytes
ENSG00000172057	ORMDL3	17	38077294	38083854	ENSG00000135842	FAM129A	1	184759858	184943682	0.0087583	8.5388	4.62E-08	0.00728568	0.0086324	1.3279	0.24177	rs9303277	2.02E-08	0.00024439	3.2717e-310	0	Skin_Sun_Exposed_Lower_leg
ENSG00000172057	ORMDL3	17	38077294	38083854	ENSG00000135842	FAM129A	1	184759858	184943682	0.0087583	8.5388	4.62E-08	0.00728568	0.0086324	1.3279	0.24177	rs8069176	4.90E-12	0	3.2717e-310	0	Whole_Blood
ENSG00000172057	ORMDL3	17	38077294	38083854	ENSG00000135842	FAM129A	1	184759858	184943682	0.0087583	8.5388	4.62E-08	0.00728568	0.0086324	1.3279	0.24177	rs2290400	3.51E-11	0	3.2717e-310	0	Cells_EBV-transformed_lymphocytes
ENSG00000172057	ORMDL3	17	38077294	38083854	ENSG00000135842	FAM129A	1	184759858	184943682	0.0087583	8.5388	4.62E-08	0.00728568	0.0086324	1.3279	0.24177	rs2290400	3.51E-11	0	3.2717e-310	0	Esophagus_Muscularis
ENSG00000172057	ORMDL3	17	38077294	38083854	ENSG00000135842	FAM129A	1	184759858	184943682	0.0087583	8.5388	4.62E-08	0.00728568	0.0086324	1.3279	0.24177	rs2290400	3.51E-11	0	3.2717e-310	0	Lung
ENSG00000172057	ORMDL3	17	38077294	38083854	ENSG00000135842	FAM129A	1	184759858	184943682	0.0087583	8.5388	4.62E-08	0.00728568	0.0086324	1.3279	0.24177	rs2290400	3.51E-11	0	3.2717e-310	0	Skin_Sun_Exposed_Lower_leg
ENSG00000172660	TAF15	17	34136459	34174246	ENSG00000133808	MICALCL	11	12297627	12380691	0.0092697	7.5335	4.66E-08	0.00731287	0.015529	2.4055	0.025947	rs9915021	8.15E-10	2.02E-05	NA	NA	Skin_Not_Sun_Exposed_Suprapubic
ENSG00000172660	TAF15	17	34136459	34174246	ENSG00000133816	MICAL2	11	12115543	12285332	0.0092697	7.5335	4.66E-08	0.00731287	0.013319	2.0585	0.055694	rs9915021	1.31E-21	0	NA	NA	Skin_Not_Sun_Exposed_Suprapubic
ENSG00000177951	BET1L	11	167784	207428	ENSG00000128266	GNAZ	22	23412540	23464889	0.010226	6.2365	4.74E-08	0.00741983	0.020938	1.7691	0.055087	rs11602954	1.35E-18	0	9.12E-206	0	Adipose_Visceral_Omentum
ENSG00000177951	BET1L	11	167784	207428	ENSG00000128266	GNAZ	22	23412540	23464889	0.010226	6.2365	4.74E-08	0.00741983	0.020938	1.7691	0.055087	rs11602954	1.35E-18	0	9.12E-206	0	Artery_Aorta
ENSG00000177951	BET1L	11	167784	207428	ENSG00000128266	GNAZ	22	23412540	23464889	0.010226	6.2365	4.74E-08	0.00741983	0.020938	1.7691	0.055087	rs11602954	1.35E-18	0	9.12E-206	0	Cells_Transformed_fibroblasts
ENSG00000177951	BET1L	11	167784	207428	ENSG00000128266	GNAZ	22	23412540	23464889	0.010226	6.2365	4.74E-08	0.00741983	0.020938	1.7691	0.055087	rs11602954	1.35E-18	0	9.12E-206	0	Pituitary
ENSG00000177951	BET1L	11	167784	207428	ENSG00000128266	GNAZ	22	23412540	23464889	0.010226	6.2365	4.74E-08	0.00741983	0.020938	1.7691	0.055087	rs11602954	1.35E-18	0	9.12E-206	0	Prostate
ENSG00000177951	BET1L	11	167784	207428	ENSG00000128266	GNAZ	22	23412540	23464889	0.010226	6.2365	4.74E-08	0.00741983	0.020938	1.7691	0.055087	rs11602954	1.35E-18	0	9.12E-206	0	Small_Intestine_Terminal_Ileum
ENSG00000177951	BET1L	11	167784	207428	ENSG00000128266	GNAZ	22	23412540	23464889	0.010226	6.2365	4.74E-08	0.00741983	0.020938	1.7691	0.055087	rs11602954	1.35E-18	0	9.12E-206	0	Whole_Blood
ENSG00000177951	BET1L	11	167784	207428	ENSG00000128266	GNAZ	22	23412540	23464889	0.010226	6.2365	4.74E-08	0.00741983	0.020938	1.7691	0.055087	rs17655730	3.37E-11	0	2.96E-166	0	Prostate
ENSG00000177951	BET1L	11	167784	207428	ENSG00000128266	GNAZ	22	23412540	23464889	0.010226	6.2365	4.74E-08	0.00741983	0.020938	1.7691	0.055087	rs505404	8.89E-15	0	3.73E-187	0	Small_Intestine_Terminal_Ileum
ENSG00000177951	BET1L	11	167784	207428	ENSG00000128266	GNAZ	22	23412540	23464889	0.010226	6.2365	4.74E-08	0.00741983	0.020938	1.7691	0.055087	rs505404	8.89E-15	0	3.73E-187	0	Whole_Blood
ENSG00000172660	TAF15	17	34136459	34174246	ENSG00000136732	GYPC	2	127413509	127454246	0.0092587	7.5245	4.78E-08	0.00747726	0.013927	2.1539	0.045297	rs9915021	3.56E-10	2.15E-05	NA	NA	Skin_Not_Sun_Exposed_Suprapubic
ENSG00000205045	SLFN12L	17	33800708	33864880	ENSG00000186350	RXRA	9	137208944	137332431	0.0097419	6.788	4.86E-08	0.00759153	0.0094777	1.2494	0.2728	rs10512472	7.56E-08	0.00096373	4.96E-30	0	Pancreas
ENSG00000142082	SIRT3	11	215458	236431	ENSG00000124491	F13A1	6	6144318	6321246	0.0075785	12.305	5.13E-08	0.00796626	0.0075363	1.7408	0.13885	rs11602954	7.25E-48	0	1.39E-80	0	Adipose_Subcutaneous
ENSG00000142082	SIRT3	11	215458	236431	ENSG00000124491	F13A1	6	6144318	6321246	0.0075785	12.305	5.13E-08	0.00796626	0.0075363	1.7408	0.13885	rs11602954	7.25E-48	0	1.39E-80	0	Artery_Tibial
ENSG00000142082	SIRT3	11	215458	236431	ENSG00000124491	F13A1	6	6144318	6321246	0.0075785	12.305	5.13E-08	0.00796626	0.0075363	1.7408	0.13885	rs11602954	7.25E-48	0	1.39E-80	0	Breast_Mammary_Tissue
ENSG00000142082	SIRT3	11	215458	236431	ENSG00000124491	F13A1	6	6144318	6321246	0.0075785	12.305	5.13E-08	0.00796626	0.0075363	1.7408	0.13885	rs11602954	7.25E-48	0	1.39E-80	0	Cells_Transformed_fibroblasts
ENSG00000142082	SIRT3	11	215458	236431	ENSG00000124491	F13A1	6	6144318	6321246	0.0075785	12.305	5.13E-08	0.00796626	0.0075363	1.7408	0.13885	rs11602954	7.25E-48	0	1.39E-80	0	Nerve_Tibial
ENSG00000142082	SIRT3	11	215458	236431	ENSG00000124491	F13A1	6	6144318	6321246	0.0075785	12.305	5.13E-08	0.00796626	0.0075363	1.7408	0.13885	rs505404	5.93E-51	0	1.05E-94	0	Adrenal_Gland
ENSG00000142082	SIRT3	11	215458	236431	ENSG00000124491	F13A1	6	6144318	6321246	0.0075785	12.305	5.13E-08	0.00796626	0.0075363	1.7408	0.13885	rs505404	5.93E-51	0	1.05E-94	0	Artery_Aorta
ENSG00000142082	SIRT3	11	215458	236431	ENSG00000124491	F13A1	6	6144318	6321246	0.0075785	12.305	5.13E-08	0.00796626	0.0075363	1.7408	0.13885	rs505404	5.93E-51	0	1.05E-94	0	Lung
ENSG00000142082	SIRT3	11	215458	236431	ENSG00000124491	F13A1	6	6144318	6321246	0.0075785	12.305	5.13E-08	0.00796626	0.0075363	1.7408	0.13885	rs505404	5.93E-51	0	1.05E-94	0	Thyroid
ENSG00000142082	SIRT3	11	215458	236431	ENSG00000124491	F13A1	6	6144318	6321246	0.0075785	12.305	5.13E-08	0.00796626	0.0075363	1.7408	0.13885	rs3782123	5.12E-07	0.00442957	NA	NA	Brain_Frontal_Cortex_BA9
ENSG00000163946	FAM208A	3	56658507	56717265	ENSG00000005249	PRKAR2B	7	106685094	106802256	0.010184	6.2105	5.19E-08	0.00804662	0.023448	3.1352	0.0028091	rs12485738	2.38E-83	0	NA	NA	Esophagus_Mucosa
ENSG00000163946	FAM208A	3	56658507	56717265	ENSG00000005249	PRKAR2B	7	106685094	106802256	0.010184	6.2105	5.19E-08	0.00804662	0.023448	3.1352	0.0028091	rs1354034	2.97E-158	0	NA	NA	Heart_Atrial_Appendage
ENSG00000008838	MED24	17	38175350	38217468	ENSG00000151726	ACSL1	4	185676749	185747972	0.0081623	9.9433	5.19E-08	0.00804662	0.017086	3.9851	0.0032695	rs3859192	1.10E-13	0	1.47E-40	0	Whole_Blood
ENSG00000008838	MED24	17	38175350	38217468	ENSG00000151726	ACSL1	4	185676749	185747972	0.0081623	9.9433	5.19E-08	0.00804662	0.017086	3.9851	0.0032695	rs3894194	1.60E-06	0.0125406	8.18E-15	0	Thyroid
ENSG00000008838	MED24	17	38175350	38217468	ENSG00000151726	ACSL1	4	185676749	185747972	0.0081623	9.9433	5.19E-08	0.00804662	0.017086	3.9851	0.0032695	rs7212938	1.56E-07	0.00161211	4.22E-10	0	Testis
ENSG00000008838	MED24	17	38175350	38217468	ENSG00000151726	ACSL1	4	185676749	185747972	0.0081623	9.9433	5.19E-08	0.00804662	0.017086	3.9851	0.0032695	rs7212938	1.56E-07	0.00161211	4.22E-10	0	Thyroid
ENSG00000008838	MED24	17	38175350	38217468	ENSG00000151726	ACSL1	4	185676749	185747972	0.0081623	9.9433	5.19E-08	0.00804662	0.017086	3.9851	0.0032695	rs8078723	1.33E-13	0	5.30E-78	0	Adipose_Subcutaneous
ENSG00000008838	MED24	17	38175350	38217468	ENSG00000151726	ACSL1	4	185676749	185747972	0.0081623	9.9433	5.19E-08	0.00804662	0.017086	3.9851	0.0032695	rs8078723	1.33E-13	0	5.30E-78	0	Artery_Tibial
ENSG00000008838	MED24	17	38175350	38217468	ENSG00000151726	ACSL1	4	185676749	185747972	0.0081623	9.9433	5.19E-08	0.00804662	0.017086	3.9851	0.0032695	rs8078723	1.33E-13	0	5.30E-78	0	Cells_Transformed_fibroblasts
ENSG00000008838	MED24	17	38175350	38217468	ENSG00000151726	ACSL1	4	185676749	185747972	0.0081623	9.9433	5.19E-08	0.00804662	0.017086	3.9851	0.0032695	rs8078723	1.33E-13	0	5.30E-78	0	Lung
ENSG00000008838	MED24	17	38175350	38217468	ENSG00000151726	ACSL1	4	185676749	185747972	0.0081623	9.9433	5.19E-08	0.00804662	0.017086	3.9851	0.0032695	rs8078723	1.33E-13	0	5.30E-78	0	Pituitary
ENSG00000008838	MED24	17	38175350	38217468	ENSG00000151726	ACSL1	4	185676749	185747972	0.0081623	9.9433	5.19E-08	0.00804662	0.017086	3.9851	0.0032695	rs8078723	1.33E-13	0	5.30E-78	0	Skin_Sun_Exposed_Lower_leg

ENSG00000008838	MED24	17	38175350	38217468	ENSG00000151726	ACSL1	4	185676749	185747972	0.0081623	9.9433	5.19E-08	0.00804662	0.017086	3.9851	0.0032695	rs8078723	1.33E-13	0	5.30E-78	0	Spleen
ENSG00000008838	MED24	17	38175350	38217468	ENSG00000151726	ACSL1	4	185676749	185747972	0.0081623	9.9433	5.19E-08	0.00804662	0.017086	3.9851	0.0032695	rs8078723	1.33E-13	0	5.30E-78	0	Stomach
ENSG00000008838	MED24	17	38175350	38217468	ENSG00000151726	ACSL1	4	185676749	185747972	0.0081623	9.9433	5.19E-08	0.00804662	0.017086	3.9851	0.0032695	rs8078723	1.33E-13	0	5.30E-78	0	Thyroid
ENSG00000008838	MED24	17	38175350	38217468	ENSG00000151726	ACSL1	4	185676749	185747972	0.0081623	9.9433	5.19E-08	0.00804662	0.017086	3.9851	0.0032695	rs4794822	1.03E-12	0	1.32E-79	0	Esophagus_Gastroesophageal_Junction
ENSG00000008838	MED24	17	38175350	38217468	ENSG00000151726	ACSL1	4	185676749	185747972	0.0081623	9.9433	5.19E-08	0.00804662	0.017086	3.9851	0.0032695	rs4794822	1.03E-12	0	1.32E-79	0	Lung
ENSG00000008838	MED24	17	38175350	38217468	ENSG00000151726	ACSL1	4	185676749	185747972	0.0081623	9.9433	5.19E-08	0.00804662	0.017086	3.9851	0.0032695	rs4794822	1.03E-12	0	1.32E-79	0	Spleen
ENSG00000008838	MED24	17	38175350	38217468	ENSG00000151726	ACSL1	4	185676749	185747972	0.0081623	9.9433	5.19E-08	0.00804662	0.017086	3.9851	0.0032695	rs4794822	1.03E-12	0	1.32E-79	0	Whole_Blood
ENSG00000008838	MED24	17	38175350	38217468	ENSG00000151726	ACSL1	4	185676749	185747972	0.0081623	9.9433	5.19E-08	0.00804662	0.017086	3.9851	0.0032695	rs4065321	2.50E-12	0	1.24E-42	0	Pituitary
ENSG00000177963	RIC8A	11	207511	215113	ENSG00000161911	TREML1	6	41117080	41122075	0.0086981	8.4796	5.30E-08	0.00820025	0.018157	2.1105	0.032435	rs11602954	8.99E-32	0	7.55E-62	0	Adipose_Subcutaneous
ENSG00000177963	RIC8A	11	207511	215113	ENSG00000161911	TREML1	6	41117080	41122075	0.0086981	8.4796	5.30E-08	0.00820025	0.018157	2.1105	0.032435	rs11602954	8.99E-32	0	7.55E-62	0	Artery_Aorta
ENSG00000177963	RIC8A	11	207511	215113	ENSG00000161911	TREML1	6	41117080	41122075	0.0086981	8.4796	5.30E-08	0.00820025	0.018157	2.1105	0.032435	rs11602954	8.99E-32	0	7.55E-62	0	Artery_Tibial
ENSG00000177963	RIC8A	11	207511	215113	ENSG00000161911	TREML1	6	41117080	41122075	0.0086981	8.4796	5.30E-08	0.00820025	0.018157	2.1105	0.032435	rs11602954	8.99E-32	0	7.55E-62	0	Brain_Caudate_basal_ganglia
ENSG00000177963	RIC8A	11	207511	215113	ENSG00000161911	TREML1	6	41117080	41122075	0.0086981	8.4796	5.30E-08	0.00820025	0.018157	2.1105	0.032435	rs11602954	8.99E-32	0	7.55E-62	0	Brain_Frontal_Cortex_BA9
ENSG00000177963	RIC8A	11	207511	215113	ENSG00000161911	TREML1	6	41117080	41122075	0.0086981	8.4796	5.30E-08	0.00820025	0.018157	2.1105	0.032435	rs11602954	8.99E-32	0	7.55E-62	0	Colon_Sigmoid
ENSG00000177963	RIC8A	11	207511	215113	ENSG00000161911	TREML1	6	41117080	41122075	0.0086981	8.4796	5.30E-08	0.00820025	0.018157	2.1105	0.032435	rs11602954	8.99E-32	0	7.55E-62	0	Colon_Transverse
ENSG00000177963	RIC8A	11	207511	215113	ENSG00000161911	TREML1	6	41117080	41122075	0.0086981	8.4796	5.30E-08	0.00820025	0.018157	2.1105	0.032435	rs11602954	8.99E-32	0	7.55E-62	0	Esophagus_Gastroesophageal_Junction
ENSG00000177963	RIC8A	11	207511	215113	ENSG00000161911	TREML1	6	41117080	41122075	0.0086981	8.4796	5.30E-08	0.00820025	0.018157	2.1105	0.032435	rs11602954	8.99E-32	0	7.55E-62	0	Esophagus_Mucosa
ENSG00000177963	RIC8A	11	207511	215113	ENSG00000161911	TREML1	6	41117080	41122075	0.0086981	8.4796	5.30E-08	0.00820025	0.018157	2.1105	0.032435	rs11602954	8.99E-32	0	7.55E-62	0	Esophagus_Muscularis
ENSG00000177963	RIC8A	11	207511	215113	ENSG00000161911	TREML1	6	41117080	41122075	0.0086981	8.4796	5.30E-08	0.00820025	0.018157	2.1105	0.032435	rs11602954	8.99E-32	0	7.55E-62	0	Lung
ENSG00000177963	RIC8A	11	207511	215113	ENSG00000161911	TREML1	6	41117080	41122075	0.0086981	8.4796	5.30E-08	0.00820025	0.018157	2.1105	0.032435	rs11602954	8.99E-32	0	7.55E-62	0	Muscle_Skeletal
ENSG00000177963	RIC8A	11	207511	215113	ENSG00000161911	TREML1	6	41117080	41122075	0.0086981	8.4796	5.30E-08	0.00820025	0.018157	2.1105	0.032435	rs11602954	8.99E-32	0	7.55E-62	0	Nerve_Tibial
ENSG00000177963	RIC8A	11	207511	215113	ENSG00000161911	TREML1	6	41117080	41122075	0.0086981	8.4796	5.30E-08	0.00820025	0.018157	2.1105	0.032435	rs11602954	8.99E-32	0	7.55E-62	0	Skin_Not_Sun_Exposed_Suprapubic
ENSG00000177963	RIC8A	11	207511	215113	ENSG00000161911	TREML1	6	41117080	41122075	0.0086981	8.4796	5.30E-08	0.00820025	0.018157	2.1105	0.032435	rs11602954	8.99E-32	0	7.55E-62	0	Skin_Sun_Exposed_Lower_leg
ENSG00000177963	RIC8A	11	207511	215113	ENSG00000161911	TREML1	6	41117080	41122075	0.0086981	8.4796	5.30E-08	0.00820025	0.018157	2.1105	0.032435	rs11602954	8.99E-32	0	7.55E-62	0	Thyroid
ENSG00000177963	RIC8A	11	207511	215113	ENSG00000161911	TREML1	6	41117080	41122075	0.0086981	8.4796	5.30E-08	0.00820025	0.018157	2.1105	0.032435	rs17655730	1.45E-32	0	5.10E-36	0	Adipose_Subcutaneous
ENSG00000177963	RIC8A	11	207511	215113	ENSG00000161911	TREML1	6	41117080	41122075	0.0086981	8.4796	5.30E-08	0.00820025	0.018157	2.1105	0.032435	rs17655730	1.45E-32	0	5.10E-36	0	Esophagus_Mucosa
ENSG00000177963	RIC8A	11	207511	215113	ENSG00000161911	TREML1	6	41117080	41122075	0.0086981	8.4796	5.30E-08	0.00820025	0.018157	2.1105	0.032435	rs17655730	1.45E-32	0	5.10E-36	0	Esophagus_Muscularis
ENSG00000177963	RIC8A	11	207511	215113	ENSG00000161911	TREML1	6	41117080	41122075	0.0086981	8.4796	5.30E-08	0.00820025	0.018157	2.1105	0.032435	rs17655730	1.45E-32	0	5.10E-36	0	Heart_Left_Ventricle
ENSG00000177963	RIC8A	11	207511	215113	ENSG00000161911	TREML1	6	41117080	41122075	0.0086981	8.4796	5.30E-08	0.00820025	0.018157	2.1105	0.032435	rs17655730	1.45E-32	0	5.10E-36	0	Ovary
ENSG00000177963	RIC8A	11	207511	215113	ENSG00000161911	TREML1	6	41117080	41122075	0.0086981	8.4796	5.30E-08	0.00820025	0.018157	2.1105	0.032435	rs17655730	1.45E-32	0	5.10E-36	0	Pancreas
ENSG00000177963	RIC8A	11	207511	215113	ENSG00000161911	TREML1	6	41117080	41122075	0.0086981	8.4796	5.30E-08	0.00820025	0.018157	2.1105	0.032435	rs17655730	1.45E-32	0	5.10E-36	0	Skin_Not_Sun_Exposed_Suprapubic
ENSG00000177963	RIC8A	11	207511	215113	ENSG00000161911	TREML1	6	41117080	41122075	0.0086981	8.4796	5.30E-08	0.00820025	0.018157	2.1105	0.032435	rs505404	1.33E-35	0	3.66E-47	0	Artery_Aorta
ENSG00000177963	RIC8A	11	207511	215113	ENSG00000161911	TREML1	6	41117080	41122075	0.0086981	8.4796	5.30E-08	0.00820025	0.018157	2.1105	0.032435	rs505404	1.33E-35	0	3.66E-47	0	Thyroid
ENSG00000092020	PPP2R3C	14	35554673	35586382	ENSG00000135929	CYP27A1	2	219646479	219680016	0.0081477	9.9254	5.37E-08	0.00828574	0.0036074	0.83	0.50615	rs2143950	1.26E-12	0	3.2717e-310	0	Adrenal_Gland
ENSG00000092020	PPP2R3C	14	35554673	35586382	ENSG00000135929	CYP27A1	2	219646479	219680016	0.0081477	9.9254	5.37E-08	0.00828574	0.0036074	0.83	0.50615	rs2143950	1.26E-12	0	3.2717e-310	0	Skin_Sun_Exposed_Lower_leg
ENSG00000092020	PPP2R3C	14	35554673	35586382	ENSG00000135929	CYP27A1	2	219646479	219680016	0.0081477	9.9254	5.37E-08	0.00828574	0.0036074	0.83	0.50615	rs2143950	1.26E-12	0	3.2717e-310	0	Whole_Blood
ENSG00000108733	PEX12	17	33901814	33905882	ENSG00000146535	GNA12	7	2767746	2883958	0.0086898	8.4714	5.40E-08	0.00833086	0.01794	3.3466	0.005304	rs9915021	1.02E-15	0	NA	NA	Brain_Caudate_basal_ganglia
ENSG00000108733	PEX12	17	33901814	33905882	ENSG00000146535	GNA12	7	2767746	2883958	0.0086898	8.4714	5.40E-08	0.00833086	0.01794	3.3466	0.005304	rs9915021	1.02E-15	0	NA	NA	Breast_Mammary_Tissue
ENSG00000108733	PEX12	17	33901814	33905882	ENSG00000146535	GNA12	7	2767746	2883958	0.0086898	8.4714	5.40E-08	0.00833086	0.01794	3.3466	0.005304	rs10512472	1.14E-18	0	1.06E-12	0	Brain_Caudate_basal_ganglia
ENSG00000204444	APOM	6	31623248	31625987	ENSG00000100448	CTSG	14	25042762	25045466	0.0092024	7.4783	5.41E-08	0.00833882	0.019931	3.1013	0.0051771	rs1150753	1.65E-06	0.01287084	8.13E-16	0	Muscle_Skeletal
ENSG00000204444	APOM	6	31623248	31625987	ENSG00000100448	CTSG	14	25042762	25045466	0.0092024	7.4783	5.41E-08	0.00833882	0.019931	3.1013	0.0051771	rs1150753	1.65E-06	0.01287084	8.13E-16	0	Prostate
ENSG00000204444	APOM	6	31623248	31625987	ENSG00000100448	CTSG	14	25042762	25045466	0.0092024	7.4783	5.41E-08	0.00833882	0.019931	3.1013	0.0051771	rs1270942	6.73E-07	0.00583171	3.56E-16	0	Prostate
ENSG00000204444	APOM	6	31623248	31625987	ENSG00000100448	CTSG	14	25042762	25045466	0.0092024	7.4783	5.41E-08	0.00833882	0.019931	3.1013	0.0051771	rs1270942	6.73E-07	0.00583171	3.56E-16	0	Testis
ENSG00000204444	APOM	6	31623248	31625987	ENSG00000100448	CTSG	14	25042762	25045466	0.0092024	7.4783	5.41E-08	0.00833882	0.019931	3.1013	0.0051771	rs3131379	1.12E-07	0.00126582	3.41E-17	0	Cells_Transformed_fibroblasts
ENSG00000204444	APOM	6	31623248	31625987	ENSG00000100448	CTSG	14	25042762	25045466	0.0092024	7.4783	5.41E-08	0.00833882	0.019931	3.1013	0.0051771	rs3131379	1.12E-07	0.00126582	3.41E-17	0	Colon_Transverse
ENSG00000204444	APOM	6	31623248	31625987	ENSG00000100448	CTSG	14	25042762	25045466	0.0092024	7.4783	5.41E-08	0.00833882	0.019931	3.1013	0.0051771	rs3131379	1.12E-07	0.00126582	3.41E-17	0	Prostate
ENSG00000204444	APOM	6	31623248	31625987	ENSG00000100448	CTSG	14	25042762	25045466	0.0092024	7.4783	5.41E-08	0.00833882	0.019931	3.1013	0.0051771	rs3131379	1.12E-07	0.00126582	3.41E-17	0	Stomach
ENSG00000204444	APOM	6	31623248	31625987	ENSG00000100448	CTSG	14	25042762	25045466	0.0092024	7.4783	5.41E-08	0.00833882	0.019931	3.1013	0.0051771	rs3131379	1.12E-07	0.00126582	3.41E-17	0	Testis
ENSG00000163946	FAM208A	3	56658507	56717265	ENSG00000119280	C1orf198	1	230972865	231005335	0.010164	6.198	5.42E-08	0.00833917	0.0058666	0.77053	0.61217	rs12485738	6.15E-59	0	NA	NA	Esophagus_Mucosa
ENSG00000163946	FAM208A	3	56658507	56717265	ENSG00000119280	C1orf198	1	230972865	231005335	0.010164	6.198	5.42E-08	0.00833917	0.0058666	0.77053	0.61217	rs1354034	1.99E-103	0	NA	NA	Heart_Atrial_Appendage
ENSG00000132139	GAS2L2	17	34071530	34079897	ENSG00000141084	RANBP10	16	67757005	67840555	0.010162	6.1971	5.44E-08	0.00835187	0.0062712	0.72022	0.67382	rs9915021	1.13E-06	0.0090001	NA	NA	Small_Intestine_Terminal_Ileum
ENSG00000006125	AP2B1	17	33913918	34053436	ENSG00000168497	SDPR	2	192699028	192711981	0.0096877	6.7499	5.47E-08	0.00839474	0.0044911	0.68798	0.65941	rs10512472	7.94E-26	0	3.62E-14	0	Heart_Left_Ventricle
ENSG00000132139	GAS2L2	17	34071530	34079897	ENSG00000130300	PLVAP	19	17462257	17488159	0.01015	6.1896	5.58E-08	0.00853486	0.0069538	0.79916	0.60342	rs9915021	2.45E-12	0	NA	NA	Small_Intestine_Terminal_Ileum
ENSG00000142082	SIRT3	11	215458	236431	ENSG00000005961	ITGA2B	17	42449548	42466873	0.007522	12.212	5.86E-08	0.00893374	0.0098338	2.2768	0.059323	rs11602954	6.34E-48	0	1.39E-80	0	Adipose_Subcutaneous
ENSG00000142082	SIRT3	11	215458	236431	ENSG00000005961	ITGA2B	17	42449548	42466873	0.007522	12.212	5.86E-08	0.00893374	0.0098338	2.2768	0.059323	rs11602954	6.34E-48	0	1.39E-80	0	Artery_Tibial
ENSG00000142082	SIRT3	11	215458	236431	ENSG00000005961	ITGA2B	17	42449548	42466873	0.007522	12.212	5.86E-08	0.00893374	0.0098338	2.2768	0.059323	rs11602954	6.34E-48	0	1.39E-80	0	Breast_Mammary_Tissue
ENSG00000142082	SIRT3	11	215458	236431	ENSG00000005961	ITGA2B	17	42449548	42466873	0.007522	12.212	5.86E-08	0.00893374	0.0098338	2.2768	0.059323	rs11602954	6.34E-48	0	1.39E-80	0	Cells_Transformed_fibroblasts
ENSG00000142082	SIRT3	11	215458	236431	ENSG00000005961	ITGA2B	17	42449548	42466873	0.007522	12.212	5.86E-08	0.00893374	0.0098338	2.2768	0.059323	rs11602954	6.34E-48	0	1.39E-80	0	Nerve_Tibial
ENSG00000142082	SIRT3	11	215458	236431	ENSG00000005961	ITGA2B	17	42449548	42466873	0.007522	12.212	5.86E-08	0.00893374	0.0098338	2.2768	0.059323	rs505404	6.63E-50	0	1.05E-94	0	Adrenal_Gland
ENSG00000142082	SIRT3	11	215458	236431	ENSG00000005961	ITGA2B	17	42449548	42466873	0.007522	12.212	5.86E-08	0.00893374	0.0098338	2.2768	0.059323	rs505404	6.63E-50	0	1.05E-94	0	Artery_Aorta
ENSG00000142082	SIRT3	11	215458	236431	ENSG00000005961	ITGA2B	17	42449548	42466873	0.007522	12.212	5.86E-08	0.00893374	0.0098338	2.2768	0.059323	rs505404	6.63E-50	0	1.05E-94	0	Lung
ENSG00000142082	SIRT3	11	215458	236431	ENSG00000005961	ITGA2B	17	42449548	42466873	0.007522	12.212	5.86E-08	0.00893374	0.0098338	2.2768	0.059323	rs505404	6.63E-50	0	1.05E-94	0	Thyroid
ENSG00000142082	SIRT3	11	215458	236431	ENSG00000005961	ITGA2B	17	42449548	42466873	0.007522	12.212	5.86E-08	0.00893374	0.0098338	2.2768	0.059323	rs3782123	6.30E-07	0.00553372	NA	NA	Brain_Frontal_Cortex_BA9
ENSG00000108733	PEX12	17	33901814	33905882	ENSG00000140479	PCSK6	15	101840818	102065405	0.0086315	8.4141	6.17E-08	0.00932792	0.021886	4.0992	0.0010961	rs9915021	4.25E-15	0	NA	NA	Brain_Caudate_basal_ganglia
ENSG00000108733	PEX12	17	33901814	33905882	ENSG00000140479	PCSK6	15	101840818	102065405	0.0086315	8.4141	6.17E-08	0.00932792	0.021886	4.0992	0.0010961	rs9915021	4.25E-15	0	NA	NA	Breast_Mammary_Tissue
ENSG00000108733	PEX12	17	33901814	33905882	ENSG00000140479	PCSK6	15	101840818	102065405	0.0086315	8.4141	6.17E-08	0.00932792	0.021886	4.0992	0.0010961	rs10512472	3.03E-22	0	1.06E-12	0	Brain_Caudate_basal_ganglia
ENSG00000108306	FBXL20	17	37415384	37558776	ENSG00000151726	ACSL1	4	185676749	185747972	0.0086297	8.4124	6.19E-08	0.00935666	0.013075	2.427	0.033775	rs7212938	1.56E-07	0.00161211	NA	NA	Brain_Nucleus_accumbens_basal_ganglia
ENSG00000167914	GSDMA	17	38119226	38134019	ENSG00000163421	PROK2	3	71820807	71834357	0.0068411	16.652	6.21E-08	0.00935912	0.0057561	2.6602	0.070471	rs3859192	5.81E-16	0	6.22E-13	0	Adipose_Subcutaneous
ENSG00000167914	GSDMA	17	38119226	38134019	ENSG00000163421	PROK2	3	71820807	71834357	0.0068411	16.652	6.21E-08	0.00935912	0.0057561	2.6602	0.070471	rs3859192	5.81E-16	0	6.22E-13	0	Adipose_Visceral_Omentum
ENSG00000167914	GSDMA	17	38119226	38134019	ENSG00000163421	PROK2	3	71820807	71834357	0.0068411	16.652	6.21E-08	0.00935912	0.0057561	2.6602	0.070471	rs3859192	5.81E-16	0	6.22E-13	0	Adrenal_Gland
ENSG00000167914	GSDMA	17	38119226	38134019	ENSG00000163421	PROK2	3	71820807	71834357	0.0068411	16.652	6.21E-08	0.00935912	0.0057561	2.6602	0.070471	rs3859192	5.81E-16	0	6.22E-13	0	Artery_Aorta
ENSG00000167914	GSDMA	17	38119226	38134019	ENSG00000163421	PROK2	3	71820807	71834357	0.0068411	16.652	6.21E-08	0.00935912	0.0057561	2.6602	0.070471	rs3859192	5.81E-16	0	6.22E-13	0	Artery_Coronary
ENSG00000167914	GSDMA	17	38119226	38134019	ENSG00000163421	PROK2	3	71820807	71834357	0.0068411	16.652	6.21E-08	0.00935912	0.0057561	2.6602	0.070471	rs3859192	5.81E-16	0	6.22E-13	0	Artery_Tibial
ENSG00000167914	GSDMA	17	38119226	38134019	ENSG00000163421	PROK2	3	71820807	71834357	0.0068411	16.652	6.21E-08	0.00935912	0.0057561	2.6602	0.070471	rs3859192	5.81E-16	0	6.22E-13	0	Breast_Mammary_Tissue
ENSG00000167914	GSDMA	17	38119226	38134019	ENSG00000163421	PROK2	3	71820807	71834357	0.0068411	16.652	6.21E-08	0.00935912	0.0057561	2.6602	0.070471	rs3859192	5.81E-16	0	6.22E-13	0	Cells_Transformed_fibroblasts
ENSG00000167914	GSDMA	17	38119226	38134019	ENSG00000163421	PROK2	3	71820807	71834357	0.0068411	16.652	6.21E-08	0.00935912	0.0057561	2.6602	0.070471	rs3859192	5.81E-16	0	6.22E-13	0	Colon_Sigmoid
ENSG00000167914	GSDMA	17	38119226	38134019	ENSG00000163421	PROK2	3	71820807	71834357	0.0068411	16.652	6.21E-08	0.00935912	0.0057561	2.6602	0.070471	rs3859192	5.81E-16	0	6.22E-13	0	Colon_Transverse
ENSG00000167914	GSDMA	17	38119226	38134019	ENSG00000163421	PROK2	3	71820807	71834357	0.0068411	16.652	6.21E-08	0.00935912	0.0057561	2.6602	0.070471	rs3859192	5.81E-16	0	6.22E-13	0	Esophagus_Gastroesophageal_Junction
ENSG00000167914	GSDMA	17	38119226	38134019	ENSG00000163421	PROK2	3	71820807	71834357	0.0068411	16.652	6.21E-08	0.00935912	0.0057561	2.6602	0.070471	rs3859192	5.81E-16	0	6.22E-13	0	Esophagus_Muscularis
ENSG00000167914	GSDMA	17	38119226	38134019	ENSG00000163421	PROK2	3	71820807	71834357	0.0068411	16.652	6.21E-08	0.00935912	0.0057561	2.6602	0.070471	rs3859192	5.81E-16	0	6.22E-13	0	Heart_Atrial_Appendage
ENSG00000167914	GSDMA	17	38119226	38134019	ENSG00000163421	PROK2	3	71820807	71834357	0.0068411	16.652	6.21E-08	0.00935912	0.0057561	2.6602	0.070471	rs3859192	5.81E-16	0	6.22E-13	0	Heart_Left_Ventricle
ENSG00000167914	GSDMA	17	38119226	38134019	ENSG00000163421	PROK2	3	71820807	71834357	0.0068411	16.652	6.21E-08	0.00935912	0.0057561	2.6602	0.070471	rs3859192	5.81E-16	0	6.22E-13	0	Liver

ENSG00000167914	GSDMA	17	38119226	38134019	ENSG00000163421	PROK2	3	71820807	71834357	0.0068411	16.652	6.21E-08	0.00935912	0.0057561	2.6602	0.070471	rs3859192	5.81E-16	0	6.22E-13	0	Lung
ENSG00000167914	GSDMA	17	38119226	38134019	ENSG00000163421	PROK2	3	71820807	71834357	0.0068411	16.652	6.21E-08	0.00935912	0.0057561	2.6602	0.070471	rs3859192	5.81E-16	0	6.22E-13	0	Muscle_Skeletal
ENSG00000167914	GSDMA	17	38119226	38134019	ENSG00000163421	PROK2	3	71820807	71834357	0.0068411	16.652	6.21E-08	0.00935912	0.0057561	2.6602	0.070471	rs3859192	5.81E-16	0	6.22E-13	0	Nerve_Tibial
ENSG00000167914	GSDMA	17	38119226	38134019	ENSG00000163421	PROK2	3	71820807	71834357	0.0068411	16.652	6.21E-08	0.00935912	0.0057561	2.6602	0.070471	rs3859192	5.81E-16	0	6.22E-13	0	Ovary
ENSG00000167914	GSDMA	17	38119226	38134019	ENSG00000163421	PROK2	3	71820807	71834357	0.0068411	16.652	6.21E-08	0.00935912	0.0057561	2.6602	0.070471	rs3859192	5.81E-16	0	6.22E-13	0	Pancreas
ENSG00000167914	GSDMA	17	38119226	38134019	ENSG00000163421	PROK2	3	71820807	71834357	0.0068411	16.652	6.21E-08	0.00935912	0.0057561	2.6602	0.070471	rs3859192	5.81E-16	0	6.22E-13	0	Small_Intestine_Terminal_Ileum
ENSG00000167914	GSDMA	17	38119226	38134019	ENSG00000163421	PROK2	3	71820807	71834357	0.0068411	16.652	6.21E-08	0.00935912	0.0057561	2.6602	0.070471	rs3859192	5.81E-16	0	6.22E-13	0	Spleen
ENSG00000167914	GSDMA	17	38119226	38134019	ENSG00000163421	PROK2	3	71820807	71834357	0.0068411	16.652	6.21E-08	0.00935912	0.0057561	2.6602	0.070471	rs3859192	5.81E-16	0	6.22E-13	0	Stomach
ENSG00000167914	GSDMA	17	38119226	38134019	ENSG00000163421	PROK2	3	71820807	71834357	0.0068411	16.652	6.21E-08	0.00935912	0.0057561	2.6602	0.070471	rs3859192	5.81E-16	0	6.22E-13	0	Thyroid
ENSG00000167914	GSDMA	17	38119226	38134019	ENSG00000163421	PROK2	3	71820807	71834357	0.0068411	16.652	6.21E-08	0.00935912	0.0057561	2.6602	0.070471	rs3859192	5.81E-16	0	6.22E-13	0	Whole_Blood
ENSG00000167914	GSDMA	17	38119226	38134019	ENSG00000163421	PROK2	3	71820807	71834357	0.0068411	16.652	6.21E-08	0.00935912	0.0057561	2.6602	0.070471	rs3894194	1.54E-06	0.01202721	6.08E-29	0	Adipose_Subcutaneous
ENSG00000167914	GSDMA	17	38119226	38134019	ENSG00000163421	PROK2	3	71820807	71834357	0.0068411	16.652	6.21E-08	0.00935912	0.0057561	2.6602	0.070471	rs3894194	1.54E-06	0.01202721	6.08E-29	0	Adipose_Visceral_Omentum
ENSG00000167914	GSDMA	17	38119226	38134019	ENSG00000163421	PROK2	3	71820807	71834357	0.0068411	16.652	6.21E-08	0.00935912	0.0057561	2.6602	0.070471	rs3894194	1.54E-06	0.01202721	6.08E-29	0	Artery_Tibial
ENSG00000167914	GSDMA	17	38119226	38134019	ENSG00000163421	PROK2	3	71820807	71834357	0.0068411	16.652	6.21E-08	0.00935912	0.0057561	2.6602	0.070471	rs3894194	1.54E-06	0.01202721	6.08E-29	0	Colon_Transverse
ENSG00000167914	GSDMA	17	38119226	38134019	ENSG00000163421	PROK2	3	71820807	71834357	0.0068411	16.652	6.21E-08	0.00935912	0.0057561	2.6602	0.070471	rs3894194	1.54E-06	0.01202721	6.08E-29	0	Esophagus_Gastroesophageal_Junction
ENSG00000167914	GSDMA	17	38119226	38134019	ENSG00000163421	PROK2	3	71820807	71834357	0.0068411	16.652	6.21E-08	0.00935912	0.0057561	2.6602	0.070471	rs3894194	1.54E-06	0.01202721	6.08E-29	0	Heart_Atrial_Appendage
ENSG00000167914	GSDMA	17	38119226	38134019	ENSG00000163421	PROK2	3	71820807	71834357	0.0068411	16.652	6.21E-08	0.00935912	0.0057561	2.6602	0.070471	rs3894194	1.54E-06	0.01202721	6.08E-29	0	Muscle_Skeletal
ENSG00000167914	GSDMA	17	38119226	38134019	ENSG00000163421	PROK2	3	71820807	71834357	0.0068411	16.652	6.21E-08	0.00935912	0.0057561	2.6602	0.070471	rs3894194	1.54E-06	0.01202721	6.08E-29	0	Whole_Blood
ENSG00000167914	GSDMA	17	38119226	38134019	ENSG00000163421	PROK2	3	71820807	71834357	0.0068411	16.652	6.21E-08	0.00935912	0.0057561	2.6602	0.070471	rs8078723	2.41E-14	0	6.97E-07	0.002194627	Breast_Mammary_Tissue
ENSG00000167914	GSDMA	17	38119226	38134019	ENSG00000163421	PROK2	3	71820807	71834357	0.0068411	16.652	6.21E-08	0.00935912	0.0057561	2.6602	0.070471	rs8078723	2.41E-14	0	6.97E-07	0.002194627	Esophagus_Gastroesophageal_Junction
ENSG00000167914	GSDMA	17	38119226	38134019	ENSG00000163421	PROK2	3	71820807	71834357	0.0068411	16.652	6.21E-08	0.00935912	0.0057561	2.6602	0.070471	rs4794822	5.82E-14	0	3.02E-06	0.008756123	Breast_Mammary_Tissue
ENSG00000167914	GSDMA	17	38119226	38134019	ENSG00000163421	PROK2	3	71820807	71834357	0.0068411	16.652	6.21E-08	0.00935912	0.0057561	2.6602	0.070471	rs4794822	5.82E-14	0	3.02E-06	0.008756123	Cells_Transformed_fibroblasts
ENSG00000167914	GSDMA	17	38119226	38134019	ENSG00000163421	PROK2	3	71820807	71834357	0.0068411	16.652	6.21E-08	0.00935912	0.0057561	2.6602	0.070471	rs4794822	5.82E-14	0	3.02E-06	0.008756123	Esophagus_Gastroesophageal_Junction
ENSG00000167914	GSDMA	17	38119226	38134019	ENSG00000163421	PROK2	3	71820807	71834357	0.0068411	16.652	6.21E-08	0.00935912	0.0057561	2.6602	0.070471	rs4794822	5.82E-14	0	3.02E-06	0.008756123	Esophagus_Muscularis
ENSG00000167914	GSDMA	17	38119226	38134019	ENSG00000163421	PROK2	3	71820807	71834357	0.0068411	16.652	6.21E-08	0.00935912	0.0057561	2.6602	0.070471	rs4065321	6.39E-13	0	2.52E-12	0	Pancreas
ENSG00000177963	RIC8A	11	207511	215113	ENSG00000102362	SYTL4	X	99929488	99987110	0.0086182	8.4011	6.36E-08	0.00955645	0.021614	2.5212	0.010277	rs505404	4.66E-06	0.03095987	3.66E-47	0	Artery_Aorta
ENSG00000177963	RIC8A	11	207511	215113	ENSG00000102362	SYTL4	X	99929488	99987110	0.0086182	8.4011	6.36E-08	0.00955645	0.021614	2.5212	0.010277	rs505404	4.66E-06	0.03095987	3.66E-47	0	Thyroid
ENSG00000092871	RFFL	17	33341759	33416338	ENSG00000104904	OAZ1	19	2271369	2273487	0.0091264	7.416	6.41E-08	0.00963073	0.0088593	1.0201	0.41884	rs9915021	1.09E-09	3.93E-05	NA	NA	Adipose_Subcutaneous
ENSG00000092871	RFFL	17	33341759	33416338	ENSG00000104904	OAZ1	19	2271369	2273487	0.0091264	7.416	6.41E-08	0.00963073	0.0088593	1.0201	0.41884	rs9915021	1.09E-09	3.93E-05	NA	NA	Artery_Tibial
ENSG00000073605	GSDMB	17	38060848	38074903	ENSG00000151726	ACSL1	4	185676749	185747972	0.0080582	9.8154	6.61E-08	0.00989317	0.0088393	2.0445	0.086216	rs3859192	1.10E-13	0	3.2717e-310	0	Adrenal_Gland
ENSG00000073605	GSDMB	17	38060848	38074903	ENSG00000151726	ACSL1	4	185676749	185747972	0.0080582	9.8154	6.61E-08	0.00989317	0.0088393	2.0445	0.086216	rs3894194	1.60E-06	0.0125406	3.2717e-310	0	Esophagus_Mucosa
ENSG00000073605	GSDMB	17	38060848	38074903	ENSG00000151726	ACSL1	4	185676749	185747972	0.0080582	9.8154	6.61E-08	0.00989317	0.0088393	2.0445	0.086216	rs3894194	1.60E-06	0.0125406	3.2717e-310	0	Ovary
ENSG00000073605	GSDMB	17	38060848	38074903	ENSG00000151726	ACSL1	4	185676749	185747972	0.0080582	9.8154	6.61E-08	0.00989317	0.0088393	2.0445	0.086216	rs7212938	1.56E-07	0.00161211	3.2717e-310	0	Colon_Transverse
ENSG00000073605	GSDMB	17	38060848	38074903	ENSG00000151726	ACSL1	4	185676749	185747972	0.0080582	9.8154	6.61E-08	0.00989317	0.0088393	2.0445	0.086216	rs4794820	5.16E-07	0.00445765	3.2717e-310	0	Spleen
ENSG00000073605	GSDMB	17	38060848	38074903	ENSG00000151726	ACSL1	4	185676749	185747972	0.0080582	9.8154	6.61E-08	0.00989317	0.0088393	2.0445	0.086216	rs4794820	5.16E-07	0.00445765	3.2717e-310	0	Whole_Blood
ENSG00000172123	SLFN12	17	33738079	33760302	ENSG00000017483	SLC38A5	X	48316920	48328644	0.013034	4.2453	6.64E-08	0.00991674	0.012451	1.9228	0.074378	rs225245	4.68E-30	0	NA	NA	Cells_EBV-transformed_lymphocytes
ENSG00000172123	SLFN12	17	33738079	33760302	ENSG00000017483	SLC38A5	X	48316920	48328644	0.013034	4.2453	6.64E-08	0.00991674	0.012451	1.9228	0.074378	rs9915021	6.46E-06	0.04076003	NA	NA	Breast_Mammary_Tissue
ENSG00000172716	SLFN11	17	33677324	33700720	ENSG00000103148	NPRL3	16	138697	188859	0.011801	4.8018	6.86E-08	0.01017105	0.019364	1.4958	0.11962	rs225245	1.20E-79	0	5.77E-07	0.001884469	Cells_Transformed_fibroblasts
ENSG00000172716	SLFN11	17	33677324	33700720	ENSG00000103148	NPRL3	16	138697	188859	0.011801	4.8018	6.86E-08	0.01017105	0.019364	1.4958	0.11962	rs8082605	5.54E-34	0	NA	NA	Adrenal_Gland
ENSG00000154760	SLFN13	17	33762115	33775856	ENSG00000095321	CRAT	9	131857089	131873083	0.011381	5.0505	6.86E-08	0.01017105	0.014559	1.1191	0.34035	rs225245	9.71E-27	0	NA	NA	Liver
ENSG00000154760	SLFN13	17	33762115	33775856	ENSG00000095321	CRAT	9	131857089	131873083	0.011381	5.0505	6.86E-08	0.01017105	0.014559	1.1191	0.34035	rs8082605	2.34E-17	0	2.08E-19	0	Artery_Tibial
ENSG00000154760	SLFN13	17	33762115	33775856	ENSG00000095321	CRAT	9	131857089	131873083	0.011381	5.0505	6.86E-08	0.01017105	0.014559	1.1191	0.34035	rs8082605	2.34E-17	0	2.08E-19	0	Esophagus_Mucosa
ENSG00000154760	SLFN13	17	33762115	33775856	ENSG00000095321	CRAT	9	131857089	131873083	0.011381	5.0505	6.86E-08	0.01017105	0.014559	1.1191	0.34035	rs8082605	2.34E-17	0	2.08E-19	0	Nerve_Tibial
ENSG00000154760	SLFN13	17	33762115	33775856	ENSG00000095321	CRAT	9	131857089	131873083	0.011381	5.0505	6.86E-08	0.01017105	0.014559	1.1191	0.34035	rs8082605	2.34E-17	0	2.08E-19	0	Pituitary
ENSG00000154760	SLFN13	17	33762115	33775856	ENSG00000095321	CRAT	9	131857089	131873083	0.011381	5.0505	6.86E-08	0.01017105	0.014559	1.1191	0.34035	rs10512472	7.64E-26	0	6.34E-38	0	Uterus
ENSG00000172660	TAF15	17	34136459	34174246	ENSG00000135924	DNAJB2	2	220143989	220151622	0.0090948	7.39	6.88E-08	0.01019243	0.010643	1.6405	0.13281	rs9915021	1.49E-09	5.75E-05	NA	NA	Skin_Not_Sun_Exposed_Suprapubic
ENSG00000163946	FAM208A	3	56658507	56717265	ENSG00000154146	NRGN	11	124609829	124615878	0.010051	6.1289	6.90E-08	0.01020271	0.022519	3.008	0.0039609	rs12485738	3.85E-230	0	NA	NA	Esophagus_Mucosa
ENSG00000163946	FAM208A	3	56658507	56717265	ENSG00000154146	NRGN	11	124609829	124615878	0.010051	6.1289	6.90E-08	0.01020271	0.022519	3.008	0.0039609	rs1354034	3.27170000000001e-	0	NA	NA	Heart_Atrial_Appendage
ENSG00000203663	OR2L2	1	248201474	248202607	ENSG00000166086	JAM3	11	133938820	134018713	0.009091	7.3869	6.94E-08	0.01025061	0.018173	2.8226	0.0099775	rs3811445	8.19E-246	0	NA	NA	Brain_Nucleus_accumbens_basal_ganglia
ENSG00000204444	APOM	6	31623248	31625987	ENSG00000165617	DACT1	14	59100685	59115039	0.0090873	7.3839	7.00E-08	0.01029558	0.018886	2.9355	0.0076607	rs1265097	6.41E-10	2.05E-05	NA	NA	Colon_Transverse
ENSG00000204444	APOM	6	31623248	31625987	ENSG00000165617	DACT1	14	59100685	59115039	0.0090873	7.3839	7.00E-08	0.01029558	0.018886	2.9355	0.0076607	rs2233956	7.14E-09	0.000134	4.06E-08	0.000158559	Muscle_Skeletal
ENSG00000204444	APOM	6	31623248	31625987	ENSG00000165617	DACT1	14	59100685	59115039	0.0090873	7.3839	7.00E-08	0.01029558	0.018886	2.9355	0.0076607	rs2233956	7.14E-09	0.000134	4.06E-08	0.000158559	Pancreas
ENSG00000204444	APOM	6	31623248	31625987	ENSG00000165617	DACT1	14	59100685	59115039	0.0090873	7.3839	7.00E-08	0.01029558	0.018886	2.9355	0.0076607	rs652888	1.35E-07	0.00147851	6.56E-13	0	Muscle_Skeletal
ENSG00000196260	SFTA2	6	30899130	30899952	ENSG00000101425	BPI	20	36888551	36965907	0.0085721	8.3557	7.06E-08	0.01035387	0.011261	2.0865	0.064895	rs6929796	2.96E-07	0.00278853	NA	NA	Whole_Blood
ENSG00000172123	SLFN12	17	33738079	33760302	ENSG00000100614	PPM1A	14	60712470	60765805	0.012994	4.2321	7.18E-08	0.0105076	0.0098368	1.515	0.16996	rs225245	1.24E-19	0	NA	NA	Cells_EBV-transformed_lymphocytes
ENSG00000172123	SLFN12	17	33738079	33760302	ENSG00000100614	PPM1A	14	60712470	60765805	0.012994	4.2321	7.18E-08	0.0105076	0.0098368	1.515	0.16996	rs9915021	6.10E-09	0.00013559	NA	NA	Breast_Mammary_Tissue
ENSG00000172660	TAF15	17	34136459	34174246	ENSG00000163554	SPTA1	1	158580278	158656488	0.0090704	7.37	7.27E-08	0.01061635	0.0053184	0.8154	0.558	rs9915021	5.11E-06	0.03338736	NA	NA	Skin_Not_Sun_Exposed_Suprapubic
ENSG00000154768	C17orf50	17	34087916	34092098	ENSG00000164849	GPR146	7	1084212	1098897	0.0090648	7.3654	7.36E-08	0.01074162	0.0075555	0.99404	0.43401	rs9915021	1.21E-09	3.90E-05	NA	NA	Pituitary
ENSG00000108733	PEX12	17	33901814	33905882	ENSG00000183508	FAM46C	1	118148556	118170994	0.0085526	8.3365	7.38E-08	0.01076184	0.0061962	1.1422	0.33623	rs10512472	6.42E-12	0	1.06E-12	0	Brain_Caudate_basal_ganglia
ENSG00000172716	SLFN11	17	33677324	33700720	ENSG00000130300	PLVAP	19	17462257	17488159	0.011759	4.7843	7.48E-08	0.01089407	0.016379	1.2614	0.23632	rs225245	1.20E-20	0	5.77E-07	0.001884469	Cells_Transformed_fibroblasts
ENSG00000172716	SLFN11	17	33677324	33700720	ENSG00000130300	PLVAP	19	17462257	17488159	0.011759	4.7843	7.48E-08	0.01089407	0.016379	1.2614	0.23632	rs8082605	1.32E-12	0	NA	NA	Adrenal_Gland
ENSG00000172716	SLFN11	17	33677324	33700720	ENSG00000140564	FURIN	15	91411822	91426688	0.011757	4.7837	7.51E-08	0.01091848	0.010247	0.78423	0.66724	rs225245	2.51E-30	0	5.77E-07	0.001884469	Cells_Transformed_fibroblasts
ENSG00000172716	SLFN11	17	33677324	33700720	ENSG00000140564	FURIN	15	91411822	91426688	0.011757	4.7837	7.51E-08	0.01091848	0.010247	0.78423	0.66724	rs8082605	2.70E-18	0	NA	NA	Adrenal_Gland
ENSG00000172123	SLFN12	17	33738079	33760302	ENSG00000158828	PINK1	1	20959948	20978004	0.01297	4.2242	7.53E-08	0.01094722	0.007518	1.1552	0.3283	rs225245	1.45E-52	0	NA	NA	Cells_EBV-transformed_lymphocytes
ENSG00000172123	SLFN12	17	33738079	33760302	ENSG00000158828	PINK1	1	20959948	20978004	0.01297	4.2242	7.53E-08	0.01094722	0.007518	1.1552	0.3283	rs9915021	5.07E-27	0	NA	NA	Breast_Mammary_Tissue
ENSG00000099849	RASSF7	11	560404	564021	ENSG00000144677	CTDSPL	3	37903451	38025960	0.0095405	6.6463	7.55E-08	0.01096496	0.009854	1.2995	0.24725	rs505404	1.75E-49	0	NA	NA	Brain_Frontal_Cortex_BA9
ENSG00000099849	RASSF7	11	560404	564021	ENSG00000144677	CTDSPL	3	37903451	38025960	0.0095405	6.6463	7.55E-08	0.01096496	0.009854	1.2995	0.24725	rs3782123	4.11E-07	0.00366492	NA	NA	Whole_Blood
ENSG00000009950	MLXIPL	7	73007524	73038873	ENSG00000140287	HDC	15	50534144	50558223	0.0079991	9.7429	7.58E-08	0.01098293	0.0079291	1.2188	0.294	rs17145738	8.16E-06	0.04922912	NA	NA	Adipose_Subcutaneous
ENSG00000009950	MLXIPL	7	73007524	73038873	ENSG00000140287	HDC	15	50534144	50558223	0.0079991	9.7429	7.58E-08	0.01098293	0.0079291	1.2188	0.294	rs17145738	8.16E-06	0.04922912	NA	NA	Cells_Transformed_fibroblasts
ENSG00000009950	MLXIPL	7	73007524	73038873	ENSG00000140287	HDC	15	50534144	50558223	0.0079991	9.7429	7.58E-08	0.01098293	0.0079291	1.2188	0.294	rs17145738	8.16E-06	0.04922912	NA	NA	Colon_Sigmoid
ENSG00000009950	MLXIPL	7	73007524	73038873	ENSG00000140287	HDC	15	50534144	50558223	0.0079991	9.7429	7.58E-08	0.01098293	0.0079291	1.2188	0.294	rs17145738	8.16E-06	0.04922912	NA	NA	Esophagus_Mucosa
ENSG00000009950	MLXIPL	7	73007524	73038873	ENSG00000140287	HDC	15	50534144	50558223	0.0079991	9.7429	7.58E-08	0.01098293	0.0079291	1.2188	0.294	rs17145738	8.16E-06	0.04922912	NA	NA	Heart_Atrial_Appendage
ENSG00000009950	MLXIPL	7	73007524	73038873	ENSG00000140287	HDC	15	50534144	50558223	0.0079991	9.7429	7.58E-08	0.01098293	0.0079291	1.2188	0.294	rs17145738	8.16E-06	0.04922912	NA	NA	Heart_Left_Ventricle
ENSG00000009950	MLXIPL	7	73007524	73038873	ENSG00000140287	HDC	15	50534144	50558223	0.0079991	9.7429	7.58E-08	0.01098293	0.0079291	1.2188	0.294	rs17145738	8.16E-06	0.04922912	NA	NA	Pancreas
ENSG00000009950	MLXIPL	7	73007524	73038873	ENSG00000140287	HDC	15	50534144	50558223	0.0079991	9.7429	7.58E-08	0.01098293	0.0079291	1.2188	0.294	rs17145738	8.16E-06	0.04922912	NA	NA	Skin_Not_Sun_Exposed_Suprapubic
ENSG00000009950	MLXIPL	7	73007524	73038873	ENSG00000140287	HDC	15	50534144	50558223	0.0079991	9.7429	7.58E-08	0.01098293	0.0079291	1.2188	0.294	rs17145738	8.16E-06	0.04922912	NA	NA	Thyroid
ENSG00000009950	MLXIPL	7	73007524	73038873	ENSG00000140287	HDC	15	50534144	50558223	0.0079991	9.7429	7.58E-08	0.01098293	0.0079291	1.2188	0.294	rs13233571	6.18E-06	0.039326	NA	NA	Pancreas
ENSG00000009950	MLXIPL	7	73007524	73038873	ENSG00000140287	HDC	15	50534144	50558223	0.0079991	9.7429	7.58E-08	0.01098293	0.0079291	1.2188	0.294	rs13233571	6.18E-06	0.039326	NA	NA	Skin_Sun_Exposed_Lower_leg
ENSG00000009950	MLXIPL	7	73007524	73038873	ENSG00000140287	HDC	15	50534144	50558223	0.0079991	9.7429	7.58E-08	0.01098293	0.0079291	1.2188	0.294	rs2240466	1.14E-06	0.00910127	NA	NA	Adipose_Visceral_Omentum
ENSG00000009950	MLXIPL	7	73007524	73038873	ENSG00000140287	HDC	15	50534144	50558223	0.0079991	9.7429	7.58E-08	0.01098293	0.0079291	1.2188	0.294	rs2240466	1.14E-06	0.00910127	NA	NA	Colon_Sigmoid
ENSG00000009950	MLXIPL	7	73007524	73038873	ENSG00000140287	HDC	15	50534144	50558223	0.0079991	9.7429	7.58E-08	0.01098293	0.0079291	1.2188	0.294	rs2240466	1.14E-06	0.00910127	NA	NA	Esophagus_Mucosa
ENSG00000009950	MLXIPL	7	73007524	73038873	ENSG00000140287	HDC	15	50534144	50558223	0.0079991	9.7429	7.58E-08	0.01098293	0.0079291	1.2188	0.294	rs2240466	1.14E-06	0.00910127	NA	NA	Pancreas

ENSG00000009950	MLXIPL	7	73007524	73038873	ENSG00000140287	HDC	15	50534144	50558223	0.0079991	9.7429	7.58E-08	0.01098293	0.0079291	1.2188	0.294	rs2240466	1.14E-06	0.00910127	NA	NA	Skin_Not_Sun_Exposed_Suprapubic
ENSG00000009950	MLXIPL	7	73007524	73038873	ENSG00000140287	HDC	15	50534144	50558223	0.0079991	9.7429	7.58E-08	0.01098293	0.0079291	1.2188	0.294	rs2240466	1.14E-06	0.00910127	NA	NA	Skin_Sun_Exposed_Lower_leg
ENSG00000009950	MLXIPL	7	73007524	73038873	ENSG00000140287	HDC	15	50534144	50558223	0.0079991	9.7429	7.58E-08	0.01098293	0.0079291	1.2188	0.294	rs13231516	4.38E-06	0.02956957	NA	NA	Adipose_Visceral_Omentum
ENSG00000009950	MLXIPL	7	73007524	73038873	ENSG00000140287	HDC	15	50534144	50558223	0.0079991	9.7429	7.58E-08	0.01098293	0.0079291	1.2188	0.294	rs13231516	4.38E-06	0.02956957	NA	NA	Colon_Sigmoid
ENSG00000009950	MLXIPL	7	73007524	73038873	ENSG00000140287	HDC	15	50534144	50558223	0.0079991	9.7429	7.58E-08	0.01098293	0.0079291	1.2188	0.294	rs13231516	4.38E-06	0.02956957	NA	NA	Esophagus_Mucosa
ENSG00000009950	MLXIPL	7	73007524	73038873	ENSG00000140287	HDC	15	50534144	50558223	0.0079991	9.7429	7.58E-08	0.01098293	0.0079291	1.2188	0.294	rs13231516	4.38E-06	0.02956957	NA	NA	Pancreas
ENSG00000009950	MLXIPL	7	73007524	73038873	ENSG00000140287	HDC	15	50534144	50558223	0.0079991	9.7429	7.58E-08	0.01098293	0.0079291	1.2188	0.294	rs13231516	4.38E-06	0.02956957	NA	NA	Skin_Not_Sun_Exposed_Suprapubic
ENSG00000009950	MLXIPL	7	73007524	73038873	ENSG00000140287	HDC	15	50534144	50558223	0.0079991	9.7429	7.58E-08	0.01098293	0.0079291	1.2188	0.294	rs13231516	4.38E-06	0.02956957	NA	NA	Skin_Sun_Exposed_Lower_leg
ENSG00000009950	MLXIPL	7	73007524	73038873	ENSG00000140287	HDC	15	50534144	50558223	0.0079991	9.7429	7.58E-08	0.01098293	0.0079291	1.2188	0.294	rs714052	3.32E-06	0.02351536	NA	NA	Adipose_Visceral_Omentum
ENSG00000009950	MLXIPL	7	73007524	73038873	ENSG00000140287	HDC	15	50534144	50558223	0.0079991	9.7429	7.58E-08	0.01098293	0.0079291	1.2188	0.294	rs714052	3.32E-06	0.02351536	NA	NA	Colon_Sigmoid
ENSG00000009950	MLXIPL	7	73007524	73038873	ENSG00000140287	HDC	15	50534144	50558223	0.0079991	9.7429	7.58E-08	0.01098293	0.0079291	1.2188	0.294	rs714052	3.32E-06	0.02351536	NA	NA	Esophagus_Mucosa
ENSG00000009950	MLXIPL	7	73007524	73038873	ENSG00000140287	HDC	15	50534144	50558223	0.0079991	9.7429	7.58E-08	0.01098293	0.0079291	1.2188	0.294	rs714052	3.32E-06	0.02351536	NA	NA	Pancreas
ENSG00000009950	MLXIPL	7	73007524	73038873	ENSG00000140287	HDC	15	50534144	50558223	0.0079991	9.7429	7.58E-08	0.01098293	0.0079291	1.2188	0.294	rs714052	3.32E-06	0.02351536	NA	NA	Skin_Not_Sun_Exposed_Suprapubic
ENSG00000009950	MLXIPL	7	73007524	73038873	ENSG00000140287	HDC	15	50534144	50558223	0.0079991	9.7429	7.58E-08	0.01098293	0.0079291	1.2188	0.294	rs714052	3.32E-06	0.02351536	NA	NA	Skin_Sun_Exposed_Lower_leg
ENSG00000142102	ATHL1	11	289138	296107	ENSG00000102362	SYTL4	X	99929488	99987110	0.014496	3.7301	7.66E-08	0.01106526	0.0058649	2.7108	0.067015	rs505404	4.66E-06	0.03095987	3.83E-63	0	Lung
ENSG00000188076	SCGB1C1	11	193080	194573	ENSG00000259207	ITGB3	17	45387505	45389182	0.0059557	28.974	7.68E-08	0.01108821	0.0013753	1.267	0.26063	rs11602954	1.90E-32	0	3.14E-203	0	Whole_Blood
ENSG00000188076	SCGB1C1	11	193080	194573	ENSG00000259207	ITGB3	17	45387505	45389182	0.0059557	28.974	7.68E-08	0.01108821	0.0013753	1.267	0.26063	rs17655730	6.92E-36	0	1.60E-163	0	Whole_Blood
ENSG00000188076	SCGB1C1	11	193080	194573	ENSG00000259207	ITGB3	17	45387505	45389182	0.0059557	28.974	7.68E-08	0.01108821	0.0013753	1.267	0.26063	rs505404	5.02E-41	0	5.81E-188	0	Whole_Blood
ENSG00000177963	RIC8A	11	207511	215113	ENSG00000185245	GP1BA	17	4835592	4838325	0.0085235	8.3079	7.88E-08	0.01134303	0.013658	1.5803	0.12651	rs11602954	3.88E-45	0	7.55E-62	0	Adipose_Subcutaneous
ENSG00000177963	RIC8A	11	207511	215113	ENSG00000185245	GP1BA	17	4835592	4838325	0.0085235	8.3079	7.88E-08	0.01134303	0.013658	1.5803	0.12651	rs11602954	3.88E-45	0	7.55E-62	0	Artery_Aorta
ENSG00000177963	RIC8A	11	207511	215113	ENSG00000185245	GP1BA	17	4835592	4838325	0.0085235	8.3079	7.88E-08	0.01134303	0.013658	1.5803	0.12651	rs11602954	3.88E-45	0	7.55E-62	0	Artery_Tibial
ENSG00000177963	RIC8A	11	207511	215113	ENSG00000185245	GP1BA	17	4835592	4838325	0.0085235	8.3079	7.88E-08	0.01134303	0.013658	1.5803	0.12651	rs11602954	3.88E-45	0	7.55E-62	0	Brain_Caudate_basal_ganglia
ENSG00000177963	RIC8A	11	207511	215113	ENSG00000185245	GP1BA	17	4835592	4838325	0.0085235	8.3079	7.88E-08	0.01134303	0.013658	1.5803	0.12651	rs11602954	3.88E-45	0	7.55E-62	0	Brain_Frontal_Cortex_BA9
ENSG00000177963	RIC8A	11	207511	215113	ENSG00000185245	GP1BA	17	4835592	4838325	0.0085235	8.3079	7.88E-08	0.01134303	0.013658	1.5803	0.12651	rs11602954	3.88E-45	0	7.55E-62	0	Colon_Sigmoid
ENSG00000177963	RIC8A	11	207511	215113	ENSG00000185245	GP1BA	17	4835592	4838325	0.0085235	8.3079	7.88E-08	0.01134303	0.013658	1.5803	0.12651	rs11602954	3.88E-45	0	7.55E-62	0	Colon_Transverse
ENSG00000177963	RIC8A	11	207511	215113	ENSG00000185245	GP1BA	17	4835592	4838325	0.0085235	8.3079	7.88E-08	0.01134303	0.013658	1.5803	0.12651	rs11602954	3.88E-45	0	7.55E-62	0	Esophagus_Gastroesophageal_Junction
ENSG00000177963	RIC8A	11	207511	215113	ENSG00000185245	GP1BA	17	4835592	4838325	0.0085235	8.3079	7.88E-08	0.01134303	0.013658	1.5803	0.12651	rs11602954	3.88E-45	0	7.55E-62	0	Esophagus_Mucosa
ENSG00000177963	RIC8A	11	207511	215113	ENSG00000185245	GP1BA	17	4835592	4838325	0.0085235	8.3079	7.88E-08	0.01134303	0.013658	1.5803	0.12651	rs11602954	3.88E-45	0	7.55E-62	0	Esophagus_Muscularis
ENSG00000177963	RIC8A	11	207511	215113	ENSG00000185245	GP1BA	17	4835592	4838325	0.0085235	8.3079	7.88E-08	0.01134303	0.013658	1.5803	0.12651	rs11602954	3.88E-45	0	7.55E-62	0	Lung
ENSG00000177963	RIC8A	11	207511	215113	ENSG00000185245	GP1BA	17	4835592	4838325	0.0085235	8.3079	7.88E-08	0.01134303	0.013658	1.5803	0.12651	rs11602954	3.88E-45	0	7.55E-62	0	Muscle_Skeletal
ENSG00000177963	RIC8A	11	207511	215113	ENSG00000185245	GP1BA	17	4835592	4838325	0.0085235	8.3079	7.88E-08	0.01134303	0.013658	1.5803	0.12651	rs11602954	3.88E-45	0	7.55E-62	0	Nerve_Tibial
ENSG00000177963	RIC8A	11	207511	215113	ENSG00000185245	GP1BA	17	4835592	4838325	0.0085235	8.3079	7.88E-08	0.01134303	0.013658	1.5803	0.12651	rs11602954	3.88E-45	0	7.55E-62	0	Skin_Not_Sun_Exposed_Suprapubic
ENSG00000177963	RIC8A	11	207511	215113	ENSG00000185245	GP1BA	17	4835592	4838325	0.0085235	8.3079	7.88E-08	0.01134303	0.013658	1.5803	0.12651	rs11602954	3.88E-45	0	7.55E-62	0	Skin_Sun_Exposed_Lower_leg
ENSG00000177963	RIC8A	11	207511	215113	ENSG00000185245	GP1BA	17	4835592	4838325	0.0085235	8.3079	7.88E-08	0.01134303	0.013658	1.5803	0.12651	rs11602954	3.88E-45	0	7.55E-62	0	Thyroid
ENSG00000177963	RIC8A	11	207511	215113	ENSG00000185245	GP1BA	17	4835592	4838325	0.0085235	8.3079	7.88E-08	0.01134303	0.013658	1.5803	0.12651	rs17655730	4.78E-41	0	5.10E-36	0	Adipose_Subcutaneous
ENSG00000177963	RIC8A	11	207511	215113	ENSG00000185245	GP1BA	17	4835592	4838325	0.0085235	8.3079	7.88E-08	0.01134303	0.013658	1.5803	0.12651	rs17655730	4.78E-41	0	5.10E-36	0	Esophagus_Mucosa
ENSG00000177963	RIC8A	11	207511	215113	ENSG00000185245	GP1BA	17	4835592	4838325	0.0085235	8.3079	7.88E-08	0.01134303	0.013658	1.5803	0.12651	rs17655730	4.78E-41	0	5.10E-36	0	Esophagus_Muscularis
ENSG00000177963	RIC8A	11	207511	215113	ENSG00000185245	GP1BA	17	4835592	4838325	0.0085235	8.3079	7.88E-08	0.01134303	0.013658	1.5803	0.12651	rs17655730	4.78E-41	0	5.10E-36	0	Heart_Left_Ventricle
ENSG00000177963	RIC8A	11	207511	215113	ENSG00000185245	GP1BA	17	4835592	4838325	0.0085235	8.3079	7.88E-08	0.01134303	0.013658	1.5803	0.12651	rs17655730	4.78E-41	0	5.10E-36	0	Ovary
ENSG00000177963	RIC8A	11	207511	215113	ENSG00000185245	GP1BA	17	4835592	4838325	0.0085235	8.3079	7.88E-08	0.01134303	0.013658	1.5803	0.12651	rs17655730	4.78E-41	0	5.10E-36	0	Pancreas
ENSG00000177963	RIC8A	11	207511	215113	ENSG00000185245	GP1BA	17	4835592	4838325	0.0085235	8.3079	7.88E-08	0.01134303	0.013658	1.5803	0.12651	rs17655730	4.78E-41	0	5.10E-36	0	Skin_Not_Sun_Exposed_Suprapubic
ENSG00000177963	RIC8A	11	207511	215113	ENSG00000185245	GP1BA	17	4835592	4838325	0.0085235	8.3079	7.88E-08	0.01134303	0.013658	1.5803	0.12651	rs7104764	4.37E-06	0.0294305	1.68E-167	0	Nerve_Tibial
ENSG00000177963	RIC8A	11	207511	215113	ENSG00000185245	GP1BA	17	4835592	4838325	0.0085235	8.3079	7.88E-08	0.01134303	0.013658	1.5803	0.12651	rs505404	2.82E-47	0	3.66E-47	0	Artery_Aorta
ENSG00000177963	RIC8A	11	207511	215113	ENSG00000185245	GP1BA	17	4835592	4838325	0.0085235	8.3079	7.88E-08	0.01134303	0.013658	1.5803	0.12651	rs505404	2.82E-47	0	3.66E-47	0	Thyroid
ENSG00000108349	CASC3	17	38296576	38328436	ENSG00000162551	ALPL	1	21835858	21904905	0.0090287	7.3358	7.98E-08	0.01145119	0.0019891	0.30394	0.935	rs12946510	8.70E-07	0.00729639	NA	NA	Prostate
ENSG00000108349	CASC3	17	38296576	38328436	ENSG00000162551	ALPL	1	21835858	21904905	0.0090287	7.3358	7.98E-08	0.01145119	0.0019891	0.30394	0.935	rs3859192	3.20E-29	0	NA	NA	Esophagus_Mucosa
ENSG00000108349	CASC3	17	38296576	38328436	ENSG00000162551	ALPL	1	21835858	21904905	0.0090287	7.3358	7.98E-08	0.01145119	0.0019891	0.30394	0.935	rs17609240	8.77E-13	0	NA	NA	Prostate
ENSG00000108349	CASC3	17	38296576	38328436	ENSG00000162551	ALPL	1	21835858	21904905	0.0090287	7.3358	7.98E-08	0.01145119	0.0019891	0.30394	0.935	rs8078723	3.63E-38	0	NA	NA	Esophagus_Mucosa
ENSG00000108349	CASC3	17	38296576	38328436	ENSG00000162551	ALPL	1	21835858	21904905	0.0090287	7.3358	7.98E-08	0.01145119	0.0019891	0.30394	0.935	rs9303277	3.21E-07	0.00291306	NA	NA	Prostate
ENSG00000180376	CCDC66	3	56591189	56653929	ENSG00000122786	CALD1	7	134429003	134655479	0.0085175	8.302	7.99E-08	0.01145581	0.011127	2.0614	0.068029	rs17825630	2.02E-25	0	NA	NA	Brain_Anterior_cingulate_cortex_BA24
ENSG00000180376	CCDC66	3	56591189	56653929	ENSG00000122786	CALD1	7	134429003	134655479	0.0085175	8.302	7.99E-08	0.01145581	0.011127	2.0614	0.068029	rs17825630	2.02E-25	0	NA	NA	Cells_EBV-transformed_lymphocytes
ENSG00000180376	CCDC66	3	56591189	56653929	ENSG00000122786	CALD1	7	134429003	134655479	0.0085175	8.302	7.99E-08	0.01145581	0.011127	2.0614	0.068029	rs1354034	3.27170000000001e-	0	NA	NA	Brain_Cerebellar_Hemisphere
ENSG00000180376	CCDC66	3	56591189	56653929	ENSG00000122786	CALD1	7	134429003	134655479	0.0085175	8.302	7.99E-08	0.01145581	0.011127	2.0614	0.068029	rs1354034	3.27170000000001e-	0	NA	NA	Cells_EBV-transformed_lymphocytes
ENSG00000180376	CCDC66	3	56591189	56653929	ENSG00000122786	CALD1	7	134429003	134655479	0.0085175	8.302	7.99E-08	0.01145581	0.011127	2.0614	0.068029	rs1354034	3.27170000000001e-	0	NA	NA	Testis
ENSG00000006125	AP2B1	17	33913918	34053436	ENSG00000151693	ASAP2	2	9346894	9541525	0.0095103	6.6251	8.07E-08	0.01154067	0.0052255	0.80107	0.56914	rs10512472	3.33E-19	0	3.62E-14	0	Heart_Left_Ventricle
ENSG00000099849	RASSF7	11	560404	564021	ENSG00000204420	C6orf25	6	31691121	31694491	0.0095043	6.6209	8.18E-08	0.01165731	0.0096433	1.2714	0.26132	rs505404	9.44E-22	0	NA	NA	Brain_Frontal_Cortex_BA9
ENSG00000006125	AP2B1	17	33913918	34053436	ENSG00000149218	ENDOD1	11	94822974	94865809	0.0094989	6.6171	8.27E-08	0.01176284	0.015	2.3223	0.031251	rs10512472	1.01E-44	0	3.62E-14	0	Heart_Left_Ventricle
ENSG00000006125	AP2B1	17	33913918	34053436	ENSG00000118816	CCNI	4	77968311	77997158	0.0094739	6.5995	8.74E-08	0.01229244	0.002338	0.35737	0.90575	rs10512472	1.80E-07	0.00175269	3.62E-14	0	Heart_Left_Ventricle
ENSG00000205045	SLFN12L	17	33800708	33864880	ENSG00000011105	TSPAN9	12	3186521	3395730	0.0094717	6.5979	8.78E-08	0.01234389	0.032284	4.356	9.20E-05	rs8082605	5.88E-65	0	2.49E-31	0	Adipose_Subcutaneous
ENSG00000205045	SLFN12L	17	33800708	33864880	ENSG00000011105	TSPAN9	12	3186521	3395730	0.0094717	6.5979	8.78E-08	0.01234389	0.032284	4.356	9.20E-05	rs8082605	5.88E-65	0	2.49E-31	0	Adipose_Visceral_Omentum
ENSG00000205045	SLFN12L	17	33800708	33864880	ENSG00000011105	TSPAN9	12	3186521	3395730	0.0094717	6.5979	8.78E-08	0.01234389	0.032284	4.356	9.20E-05	rs8082605	5.88E-65	0	2.49E-31	0	Heart_Left_Ventricle
ENSG00000205045	SLFN12L	17	33800708	33864880	ENSG00000011105	TSPAN9	12	3186521	3395730	0.0094717	6.5979	8.78E-08	0.01234389	0.032284	4.356	9.20E-05	rs8082605	5.88E-65	0	2.49E-31	0	Nerve_Tibial
ENSG00000205045	SLFN12L	17	33800708	33864880	ENSG00000011105	TSPAN9	12	3186521	3395730	0.0094717	6.5979	8.78E-08	0.01234389	0.032284	4.356	9.20E-05	rs10512472	5.91E-134	0	4.96E-30	0	Pancreas
ENSG00000174885	NLRP6	11	278365	285359	ENSG00000185245	GP1BA	17	4835592	4838325	0.0094706	6.5972	8.80E-08	0.0123638	0.021402	2.2162	0.019147	rs11602954	3.88E-45	0	2.12E-70	0	Nerve_Tibial
ENSG00000174885	NLRP6	11	278365	285359	ENSG00000185245	GP1BA	17	4835592	4838325	0.0094706	6.5972	8.80E-08	0.0123638	0.021402	2.2162	0.019147	rs17655730	4.78E-41	0	5.43E-117	0	Artery_Aorta
ENSG00000174885	NLRP6	11	278365	285359	ENSG00000185245	GP1BA	17	4835592	4838325	0.0094706	6.5972	8.80E-08	0.0123638	0.021402	2.2162	0.019147	rs17655730	4.78E-41	0	5.43E-117	0	Nerve_Tibial
ENSG00000174885	NLRP6	11	278365	285359	ENSG00000185245	GP1BA	17	4835592	4838325	0.0094706	6.5972	8.80E-08	0.0123638	0.021402	2.2162	0.019147	rs17655730	4.78E-41	0	5.43E-117	0	Skin_Sun_Exposed_Lower_leg
ENSG00000174885	NLRP6	11	278365	285359	ENSG00000185245	GP1BA	17	4835592	4838325	0.0094706	6.5972	8.80E-08	0.0123638	0.021402	2.2162	0.019147	rs505404	2.82E-47	0	2.65E-88	0	Nerve_Tibial
ENSG00000174885	NLRP6	11	278365	285359	ENSG00000185245	GP1BA	17	4835592	4838325	0.0094706	6.5972	8.80E-08	0.0123638	0.021402	2.2162	0.019147	rs505404	2.82E-47	0	2.65E-88	0	Prostate
ENSG00000092871	RFFL	17	33341759	33416338	ENSG00000238243	OR2W3	1	248058859	248060449	0.0089763	7.2928	8.96E-08	0.01256272	0.0073205	0.84162	0.566	rs9915021	7.68E-06	0.04691593	NA	NA	Adipose_Subcutaneous
ENSG00000092871	RFFL	17	33341759	33416338	ENSG00000238243	OR2W3	1	248058859	248060449	0.0089763	7.2928	8.96E-08	0.01256272	0.0073205	0.84162	0.566	rs9915021	7.68E-06	0.04691593	NA	NA	Artery_Tibial
ENSG00000006125	AP2B1	17	33913918	34053436	ENSG00000035403	VCL	10	75757872	75879918	0.0094557	6.5867	9.09E-08	0.01271242	0.0030852	0.47195	0.82944	rs10512472	4.73E-19	0	3.62E-14	0	Heart_Left_Ventricle
ENSG00000180376	CCDC66	3	56591189	56653929	ENSG00000120885	CLU	8	27454434	27472548	0.0084601	8.2456	9.10E-08	0.01271409	0.016292	3.0342	0.010061	rs17825630	4.19E-07	0.00376578	NA	NA	Brain_Anterior_cingulate_cortex_BA24
ENSG00000180376	CCDC66	3	56591189	56653929	ENSG00000120885	CLU	8	27454434	27472548	0.0084601	8.2456	9.10E-08	0.01271409	0.016292	3.0342	0.010061	rs17825630	4.19E-07	0.00376578	NA	NA	Cells_EBV-transformed_lymphocytes
ENSG00000180376	CCDC66	3	56591189	56653929	ENSG00000120885	CLU	8	27454434	27472548	0.0084601	8.2456	9.10E-08	0.01271409	0.016292	3.0342	0.010061	rs1354034	2.24E-91	0	NA	NA	Brain_Cerebellar_Hemisphere
ENSG00000180376	CCDC66	3	56591189	56653929	ENSG00000120885	CLU	8	27454434	27472548	0.0084601	8.2456	9.10E-08	0.01271409	0.016292	3.0342	0.010061	rs1354034	2.24E-91	0	NA	NA	Cells_EBV-transformed_lymphocytes
ENSG00000180376	CCDC66	3	56591189	56653929	ENSG00000120885	CLU	8	27454434	27472548	0.0084601	8.2456	9.10E-08	0.01271409	0.016292	3.0342	0.010061	rs1354034	2.24E-91	0	NA	NA	Testis
ENSG00000174885	NLRP6	11	278365	285359	ENSG00000174175	SELP	1	169558087	169599431	0.0094471	6.5806	9.27E-08	0.01289271	0.011656	1.1951	0.29442	rs11602954	2.72E-34	0	2.12E-70	0	Nerve_Tibial
ENSG00000174885	NLRP6	11	278365	285359	ENSG00000174175	SELP	1	169558087	169599431	0.0094471	6.5806	9.27E-08	0.01289271	0.011656	1.1951	0.29442	rs17655730	4.69E-37	0	5.43E-117	0	Artery_Aorta
ENSG00000174885	NLRP6	11	278365	285359	ENSG00000174175	SELP	1	169558087	169599431	0.0094471	6.5806	9.27E-08	0.01289271	0.011656	1.1951	0.29442	rs17655730	4.69E-37	0	5.43E-117	0	Nerve_Tibial
ENSG00000174885	NLRP6	11	278365	285359	ENSG00000174175	SELP	1	169558087	169599431	0.0094471	6.5806	9.27E-08	0.01289271	0.011656	1.1951	0.29442	rs17655730	4.69E-37	0	5.43E-117	0	Skin_Sun_Exposed_Lower_leg
ENSG00000174885	NLRP6	11	278365	285359	ENSG00000174175	SELP	1	169558087	169599431	0.0094471	6.5806	9.27E-08	0.01289271	0.011656	1.1951	0.29442	rs505404	2.87E-40	0	2.65E-88	0	Nerve_Tibial

ENSG00000174885	NLRP6	11	278365	285359	ENSG00000174175	SELP	1	169558087	169599431	0.0094471	6.5806	9.27E-08	0.01289271	0.011656	1.1951	0.29442	rs505404	2.87E-40	0	2.65E-88	0	Prostate
ENSG00000157500	APPL1	3	57261765	57301820	ENSG00000259207	ITGB3	17	45387505	45389182	0.0079014	9.6229	9.50E-08	0.01317565	0.015751	4.8969	0.0022106	rs12485738	2.40E-225	0	NA	NA	Brain_Anterior_cingulate_cortex_BA24
ENSG00000239388	ASB14	3	57310556	57326710	ENSG00000174175	SELP	1	169558087	169599431	0.0084373	8.2232	9.58E-08	0.01327704	0.014952	2.7808	0.016779	rs17825630	1.73E-18	0	NA	NA	Skin_Not_Sun_Exposed_Suprapubic
ENSG00000239388	ASB14	3	57310556	57326710	ENSG00000174175	SELP	1	169558087	169599431	0.0084373	8.2232	9.58E-08	0.01327704	0.014952	2.7808	0.016779	rs12485738	4.31E-202	0	NA	NA	Artery_Tibial
ENSG00000239388	ASB14	3	57310556	57326710	ENSG00000174175	SELP	1	169558087	169599431	0.0084373	8.2232	9.58E-08	0.01327704	0.014952	2.7808	0.016779	rs1354034	3.27170000000001e-	0	NA	NA	Skin_Not_Sun_Exposed_Suprapubic
ENSG00000205045	SLFN12L	17	33800708	33864880	ENSG00000148341	SH3GLB2	9	131771385	131790582	0.0094186	6.5606	9.86E-08	0.01357188	0.0072217	0.94981	0.46701	rs8082605	2.41E-14	0	2.49E-31	0	Adipose_Subcutaneous
ENSG00000205045	SLFN12L	17	33800708	33864880	ENSG00000148341	SH3GLB2	9	131771385	131790582	0.0094186	6.5606	9.86E-08	0.01357188	0.0072217	0.94981	0.46701	rs8082605	2.41E-14	0	2.49E-31	0	Adipose_Visceral_Omentum
ENSG00000205045	SLFN12L	17	33800708	33864880	ENSG00000148341	SH3GLB2	9	131771385	131790582	0.0094186	6.5606	9.86E-08	0.01357188	0.0072217	0.94981	0.46701	rs8082605	2.41E-14	0	2.49E-31	0	Heart_Left_Ventricle
ENSG00000205045	SLFN12L	17	33800708	33864880	ENSG00000148341	SH3GLB2	9	131771385	131790582	0.0094186	6.5606	9.86E-08	0.01357188	0.0072217	0.94981	0.46701	rs8082605	2.41E-14	0	2.49E-31	0	Nerve_Tibial
ENSG00000172716	SLFN11	17	33677324	33700720	ENSG00000103257	SLC7A5	16	87863629	87903094	0.011611	4.7232	1.01E-07	0.01384367	0.018272	1.4098	0.15515	rs225245	2.55E-09	7.35E-05	5.77E-07	0.001884469	Cells_Transformed_fibroblasts
ENSG00000172716	SLFN11	17	33677324	33700720	ENSG00000103257	SLC7A5	16	87863629	87903094	0.011611	4.7232	1.01E-07	0.01384367	0.018272	1.4098	0.15515	rs8082605	1.28E-07	0.00137418	NA	NA	Adrenal_Gland
ENSG00000008838	MED24	17	38175350	38217468	ENSG00000135842	FAM129A	1	184759858	184943682	0.0078695	9.5837	1.02E-07	0.01398789	0.014187	3.2991	0.010721	rs12946510	1.75E-09	5.67E-05	NA	NA	Prostate
ENSG00000008838	MED24	17	38175350	38217468	ENSG00000135842	FAM129A	1	184759858	184943682	0.0078695	9.5837	1.02E-07	0.01398789	0.014187	3.2991	0.010721	rs3859192	2.10E-34	0	1.47E-40	0	Whole_Blood
ENSG00000008838	MED24	17	38175350	38217468	ENSG00000135842	FAM129A	1	184759858	184943682	0.0078695	9.5837	1.02E-07	0.01398789	0.014187	3.2991	0.010721	rs3894194	3.63E-19	0	8.18E-15	0	Thyroid
ENSG00000008838	MED24	17	38175350	38217468	ENSG00000135842	FAM129A	1	184759858	184943682	0.0078695	9.5837	1.02E-07	0.01398789	0.014187	3.2991	0.010721	rs7212938	1.48E-15	0	4.22E-10	0	Testis
ENSG00000008838	MED24	17	38175350	38217468	ENSG00000135842	FAM129A	1	184759858	184943682	0.0078695	9.5837	1.02E-07	0.01398789	0.014187	3.2991	0.010721	rs7212938	1.48E-15	0	4.22E-10	0	Thyroid
ENSG00000008838	MED24	17	38175350	38217468	ENSG00000135842	FAM129A	1	184759858	184943682	0.0078695	9.5837	1.02E-07	0.01398789	0.014187	3.2991	0.010721	rs8078723	1.78E-38	0	5.30E-78	0	Adipose_Subcutaneous
ENSG00000008838	MED24	17	38175350	38217468	ENSG00000135842	FAM129A	1	184759858	184943682	0.0078695	9.5837	1.02E-07	0.01398789	0.014187	3.2991	0.010721	rs8078723	1.78E-38	0	5.30E-78	0	Artery_Tibial
ENSG00000008838	MED24	17	38175350	38217468	ENSG00000135842	FAM129A	1	184759858	184943682	0.0078695	9.5837	1.02E-07	0.01398789	0.014187	3.2991	0.010721	rs8078723	1.78E-38	0	5.30E-78	0	Cells_Transformed_fibroblasts
ENSG00000008838	MED24	17	38175350	38217468	ENSG00000135842	FAM129A	1	184759858	184943682	0.0078695	9.5837	1.02E-07	0.01398789	0.014187	3.2991	0.010721	rs8078723	1.78E-38	0	5.30E-78	0	Lung
ENSG00000008838	MED24	17	38175350	38217468	ENSG00000135842	FAM129A	1	184759858	184943682	0.0078695	9.5837	1.02E-07	0.01398789	0.014187	3.2991	0.010721	rs8078723	1.78E-38	0	5.30E-78	0	Pituitary
ENSG00000008838	MED24	17	38175350	38217468	ENSG00000135842	FAM129A	1	184759858	184943682	0.0078695	9.5837	1.02E-07	0.01398789	0.014187	3.2991	0.010721	rs8078723	1.78E-38	0	5.30E-78	0	Skin_Sun_Exposed_Lower_leg
ENSG00000008838	MED24	17	38175350	38217468	ENSG00000135842	FAM129A	1	184759858	184943682	0.0078695	9.5837	1.02E-07	0.01398789	0.014187	3.2991	0.010721	rs8078723	1.78E-38	0	5.30E-78	0	Spleen
ENSG00000008838	MED24	17	38175350	38217468	ENSG00000135842	FAM129A	1	184759858	184943682	0.0078695	9.5837	1.02E-07	0.01398789	0.014187	3.2991	0.010721	rs8078723	1.78E-38	0	5.30E-78	0	Stomach
ENSG00000008838	MED24	17	38175350	38217468	ENSG00000135842	FAM129A	1	184759858	184943682	0.0078695	9.5837	1.02E-07	0.01398789	0.014187	3.2991	0.010721	rs8078723	1.78E-38	0	5.30E-78	0	Thyroid
ENSG00000008838	MED24	17	38175350	38217468	ENSG00000135842	FAM129A	1	184759858	184943682	0.0078695	9.5837	1.02E-07	0.01398789	0.014187	3.2991	0.010721	rs4794822	2.20E-38	0	1.32E-79	0	Esophagus_Gastroesophageal_Junction
ENSG00000008838	MED24	17	38175350	38217468	ENSG00000135842	FAM129A	1	184759858	184943682	0.0078695	9.5837	1.02E-07	0.01398789	0.014187	3.2991	0.010721	rs4794822	2.20E-38	0	1.32E-79	0	Lung
ENSG00000008838	MED24	17	38175350	38217468	ENSG00000135842	FAM129A	1	184759858	184943682	0.0078695	9.5837	1.02E-07	0.01398789	0.014187	3.2991	0.010721	rs4794822	2.20E-38	0	1.32E-79	0	Spleen
ENSG00000008838	MED24	17	38175350	38217468	ENSG00000135842	FAM129A	1	184759858	184943682	0.0078695	9.5837	1.02E-07	0.01398789	0.014187	3.2991	0.010721	rs4794822	2.20E-38	0	1.32E-79	0	Whole_Blood
ENSG00000008838	MED24	17	38175350	38217468	ENSG00000135842	FAM129A	1	184759858	184943682	0.0078695	9.5837	1.02E-07	0.01398789	0.014187	3.2991	0.010721	rs4065321	7.18E-33	0	1.24E-42	0	Pituitary
ENSG00000008838	MED24	17	38175350	38217468	ENSG00000135842	FAM129A	1	184759858	184943682	0.0078695	9.5837	1.02E-07	0.01398789	0.014187	3.2991	0.010721	rs8067378	3.15E-09	0.00010811	NA	NA	Brain_Hypothalamus
ENSG00000186075	ZPBP2	17	38024417	38034149	ENSG00000151726	ACSL1	4	185676749	185747972	0.0058384	28.4	1.03E-07	0.01405313	0.00091958	0.84679	0.3577	rs3859192	1.10E-13	0	NA	NA	Testis
ENSG00000186075	ZPBP2	17	38024417	38034149	ENSG00000151726	ACSL1	4	185676749	185747972	0.0058384	28.4	1.03E-07	0.01405313	0.00091958	0.84679	0.3577	rs4794820	5.16E-07	0.00445765	NA	NA	Testis
ENSG00000142102	ATHL1	11	289138	296107	ENSG00000095303	PTGS1	9	125132824	125157982	0.014326	3.6855	1.05E-07	0.01432979	0.00080099	0.36835	0.69198	rs11602954	3.11E-14	0	8.23E-62	0	Thyroid
ENSG00000142102	ATHL1	11	289138	296107	ENSG00000095303	PTGS1	9	125132824	125157982	0.014326	3.6855	1.05E-07	0.01432979	0.00080099	0.36835	0.69198	rs17655730	1.75E-16	0	1.10E-69	0	Muscle_Skeletal
ENSG00000142102	ATHL1	11	289138	296107	ENSG00000095303	PTGS1	9	125132824	125157982	0.014326	3.6855	1.05E-07	0.01432979	0.00080099	0.36835	0.69198	rs505404	4.38E-17	0	3.83E-63	0	Lung
ENSG00000172123	SLFN12	17	33738079	33760302	ENSG00000204463	BAG6	6	31606805	31620170	0.012796	4.1667	1.06E-07	0.01437596	0.023628	3.6904	0.0012485	rs225245	2.41E-35	0	NA	NA	Cells_EBV-transformed_lymphocytes
ENSG00000172123	SLFN12	17	33738079	33760302	ENSG00000204463	BAG6	6	31606805	31620170	0.012796	4.1667	1.06E-07	0.01437596	0.023628	3.6904	0.0012485	rs9915021	2.40E-12	0	NA	NA	Breast_Mammary_Tissue
ENSG00000172660	TAF15	17	34136459	34174246	ENSG00000143774	GUK1	1	228327663	228336685	0.0088923	7.224	1.08E-07	0.01459198	0.013334	2.061	0.055402	rs9915021	1.78E-10	0	NA	NA	Skin_Not_Sun_Exposed_Suprapubic
ENSG00000151327	FAM177A1	14	35514113	35582336	ENSG00000135929	CYP27A1	2	219646479	219680016	0.011159	4.9511	1.08E-07	0.01460397	0.02169	1.8341	0.044618	rs2143950	1.26E-12	0	1.11E-25	0	Adrenal_Gland
ENSG00000151327	FAM177A1	14	35514113	35582336	ENSG00000135929	CYP27A1	2	219646479	219680016	0.011159	4.9511	1.08E-07	0.01460397	0.02169	1.8341	0.044618	rs2143950	1.26E-12	0	1.11E-25	0	Skin_Sun_Exposed_Lower_leg
ENSG00000167914	GSDMA	17	38119226	38134019	ENSG00000125538	IL1B	2	113587328	113594480	0.0066084	16.082	1.09E-07	0.01473416	0.010589	4.9177	0.0075096	rs3859192	3.70E-26	0	6.22E-13	0	Adipose_Subcutaneous
ENSG00000167914	GSDMA	17	38119226	38134019	ENSG00000125538	IL1B	2	113587328	113594480	0.0066084	16.082	1.09E-07	0.01473416	0.010589	4.9177	0.0075096	rs3859192	3.70E-26	0	6.22E-13	0	Adipose_Visceral_Omentum
ENSG00000167914	GSDMA	17	38119226	38134019	ENSG00000125538	IL1B	2	113587328	113594480	0.0066084	16.082	1.09E-07	0.01473416	0.010589	4.9177	0.0075096	rs3859192	3.70E-26	0	6.22E-13	0	Adrenal_Gland
ENSG00000167914	GSDMA	17	38119226	38134019	ENSG00000125538	IL1B	2	113587328	113594480	0.0066084	16.082	1.09E-07	0.01473416	0.010589	4.9177	0.0075096	rs3859192	3.70E-26	0	6.22E-13	0	Artery_Aorta
ENSG00000167914	GSDMA	17	38119226	38134019	ENSG00000125538	IL1B	2	113587328	113594480	0.0066084	16.082	1.09E-07	0.01473416	0.010589	4.9177	0.0075096	rs3859192	3.70E-26	0	6.22E-13	0	Artery_Coronary
ENSG00000167914	GSDMA	17	38119226	38134019	ENSG00000125538	IL1B	2	113587328	113594480	0.0066084	16.082	1.09E-07	0.01473416	0.010589	4.9177	0.0075096	rs3859192	3.70E-26	0	6.22E-13	0	Artery_Tibial
ENSG00000167914	GSDMA	17	38119226	38134019	ENSG00000125538	IL1B	2	113587328	113594480	0.0066084	16.082	1.09E-07	0.01473416	0.010589	4.9177	0.0075096	rs3859192	3.70E-26	0	6.22E-13	0	Breast_Mammary_Tissue
ENSG00000167914	GSDMA	17	38119226	38134019	ENSG00000125538	IL1B	2	113587328	113594480	0.0066084	16.082	1.09E-07	0.01473416	0.010589	4.9177	0.0075096	rs3859192	3.70E-26	0	6.22E-13	0	Cells_Transformed_fibroblasts
ENSG00000167914	GSDMA	17	38119226	38134019	ENSG00000125538	IL1B	2	113587328	113594480	0.0066084	16.082	1.09E-07	0.01473416	0.010589	4.9177	0.0075096	rs3859192	3.70E-26	0	6.22E-13	0	Colon_Sigmoid
ENSG00000167914	GSDMA	17	38119226	38134019	ENSG00000125538	IL1B	2	113587328	113594480	0.0066084	16.082	1.09E-07	0.01473416	0.010589	4.9177	0.0075096	rs3859192	3.70E-26	0	6.22E-13	0	Colon_Transverse
ENSG00000167914	GSDMA	17	38119226	38134019	ENSG00000125538	IL1B	2	113587328	113594480	0.0066084	16.082	1.09E-07	0.01473416	0.010589	4.9177	0.0075096	rs3859192	3.70E-26	0	6.22E-13	0	Esophagus_Gastroesophageal_Junction
ENSG00000167914	GSDMA	17	38119226	38134019	ENSG00000125538	IL1B	2	113587328	113594480	0.0066084	16.082	1.09E-07	0.01473416	0.010589	4.9177	0.0075096	rs3859192	3.70E-26	0	6.22E-13	0	Esophagus_Muscularis
ENSG00000167914	GSDMA	17	38119226	38134019	ENSG00000125538	IL1B	2	113587328	113594480	0.0066084	16.082	1.09E-07	0.01473416	0.010589	4.9177	0.0075096	rs3859192	3.70E-26	0	6.22E-13	0	Heart_Atrial_Appendage
ENSG00000167914	GSDMA	17	38119226	38134019	ENSG00000125538	IL1B	2	113587328	113594480	0.0066084	16.082	1.09E-07	0.01473416	0.010589	4.9177	0.0075096	rs3859192	3.70E-26	0	6.22E-13	0	Heart_Left_Ventricle
ENSG00000167914	GSDMA	17	38119226	38134019	ENSG00000125538	IL1B	2	113587328	113594480	0.0066084	16.082	1.09E-07	0.01473416	0.010589	4.9177	0.0075096	rs3859192	3.70E-26	0	6.22E-13	0	Liver
ENSG00000167914	GSDMA	17	38119226	38134019	ENSG00000125538	IL1B	2	113587328	113594480	0.0066084	16.082	1.09E-07	0.01473416	0.010589	4.9177	0.0075096	rs3859192	3.70E-26	0	6.22E-13	0	Lung
ENSG00000167914	GSDMA	17	38119226	38134019	ENSG00000125538	IL1B	2	113587328	113594480	0.0066084	16.082	1.09E-07	0.01473416	0.010589	4.9177	0.0075096	rs3859192	3.70E-26	0	6.22E-13	0	Muscle_Skeletal
ENSG00000167914	GSDMA	17	38119226	38134019	ENSG00000125538	IL1B	2	113587328	113594480	0.0066084	16.082	1.09E-07	0.01473416	0.010589	4.9177	0.0075096	rs3859192	3.70E-26	0	6.22E-13	0	Nerve_Tibial
ENSG00000167914	GSDMA	17	38119226	38134019	ENSG00000125538	IL1B	2	113587328	113594480	0.0066084	16.082	1.09E-07	0.01473416	0.010589	4.9177	0.0075096	rs3859192	3.70E-26	0	6.22E-13	0	Ovary
ENSG00000167914	GSDMA	17	38119226	38134019	ENSG00000125538	IL1B	2	113587328	113594480	0.0066084	16.082	1.09E-07	0.01473416	0.010589	4.9177	0.0075096	rs3859192	3.70E-26	0	6.22E-13	0	Pancreas
ENSG00000167914	GSDMA	17	38119226	38134019	ENSG00000125538	IL1B	2	113587328	113594480	0.0066084	16.082	1.09E-07	0.01473416	0.010589	4.9177	0.0075096	rs3859192	3.70E-26	0	6.22E-13	0	Small_Intestine_Terminal_Ileum
ENSG00000167914	GSDMA	17	38119226	38134019	ENSG00000125538	IL1B	2	113587328	113594480	0.0066084	16.082	1.09E-07	0.01473416	0.010589	4.9177	0.0075096	rs3859192	3.70E-26	0	6.22E-13	0	Spleen
ENSG00000167914	GSDMA	17	38119226	38134019	ENSG00000125538	IL1B	2	113587328	113594480	0.0066084	16.082	1.09E-07	0.01473416	0.010589	4.9177	0.0075096	rs3859192	3.70E-26	0	6.22E-13	0	Stomach
ENSG00000167914	GSDMA	17	38119226	38134019	ENSG00000125538	IL1B	2	113587328	113594480	0.0066084	16.082	1.09E-07	0.01473416	0.010589	4.9177	0.0075096	rs3859192	3.70E-26	0	6.22E-13	0	Thyroid
ENSG00000167914	GSDMA	17	38119226	38134019	ENSG00000125538	IL1B	2	113587328	113594480	0.0066084	16.082	1.09E-07	0.01473416	0.010589	4.9177	0.0075096	rs3859192	3.70E-26	0	6.22E-13	0	Whole_Blood
ENSG00000167914	GSDMA	17	38119226	38134019	ENSG00000125538	IL1B	2	113587328	113594480	0.0066084	16.082	1.09E-07	0.01473416	0.010589	4.9177	0.0075096	rs17609240	3.00E-18	0	1.53E-144	0	Adipose_Subcutaneous
ENSG00000167914	GSDMA	17	38119226	38134019	ENSG00000125538	IL1B	2	113587328	113594480	0.0066084	16.082	1.09E-07	0.01473416	0.010589	4.9177	0.0075096	rs17609240	3.00E-18	0	1.53E-144	0	Adipose_Visceral_Omentum
ENSG00000167914	GSDMA	17	38119226	38134019	ENSG00000125538	IL1B	2	113587328	113594480	0.0066084	16.082	1.09E-07	0.01473416	0.010589	4.9177	0.0075096	rs17609240	3.00E-18	0	1.53E-144	0	Artery_Aorta
ENSG00000167914	GSDMA	17	38119226	38134019	ENSG00000125538	IL1B	2	113587328	113594480	0.0066084	16.082	1.09E-07	0.01473416	0.010589	4.9177	0.0075096	rs17609240	3.00E-18	0	1.53E-144	0	Breast_Mammary_Tissue
ENSG00000167914	GSDMA	17	38119226	38134019	ENSG00000125538	IL1B	2	113587328	113594480	0.0066084	16.082	1.09E-07	0.01473416	0.010589	4.9177	0.0075096	rs17609240	3.00E-18	0	1.53E-144	0	Colon_Sigmoid
ENSG00000167914	GSDMA	17	38119226	38134019	ENSG00000125538	IL1B	2	113587328	113594480	0.0066084	16.082	1.09E-07	0.01473416	0.010589	4.9177	0.0075096	rs17609240	3.00E-18	0	1.53E-144	0	Esophagus_Gastroesophageal_Junction
ENSG00000167914	GSDMA	17	38119226	38134019	ENSG00000125538	IL1B	2	113587328	113594480	0.0066084	16.082	1.09E-07	0.01473416	0.010589	4.9177	0.0075096	rs17609240	3.00E-18	0	1.53E-144	0	Esophagus_Muscularis
ENSG00000167914	GSDMA	17	38119226	38134019	ENSG00000125538	IL1B	2	113587328	113594480	0.0066084	16.082	1.09E-07	0.01473416	0.010589	4.9177	0.0075096	rs17609240	3.00E-18	0	1.53E-144	0	Heart_Left_Ventricle
ENSG00000167914	GSDMA	17	38119226	38134019	ENSG00000125538	IL1B	2	113587328	113594480	0.0066084	16.082	1.09E-07	0.01473416	0.010589	4.9177	0.0075096	rs17609240	3.00E-18	0	1.53E-144	0	Muscle_Skeletal
ENSG00000167914	GSDMA	17	38119226	38134019	ENSG00000125538	IL1B	2	113587328	113594480	0.0066084	16.082	1.09E-07	0.01473416	0.010589	4.9177	0.0075096	rs3894194	8.02E-17	0	6.08E-29	0	Adipose_Subcutaneous
ENSG00000167914	GSDMA	17	38119226	38134019	ENSG00000125538	IL1B	2	113587328	113594480	0.0066084	16.082	1.09E-07	0.01473416	0.010589	4.9177	0.0075096	rs3894194	8.02E-17	0	6.08E-29	0	Adipose_Visceral_Omentum
ENSG00000167914	GSDMA	17	38119226	38134019	ENSG00000125538	IL1B	2	113587328	113594480	0.0066084	16.082	1.09E-07	0.01473416	0.010589	4.9177	0.0075096	rs3894194	8.02E-17	0	6.08E-29	0	Artery_Tibial
ENSG00000167914	GSDMA	17	38119226	38134019	ENSG00000125538	IL1B	2	113587328	113594480	0.0066084	16.082	1.09E-07	0.01473416	0.010589	4.9177	0.0075096	rs3894194	8.02E-17	0	6.08E-29	0	Colon_Transverse
ENSG00000167914	GSDMA	17	38119226	38134019	ENSG00000125538	IL1B	2	113587328	113594480	0.0066084	16.082	1.09E-07	0.01473416	0.010589	4.9177	0.0075096	rs3894194	8.02E-17	0	6.08E-29	0	Esophagus_Gastroesophageal_Junction
ENSG00000167914	GSDMA	17	38119226	38134019	ENSG00000125538	IL1B	2	113587328	113594480	0.0066084	16.082	1.09E-07	0.01473416	0.010589	4.9177	0.0075096	rs3894194	8.02E-17	0	6.08E-29	0	Heart_Atrial_Appendage
ENSG00000167914	GSDMA	17	38119226	38134019	ENSG00000125538	IL1B	2	113587328	113594480	0.0066084	16.082	1.09E-07	0.01473416	0.010589	4.9177	0.0075096	rs3894194	8.02E-17	0	6.08E-29	0	Muscle_Skeletal

ENSG00000167914	GSDMA	17	38119226	38134019	ENSG00000125538	IL1B	2	113587328	113594480	0.0066084	16.082	1.09E-07	0.01473416	0.010589	4.9177	0.0075096	rs3894194	8.02E-17	0	6.08E-29	0	Whole_Blood
ENSG00000167914	GSDMA	17	38119226	38134019	ENSG00000125538	IL1B	2	113587328	113594480	0.0066084	16.082	1.09E-07	0.01473416	0.010589	4.9177	0.0075096	rs7212938	5.02E-16	0	5.34E-44	0	Adipose_Subcutaneous
ENSG00000167914	GSDMA	17	38119226	38134019	ENSG00000125538	IL1B	2	113587328	113594480	0.0066084	16.082	1.09E-07	0.01473416	0.010589	4.9177	0.0075096	rs7212938	5.02E-16	0	5.34E-44	0	Adipose_Visceral_Omentum
ENSG00000167914	GSDMA	17	38119226	38134019	ENSG00000125538	IL1B	2	113587328	113594480	0.0066084	16.082	1.09E-07	0.01473416	0.010589	4.9177	0.0075096	rs7212938	5.02E-16	0	5.34E-44	0	Artery_Coronary
ENSG00000167914	GSDMA	17	38119226	38134019	ENSG00000125538	IL1B	2	113587328	113594480	0.0066084	16.082	1.09E-07	0.01473416	0.010589	4.9177	0.0075096	rs7212938	5.02E-16	0	5.34E-44	0	Colon_Sigmoid
ENSG00000167914	GSDMA	17	38119226	38134019	ENSG00000125538	IL1B	2	113587328	113594480	0.0066084	16.082	1.09E-07	0.01473416	0.010589	4.9177	0.0075096	rs7212938	5.02E-16	0	5.34E-44	0	Heart_Atrial_Appendage
ENSG00000167914	GSDMA	17	38119226	38134019	ENSG00000125538	IL1B	2	113587328	113594480	0.0066084	16.082	1.09E-07	0.01473416	0.010589	4.9177	0.0075096	rs7212938	5.02E-16	0	5.34E-44	0	Heart_Left_Ventricle
ENSG00000167914	GSDMA	17	38119226	38134019	ENSG00000125538	IL1B	2	113587328	113594480	0.0066084	16.082	1.09E-07	0.01473416	0.010589	4.9177	0.0075096	rs7212938	5.02E-16	0	5.34E-44	0	Thyroid
ENSG00000167914	GSDMA	17	38119226	38134019	ENSG00000125538	IL1B	2	113587328	113594480	0.0066084	16.082	1.09E-07	0.01473416	0.010589	4.9177	0.0075096	rs7212938	5.02E-16	0	5.34E-44	0	Whole_Blood
ENSG00000167914	GSDMA	17	38119226	38134019	ENSG00000125538	IL1B	2	113587328	113594480	0.0066084	16.082	1.09E-07	0.01473416	0.010589	4.9177	0.0075096	rs8078723	7.77E-27	0	6.97E-07	0.002194627	Breast_Mammary_Tissue
ENSG00000167914	GSDMA	17	38119226	38134019	ENSG00000125538	IL1B	2	113587328	113594480	0.0066084	16.082	1.09E-07	0.01473416	0.010589	4.9177	0.0075096	rs8078723	7.77E-27	0	6.97E-07	0.002194627	Esophagus_Gastroesophageal_Junction
ENSG00000167914	GSDMA	17	38119226	38134019	ENSG00000125538	IL1B	2	113587328	113594480	0.0066084	16.082	1.09E-07	0.01473416	0.010589	4.9177	0.0075096	rs4794820	2.15E-17	0	2.26E-66	0	Artery_Tibial
ENSG00000167914	GSDMA	17	38119226	38134019	ENSG00000125538	IL1B	2	113587328	113594480	0.0066084	16.082	1.09E-07	0.01473416	0.010589	4.9177	0.0075096	rs4794820	2.15E-17	0	2.26E-66	0	Cells_Transformed_fibroblasts
ENSG00000167914	GSDMA	17	38119226	38134019	ENSG00000125538	IL1B	2	113587328	113594480	0.0066084	16.082	1.09E-07	0.01473416	0.010589	4.9177	0.0075096	rs4794822	6.22E-26	0	3.02E-06	0.008756123	Breast_Mammary_Tissue
ENSG00000167914	GSDMA	17	38119226	38134019	ENSG00000125538	IL1B	2	113587328	113594480	0.0066084	16.082	1.09E-07	0.01473416	0.010589	4.9177	0.0075096	rs4794822	6.22E-26	0	3.02E-06	0.008756123	Cells_Transformed_fibroblasts
ENSG00000167914	GSDMA	17	38119226	38134019	ENSG00000125538	IL1B	2	113587328	113594480	0.0066084	16.082	1.09E-07	0.01473416	0.010589	4.9177	0.0075096	rs4794822	6.22E-26	0	3.02E-06	0.008756123	Esophagus_Gastroesophageal_Junction
ENSG00000167914	GSDMA	17	38119226	38134019	ENSG00000125538	IL1B	2	113587328	113594480	0.0066084	16.082	1.09E-07	0.01473416	0.010589	4.9177	0.0075096	rs4794822	6.22E-26	0	3.02E-06	0.008756123	Esophagus_Muscularis
ENSG00000167914	GSDMA	17	38119226	38134019	ENSG00000125538	IL1B	2	113587328	113594480	0.0066084	16.082	1.09E-07	0.01473416	0.010589	4.9177	0.0075096	rs4065321	7.99E-23	0	2.52E-12	0	Pancreas
ENSG00000137310	TCF19	6	31126319	31131992	ENSG00000101425	BPI	20	36888551	36965907	0.0093713	6.5273	1.09E-07	0.01473416	0.015213	2.017	0.050309	rs3131379	3.76E-06	0.02621495	NA	NA	Adipose_Visceral_Omentum
ENSG00000137310	TCF19	6	31126319	31131992	ENSG00000101425	BPI	20	36888551	36965907	0.0093713	6.5273	1.09E-07	0.01473416	0.015213	2.017	0.050309	rs3131379	3.76E-06	0.02621495	NA	NA	Brain_Frontal_Cortex_BA9
ENSG00000137310	TCF19	6	31126319	31131992	ENSG00000101425	BPI	20	36888551	36965907	0.0093713	6.5273	1.09E-07	0.01473416	0.015213	2.017	0.050309	rs6929796	2.96E-07	0.00278853	NA	NA	Skin_Not_Sun_Exposed_Suprapubic
ENSG00000186075	ZPBP2	17	38024417	38034149	ENSG00000072952	MRVI1	11	10594638	10715535	0.0058116	28.269	1.10E-07	0.0148551	0.0016782	1.5466	0.21396	rs3859192	5.47E-07	0.00481306	NA	NA	Testis
ENSG00000198917	C9orf114	9	131586026	131592100	ENSG00000179869	ABCA13	7	48211055	48687092	0.0078209	9.524	1.15E-07	0.01534737	0.015737	4.8925	0.0022242	rs15676	5.39E-15	0	NA	NA	Breast_Mammary_Tissue
ENSG00000205045	SLFN12L	17	33800708	33864880	ENSG00000184792	OSBP2	22	31089769	31303811	0.0093384	6.5043	1.17E-07	0.01565332	0.012012	1.5875	0.13537	rs8082605	1.36E-06	0.01074732	2.49E-31	0	Adipose_Subcutaneous
ENSG00000205045	SLFN12L	17	33800708	33864880	ENSG00000184792	OSBP2	22	31089769	31303811	0.0093384	6.5043	1.17E-07	0.01565332	0.012012	1.5875	0.13537	rs8082605	1.36E-06	0.01074732	2.49E-31	0	Adipose_Visceral_Omentum
ENSG00000205045	SLFN12L	17	33800708	33864880	ENSG00000184792	OSBP2	22	31089769	31303811	0.0093384	6.5043	1.17E-07	0.01565332	0.012012	1.5875	0.13537	rs8082605	1.36E-06	0.01074732	2.49E-31	0	Heart_Left_Ventricle
ENSG00000205045	SLFN12L	17	33800708	33864880	ENSG00000184792	OSBP2	22	31089769	31303811	0.0093384	6.5043	1.17E-07	0.01565332	0.012012	1.5875	0.13537	rs8082605	1.36E-06	0.01074732	2.49E-31	0	Nerve_Tibial
ENSG00000205045	SLFN12L	17	33800708	33864880	ENSG00000184792	OSBP2	22	31089769	31303811	0.0093384	6.5043	1.17E-07	0.01565332	0.012012	1.5875	0.13537	rs10512472	2.35E-08	0.00031703	4.96E-30	0	Pancreas
ENSG00000108278	ZNHIT3	17	34842473	34849850	ENSG00000022840	RNF10	12	120971283	121015397	0.0083456	8.1331	1.18E-07	0.01569953	0.012039	2.2324	0.049193	rs8082605	1.03E-82	0	NA	NA	Stomach
ENSG00000228008	CTD-2330K9.	3	49943495	49954370	ENSG00000175691	ZNF77	19	2933216	2944969	0.0072264	11.729	1.18E-07	0.01571195	0.0037812	0.87012	0.48128	rs2013208	7.61E-06	0.04648663	NA	NA	Artery_Tibial
ENSG00000177951	BET1L	11	167784	207428	ENSG00000120885	CLU	8	27454434	27472548	0.009794	5.9704	1.20E-07	0.01590008	0.031343	2.6768	0.0021733	rs11602954	1.24E-10	0	9.12E-206	0	Adipose_Visceral_Omentum
ENSG00000177951	BET1L	11	167784	207428	ENSG00000120885	CLU	8	27454434	27472548	0.009794	5.9704	1.20E-07	0.01590008	0.031343	2.6768	0.0021733	rs11602954	1.24E-10	0	9.12E-206	0	Artery_Aorta
ENSG00000177951	BET1L	11	167784	207428	ENSG00000120885	CLU	8	27454434	27472548	0.009794	5.9704	1.20E-07	0.01590008	0.031343	2.6768	0.0021733	rs11602954	1.24E-10	0	9.12E-206	0	Cells_Transformed_fibroblasts
ENSG00000177951	BET1L	11	167784	207428	ENSG00000120885	CLU	8	27454434	27472548	0.009794	5.9704	1.20E-07	0.01590008	0.031343	2.6768	0.0021733	rs11602954	1.24E-10	0	9.12E-206	0	Pituitary
ENSG00000177951	BET1L	11	167784	207428	ENSG00000120885	CLU	8	27454434	27472548	0.009794	5.9704	1.20E-07	0.01590008	0.031343	2.6768	0.0021733	rs11602954	1.24E-10	0	9.12E-206	0	Prostate
ENSG00000177951	BET1L	11	167784	207428	ENSG00000120885	CLU	8	27454434	27472548	0.009794	5.9704	1.20E-07	0.01590008	0.031343	2.6768	0.0021733	rs11602954	1.24E-10	0	9.12E-206	0	Small_Intestine_Terminal_Ileum
ENSG00000177951	BET1L	11	167784	207428	ENSG00000120885	CLU	8	27454434	27472548	0.009794	5.9704	1.20E-07	0.01590008	0.031343	2.6768	0.0021733	rs11602954	1.24E-10	0	9.12E-206	0	Whole_Blood
ENSG00000177951	BET1L	11	167784	207428	ENSG00000120885	CLU	8	27454434	27472548	0.009794	5.9704	1.20E-07	0.01590008	0.031343	2.6768	0.0021733	rs17655730	2.73E-13	0	2.96E-166	0	Prostate
ENSG00000177951	BET1L	11	167784	207428	ENSG00000120885	CLU	8	27454434	27472548	0.009794	5.9704	1.20E-07	0.01590008	0.031343	2.6768	0.0021733	rs505404	7.47E-15	0	3.73E-187	0	Small_Intestine_Terminal_Ileum
ENSG00000177951	BET1L	11	167784	207428	ENSG00000120885	CLU	8	27454434	27472548	0.009794	5.9704	1.20E-07	0.01590008	0.031343	2.6768	0.0021733	rs505404	7.47E-15	0	3.73E-187	0	Whole_Blood
ENSG00000188895	MSL1	17	38278551	38291643	ENSG00000162551	ALPL	1	21835858	21904905	0.011107	4.9277	1.20E-07	0.01593115	0.014447	4.4856	0.0039078	rs3894194	2.28E-14	0	NA	NA	Spleen
ENSG00000188895	MSL1	17	38278551	38291643	ENSG00000162551	ALPL	1	21835858	21904905	0.011107	4.9277	1.20E-07	0.01593115	0.014447	4.4856	0.0039078	rs7212938	1.21E-12	0	NA	NA	Thyroid
ENSG00000188895	MSL1	17	38278551	38291643	ENSG00000162551	ALPL	1	21835858	21904905	0.011107	4.9277	1.20E-07	0.01593115	0.014447	4.4856	0.0039078	rs8078723	3.63E-38	0	NA	NA	Cells_Transformed_fibroblasts
ENSG00000188895	MSL1	17	38278551	38291643	ENSG00000162551	ALPL	1	21835858	21904905	0.011107	4.9277	1.20E-07	0.01593115	0.014447	4.4856	0.0039078	rs4794820	1.24E-10	0	1.11E-05	0.031069922	Pancreas
ENSG00000163946	FAM208A	3	56658507	56717265	ENSG00000173210	ABLIM3	5	148521046	148640105	0.0097907	5.9684	1.21E-07	0.01598037	0.039116	5.3154	5.66E-06	rs12485738	8.72E-221	0	NA	NA	Esophagus_Mucosa
ENSG00000163946	FAM208A	3	56658507	56717265	ENSG00000173210	ABLIM3	5	148521046	148640105	0.0097907	5.9684	1.21E-07	0.01598037	0.039116	5.3154	5.66E-06	rs1354034	3.27170000000001e-	0	NA	NA	Heart_Atrial_Appendage
ENSG00000177666	PNPLA2	11	818902	825573	ENSG00000122786	CALD1	7	134429003	134655479	0.0083324	8.1201	1.22E-07	0.01604055	0.010075	1.5521	0.15814	rs11602954	3.38E-37	0	NA	NA	Brain_Frontal_Cortex_BA9
ENSG00000177666	PNPLA2	11	818902	825573	ENSG00000122786	CALD1	7	134429003	134655479	0.0083324	8.1201	1.22E-07	0.01604055	0.010075	1.5521	0.15814	rs17655730	3.56E-40	0	NA	NA	Brain_Frontal_Cortex_BA9
ENSG00000177666	PNPLA2	11	818902	825573	ENSG00000122786	CALD1	7	134429003	134655479	0.0083324	8.1201	1.22E-07	0.01604055	0.010075	1.5521	0.15814	rs17655730	3.56E-40	0	NA	NA	Esophagus_Muscularis
ENSG00000154760	SLFN13	17	33762115	33775856	ENSG00000171552	BCL2L1	20	30252255	30311792	0.011101	4.9251	1.22E-07	0.01604651	0.0078859	0.6021	0.84162	rs225245	2.42E-12	0	NA	NA	Liver
ENSG00000154760	SLFN13	17	33762115	33775856	ENSG00000171552	BCL2L1	20	30252255	30311792	0.011101	4.9251	1.22E-07	0.01604651	0.0078859	0.6021	0.84162	rs8082605	3.39E-06	0.02394735	2.08E-19	0	Artery_Tibial
ENSG00000154760	SLFN13	17	33762115	33775856	ENSG00000171552	BCL2L1	20	30252255	30311792	0.011101	4.9251	1.22E-07	0.01604651	0.0078859	0.6021	0.84162	rs8082605	3.39E-06	0.02394735	2.08E-19	0	Esophagus_Mucosa
ENSG00000154760	SLFN13	17	33762115	33775856	ENSG00000171552	BCL2L1	20	30252255	30311792	0.011101	4.9251	1.22E-07	0.01604651	0.0078859	0.6021	0.84162	rs8082605	3.39E-06	0.02394735	2.08E-19	0	Nerve_Tibial
ENSG00000154760	SLFN13	17	33762115	33775856	ENSG00000171552	BCL2L1	20	30252255	30311792	0.011101	4.9251	1.22E-07	0.01604651	0.0078859	0.6021	0.84162	rs8082605	3.39E-06	0.02394735	2.08E-19	0	Pituitary
ENSG00000164442	CITED2	6	139693393	139695757	ENSG00000187017	ESPN	1	6484848	6521040	0.0072098	11.702	1.23E-07	0.01617836	0.028178	8.8725	8.44E-06	rs592423	4.01E-74	0	NA	NA	Esophagus_Mucosa
ENSG00000164442	CITED2	6	139693393	139695757	ENSG00000187017	ESPN	1	6484848	6521040	0.0072098	11.702	1.23E-07	0.01617836	0.028178	8.8725	8.44E-06	rs590856	2.31E-73	0	NA	NA	Esophagus_Mucosa
ENSG00000099834	CDHR5	11	616565	626078	ENSG00000144677	CTDSPL	3	37903451	38025960	0.0088228	7.1671	1.26E-07	0.0165161	0.0034665	0.53047	0.78539	rs11602954	1.10E-48	0	NA	NA	Testis
ENSG00000099834	CDHR5	11	616565	626078	ENSG00000144677	CTDSPL	3	37903451	38025960	0.0088228	7.1671	1.26E-07	0.0165161	0.0034665	0.53047	0.78539	rs3782123	4.11E-07	0.00366492	NA	NA	Liver
ENSG00000099834	CDHR5	11	616565	626078	ENSG00000144677	CTDSPL	3	37903451	38025960	0.0088228	7.1671	1.26E-07	0.0165161	0.0034665	0.53047	0.78539	rs3782123	4.11E-07	0.00366492	NA	NA	Testis
ENSG00000168374	ARF4	3	57557090	57583947	ENSG00000154146	NRGN	11	124609829	124615878	0.0083099	8.0979	1.28E-07	0.01672905	0.02271	4.2572	0.00078341	rs17825630	1.92E-21	0	NA	NA	Artery_Tibial
ENSG00000168374	ARF4	3	57557090	57583947	ENSG00000154146	NRGN	11	124609829	124615878	0.0083099	8.0979	1.28E-07	0.01672905	0.02271	4.2572	0.00078341	rs17825630	1.92E-21	0	NA	NA	Heart_Atrial_Appendage
ENSG00000154768	C17orf50	17	34087916	34092098	ENSG00000103148	NPRL3	16	138697	188859	0.0088151	7.1607	1.28E-07	0.01673755	0.0086231	1.1357	0.33815	rs9915021	1.32E-25	0	NA	NA	Pituitary
ENSG00000108733	PEX12	17	33901814	33905882	ENSG00000085733	CTTN	11	70244510	70282690	0.0082878	8.0762	1.34E-07	0.01736409	0.012633	2.3439	0.039688	rs9915021	8.57E-07	0.00721415	NA	NA	Brain_Caudate_basal_ganglia
ENSG00000108733	PEX12	17	33901814	33905882	ENSG00000085733	CTTN	11	70244510	70282690	0.0082878	8.0762	1.34E-07	0.01736409	0.012633	2.3439	0.039688	rs9915021	8.57E-07	0.00721415	NA	NA	Breast_Mammary_Tissue
ENSG00000108733	PEX12	17	33901814	33905882	ENSG00000085733	CTTN	11	70244510	70282690	0.0082878	8.0762	1.34E-07	0.01736409	0.012633	2.3439	0.039688	rs10512472	4.18E-19	0	1.06E-12	0	Brain_Caudate_basal_ganglia
ENSG00000006125	AP2B1	17	33913918	34053436	ENSG00000187017	ESPN	1	6484848	6521040	0.0092738	6.4588	1.35E-07	0.01740033	0.0046699	0.7155	0.63718	rs10512472	5.66E-18	0	3.62E-14	0	Heart_Left_Ventricle
ENSG00000132139	GAS2L2	17	34071530	34079897	ENSG00000198959	TGM2	20	36756863	36794980	0.0097295	5.9307	1.38E-07	0.01770791	0.0074489	0.85649	0.55304	rs9915021	2.01E-09	5.61E-05	NA	NA	Small_Intestine_Terminal_Ileum
ENSG00000141150	RASL10B	17	34058668	34070540	ENSG00000187017	ESPN	1	6484848	6521040	0.0097292	5.9305	1.38E-07	0.01770791	0.0047616	0.6247	0.73578	rs9915021	2.82E-16	0	NA	NA	Cells_Transformed_fibroblasts
ENSG00000154760	SLFN13	17	33762115	33775856	ENSG00000135924	DNAJB2	2	220143989	220151622	0.011038	4.8967	1.39E-07	0.01773797	0.016154	1.2438	0.24778	rs225245	1.29E-19	0	NA	NA	Liver
ENSG00000154760	SLFN13	17	33762115	33775856	ENSG00000135924	DNAJB2	2	220143989	220151622	0.011038	4.8967	1.39E-07	0.01773797	0.016154	1.2438	0.24778	rs8082605	2.06E-11	0	2.08E-19	0	Artery_Tibial
ENSG00000154760	SLFN13	17	33762115	33775856	ENSG00000135924	DNAJB2	2	220143989	220151622	0.011038	4.8967	1.39E-07	0.01773797	0.016154	1.2438	0.24778	rs8082605	2.06E-11	0	2.08E-19	0	Esophagus_Mucosa
ENSG00000154760	SLFN13	17	33762115	33775856	ENSG00000135924	DNAJB2	2	220143989	220151622	0.011038	4.8967	1.39E-07	0.01773797	0.016154	1.2438	0.24778	rs8082605	2.06E-11	0	2.08E-19	0	Nerve_Tibial
ENSG00000154760	SLFN13	17	33762115	33775856	ENSG00000135924	DNAJB2	2	220143989	220151622	0.011038	4.8967	1.39E-07	0.01773797	0.016154	1.2438	0.24778	rs8082605	2.06E-11	0	2.08E-19	0	Pituitary
ENSG00000154760	SLFN13	17	33762115	33775856	ENSG00000135924	DNAJB2	2	220143989	220151622	0.011038	4.8967	1.39E-07	0.01773797	0.016154	1.2438	0.24778	rs10512472	6.15E-09	0.00013543	6.34E-38	0	Uterus
ENSG00000239388	ASB14	3	57310556	57326710	ENSG00000049323	LTBP1	2	33172039	33624576	0.0082742	8.0629	1.39E-07	0.01773797	0.0050775	0.93495	0.4574	rs12485738	1.13E-71	0	NA	NA	Artery_Tibial
ENSG00000239388	ASB14	3	57310556	57326710	ENSG00000049323	LTBP1	2	33172039	33624576	0.0082742	8.0629	1.39E-07	0.01773797	0.0050775	0.93495	0.4574	rs1354034	1.91E-136	0	NA	NA	Skin_Not_Sun_Exposed_Suprapubic
ENSG00000205045	SLFN12L	17	33800708	33864880	ENSG00000159335	PTMS	12	6874682	6880116	0.0092526	6.4439	1.42E-07	0.01805371	0.01037	1.3682	0.21533	rs8082605	4.68E-10	2.10E-05	2.49E-31	0	Adipose_Subcutaneous
ENSG00000205045	SLFN12L	17	33800708	33864880	ENSG00000159335	PTMS	12	6874682	6880116	0.0092526	6.4439	1.42E-07	0.01805371	0.01037	1.3682	0.21533	rs8082605	4.68E-10	2.10E-05	2.49E-31	0	Adipose_Visceral_Omentum
ENSG00000205045	SLFN12L	17	33800708	33864880	ENSG00000159335	PTMS	12	6874682	6880116	0.0092526	6.4439	1.42E-07	0.01805371	0.01037	1.3682	0.21533	rs8082605	4.68E-10	2.10E-05	2.49E-31	0	Heart_Left_Ventricle
ENSG00000205045	SLFN12L	17	33800708	33864880	ENSG00000159335	PTMS	12	6874682	6880116	0.0092526	6.4439	1.42E-07	0.01805371	0.01037	1.3682	0.21533	rs8082605	4.68E-10	2.10E-05	2.49E-31	0	Nerve_Tibial
ENSG00000205045	SLFN12L	17	33800708	33864880	ENSG00000159335	PTMS	12	6874682	6880116	0.0092526	6.4439	1.42E-07	0.01805371	0.01037	1.3682	0.21533	rs10512472	3.56E-10	2.15E-05	4.96E-30	0	Pancreas
ENSG00000163946	FAM208A	3	56658507	56717265	ENSG00000151693	ASAP2	2	9346894	9541525	0.0097089	5.918	1.44E-07	0.01829007	0.013762	1.822	0.079752	rs12485738	6.58E-35	0	NA	NA	Esophagus_Mucosa

ENSG00000163946	FAM208A	3	56658507	56717265	ENSG00000151693	ASAP2	2	9346894	9541525	0.0097089	5.918	1.44E-07	0.01829007	0.013762	1.822	0.079752	rs1354034	5.42E-66	0	NA	NA	Heart_Atrial_Appendage
ENSG00000205045	SLFN12L	17	33800708	33864880	ENSG00000136840	ST6GALNAC4	9	130670165	130679317	0.0092401	6.4352	1.46E-07	0.01842473	0.018652	2.4818	0.015816	rs8082605	1.97E-14	0	2.49E-31	0	Adipose_Subcutaneous
ENSG00000205045	SLFN12L	17	33800708	33864880	ENSG00000136840	ST6GALNAC4	9	130670165	130679317	0.0092401	6.4352	1.46E-07	0.01842473	0.018652	2.4818	0.015816	rs8082605	1.97E-14	0	2.49E-31	0	Adipose_Visceral_Omentum
ENSG00000205045	SLFN12L	17	33800708	33864880	ENSG00000136840	ST6GALNAC4	9	130670165	130679317	0.0092401	6.4352	1.46E-07	0.01842473	0.018652	2.4818	0.015816	rs8082605	1.97E-14	0	2.49E-31	0	Heart_Left_Ventricle
ENSG00000205045	SLFN12L	17	33800708	33864880	ENSG00000136840	ST6GALNAC4	9	130670165	130679317	0.0092401	6.4352	1.46E-07	0.01842473	0.018652	2.4818	0.015816	rs8082605	1.97E-14	0	2.49E-31	0	Nerve_Tibial
ENSG00000205045	SLFN12L	17	33800708	33864880	ENSG00000136840	ST6GALNAC4	9	130670165	130679317	0.0092401	6.4352	1.46E-07	0.01842473	0.018652	2.4818	0.015816	rs10512472	5.27E-17	0	4.96E-30	0	Pancreas
ENSG00000174885	NLRP6	11	278365	285359	ENSG00000204420	C6orf25	6	31691121	31694491	0.0092213	6.4219	1.52E-07	0.01907578	0.022521	2.3347	0.013317	rs11602954	2.23E-16	0	2.12E-70	0	Nerve_Tibial
ENSG00000174885	NLRP6	11	278365	285359	ENSG00000204420	C6orf25	6	31691121	31694491	0.0092213	6.4219	1.52E-07	0.01907578	0.022521	2.3347	0.013317	rs17655730	8.25E-20	0	5.43E-117	0	Artery_Aorta
ENSG00000174885	NLRP6	11	278365	285359	ENSG00000204420	C6orf25	6	31691121	31694491	0.0092213	6.4219	1.52E-07	0.01907578	0.022521	2.3347	0.013317	rs17655730	8.25E-20	0	5.43E-117	0	Nerve_Tibial
ENSG00000174885	NLRP6	11	278365	285359	ENSG00000204420	C6orf25	6	31691121	31694491	0.0092213	6.4219	1.52E-07	0.01907578	0.022521	2.3347	0.013317	rs17655730	8.25E-20	0	5.43E-117	0	Skin_Sun_Exposed_Lower_leg
ENSG00000174885	NLRP6	11	278365	285359	ENSG00000204420	C6orf25	6	31691121	31694491	0.0092213	6.4219	1.52E-07	0.01907578	0.022521	2.3347	0.013317	rs505404	9.44E-22	0	2.65E-88	0	Nerve_Tibial
ENSG00000174885	NLRP6	11	278365	285359	ENSG00000204420	C6orf25	6	31691121	31694491	0.0092213	6.4219	1.52E-07	0.01907578	0.022521	2.3347	0.013317	rs505404	9.44E-22	0	2.65E-88	0	Prostate
ENSG00000108733	PEX12	17	33901814	33905882	ENSG00000177666	PNPLA2	11	818902	825573	0.0082332	8.0226	1.52E-07	0.01910136	0.0024098	0.44255	0.81885	rs9915021	4.08E-09	0.00010556	NA	NA	Brain_Caudate_basal_ganglia
ENSG00000108733	PEX12	17	33901814	33905882	ENSG00000177666	PNPLA2	11	818902	825573	0.0082332	8.0226	1.52E-07	0.01910136	0.0024098	0.44255	0.81885	rs9915021	4.08E-09	0.00010556	NA	NA	Breast_Mammary_Tissue
ENSG00000172660	TAF15	17	34136459	34174246	ENSG00000177666	PNPLA2	11	818902	825573	0.0087226	7.085	1.57E-07	0.01960294	0.0017936	0.27401	0.9492	rs9915021	4.08E-09	0.00010556	NA	NA	Skin_Not_Sun_Exposed_Suprapubic
ENSG00000106635	BCL7B	7	72950686	72972332	ENSG00000140287	HDC	15	50534144	50558223	0.0064536	15.703	1.59E-07	0.01981792	0.0051075	2.3589	0.095092	rs17145738	8.16E-06	0.04922912	NA	NA	Adipose_Subcutaneous
ENSG00000106635	BCL7B	7	72950686	72972332	ENSG00000140287	HDC	15	50534144	50558223	0.0064536	15.703	1.59E-07	0.01981792	0.0051075	2.3589	0.095092	rs17145738	8.16E-06	0.04922912	NA	NA	Nerve_Tibial
ENSG00000106635	BCL7B	7	72950686	72972332	ENSG00000140287	HDC	15	50534144	50558223	0.0064536	15.703	1.59E-07	0.01981792	0.0051075	2.3589	0.095092	rs2240466	1.14E-06	0.00910127	NA	NA	Nerve_Tibial
ENSG00000106635	BCL7B	7	72950686	72972332	ENSG00000140287	HDC	15	50534144	50558223	0.0064536	15.703	1.59E-07	0.01981792	0.0051075	2.3589	0.095092	rs13231516	4.38E-06	0.02956957	NA	NA	Nerve_Tibial
ENSG00000106635	BCL7B	7	72950686	72972332	ENSG00000140287	HDC	15	50534144	50558223	0.0064536	15.703	1.59E-07	0.01981792	0.0051075	2.3589	0.095092	rs714052	3.32E-06	0.02351536	NA	NA	Nerve_Tibial
ENSG00000174885	NLRP6	11	278365	285359	ENSG00000005961	ITGA2B	17	42449548	42466873	0.0091719	6.3872	1.69E-07	0.02078738	0.019451	2.0101	0.035374	rs11602954	6.34E-48	0	2.12E-70	0	Nerve_Tibial
ENSG00000174885	NLRP6	11	278365	285359	ENSG00000005961	ITGA2B	17	42449548	42466873	0.0091719	6.3872	1.69E-07	0.02078738	0.019451	2.0101	0.035374	rs17655730	6.64E-43	0	5.43E-117	0	Artery_Aorta
ENSG00000174885	NLRP6	11	278365	285359	ENSG00000005961	ITGA2B	17	42449548	42466873	0.0091719	6.3872	1.69E-07	0.02078738	0.019451	2.0101	0.035374	rs17655730	6.64E-43	0	5.43E-117	0	Nerve_Tibial
ENSG00000174885	NLRP6	11	278365	285359	ENSG00000005961	ITGA2B	17	42449548	42466873	0.0091719	6.3872	1.69E-07	0.02078738	0.019451	2.0101	0.035374	rs17655730	6.64E-43	0	5.43E-117	0	Skin_Sun_Exposed_Lower_leg
ENSG00000174885	NLRP6	11	278365	285359	ENSG00000005961	ITGA2B	17	42449548	42466873	0.0091719	6.3872	1.69E-07	0.02078738	0.019451	2.0101	0.035374	rs505404	6.63E-50	0	2.65E-88	0	Nerve_Tibial
ENSG00000174885	NLRP6	11	278365	285359	ENSG00000005961	ITGA2B	17	42449548	42466873	0.0091719	6.3872	1.69E-07	0.02078738	0.019451	2.0101	0.035374	rs505404	6.63E-50	0	2.65E-88	0	Prostate
ENSG00000172716	SLFN11	17	33677324	33700720	ENSG00000102145	GATA1	X	48644962	48652718	0.011353	4.6173	1.70E-07	0.02090457	0.018765	1.4486	0.13818	rs225245	2.47E-25	0	5.77E-07	0.001884469	Cells_Transformed_fibroblasts
ENSG00000172716	SLFN11	17	33677324	33700720	ENSG00000102145	GATA1	X	48644962	48652718	0.011353	4.6173	1.70E-07	0.02090457	0.018765	1.4486	0.13818	rs8082605	5.92E-08	0.00080711	NA	NA	Adrenal_Gland
ENSG00000132139	GAS2L2	17	34071530	34079897	ENSG00000143774	GUK1	1	228327663	228336685	0.0096256	5.8667	1.72E-07	0.02107841	0.0091645	1.0556	0.39232	rs9915021	1.78E-10	0	NA	NA	Small_Intestine_Terminal_Ileum
ENSG00000008838	MED24	17	38175350	38217468	ENSG00000158089	GALNT14	2	31133333	31378068	0.0076432	9.306	1.73E-07	0.02110303	0.0054836	1.264	0.28243	rs3859192	8.66E-08	0.00102902	1.47E-40	0	Whole_Blood
ENSG00000008838	MED24	17	38175350	38217468	ENSG00000158089	GALNT14	2	31133333	31378068	0.0076432	9.306	1.73E-07	0.02110303	0.0054836	1.264	0.28243	rs8078723	1.32E-08	0.00017458	5.30E-78	0	Adipose_Subcutaneous
ENSG00000008838	MED24	17	38175350	38217468	ENSG00000158089	GALNT14	2	31133333	31378068	0.0076432	9.306	1.73E-07	0.02110303	0.0054836	1.264	0.28243	rs8078723	1.32E-08	0.00017458	5.30E-78	0	Artery_Tibial
ENSG00000008838	MED24	17	38175350	38217468	ENSG00000158089	GALNT14	2	31133333	31378068	0.0076432	9.306	1.73E-07	0.02110303	0.0054836	1.264	0.28243	rs8078723	1.32E-08	0.00017458	5.30E-78	0	Cells_Transformed_fibroblasts
ENSG00000008838	MED24	17	38175350	38217468	ENSG00000158089	GALNT14	2	31133333	31378068	0.0076432	9.306	1.73E-07	0.02110303	0.0054836	1.264	0.28243	rs8078723	1.32E-08	0.00017458	5.30E-78	0	Lung
ENSG00000008838	MED24	17	38175350	38217468	ENSG00000158089	GALNT14	2	31133333	31378068	0.0076432	9.306	1.73E-07	0.02110303	0.0054836	1.264	0.28243	rs8078723	1.32E-08	0.00017458	5.30E-78	0	Pituitary
ENSG00000008838	MED24	17	38175350	38217468	ENSG00000158089	GALNT14	2	31133333	31378068	0.0076432	9.306	1.73E-07	0.02110303	0.0054836	1.264	0.28243	rs8078723	1.32E-08	0.00017458	5.30E-78	0	Skin_Sun_Exposed_Lower_leg
ENSG00000008838	MED24	17	38175350	38217468	ENSG00000158089	GALNT14	2	31133333	31378068	0.0076432	9.306	1.73E-07	0.02110303	0.0054836	1.264	0.28243	rs8078723	1.32E-08	0.00017458	5.30E-78	0	Spleen
ENSG00000008838	MED24	17	38175350	38217468	ENSG00000158089	GALNT14	2	31133333	31378068	0.0076432	9.306	1.73E-07	0.02110303	0.0054836	1.264	0.28243	rs8078723	1.32E-08	0.00017458	5.30E-78	0	Stomach
ENSG00000008838	MED24	17	38175350	38217468	ENSG00000158089	GALNT14	2	31133333	31378068	0.0076432	9.306	1.73E-07	0.02110303	0.0054836	1.264	0.28243	rs8078723	1.32E-08	0.00017458	5.30E-78	0	Thyroid
ENSG00000008838	MED24	17	38175350	38217468	ENSG00000158089	GALNT14	2	31133333	31378068	0.0076432	9.306	1.73E-07	0.02110303	0.0054836	1.264	0.28243	rs4794822	3.64E-08	0.0005023	1.32E-79	0	Esophagus_Gastroesophageal_Junction
ENSG00000008838	MED24	17	38175350	38217468	ENSG00000158089	GALNT14	2	31133333	31378068	0.0076432	9.306	1.73E-07	0.02110303	0.0054836	1.264	0.28243	rs4794822	3.64E-08	0.0005023	1.32E-79	0	Lung
ENSG00000008838	MED24	17	38175350	38217468	ENSG00000158089	GALNT14	2	31133333	31378068	0.0076432	9.306	1.73E-07	0.02110303	0.0054836	1.264	0.28243	rs4794822	3.64E-08	0.0005023	1.32E-79	0	Spleen
ENSG00000008838	MED24	17	38175350	38217468	ENSG00000158089	GALNT14	2	31133333	31378068	0.0076432	9.306	1.73E-07	0.02110303	0.0054836	1.264	0.28243	rs4794822	3.64E-08	0.0005023	1.32E-79	0	Whole_Blood
ENSG00000008838	MED24	17	38175350	38217468	ENSG00000158089	GALNT14	2	31133333	31378068	0.0076432	9.306	1.73E-07	0.02110303	0.0054836	1.264	0.28243	rs4065321	8.45E-08	0.00103161	1.24E-42	0	Pituitary
ENSG00000154760	SLFN13	17	33762115	33775856	ENSG00000008441	NFIX	19	13106422	13209610	0.010923	4.8451	1.76E-07	0.02139365	0.011126	0.85227	0.59614	rs225245	5.48E-06	0.03555119	NA	NA	Liver
ENSG00000172660	TAF15	17	34136459	34174246	ENSG00000204613	TRIM10	6	30119722	30128711	0.0086574	7.0315	1.82E-07	0.02199977	0.013125	2.0282	0.059438	rs9915021	1.45E-07	0.00155087	NA	NA	Skin_Not_Sun_Exposed_Suprapubic
ENSG00000142082	SIRT3	11	215458	236431	ENSG00000128266	GNAZ	22	23412540	23464889	0.0070413	11.426	1.83E-07	0.02210695	0.0010385	0.23833	0.91671	rs11602954	1.35E-18	0	1.39E-80	0	Adipose_Subcutaneous
ENSG00000142082	SIRT3	11	215458	236431	ENSG00000128266	GNAZ	22	23412540	23464889	0.0070413	11.426	1.83E-07	0.02210695	0.0010385	0.23833	0.91671	rs11602954	1.35E-18	0	1.39E-80	0	Artery_Tibial
ENSG00000142082	SIRT3	11	215458	236431	ENSG00000128266	GNAZ	22	23412540	23464889	0.0070413	11.426	1.83E-07	0.02210695	0.0010385	0.23833	0.91671	rs11602954	1.35E-18	0	1.39E-80	0	Breast_Mammary_Tissue
ENSG00000142082	SIRT3	11	215458	236431	ENSG00000128266	GNAZ	22	23412540	23464889	0.0070413	11.426	1.83E-07	0.02210695	0.0010385	0.23833	0.91671	rs11602954	1.35E-18	0	1.39E-80	0	Cells_Transformed_fibroblasts
ENSG00000142082	SIRT3	11	215458	236431	ENSG00000128266	GNAZ	22	23412540	23464889	0.0070413	11.426	1.83E-07	0.02210695	0.0010385	0.23833	0.91671	rs11602954	1.35E-18	0	1.39E-80	0	Nerve_Tibial
ENSG00000142082	SIRT3	11	215458	236431	ENSG00000128266	GNAZ	22	23412540	23464889	0.0070413	11.426	1.83E-07	0.02210695	0.0010385	0.23833	0.91671	rs505404	8.89E-15	0	1.05E-94	0	Adrenal_Gland
ENSG00000142082	SIRT3	11	215458	236431	ENSG00000128266	GNAZ	22	23412540	23464889	0.0070413	11.426	1.83E-07	0.02210695	0.0010385	0.23833	0.91671	rs505404	8.89E-15	0	1.05E-94	0	Artery_Aorta
ENSG00000142082	SIRT3	11	215458	236431	ENSG00000128266	GNAZ	22	23412540	23464889	0.0070413	11.426	1.83E-07	0.02210695	0.0010385	0.23833	0.91671	rs505404	8.89E-15	0	1.05E-94	0	Lung
ENSG00000142082	SIRT3	11	215458	236431	ENSG00000128266	GNAZ	22	23412540	23464889	0.0070413	11.426	1.83E-07	0.02210695	0.0010385	0.23833	0.91671	rs505404	8.89E-15	0	1.05E-94	0	Thyroid
ENSG00000006125	AP2B1	17	33913918	34053436	ENSG00000148337	CIZ1	9	130928343	130966662	0.0091331	6.3599	1.84E-07	0.02214266	0.014945	2.3137	0.031856	rs10512472	6.84E-08	0.00086381	3.62E-14	0	Heart_Left_Ventricle
ENSG00000174885	NLRP6	11	278365	285359	ENSG00000117400	MPL	1	43803478	43818443	0.0091317	6.3589	1.84E-07	0.02219708	0.017381	1.7925	0.065794	rs11602954	1.38E-06	0.01088461	2.12E-70	0	Nerve_Tibial
ENSG00000174885	NLRP6	11	278365	285359	ENSG00000117400	MPL	1	43803478	43818443	0.0091317	6.3589	1.84E-07	0.02219708	0.017381	1.7925	0.065794	rs17655730	9.81E-10	3.97E-05	5.43E-117	0	Artery_Aorta
ENSG00000174885	NLRP6	11	278365	285359	ENSG00000117400	MPL	1	43803478	43818443	0.0091317	6.3589	1.84E-07	0.02219708	0.017381	1.7925	0.065794	rs17655730	9.81E-10	3.97E-05	5.43E-117	0	Nerve_Tibial
ENSG00000174885	NLRP6	11	278365	285359	ENSG00000117400	MPL	1	43803478	43818443	0.0091317	6.3589	1.84E-07	0.02219708	0.017381	1.7925	0.065794	rs17655730	9.81E-10	3.97E-05	5.43E-117	0	Skin_Sun_Exposed_Lower_leg
ENSG00000174885	NLRP6	11	278365	285359	ENSG00000117400	MPL	1	43803478	43818443	0.0091317	6.3589	1.84E-07	0.02219708	0.017381	1.7925	0.065794	rs505404	1.21E-10	0	2.65E-88	0	Nerve_Tibial
ENSG00000174885	NLRP6	11	278365	285359	ENSG00000117400	MPL	1	43803478	43818443	0.0091317	6.3589	1.84E-07	0.02219708	0.017381	1.7925	0.065794	rs505404	1.21E-10	0	2.65E-88	0	Prostate
ENSG00000144224	UBXN4	2	136499189	136542625	ENSG00000140968	IRF8	16	85932409	85956215	0.0086489	7.0246	1.85E-07	0.02229852	0.022817	3.5608	0.0017132	rs4954218	4.49E-09	0.0001048	NA	NA	Testis
ENSG00000141150	RASL10B	17	34058668	34070540	ENSG00000100614	PPM1A	14	60712470	60765805	0.009586	5.8423	1.87E-07	0.02245758	0.011187	1.4772	0.17167	rs9915021	6.10E-09	0.00013559	NA	NA	Cells_Transformed_fibroblasts
ENSG00000163946	FAM208A	3	56658507	56717265	ENSG00000128266	GNAZ	22	23412540	23464889	0.0095625	5.8279	1.97E-07	0.02340255	0.021893	2.9226	0.0049805	rs12485738	8.99E-47	0	NA	NA	Esophagus_Mucosa
ENSG00000163946	FAM208A	3	56658507	56717265	ENSG00000128266	GNAZ	22	23412540	23464889	0.0095625	5.8279	1.97E-07	0.02340255	0.021893	2.9226	0.0049805	rs1354034	1.89E-88	0	NA	NA	Heart_Atrial_Appendage
ENSG00000132436	FIGNL1	7	50511831	50518088	ENSG00000167992	VWCE	11	61025762	61062896	0.0055652	27.064	2.05E-07	0.02402937	0.0068523	6.3476	0.011922	rs12718598	1.11E-132	0	2.35E-199	0	Adipose_Visceral_Omentum
ENSG00000132436	FIGNL1	7	50511831	50518088	ENSG00000167992	VWCE	11	61025762	61062896	0.0055652	27.064	2.05E-07	0.02402937	0.0068523	6.3476	0.011922	rs12718598	1.11E-132	0	2.35E-199	0	Artery_Coronary
ENSG00000132436	FIGNL1	7	50511831	50518088	ENSG00000167992	VWCE	11	61025762	61062896	0.0055652	27.064	2.05E-07	0.02402937	0.0068523	6.3476	0.011922	rs12718598	1.11E-132	0	2.35E-199	0	Colon_Sigmoid
ENSG00000132436	FIGNL1	7	50511831	50518088	ENSG00000167992	VWCE	11	61025762	61062896	0.0055652	27.064	2.05E-07	0.02402937	0.0068523	6.3476	0.011922	rs12718598	1.11E-132	0	2.35E-199	0	Lung
ENSG00000132436	FIGNL1	7	50511831	50518088	ENSG00000167992	VWCE	11	61025762	61062896	0.0055652	27.064	2.05E-07	0.02402937	0.0068523	6.3476	0.011922	rs12718598	1.11E-132	0	2.35E-199	0	Pituitary
ENSG00000132436	FIGNL1	7	50511831	50518088	ENSG00000167992	VWCE	11	61025762	61062896	0.0055652	27.064	2.05E-07	0.02402937	0.0068523	6.3476	0.011922	rs12718598	1.11E-132	0	2.35E-199	0	Spleen
ENSG00000132436	FIGNL1	7	50511831	50518088	ENSG00000167992	VWCE	11	61025762	61062896	0.0055652	27.064	2.05E-07	0.02402937	0.0068523	6.3476	0.011922	rs12718598	1.11E-132	0	2.35E-199	0	Vagina
ENSG00000132436	FIGNL1	7	50511831	50518088	ENSG00000167992	VWCE	11	61025762	61062896	0.0055652	27.064	2.05E-07	0.02402937	0.0068523	6.3476	0.011922	rs12718597	2.60E-91	0	1.76E-281	0	Brain_Hippocampus
ENSG00000132436	FIGNL1	7	50511831	50518088	ENSG00000167992	VWCE	11	61025762	61062896	0.0055652	27.064	2.05E-07	0.02402937	0.0068523	6.3476	0.011922	rs12718597	2.60E-91	0	1.76E-281	0	Vagina
ENSG00000205045	SLFN12L	17	33800708	33864880	ENSG00000148337	CIZ1	9	130928343	130966662	0.0090734	6.318	2.09E-07	0.02444843	0.010547	1.3918	0.20515	rs10512472	6.84E-08	0.00086381	4.96E-30	0	Pancreas
ENSG00000131771	PPP1R1B	17	37782993	37792879	ENSG00000125538	IL1B	2	113587328	113594480	0.0099585	5.3959	2.19E-07	0.02540197	0.014533	1.4944	0.14521	rs3859192	3.70E-26	0	NA	NA	Adipose_Subcutaneous
ENSG00000131771	PPP1R1B	17	37782993	37792879	ENSG00000125538	IL1B	2	113587328	113594480	0.0099585	5.3959	2.19E-07	0.02540197	0.014533	1.4944	0.14521	rs3894194	8.02E-17	0	NA	NA	Heart_Atrial_Appendage
ENSG00000131771	PPP1R1B	17	37782993	37792879	ENSG00000125538	IL1B	2	113587328	113594480	0.0099585	5.3959	2.19E-07	0.02540197	0.014533	1.4944	0.14521	rs4794820	2.15E-17	0	NA	NA	Heart_Left_Ventricle
ENSG00000131771	PPP1R1B	17	37782993	37792879	ENSG00000125538	IL1B	2	113587328	113594480	0.0099585	5.3959	2.19E-07	0.02540197	0.014533	1.4944	0.14521	rs9303280	2.34E-11	0	NA	NA	Heart_Atrial_Appendage
ENSG00000141140	MYO19	17	34856670	34899284	ENSG00000158856	DMTN	8	21906506	21940038	0.0080668	7.8592	2.22E-07	0.02560418	NA	NA	NA	rs225245	4.74E-222	0	NA	NA	Esophagus_Muscularis
ENSG00000141741	MIEN1	17	37885409	37886014	ENSG00000146592	CREB5	7	28338940	28865511	0.0080654	7.8578	2.22E-07	0.02562726	0.0029215	0.53679	0.7485	rs7212938	2.44E-08	0.00033038	NA	NA	Testis

ENSG00000006125	AP2B1	17	33913918	34053436	ENSG00000095321	CRAT	9	131857089	131873083	0.0090377	6.2929	2.26E-07	0.025934	0.015789	2.4465	0.02366	rs10512472	7.64E-26	0	3.62E-14	0	Heart_Left_Ventricle
ENSG00000163946	FAM208A	3	56658507	56717265	ENSG00000107438	PDLIM1	10	96997329	97050781	0.0094953	5.7865	2.27E-07	0.0260451	0.017383	2.3099	0.024468	rs12485738	1.57E-84	0	NA	NA	Esophagus_Mucosa
ENSG00000163946	FAM208A	3	56658507	56717265	ENSG00000107438	PDLIM1	10	96997329	97050781	0.0094953	5.7865	2.27E-07	0.0260451	0.017383	2.3099	0.024468	rs1354034	1.35E-151	0	NA	NA	Heart_Atrial_Appendage
ENSG00000174840	PDE12	3	57542003	57552571	ENSG00000259207	ITGB3	17	45387505	45389182	0.0075218	9.1571	2.29E-07	0.02614184	0.0091547	2.8272	0.037617	rs17825630	4.73E-14	0	NA	NA	Artery_Coronary
ENSG00000174840	PDE12	3	57542003	57552571	ENSG00000259207	ITGB3	17	45387505	45389182	0.0075218	9.1571	2.29E-07	0.02614184	0.0091547	2.8272	0.037617	rs1354034	3.27170000000001e-	0	NA	NA	Brain_Putamen_basal_ganglia
ENSG00000131771	PPP1R1B	17	37782993	37792879	ENSG00000135842	FAM129A	1	184759858	184943682	0.0099229	5.3764	2.36E-07	0.02681875	0.012831	1.3171	0.22351	rs3859192	2.10E-34	0	NA	NA	Adipose_Subcutaneous
ENSG00000131771	PPP1R1B	17	37782993	37792879	ENSG00000135842	FAM129A	1	184759858	184943682	0.0099229	5.3764	2.36E-07	0.02681875	0.012831	1.3171	0.22351	rs3894194	3.63E-19	0	NA	NA	Heart_Atrial_Appendage
ENSG00000131771	PPP1R1B	17	37782993	37792879	ENSG00000135842	FAM129A	1	184759858	184943682	0.0099229	5.3764	2.36E-07	0.02681875	0.012831	1.3171	0.22351	rs4794820	1.01E-16	0	NA	NA	Heart_Left_Ventricle
ENSG00000131771	PPP1R1B	17	37782993	37792879	ENSG00000135842	FAM129A	1	184759858	184943682	0.0099229	5.3764	2.36E-07	0.02681875	0.012831	1.3171	0.22351	rs9303280	2.35E-12	0	NA	NA	Heart_Atrial_Appendage
ENSG00000188076	SCGB1C1	11	193080	194573	ENSG00000205038	PKHD1L1	8	110374706	110542559	0.0055088	26.788	2.36E-07	0.02681875	0.00045496	0.41875	0.51772	rs11602954	3.21E-08	0.00042188	3.14E-203	0	Whole_Blood
ENSG00000188076	SCGB1C1	11	193080	194573	ENSG00000205038	PKHD1L1	8	110374706	110542559	0.0055088	26.788	2.36E-07	0.02681875	0.00045496	0.41875	0.51772	rs17655730	2.44E-13	0	1.60E-163	0	Whole_Blood
ENSG00000188076	SCGB1C1	11	193080	194573	ENSG00000205038	PKHD1L1	8	110374706	110542559	0.0055088	26.788	2.36E-07	0.02681875	0.00045496	0.41875	0.51772	rs505404	1.11E-15	0	5.81E-188	0	Whole_Blood
ENSG00000205045	SLFN12L	17	33800708	33864880	ENSG00000087460	GNAS	20	57414773	57486247	0.0090111	6.2742	2.40E-07	0.02712625	0.010727	1.4158	0.19525	rs8082605	2.57E-30	0	2.49E-31	0	Adipose_Subcutaneous
ENSG00000205045	SLFN12L	17	33800708	33864880	ENSG00000087460	GNAS	20	57414773	57486247	0.0090111	6.2742	2.40E-07	0.02712625	0.010727	1.4158	0.19525	rs8082605	2.57E-30	0	2.49E-31	0	Adipose_Visceral_Omentum
ENSG00000205045	SLFN12L	17	33800708	33864880	ENSG00000087460	GNAS	20	57414773	57486247	0.0090111	6.2742	2.40E-07	0.02712625	0.010727	1.4158	0.19525	rs8082605	2.57E-30	0	2.49E-31	0	Heart_Left_Ventricle
ENSG00000205045	SLFN12L	17	33800708	33864880	ENSG00000087460	GNAS	20	57414773	57486247	0.0090111	6.2742	2.40E-07	0.02712625	0.010727	1.4158	0.19525	rs8082605	2.57E-30	0	2.49E-31	0	Nerve_Tibial
ENSG00000205045	SLFN12L	17	33800708	33864880	ENSG00000087460	GNAS	20	57414773	57486247	0.0090111	6.2742	2.40E-07	0.02712625	0.010727	1.4158	0.19525	rs10512472	2.13E-45	0	4.96E-30	0	Pancreas
ENSG00000205045	SLFN12L	17	33800708	33864880	ENSG00000154917	RAB6B	3	133553419	133614680	0.0090084	6.2723	2.41E-07	0.02723025	0.0056981	0.74828	0.63103	rs8082605	1.89E-09	5.63E-05	2.49E-31	0	Adipose_Subcutaneous
ENSG00000205045	SLFN12L	17	33800708	33864880	ENSG00000154917	RAB6B	3	133553419	133614680	0.0090084	6.2723	2.41E-07	0.02723025	0.0056981	0.74828	0.63103	rs8082605	1.89E-09	5.63E-05	2.49E-31	0	Adipose_Visceral_Omentum
ENSG00000205045	SLFN12L	17	33800708	33864880	ENSG00000154917	RAB6B	3	133553419	133614680	0.0090084	6.2723	2.41E-07	0.02723025	0.0056981	0.74828	0.63103	rs8082605	1.89E-09	5.63E-05	2.49E-31	0	Heart_Left_Ventricle
ENSG00000205045	SLFN12L	17	33800708	33864880	ENSG00000154917	RAB6B	3	133553419	133614680	0.0090084	6.2723	2.41E-07	0.02723025	0.0056981	0.74828	0.63103	rs8082605	1.89E-09	5.63E-05	2.49E-31	0	Nerve_Tibial
ENSG00000205045	SLFN12L	17	33800708	33864880	ENSG00000154917	RAB6B	3	133553419	133614680	0.0090084	6.2723	2.41E-07	0.02723025	0.0056981	0.74828	0.63103	rs10512472	1.58E-20	0	4.96E-30	0	Pancreas
ENSG00000163946	FAM208A	3	56658507	56717265	ENSG00000180354	C7orf41	7	30174426	30202378	0.0094592	5.7644	2.46E-07	0.02766523	0.020022	2.6677	0.0097717	rs12485738	9.22E-106	0	NA	NA	Esophagus_Mucosa
ENSG00000163946	FAM208A	3	56658507	56717265	ENSG00000180354	C7orf41	7	30174426	30202378	0.0094592	5.7644	2.46E-07	0.02766523	0.020022	2.6677	0.0097717	rs1354034	3.66E-180	0	NA	NA	Heart_Atrial_Appendage
ENSG00000092871	RFFL	17	33341759	33416338	ENSG00000198959	TGM2	20	36756863	36794980	0.0085209	6.9197	2.46E-07	0.02768885	0.0053425	0.61298	0.76749	rs9915021	2.01E-09	5.61E-05	NA	NA	Adipose_Subcutaneous
ENSG00000092871	RFFL	17	33341759	33416338	ENSG00000198959	TGM2	20	36756863	36794980	0.0085209	6.9197	2.46E-07	0.02768885	0.0053425	0.61298	0.76749	rs9915021	2.01E-09	5.61E-05	NA	NA	Artery_Tibial
ENSG00000092871	RFFL	17	33341759	33416338	ENSG00000090674	MCOLN1	19	7587512	7595387	0.0085186	6.9178	2.47E-07	0.02779606	0.0049879	0.5721	0.80135	rs9915021	9.31E-12	0	NA	NA	Adipose_Subcutaneous
ENSG00000092871	RFFL	17	33341759	33416338	ENSG00000090674	MCOLN1	19	7587512	7595387	0.0085186	6.9178	2.47E-07	0.02779606	0.0049879	0.5721	0.80135	rs9915021	9.31E-12	0	NA	NA	Artery_Tibial
ENSG00000142082	SIRT3	11	215458	236431	ENSG00000185245	GP1BA	17	4835592	4838325	0.0069122	11.215	2.49E-07	0.02784713	0.011592	2.6886	0.030106	rs11602954	3.88E-45	0	1.39E-80	0	Adipose_Subcutaneous
ENSG00000142082	SIRT3	11	215458	236431	ENSG00000185245	GP1BA	17	4835592	4838325	0.0069122	11.215	2.49E-07	0.02784713	0.011592	2.6886	0.030106	rs11602954	3.88E-45	0	1.39E-80	0	Artery_Tibial
ENSG00000142082	SIRT3	11	215458	236431	ENSG00000185245	GP1BA	17	4835592	4838325	0.0069122	11.215	2.49E-07	0.02784713	0.011592	2.6886	0.030106	rs11602954	3.88E-45	0	1.39E-80	0	Breast_Mammary_Tissue
ENSG00000142082	SIRT3	11	215458	236431	ENSG00000185245	GP1BA	17	4835592	4838325	0.0069122	11.215	2.49E-07	0.02784713	0.011592	2.6886	0.030106	rs11602954	3.88E-45	0	1.39E-80	0	Cells_Transformed_fibroblasts
ENSG00000142082	SIRT3	11	215458	236431	ENSG00000185245	GP1BA	17	4835592	4838325	0.0069122	11.215	2.49E-07	0.02784713	0.011592	2.6886	0.030106	rs11602954	3.88E-45	0	1.39E-80	0	Nerve_Tibial
ENSG00000142082	SIRT3	11	215458	236431	ENSG00000185245	GP1BA	17	4835592	4838325	0.0069122	11.215	2.49E-07	0.02784713	0.011592	2.6886	0.030106	rs7104764	4.37E-06	0.0294305	1.64E-91	0	Brain_Caudate_basal_ganglia
ENSG00000142082	SIRT3	11	215458	236431	ENSG00000185245	GP1BA	17	4835592	4838325	0.0069122	11.215	2.49E-07	0.02784713	0.011592	2.6886	0.030106	rs7104764	4.37E-06	0.0294305	1.64E-91	0	Colon_Transverse
ENSG00000142082	SIRT3	11	215458	236431	ENSG00000185245	GP1BA	17	4835592	4838325	0.0069122	11.215	2.49E-07	0.02784713	0.011592	2.6886	0.030106	rs7104764	4.37E-06	0.0294305	1.64E-91	0	Nerve_Tibial
ENSG00000142082	SIRT3	11	215458	236431	ENSG00000185245	GP1BA	17	4835592	4838325	0.0069122	11.215	2.49E-07	0.02784713	0.011592	2.6886	0.030106	rs7104764	4.37E-06	0.0294305	1.64E-91	0	Spleen
ENSG00000142082	SIRT3	11	215458	236431	ENSG00000185245	GP1BA	17	4835592	4838325	0.0069122	11.215	2.49E-07	0.02784713	0.011592	2.6886	0.030106	rs505404	2.82E-47	0	1.05E-94	0	Adrenal_Gland
ENSG00000142082	SIRT3	11	215458	236431	ENSG00000185245	GP1BA	17	4835592	4838325	0.0069122	11.215	2.49E-07	0.02784713	0.011592	2.6886	0.030106	rs505404	2.82E-47	0	1.05E-94	0	Artery_Aorta
ENSG00000142082	SIRT3	11	215458	236431	ENSG00000185245	GP1BA	17	4835592	4838325	0.0069122	11.215	2.49E-07	0.02784713	0.011592	2.6886	0.030106	rs505404	2.82E-47	0	1.05E-94	0	Lung
ENSG00000142082	SIRT3	11	215458	236431	ENSG00000185245	GP1BA	17	4835592	4838325	0.0069122	11.215	2.49E-07	0.02784713	0.011592	2.6886	0.030106	rs505404	2.82E-47	0	1.05E-94	0	Thyroid
ENSG00000142082	SIRT3	11	215458	236431	ENSG00000185245	GP1BA	17	4835592	4838325	0.0069122	11.215	2.49E-07	0.02784713	0.011592	2.6886	0.030106	rs3782123	3.24E-06	0.02309107	NA	NA	Brain_Frontal_Cortex_BA9
ENSG00000157500	APPL1	3	57261765	57301820	ENSG00000049323	LTBP1	2	33172039	33624576	0.0074766	9.1017	2.54E-07	0.02820429	0.0018956	0.58117	0.62746	rs12485738	1.13E-71	0	NA	NA	Brain_Anterior_cingulate_cortex_BA24
ENSG00000163946	FAM208A	3	56658507	56717265	ENSG00000163737	PF4	4	74846794	74847841	0.0094274	5.7447	2.63E-07	0.02888211	0.055076	7.6105	6.05E-09	rs12485738	2.83E-66	0	NA	NA	Esophagus_Mucosa
ENSG00000163946	FAM208A	3	56658507	56717265	ENSG00000163737	PF4	4	74846794	74847841	0.0094274	5.7447	2.63E-07	0.02888211	0.055076	7.6105	6.05E-09	rs1354034	1.41E-124	0	NA	NA	Heart_Atrial_Appendage
ENSG00000187664	HAPLN4	19	19366450	19373605	ENSG00000108309	RUNDC3A	17	42385927	42396039	0.0062464	15.195	2.64E-07	0.02894674	0.016089	3.7487	0.0049357	rs7245983	5.19E-36	0	NA	NA	Adipose_Subcutaneous
ENSG00000187664	HAPLN4	19	19366450	19373605	ENSG00000108309	RUNDC3A	17	42385927	42396039	0.0062464	15.195	2.64E-07	0.02894674	0.016089	3.7487	0.0049357	rs7245983	5.19E-36	0	NA	NA	Pituitary
ENSG00000187664	HAPLN4	19	19366450	19373605	ENSG00000108309	RUNDC3A	17	42385927	42396039	0.0062464	15.195	2.64E-07	0.02894674	0.016089	3.7487	0.0049357	rs7245983	5.19E-36	0	NA	NA	Stomach
ENSG00000187664	HAPLN4	19	19366450	19373605	ENSG00000108309	RUNDC3A	17	42385927	42396039	0.0062464	15.195	2.64E-07	0.02894674	0.016089	3.7487	0.0049357	rs2304130	1.24E-08	0.00017583	NA	NA	Nerve_Tibial
ENSG00000187664	HAPLN4	19	19366450	19373605	ENSG00000108309	RUNDC3A	17	42385927	42396039	0.0062464	15.195	2.64E-07	0.02894674	0.016089	3.7487	0.0049357	rs2304130	1.24E-08	0.00017583	NA	NA	Skin_Sun_Exposed_Lower_leg
ENSG00000187664	HAPLN4	19	19366450	19373605	ENSG00000108309	RUNDC3A	17	42385927	42396039	0.0062464	15.195	2.64E-07	0.02894674	0.016089	3.7487	0.0049357	rs2905424	2.48E-33	0	NA	NA	Adipose_Visceral_Omentum
ENSG00000187664	HAPLN4	19	19366450	19373605	ENSG00000108309	RUNDC3A	17	42385927	42396039	0.0062464	15.195	2.64E-07	0.02894674	0.016089	3.7487	0.0049357	rs10401969	3.38E-10	2.16E-05	NA	NA	Lung
ENSG00000187664	HAPLN4	19	19366450	19373605	ENSG00000108309	RUNDC3A	17	42385927	42396039	0.0062464	15.195	2.64E-07	0.02894674	0.016089	3.7487	0.0049357	rs16996148	7.43E-11	0	NA	NA	Lung
ENSG00000187664	HAPLN4	19	19366450	19373605	ENSG00000108309	RUNDC3A	17	42385927	42396039	0.0062464	15.195	2.64E-07	0.02894674	0.016089	3.7487	0.0049357	rs1064395	3.01E-09	0.00010866	NA	NA	Artery_Tibial
ENSG00000132139	GAS2L2	17	34071530	34079897	ENSG00000177666	PNPLA2	11	818902	825573	0.0094254	5.7436	2.64E-07	0.02895428	0.0082857	0.95351	0.47131	rs9915021	4.08E-09	0.00010556	NA	NA	Small_Intestine_Terminal_Ileum
ENSG00000154760	SLFN13	17	33762115	33775856	ENSG00000101335	MYL9	20	35169887	35178228	0.010721	4.7546	2.65E-07	0.02905058	0.016008	1.2323	0.25544	rs225245	1.09E-37	0	NA	NA	Liver
ENSG00000154760	SLFN13	17	33762115	33775856	ENSG00000101335	MYL9	20	35169887	35178228	0.010721	4.7546	2.65E-07	0.02905058	0.016008	1.2323	0.25544	rs8082605	3.70E-82	0	2.08E-19	0	Artery_Tibial
ENSG00000154760	SLFN13	17	33762115	33775856	ENSG00000101335	MYL9	20	35169887	35178228	0.010721	4.7546	2.65E-07	0.02905058	0.016008	1.2323	0.25544	rs8082605	3.70E-82	0	2.08E-19	0	Esophagus_Mucosa
ENSG00000154760	SLFN13	17	33762115	33775856	ENSG00000101335	MYL9	20	35169887	35178228	0.010721	4.7546	2.65E-07	0.02905058	0.016008	1.2323	0.25544	rs8082605	3.70E-82	0	2.08E-19	0	Nerve_Tibial
ENSG00000154760	SLFN13	17	33762115	33775856	ENSG00000101335	MYL9	20	35169887	35178228	0.010721	4.7546	2.65E-07	0.02905058	0.016008	1.2323	0.25544	rs8082605	3.70E-82	0	2.08E-19	0	Pituitary
ENSG00000154760	SLFN13	17	33762115	33775856	ENSG00000101335	MYL9	20	35169887	35178228	0.010721	4.7546	2.65E-07	0.02905058	0.016008	1.2323	0.25544	rs10512472	4.66E-133	0	6.34E-38	0	Uterus
ENSG00000148334	PTGES2	9	130882972	130887720	ENSG00000164821	DEFA4	8	6793344	6795860	0.0079793	7.7732	2.70E-07	0.02943894	0.007718	1.4249	0.21268	rs13289095	1.50E-34	0	NA	NA	Heart_Left_Ventricle
ENSG00000180376	CCDC66	3	56591189	56653929	ENSG00000085733	CTTN	11	70244510	70282690	0.007975	7.769	2.73E-07	0.0296772	0.0035824	0.65866	0.65495	rs17825630	1.56E-19	0	NA	NA	Brain_Anterior_cingulate_cortex_BA24
ENSG00000180376	CCDC66	3	56591189	56653929	ENSG00000085733	CTTN	11	70244510	70282690	0.007975	7.769	2.73E-07	0.0296772	0.0035824	0.65866	0.65495	rs17825630	1.56E-19	0	NA	NA	Cells_EBV-transformed_lymphocytes
ENSG00000180376	CCDC66	3	56591189	56653929	ENSG00000085733	CTTN	11	70244510	70282690	0.007975	7.769	2.73E-07	0.0296772	0.0035824	0.65866	0.65495	rs1354034	3.27170000000001e-	0	NA	NA	Brain_Cerebellar_Hemisphere
ENSG00000180376	CCDC66	3	56591189	56653929	ENSG00000085733	CTTN	11	70244510	70282690	0.007975	7.769	2.73E-07	0.0296772	0.0035824	0.65866	0.65495	rs1354034	3.27170000000001e-	0	NA	NA	Cells_EBV-transformed_lymphocytes
ENSG00000180376	CCDC66	3	56591189	56653929	ENSG00000085733	CTTN	11	70244510	70282690	0.007975	7.769	2.73E-07	0.0296772	0.0035824	0.65866	0.65495	rs1354034	3.27170000000001e-	0	NA	NA	Testis
ENSG00000108733	PEX12	17	33901814	33905882	ENSG00000187017	ESPN	1	6484848	6521040	0.0079698	7.7639	2.76E-07	0.02985435	0.0063304	1.1671	0.32346	rs9915021	2.82E-16	0	NA	NA	Brain_Caudate_basal_ganglia
ENSG00000108733	PEX12	17	33901814	33905882	ENSG00000187017	ESPN	1	6484848	6521040	0.0079698	7.7639	2.76E-07	0.02985435	0.0063304	1.1671	0.32346	rs9915021	2.82E-16	0	NA	NA	Breast_Mammary_Tissue
ENSG00000108733	PEX12	17	33901814	33905882	ENSG00000187017	ESPN	1	6484848	6521040	0.0079698	7.7639	2.76E-07	0.02985435	0.0063304	1.1671	0.32346	rs10512472	5.66E-18	0	1.06E-12	0	Brain_Caudate_basal_ganglia
ENSG00000142082	SIRT3	11	215458	236431	ENSG00000102362	SYTL4	X	99929488	99987110	0.0068598	11.13	2.81E-07	0.03023895	0.0036912	0.84935	0.49405	rs505404	4.66E-06	0.03095987	1.05E-94	0	Adrenal_Gland
ENSG00000142082	SIRT3	11	215458	236431	ENSG00000102362	SYTL4	X	99929488	99987110	0.0068598	11.13	2.81E-07	0.03023895	0.0036912	0.84935	0.49405	rs505404	4.66E-06	0.03095987	1.05E-94	0	Artery_Aorta
ENSG00000142082	SIRT3	11	215458	236431	ENSG00000102362	SYTL4	X	99929488	99987110	0.0068598	11.13	2.81E-07	0.03023895	0.0036912	0.84935	0.49405	rs505404	4.66E-06	0.03095987	1.05E-94	0	Lung
ENSG00000142082	SIRT3	11	215458	236431	ENSG00000102362	SYTL4	X	99929488	99987110	0.0068598	11.13	2.81E-07	0.03023895	0.0036912	0.84935	0.49405	rs505404	4.66E-06	0.03095987	1.05E-94	0	Thyroid
ENSG00000180376	CCDC66	3	56591189	56653929	ENSG00000259207	ITGB3	17	45387505	45389182	0.0079571	7.7514	2.84E-07	0.03039169	0.018405	3.4351	0.004417	rs17825630	4.73E-14	0	NA	NA	Brain_Anterior_cingulate_cortex_BA24
ENSG00000180376	CCDC66	3	56591189	56653929	ENSG00000259207	ITGB3	17	45387505	45389182	0.0079571	7.7514	2.84E-07	0.03039169	0.018405	3.4351	0.004417	rs17825630	4.73E-14	0	NA	NA	Cells_EBV-transformed_lymphocytes
ENSG00000180376	CCDC66	3	56591189	56653929	ENSG00000259207	ITGB3	17	45387505	45389182	0.0079571	7.7514	2.84E-07	0.03039169	0.018405	3.4351	0.004417	rs1354034	3.27170000000001e-	0	NA	NA	Brain_Cerebellar_Hemisphere
ENSG00000180376	CCDC66	3	56591189	56653929	ENSG00000259207	ITGB3	17	45387505	45389182	0.0079571	7.7514	2.84E-07	0.03039169	0.018405	3.4351	0.004417	rs1354034	3.27170000000001e-	0	NA	NA	Cells_EBV-transformed_lymphocytes
ENSG00000180376	CCDC66	3	56591189	56653929	ENSG00000259207	ITGB3	17	45387505	45389182	0.0079571	7.7514	2.84E-07	0.03039169	0.018405	3.4351	0.004417	rs1354034	3.27170000000001e-	0	NA	NA	Testis
ENSG00000101017	CD40	20	44746911	44758502	ENSG00000082293	COL19A1	6	70576463	70919679	0.0089302	6.2174	2.86E-07	0.0305321	0.01222	1.6153	0.12734	rs1569723	5.91E-13	0	1.13E-219	0	Adipose_Subcutaneous
ENSG00000101017	CD40	20	44746911	44758502	ENSG00000082293	COL19A1	6	70576463	70919679	0.0089302	6.2174	2.86E-07	0.0305321	0.01222	1.6153	0.12734	rs1569723	5.91E-13	0	1.13E-219	0	Adrenal_Gland
ENSG00000101017	CD40	20	44746911	44758502	ENSG00000082293	COL19A1	6	70576463	70919679	0.0089302	6.2174	2.86E-07	0.0305321	0.01222	1.6153	0.12734	rs1883832	2.33E-13	0	5.35E-221	0	Adipose_Subcutaneous

ENSG00000101017	CD40	20	44746911	44758502	ENSG00000082293	COL19A1	6	70576463	70919679	0.0089302	6.2174	2.86E-07	0.0305321	0.01222	1.6153	0.12734	rs1883832	2.33E-13	0	5.35E-221	0	Adrenal_Gland
ENSG00000101017	CD40	20	44746911	44758502	ENSG00000082293	COL19A1	6	70576463	70919679	0.0089302	6.2174	2.86E-07	0.0305321	0.01222	1.6153	0.12734	rs1883832	2.33E-13	0	5.35E-221	0	Breast_Mammary_Tissue
ENSG00000101017	CD40	20	44746911	44758502	ENSG00000082293	COL19A1	6	70576463	70919679	0.0089302	6.2174	2.86E-07	0.0305321	0.01222	1.6153	0.12734	rs1883832	2.33E-13	0	5.35E-221	0	Cells_Transformed_fibroblasts
ENSG00000101017	CD40	20	44746911	44758502	ENSG00000082293	COL19A1	6	70576463	70919679	0.0089302	6.2174	2.86E-07	0.0305321	0.01222	1.6153	0.12734	rs1883832	2.33E-13	0	5.35E-221	0	Esophagus_Gastroesophageal_Junction
ENSG00000101017	CD40	20	44746911	44758502	ENSG00000082293	COL19A1	6	70576463	70919679	0.0089302	6.2174	2.86E-07	0.0305321	0.01222	1.6153	0.12734	rs1883832	2.33E-13	0	5.35E-221	0	Lung
ENSG00000101017	CD40	20	44746911	44758502	ENSG00000082293	COL19A1	6	70576463	70919679	0.0089302	6.2174	2.86E-07	0.0305321	0.01222	1.6153	0.12734	rs1883832	2.33E-13	0	5.35E-221	0	Nerve_Tibial
ENSG00000101017	CD40	20	44746911	44758502	ENSG00000082293	COL19A1	6	70576463	70919679	0.0089302	6.2174	2.86E-07	0.0305321	0.01222	1.6153	0.12734	rs1883832	2.33E-13	0	5.35E-221	0	Thyroid
ENSG00000101017	CD40	20	44746911	44758502	ENSG00000082293	COL19A1	6	70576463	70919679	0.0089302	6.2174	2.86E-07	0.0305321	0.01222	1.6153	0.12734	rs6032662	1.57E-13	0	3.58E-222	0	Adrenal_Gland
ENSG00000101017	CD40	20	44746911	44758502	ENSG00000082293	COL19A1	6	70576463	70919679	0.0089302	6.2174	2.86E-07	0.0305321	0.01222	1.6153	0.12734	rs6032662	1.57E-13	0	3.58E-222	0	Lung
ENSG00000101017	CD40	20	44746911	44758502	ENSG00000082293	COL19A1	6	70576463	70919679	0.0089302	6.2174	2.86E-07	0.0305321	0.01222	1.6153	0.12734	rs6032662	1.57E-13	0	3.58E-222	0	Skin_Sun_Exposed_Lower_leg
ENSG00000101017	CD40	20	44746911	44758502	ENSG00000082293	COL19A1	6	70576463	70919679	0.0089302	6.2174	2.86E-07	0.0305321	0.01222	1.6153	0.12734	rs6032662	1.57E-13	0	3.58E-222	0	Thyroid
ENSG00000101017	CD40	20	44746911	44758502	ENSG00000082293	COL19A1	6	70576463	70919679	0.0089302	6.2174	2.86E-07	0.0305321	0.01222	1.6153	0.12734	rs6074022	1.94E-13	0	1.15E-220	0	Adrenal_Gland
ENSG00000101017	CD40	20	44746911	44758502	ENSG00000082293	COL19A1	6	70576463	70919679	0.0089302	6.2174	2.86E-07	0.0305321	0.01222	1.6153	0.12734	rs6074022	1.94E-13	0	1.15E-220	0	Lung
ENSG00000101017	CD40	20	44746911	44758502	ENSG00000082293	COL19A1	6	70576463	70919679	0.0089302	6.2174	2.86E-07	0.0305321	0.01222	1.6153	0.12734	rs6074022	1.94E-13	0	1.15E-220	0	Skin_Sun_Exposed_Lower_leg
ENSG00000101017	CD40	20	44746911	44758502	ENSG00000082293	COL19A1	6	70576463	70919679	0.0089302	6.2174	2.86E-07	0.0305321	0.01222	1.6153	0.12734	rs6074022	1.94E-13	0	1.15E-220	0	Thyroid
ENSG00000101017	CD40	20	44746911	44758502	ENSG00000082293	COL19A1	6	70576463	70919679	0.0089302	6.2174	2.86E-07	0.0305321	0.01222	1.6153	0.12734	rs4810485	1.23E-13	0	2.72E-222	0	Adrenal_Gland
ENSG00000101017	CD40	20	44746911	44758502	ENSG00000082293	COL19A1	6	70576463	70919679	0.0089302	6.2174	2.86E-07	0.0305321	0.01222	1.6153	0.12734	rs4239702	8.70E-13	0	1.37E-256	0	Adrenal_Gland
ENSG00000101017	CD40	20	44746911	44758502	ENSG00000082293	COL19A1	6	70576463	70919679	0.0089302	6.2174	2.86E-07	0.0305321	0.01222	1.6153	0.12734	rs4239702	8.70E-13	0	1.37E-256	0	Brain_Frontal_Cortex_BA9
ENSG00000101017	CD40	20	44746911	44758502	ENSG00000082293	COL19A1	6	70576463	70919679	0.0089302	6.2174	2.86E-07	0.0305321	0.01222	1.6153	0.12734	rs4239702	8.70E-13	0	1.37E-256	0	Esophagus_Gastroesophageal_Junction
ENSG00000101017	CD40	20	44746911	44758502	ENSG00000082293	COL19A1	6	70576463	70919679	0.0089302	6.2174	2.86E-07	0.0305321	0.01222	1.6153	0.12734	rs4239702	8.70E-13	0	1.37E-256	0	Skin_Not_Sun_Exposed_Suprapubic
ENSG00000101017	CD40	20	44746911	44758502	ENSG00000082293	COL19A1	6	70576463	70919679	0.0089302	6.2174	2.86E-07	0.0305321	0.01222	1.6153	0.12734	rs2425752	1.95E-11	0	6.47E-196	0	Esophagus_Gastroesophageal_Junction
ENSG00000126351	THRA	17	38214543	38248880	ENSG00000134755	DSC2	18	28645940	28682378	0.007948	7.7425	2.90E-07	0.03088446	0.0065521	0.86116	0.53686	rs3859192	2.56E-13	0	NA	NA	Muscle_Skeletal
ENSG00000172716	SLFN11	17	33677324	33700720	ENSG00000204463	BAG6	6	31606805	31620170	0.011084	4.5068	2.92E-07	0.03101653	0.016969	1.3076	0.20818	rs225245	2.41E-35	0	5.77E-07	0.001884469	Cells_Transformed_fibroblasts
ENSG00000172716	SLFN11	17	33677324	33700720	ENSG00000204463	BAG6	6	31606805	31620170	0.011084	4.5068	2.92E-07	0.03101653	0.016969	1.3076	0.20818	rs8082605	6.38E-11	0	NA	NA	Adrenal_Gland
ENSG00000177963	RIC8A	11	207511	215113	ENSG00000128266	GNAZ	22	23412540	23464889	0.007932	7.7268	3.00E-07	0.03165847	0.013619	1.5757	0.1279	rs11602954	1.35E-18	0	7.55E-62	0	Adipose_Subcutaneous
ENSG00000177963	RIC8A	11	207511	215113	ENSG00000128266	GNAZ	22	23412540	23464889	0.007932	7.7268	3.00E-07	0.03165847	0.013619	1.5757	0.1279	rs11602954	1.35E-18	0	7.55E-62	0	Artery_Aorta
ENSG00000177963	RIC8A	11	207511	215113	ENSG00000128266	GNAZ	22	23412540	23464889	0.007932	7.7268	3.00E-07	0.03165847	0.013619	1.5757	0.1279	rs11602954	1.35E-18	0	7.55E-62	0	Artery_Tibial
ENSG00000177963	RIC8A	11	207511	215113	ENSG00000128266	GNAZ	22	23412540	23464889	0.007932	7.7268	3.00E-07	0.03165847	0.013619	1.5757	0.1279	rs11602954	1.35E-18	0	7.55E-62	0	Brain_Caudate_basal_ganglia
ENSG00000177963	RIC8A	11	207511	215113	ENSG00000128266	GNAZ	22	23412540	23464889	0.007932	7.7268	3.00E-07	0.03165847	0.013619	1.5757	0.1279	rs11602954	1.35E-18	0	7.55E-62	0	Brain_Frontal_Cortex_BA9
ENSG00000177963	RIC8A	11	207511	215113	ENSG00000128266	GNAZ	22	23412540	23464889	0.007932	7.7268	3.00E-07	0.03165847	0.013619	1.5757	0.1279	rs11602954	1.35E-18	0	7.55E-62	0	Colon_Sigmoid
ENSG00000177963	RIC8A	11	207511	215113	ENSG00000128266	GNAZ	22	23412540	23464889	0.007932	7.7268	3.00E-07	0.03165847	0.013619	1.5757	0.1279	rs11602954	1.35E-18	0	7.55E-62	0	Colon_Transverse
ENSG00000177963	RIC8A	11	207511	215113	ENSG00000128266	GNAZ	22	23412540	23464889	0.007932	7.7268	3.00E-07	0.03165847	0.013619	1.5757	0.1279	rs11602954	1.35E-18	0	7.55E-62	0	Esophagus_Gastroesophageal_Junction
ENSG00000177963	RIC8A	11	207511	215113	ENSG00000128266	GNAZ	22	23412540	23464889	0.007932	7.7268	3.00E-07	0.03165847	0.013619	1.5757	0.1279	rs11602954	1.35E-18	0	7.55E-62	0	Esophagus_Mucosa
ENSG00000177963	RIC8A	11	207511	215113	ENSG00000128266	GNAZ	22	23412540	23464889	0.007932	7.7268	3.00E-07	0.03165847	0.013619	1.5757	0.1279	rs11602954	1.35E-18	0	7.55E-62	0	Esophagus_Muscularis
ENSG00000177963	RIC8A	11	207511	215113	ENSG00000128266	GNAZ	22	23412540	23464889	0.007932	7.7268	3.00E-07	0.03165847	0.013619	1.5757	0.1279	rs11602954	1.35E-18	0	7.55E-62	0	Lung
ENSG00000177963	RIC8A	11	207511	215113	ENSG00000128266	GNAZ	22	23412540	23464889	0.007932	7.7268	3.00E-07	0.03165847	0.013619	1.5757	0.1279	rs11602954	1.35E-18	0	7.55E-62	0	Muscle_Skeletal
ENSG00000177963	RIC8A	11	207511	215113	ENSG00000128266	GNAZ	22	23412540	23464889	0.007932	7.7268	3.00E-07	0.03165847	0.013619	1.5757	0.1279	rs11602954	1.35E-18	0	7.55E-62	0	Nerve_Tibial
ENSG00000177963	RIC8A	11	207511	215113	ENSG00000128266	GNAZ	22	23412540	23464889	0.007932	7.7268	3.00E-07	0.03165847	0.013619	1.5757	0.1279	rs11602954	1.35E-18	0	7.55E-62	0	Skin_Not_Sun_Exposed_Suprapubic
ENSG00000177963	RIC8A	11	207511	215113	ENSG00000128266	GNAZ	22	23412540	23464889	0.007932	7.7268	3.00E-07	0.03165847	0.013619	1.5757	0.1279	rs11602954	1.35E-18	0	7.55E-62	0	Skin_Sun_Exposed_Lower_leg
ENSG00000177963	RIC8A	11	207511	215113	ENSG00000128266	GNAZ	22	23412540	23464889	0.007932	7.7268	3.00E-07	0.03165847	0.013619	1.5757	0.1279	rs11602954	1.35E-18	0	7.55E-62	0	Thyroid
ENSG00000177963	RIC8A	11	207511	215113	ENSG00000128266	GNAZ	22	23412540	23464889	0.007932	7.7268	3.00E-07	0.03165847	0.013619	1.5757	0.1279	rs17655730	3.37E-11	0	5.10E-36	0	Adipose_Subcutaneous
ENSG00000177963	RIC8A	11	207511	215113	ENSG00000128266	GNAZ	22	23412540	23464889	0.007932	7.7268	3.00E-07	0.03165847	0.013619	1.5757	0.1279	rs17655730	3.37E-11	0	5.10E-36	0	Esophagus_Mucosa
ENSG00000177963	RIC8A	11	207511	215113	ENSG00000128266	GNAZ	22	23412540	23464889	0.007932	7.7268	3.00E-07	0.03165847	0.013619	1.5757	0.1279	rs17655730	3.37E-11	0	5.10E-36	0	Esophagus_Muscularis
ENSG00000177963	RIC8A	11	207511	215113	ENSG00000128266	GNAZ	22	23412540	23464889	0.007932	7.7268	3.00E-07	0.03165847	0.013619	1.5757	0.1279	rs17655730	3.37E-11	0	5.10E-36	0	Heart_Left_Ventricle
ENSG00000177963	RIC8A	11	207511	215113	ENSG00000128266	GNAZ	22	23412540	23464889	0.007932	7.7268	3.00E-07	0.03165847	0.013619	1.5757	0.1279	rs17655730	3.37E-11	0	5.10E-36	0	Ovary
ENSG00000177963	RIC8A	11	207511	215113	ENSG00000128266	GNAZ	22	23412540	23464889	0.007932	7.7268	3.00E-07	0.03165847	0.013619	1.5757	0.1279	rs17655730	3.37E-11	0	5.10E-36	0	Pancreas
ENSG00000177963	RIC8A	11	207511	215113	ENSG00000128266	GNAZ	22	23412540	23464889	0.007932	7.7268	3.00E-07	0.03165847	0.013619	1.5757	0.1279	rs17655730	3.37E-11	0	5.10E-36	0	Skin_Not_Sun_Exposed_Suprapubic
ENSG00000177963	RIC8A	11	207511	215113	ENSG00000128266	GNAZ	22	23412540	23464889	0.007932	7.7268	3.00E-07	0.03165847	0.013619	1.5757	0.1279	rs505404	8.89E-15	0	3.66E-47	0	Artery_Aorta
ENSG00000177963	RIC8A	11	207511	215113	ENSG00000128266	GNAZ	22	23412540	23464889	0.007932	7.7268	3.00E-07	0.03165847	0.013619	1.5757	0.1279	rs505404	8.89E-15	0	3.66E-47	0	Thyroid
ENSG00000108733	PEX12	17	33901814	33905882	ENSG00000204613	TRIM10	6	30119722	30128711	0.0079242	7.7191	3.06E-07	0.03207578	0.0097881	1.8109	0.10808	rs9915021	1.45E-07	0.00155087	NA	NA	Brain_Caudate_basal_ganglia
ENSG00000108733	PEX12	17	33901814	33905882	ENSG00000204613	TRIM10	6	30119722	30128711	0.0079242	7.7191	3.06E-07	0.03207578	0.0097881	1.8109	0.10808	rs9915021	1.45E-07	0.00155087	NA	NA	Breast_Mammary_Tissue
ENSG00000108733	PEX12	17	33901814	33905882	ENSG00000204613	TRIM10	6	30119722	30128711	0.0079242	7.7191	3.06E-07	0.03207578	0.0097881	1.8109	0.10808	rs10512472	1.74E-11	0	1.06E-12	0	Brain_Caudate_basal_ganglia
ENSG00000001617	SEMA3F	3	50192478	50226508	ENSG00000175691	ZNF77	19	2933216	2944969	0.0079235	7.7184	3.06E-07	0.03210305	0.0063667	1.1739	0.32007	rs2013208	7.61E-06	0.04648663	NA	NA	Nerve_Tibial
ENSG00000172660	TAF15	17	34136459	34174246	ENSG00000120885	CLU	8	27454434	27472548	0.008418	6.8354	3.09E-07	0.03229805	0.0097733	1.5051	0.17323	rs9915021	2.20E-17	0	NA	NA	Skin_Not_Sun_Exposed_Suprapubic
ENSG00000204444	APOM	6	31623248	31625987	ENSG00000101425	BPI	20	36888551	36965907	0.0084133	6.8316	3.12E-07	0.03255028	0.022829	3.5628	0.0017049	rs3131379	3.76E-06	0.02621495	3.41E-17	0	Cells_Transformed_fibroblasts
ENSG00000204444	APOM	6	31623248	31625987	ENSG00000101425	BPI	20	36888551	36965907	0.0084133	6.8316	3.12E-07	0.03255028	0.022829	3.5628	0.0017049	rs3131379	3.76E-06	0.02621495	3.41E-17	0	Colon_Transverse
ENSG00000204444	APOM	6	31623248	31625987	ENSG00000101425	BPI	20	36888551	36965907	0.0084133	6.8316	3.12E-07	0.03255028	0.022829	3.5628	0.0017049	rs3131379	3.76E-06	0.02621495	3.41E-17	0	Prostate
ENSG00000204444	APOM	6	31623248	31625987	ENSG00000101425	BPI	20	36888551	36965907	0.0084133	6.8316	3.12E-07	0.03255028	0.022829	3.5628	0.0017049	rs3131379	3.76E-06	0.02621495	3.41E-17	0	Stomach
ENSG00000204444	APOM	6	31623248	31625987	ENSG00000101425	BPI	20	36888551	36965907	0.0084133	6.8316	3.12E-07	0.03255028	0.022829	3.5628	0.0017049	rs3131379	3.76E-06	0.02621495	3.41E-17	0	Testis
ENSG00000006125	AP2B1	17	33913918	34053436	ENSG00000183508	FAM46C	1	118148556	118170994	0.0088751	6.1786	3.22E-07	0.03326444	0.012151	1.8758	0.082085	rs10512472	6.42E-12	0	3.62E-14	0	Heart_Left_Ventricle
ENSG00000172123	SLFN12	17	33738079	33760302	ENSG00000166169	POLL	10	103338639	103346957	0.012221	3.9774	3.23E-07	0.03333183	0.013958	2.1588	0.044816	rs225245	1.93E-71	0	NA	NA	Cells_EBV-transformed_lymphocytes
ENSG00000172123	SLFN12	17	33738079	33760302	ENSG00000166169	POLL	10	103338639	103346957	0.012221	3.9774	3.23E-07	0.03333183	0.013958	2.1588	0.044816	rs9915021	3.09E-30	0	NA	NA	Breast_Mammary_Tissue
ENSG00000163946	FAM208A	3	56658507	56717265	ENSG00000166333	ILK	11	6624961	6631511	0.0093184	5.6778	3.32E-07	0.03409332	0.01827	2.4299	0.01806	rs12485738	1.99E-121	0	NA	NA	Esophagus_Mucosa
ENSG00000163946	FAM208A	3	56658507	56717265	ENSG00000166333	ILK	11	6624961	6631511	0.0093184	5.6778	3.32E-07	0.03409332	0.01827	2.4299	0.01806	rs1354034	1.40E-206	0	NA	NA	Heart_Atrial_Appendage
ENSG00000142082	SIRT3	11	215458	236431	ENSG00000164116	GUCY1A3	4	156587863	156653501	0.0067812	11.001	3.39E-07	0.03465031	0.025069	5.8949	0.00010995	rs11602954	3.16E-25	0	1.39E-80	0	Adipose_Subcutaneous
ENSG00000142082	SIRT3	11	215458	236431	ENSG00000164116	GUCY1A3	4	156587863	156653501	0.0067812	11.001	3.39E-07	0.03465031	0.025069	5.8949	0.00010995	rs11602954	3.16E-25	0	1.39E-80	0	Artery_Tibial
ENSG00000142082	SIRT3	11	215458	236431	ENSG00000164116	GUCY1A3	4	156587863	156653501	0.0067812	11.001	3.39E-07	0.03465031	0.025069	5.8949	0.00010995	rs11602954	3.16E-25	0	1.39E-80	0	Breast_Mammary_Tissue
ENSG00000142082	SIRT3	11	215458	236431	ENSG00000164116	GUCY1A3	4	156587863	156653501	0.0067812	11.001	3.39E-07	0.03465031	0.025069	5.8949	0.00010995	rs11602954	3.16E-25	0	1.39E-80	0	Cells_Transformed_fibroblasts
ENSG00000142082	SIRT3	11	215458	236431	ENSG00000164116	GUCY1A3	4	156587863	156653501	0.0067812	11.001	3.39E-07	0.03465031	0.025069	5.8949	0.00010995	rs11602954	3.16E-25	0	1.39E-80	0	Nerve_Tibial
ENSG00000142082	SIRT3	11	215458	236431	ENSG00000164116	GUCY1A3	4	156587863	156653501	0.0067812	11.001	3.39E-07	0.03465031	0.025069	5.8949	0.00010995	rs505404	5.62E-29	0	1.05E-94	0	Adrenal_Gland
ENSG00000142082	SIRT3	11	215458	236431	ENSG00000164116	GUCY1A3	4	156587863	156653501	0.0067812	11.001	3.39E-07	0.03465031	0.025069	5.8949	0.00010995	rs505404	5.62E-29	0	1.05E-94	0	Artery_Aorta
ENSG00000142082	SIRT3	11	215458	236431	ENSG00000164116	GUCY1A3	4	156587863	156653501	0.0067812	11.001	3.39E-07	0.03465031	0.025069	5.8949	0.00010995	rs505404	5.62E-29	0	1.05E-94	0	Lung
ENSG00000142082	SIRT3	11	215458	236431	ENSG00000164116	GUCY1A3	4	156587863	156653501	0.0067812	11.001	3.39E-07	0.03465031	0.025069	5.8949	0.00010995	rs505404	5.62E-29	0	1.05E-94	0	Thyroid
ENSG00000168374	ARF4	3	57557090	57583947	ENSG00000166091	CMTM5	14	23846017	23848981	0.0078635	7.6595	3.51E-07	0.03552537	0.056114	10.891	3.39E-10	rs17825630	1.22E-20	0	NA	NA	Artery_Tibial
ENSG00000168374	ARF4	3	57557090	57583947	ENSG00000166091	CMTM5	14	23846017	23848981	0.0078635	7.6595	3.51E-07	0.03552537	0.056114	10.891	3.39E-10	rs17825630	1.22E-20	0	NA	NA	Heart_Atrial_Appendage
ENSG00000099849	RASSF7	11	560404	564021	ENSG00000185245	GP1BA	17	4835592	4838325	0.008831	6.1477	3.54E-07	0.03582479	0.016945	2.2507	0.02838	rs505404	2.82E-47	0	NA	NA	Brain_Frontal_Cortex_BA9
ENSG00000099849	RASSF7	11	560404	564021	ENSG00000185245	GP1BA	17	4835592	4838325	0.008831	6.1477	3.54E-07	0.03582479	0.016945	2.2507	0.02838	rs3782123	3.24E-06	0.02309107	NA	NA	Whole_Blood
ENSG00000177951	BET1L	11	167784	207428	ENSG00000204420	C6orf25	6	31691121	31694491	0.0092744	5.6507	3.64E-07	0.03643888	0.028543	2.4306	0.0054996	rs11602954	2.23E-16	0	9.12E-206	0	Adipose_Visceral_Omentum
ENSG00000177951	BET1L	11	167784	207428	ENSG00000204420	C6orf25	6	31691121	31694491	0.0092744	5.6507	3.64E-07	0.03643888	0.028543	2.4306	0.0054996	rs11602954	2.23E-16	0	9.12E-206	0	Artery_Aorta
ENSG00000177951	BET1L	11	167784	207428	ENSG00000204420	C6orf25	6	31691121	31694491	0.0092744	5.6507	3.64E-07	0.03643888	0.028543	2.4306	0.0054996	rs11602954	2.23E-16	0	9.12E-206	0	Cells_Transformed_fibroblasts
ENSG00000177951	BET1L	11	167784	207428	ENSG00000204420	C6orf25	6	31691121	31694491	0.0092744	5.6507	3.64E-07	0.03643888	0.028543	2.4306	0.0054996	rs11602954	2.23E-16	0	9.12E-206	0	Pituitary
ENSG00000177951	BET1L	11	167784	207428	ENSG00000204420	C6orf25	6	31691121	31694491	0.0092744	5.6507	3.64E-07	0.03643888	0.028543	2.4306	0.0054996	rs11602954	2.23E-16	0	9.12E-206	0	Prostate

ENSG00000177951	BET1L	11	167784	207428	ENSG00000204420	C6orf25	6	31691121	31694491	0.0092744	5.6507	3.64E-07	0.03643888	0.028543	2.4306	0.0054996	rs11602954	2.23E-16	0	9.12E-206	0	Small_Intestine_Terminal_Ileum
ENSG00000177951	BET1L	11	167784	207428	ENSG00000204420	C6orf25	6	31691121	31694491	0.0092744	5.6507	3.64E-07	0.03643888	0.028543	2.4306	0.0054996	rs11602954	2.23E-16	0	9.12E-206	0	Whole_Blood
ENSG00000177951	BET1L	11	167784	207428	ENSG00000204420	C6orf25	6	31691121	31694491	0.0092744	5.6507	3.64E-07	0.03643888	0.028543	2.4306	0.0054996	rs17655730	8.25E-20	0	2.96E-166	0	Prostate
ENSG00000177951	BET1L	11	167784	207428	ENSG00000204420	C6orf25	6	31691121	31694491	0.0092744	5.6507	3.64E-07	0.03643888	0.028543	2.4306	0.0054996	rs505404	9.44E-22	0	3.73E-187	0	Small_Intestine_Terminal_Ileum
ENSG00000177951	BET1L	11	167784	207428	ENSG00000204420	C6orf25	6	31691121	31694491	0.0092744	5.6507	3.64E-07	0.03643888	0.028543	2.4306	0.0054996	rs505404	9.44E-22	0	3.73E-187	0	Whole_Blood
ENSG00000106635	BCL7B	7	72950686	72972332	ENSG00000163751	CPA3	3	148583043	148614983	0.0061112	14.865	3.67E-07	0.0365875	0.0068965	3.191	0.041588	rs17145738	1.15E-06	0.00915734	NA	NA	Adipose_Subcutaneous
ENSG00000106635	BCL7B	7	72950686	72972332	ENSG00000163751	CPA3	3	148583043	148614983	0.0061112	14.865	3.67E-07	0.0365875	0.0068965	3.191	0.041588	rs17145738	1.15E-06	0.00915734	NA	NA	Nerve_Tibial
ENSG00000106635	BCL7B	7	72950686	72972332	ENSG00000163751	CPA3	3	148583043	148614983	0.0061112	14.865	3.67E-07	0.0365875	0.0068965	3.191	0.041588	rs2240466	6.70E-08	0.00085262	NA	NA	Nerve_Tibial
ENSG00000106635	BCL7B	7	72950686	72972332	ENSG00000163751	CPA3	3	148583043	148614983	0.0061112	14.865	3.67E-07	0.0365875	0.0068965	3.191	0.041588	rs13231516	5.87E-08	0.00080756	NA	NA	Nerve_Tibial
ENSG00000106635	BCL7B	7	72950686	72972332	ENSG00000163751	CPA3	3	148583043	148614983	0.0061112	14.865	3.67E-07	0.0365875	0.0068965	3.191	0.041588	rs714052	5.04E-08	0.00069522	NA	NA	Nerve_Tibial
ENSG00000108306	FBXL20	17	37415384	37558776	ENSG00000137757	CASP5	11	104864962	104893895	0.0078408	7.6372	3.69E-07	0.0367873	0.0082336	1.5209	0.1806	rs12946510	9.70E-07	0.00794914	NA	NA	Brain_Nucleus_accumbens_basal_ganglia
ENSG00000108306	FBXL20	17	37415384	37558776	ENSG00000137757	CASP5	11	104864962	104893895	0.0078408	7.6372	3.69E-07	0.0367873	0.0082336	1.5209	0.1806	rs7212938	5.95E-08	0.00080678	NA	NA	Brain_Nucleus_accumbens_basal_ganglia
ENSG00000177951	BET1L	11	167784	207428	ENSG00000164116	GUCY1A3	4	156587863	156653501	0.0092649	5.6448	3.72E-07	0.03699344	0.030556	2.6074	0.002832	rs11602954	3.16E-25	0	9.12E-206	0	Adipose_Visceral_Omentum
ENSG00000177951	BET1L	11	167784	207428	ENSG00000164116	GUCY1A3	4	156587863	156653501	0.0092649	5.6448	3.72E-07	0.03699344	0.030556	2.6074	0.002832	rs11602954	3.16E-25	0	9.12E-206	0	Artery_Aorta
ENSG00000177951	BET1L	11	167784	207428	ENSG00000164116	GUCY1A3	4	156587863	156653501	0.0092649	5.6448	3.72E-07	0.03699344	0.030556	2.6074	0.002832	rs11602954	3.16E-25	0	9.12E-206	0	Cells_Transformed_fibroblasts
ENSG00000177951	BET1L	11	167784	207428	ENSG00000164116	GUCY1A3	4	156587863	156653501	0.0092649	5.6448	3.72E-07	0.03699344	0.030556	2.6074	0.002832	rs11602954	3.16E-25	0	9.12E-206	0	Pituitary
ENSG00000177951	BET1L	11	167784	207428	ENSG00000164116	GUCY1A3	4	156587863	156653501	0.0092649	5.6448	3.72E-07	0.03699344	0.030556	2.6074	0.002832	rs11602954	3.16E-25	0	9.12E-206	0	Prostate
ENSG00000177951	BET1L	11	167784	207428	ENSG00000164116	GUCY1A3	4	156587863	156653501	0.0092649	5.6448	3.72E-07	0.03699344	0.030556	2.6074	0.002832	rs11602954	3.16E-25	0	9.12E-206	0	Small_Intestine_Terminal_Ileum
ENSG00000177951	BET1L	11	167784	207428	ENSG00000164116	GUCY1A3	4	156587863	156653501	0.0092649	5.6448	3.72E-07	0.03699344	0.030556	2.6074	0.002832	rs11602954	3.16E-25	0	9.12E-206	0	Whole_Blood
ENSG00000177951	BET1L	11	167784	207428	ENSG00000164116	GUCY1A3	4	156587863	156653501	0.0092649	5.6448	3.72E-07	0.03699344	0.030556	2.6074	0.002832	rs17655730	2.28E-25	0	2.96E-166	0	Prostate
ENSG00000177951	BET1L	11	167784	207428	ENSG00000164116	GUCY1A3	4	156587863	156653501	0.0092649	5.6448	3.72E-07	0.03699344	0.030556	2.6074	0.002832	rs505404	5.62E-29	0	3.73E-187	0	Small_Intestine_Terminal_Ileum
ENSG00000177951	BET1L	11	167784	207428	ENSG00000164116	GUCY1A3	4	156587863	156653501	0.0092649	5.6448	3.72E-07	0.03699344	0.030556	2.6074	0.002832	rs505404	5.62E-29	0	3.73E-187	0	Whole_Blood
ENSG00000205045	SLFN12L	17	33800708	33864880	ENSG00000124491	F13A1	6	6144318	6321246	0.0088072	6.131	3.73E-07	0.03707891	0.0031377	0.41098	0.89586	rs8082605	1.35E-14	0	2.49E-31	0	Adipose_Subcutaneous
ENSG00000205045	SLFN12L	17	33800708	33864880	ENSG00000124491	F13A1	6	6144318	6321246	0.0088072	6.131	3.73E-07	0.03707891	0.0031377	0.41098	0.89586	rs8082605	1.35E-14	0	2.49E-31	0	Adipose_Visceral_Omentum
ENSG00000205045	SLFN12L	17	33800708	33864880	ENSG00000124491	F13A1	6	6144318	6321246	0.0088072	6.131	3.73E-07	0.03707891	0.0031377	0.41098	0.89586	rs8082605	1.35E-14	0	2.49E-31	0	Heart_Left_Ventricle
ENSG00000205045	SLFN12L	17	33800708	33864880	ENSG00000124491	F13A1	6	6144318	6321246	0.0088072	6.131	3.73E-07	0.03707891	0.0031377	0.41098	0.89586	rs8082605	1.35E-14	0	2.49E-31	0	Nerve_Tibial
ENSG00000205045	SLFN12L	17	33800708	33864880	ENSG00000124491	F13A1	6	6144318	6321246	0.0088072	6.131	3.73E-07	0.03707891	0.0031377	0.41098	0.89586	rs10512472	3.64E-32	0	4.96E-30	0	Pancreas
ENSG00000168374	ARF4	3	57557090	57583947	ENSG00000151693	ASAP2	2	9346894	9541525	0.0078345	7.6311	3.74E-07	0.03716701	0.0097046	1.7953	0.11118	rs17825630	1.03E-06	0.00841761	NA	NA	Artery_Tibial
ENSG00000168374	ARF4	3	57557090	57583947	ENSG00000151693	ASAP2	2	9346894	9541525	0.0078345	7.6311	3.74E-07	0.03716701	0.0097046	1.7953	0.11118	rs17825630	1.03E-06	0.00841761	NA	NA	Heart_Atrial_Appendage
ENSG00000006125	AP2B1	17	33913918	34053436	ENSG00000179526	SHARPIN	8	145153536	145159172	0.008801	6.1266	3.78E-07	0.03749013	0.017702	2.7482	0.011862	rs10512472	2.01E-17	0	3.62E-14	0	Heart_Left_Ventricle
ENSG00000205045	SLFN12L	17	33800708	33864880	ENSG00000256269	HMBS	11	118955576	118964259	0.0087956	6.1228	3.82E-07	0.0377204	0.0037144	0.48681	0.8446	rs8082605	4.57E-09	0.00010464	2.49E-31	0	Adipose_Subcutaneous
ENSG00000205045	SLFN12L	17	33800708	33864880	ENSG00000256269	HMBS	11	118955576	118964259	0.0087956	6.1228	3.82E-07	0.0377204	0.0037144	0.48681	0.8446	rs8082605	4.57E-09	0.00010464	2.49E-31	0	Adipose_Visceral_Omentum
ENSG00000205045	SLFN12L	17	33800708	33864880	ENSG00000256269	HMBS	11	118955576	118964259	0.0087956	6.1228	3.82E-07	0.0377204	0.0037144	0.48681	0.8446	rs8082605	4.57E-09	0.00010464	2.49E-31	0	Heart_Left_Ventricle
ENSG00000205045	SLFN12L	17	33800708	33864880	ENSG00000256269	HMBS	11	118955576	118964259	0.0087956	6.1228	3.82E-07	0.0377204	0.0037144	0.48681	0.8446	rs8082605	4.57E-09	0.00010464	2.49E-31	0	Nerve_Tibial
ENSG00000205045	SLFN12L	17	33800708	33864880	ENSG00000256269	HMBS	11	118955576	118964259	0.0087956	6.1228	3.82E-07	0.0377204	0.0037144	0.48681	0.8446	rs10512472	1.67E-17	0	4.96E-30	0	Pancreas
ENSG00000186075	ZPBP2	17	38024417	38034149	ENSG00000110080	ST3GAL4	11	126225535	126310239	0.0053032	25.783	3.96E-07	0.03872727	0.0021759	2.0062	0.15699	rs3859192	1.04E-10	0	NA	NA	Testis
ENSG00000186075	ZPBP2	17	38024417	38034149	ENSG00000110080	ST3GAL4	11	126225535	126310239	0.0053032	25.783	3.96E-07	0.03872727	0.0021759	2.0062	0.15699	rs4794820	6.62E-06	0.04163105	NA	NA	Testis
ENSG00000172123	SLFN12	17	33738079	33760302	ENSG00000167992	VWCE	11	61025762	61062896	0.012114	3.9421	3.97E-07	0.03872727	0.0047535	0.72837	0.62683	rs225245	4.65E-96	0	NA	NA	Cells_EBV-transformed_lymphocytes
ENSG00000172123	SLFN12	17	33738079	33760302	ENSG00000167992	VWCE	11	61025762	61062896	0.012114	3.9421	3.97E-07	0.03872727	0.0047535	0.72837	0.62683	rs9915021	1.45E-41	0	NA	NA	Breast_Mammary_Tissue
ENSG00000099849	RASSF7	11	560404	564021	ENSG00000174175	SELP	1	169558087	169599431	0.0087779	6.1104	3.97E-07	0.03872727	0.0029109	0.38119	0.91362	rs505404	2.87E-40	0	NA	NA	Brain_Frontal_Cortex_BA9
ENSG00000180376	CCDC66	3	56591189	56653929	ENSG00000161911	TREML1	6	41117080	41122075	0.0077991	7.5963	4.05E-07	0.03921783	0.012473	2.3139	0.042057	rs17825630	1.16E-19	0	NA	NA	Brain_Anterior_cingulate_cortex_BA24
ENSG00000180376	CCDC66	3	56591189	56653929	ENSG00000161911	TREML1	6	41117080	41122075	0.0077991	7.5963	4.05E-07	0.03921783	0.012473	2.3139	0.042057	rs17825630	1.16E-19	0	NA	NA	Cells_EBV-transformed_lymphocytes
ENSG00000180376	CCDC66	3	56591189	56653929	ENSG00000161911	TREML1	6	41117080	41122075	0.0077991	7.5963	4.05E-07	0.03921783	0.012473	2.3139	0.042057	rs1354034	3.27170000000001e-	0	NA	NA	Brain_Cerebellar_Hemisphere
ENSG00000180376	CCDC66	3	56591189	56653929	ENSG00000161911	TREML1	6	41117080	41122075	0.0077991	7.5963	4.05E-07	0.03921783	0.012473	2.3139	0.042057	rs1354034	3.27170000000001e-	0	NA	NA	Cells_EBV-transformed_lymphocytes
ENSG00000180376	CCDC66	3	56591189	56653929	ENSG00000161911	TREML1	6	41117080	41122075	0.0077991	7.5963	4.05E-07	0.03921783	0.012473	2.3139	0.042057	rs1354034	3.27170000000001e-	0	NA	NA	Testis
ENSG00000142082	SIRT3	11	215458	236431	ENSG00000117400	MPL	1	43803478	43818443	0.0067021	10.872	4.09E-07	0.0393653	0.0038327	0.88203	0.47406	rs11602954	1.38E-06	0.01088461	1.39E-80	0	Adipose_Subcutaneous
ENSG00000142082	SIRT3	11	215458	236431	ENSG00000117400	MPL	1	43803478	43818443	0.0067021	10.872	4.09E-07	0.0393653	0.0038327	0.88203	0.47406	rs11602954	1.38E-06	0.01088461	1.39E-80	0	Artery_Tibial
ENSG00000142082	SIRT3	11	215458	236431	ENSG00000117400	MPL	1	43803478	43818443	0.0067021	10.872	4.09E-07	0.0393653	0.0038327	0.88203	0.47406	rs11602954	1.38E-06	0.01088461	1.39E-80	0	Breast_Mammary_Tissue
ENSG00000142082	SIRT3	11	215458	236431	ENSG00000117400	MPL	1	43803478	43818443	0.0067021	10.872	4.09E-07	0.0393653	0.0038327	0.88203	0.47406	rs11602954	1.38E-06	0.01088461	1.39E-80	0	Cells_Transformed_fibroblasts
ENSG00000142082	SIRT3	11	215458	236431	ENSG00000117400	MPL	1	43803478	43818443	0.0067021	10.872	4.09E-07	0.0393653	0.0038327	0.88203	0.47406	rs11602954	1.38E-06	0.01088461	1.39E-80	0	Nerve_Tibial
ENSG00000142082	SIRT3	11	215458	236431	ENSG00000117400	MPL	1	43803478	43818443	0.0067021	10.872	4.09E-07	0.0393653	0.0038327	0.88203	0.47406	rs505404	1.21E-10	0	1.05E-94	0	Adrenal_Gland
ENSG00000142082	SIRT3	11	215458	236431	ENSG00000117400	MPL	1	43803478	43818443	0.0067021	10.872	4.09E-07	0.0393653	0.0038327	0.88203	0.47406	rs505404	1.21E-10	0	1.05E-94	0	Artery_Aorta
ENSG00000142082	SIRT3	11	215458	236431	ENSG00000117400	MPL	1	43803478	43818443	0.0067021	10.872	4.09E-07	0.0393653	0.0038327	0.88203	0.47406	rs505404	1.21E-10	0	1.05E-94	0	Lung
ENSG00000142082	SIRT3	11	215458	236431	ENSG00000117400	MPL	1	43803478	43818443	0.0067021	10.872	4.09E-07	0.0393653	0.0038327	0.88203	0.47406	rs505404	1.21E-10	0	1.05E-94	0	Thyroid
ENSG00000108733	PEX12	17	33901814	33905882	ENSG00000168497	SDPR	2	192699028	192711981	0.0077936	7.5909	4.10E-07	0.03949075	0.0092658	1.7134	0.12884	rs9915021	1.02E-10	0	NA	NA	Brain_Caudate_basal_ganglia
ENSG00000108733	PEX12	17	33901814	33905882	ENSG00000168497	SDPR	2	192699028	192711981	0.0077936	7.5909	4.10E-07	0.03949075	0.0092658	1.7134	0.12884	rs9915021	1.02E-10	0	NA	NA	Breast_Mammary_Tissue
ENSG00000108733	PEX12	17	33901814	33905882	ENSG00000168497	SDPR	2	192699028	192711981	0.0077936	7.5909	4.10E-07	0.03949075	0.0092658	1.7134	0.12884	rs10512472	7.94E-26	0	1.06E-12	0	Brain_Caudate_basal_ganglia
ENSG00000092871	RFFL	17	33341759	33416338	ENSG00000143774	GUK1	1	228327663	228336685	0.0082763	6.7194	4.23E-07	0.04043488	0.013902	1.6089	0.11808	rs9915021	1.78E-10	0	NA	NA	Adipose_Subcutaneous
ENSG00000092871	RFFL	17	33341759	33416338	ENSG00000143774	GUK1	1	228327663	228336685	0.0082763	6.7194	4.23E-07	0.04043488	0.013902	1.6089	0.11808	rs9915021	1.78E-10	0	NA	NA	Artery_Tibial
ENSG00000172123	SLFN12	17	33738079	33760302	ENSG00000123908	AGO2	8	141541264	141645718	0.012081	3.931	4.24E-07	0.04050518	NA	NA	NA	rs225245	8.22E-19	0	NA	NA	Cells_EBV-transformed_lymphocytes
ENSG00000172123	SLFN12	17	33738079	33760302	ENSG00000123908	AGO2	8	141541264	141645718	0.012081	3.931	4.24E-07	0.04050518	NA	NA	NA	rs9915021	4.09E-08	0.00058148	NA	NA	Breast_Mammary_Tissue
ENSG00000172660	TAF15	17	34136459	34174246	ENSG00000144567	FAM134A	2	220042939	220050201	0.0082644	6.7097	4.34E-07	0.04122164	0.0205	3.1917	0.0041736	rs9915021	2.69E-24	0	NA	NA	Skin_Not_Sun_Exposed_Suprapubic
ENSG00000006125	AP2B1	17	33913918	34053436	ENSG00000198478	SH3BGRL2	6	80341000	80413372	0.0087359	6.0809	4.35E-07	0.04128584	0.016252	2.5194	0.020058	rs10512472	1.41E-46	0	3.62E-14	0	Heart_Left_Ventricle
ENSG00000154760	SLFN13	17	33762115	33775856	ENSG00000183508	FAM46C	1	118148556	118170994	0.010477	4.645	4.36E-07	0.0413178	0.0095587	0.73106	0.72172	rs10512472	6.42E-12	0	6.34E-38	0	Uterus
ENSG00000177666	PNPLA2	11	818902	825573	ENSG00000205038	PKHD1L1	8	110374706	110542559	0.0077659	7.5637	4.37E-07	0.04139155	0.010072	1.5516	0.1583	rs11602954	3.21E-08	0.00042188	NA	NA	Brain_Frontal_Cortex_BA9
ENSG00000177666	PNPLA2	11	818902	825573	ENSG00000205038	PKHD1L1	8	110374706	110542559	0.0077659	7.5637	4.37E-07	0.04139155	0.010072	1.5516	0.1583	rs17655730	2.44E-13	0	NA	NA	Brain_Frontal_Cortex_BA9
ENSG00000177666	PNPLA2	11	818902	825573	ENSG00000205038	PKHD1L1	8	110374706	110542559	0.0077659	7.5637	4.37E-07	0.04139155	0.010072	1.5516	0.1583	rs17655730	2.44E-13	0	NA	NA	Esophagus_Muscularis
ENSG00000172123	SLFN12	17	33738079	33760302	ENSG00000137193	PIM1	6	37137979	37143202	0.012057	3.9234	4.43E-07	0.04173891	0.015273	2.3653	0.028391	rs225245	2.86E-14	0	NA	NA	Cells_EBV-transformed_lymphocytes
ENSG00000174885	NLRP6	11	278365	285359	ENSG00000140479	PCSK6	15	101840818	102065405	0.0087272	6.0748	4.43E-07	0.04176023	0.025339	2.6344	0.0051725	rs11602954	1.74E-12	0	2.12E-70	0	Nerve_Tibial
ENSG00000174885	NLRP6	11	278365	285359	ENSG00000140479	PCSK6	15	101840818	102065405	0.0087272	6.0748	4.43E-07	0.04176023	0.025339	2.6344	0.0051725	rs17655730	3.01E-13	0	5.43E-117	0	Artery_Aorta
ENSG00000174885	NLRP6	11	278365	285359	ENSG00000140479	PCSK6	15	101840818	102065405	0.0087272	6.0748	4.43E-07	0.04176023	0.025339	2.6344	0.0051725	rs17655730	3.01E-13	0	5.43E-117	0	Nerve_Tibial
ENSG00000174885	NLRP6	11	278365	285359	ENSG00000140479	PCSK6	15	101840818	102065405	0.0087272	6.0748	4.43E-07	0.04176023	0.025339	2.6344	0.0051725	rs17655730	3.01E-13	0	5.43E-117	0	Skin_Sun_Exposed_Lower_leg
ENSG00000174885	NLRP6	11	278365	285359	ENSG00000140479	PCSK6	15	101840818	102065405	0.0087272	6.0748	4.43E-07	0.04176023	0.025339	2.6344	0.0051725	rs505404	3.18E-16	0	2.65E-88	0	Nerve_Tibial
ENSG00000174885	NLRP6	11	278365	285359	ENSG00000140479	PCSK6	15	101840818	102065405	0.0087272	6.0748	4.43E-07	0.04176023	0.025339	2.6344	0.0051725	rs505404	3.18E-16	0	2.65E-88	0	Prostate
ENSG00000198917	C9orf114	9	131586026	131592100	ENSG00000118113	MMP8	11	102582526	102597781	0.0072244	8.7924	4.54E-07	0.0423508	0.0078067	2.4076	0.065831	rs15676	1.65E-07	0.00162691	NA	NA	Breast_Mammary_Tissue
ENSG00000172123	SLFN12	17	33738079	33760302	ENSG00000120885	CLU	8	27454434	27472548	0.012038	3.9168	4.60E-07	0.04273093	0.018151	2.8191	0.01006	rs225245	1.24E-08	0.00017575	NA	NA	Cells_EBV-transformed_lymphocytes
ENSG00000172123	SLFN12	17	33738079	33760302	ENSG00000120885	CLU	8	27454434	27472548	0.012038	3.9168	4.60E-07	0.04273093	0.018151	2.8191	0.01006	rs9915021	2.20E-17	0	NA	NA	Breast_Mammary_Tissue
ENSG00000172123	SLFN12	17	33738079	33760302	ENSG00000196961	AP2A1	19	50270225	50309510	0.012038	3.9169	4.60E-07	0.04273093	0.009814	1.5115	0.17113	rs225245	1.75E-29	0	NA	NA	Cells_EBV-transformed_lymphocytes
ENSG00000172123	SLFN12	17	33738079	33760302	ENSG00000196961	AP2A1	19	50270225	50309510	0.012038	3.9169	4.60E-07	0.04273093	0.009814	1.5115	0.17113	rs9915021	1.56E-11	0	NA	NA	Breast_Mammary_Tissue
ENSG00000174915	PTDSS2	11	448268	491387	ENSG00000205038	PKHD1L1	8	110374706	110542559	0.011631	4.0541	4.78E-07	0.04378006	0.0041283	0.95035	0.43408	rs11602954	3.21E-08	0.00042188	5.60E-10	0	Thyroid
ENSG00000174915	PTDSS2	11	448268	491387	ENSG00000205038	PKHD1L1	8	110374706	110542559	0.011631	4.0541	4.78E-07	0.04378006	0.0041283	0.95035	0.43408	rs17655730	2.44E-13	0	5.39E-11	0	Artery_Tibial
ENSG00000174915	PTDSS2	11	448268	491387	ENSG00000205038	PKHD1L1	8	110374706	110542559	0.011631	4.0541	4.78E-07	0.04378006	0.0041283	0.95035	0.43408	rs505404	1.11E-15	0	1.98E-10	0	Brain_Cerebellar_Hemisphere

ENSG00000100890	KIAA0391	14	35593499	35593616	ENSG00000135929	CYP27A1	2	219646479	219680016	0.0095819	5.1899	4.82E-07	0.04404431	0.013665	1.4039	0.18174	rs2143950	1.26E-12	0	NA	NA	Artery_Tibial
ENSG00000100890	KIAA0391	14	35593499	35593616	ENSG00000135929	CYP27A1	2	219646479	219680016	0.0095819	5.1899	4.82E-07	0.04404431	0.013665	1.4039	0.18174	rs2143950	1.26E-12	0	NA	NA	Skin_Sun_Exposed_Lower_leg
ENSG00000100890	KIAA0391	14	35593499	35593616	ENSG00000135929	CYP27A1	2	219646479	219680016	0.0095819	5.1899	4.82E-07	0.04404431	0.013665	1.4039	0.18174	rs2143950	1.26E-12	0	NA	NA	Spleen
ENSG00000132139	GAS2L2	17	34071530	34079897	ENSG00000007541	PIGQ	16	619947	634136	0.0091394	5.5677	4.85E-07	0.0441955	0.010543	1.2161	0.28605	rs9915021	1.41E-07	0.00153214	NA	NA	Small_Intestine_Terminal_Ileum
ENSG00000100418	DESI1	22	41994032	42017100	ENSG00000175556	LONRF3	X	118108581	118156888	0.0086728	6.0366	4.99E-07	0.04515021	0.013765	1.5928	0.12275	rs4822024	1.77E-35	0	1.61E-36	0	Brain_Hippocampus
ENSG00000172123	SLFN12	17	33738079	33760302	ENSG00000166947	EPB42	15	43398423	43513481	0.01199	3.9011	5.04E-07	0.04558023	0.013899	2.1495	0.045738	rs225245	6.28E-40	0	NA	NA	Cells_EBV-transformed_lymphocytes
ENSG00000172123	SLFN12	17	33738079	33760302	ENSG00000166947	EPB42	15	43398423	43513481	0.01199	3.9011	5.04E-07	0.04558023	0.013899	2.1495	0.045738	rs9915021	3.48E-18	0	NA	NA	Breast_Mammary_Tissue
ENSG00000163823	CCR1	3	46243200	46249887	ENSG00000144290	SLC4A10	2	162480845	162841792	0.0081859	6.6454	5.16E-07	0.04621776	0.013532	2.0919	0.051824	rs13098911	6.97E-10	2.04E-05	1.81E-06	0.005352798	Cells_Transformed_fibroblasts
ENSG00000172716	SLFN11	17	33677324	33700720	ENSG00000196961	AP2A1	19	50270225	50309510	0.010798	4.3889	5.19E-07	0.04645673	0.011792	0.9039	0.54253	rs225245	1.75E-29	0	5.77E-07	0.001884469	Cells_Transformed_fibroblasts
ENSG00000172716	SLFN11	17	33677324	33700720	ENSG00000196961	AP2A1	19	50270225	50309510	0.010798	4.3889	5.19E-07	0.04645673	0.011792	0.9039	0.54253	rs8082605	6.57E-08	0.00085447	NA	NA	Adrenal_Gland
ENSG00000006125	AP2B1	17	33913918	34053436	ENSG00000204613	TRIM10	6	30119722	30128711	0.0086527	6.0224	5.21E-07	0.0465607	0.0073509	1.1293	0.34307	rs10512472	1.74E-11	0	3.62E-14	0	Heart_Left_Ventricle
ENSG00000174840	PDE12	3	57542003	57552571	ENSG00000172572	PDE3A	12	20522179	20837315	0.007164	8.7184	5.22E-07	0.04657013	NA	NA	NA	rs1354034	5.07E-54	0	NA	NA	Brain_Putamen_basal_ganglia
ENSG00000154768	C17orf50	17	34087916	34092098	ENSG00000160445	ZER1	9	131492065	131534693	0.0081756	6.6369	5.28E-07	0.04700549	0.010356	1.3663	0.21615	rs9915021	1.28E-09	5.83E-05	NA	NA	Pituitary
ENSG00000099849	RASSF7	11	560404	564021	ENSG00000108576	SLC6A4	17	28521337	28563020	0.0086444	6.0166	5.30E-07	0.04710167	0.020396	2.7186	0.008552	rs505404	9.44E-25	0	NA	NA	Brain_Frontal_Cortex_BA9
ENSG00000172123	SLFN12	17	33738079	33760302	ENSG00000103257	SLC7A5	16	87863629	87903094	0.011961	3.8916	5.33E-07	0.04726661	0.013208	2.0412	0.057804	rs225245	2.55E-09	7.35E-05	NA	NA	Cells_EBV-transformed_lymphocytes
ENSG00000009954	BAZ1B	7	72854728	72936608	ENSG00000163751	CPA3	3	148583043	148614983	0.0076625	7.4622	5.51E-07	0.04851522	0.0065098	1.2004	0.30697	rs17145738	1.15E-06	0.00915734	NA	NA	Heart_Atrial_Appendage
ENSG00000108733	PEX12	17	33901814	33905882	ENSG00000125257	ABCC4	13	95672083	95953687	0.0076534	7.4533	5.63E-07	0.04924434	0.0027843	0.5115	0.76772	rs9915021	1.01E-11	0	NA	NA	Brain_Caudate_basal_ganglia
ENSG00000108733	PEX12	17	33901814	33905882	ENSG00000125257	ABCC4	13	95672083	95953687	0.0076534	7.4533	5.63E-07	0.04924434	0.0027843	0.5115	0.76772	rs9915021	1.01E-11	0	NA	NA	Breast_Mammary_Tissue
ENSG00000108733	PEX12	17	33901814	33905882	ENSG00000125257	ABCC4	13	95672083	95953687	0.0076534	7.4533	5.63E-07	0.04924434	0.0027843	0.5115	0.76772	rs10512472	2.65E-18	0	1.06E-12	0	Brain_Caudate_basal_ganglia
ENSG00000243646	IL10RB	21	34638663	34669539	ENSG00000165949	IFI27	14	94571182	94583033	0.011542	4.0226	5.69E-07	0.04962985	0.0603	6.5025	5.19E-09	rs2834188	1.92E-55	0	1.27E-39	0	Brain_Anterior_cingulate_cortex_BA24
ENSG00000243646	IL10RB	21	34638663	34669539	ENSG00000165949	IFI27	14	94571182	94583033	0.011542	4.0226	5.69E-07	0.04962985	0.0603	6.5025	5.19E-09	rs2834188	1.92E-55	0	1.27E-39	0	Brain_Cerebellar_Hemisphere
ENSG00000243646	IL10RB	21	34638663	34669539	ENSG00000165949	IFI27	14	94571182	94583033	0.011542	4.0226	5.69E-07	0.04962985	0.0603	6.5025	5.19E-09	rs2834188	1.92E-55	0	1.27E-39	0	Brain_Frontal_Cortex_BA9
ENSG00000142102	ATHL1	11	289138	296107	ENSG00000138798	EGF	4	110834040	110933422	0.013411	3.447	5.74E-07	0.04989385	0.00015683	0.072074	0.93047	rs11602954	3.92E-10	2.13E-05	8.23E-62	0	Thyroid
ENSG00000142102	ATHL1	11	289138	296107	ENSG00000138798	EGF	4	110834040	110933422	0.013411	3.447	5.74E-07	0.04989385	0.00015683	0.072074	0.93047	rs17655730	3.08E-11	0	1.10E-69	0	Muscle_Skeletal
ENSG00000142102	ATHL1	11	289138	296107	ENSG00000138798	EGF	4	110834040	110933422	0.013411	3.447	5.74E-07	0.04989385	0.00015683	0.072074	0.93047	rs505404	2.92E-13	0	3.83E-63	0	Lung
