# Supplementary material for: Imputed gene associations identify replicable trans‐acting genes enriched in transcription pathways and complex traits
Source: Genet Epidemiol. 2019 Apr 4;43(6):596–608. doi: 10.1002/gepi.22205 (PMC6687523; doi:10.1002/gepi.22205)
Supplement: Supplementary file 9 — Supplementary Information [file GEPI-43-596-s009.docx]

**Trans-acting gene enrichment**

| Source | GeneSet | N | n | P-value | adjusted P | genes |
| --- | --- | --- | --- | --- | --- | --- |
| GO molecular functions (MSigDB v6.1 c5) | GO_NUCLEIC_ACID_BINDING_TRANSCRIPTION_FACTOR_ACTIVITY | 1000 | 33 | 6.40E-09 | 5.76E-06 | RFX5, ZNF438, ZNF641, ZSCAN10, ZNF200, ZNF263, ZNF174, TCF25, GATAD2A, ZFP14, ZFP82, ZNF260, ZNF382, ZNF790, ZNF345, ZNF829, ZNF568, ZNF585A, ZNF570, ZNF793, ZNF540, ZFP30, ZNF607, ZNF573, ZNF649, ZNF613, ZNF132, TEF, ZBTB11, REST, CITED2, PLAGL1, ZNF12 |
| Reactome (MSigDB v6.1 c2) | REACTOME_GENERIC_TRANSCRIPTION_PATHWAY | 292 | 15 | 2.18E-07 | 1.47E-04 | ZNF641, ZNF200, ZNF263, NCOR1, MED24, ZNF566, ZNF382, ZNF585A, ZNF569, ZNF570, ZNF540, ZNF607, ZNF649, ZNF613, ZNF12 |
| GWAS catalog (e91_r2018-02-06) | Reticulocyte count | 138 | 10 | 4.80E-07 | 5.88E-04 | FAM46C, OR2W3, BAZ1A, TCF25, SLFN12L, AP2B1, TAF15, SMOX, ARHGEF3, C6orf10 |
| GWAS catalog (e91_r2018-02-06) | Reticulocyte fraction of red cells | 147 | 9 | 6.76E-06 | 2.75E-03 | FAM46C, OR2W3, BAZ1A, TCF25, SLFN12L, AP2B1, TAF15, SMOX, ARHGEF3 |
| GWAS catalog (e91_r2018-02-06) | White blood cell count | 152 | 8 | 5.90E-05 | 1.57E-02 | FAM186A, PIGL, ORMDL3, GSDMA, MED24, ATAD2B, ITGA4, CERKL |
| GWAS catalog (e91_r2018-02-06) | Neuroticism | 134 | 7 | 1.44E-04 | 2.20E-02 | TEF, ACO2, POLR3H, CSDC2, PMM1, NHP2L1, MEI1 |
| GWAS catalog (e91_r2018-02-06) | Platelet count | 228 | 9 | 2.78E-04 | 3.40E-02 | FAM46C, OR2W3, BET1L, SCGB1C1, RIC8A, SIRT3, NLRP6, AP2B1, ARHGEF3 |
| GWAS catalog (e91_r2018-02-06) | Crohn's disease | 525 | 15 | 3.19E-04 | 3.54E-02 | ORMDL3, GSDMA, PUS10, USP34, ITGA4, SP110, IFNAR2, AP000295.9, IL10RB, TEF, PMM1, NHP2L1, IMPG2, SENP7, TNIP1, FIGNL1 |

**Target gene enrichment**

| Source | GeneSet | N | n | P-value | adjusted P | genes |
| --- | --- | --- | --- | --- | --- | --- |
| GO cellular components (MSigDB v6.1 c5) | GO_PLATELET_ALPHA_GRANULE | 48 | 12 | 8.40E-13 | 4.87E-10 | SELP, THBS1, ITGA2B, ITGB3, ITGB3, PF4, PPBP, MMRN1, EGF, SPARC, F13A1, TREML1, CLU |
| Reactome (MSigDB v6.1 c2) | REACTOME_HEMOSTASIS | 341 | 28 | 7.57E-13 | 5.10E-10 | SELP, VCL, MRVI1, PPP2R5B, ESAM, JAM3, THBS1, GNB5, SLC7A5, GP1BA, ITGA2B, ITGB3, ITGB3, PF4V1, PF4, PPBP, MMRN1, EGF, GUCY1A3, GUCY1B3, SPARC, F13A1, HIST1H3D, GNA12, GNG11, PRKAR2B, DOCK4, CLU, GATA1 |
| Reactome (MSigDB v6.1 c2) | REACTOME_RESPONSE_TO_ELEVATED_PLATELET_CYTOSOLIC_CA2_ | 58 | 12 | 1.18E-11 | 3.98E-09 | SELP, VCL, THBS1, ITGA2B, ITGB3, ITGB3, PF4, PPBP, MMRN1, EGF, SPARC, F13A1, CLU |
| GO cellular components (MSigDB v6.1 c5) | GO_SECRETORY_GRANULE | 224 | 21 | 3.33E-11 | 9.65E-09 | SELP, OLFM4, CTSG, THBS1, ITGA2B, ITGB3, ITGB3, ELANE, IL1B, GHRL, CPA3, PF4, PPBP, MMRN1, EGF, SPARC, F13A1, TREML1, CRISP3, DEFA4, CLU, SYTL4 |
| Reactome (MSigDB v6.1 c2) | REACTOME_PLATELET_ACTIVATION_SIGNALING_AND_AGGREGATION | 150 | 16 | 6.95E-10 | 1.56E-07 | SELP, VCL, THBS1, GNB5, GP1BA, ITGA2B, ITGB3, ITGB3, PF4, PPBP, MMRN1, EGF, SPARC, F13A1, GNA12, GNG11, CLU |
| GO cellular components (MSigDB v6.1 c5) | GO_SECRETORY_VESICLE | 295 | 22 | 1.20E-09 | 2.32E-07 | SELP, OLFM4, CTSG, STON2, THBS1, ITGA2B, ITGB3, ITGB3, ELANE, IL1B, GHRL, CPA3, PF4, PPBP, MMRN1, EGF, SPARC, F13A1, TREML1, CRISP3, DEFA4, CLU, SYTL4 |
| GO cellular components (MSigDB v6.1 c5) | GO_PLATELET_ALPHA_GRANULE_LUMEN | 32 | 8 | 2.11E-09 | 3.06E-07 | THBS1, PF4, PPBP, MMRN1, EGF, SPARC, F13A1, CLU |
| GO cellular components (MSigDB v6.1 c5) | GO_EXTRACELLULAR_SPACE | 705 | 35 | 2.75E-09 | 3.19E-07 | ALPL, MUC1, SELP, JAM3, OLFM4, RNASE3, CMTM5, CTSG, THBS1, PCSK6, IL4R, SERPINF1, ITGA2B, ELANE, LRG1, IL1B, BPI, SLPI, FAM3B, GHRL, CPA3, EIF2A, IL8, PF4V1, PF4, PPBP, EGF, SPARC, F13A1, BMP6, CRISP3, DEFA4, CLU, PKHD1L1, GSN |
| GO cellular components (MSigDB v6.1 c5) | GO_SECRETORY_GRANULE_LUMEN | 46 | 9 | 4.13E-09 | 3.99E-07 | THBS1, GHRL, PF4, PPBP, MMRN1, EGF, SPARC, F13A1, CLU |
| GO biological processes (MSigDB v6.1 c5) | GO_PLATELET_DEGRANULATION | 73 | 12 | 2.58E-10 | 1.14E-06 | SELP, VCL, THBS1, ITGA2B, ITGB3, ITGB3, PF4, PPBP, MMRN1, EGF, SPARC, F13A1, CLU |
| GO biological processes (MSigDB v6.1 c5) | GO_PLATELET_ACTIVATION | 110 | 14 | 5.32E-10 | 1.18E-06 | VCL, ILK, GP1BA, ITGA2B, ITGB3, ITGB3, GP6, MYL9, PF4V1, PF4, C6orf25, TREML1, GNA12, FZD6, GATA1 |
| GO biological processes (MSigDB v6.1 c5) | GO_HEMOSTASIS | 217 | 19 | 8.53E-10 | 1.26E-06 | VCL, ILK, GP1BA, ITGA2B, ITGB3, ITGB3, GP6, EHD3, MYL9, PF4V1, PF4, MMRN1, F13A1, HIST1H3D, C6orf25, TREML1, GNA12, PRKAR2B, FZD6, GATA1 |
| GO biological processes (MSigDB v6.1 c5) | GO_WOUND_HEALING | 321 | 23 | 1.19E-09 | 1.32E-06 | VCL, ILK, GP1BA, ITGA2B, ITGB3, ITGB3, GP6, EHD3, IL1B, MYL9, PF4V1, PF4, MMRN1, SPARC, F13A1, HIST1H3D, C6orf25, TREML1, GNA12, PRKAR2B, SDC2, FZD6, GSN, GATA1 |
| GO biological processes (MSigDB v6.1 c5) | GO_RESPONSE_TO_WOUNDING | 383 | 25 | 1.82E-09 | 1.61E-06 | VCL, ILK, JAM3, GP1BA, ITGA2B, ITGB3, ITGB3, GP6, EHD3, IL1B, MYL9, PF4V1, PF4, MMRN1, SPARC, F13A1, HIST1H3D, C6orf25, TREML1, GNA12, PRKAR2B, CLU, SDC2, FZD6, GSN, GATA1 |
| GO cellular components (MSigDB v6.1 c5) | GO_VESICLE_LUMEN | 57 | 9 | 3.71E-08 | 3.07E-06 | THBS1, GHRL, PF4, PPBP, MMRN1, EGF, SPARC, F13A1, CLU |
| GO cellular components (MSigDB v6.1 c5) | GO_CORTICAL_CYTOSKELETON | 58 | 9 | 4.42E-08 | 3.20E-06 | VCL, CTTN, SPTB, EPB42, CDH1, GYPC, CALD1, GSN, MPP1 |
| GO biological processes (MSigDB v6.1 c5) | GO_REGULATION_OF_LEUKOCYTE_MIGRATION | 103 | 12 | 2.04E-08 | 1.51E-05 | SELP, JAM3, OLFM4, THBS1, ITGA2B, PLVAP, IL1B, IL8, PF4V1, PF4, PPBP, MPP1 |
| GO biological processes (MSigDB v6.1 c5) | GO_REGULATION_OF_BODY_FLUID_LEVELS | 336 | 21 | 6.74E-08 | 4.27E-05 | SELP, VCL, ILK, THBS1, GP1BA, ITGA2B, ITGB3, ITGB3, GP6, EHD3, MYL9, PF4V1, PF4, MMRN1, F13A1, HIST1H3D, C6orf25, TREML1, GNA12, PRKAR2B, FZD6, GATA1 |
| GO cellular components (MSigDB v6.1 c5) | GO_CELL_CORTEX_PART | 83 | 9 | 1.42E-06 | 8.24E-05 | VCL, CTTN, SPTB, EPB42, CDH1, GYPC, CALD1, GSN, MPP1 |
| GO biological processes (MSigDB v6.1 c5) | GO_REGULATED_EXOCYTOSIS | 145 | 13 | 1.80E-07 | 9.98E-05 | SELP, VCL, THBS1, ITGA2B, ITGB3, ITGB3, PF4, PPBP, MMRN1, EGF, SPARC, F13A1, CLU, SYTL4 |
| GO cellular components (MSigDB v6.1 c5) | GO_EXTRACELLULAR_MATRIX | 219 | 14 | 5.28E-06 | 2.78E-04 | ALPL, NID1, ILK, CTSG, THBS1, PCSK6, SERPINF1, LTBP1, PI3, SLPI, FBN2, SPARC, CRISP3, CLU |
| GO biological processes (MSigDB v6.1 c5) | GO_POSITIVE_REGULATION_OF_LEUKOCYTE_MIGRATION | 77 | 9 | 7.00E-07 | 3.20E-04 | SELP, THBS1, ITGA2B, PLVAP, IL1B, IL8, PF4V1, PF4, PPBP |
| GO biological processes (MSigDB v6.1 c5) | GO_PLATELET_AGGREGATION | 31 | 6 | 7.21E-07 | 3.20E-04 | VCL, ILK, ITGA2B, ITGB3, ITGB3, MYL9, GATA1 |
| GO biological processes (MSigDB v6.1 c5) | GO_REGULATION_OF_LEUKOCYTE_CHEMOTAXIS | 63 | 8 | 1.12E-06 | 4.54E-04 | JAM3, THBS1, IL1B, IL8, PF4V1, PF4, PPBP, MPP1 |
| GO biological processes (MSigDB v6.1 c5) | GO_REGULATION_OF_NEUTROPHIL_MIGRATION | 23 | 5 | 1.85E-06 | 6.84E-04 | JAM3, OLFM4, IL1B, IL8, MPP1 |
| GO biological processes (MSigDB v6.1 c5) | GO_EXOCYTOSIS | 205 | 14 | 2.33E-06 | 7.65E-04 | SELP, VCL, THBS1, ITGA2B, ITGB3, ITGB3, PF4, PPBP, MMRN1, EGF, SPARC, F13A1, CLU, ANK1, SYTL4 |
| GO biological processes (MSigDB v6.1 c5) | GO_REGULATION_OF_GRANULOCYTE_CHEMOTAXIS | 24 | 5 | 2.43E-06 | 7.65E-04 | JAM3, THBS1, IL1B, IL8, MPP1 |
| GO biological processes (MSigDB v6.1 c5) | GO_HOMOTYPIC_CELL_CELL_ADHESION | 37 | 6 | 2.59E-06 | 7.65E-04 | VCL, ILK, ITGA2B, ITGB3, ITGB3, MYL9, GATA1 |
| GO cellular components (MSigDB v6.1 c5) | GO_ANCHORING_JUNCTION | 345 | 17 | 2.63E-05 | 1.27E-03 | VCL, PDLIM1, ILK, CTTN, CAPN5, ESAM, JAM3, TSPAN9, CDH1, ITGA2B, ITGB3, ITGB3, DSC1, EHD3, TNS1, PARVB, GNA12, GSN |
| GO biological processes (MSigDB v6.1 c5) | GO_DEFENSE_RESPONSE | 764 | 30 | 5.89E-06 | 1.63E-03 | TSPAN2, SH2D1B, SELP, JAM3, RNASE3, CTSG, IFI27, THBS1, IL4R, IRF8, ELANE, ECSIT, IL1B, BPI, SLPI, PROK2, CPA3, IL8, PF4V1, PF4, PPBP, BMP6, HIST1H3D, SRPK1, TREML1, TNFRSF21, CRISP3, DEFA4, CLU, PTGS1 |
| GO cellular components (MSigDB v6.1 c5) | GO_CELL_SURFACE | 467 | 20 | 5.04E-05 | 2.25E-03 | SELP, CAPN5, CTSG, SPTB, THBS1, PCSK6, GP1BA, ITGA2B, ITGB3, ITGB3, ELANE, PLVAP, GP6, CTLA4, TNS1, SPARC, TREML1, TSPAN33, CLU, SDC2, FZD6 |
| GO biological processes (MSigDB v6.1 c5) | GO_SECRETION_BY_CELL | 320 | 17 | 9.54E-06 | 2.47E-03 | SELP, VCL, THBS1, PCSK6, ITGA2B, ITGB3, ITGB3, FAM3B, GHRL, PF4, PPBP, MMRN1, EGF, SPARC, F13A1, CLU, ANK1, SYTL4 |
| Reactome (MSigDB v6.1 c2) | REACTOME_CELL_SURFACE_INTERACTIONS_AT_THE_VASCULAR_WALL | 70 | 7 | 2.44E-05 | 2.74E-03 | SELP, ESAM, JAM3, SLC7A5, ITGB3, ITGB3, PF4V1, PF4 |
| GO biological processes (MSigDB v6.1 c5) | GO_EXTRACELLULAR_MATRIX_DISASSEMBLY | 46 | 6 | 1.18E-05 | 2.76E-03 | NID1, CTSG, CDH1, ELANE, FBN2, GSN |
| GO biological processes (MSigDB v6.1 c5) | GO_EXTRACELLULAR_STRUCTURE_ORGANIZATION | 181 | 12 | 1.37E-05 | 2.92E-03 | NID1, ILK, JAM3, CTSG, THBS1, CDH1, ITGA2B, ITGB3, ITGB3, ELANE, FBN2, SPARC, GSN |
| GO biological processes (MSigDB v6.1 c5) | GO_RESPONSE_TO_LIPID | 606 | 25 | 1.38E-05 | 2.92E-03 | ALPL, PAQR7, SELP, HMBS, CTSG, THBS1, IL4R, IRF8, SERPINF1, SLC6A4, ELANE, IL1B, SLPI, GHRL, IL8, PF4V1, PF4, PPBP, ACSL1, SPARC, BMP6, PIM1, TNFRSF21, GDAP1, PTGS1 |
| Reactome (MSigDB v6.1 c2) | REACTOME_PLATELET_HOMEOSTASIS | 54 | 6 | 3.48E-05 | 3.35E-03 | MRVI1, PPP2R5B, GNB5, GUCY1A3, GUCY1B3, GNG11 |
| GO biological processes (MSigDB v6.1 c5) | GO_LEUKOCYTE_MIGRATION | 185 | 12 | 1.73E-05 | 3.49E-03 | SELP, ESAM, JAM3, SLC7A5, ITGB3, ITGB3, ELANE, GP6, IL1B, IL8, PF4V1, PF4, PPBP |
| GO molecular functions (MSigDB v6.1 c5) | GO_HEPARIN_BINDING | 83 | 8 | 1.16E-05 | 3.49E-03 | SELP, CTSG, THBS1, PCSK6, ELANE, PF4V1, PF4, C6orf25 |
| GO cellular components (MSigDB v6.1 c5) | GO_CELL_SUBSTRATE_JUNCTION | 279 | 14 | 9.02E-05 | 3.74E-03 | VCL, PDLIM1, ILK, CTTN, CAPN5, TSPAN9, CDH1, ITGA2B, ITGB3, ITGB3, EHD3, TNS1, PARVB, GNA12, GSN |
| GO cellular components (MSigDB v6.1 c5) | GO_APICAL_JUNCTION_COMPLEX | 85 | 7 | 1.00E-04 | 3.87E-03 | FRMD4A, PARD3, VCL, ESAM, JAM3, CDH1, RAP2C |
| GO cellular components (MSigDB v6.1 c5) | GO_CORTICAL_ACTIN_CYTOSKELETON | 45 | 5 | 1.09E-04 | 3.96E-03 | VCL, SPTB, CDH1, CALD1, GSN |
| GO biological processes (MSigDB v6.1 c5) | GO_DEFENSE_RESPONSE_TO_BACTERIUM | 113 | 9 | 2.32E-05 | 4.29E-03 | SELP, RNASE3, CTSG, IRF8, ELANE, BPI, SLPI, PPBP, DEFA4 |
| GWAS catalog reported genes (e91_r2018-02-06) | Reticulocyte fraction of red cells | 131 | 10 | 1.49E-05 | 4.56E-03 | PRTFDC1, RNF10, SPTB, NPRL3, CDH1, GP6, EHD3, SMOX, EGF, ANK1 |
| GO biological processes (MSigDB v6.1 c5) | GO_ENSHEATHMENT_OF_NEURONS | 71 | 7 | 2.71E-05 | 4.81E-03 | TSPAN2, PARD3, ILK, JAM3, TNFRSF21, KEL, CLU |
| GO biological processes (MSigDB v6.1 c5) | GO_NEGATIVE_REGULATION_OF_VASCULATURE_DEVELOPMENT | 54 | 6 | 3.48E-05 | 5.62E-03 | THBS1, SERPINF1, GHRL, PF4, SPARC, FOXO4 |
| GO biological processes (MSigDB v6.1 c5) | GO_REGULATION_OF_SMOOTH_MUSCLE_CONTRACTION | 37 | 5 | 3.49E-05 | 5.62E-03 | CTTN, PROK2, GUCY1A3, DOCK4, PTGS1 |
| GO biological processes (MSigDB v6.1 c5) | GO_REGULATION_OF_CHEMOTAXIS | 119 | 9 | 3.64E-05 | 5.62E-03 | JAM3, THBS1, ELANE, IL1B, IL8, PF4V1, PF4, PPBP, MPP1 |
| GO biological processes (MSigDB v6.1 c5) | GO_REGULATION_OF_CELLULAR_COMPONENT_MOVEMENT | 529 | 22 | 3.67E-05 | 5.62E-03 | SELP, VCL, ILK, JAM3, OLFM4, THBS1, SERPINF1, ITGA2B, ITGB3, ITGB3, PLVAP, IL1B, IL8, PF4V1, PF4, PPBP, EGF, SPARC, GNA12, DOCK4, GSN, RAP2C, MPP1 |
| GO biological processes (MSigDB v6.1 c5) | GO_REGULATION_OF_CELLULAR_RESPONSE_TO_GROWTH_FACTOR_STIMULUS | 145 | 10 | 3.86E-05 | 5.62E-03 | ILK, PPP2R5B, PPM1A, THBS1, PCSK6, ITGB3, ITGB3, LRG1, LTBP1, IL1B, FBN2 |
| GO biological processes (MSigDB v6.1 c5) | GO_POSITIVE_REGULATION_OF_LEUKOCYTE_CHEMOTAXIS | 55 | 6 | 3.93E-05 | 5.62E-03 | THBS1, IL1B, IL8, PF4V1, PF4, PPBP |
| GO biological processes (MSigDB v6.1 c5) | GO_RESPONSE_TO_BACTERIUM | 326 | 16 | 4.47E-05 | 6.19E-03 | ALPL, SELP, RNASE3, CTSG, IRF8, ELANE, IL1B, BPI, SLPI, IL8, PF4V1, PF4, PPBP, SPARC, TNFRSF21, DEFA4 |
| GO molecular functions (MSigDB v6.1 c5) | GO_EXTRACELLULAR_MATRIX_BINDING | 36 | 5 | 2.97E-05 | 6.68E-03 | NID1, THBS1, ITGA2B, ITGB3, ITGB3, SPARC |
| GO molecular functions (MSigDB v6.1 c5) | GO_CALCIUM_ION_BINDING | 388 | 18 | 3.74E-05 | 6.74E-03 | SLC25A24, NID1, PRRG4, VWCE, THBS1, CDH1, DSC1, CABP5, EHD3, LTBP1, SLC24A3, MYL9, MMRN1, EGF, FBN2, SPARC, GSN, SYTL4 |
| GO molecular functions (MSigDB v6.1 c5) | GO_CYTOKINE_ACTIVITY | 100 | 8 | 5.24E-05 | 6.74E-03 | CMTM5, IL1B, FAM3B, IL8, PF4V1, PF4, PPBP, BMP6 |
| GO cellular components (MSigDB v6.1 c5) | GO_CELL_CORTEX | 175 | 10 | 2.09E-04 | 6.75E-03 | PARD3, VCL, CTTN, SPTB, EPB42, CDH1, GYPC, CALD1, GSN, MPP1 |
| GO biological processes (MSigDB v6.1 c5) | GO_CELL_MATRIX_ADHESION | 79 | 7 | 5.92E-05 | 6.76E-03 | NID1, VCL, ILK, CTTN, JAM3, ITGA2B, ITGB3, ITGB3 |
| GO biological processes (MSigDB v6.1 c5) | GO_REGULATION_OF_TRANSMEMBRANE_RECEPTOR_PROTEIN_SERINE_THREONINE_KINASE_SIGNALING_PATHWAY | 126 | 9 | 5.95E-05 | 6.76E-03 | ILK, DACT1, PPM1A, THBS1, PCSK6, LRG1, LTBP1, FBN2, BMP6 |
| GO biological processes (MSigDB v6.1 c5) | GO_IMMUNE_RESPONSE | 702 | 26 | 6.37E-05 | 7.06E-03 | SH2D1B, JAM3, RNASE3, CTSG, IFI27, THBS1, IL4R, IRF8, ELANE, ECSIT, IL1B, CTLA4, BPI, SLPI, IL8, PF4V1, PF4, PPBP, BMP6, SRPK1, TREML1, TNFRSF21, CRISP3, DEFA4, CLU, PKHD1L1 |
| GO biological processes (MSigDB v6.1 c5) | GO_REGULATION_OF_LIPID_TRANSPORT | 60 | 6 | 6.96E-05 | 7.53E-03 | THBS1, ITGB3, ITGB3, IL1B, GHRL, EGF, BMP6 |
| GO biological processes (MSigDB v6.1 c5) | GO_RESPONSE_TO_MOLECULE_OF_BACTERIAL_ORIGIN | 244 | 13 | 7.79E-05 | 7.85E-03 | ALPL, SELP, CTSG, IRF8, ELANE, IL1B, SLPI, IL8, PF4V1, PF4, PPBP, SPARC, TNFRSF21 |
| GO biological processes (MSigDB v6.1 c5) | GO_INFLAMMATORY_RESPONSE | 309 | 15 | 8.18E-05 | 8.07E-03 | TSPAN2, SELP, JAM3, THBS1, IL4R, ELANE, IL1B, PROK2, IL8, PF4V1, PF4, PPBP, BMP6, TNFRSF21, PTGS1 |
| GO biological processes (MSigDB v6.1 c5) | GO_SECRETION | 379 | 17 | 8.93E-05 | 8.61E-03 | SELP, VCL, THBS1, PCSK6, ITGA2B, ITGB3, ITGB3, FAM3B, GHRL, PF4, PPBP, MMRN1, EGF, SPARC, F13A1, CLU, ANK1, SYTL4 |
| GO cellular components (MSigDB v6.1 c5) | GO_SECRETORY_GRANULE_MEMBRANE | 54 | 5 | 3.05E-04 | 9.30E-03 | SELP, ITGA2B, ITGB3, ITGB3, SPARC, SYTL4 |
| GWAS catalog reported genes (e91_r2018-02-06) | High light scatter reticulocyte percentage of red cells | 125 | 9 | 5.56E-05 | 9.71E-03 | PRTFDC1, SPTB, NPRL3, CDH1, EHD3, TNS1, SMOX, KEL, ANK1 |
| GO cellular components (MSigDB v6.1 c5) | GO_INTRACELLULAR_VESICLE | 863 | 28 | 3.49E-04 | 1.01E-02 | SELP, NRGN, OLFM4, CTSG, STON2, THBS1, SERPINF1, ITGA2B, ITGB3, ITGB3, ELANE, YIPF2, EHD3, IL1B, CTLA4, GHRL, CPA3, PF4, PPBP, MMRN1, EGF, SPARC, F13A1, TREML1, CRISP3, DEFA4, CLU, FZD6, SYTL4 |
| GO biological processes (MSigDB v6.1 c5) | GO_REGULATION_OF_VASCULATURE_DEVELOPMENT | 165 | 10 | 1.25E-04 | 1.13E-02 | THBS1, SERPINF1, LRG1, IL1B, GHRL, PROK2, IL8, PF4, SPARC, FOXO4 |
| GO biological processes (MSigDB v6.1 c5) | GO_CELL_ACTIVATION | 429 | 18 | 1.42E-04 | 1.26E-02 | SH2D1B, VCL, ILK, GP1BA, ITGA2B, ITGB3, ITGB3, GP6, MYL9, IL8, PF4V1, PF4, PPBP, C6orf25, TREML1, GNA12, CLU, FZD6, GATA1 |
| GWAS catalog reported genes (e91_r2018-02-06) | High light scatter reticulocyte count | 131 | 9 | 8.27E-05 | 1.26E-02 | PRTFDC1, SPTB, CDH1, EHD3, TNS1, CYP27A1, SMOX, KEL, ANK1 |
| TF targets (MSigDB v6.1 c3) | WGGAATGY_TEF1_Q6 | 247 | 14 | 2.23E-05 | 1.37E-02 | NID1, VCL, PRRG4, TSPAN9, PTMS, DACT1, SPTB, ROGDI, GHRL, PROK2, SPARC, SH3BGRL2, FBXL13, TSPAN33 |
| GO cellular components (MSigDB v6.1 c5) | GO_BLOOD_MICROPARTICLE | 61 | 5 | 5.93E-04 | 1.46E-02 | ITGA2B, EIF2A, F13A1, CLU, GSN |
| GO cellular components (MSigDB v6.1 c5) | GO_LAMELLIPODIUM | 138 | 8 | 6.04E-04 | 1.46E-02 | ILK, CTTN, CDH1, ITGB3, ITGB3, PARVB, ABLIM3, TIAM2, GSN |
| GO molecular functions (MSigDB v6.1 c5) | GO_GLYCOSAMINOGLYCAN_BINDING | 114 | 8 | 1.45E-04 | 1.64E-02 | SELP, CTSG, THBS1, PCSK6, ELANE, PF4V1, PF4, C6orf25 |
| GO molecular functions (MSigDB v6.1 c5) | GO_G_PROTEIN_COUPLED_RECEPTOR_BINDING | 143 | 9 | 1.71E-04 | 1.72E-02 | ARHGEF12, GNAZ, GHRL, PROK2, IL8, PF4V1, PF4, PPBP, GNA12 |
| GO biological processes (MSigDB v6.1 c5) | GO_RESPONSE_TO_VITAMIN | 71 | 6 | 2.04E-04 | 1.74E-02 | ALPL, HMBS, IL1B, SPARC, PIM1, GSN |
| Reactome (MSigDB v6.1 c2) | REACTOME_G_ALPHA_I_SIGNALLING_EVENTS | 100 | 7 | 3.11E-04 | 1.75E-02 | HEBP1, GNB5, GNAZ, IL8, PF4, PPBP, GNG11 |
| GO biological processes (MSigDB v6.1 c5) | GO_REGULATION_OF_ENDOTHELIAL_CELL_PROLIFERATION | 72 | 6 | 2.23E-04 | 1.78E-02 | THBS1, ITGB3, ITGB3, LRG1, GHRL, SPARC, BMP6 |
| GO biological processes (MSigDB v6.1 c5) | GO_RESPONSE_TO_BIOTIC_STIMULUS | 560 | 21 | 2.37E-04 | 1.78E-02 | ALPL, SELP, RNASE3, CTSG, IL4R, IRF8, ELANE, IL1B, BPI, SLPI, CPA3, IL8, PF4V1, PF4, PPBP, GUCY1A3, SPARC, HIST1H3D, TNFRSF21, DEFA4, CLU |
| GO biological processes (MSigDB v6.1 c5) | GO_IMMUNE_SYSTEM_PROCESS | 1377 | 40 | 2.59E-04 | 1.78E-02 | TSPAN2, SH2D1B, SELP, POLL, ESAM, JAM3, PTMS, RNASE3, CTSG, STON2, IFI27, THBS1, EPB42, ROGDI, IL4R, IRF8, SLC7A5, ITGB3, ITGB3, ELANE, ECSIT, GP6, IL1B, CTLA4, BPI, SLPI, IL8, PF4V1, PF4, PPBP, BMP6, BAG6, C6orf25, SRPK1, TREML1, TNFRSF21, CRISP3, DEFA4, CLU, PKHD1L1, GATA1 |
| GO biological processes (MSigDB v6.1 c5) | GO_G_PROTEIN_COUPLED_RECEPTOR_SIGNALING_PATHWAY | 378 | 16 | 2.69E-04 | 1.78E-02 | PARD3, ARHGEF12, GPRC5D, GNB5, GP1BA, GNAZ, GHRL, PROK2, IL8, PF4V1, PF4, PPBP, GPR146, GNA12, GNG11, FZD6 |
| GO biological processes (MSigDB v6.1 c5) | GO_GLIAL_CELL_DEVELOPMENT | 53 | 5 | 2.75E-04 | 1.78E-02 | TSPAN2, PARD3, ILK, CLU, GSN |
| GO biological processes (MSigDB v6.1 c5) | GO_RESPONSE_TO_ACID_CHEMICAL | 211 | 11 | 2.75E-04 | 1.78E-02 | HMBS, AARS, SERPINF1, SLC6A4, IL1B, ACSL1, SPARC, BMP6, GDAP1, GSN, PTGS1 |
| GO biological processes (MSigDB v6.1 c5) | GO_MULTICELLULAR_ORGANISM_REPRODUCTION | 452 | 18 | 2.77E-04 | 1.78E-02 | PAQR7, MICALCL, CAPN5, JAM3, SPATA7, IL4R, SERPINF1, SLC6A4, IL1B, OSBP2, LIMK2, GHRL, PROK2, CCNI, BAG6, SRPK1, SLC26A8, CLU |
| Reactome (MSigDB v6.1 c2) | REACTOME_INTEGRIN_CELL_SURFACE_INTERACTIONS | 58 | 5 | 4.51E-04 | 1.90E-02 | JAM3, THBS1, CDH1, ITGA2B, ITGB3, ITGB3 |
| Reactome (MSigDB v6.1 c2) | REACTOME_G_ALPHA1213_SIGNALLING_EVENTS | 58 | 5 | 4.51E-04 | 1.90E-02 | ARHGEF12, GNB5, TIAM2, GNA12, GNG11 |
| GO cellular components (MSigDB v6.1 c5) | GO_CYTOPLASMIC_REGION | 208 | 10 | 9.04E-04 | 2.02E-02 | PARD3, VCL, CTTN, SPTB, EPB42, CDH1, GYPC, CALD1, GSN, MPP1 |
| GO cellular components (MSigDB v6.1 c5) | GO_CYTOPLASMIC_VESICLE_PART | 386 | 15 | 9.82E-04 | 2.11E-02 | SELP, NRGN, THBS1, ITGA2B, ITGB3, ITGB3, GHRL, PF4, PPBP, MMRN1, EGF, SPARC, F13A1, CLU, FZD6, SYTL4 |
| GWAS catalog reported genes (e91_r2018-02-06) | Reticulocyte count | 120 | 8 | 2.15E-04 | 2.19E-02 | PRTFDC1, SPTB, CDH1, GP6, CYP27A1, SMOX, EGF, ANK1 |
| GWAS catalog reported genes (e91_r2018-02-06) | Monocyte count | 125 | 8 | 2.92E-04 | 2.23E-02 | PRTFDC1, IRF8, NFIX, SSBP4, FAM134A, PROK2, ANKRD55, GNA12 |
| GO cellular components (MSigDB v6.1 c5) | GO_EXTRACELLULAR_MATRIX_COMPONENT | 70 | 5 | 1.23E-03 | 2.26E-02 | NID1, SERPINF1, LTBP1, FBN2, SPARC |
| GO biological processes (MSigDB v6.1 c5) | GO_POSITIVE_REGULATION_OF_CHEMOTAXIS | 80 | 6 | 4.29E-04 | 2.41E-02 | THBS1, IL1B, IL8, PF4V1, PF4, PPBP |
| GO biological processes (MSigDB v6.1 c5) | GO_RESPONSE_TO_NUTRIENT | 133 | 8 | 4.62E-04 | 2.56E-02 | ALPL, HMBS, SLC6A4, IL1B, ACSL1, SPARC, PIM1, GSN |
| GO biological processes (MSigDB v6.1 c5) | GO_POSITIVE_REGULATION_OF_LOCOMOTION | 290 | 13 | 4.67E-04 | 2.56E-02 | SELP, ILK, THBS1, ITGA2B, ITGB3, ITGB3, PLVAP, IL1B, IL8, PF4V1, PF4, PPBP, SPARC, DOCK4 |
| GO biological processes (MSigDB v6.1 c5) | GO_CELLULAR_RESPONSE_TO_INORGANIC_SUBSTANCE | 108 | 7 | 5.23E-04 | 2.76E-02 | SLC25A24, HMBS, CDH1, SERPINF1, GUCY1B3, BMP6, GSN |
| GO biological processes (MSigDB v6.1 c5) | GO_REGULATION_OF_ANATOMICAL_STRUCTURE_MORPHOGENESIS | 717 | 24 | 5.40E-04 | 2.76E-02 | PINK1, ILK, CTTN, OLFM4, DACT1, THBS1, EPB42, IL4R, SERPINF1, LRG1, ZNF135, IL1B, PARVB, GHRL, PROK2, IL8, PF4, SPARC, GNA12, KEL, SDC2, FZD6, GSN, FOXO4 |
| GO biological processes (MSigDB v6.1 c5) | GO_RESPONSE_TO_EXTERNAL_STIMULUS | 1152 | 34 | 5.48E-04 | 2.76E-02 | ALPL, SELP, HMBS, RNASE3, CMTM5, CTSG, SPTB, NPRL3, IL4R, IRF8, SLC6A4, ITGB3, ITGB3, ELANE, IL1B, BPI, SLPI, GHRL, PROK2, CPA3, IL8, PF4V1, PF4, PPBP, GUCY1A3, ACSL1, SPARC, BMP6, HIST1H3D, PIM1, TNFRSF21, DOCK4, DEFA4, CLU, GSN |
| GO biological processes (MSigDB v6.1 c5) | GO_PROTEIN_TARGETING_TO_MEMBRANE | 61 | 5 | 5.93E-04 | 2.96E-02 | PARD3, SRP54, EHD3, SSR3, ANK1 |
| GWAS catalog reported genes (e91_r2018-02-06) | Mean platelet volume | 192 | 10 | 4.64E-04 | 3.02E-02 | PDLIM1, MRVI1, ENDOD1, ST3GAL4, GP1BA, ITGA2B, ITGB3, ITGB3, GP6, EHD3, SLC24A3 |
| GO biological processes (MSigDB v6.1 c5) | GO_RESPONSE_TO_OXYGEN_CONTAINING_COMPOUND | 937 | 29 | 6.16E-04 | 3.03E-02 | ALPL, SELP, HMBS, CTSG, THBS1, CDH1, AARS, IRF8, SERPINF1, SLC6A4, ELANE, IL1B, SLPI, IL8, PF4V1, PF4, PPBP, GUCY1B3, ACSL1, SPARC, BMP6, PIM1, TNFRSF21, GNG11, PRKAR2B, GDAP1, GSN, PTGS1, FOXO4 |
| Reactome (MSigDB v6.1 c2) | REACTOME_PEPTIDE_LIGAND_BINDING_RECEPTORS | 69 | 5 | 1.14E-03 | 3.21E-02 | HEBP1, GHRL, IL8, PF4, PPBP |
| GO biological processes (MSigDB v6.1 c5) | GO_CELL_SUBSTRATE_ADHESION | 113 | 7 | 7.08E-04 | 3.38E-02 | NID1, VCL, ILK, CTTN, JAM3, ITGA2B, ITGB3, ITGB3 |
| GO biological processes (MSigDB v6.1 c5) | GO_CELL_JUNCTION_ASSEMBLY | 88 | 6 | 7.64E-04 | 3.50E-02 | PARD3, VCL, ILK, CTTN, ITGB3, ITGB3, TNS1 |
| GO cellular components (MSigDB v6.1 c5) | GO_NEURON_PART | 838 | 25 | 2.26E-03 | 3.55E-02 | ESPN, PINK1, PARD3, ILK, CTTN, HMBS, NRGN, STON2, GNB5, SERPINF1, SLC6A4, GNAZ, OSBP2, GHRL, TNFRSF21, TIAM2, PRKAR2B, DOCK4, CLU, ANK1, SDC2, SHARPIN, PTGS1, SYTL4, MPP1 |
| Reactome (MSigDB v6.1 c2) | REACTOME_SIGNALING_BY_GPCR | 328 | 13 | 1.54E-03 | 3.77E-02 | ARHGEF12, HEBP1, GNB5, GNAZ, GHRL, IL8, PF4, PPBP, TIAM2, GNA12, GNG11, PRKAR2B, FZD6 |
| Reactome (MSigDB v6.1 c2) | REACTOME_GPCR_DOWNSTREAM_SIGNALING | 259 | 11 | 1.68E-03 | 3.84E-02 | ARHGEF12, HEBP1, GNB5, GNAZ, GHRL, IL8, PF4, PPBP, TIAM2, GNA12, GNG11 |
| GO biological processes (MSigDB v6.1 c5) | GO_NEGATIVE_REGULATION_OF_APOPTOTIC_SIGNALING_PATHWAY | 146 | 8 | 9.06E-04 | 3.88E-02 | PINK1, MUC1, CTTN, THBS1, IL1B, PF4, CLU, GATA1 |
| GO biological processes (MSigDB v6.1 c5) | GO_REGULATION_OF_MUSCLE_CONTRACTION | 91 | 6 | 9.33E-04 | 3.88E-02 | CTTN, MYL9, PROK2, GUCY1A3, DOCK4, PTGS1 |
| GO biological processes (MSigDB v6.1 c5) | GO_RESPONSE_TO_CORTICOSTEROID | 118 | 7 | 9.41E-04 | 3.88E-02 | ALPL, HMBS, SERPINF1, IL1B, SPARC, BMP6, PTGS1 |
| GO cellular components (MSigDB v6.1 c5) | GO_CELL_JUNCTION | 758 | 23 | 2.58E-03 | 3.94E-02 | FRMD4A, PARD3, VCL, PDLIM1, ILK, CTTN, CAPN5, ESAM, JAM3, TSPAN9, DACT1, STON2, CDH1, ITGA2B, ITGB3, ITGB3, DSC1, EHD3, TNS1, PARVB, GNA12, SHARPIN, GSN, RAP2C |
| GO biological processes (MSigDB v6.1 c5) | GO_CELL_JUNCTION_ORGANIZATION | 120 | 7 | 1.05E-03 | 4.12E-02 | PARD3, VCL, ILK, CTTN, CDH1, ITGB3, ITGB3, TNS1 |
| GO biological processes (MSigDB v6.1 c5) | GO_GLIAL_CELL_DIFFERENTIATION | 94 | 6 | 1.13E-03 | 4.40E-02 | TSPAN2, PARD3, ILK, HMBS, CLU, GSN |
| GO biological processes (MSigDB v6.1 c5) | GO_REGULATION_OF_CELLULAR_RESPONSE_TO_TRANSFORMING_GROWTH_FACTOR_BETA_STIMULUS | 69 | 5 | 1.14E-03 | 4.40E-02 | PPM1A, THBS1, LRG1, LTBP1, FBN2 |
| GO biological processes (MSigDB v6.1 c5) | GO_ESTABLISHMENT_OF_PROTEIN_LOCALIZATION_TO_MEMBRANE | 151 | 8 | 1.15E-03 | 4.40E-02 | PARD3, SRP54, CDH1, EHD3, SSR3, BAG6, TSPAN33, ANK1 |
| Reactome (MSigDB v6.1 c2) | REACTOME_AXON_GUIDANCE | 171 | 8 | 2.71E-03 | 4.52E-02 | ARHGEF12, SPTB, ITGA2B, ITGB3, ITGB3, MYL9, LIMK2, ABLIM3, ANK1 |
| GO biological processes (MSigDB v6.1 c5) | GO_POSITIVE_REGULATION_OF_NUCLEOCYTOPLASMIC_TRANSPORT | 96 | 6 | 1.28E-03 | 4.69E-02 | DACT1, PPM1A, CDH1, IL1B, EGF, BMP6 |
| GO molecular functions (MSigDB v6.1 c5) | GO_SULFUR_COMPOUND_BINDING | 143 | 8 | 7.81E-04 | 4.77E-02 | SELP, CTSG, THBS1, PCSK6, ELANE, PF4V1, PF4, C6orf25 |
| TF targets (MSigDB v6.1 c3) | PAX8_B | 68 | 6 | 1.56E-04 | 4.79E-02 | TSPAN2, NRGN, PPM1A, ABLIM3, CALD1, BNC2 |
| GWAS catalog reported genes (e91_r2018-02-06) | Hip circumference adjusted for BMI | 70 | 5 | 1.23E-03 | 4.87E-02 | PPM1A, NPRL3, LTBP1, BMP6, GNA12 |
