# Supplementary material for: Imputed gene associations identify replicable trans‐acting genes enriched in transcription pathways and complex traits
Source: Genet Epidemiol. 2019 Apr 4;43(6):596–608. doi: 10.1002/gepi.22205 (PMC6687523; doi:10.1002/gepi.22205)
Supplement: Supplementary file 4 — Supplementary Information [file GEPI-43-596-s004.pdf]

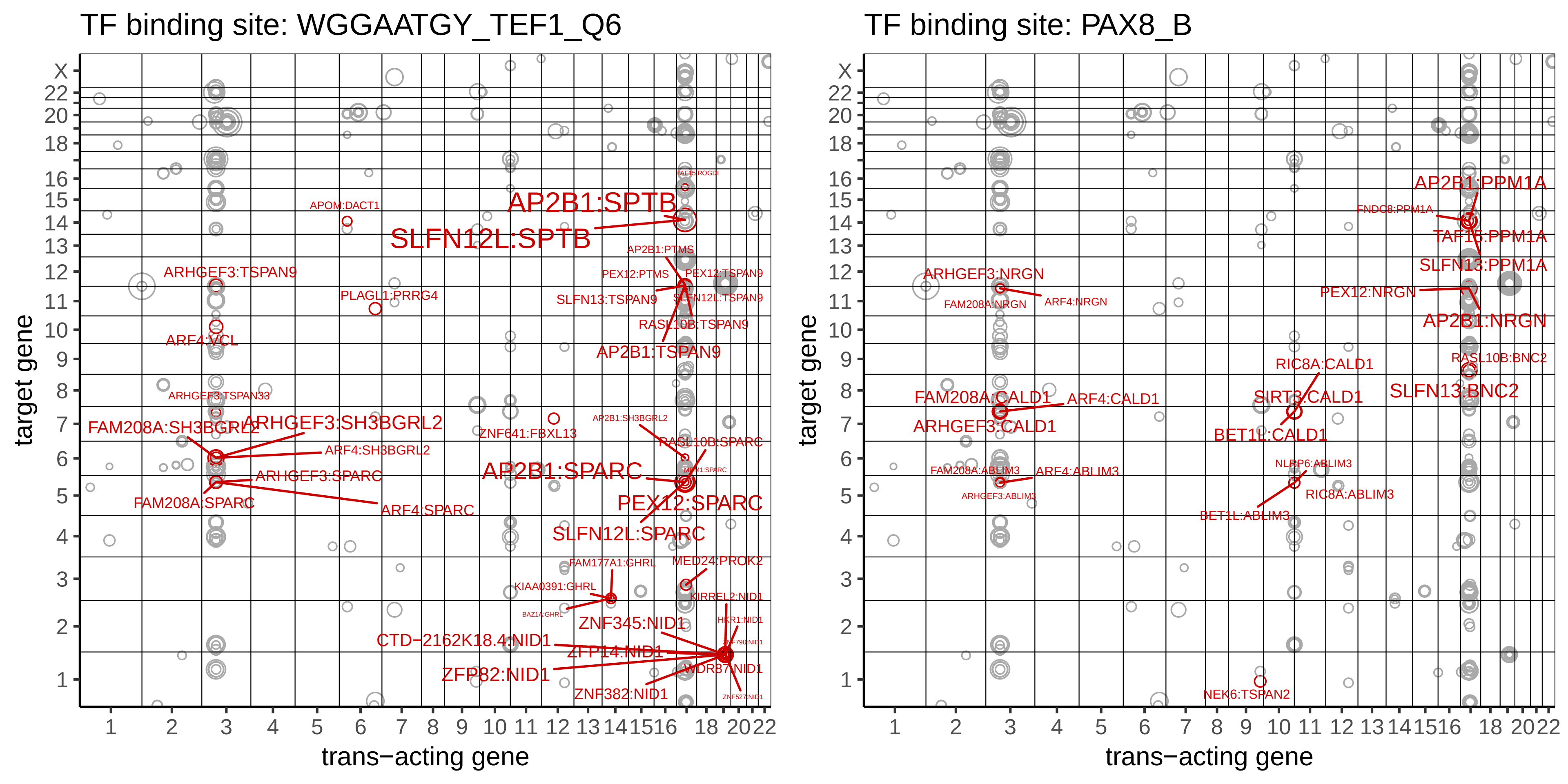

TF binding site: WGGAATGY_TEF1_Q6
target gene
trans−acting gene
AP2B1:SPTB
SLFN12L:SPTB
TAF15:ROGDI
APOM:DACT1
AP2B1:PTMS
PEX12:PTMS
PEX12:TSPAN9
SLFN13:TSPAN9
SLFN12L:TSPAN9
RASL10B:TSPAN9
AP2B1:TSPAN9
ARHGEF3:TSPAN9
PLAGL1:PRRG4
ARF4:VCL
ARHGEF3:TSPAN33
FAM208A:SH3BGRL2
ARHGEF3:SH3BGRL2
ARF4:SH3BGRL2
ZNF641:FBXL13
AP2B1:SH3BGRL2
RASL10B:SPARC
MFN1:SPARC
AP2B1:SPARC
ARHGEF3:SPARC
FAM208A:SPARC
ARF4:SPARC
PEX12:SPARC
SLFN12L:SPARC
FAM177A1:GHRL
MED24:PROK2
KIAA0391:GHRL
KIRREL2:NID1
BAZ1A:GHRL
ZNF345:NID1
HKR1:NID1
CTD−2162K18.4:NID1
ZNF790:NID1
ZFP14:NID1
ZFP82:NID1
WDR87:NID1
ZNF382:NID1
ZNF527:NID1
TF binding site: PAX8_B
target gene
trans−acting gene
AP2B1:PPM1A
FNDC8:PPM1A
TAF15:PPM1A
SLFN13:PPM1A
ARHGEF3:NRGN
FAM208A:NRGN
ARF4:NRGN
PEX12:NRGN
AP2B1:NRGN
RASL10B:BNC2
RIC8A:CALD1
SLFN13:BNC2
FAM208A:CALD1
ARF4:CALD1
SIRT3:CALD1
ARHGEF3:CALD1
BET1L:CALD1
NLRP6:ABLIM3
FAM208A:ABLIM3
ARF4:ABLIM3
ARHGEF3:ABLIM3
RIC8A:ABLIM3
BET1L:ABLIM3
NEK6:TSPAN2
